# Development of vegetative oil sorghum: From lab‐to‐field

**DOI:** 10.1111/pbi.14527

**Published:** 2024-11-30

**Authors:** Kiyoul Park, Truyen Quach, Teresa J. Clark, Hyojin Kim, Tieling Zhang, Mengyuan Wang, Ming Guo, Shirley Sato, Tara J. Nazarenus, Rostislav Blume, Yaroslav Blume, Chi Zhang, Stephen P. Moose, Kankshita Swaminathan, Jörg Schwender, Thomas Elmo Clemente, Edgar B. Cahoon

**Affiliations:** ^1^ Center for Plant Science Innovation University of Nebraska‐Lincoln Lincoln NE USA; ^2^ Department of Biochemistry University of Nebraska‐Lincoln Lincoln NE USA; ^3^ Biology Department Brookhaven National Laboratory Upton NY USA; ^4^ Plant Transformation Core Research Facility, Agricultural Research Division, Institute of Agriculture and Natural Resources University of Nebraska‐Lincoln Lincoln NE USA; ^5^ Institute of Food Biotechnology and Genomics National Academy of Sciences of Ukraine Kyiv Ukraine; ^6^ School of Biological Sciences University of Nebraska‐Lincoln Lincoln NE USA; ^7^ Department of Crop Sciences University of Illinois Urbana‐Champaign Urbana IL USA; ^8^ HudsonAlpha Institute for Biotechnology Huntsville AL USA; ^9^ Department of Agronomy & Horticulture University of Nebraska‐Lincoln Lincoln NE USA

**Keywords:** vegetative oils, triacylglycerol, fatty acids, renewable fuels, biomass feedstocks

## Abstract

Biomass crops engineered to accumulate energy‐dense triacylglycerols (TAG or ‘vegetable oils’) in their vegetative tissues have emerged as potential feedstocks to meet the growing demand for renewable diesel and sustainable aviation fuel (SAF). Unlike oil palm and oilseed crops, the current commercial sources of TAG, vegetative tissues, such as leaves and stems, only transiently accumulate TAG. In this report, we used grain (Texas430 or TX430) and sugar‐accumulating ‘sweet’ (Ramada) genotypes of sorghum, a high‐yielding, environmentally resilient biomass crop, to accumulate TAG in leaves and stems. We initially tested several gene combinations for a ‘push‐pull‐protect’ strategy. The top TAG‐yielding constructs contained five oil transgenes for a sorghum WRINKLED1 transcription factor (‘push’), a *Cuphea viscosissima* diacylglycerol acyltransferase (DGAT; ‘pull’), a modified sesame oleosin (‘protect’) and two combinations of specialized Cuphea lysophosphatidic acid acyltransferases and medium‐chain acyl‐acyl carrier protein thioesterases. Though intended to generate oils with medium‐chain fatty acids, engineered lines accumulated oleic acid‐rich oil to amounts of up to 2.5% DW in leaves and 2.0% DW in stems in the greenhouse, 36‐fold and 49‐fold increases relative to wild‐type (WT) plants, respectively. Under field conditions, the top‐performing event accumulated TAG to amount to 5.5% DW in leaves and 3.5% DW in stems, 78‐fold and 58‐fold increases, respectively, relative to WT TX430. Transcriptomic and fluxomic analyses revealed potential bottlenecks for increased TAG accumulation. Overall, our studies highlight the utility of a lab‐to‐field pipeline coupled with systems biology studies to deliver high vegetative oil sorghum for SAF and renewable diesel production.

## Introduction

Energy‐dense triacylglycerols (TAG), the principal component of vegetable oils, are the major starting material for the commercial production of sustainable aviation fuel (SAF) and renewable diesel. Currently, TAG‐rich feedstocks for renewable liquid fuels include oil palm and oilseeds such as soybean. Initiatives including the SAF Grand Challenge emphasize the need for additional TAG feedstocks (Holladay *et al*., 2020; U.S. Department of Energy *et al*., 2022). For example, the SAF Grand Challenge aims to produce 35 billion gallons of SAF annually by 2050, requiring more than 60% of vegetable oil from all current global sources (U.S. Department of Energy *et al*., 2022). Given this projected demand, “feedstock innovation” is one of the research priorities of the SAF Grand Challenge. Potential new sources of TAG to complement oil palm and oilseed feedstocks are harvested vegetative tissues (leaves and stems) of biomass grasses. In particular, C4 grasses, such as sorghum, sugarcane, and miscanthus have high photosynthetic efficiency and can generate high biomass yields (Mullet *et al*., 2014; Somerville *et al*., 2010). In sorghum, vegetative TAG accumulation at 5% of dry weight could yield up to 215 gallons per hectare, assuming biomass yields of 15 Mg/ha typical in the U.S. Midwest (Gautam *et al*., 2020). This is ~1.4 times the typical oil yield from soybean. However, this potential is currently constrained by the inability of conventional C4 grasses and other biomass crops to store TAG in vegetative tissues.

In contrast to oil‐rich seeds and mesocarp of plants such as oil palm and olive, accumulation of TAG in vegetative organs is typically limited to stress responses that result in membrane damage (Lin and Oliver, 2008; Lu *et al*., 2020). Attempts to redesign vegetative organs for non‐transient oil accumulation have largely focused on a ‘push, pull, protect’ (or “3P”) strategy (Park *et al*., 2021; Xu and Shanklin, 2016). In this strategy, transgenes, often those for the transcription factor WRINKLED1 (WRI1), are used to enhance the flux or ‘push’ of photosynthetic carbon into fatty acid biosynthesis in chloroplasts (Focks and Benning, 1998; Ma *et al*., 2013). The fatty acids are then “pulled” into storage in TAG by expression of transgenes for enzymes that assemble fatty acids onto glycerol backbones in the ER. Typically, this is achieved by overexpression of genes for diacylglycerol acyltransferase (DGAT), the final step in TAG biosynthesis (Turchetto‐Zolet *et al*., 2011; Zhang *et al*., 2009). Finally, to “protect” the synthesized TAG from turnover, transgenes for products that limit TAG catabolism are also expressed. These transgenes often include those for proteins, such as oleosin, that coat oil bodies to reduce access of lipolytic enzymes to sequestered TAG (Frandsen *et al*., 2001; Napier *et al*., 1996). RNAi or gene editing for suppression of native lipases in vegetative organs can also be used to protect TAG from catabolism (Kelly *et al*., 2013; Vanhercke *et al*., 2017). The most successful demonstration of the 3P strategy was the constitutive co‐expression of transgenes for the Arabidopsis WRI1, Arabidopsis DGAT1, and *Sesamum indicum* oleosin in *Nicotiana tabacum* (Vanhercke *et al*., 2014, 2017). The introduction of this transgene assembly yielded the stable accumulation of TAG in leaves to >15% DW.

As noted above, C4 grasses are a particularly appealing platform for engineering vegetative oil production. Several reports have described successful efforts to confer vegetative oil production in sorghum (*Sorghum bicolor*) as well as sugarcane (*Saccharum officinarum*) and its close relative energycane (Luo *et al*., 2022; Parajuli *et al*., 2020; Vanhercke *et al*., 2019; Zale *et al*., 2016). In the case of sorghum, biolistics were used to deliver transgenes for the maize Wri1, *Umbelopsis ramanniana* DGAT2a, and sesame oleosin‐L. In the resulting T_0_ plants, TAG concentrations in leaves as high as 8.4% DW were reported (Vanhercke *et al*., 2019). Whilst this study provided proof of principle, the top lines contained >25 copies of transgenes, and the stability of the trait in subsequent generations and under field conditions was not assessed. A similar 3P strategy combined with RNAi suppression of the gene for the sugar‐dependent lipase1 was used to engineer sugarcane (or ‘oilcane’). This approach yielded TAG concentrations of ~4.6% DW in stems and ~8% DW in leaves (Parajuli *et al*., 2020). High TAG production; however, was accompanied by significant reductions in biomass, a phenotype that was also observed in energycane engineered to produce similar TAG concentrations (Luo *et al*., 2022).

**Figure 1:**
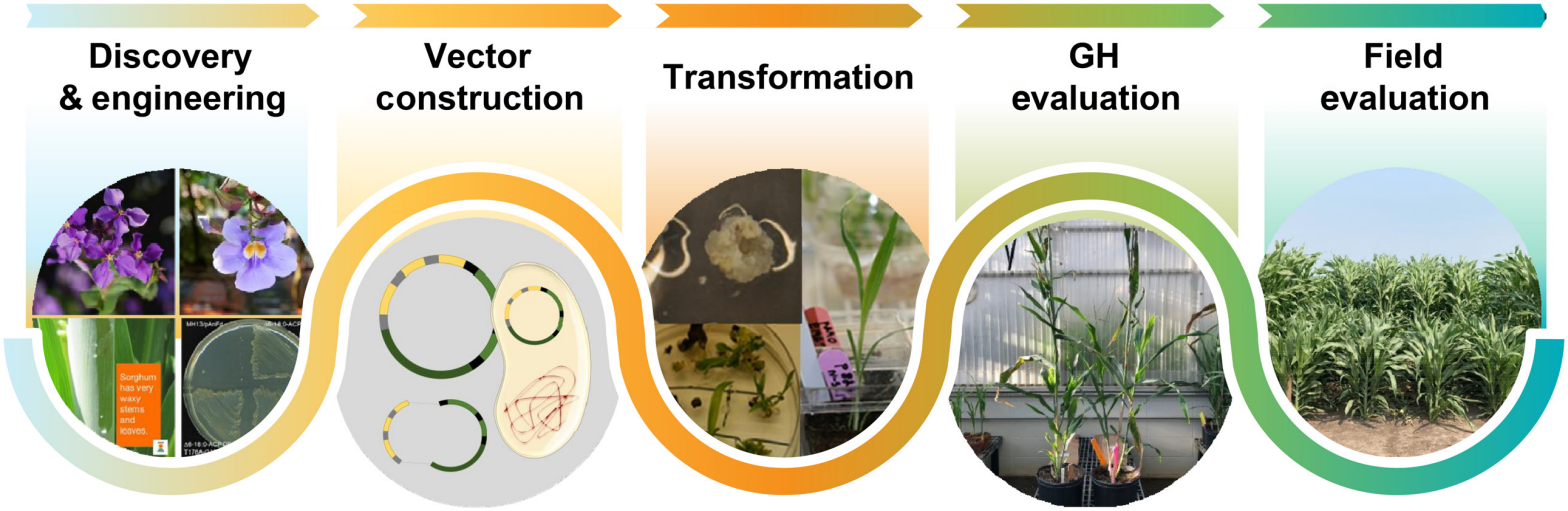
Overview of Lab‐to‐field pipeline.

Building on these studies, we explored the feasibility of developing sorghum as a viable C4 crop for field‐scale production of vegetative oil. We chose sorghum as our production platform because of its well‐developed functional genomics toolbox that includes a sequenced diploid genome and the availability of numerous genotypes for grain, sugar, forage, and biomass production (Bedell *et al*., 2005; Howe *et al*., 2006). In addition, sorghum has heat‐ and drought‐tolerant properties that make it well‐suited for dryland climates such as those in the US Great Plains. Our studies also took advantage of the ability of sorghum to be transformed by *Agrobacterium tumefaciens* to obtain less complexity of transgenes compared with biolistics, which was used for the transformation of sorghum, sugarcane, and energycane in the studies described above (Gao and Nielsen, 2013). This attribute enhances trait stability in subsequent generations and facilitates introgression of vegetative oil traits in other genotypes. In this report, we initially compared transgene combinations for a “standard” 3P strategy using Arabidopsis diacylglycerol acyltransferase1 (DGAT1) with those strategies to generate oils containing medium‐chain length fatty acids (MCFA). The latter transgene combinations contained constitutively expressed genes for variant *Cuphea* FatB acyl‐acyl carrier protein (ACP)‐thioesterases and MCFA specialized lysophosphatidic acid acyltransferases (LPATs) and DGAT1 (Iskandarov *et al*., 2017; Kim *et al*., 2015). Both strategies used constitutively expressed transgenes for sorghum WRI1 and sesame oleosin. Unexpectedly, the introduction of the MCFA transgenes did not generate MCFA oils but did yield the highest TAG concentrations of the two strategies and the oil contained typical C16 and C18 fatty acids. We show that the TAG phenotype in these lines is stable over multiple generations, including two seasons under field conditions. Oil concentrations of >5% of DW in leaves were obtained from field‐grown plants with no effects on biomass yields in grain and sweet sorghum backgrounds, in contrast to prior oilcane studies. We also applied systems biology analysis to gain insights into strategies for further enhancing vegetative oil concentrations. Overall, our lab‐to‐field pipeline strategy not only allows full agronomic and oil yield assessment of our lines, but also enables downstream functional evaluation of oil sorghum for applications such as SAF production (Figure 1).

## Results

### Evaluating strategies for vegetative oil sorghum production

**Figure 2:**
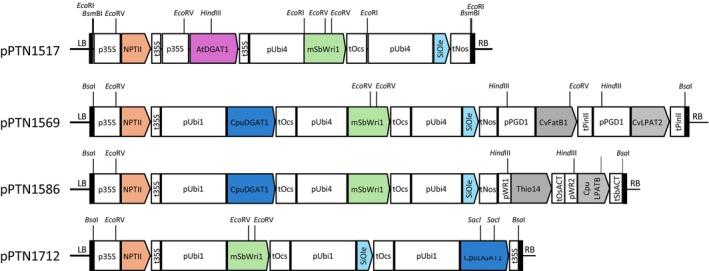
Schematic structures of T‐DNA for generation of transgenic sorghum plants. LB, left border; p35S, cauliflower mosaic virus 35S promoter; pPGD1, rice PGD1 promoter; pUbi1, maize ubiquitin1 promoter; pUbi4, sugarcane ubiquitin4 promoter; pWR1, WRINKLED1‐responsive promoter1; pWR2, WRINKLED1‐responsive promoter2; RB, right border; t35S, cauliflower mosaic virus 35S terminator; tNos, *Agrobacterium* nopaline synthase (Nos) terminator; tOcs, *Agrobacterium* Ocs terminator; tOsACT, rice actin1 terminator; tPinII, potato PinII terminator; tSbACT, sorghum actin1 terminator.

Our initial goal was to develop vegetative oil sorghum using a 3P strategy with WRI1, oleosin, and DGAT transgenes. Our second goal was to create vegetative oil sorghum rich in MCFA. For the first goal, we assembled transgenes for sorghum WRI1 (SbWRI1, “push”), Arabidopsis DGAT1 (AtDGAT1, “pull”), and sesame oleosin (SiOle, “protect”) in vector pPTN1517 (Figure 2) using GoldenBraid assembly. For MCFA oil production, we generated two binary vectors (pPTN1569 and pPTN1586, Figure 2) containing transgenes for SbWRI1, Cuphea DGAT1 (CpuDGAT1) instead of AtDGAT1, SiOle, LPAT from Cuphea (CvLPAT for pPTN1569 and CpuLPAT for pPTN1586), and specialized MCFA FatB thioesterases from Cuphea (CvFatB1 for pPTN1569 and Thio14 for pPTN1586). CpuDGAT1, CvLPAT, and CpuLPAT enhance MCFA TAG production (Iskandarov *et al*., 2017; Kim *et al*., 2015). We used Ubiquitin promoters from maize (Ubi1) and sugarcane (Ubi4), the PGD1 promoter from rice (Park *et al*., 2010), and synthetic promoters (pWR1 and pWR2) for ectopic expression. The design of the synthetic promoters pWR1 and pWR2 was based on the AW‐box, a WRINKLED1‐binding motif, as described previously (Maeo *et al*., 2009). These synthetic promoters consist of 10 tandem repeats of the AW‐box, oriented either in the forward (pWR1) or reverse (pWR2) direction, and fused to a minimal 35S promoter. These binary vectors were used to transform TX430 (grain sorghum) or Ramada (sweet sorghum) genotypes via *Agrobacterium*‐mediated transformation, generating T_0_ transgenic events.

**Figure 3:**
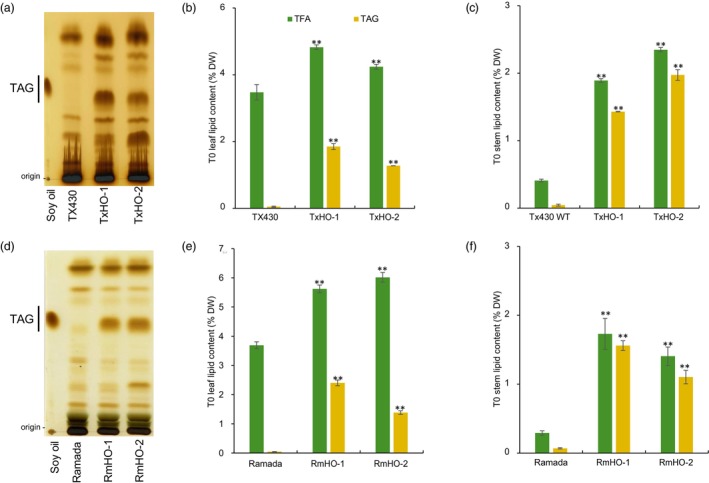
Oil content in leaves and stems from regenerated T_0_ pPTN1569‐expressing Tx430 background and pPTN1586‐expressing transgenic Ramada background events. (a) TLC analysis of TX430 transgenic events (lane 3 and 4). Total lipid extracts from fully matured leaves were developed under the mobile phase consisting of heptane:ethyl ether:acetic acid (70:30:1, v/v/v). Soybean oil in lane 1 was developed together as a TAG marker. (b) Lipid content in leaves and (c) stems of TX430 grain sorghum background transgenic events. (d) TLC analysis of Ramada transgenic events (lane 3 and 4). (e) Lipid content of leaf and (f) stem tissues from Ramada sweet sorghum background events. Error Bars mean ± SD (*n* = 3, technical replicates). **P < 0.01, Student's *t*‐test.

We used thin‐layer chromatography (TLC) of extracted lipids to screen fully expanded, mature leaves from T_0_ transformants. Leaf TAG content of 16 sorghum events carrying pPTN1517 was analysed to determine if the “typical” 3P strategy led to an increase in TAG content, and 31 and 19 events carrying pPTN1569 or pPTN1586, respectively, were analysed for MCFA production. TAG levels in T_0_ event leaves varied: 0.0%–0.8% DW for pPTN1517, 0.1%–1.8% DW for pPTN1569, and 0.0%–2.4% DW for pPTN1586 (Figure S1). Four events (two in TX430 backgrounds, TxHO‐1 and TxHO‐2 from pPTN1569; and two in Ramada backgrounds, RmHO‐1 and RmHO‐2 from pPTN1586) showed enhanced TAG production based on TLC analysis (Figure 3a,d). Gas chromatography (GC) analysis revealed that leaves of these four events accumulated TAG to the concentration of 1.3%–2.4% DW, whilst WT sorghum leaves contained <0.1% DW TAG (Figure 3b,e). However, these events, designed to produce medium‐chain fatty acyl TAG, instead produced TAGs enriched in C16 and C18 fatty acids with no detectable MCFA. After harvesting T_1_ seeds, stem tissues of T_0_ plants showed similar TAG patterns: four events accumulated TAG in stems about 1.1%–2.0% DW compared with >0.1% DW in stems of wild‐type (WT) plants (Figure 3c,f). Based on these results, we advanced the top lines from the MCFA vectors pTN1569 (TxHO‐1 and TxHO‐2) and pTN1586 (RmHO‐1 and RmHO‐2).

### Vegetative oil production is heritable

**Figure 4:**
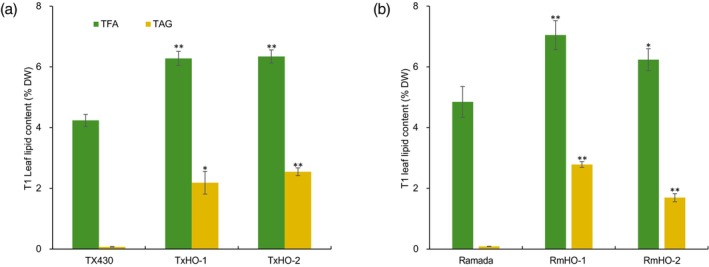
Greenhouse experiment of *Cuphea* DGAT1‐expressing transgenic T1 sorghum plants. TFA and TAG concentrations of leaf were measured from (a) Tx430 background plants and (b) Ramada background transgenics at boot‐stage. Error bar indicates mean ± SD (*n* = 3, biological replicates). Statistical significance was determined by comparing total fatty acid (TFA) and triacylglycerol (TAG) levels, respectively. *P < 0.05, **P < 0.01, Student's *t*‐test.

Oil content was measured in greenhouse‐grown T_1_ oil sorghum events to confirm stable inheritance of the oil trait. Leaves from TX430 events collected at boot‐stage were found to have TAG concentrations of 2.2% DW in TxHO‐1 and 2.5% DW in TxHO‐2 (Figure 4a). Leaves collected at the same stage from Ramada events had TAG concentrations of 2.8% DW in RmHO‐1 and 1.7% DW in RmHO‐2 (Figure 4b). By comparison, boot‐stage leaves from non‐transformed WT TX430 and Ramada plants had TAG concentrations of <0.1% DW (Figure 4). In addition, total fatty acid (TFA) concentrations in the transgenic events were increased by as much as 1.5‐times relative to concentrations in WT leaves. Increased TFA concentrations were also seen in leaves of T_0_ plants, and even larger in stalks of transgenic events (>4‐fold; Figure 3). These results suggest that increased TAG concentrations in transgenic lines arise, at least in part, from increased *de novo* fatty acid synthesis. Consistently, TX430 background T_1_ oil sorghum plants (TxHO‐2) grown in the greenhouse showed no significant growth differences compared with WT (Figure S2), in contrast to previous reports of vegetative TAG production using the 3P strategy in biomass crops (Parajuli *et al*., 2020; Vanhercke *et al*., 2019; Zale *et al*., 2016).

### Field evaluation of oil sorghum lipid and growth phenotypes

**Figure 5:**
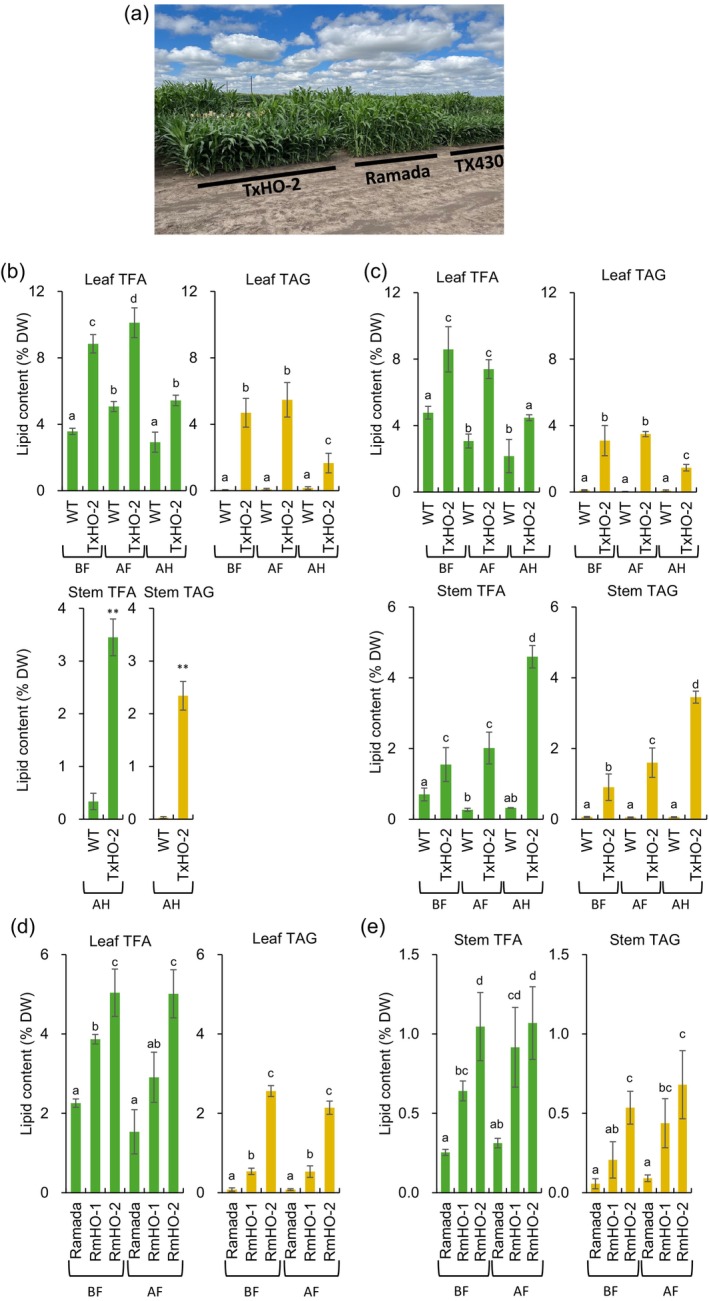
Phenotype analysis of a high oil sorghum event under the 2021 and 2022 Nebraska field summer conditions. (a) 2022 field view. The picture was taken on 4th September 2022, 70 DAS. (b) Lipid content of field‐grown TX430 background event from 2021 field trial. (c) Lipid levels in vegetative tissues of TX430 sorghum plants from 2022 field trial. Leaf and stem were collected at three different developmental stages and analysed using GC‐FID. (d) Lipid content of leaf tissues from 2022 field‐grown Ramada background events. (e) Lipid analysis of stem tissues obtained from 2022 field‐grown Ramada background events. T_3_ generation of Ramada transgenic events were planted in the field of eastern Nebraska. Leaf and stem tissues were collected at two timepoint, BF and AF. Ramada event could not survive after frost. Error bars represent ±SD (*n* ≥ 4, biological replicates). BF, before flowering (boot‐stage); AF, after flowering (after anthesis); AH, at harvest (soft‐dough stage). Letters indicate the result of one‐way ANOVA (α = 0.05). Different letters show significant differences at *P* < 0.05 determined by Tukey's multiple comparisons test.

Given the lack of an observed growth penalty in our greenhouse‐grown oil sorghum plants, we assessed the biomass production and oil concentrations in these events under field conditions over two seasons. The best‐performing TxHO‐2 event was grown in the field at the Eastern Nebraska Research, Extension, and Education Center (ENREEC) in 2021 (T_1_ generation) and 2022 (T_3_ generation) (Figure 5a). Leaves were collected at three different time points: before flowering (BF, boot‐stage), after flowering (AF, after anthesis), and at harvest (AH, soft‐dough stage) to gain information about growth and TAG titers in the vegetative stage, reproductive stage, and full maturity, respectively. In 2021, stem tissues were only collected and analysed at the AH stage, whilst in 2022, samples were collected at all three time points.

Results from lipid analyses of 2021 sorghum leaf samples showed that leaf TFA and TAG levels were maximal at the AF stage (10.3% DW for TFA, 5.5% DW for TAG; Figure 5b). TFA and TAG concentrations in leaves decreased at the AH stage (5.5% DW for TFA, 1.7% DW for TAG). A similar trend was observed for TFA concentrations of the WT TX430 control plant, which increased to 5.1% DW at the AF stage and then decreased to 3.0% DW. Lipid analysis of the 2021 stem samples at the AH stage showed that TFA and TAG accumulated to 3.5% DW and 2.3% DW, respectively. TAG concentrations in WT leaves at all stages were ≤0.3% DW.

In 2022, leaf TAG concentrations increased and were maximal at the BF stage and maintained until at the AF stage (3.1~3.5% DW; Figure 5c). TAG concentrations decreased to 1.5% DW at the AH stage, reflecting a similar pattern observed in the 2021 field trial. TFA concentrations were highest at the BF stage (8.6% DW) and stayed at the AF stage (7.4% DW). This was nearly a 2‐fold increase in TFA concentrations compared with those in WT leaves (Figure 5c). As of 2021, TAG concentrations in WT leaves were ≤0.3% DW. Stem TFA and TAG concentrations in 2022 were maximal at the AH stage (4.6% DW for TFA and 3.5% DW for TAG). We also observed that the fatty acid profile of TAG in TxHO‐2 changed in later developmental stages (Figure S3). In WT leaves, although trace amounts of TAG were detected, linolenic acid was the major fatty acid at both BF and AH time points, whilst palmitic acid became predominant at AF. In the engineered oil sorghum leaves, however, there was a significant increase in oleic acid at all three timepoints, with its percentage increasing in later stages. The same but more significant trend was observed in oil sorghum stalk samples, whilst linoleic acid was the major fatty acid species found in WT stalk samples at all three stages. In TAG from the oil of sorghum plants, oleic acid accounted for >40% of the TFAs.

For oil sorghum events in the Ramada background, we were only able to test them in the field in 2022, due to the insufficient seed quantity in 2021. Even though the same sowing date with TX430 background, Ramada plants did reach the soft‐dough stage and encountered frost because of the longer life cycle. Lipid analysis results of Ramada oil sorghum events from two developmental stages, BF and AF showed that oil accumulation pattern is very similar to TX430 oil sorghum event, which may suggest that sugar content difference between two genotypes does not confer oil accumulation in vegetative tissues (Figure 5d). In RmHO‐1, the best‐performing Ramada event, both TFA and TAG concentrations of leaf and stem tissues were maximal at BF stage (5.4% DW and 2.6% DW for leaves and 1.1% DW and 0.7% DW for stems, respectively). These lipid contents were all significantly higher than the WT Ramada control: 2.3‐fold more than WT leaf TFA, 52‐fold more than WT leaf TAG, 3.7‐fold more than WT stem TFA, and 7.1‐fold more than WT stem TAG.

Leaf‐level gas exchange measurements were conducted during the 2022 season to assess potential differences in photosynthetic capacity between WT and oil‐accumulating sorghum events (Figure S4). The analysis revealed no significant differences between the two groups in any of the three photosynthetic parameters: net CO₂ assimilation rate, stomatal conductance, and transpiration rate.

We also analysed galactolipids content in the oil sorghum events. Both monogalactosyldiacylglycerol (MGDG) and digalactosyldiacylglycerol (DGDG) concentrations were not statistically different between leaves of transgenic plants compared with those of WT, whilst a marked increase in neutral lipid content was observed (Figure S5a). In addition, relative amounts of oleic and linoleic acids in both MGDG and DGDG fractions were significantly higher in the leaves of transgenic oil sorghum events compared with the wild‐type (Figure S5b,c). This enrichment in oleic and linoleic acids was also observed in the neutral lipid fraction (Figure S5c), consistent with the TAG composition in greenhouse‐grown plants (Figure S3). We further measured concentrations of non‐structural carbohydrates (NSC), including sucrose, D‐glucose, and starch in stems of engineered and non‐engineered plants (Figure S6). In the TX430 genotype background, stems of the high oil lines had higher NSC concentrations compared with WT stems. The Ramada sweet sorghum genotype overall contained more starch and sucrose than the TX430 grain sorghum genotype. However, no differences in NSC concentrations were observed between WT Ramada and the transgenic events RmHO‐1 and RmHO‐2.

**Figure 6:**
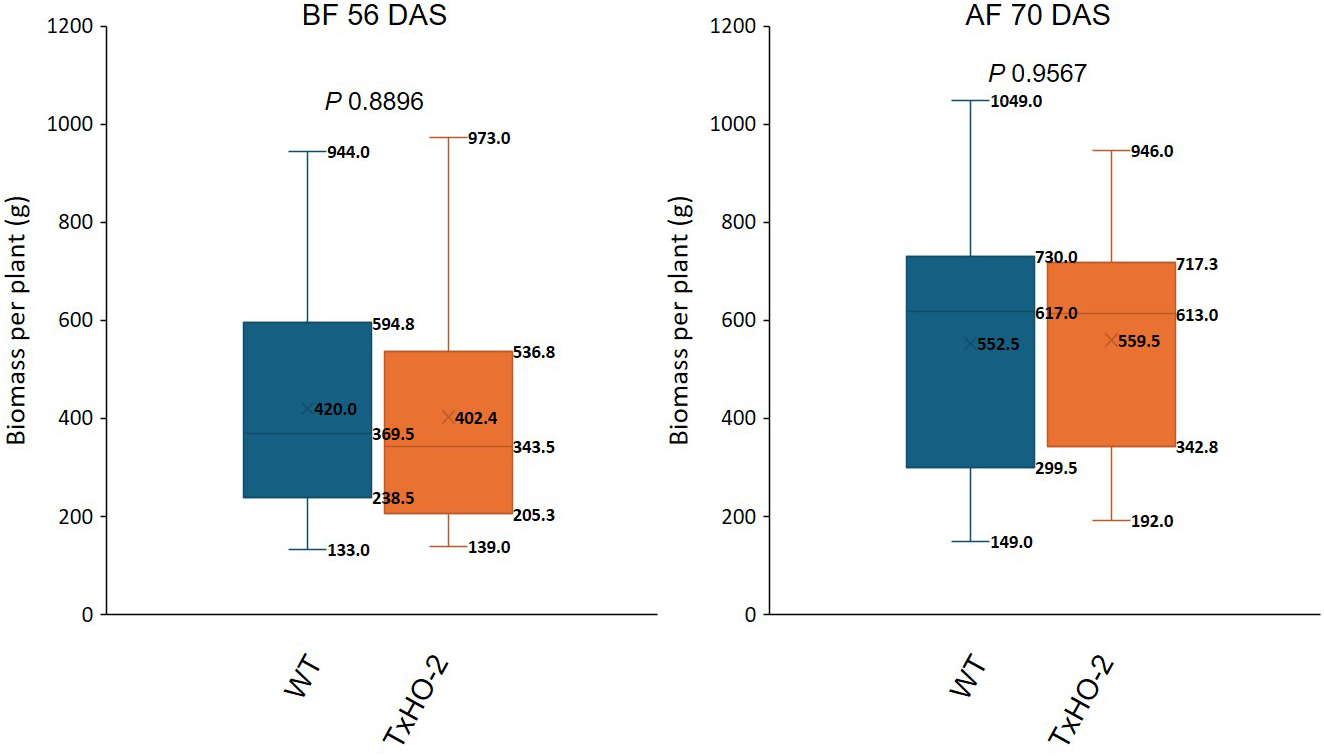
Fresh biomass comparison between WT and a high oil sorghum event in 2022 field trial. Arial vegetative parts, including leaves and stems, of plants were cut and weighed for the fresh weight. AF, after flowering (after anthesis); BF, before flowering (boot‐stage); DAS, day after sowing. *n* = 12, biological replicates. *P*‐values from Student's *t*‐test are shown.

Overall, the 2‐year field trial results indicate that leaf and stem tissues have different time points for maximizing lipid content: the early reproductive stage for the leaf and the late reproductive stage for the stem. To confirm the normal growth of the TxHO‐2 oil sorghum event, we measured the fresh weight of the aerial part of oil sorghum plants at two different time points, BF and AF stages, in 2022. Again, results showed no significant differences in fresh weight between TxHO‐2 and WT plants under field growth conditions (Figure 6).

### Correlation of oil accumulation and transgene expression

To examine the correlation between transgene expression and oil accumulation phenotype, digital droplet PCR (ddPCR) was conducted using leaves at the V5 vegetative stage of greenhouse‐grown pPTN1569‐harbouring events with differing TAG concentrations. Four transgenic events were selected for this experiment with TAG concentrations representing low, intermediate, and high oil accumulation. TFA and TAG concentrations were measured for leaves at the V5 stage and after anthesis (after flowering, AF), and for stem tissues at full maturity (after seed harvest, AH). Results showed that three out of five transgenes, *CpuDGAT1*, *SiOle*, and *SbWRI1*, were highly expressed under the control of ubiquitin promoters (Table S1). However, the expression levels of *CvFATB1* and *CvLPAT2* transgenes were much lower, possibly due to the inefficiency of rice PGD1 promoters in sorghum. Based on transgene expression levels and TFA and TAG concentrations of these four independent transgenic sorghum events (Table S1), correlation analysis indicated that expression of *CpuDGAT1* and *SiOle* was most correlated with TFA and TAG accumulation (Table 1). Whilst the expression level of *SbWRI1* was high in the transgenic events overall, it was less correlated with oil accumulation in the transgenic sorghum plants.

**Table 1:** Correlation between transgene expression and lipid content in transgenic sorghum harbouring pPTN1569

Correlation coefficient of transgene expression/lipid content of leaf and stem					
	SbWRI1	SiOle	CpuDGAT1	CvFATB1	CvLPAT2
Leaf V5 – TFA	0.02	0.52	0.79	0.03	−0.51
Leaf V5 – TAG	0.34	0.80	0.91	0.09	−0.50
Leaf AF – TFA	0.23	0.69	0.79	−0.12	−0.59
Leaf AF – TAG	0.06	0.54	0.70	−0.15	−0.53
Stem – TFA	0.25	0.60	0.69	0.11	−0.53
Stem – TAG	0.30	0.63	0.68	0.05	−0.55

For expression analysis, RNA was extracted from the middle section of the first fully developed leaf blade at V5 stage. Transgene expression levels were determined using ddPCR with gene‐specific primers. Data was normalized using EIFA4 reference gene (*n* = 4 biological replicates, mean ± SD). For lipid analyses, leaf and stem tissues were collected from the same plants used in transgene expression analysis at three stages (V5 and after flowering stages for leaf and full maturity for stem tissues). TFA and TAG contents were determined by GC‐FID (*n* = 4, mean ± SD). Correlation coefficient was calculated using Microsoft Excel function CORREL based on expression and lipid content data (Table S1).

### RNAseq analysis of oil sorghum

Despite using strong ectopic promoters, TAG levels in vegetative tissues never reached seed oil levels (~30 wt%). To identify the bottleneck in TAG accumulation, we performed RNA sequencing (RNAseq) on fully developed leaves from the 2021 field trial. To examine the bottleneck of TAG accumulation in oil sorghum, RNA sequencing (RNAseq) was carried out using RNA from fully developed leaves from the 2021 field trial. We identified differentially expressed genes between the WT and transgenic TxHO‐2 event and applied Gene Ontology (GO) term enrichment analysis to sets of genes that were up‐ or down‐regulated in the transgenic lines. Amongst these, 342 genes were up‐regulated, with GO terms like ‘glycolytic process’, ‘glutathione metabolic process’, and ‘fatty acid biosynthetic process’ being overrepresented (Tables S2 and S3). Conversely, 212 genes were down‐regulated and the GO terms ‘photosynthesis, light harvesting in photosystem I’, ‘protein‐chromophore linkage’, and ‘response to light stimulus’ were overrepresented.

To further analyse the function of differentially expressed genes, we identified candidate sorghum homologues to 332 *Arabidopsis thaliana* genes with established functions in primary metabolism, including glycolysis, plastidic fatty acid, and TAG biosynthesis, as well as lipid degradation and beta‐oxidation. Homology relations based on protein sequence similarity were catalogued along with predictions on subcellular localization in Table S4. Of 393 sorghum homologues, 45 were significantly up‐regulated and five down‐regulated in the transgenic line (*P*adj < 0.05, Table S2), which is consistent with the GO term enrichment analysis of up‐regulated genes (Table S3).

Since the transcription factor, WRI1 was overexpressed in the transgenic lines, and WRI1 is known as a master regulator of oil biosynthesis in developing seeds (Focks and Benning, 1998; Marchive *et al*., 2014), it was expected that many sorghum genes involved in *de novo* fatty acid biosynthesis and glycolysis would be up‐regulated in the transgenic line. Indeed, amongst the 393 homologues, 51 are candidate homologues to expected WRI1 targets, 13 of which are significantly up‐regulated in the transgenic event (Table S4) (Kuczynski *et al*., 2022). Whilst many of the well‐characterized *A. thaliana* genes can be uniquely associated with a homologue in *S. bicolor* (Table S4), the homology relationship is not as clearly defined for acetyl‐CoA carboxylase (ACCase), the enzyme responsible for the first specific step in fatty acid biosynthesis. Plants harbour two different types of ACCase: a heteromeric form composed of four separate protein subunits, and a homomeric form where all four subunit domains are combined in a large polypeptide (Gornicki *et al*., 1997; Sasaki and Nagano, 2004). In *A. thaliana* and probably most land plants, the heteromeric form is predominantly active in *de novo* fatty acid biosynthesis in the chloroplast, but in the Poaceae, this role is assumed by the homomeric form (Sasaki and Nagano, 2004). Therefore, we aimed to determine which ACCase isoform is controlled by WRI1 in sorghum. No gene homologues for the *A. thaliana* heteromeric ACCase subunits was found in sorghum (Table S4). Instead, we found that Sobic.006G030100, one of three homologues to the plastidic homomeric ACCase in Arabidopsis (AT1G36180), was significantly up‐regulated ~10‐fold in the transgenic line (*P*adj < 0.001, Table S2).

If this ACCase gene is controlled by WRI1, its promoter should contain the AW‐box transcription factor binding motif, which has been identified in the non‐coding upstream regions of WRI1 gene targets in Arabidopsis (Maeo *et al*., 2009). We recently found that in *A. thaliana*, such WRI1 binding sites tend to be highly conserved across closely related Brassicaceae species (Kuczynski *et al*., 2022). Therefore, we searched for homologues to Sobic.006G030100 in the genomes of five other Poaceae species and found two AW‐box motifs in the non‐coding upstream regions of these homologues to be conserved (Figure S7a). Using MicroScale Thermophoresis (MST), we measured the binding affinity of the *A. thaliana* WRI1 protein to short DNA fragments encoding the two sorghum sites as described in Kuczynski *et al*. (2022). The equilibrium dissociation constants values measured were in the nM range (Figure S7b), indicating strong in vitro binding of WRI1 to these sites.

### Potential gene expression inhibitions

Triacylglycerols accumulation in engineered sorghum balances biosynthesis and turnover. Reducing lipase activity could lower vegetative TAG catabolism, given lipases' role in TAG breakdown during germination and senescence (Troncoso‐Ponce *et al*., 2013). Down‐regulating lipases to prevent breakdown has increased seed oil content (Aznar‐Moreno *et al*., 2022). We searched the oil sorghum RNAseq data for lipase genes with increased expression relative to WT control. In *A. thaliana*, 31 genes are designated as TAG or monoacylglycerol (MAG) lipases (Li‐Beisson *et al*., 2013). We identified 38 candidate *S. bicolor* homologues (Table S4), with Sobic.003G348700 up‐regulated over 60‐fold in the transgenic line (*P*adj < 0.001, Table S2). This gene, a candidate homologue to 8 *A. thaliana* MAG lipases (Table S4), is a promising target for future engineering.

Additional target genes for TAG catabolism are associated with β‐oxidation for recycling fatty acid chains through the glyoxylate cycle (Canvin and Beevers, 1961; Eccleston and Ohlrogge, 1998). From 22 *A. thaliana* genes, we identified 25 sorghum candidate homologues, with two (Sobic.009G111400 and Sobic.010G001900) overexpressed at least 3‐fold in the transgenics (*P*adj < 0.03, Table S4).

Despite the expression of our specialized FatB thioesterases (*CvFatB1*, *Thio14*), it could be that the accumulation of MCFA in TAG is prevented by re‐acylation of FatB‐released MCFA to ACP to be subjected to further elongation. Acyl‐activating enzymes (AAE) are involved in activating acyl chains for fatty acid elongation (Shockey *et al*., 2003). In *A. thaliana*, AAE15 (AT4G14070) and AAE16 (AT3G23790) have specificity for MCFA, and their expression is expected to prevent MCFA from accumulating in storage lipids (Kaczmarzyk *et al*., 2016; Tjellström *et al*., 2013). We identified a candidate homologue to *A. thaliana* AAE15/16 in sorghum (Sobic.001G014900, Table S4). This gene was expressed at similar levels in WT and high oil sorghum (*P*adj > 0.9, Table S2). This means that Sobic.001G014900 might be responsible for activating part of the MCFA released by the specialized FatB thioesterases, which might explain the lack of MCFA accumulation.

### Differences between MCFA and normal TAG constructs

Despite the low expression levels of specialized FatB thioesterase transgenes in oil sorghum lines and the lack of detectable MCFA in TAG from these lines, we examined whether the specialized FatB thioesterases might contribute to oil accumulation. As one test, we generated a new binary vector construct (pPTN1712, Figure S1) containing transgenes for SbWRI1, CpuDGAT1, and SiOle but lacking specialized FatB thioesterase and LPAT transgenes. The 66 T_0_ transgenic plants obtained from the transformation of this construct contained on average more oil (leaf TAG concentrations ranged 0% DW to 1.1% DW) than our typical 3P lines from pPTN1517 (AtDGAT1, SbWRI1, SiOle transgenes, highest event accumulating TAG up to 0.8% DW in leaves). These lines, however, produced less oil than lines from the MCFA vector constructs, pPTN1569 and pPTN1586 (the highest events store amount of TAG up to 1.8% DW and 2.4% DW in leaves, respectively).

Secondly, we were able to detect four‐fold higher myristoyl (14:0)‐ACP thioesterase activity relative to WT in leaves from pTN1569 that contains the transgene for the CvFatB1 14:0‐ACP thioesterase (Figure S8). Based on these results, we cannot exclude a role of specialized FatB thioesterases and/or LPATs in contributing to the high oil phenotype of lines from the MCFA vectors.

### Using labelling to compare fatty acid synthesis

**Figure 7:**
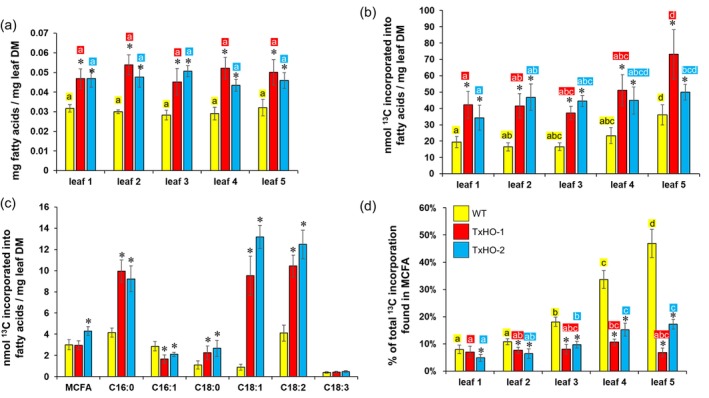
Fatty acid content and labelling in *S. bicolor* leaves. Leaf disc punches from WT (yellow) and high‐oil transgenic (red, blue) *S. bicolor* leaves of 5 developmental stages were labelled with [2‐^13^C]acetate for 2 days before analysis of total fatty acids. Leaf 1 denotes the youngest leaf stage. (a) Total fatty acid content, (b–d) incorporation of ^13^C into leaves. Bars show averages and error bars indicate SD (*n* = 5, biological replicates). Asterisks indicate if there was a significant difference between the WT and each of the transgenic lines (two‐sided *t*‐test, *P* < 0.05). Letters show the result of one‐way ANOVA (α = 0.05) for each genotype in separate. Different letters represent significant differences at *P* < 0.05 determined by Tukey's multiple comparisons test. MCFA, medium‐chain fatty acids (C12:0 and C14:0 were abundantly present).

To investigate why oil sorghum lines did not accumulate more MCFA and to estimate lipid biosynthesis rates, we used isotope tracer experiments. Leaf samples were taken from WT and transgenic plants (TxHO‐1 and TxHO‐2) before flowering. Five leaf developmental stages were sampled, ranging from still expanding at the base (stage 1) to starting to senesce (stage 5). After incubation of leaf discs with 1 mM [2‐^13^C]acetate for 2 days under continuous light (Figure S9a), total lipids were extracted and analysed by GC/MS for fatty content, composition, and label enrichment (Figures 7 and S9). Similar to greenhouse and field results, transgenic oil sorghum leaves of all stages examined contained about 50% more TFAs per dry weight than WT (Figure 7a). Across the leaf stages and within a genotype, there was little change in TFA content (Figure 7a). The transgenic lipids contained ~10% more oleic acid (C18:1) and linoleic acid (C18:2) than WT, whilst linolenic acid (C18:3) comprised over 15% more in the WT (Figure S9b). This trend is consistent across leaf stages (Figure S9c). Similar to the absence of MCFA chains in TAG of transgenic lines, we found that MCFA are minor components of total leaf fatty acids at all stages and did not increase in the transgenics (Figure S9b). With increasing leaf age, the fraction of MCFAs in transgenics actually decreased relative to the WT (Figure S9c).

After feeding ^13^C‐labelled acetate for 2 days, the amount of ^13^C incorporated in fatty acids was comparable between WT and transgenics. Significantly more ^13^C was incorporated into the TFAs of transgenic leaves than in WT (Figure 7b). This trend persisted across the leaf developmental stages with a mild increase in ^13^C ‐incorporation towards stage 5 (Figure 7b). Since the TFA levels did not differ significantly between the leaf stages (Figure 7a), it can be assumed that *de novo* fatty acids biosynthesis does not lead to lipid accumulation over time. Additionally, although stage 1 leaves were still growing, none of the sampled leaf sections likely accumulate lipids by cell growth, as grass leaves grow from the base where they are enclosed by older leaf sheaths (Fricke, 2002). Thus, the ^13^C incorporation rates in Figure 7b can be viewed as metabolic turnover rather than new synthesis. It can be deduced from Figure 7a,b that higher contents of TFAs in the transgenics (Figure 7a) are associated with higher lipid turnover rates (Figure 7b). This suggests that higher lipid levels in the transgenics are prevented by higher lipid degradation.

Figure 7c shows ^13^C incorporation rates into individual fatty acid species at leaf stage 3, revealing substantially increased rates of palmitic acid (C16:0), oleic acid (C18:1), and linoleic acid (C18:2) in the transgenics, whilst new synthesis of linolenic acid (C18:3) remains low (Figure 7c). Similar trends across all leaf stages (Figure S9d) might indicate limited desaturation of C18:2 to C18:3 in the transgenics. However, since C18:1 and C18:2 are precursors of C18:3, there will be a delay until newly incorporated ^13^C‐acetate reaches C18:3. If the 2‐day labelling experiment was within this initial phase, C18:3 new synthesis may not be detectable. Additional labelling experiments using ^13^CO_2_ are underway to better address the turnover rates of individual fatty acids. Regarding MCFAs, Figure 7d shows that in WT, the proportion of ^13^C incorporation found in MCFAs increases with leaf age to about 50%. This is not the case for the transgenic lines: relative to the WT, the proportion of ^13^C incorporation found in MCFAs is lower in the transgenic lines (Figure 7d). The results in Figure 7d are difficult to reconcile with the idea that transgenic lines produce more MCFAs. Note that increased C14:0‐ACP thioesterase activity was demonstrated for line TxHO‐2 (Figure S8). One interpretation of these results is that although there is increased release of MCFA from the fatty acid elongation cycle in the transgenics, most of these released chains are immediately degraded by β‐oxidation, which may be increased in the transgenics.

## Discussion

This study highlighted our lab‐to‐field pipeline for the development, trait assessment, and systems level interrogation of vegetative oil sorghum. We demonstrated that 2 five‐transgene constructs intended to produce MCFA vegetative oils resulted, instead, in accumulation of TAG in leaves and stems enriched in oleic acid, without increased MCFA content. Moreover, TAG concentrations in our lines were considerably higher than those achieved with a three‐transgene construct using a conventional push‐pull‐protect strategy (Figure S1). This high oil trait was observed in grain and sweet sorghum genotypes. Our top line TxHO‐2 produced TAG up to 3.5% DW and TFA up to 4.6% DW in stem tissues in 2022 under Nebraska field conditions. In addition, the maximal concentrations of TFA in leaves in these lines reached ~8% DW, ~twofold higher than those in leaves of WT plants at comparable stages. Notably, no obvious growth penalty was observed in our oil sorghum events under greenhouse or field conditions, contrasting with previous reports of TAG‐accumulating C4 biomass crops (Figures 6 and S2) (Parajuli *et al*., 2020; Vanhercke *et al*., 2019; Zale *et al*., 2016). Consistent with this, we found no significant differences in CO_2_ assimilation, stomatal conductance, transpiration rates, or major thylakoid lipid (MGDG and DGDG) concentrations between WT and high oil sorghum plants under field conditions (Figures S4 and S5). Despite the use of constitutive promoters, we also detected no effects on seed oil content or germination. Whilst we detected increases in root TAG concentrations to amounts of ~1% DW in TxHO‐1, no effects on root growth were observed. Overall, these findings show the utility of our lab‐to‐field approach to obtain better estimates of oil concentrations compared with greenhouse production and to fullyassess potential yield drag associated with vegetative oil traits.

The lack of a measurable reduction in biomass in oil‐accumulating sorghum is one of the notable observations of our studies. We cannot exclude the possibility that the TAG and TFA concentrations obtained in our studies are not sufficiently high to exceed the photosynthetic capacity of the engineered sorghum. Alternatively, the sequestration of photosynthetic carbon in fatty acids may alleviate negative regulation of carbon capture to provide enough photosynthate to support growth whilst also providing additional flux for enhanced fatty acid biosynthesis. This phenomenon was previously described for oil‐accumulating perennial ryegrass that exhibited biomass increases relatively to WT plants (Beechey‐Gradwell *et al*., 2020) and may explain increases we detected in non‐structural carbohydrates in oil‐accumulating lines in the TX430 background (Figure S6). It has also been previously suggested that an imbalance between WRI1 transcriptional induction and DGAT activity can result in excessive production of toxic‐free fatty acids, resulting in growth inhibition (Kannan *et al*., 2022; Parajuli *et al*., 2020; Yang *et al*., 2015). Of note, we detected considerably higher expression of *CpuDGAT1* than SbWRI1 (5723 vs 509, mean expression, Table S1) in TxHO‐2, the top‐oil accumulating event. This finding may suggest that in our lines, we obtained an optimal balance of DGAT activity to restore the enhanced flux of fatty acid biosynthesis conferred by WRI1, thus minimizing any detrimental effect on growth from free fatty acids.

It is also unclear if the FatB thioesterases and LPATs specialized for MCFA biosynthesis and metabolism were important for generating the high oil phenotype of the engineered sorghum. Although there was relatively low expression of the corresponding transgenes (Table S1), less oil was accumulated in engineered sorghum if the specialized FatB and LPAT transgenes were omitted suggesting that these enzymes may contribute to the high oil phenotype. It is possible, for example, that MCFAs generated by specialized FatB activity are preferentially used for β‐oxidation and effectively mask long‐chain fatty acids from turnover, allowing for these to accumulate in TAG. Regardless, more research is needed to confirm the utility of MCFA transgenes to obtain elevated vegetative oil accumulation.

In addition to assessing the levels of TAG and TFA in leaves and stems of transgenic lines, we assessed lipid biosynthesis by use of ^13^C‐labelled acetate in *in vitro* labelling experiments with leaf discs isolated from different developmental stages. Whilst total lipid levels remained largely unchanged in different stages, we consistently found higher ^13^C incorporation into lipid‐bound fatty acids in the transgenics compared with the WT control (Figure 7b,c). Our results indicate that lipid turnover continuously takes place and is higher in the transgenics. Thus, it is likely that lipid accumulation is limited by catabolic processes. Similar results were previously obtained in *Nicotiana tabacum* engineered for vegetative oil accumulation by overexpressing *A. thaliana* DGAT1 and oleosin from *Sesamum indicum*. Pulse‐chase labelling of leaf discs with ^14^C‐acetate indicated that *de novo* lipid biosynthesis as well as lipid turnover were increased in transgenics compared with the WT control (Vanhercke *et al*., 2017). Upon additional overexpression of the LEC2 transcription factor or silencing of SDP1 lipase in the transgenic background, leaf disks in ^14^C‐acetate labelling experiments showed reduced lipid turnover (Vanhercke *et al*., 2017). Further studies on these transgenic tobacco lines revealed that lipid turnover and associated changes in carbon metabolism may represent major hurdles for vegetable oil engineering approaches (Johnson *et al*., 2024; Mitchell *et al*., 2020; Zhou *et al*., 2020). A current goal of our research is to obtain additional oil accumulation in engineered sorghum. The evaluation of our lines in the greenhouse and field coupled with their systems biology interrogation has provided a Design‐Build‐Test‐Learn (DBTL) cycle for our first iteration of oil sorghum. The systems biology‐enabled “learning” has provided clues for improved transgene “design” for increased oil production. For example, our analysis of transgene expression indicated that the oil phenotype is most correlated with expression levels of DGAT and oleosin transgenes. Based on this, obtaining higher expression levels of these transgenes, plus identifying more catalytically active DGATs, may be a viable strategy for obtaining increased oil concentrations. RNAseq studies also identified a MAG lipase gene (Sobic.003G348700) is significantly up‐regulated >60‐fold in the transgenic sorghum event (*P*adj < 0.001, Table S2). Given the central role of lipases in oil and fatty acid catabolism, down‐regulation of this gene may provide an additional strategy for enhancing TAG accumulation in engineered sorghum (Aznar‐Moreno *et al*., 2022). Furthermore, our RNAseq studies also identified strong *WRI1*‐conferred upregulation of Sobic.006G030100 that encodes a plastid homomeric ACCase (Gornicki *et al*., 1997). This enzyme, which generates malonyl‐CoA to support fatty acid biosynthesis, is generally regarded as a major rate‐limiting enzyme for fatty acid production (Ohlrogge and Browse, 1995). In contrast to non‐Poaceae species, where plastidic ACCase is a heteromeric enzyme with three distinct subunits, the Poaceae lack the heteromeric enzyme and a homomeric ACCase is responsible for plastidic malonyl‐CoA production (Sasaki and Nagano, 2004). To our knowledge, this is the first report of WRI1 regulation of the expression of genes for plastidic homomeric ACCase, and we also functionally confirmed the presence of WRI1 binding sites in the promoters of genes for Poaceae homomeric ACCase. As such, targeting the overexpression of homomeric ACCase in sorghum and other Poaceae may generate additional vegetative oil accumulation.

Overall, our lab‐to‐field work has set the stage for translational research for the commercial use of high vegetative oil sorghum as renewable fuel feedstock and for energy‐dense forage. We identified optimal stages for TAG accumulation in leaves and stems of engineered plants. This information may guide the development of strategies to mitigate potential regulatory concerns with unintended dispersal of engineered seeds. In addition, our germplasm may be useful for introgression into other sorghum genotypes (e.g., ‘energy sorghum’) or into regionally adapted germplasm as part of breeding programs. Beyond biological studies, our current high oil sorghum can be used to develop and optimize effective harvesting strategies and equipment as well as extraction methods that allow for maximal oil recovery. These collective efforts will pave the way for vegetative oil feedstocks as solutions for addressing the highly anticipated demand for vegetable oils for food applications and biofuel needs, particularly for the SAF Grand Challenge goals.

## Experimental procedures

### Plant materials and growth conditions

Sorghum (*Sorghum bicolor* L. Moench. TX430 and Ramada genotypes) seeds were sown into peat pots (Jiffy Group) with soil mix composed of Metromix 300 (Sun Gro Horticulture) and grown under the greenhouse condition utilizing a 12/12 h photoperiod with day/night temperatures of 27~29 °C/19~21 °C. For field trials, transgenic sorghum event and WT plants were grown in the field at Eastern Nebraska Research, Extension and Education Center (ENREEC) in Mead, NE (41°08′42.7″ N 96°26′20.5″ W) in the 2021 and 2022 seasons. To measure lipid content of field‐grown vegetative tissues, leaf and stalk samples were harvested from the fifth node count from the bottom. Tissues were collected at three different stages; boot‐stage (before flowering, BF), after anthesis (after flowering, AF), and soft‐dough (at harvest, AH) stages. Samples were immediately frozen by liquid nitrogen and then lyophilized using a freeze‐dryer (FreeZone 2.5; Labconco) for 2 days. After the lyophilization, samples were immediately analysed or stored in a −80 °C freezer until analysis.

### Vector construction and sorghum transformation

Binary vectors for sorghum plants were constructed by GoldenBraid modular assembly (Sarrion‐Perdigones *et al*., 2013). Genes used in vector construction were GoldenBraid‐domesticated and codon‐optimized for sorghum by gene synthesis (GenScript Biotech). GenBank accession number of genes used in this study were Arabidopsis DGAT1 (AtDGAT1, NP_179535.1), Cuphea DGAT1 (CpuDGAT1, ANN46862.1), sorghum WRINKLED1 (SbWRI1, XP_002450194.1), sesame oleosin (SiOle, Q9XHP2.1), Cuphea FatB1 (CvFatB1, G3ESU9.1; Thio14, AAC49180.1), and Cuphea LPAT (CvLPAT2, ALM22867.1; CpuLPATB, ALM22873.1). For sorghum WRINKLED1, one amino acid residue was mutated (K10R) to increase protein stability (Zhai *et al*., 2017). Each gene was driven by constitutive promoters such as upstream regions of maize ubiquitin1, sugarcane ubiquitin4, and rice PGD1 (Christensen and Quail, 1996; Park *et al*., 2010; Wei *et al*., 1999). *Agrobacterium tumefaciens* cells (strain NTL4/Chry5) containing sorghum oil binary vectors were obtained by electroporation. Sorghum plants were transformed using immature embryos as described previously (Howe *et al*., 2006). In this study, four top‐performing transgenic events were analysed. Transgenic lines harbouring pPTN1569 (Figure 2), TZ424‐4‐5a and TZ424‐5‐3a in the TX430 genotype background were renamed as “TxHO‐1” and “TxHO‐2”, respectively. Similarly, transgenic events harbouring pPTN1586 (Figure 2), MW144‐2‐4 and MW144‐4‐3 in the Ramada genotype background were renamed as “RmHO‐1” and “RmHO‐2”.

### Extraction of total lipids and analysis

Total lipid was extracted from vegetative tissues using a modification of the Bligh–Dyer method (Bligh and Dyer, 1959). A 30 mg aliquot of freeze‐dried sorghum leaves was ground in 3 mL of methanol:chloroform (2:1 v/v) with 50 μg of triheptadecanoin (Nu‐Chek Prep) as an internal standard. Homogenized samples were shake‐incubated for 30 min at room temperature, and lipids were partitioned and extracted by adding 1 mL of chloroform and 1.9 mL of distilled water. The extracted organic phase was fully evaporated using nitrogen gas and resuspended in 100 μL of chloroform. To separate TAG, TLC was developed using the 50 μL of lipid extract using mobile phase (heptane:diethyl ether:acetic acid, 70:30:1, v/v/v). TAG was visualized and scraped by spraying 0.01% primuline (dissolved in 80% acetone, w/v; Sigma‐Aldrich) on a TLC plate. Fatty acid methyl esters (FAMEs) were generated by adding 1 mL of 2.5% sulfuric acid (v/v) in methanol on both total lipids and TAG samples and heating for 30 min at 90 °C. Partitioned FAMEs were analysed by an Agilent 7890A GC with a 30 m × 0.25 mm HP‐INNOWax column (Agilent). The gas chromatograph was programmed for an initial temperature of 90 °C (1‐min hold) followed by 30 °C/min to 235 °C and maintained for 5 min. Detection was achieved using flame ionization (FID). For sorghum stalk lipid analysis, a 60 mg aliquot of freeze‐dried samples was used.

### RNA isolation for droplet digital PCR and transcriptome analysis

Total RNA was isolated from fully developed leaves of WT and transgenic sorghum plants using an RNeasy Plant Mini Kit according to the manufacturer's protocol (Qiagen). RNA was cleaned from DNA contamination using TURBO DNA‐free™ Kit (ThermoScientific). To determine the expression level of transgenes, first‐strand cDNA was synthesized from 1 μg of total RNA with a High‐Capacity cDNA Reverse Transcription Kit (ThermoScientific), and then droplet digital PCR (ddPCR) was performed using gene‐specific primer sets (Table S5) in a total volume of 20 μL, using 5 μL of 25X diluted cDNA according to the manufacturer's protocol (Bio‐Rad, Hercules, CA). The data were normalized to a sorghum reference gene Eukaryotic initiation factor‐4A, EIF4A (Sudhakar Reddy *et al*., 2016). For transcriptome analysis, RNAseq was carried out by a company (BGI North America). The leaves collected at the 2021 field were used for the transcriptome analysis.

### Transcriptome analysis

Differential gene expression between WT and high‐oil *S. bicolor* was analysed with DEseq2 (Love *et al*., 2014) using R‐4.2.2. Thresholds for significant differential expression were set to log_2_foldchange > 2 and *P*adj < 0.05. Significantly up or down‐regulated genes were characterized with GO term enrichment analysis using the Database for Annotation, Visualization and Integrated Discovery (DAVID) (Huang *et al*., 2009; Sherman *et al*., 2022).

To identify candidate homologues, we used known *A. thaliana* genes as queries to search across the *S. bicolor* genome with protein BLAST (version 2.8.1) (Altschul *et al*., 1997). The group of *S. bicolor* genes with percent identity scores that were similarly ranked as top hits were further analysed. For each gene family (e.g., hexokinase), the known *A. thaliana* genes and high‐ranking *S. bicolor* genes were analysed by multiple sequence analysis (Clustal Omega version 2.1) (Sievers *et al*., 2011). The gene(s) found to still have high percent identity with the *A. thaliana* genes were designated as candidate homologues. Characterization of candidate homologues, including percent identities, predicted subcellular localization (TargetP version 2.0) (Armenteros *et al*., 2019), and independent predictions of orthologous relationships (PANTHER version 16.0) (Mi *et al*., 2021), are provided in Table S4.

### Acyl‐ACP thioesterase activity assay

Acyl‐ACP thioesterase activity assay was examined from the leaves of GH‐grown sorghum plants at the vegetative stage. Total crude protein extracts were prepared by grinding sorghum leaves in 100 mM Tris (pH 8.5) buffer with 1 mM EDTA. After centrifugation to remove cell debris, the enzyme activity assay was carried out similar to the method described previously (Gan *et al*., 2022). Details are provided in Experimental Procedures in Data S1.

### ^13^C labelling

Seeds for WT and transgenic *S. bicolor* to be used for ^13^C labelling were planted in 12‐inch pots containing PRO‐MIX BX soil and 30 g Osmocote Plus slow‐release fertilizer pellets. They were grown in a greenhouse at 15–22 °C with a 16/8 h photoperiod. [^13^C_2_]acetate labelling was performed on leaf discs (1.0 cm diameter, ~3.7 mg DW) excised from 9‐week‐old plants that each had about 10 attached leaves on the main stalk. The leaves used for labelling ranged from still expanding (~1 week old; denoted as Leaf 1) to starting to senesce (~6 weeks old; denoted as Leaf 5). Details of labelling studies are provided in Experimental Procedures in Data S1.

## Author contributions

KP, TEC, and EBC conceived the study. KP, TJC, JS, and EBC wrote the manuscript. KP generated expression constructs and conducted field studies, sorghum lipid analyses, and carbohydrate analyses. TEC managed generation of transgenic plants and TZ, MW, and SS generated transgenic plants. KP, TEC, and EBC managed field evaluations. TQ contributed to field evaluations and conducted gas exchange measurement and gene expression analyses of engineered sorghum. CZ managed initial transcriptomic analyses. HK conducted thioesterase assays and contributed to field studies. TJC conducted flux analyses and TJC and JS evaluated resulting data. MG contributed to field studies. JS conducted comparative analysis of gene expression data and led research on *WRI1* promoter binding measurements. TJN contributed to field evaluations and lipid analyses. RB and YB contributed to acquisition of transcriptomic data. SPM and KS contributed to experimental design and data interpretation. EBC provided overall supervision for the project.

## Conflict of interest

The authors declare no competing financial interests.

## Accession numbers

RNA sequencing data is available at the Gene Expression Omnibus (GEO) under the accession GSE272636.

## Supporting information


**Figure S1** Distribution of leaf TAG levels of T0 transgenic sorghum events carrying oil vector constructs (Figure 2).
**Figure S2** Oil sorghum in the greenhouse.
**Figure S3** Fatty acid profiling of TAG of leaves and stalks from field‐grown oil sorghum event in 2022.
**Figure S4** Photosynthetic parameters of field‐grown oil sorghum during the 2022 season.
**Figure S5** Galactolipids and neutral lipid content in oil sorghum leaves.
**Figure S6** Non‐structural carbohydrate content in oil sorghum events.
**Figure S7** Promoter region of sorghum plastidic homomeric ACCase (Sobic.006G030100).
**Figure S8** Acyl‐ACP thioesterase activity assay.
**Figure S9** Acetate labelling in *S. bicolor* leaves.

**Table S1** Expression level of transgenes and lipid content of GH‐grown transgenic sorghum events carrying pPTN1569.

**Table S2** Differential gene expression in WT and high‐oil leaves.

**Table S3** GO analysis on differentially expressed genes.

**Table S4** *Sorghum bicolor* candidate homologues to *Arabidopsis thaliana* central metabolism genes.

**Table S5** Primers used in this study.

**Data S1** Supporting experimental procedures.

## Data Availability

The data that support the findings of this study are openly available in Gene Expression Omnibus (GEO) at https://www.ncbi.nlm.nih.gov/geo/, reference number GSE272636.
