# Supplementary material for: Development of vegetative oil sorghum: From lab‐to‐field
Source: Plant Biotechnol J. 2024 Nov 30;23(2):660–73. doi: 10.1111/pbi.14527 (PMC11772366; doi:10.1111/pbi.14527)
Supplement: Supplementary file 1 — Figure S1 Distribution of leaf TAG levels of T0 transgenic sorghum events carrying oil vector constructs (Figure 2). Figure S2 Oil sorghum in the greenhouse. Figure S3 Fatty acid profiling of TAG of leaves and stalks from field‐grown oil sorghum event in 2022. Figure S4 Photosynthetic parameters of field‐grown oil sorghum during the 2022 season. Figure S5 Galactolipids and neutral lipid content in oil sorghum leaves. Figure S6 Non‐structural carbohydrate content in oil sorghum events. Figure S7 Promoter region of sorghum plastidic homomeric ACCase (Sobic.006G030100). Figure S8 Acyl‐ACP thioesterase activity assay. Figure S9 Acetate labelling in S. bicolor leaves. [file PBI-23-660-s007.docx]

Leaf TAG level (% DW)


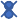


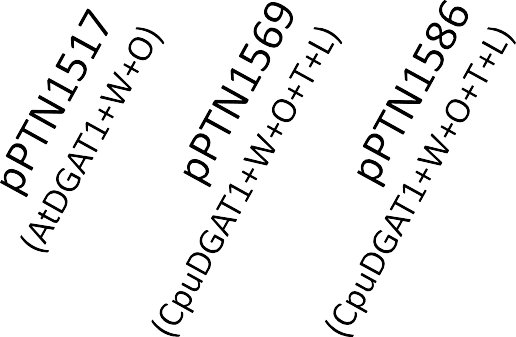

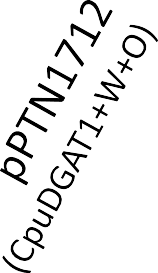


Figure S1. Distribution of leaf TAG levels of T_0_ transgenic sorghum events carrying oil vector constructs (Fig. 2). Lipids

were extracted from the leaves of transgenic sorghum and then TAG was separated and measured to screen the best oil-

accumulating events. n≥16. W, Wrinkled1; O, oleosin; T, MCFA-specialized thioesterase; L, MCFA-specialized LPAT.


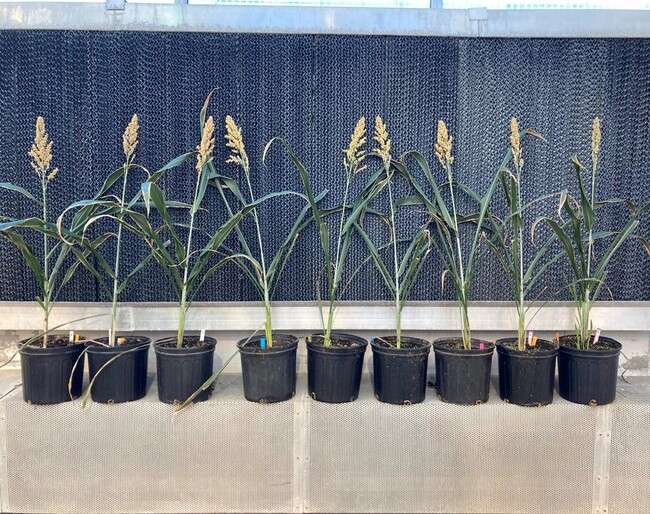


Wild-type

TZ424-5-3a

Figure S2. Oil sorghum in greenhouse. Fully-grown sorghum plants under the greenhouse condition.

100%

Leaf TAG profiling

100%

BF

AF

WT

AH

BF

AF

TZ424-5-3a

AH

Stem TAG profiling

75% 75%

BF

AF

WT

AH

BF

AF

TZ424-5-3a

AH

50% 50%

25% 25%

0% 0%

C16:0 C18:0 C18:1 C18;2 C18:3 C16:0 C18:0 C18:1 C18;2 C18:3

Figure S3. Fatty acid profiling of TAG of leaves and stalks from field-grown oil sorghum event in 2022. Leaf and stem tissues were collected at three different time points; Before flowering (BF, boot stage), after flowering (AF, after anthesis), and at harvest (AH, soft-dough stage). Fatty acids were extracted from lyophilized samples and transesterified to FAME. FAME is analyzed though GC-FID. Error bars represent ±SD (n≥4, biological replicates).

# a

ns

Net CO_2_ assimilation rate (μmol CO2 m-2 s-1)

# b

ns

Stomatal conductance (mol H2O m-2 s-1)

# c

ns

ns

ns

Transpiration rate (mmol H2O m-2 s-1)

ns

ns

ns

ns

Figure S4. Photosynthetic parameters of field-grown oil sorghum during the 2022 season. Leaf-level gas exchange was measured on oil sorghum plants at the boot stage, assessing **a** net CO₂ assimilation rate, **b** stomatal conductance, and **c** transpiration rate. Boxplots represent the interquartile range, with error bars showing the standard error (n=10–15 biological replicates.). Ns indicates no significant difference (Student’s *t*-test).

# a

6

TX430

TxHO-1

TxHO-2

~~**~~

Ramada RmHO-1 RmHO-2

~~**~~

~~**~~

~~**~~

ns

ns

ns

ns

ns

ns ns

ns

5

4

Lipid levels (% DW)

3

2

1

0

**b** 100

MGDG

DGDG

# c

100

Neutral lipids

80 80

Fatty acids composition

of MGDG (mol%)

Fatty acids composition

of DGDG (mol%)

60 60

40 40

20 20

0 0


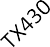

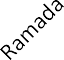

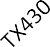

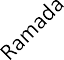


C16:0 C16:1 C18:0 C18:1 C18:2 C18:3

**d** 100

C16:0 C18:0 C18:1 C18:2 C18:3

80

Fatty acids composition

of neutral lipids (wt%)

60

40

20

0


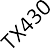

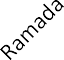


C16:0 C18:0 C18:1 C18:2 C18:3 Others

Figure S5. Galactolipids and neutral lipid content in oil sorghum leaves. Fatty acid amount (**a**) and composition of MGDG (**b**), DGDG (**c**), and neutral lipids (**d**) were analyzed using the leaves of field-grown oil sorghum events at soft-dough stage. Others represent the sum of fatty acids which are short or very long-chain

fatty acids, such as C12:0, C14:0, C20:0, C20:1, and C22:0. Error bars mean ±SD (n=4, biological replicates). Ns, not significant;

***p*<0.01, Student’s *t* test.

ns

ns

~~*~~

~~*~~

**a b c**

2.0 6 60

ns

ns

~~**~~

~~**~~

~~*~~

**

ns

ns

Starch content (g/100g DW)

D-Glucose content (g/100g DW)

Sucrose content (g/100g DW)

1.6 5 50

1.2 4 40

3 30

0.8

2 20

0.4 1 10

0.0 0 0


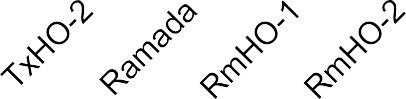

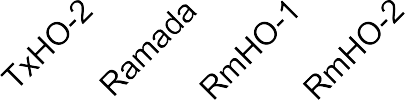


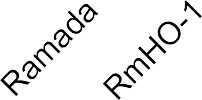


Figure S6. Non-structural carbohydrate content in oil sorghum events. Starch (**a**), D-glucose (**b**), and

sucrose content (**c**) was analyzed using field-grown oil sorghum stem tissues harvested at soft-dough stage. Error bars represent ±SD (n=4, biological replicates). **P* < 0.05, ***P* < 0.01, and ns for not significant, Student’s *t*-test.


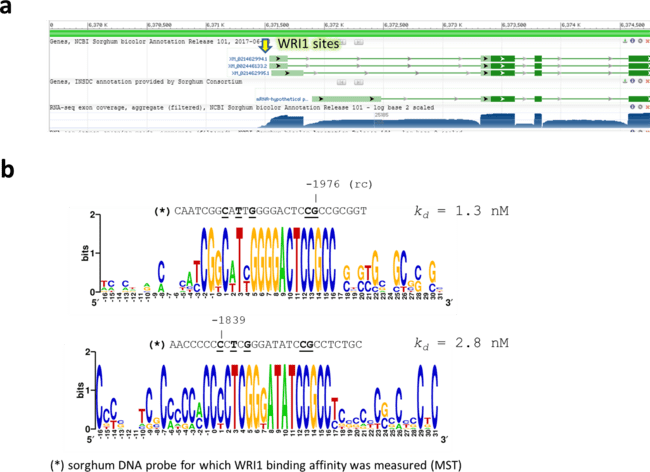


Figure S7. Promoter region of sorghum plastidic homomeric ACC (Sobic.006G030100). **a** Gene model structure of sorghum plastidic homomeric ACC found in sorghum genome at NCBI Genome Browser (gene ID 8067789 , www.ncbi.nlm.nih.gov). Three transcript variants of Sobic.006G030100; XM_021462994.1, XM_002446133.2, and XM_021462995.1 are shown. The arrow indicates the location of WRI1-binding sites (AW-box). **b** Two AW- box motifs were found in the non-coding upstream region of Sobic.006G030100 (position upstream the start codon indicated).

Sequence logos show the conservation of the AW-box sequence in the upstream regions of ACCase gene homologs in six other species (Zea mays, gene ID 542319; Oryza sativa, gene ID 4338322; Lolium perenne, gene ID 127331194; Panicum virgatum, gene ID 120639673; Brachypodium distachyon, gene ID 100846375; Setaria italica, gene ID 101781270). Bold and underlined nucleotides indicate the AW-box consensus. The binding affinity (Kd) of WRI1 to the AW-box was measured using MicroScale Thermophoresis (MST) as described in Kuczynski, C., et al. (2022). "An expanded role for the transcription factor WRINKLED1 in the biosynthesis of triacylglycerols during seed development." Front Plant Sci 13: 955589 (https://doi.org/10.3389/fpls.2022.955589).

2

*P*: 0.0069

**

1.5

Thioesterase activity (*10^3 nmol/min/mg)

1

0.5

0

TX430

14:0-ACP 18:1-ACP

TxHO-2

Figure S8. Acyl-ACP thioesterase activity assay. MCFA-specialized thioesterase (FatB) activity was measured in the presence of 14C 14:0-ACP. Enzyme reaction with 14C 18:1Δ9-ACP is to measure common thioesterase (FatA)

activity. Error bars represent ±SD (n=5, biological replicates). ***P* < 0.01, Student’s *t* test.


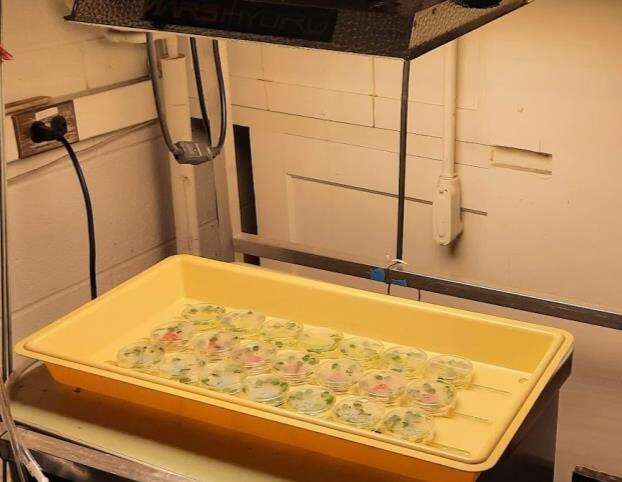
**a b**


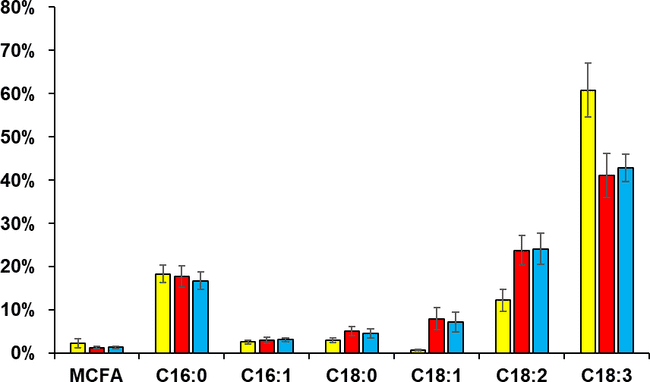


WT

TxHO-1 TxHO-2

* *

* *

* *

* *

**mol% fatty acids**

**c**


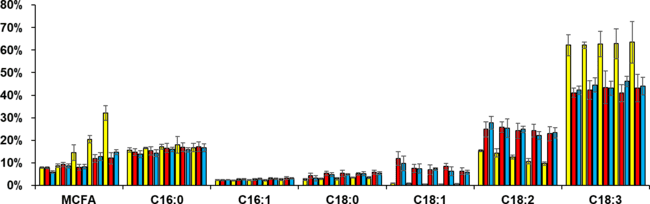

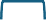


values X 10

leaf 1 leaf 5

** * * *

* *

* **

** ** ** ** **

** **

*

**

*

**

* **

*

** ** ** **

** ** ** **

**mol% fatty acids**

**d**


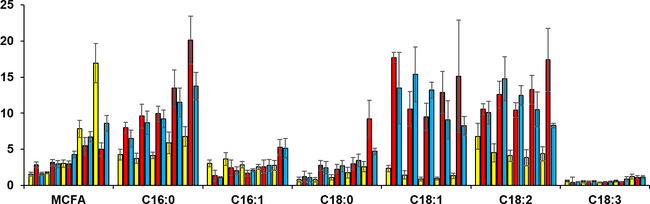


*

*

*

**

*

* *

*

*

*

*

*

*

*

*

* *

* *

**

* *

*

*

*

*

*

*

*

*

*

*

*

**

*

**

** * **

**

*

* **

*

**nmol 13C incorporated into fatty acids / mg leaf DM**

Figure S9. Acetate labeling in *S. bicolor* leaves. **a** Leaf disc punches from *S. bicolor* leaves of 5 ages were labeled with [13C_2_]acetate in solution for 2 days under continuous light (500 µmol/m2/s) and extracted for total fatty acids. Fatty acids in wildtype (yellow) and high-oil transgenic (red, blue) tissues were analyzed with GC-MS. **b** Fatty acid composition averaged over leaf age and for leaf ages in separate (**c**). Leaf 1 denotes the youngest leaf stage. **d** incorporation of 13C into leaves. Bars show averages (n=5, biological replicates), and error bars indicate SD. Asterisks indicate if there was a significant difference between the wildtype and each of the transgenic lines (two-sided t-test, p < 0.05).
