# Supplementary material for: Development of vegetative oil sorghum: From lab‐to‐field
Source: Plant Biotechnol J. 2024 Nov 30;23(2):660–73. doi: 10.1111/pbi.14527 (PMC11772366; doi:10.1111/pbi.14527)
Supplement: Supplementary file 2 — Table S1 Expression level of transgenes and lipid content of GH‐grown transgenic sorghum events carrying pPTN1569. [file PBI-23-660-s004.docx]

Table S1. Expression level of transgenes and lipid content of GH-grown transgenic sorghum events carrying pPTN1569. The expression level of transgenes in 4 independent events was normalized by EIFA4 reference gene (n=4, mean ± SD.). For lipid analysis, the amount of TFA and TAG (% DW) from leaf or stem tissues were determined via GD- FID (n=4, mean ± SD.).

| Independent events carrying pPTN1569 | Expression level of transgenes (mRNA copies/ng total RNA, V5) | | | | | Leaf (V5) (% DW) | | Leaf (AF) (% DW) | | Stem (AH) (% DW) | |
| --- | --- | --- | --- | --- | --- | --- | --- | --- | --- | --- | --- |
|  | SbWRI1 | SiOle | CpuDGAT1 | CvFATB1 | CvLPAT2 | TFA | TAG | TFA | TAG | TFA | TAG |
| ZG1054-3-30c | 458.0 ± 327.8 | 254.1 ± 274.3 | 768.7 ± 767.7 | 18.4 ± 16.9 | 164.9 ± 107.0 | 4.6 ± 0.8 | 0.1 ± 0.1 | 3.4 ± 0.1 | 0.3 ± 0.1 | 0.2 ± 0.1 | 0.0 ± 0.0 |
| TZ424-5-2c | 232.6 ± 133.9 | 593.4 ± 280.5 | 4206.4 ± 2610.4 | 11.1 ± 11.1 | 13.7 ± 2.3 | 5.3 ± 0.6 | 1 ± 0.3 | 6 ± 1.6 | 2.6 ± 1.6 | 1.5 ± 0.6 | 1.1 ± 0.5 |
| TZ424-4-5a | 703.8 ± 263.6 | 1226.7 ± 273.2 | 5246.1 ± 802.8 | 3.8 ± 4.0 | 0.4 ± 0.5 | 5.1 ± 0.4 | 1.1 ± 0.2 | 6.7 ± 1.0 | 2.4 ± 0.9 | 1.8 ± 0.4 | 1.3 ± 0.5 |
| TZ424-5-3a | 644.7 ± 118.7 | 1314.3 ± 243.7 | 12672.1 ± 3751.7 | 19.5 ± 18.7 | 19.9 ± 12.9 | 6.5 ± 0.7 | 2.3 ± 0.6 | 8.0 ± 1.6 | 3.7 ± 1.4 | 2.4 ± 1.4 | 1.7 ± 1.0 |
