# Supplementary material for: Development of vegetative oil sorghum: From lab‐to‐field
Source: Plant Biotechnol J. 2024 Nov 30;23(2):660–73. doi: 10.1111/pbi.14527 (PMC11772366; doi:10.1111/pbi.14527)
Supplement: Supplementary file 3 — Table S2 Differential gene expression in WT and high‐oil leaves. [file PBI-23-660-s003.docx]

Table S2. Differential gene expression in WT and high-oil leaves. RNAseq was performed on matur *Sorghum bicolor* leaves and analyzed with DEseq2 to determine differential gene expression. Significant (sig) up-regulation was characterized by the transgenic gene having at least |log2FC| = 2 compared to the wildtype with padj < 0.05.

| gene | baseMean | log2FC | lfcSE | stat | pvalue | padj | sig? |
| --- | --- | --- | --- | --- | --- | --- | --- |
| Sobic.007G176800 | 23607.5241 | 6.1605 | 0.2793 | 22.0598 | 7.69E-108 | 1.62E-103 | up |
| Sobic.007G112100 | 4304.5118 | 6.3802 | 0.3750 | 17.0142 | 6.45E-65 | 6.80E-61 | up |
| Sobic.005G054900 | 1849.4007 | 7.7064 | 0.4550 | 16.9378 | 2.37E-64 | 1.66E-60 | up |
| Sobic.001G155500 | 2670.9732 | 5.0709 | 0.3000 | 16.9036 | 4.23E-64 | 2.23E-60 | up |
| Sobic.001G530100 | 3835.9184 | 5.4020 | 0.3478 | 15.5312 | 2.13E-54 | 9.00E-51 | up |
| Sobic.006G041700 | 3225.2800 | 7.4771 | 0.5125 | 14.5903 | 3.24E-48 | 1.14E-44 | up |
| Sobic.004G330600 | 6482.1418 | 4.1611 | 0.2897 | 14.3627 | 8.86E-47 | 2.67E-43 | up |
| Sobic.002G073700 | 4438.8805 | 3.9304 | 0.2960 | 13.2794 | 3.05E-40 | 8.04E-37 | up |
| Sobic.007G134800 | 4023.7106 | 5.3125 | 0.4235 | 12.5437 | 4.30E-36 | 1.01E-32 | up |
| Sobic.003G309300 | 3798.8776 | 3.4779 | 0.2813 | 12.3626 | 4.16E-35 | 8.78E-32 | up |
| Sobic.002G256900 | 2454.2377 | 9.1070 | 0.7541 | 12.0765 | 1.41E-33 | 2.69E-30 | up |
| Sobic.001G088200 | 8257.4982 | 2.7487 | 0.2313 | 11.8828 | 1.45E-32 | 2.56E-29 | up |
| Sobic.009G052200 | 5867.0300 | 4.1651 | 0.3551 | 11.7302 | 8.92E-32 | 1.45E-28 | up |
| Sobic.002G256800 | 5604.9910 | 2.7263 | 0.2376 | 11.4732 | 1.80E-30 | 2.71E-27 | up |
| Sobic.001G403400 | 14094.4545 | 4.1860 | 0.3660 | 11.4363 | 2.75E-30 | 3.87E-27 | up |
| Sobic.006G167700 | 514.9526 | 9.3698 | 0.8218 | 11.4020 | 4.09E-30 | 5.38E-27 | up |
| Sobic.008G173000 | 14328.6005 | 3.1926 | 0.2803 | 11.3889 | 4.75E-30 | 5.89E-27 | up |
| Sobic.001G261513 | 2039.4373 | -2.9811 | 0.2662 | -11.1989 | 4.13E-29 | 4.84E-26 | down |
| Sobic.001G005700 | 186.6830 | 5.4976 | 0.4930 | 11.1512 | 7.06E-29 | 7.84E-26 | up |
| Sobic.006G043700 | 13217.4296 | 3.3120 | 0.2999 | 11.0425 | 2.38E-28 | 2.51E-25 | up |
| Sobic.003G431100 | 287.6965 | 5.2099 | 0.4750 | 10.9674 | 5.48E-28 | 5.51E-25 | up |
| Sobic.010G073500 | 6391.8428 | 3.4001 | 0.3245 | 10.4767 | 1.11E-25 | 1.06E-22 | up |
| Sobic.009G154700 | 3355.3820 | 4.2396 | 0.4066 | 10.4258 | 1.89E-25 | 1.73E-22 | up |
| Sobic.003G259300 | 393.0981 | 4.4893 | 0.4361 | 10.2942 | 7.49E-25 | 6.58E-22 | up |
| Sobic.006G237000 | 2436.4072 | 3.2116 | 0.3134 | 10.2491 | 1.19E-24 | 1.01E-21 | up |
| Sobic.008G190100 | 6161.2925 | 2.9493 | 0.2904 | 10.1572 | 3.08E-24 | 2.50E-21 | up |
| Sobic.001G434700 | 2700.9885 | 3.2906 | 0.3453 | 9.5290 | 1.59E-21 | 1.24E-18 | up |
| Sobic.006G011200 | 26537.8194 | 3.0362 | 0.3194 | 9.5056 | 1.99E-21 | 1.50E-18 | up |
| Sobic.003G096000 | 2060.9907 | 2.6211 | 0.2774 | 9.4486 | 3.43E-21 | 2.50E-18 | up |
| Sobic.007G043400 | 2762.2577 | 3.0373 | 0.3231 | 9.4000 | 5.46E-21 | 3.84E-18 | up |
| Sobic.005G147600 | 136.5383 | 7.1483 | 0.7640 | 9.3561 | 8.28E-21 | 5.63E-18 | up |
| Sobic.003G048200 | 101.6977 | -4.1012 | 0.4400 | -9.3212 | 1.15E-20 | 7.58E-18 | down |
| Sobic.004G107800 | 204.7391 | 3.0172 | 0.3277 | 9.2081 | 3.32E-20 | 2.12E-17 | up |
| Sobic.001G198800 | 938.9215 | -3.6780 | 0.4093 | -8.9852 | 2.58E-19 | 1.60E-16 | down |
| Sobic.001G278600 | 146.0693 | 6.3604 | 0.7176 | 8.8629 | 7.80E-19 | 4.70E-16 | up |
| Sobic.007G025700 | 437.0869 | 2.7053 | 0.3144 | 8.6048 | 7.65E-18 | 4.48E-15 | up |
| Sobic.001G290600 | 12978.9003 | 2.2859 | 0.2663 | 8.5832 | 9.23E-18 | 5.26E-15 | up |
| Sobic.008G059900 | 4208.3711 | 3.6976 | 0.4361 | 8.4784 | 2.28E-17 | 1.27E-14 | up |
| Sobic.002G280400 | 14213.7718 | 2.3955 | 0.2827 | 8.4737 | 2.38E-17 | 1.29E-14 | up |
| Sobic.008G161500 | 171.7702 | 10.6381 | 1.2583 | 8.4543 | 2.81E-17 | 1.48E-14 | up |
| Sobic.004G234100 | 1564.1190 | 2.5905 | 0.3127 | 8.2839 | 1.19E-16 | 5.99E-14 | up |
| Sobic.009G216800 | 1500.4272 | 3.6829 | 0.4445 | 8.2861 | 1.17E-16 | 5.99E-14 | up |
| Sobic.005G048700 | 361.0879 | 3.2735 | 0.3993 | 8.1988 | 2.43E-16 | 1.19E-13 | up |
| Sobic.004G259366 | 124.5650 | 3.2700 | 0.4166 | 7.8486 | 4.21E-15 | 2.02E-12 | up |
| Sobic.006G030100 | 22439.6037 | 3.2362 | 0.4127 | 7.8406 | 4.48E-15 | 2.10E-12 | up |
| Sobic.009G150700 | 312.8334 | 2.2401 | 0.2937 | 7.6280 | 2.38E-14 | 1.09E-11 | up |
| Sobic.010G236100 | 338.4499 | 3.3610 | 0.4437 | 7.5754 | 3.58E-14 | 1.61E-11 | up |
| Sobic.003G212300 | 226.4713 | 2.4498 | 0.3247 | 7.5453 | 4.51E-14 | 1.98E-11 | up |
| Sobic.003G244700 | 433.7883 | 3.2124 | 0.4302 | 7.4666 | 8.23E-14 | 3.54E-11 | up |
| Sobic.004G044401 | 228.1786 | 2.8372 | 0.3911 | 7.2552 | 4.01E-13 | 1.69E-10 | up |
| Sobic.004G025400 | 1208.2398 | 2.1714 | 0.3006 | 7.2232 | 5.08E-13 | 2.10E-10 | up |
| Sobic.004G259432 | 78.9892 | 3.4806 | 0.4854 | 7.1710 | 7.45E-13 | 3.02E-10 | up |

| Sobic.001G051600 | 5600.2212 | 2.9723 | 0.4151 | 7.1612 | 8.00E-13 | 3.18E-10 | up |
| --- | --- | --- | --- | --- | --- | --- | --- |
| Sobic.005G224700 | 55.1016 | -5.2673 | 0.7441 | -7.0788 | 1.45E-12 | 5.68E-10 | down |
| Sobic.003G222800 | 186.0267 | 4.1952 | 0.6013 | 6.9766 | 3.02E-12 | 1.16E-09 | up |
| Sobic.001G403800 | 195.8924 | 3.1624 | 0.4556 | 6.9412 | 3.89E-12 | 1.46E-09 | up |
| Sobic.007G187900 | 1664.8689 | 3.4851 | 0.5038 | 6.9174 | 4.60E-12 | 1.70E-09 | up |
| Sobic.001G422300 | 62.2195 | -5.5733 | 0.8112 | -6.8706 | 6.39E-12 | 2.32E-09 | down |
| Sobic.001G280800 | 97.5280 | 9.8211 | 1.4634 | 6.7112 | 1.93E-11 | 6.90E-09 | up |
| Sobic.002G311400 | 542.0878 | 3.8753 | 0.5802 | 6.6795 | 2.40E-11 | 8.43E-09 | up |
| Sobic.010G146100 | 1059.0627 | -1.8378 | 0.2752 | -6.6769 | 2.44E-11 | 8.44E-09 | no |
| Sobic.010G156100 | 150.5022 | 2.9595 | 0.4471 | 6.6192 | 3.61E-11 | 1.23E-08 | up |
| Sobic.004G193400 | 47018.7621 | -2.1793 | 0.3313 | -6.5781 | 4.76E-11 | 1.59E-08 | down |
| Sobic.007G079600 | 1021.1891 | 4.7012 | 0.7223 | 6.5091 | 7.56E-11 | 2.49E-08 | up |
| Sobic.001G196300 | 1006.7807 | 2.1058 | 0.3245 | 6.4885 | 8.67E-11 | 2.77E-08 | up |
| Sobic.002G054400 | 6679.5948 | 2.7090 | 0.4174 | 6.4905 | 8.55E-11 | 2.77E-08 | up |
| Sobic.009G203600 | 439.9168 | 2.2798 | 0.3539 | 6.4425 | 1.18E-10 | 3.70E-08 | up |
| Sobic.009G025700 | 137.3931 | 5.1074 | 0.7975 | 6.4045 | 1.51E-10 | 4.68E-08 | up |
| Sobic.010G006400 | 1608.1975 | -3.3340 | 0.5210 | -6.3990 | 1.56E-10 | 4.78E-08 | down |
| Sobic.003G234101 | 178.0669 | -4.5772 | 0.7263 | -6.3025 | 2.93E-10 | 8.83E-08 | down |
| Sobic.003G357100 | 166.2854 | 2.3918 | 0.3803 | 6.2894 | 3.19E-10 | 9.47E-08 | up |
| Sobic.005G039100 | 484.2267 | 2.5812 | 0.4106 | 6.2865 | 3.25E-10 | 9.51E-08 | up |
| Sobic.001G537100 | 1031.1980 | 2.3042 | 0.3667 | 6.2830 | 3.32E-10 | 9.59E-08 | up |
| Sobic.002G420800 | 4421.7795 | 5.1536 | 0.8282 | 6.2227 | 4.89E-10 | 1.39E-07 | up |
| Sobic.004G351700 | 3343.9275 | -2.0176 | 0.3255 | -6.1988 | 5.69E-10 | 1.60E-07 | down |
| Sobic.005G115800 | 784.0944 | -3.6582 | 0.5913 | -6.1865 | 6.15E-10 | 1.69E-07 | down |
| Sobic.001G217700 | 1591.8454 | -1.7070 | 0.2759 | -6.1862 | 6.16E-10 | 1.69E-07 | no |
| Sobic.008G030100 | 1988.3685 | 1.8258 | 0.2961 | 6.1661 | 7.00E-10 | 1.89E-07 | no |
| Sobic.001G107800 | 163.2866 | -9.1944 | 1.5060 | -6.1051 | 1.03E-09 | 2.74E-07 | down |
| Sobic.010G242200 | 72.5573 | 3.7899 | 0.6233 | 6.0807 | 1.20E-09 | 3.15E-07 | up |
| Sobic.004G243400 | 365.5619 | -2.1391 | 0.3544 | -6.0354 | 1.59E-09 | 4.08E-07 | down |
| Sobic.010G163000 | 431.7467 | 2.1502 | 0.3563 | 6.0355 | 1.58E-09 | 4.08E-07 | up |
| Sobic.005G134501 | 200.7511 | 3.8235 | 0.6361 | 6.0113 | 1.84E-09 | 4.68E-07 | up |
| Sobic.002G319900 | 85.2269 | 4.4159 | 0.7379 | 5.9848 | 2.17E-09 | 5.44E-07 | up |
| Sobic.009G009600 | 227.6859 | 3.8833 | 0.6518 | 5.9575 | 2.56E-09 | 6.36E-07 | up |
| Sobic.003G223400 | 3346.4643 | -2.6533 | 0.4458 | -5.9512 | 2.66E-09 | 6.53E-07 | down |
| Sobic.004G161301 | 1905.7803 | -2.5884 | 0.4373 | -5.9191 | 3.24E-09 | 7.76E-07 | down |
| Sobic.002G333300 | 189.8230 | 2.1794 | 0.3682 | 5.9198 | 3.22E-09 | 7.76E-07 | up |
| Sobic.002G214300 | 881.3976 | -2.0649 | 0.3503 | -5.8948 | 3.75E-09 | 8.89E-07 | down |
| Sobic.009G119200 | 20332.3802 | -4.0388 | 0.6866 | -5.8824 | 4.04E-09 | 9.48E-07 | down |
| Sobic.001G520400 | 703.2741 | 3.8729 | 0.6607 | 5.8617 | 4.58E-09 | 1.06E-06 | up |
| Sobic.010G247200 | 508.5440 | 3.0240 | 0.5174 | 5.8443 | 5.09E-09 | 1.17E-06 | up |
| Sobic.003G080300 | 94.4132 | 3.5985 | 0.6172 | 5.8303 | 5.53E-09 | 1.25E-06 | up |
| Sobic.008G152000 | 821.6053 | 1.7831 | 0.3059 | 5.8285 | 5.59E-09 | 1.26E-06 | no |
| Sobic.010G270100 | 146.2242 | 3.2673 | 0.5610 | 5.8239 | 5.75E-09 | 1.28E-06 | up |
| Sobic.010G179500 | 143.7645 | -3.0534 | 0.5256 | -5.8090 | 6.29E-09 | 1.38E-06 | down |
| Sobic.002G230100 | 11305.7332 | 1.7733 | 0.3065 | 5.7863 | 7.20E-09 | 1.56E-06 | no |
| Sobic.003G057800 | 281.5922 | -3.7895 | 0.6556 | -5.7800 | 7.47E-09 | 1.61E-06 | down |
| Sobic.005G106500 | 2121.7257 | 1.6792 | 0.2914 | 5.7620 | 8.31E-09 | 1.77E-06 | no |
| Sobic.002G242000 | 94948.4349 | -2.1611 | 0.3774 | -5.7262 | 1.03E-08 | 2.17E-06 | down |
| Sobic.001G409200 | 45.3104 | 3.4982 | 0.6151 | 5.6873 | 1.29E-08 | 2.70E-06 | up |
| Sobic.003G239300 | 341.3976 | -2.8890 | 0.5112 | -5.6512 | 1.59E-08 | 3.26E-06 | down |
| Sobic.001G064200 | 9789.0083 | 2.3088 | 0.4085 | 5.6524 | 1.58E-08 | 3.26E-06 | up |
| Sobic.007G039300 | 882.3758 | 1.7064 | 0.3020 | 5.6495 | 1.61E-08 | 3.26E-06 | no |
| Sobic.002G355200 | 85.1655 | 3.4083 | 0.6044 | 5.6394 | 1.71E-08 | 3.43E-06 | up |
| Sobic.002G159900 | 3650.4007 | 2.9789 | 0.5288 | 5.6329 | 1.77E-08 | 3.53E-06 | up |
| Sobic.003G352300 | 97.7932 | 3.6272 | 0.6442 | 5.6308 | 1.79E-08 | 3.53E-06 | up |
| Sobic.003G432100 | 934.6043 | 2.1993 | 0.3920 | 5.6104 | 2.02E-08 | 3.94E-06 | up |
| Sobic.006G266800 | 34.6773 | 5.8580 | 1.0445 | 5.6087 | 2.04E-08 | 3.94E-06 | up |

| Sobic.004G337066 | 33.9005 | 5.8468 | 1.0474 | 5.5824 | 2.37E-08 | 4.55E-06 | up |
| --- | --- | --- | --- | --- | --- | --- | --- |
| Sobic.007G052700 | 2726.8205 | 1.9696 | 0.3535 | 5.5711 | 2.53E-08 | 4.81E-06 | no |
| Sobic.006G153500 | 175.9515 | -3.9875 | 0.7175 | -5.5579 | 2.73E-08 | 5.14E-06 | down |
| Sobic.003G326400 | 100.6234 | -8.4543 | 1.5230 | -5.5510 | 2.84E-08 | 5.30E-06 | down |
| Sobic.010G268400 | 948.7942 | 1.5342 | 0.2768 | 5.5427 | 2.98E-08 | 5.51E-06 | no |
| Sobic.009G073500 | 103.7994 | 2.2515 | 0.4096 | 5.4967 | 3.87E-08 | 7.10E-06 | up |
| Sobic.001G422366 | 170.2652 | -2.5815 | 0.4699 | -5.4935 | 3.94E-08 | 7.17E-06 | down |
| Sobic.004G205100 | 3691.5030 | 2.6471 | 0.4835 | 5.4748 | 4.38E-08 | 7.89E-06 | up |
| Sobic.003G316300 | 1624.6920 | 1.5130 | 0.2768 | 5.4661 | 4.60E-08 | 8.22E-06 | no |
| Sobic.001G341600 | 122.5411 | 1.9223 | 0.3540 | 5.4296 | 5.65E-08 | 1.00E-05 | no |
| Sobic.009G043600 | 618.2324 | 2.9354 | 0.5415 | 5.4207 | 5.94E-08 | 1.04E-05 | up |
| Sobic.002G064000 | 1007.4599 | -3.5656 | 0.6605 | -5.3979 | 6.74E-08 | 1.17E-05 | down |
| Sobic.001G175700 | 10830.2479 | -1.5200 | 0.2818 | -5.3941 | 6.89E-08 | 1.19E-05 | no |
| Sobic.001G098600 | 4745.2650 | -1.9612 | 0.3640 | -5.3882 | 7.12E-08 | 1.22E-05 | no |
| Sobic.002G345850 | 801.8725 | -1.8227 | 0.3393 | -5.3722 | 7.78E-08 | 1.32E-05 | no |
| Sobic.003G095200 | 105.6967 | 2.0046 | 0.3733 | 5.3696 | 7.89E-08 | 1.33E-05 | up |
| Sobic.001G410450 | 543.7131 | 3.4522 | 0.6435 | 5.3647 | 8.11E-08 | 1.36E-05 | up |
| Sobic.001G526900 | 108.9759 | 2.8673 | 0.5351 | 5.3585 | 8.39E-08 | 1.39E-05 | up |
| Sobic.009G010700 | 9806.0159 | -3.7655 | 0.7040 | -5.3487 | 8.86E-08 | 1.46E-05 | down |
| Sobic.001G482700 | 225.2092 | 2.2472 | 0.4223 | 5.3209 | 1.03E-07 | 1.69E-05 | up |
| Sobic.007G075700 | 1727.0002 | -2.1127 | 0.3977 | -5.3119 | 1.08E-07 | 1.75E-05 | down |
| Sobic.006G030800 | 1329.2828 | 1.7495 | 0.3294 | 5.3112 | 1.09E-07 | 1.75E-05 | no |
| Sobic.002G254100 | 108.8835 | -2.1920 | 0.4129 | -5.3083 | 1.11E-07 | 1.76E-05 | down |
| Sobic.002G140400 | 474.5883 | 2.2569 | 0.4252 | 5.3079 | 1.11E-07 | 1.76E-05 | up |
| Sobic.003G098700 | 905.1175 | 2.0426 | 0.3856 | 5.2965 | 1.18E-07 | 1.86E-05 | up |
| Sobic.008G036900 | 552.6170 | -1.5159 | 0.2871 | -5.2807 | 1.29E-07 | 2.01E-05 | no |
| Sobic.004G359300 | 1162.0256 | -2.0129 | 0.3814 | -5.2775 | 1.31E-07 | 2.03E-05 | down |
| Sobic.006G205100 | 3752.5700 | -1.6665 | 0.3173 | -5.2528 | 1.50E-07 | 2.31E-05 | no |
| Sobic.010G001300 | 560.5349 | 2.1969 | 0.4184 | 5.2507 | 1.52E-07 | 2.32E-05 | up |
| Sobic.003G209900 | ######### | -2.5186 | 0.4834 | -5.2102 | 1.89E-07 | 2.86E-05 | down |
| Sobic.003G304100 | 1127.9514 | 3.0615 | 0.5882 | 5.2047 | 1.94E-07 | 2.93E-05 | up |
| Sobic.004G005000 | 236.7298 | -2.6581 | 0.5112 | -5.1993 | 2.00E-07 | 2.95E-05 | down |
| Sobic.010G066200 | 583.9231 | 1.6320 | 0.3138 | 5.2013 | 1.98E-07 | 2.95E-05 | no |
| Sobic.001G349800 | 612.8939 | 2.5261 | 0.4858 | 5.1999 | 1.99E-07 | 2.95E-05 | up |
| Sobic.003G215200 | 56.7659 | -3.3747 | 0.6494 | -5.1966 | 2.03E-07 | 2.97E-05 | down |
| Sobic.005G114000 | 242.0660 | -2.0060 | 0.3861 | -5.1952 | 2.05E-07 | 2.97E-05 | down |
| Sobic.007G035500 | 110.0077 | 5.9444 | 1.1478 | 5.1790 | 2.23E-07 | 3.22E-05 | up |
| Sobic.001G474500 | 2104.7968 | 1.3361 | 0.2582 | 5.1744 | 2.29E-07 | 3.28E-05 | no |
| Sobic.001G474200 | 39.9100 | 3.1843 | 0.6160 | 5.1696 | 2.35E-07 | 3.34E-05 | up |
| Sobic.002G359600 | 1059.4109 | -2.0356 | 0.3940 | -5.1660 | 2.39E-07 | 3.39E-05 | down |
| Sobic.005G137100 | 37.1416 | 3.9375 | 0.7628 | 5.1621 | 2.44E-07 | 3.43E-05 | up |
| Sobic.009G157100 | 1164.5749 | -1.6528 | 0.3207 | -5.1538 | 2.55E-07 | 3.56E-05 | no |
| Sobic.009G085100 | 17285.0124 | -1.6986 | 0.3299 | -5.1486 | 2.62E-07 | 3.64E-05 | no |
| Sobic.009G159100 | 611.5218 | -2.2701 | 0.4411 | -5.1467 | 2.65E-07 | 3.65E-05 | down |
| Sobic.004G095400 | 195.6817 | 1.5176 | 0.2953 | 5.1386 | 2.77E-07 | 3.79E-05 | no |
| Sobic.001G037300 | 53.9199 | -2.7760 | 0.5420 | -5.1219 | 3.02E-07 | 4.12E-05 | down |
| Sobic.001G376200 | 41.6854 | 4.4235 | 0.8658 | 5.1091 | 3.24E-07 | 4.38E-05 | up |
| Sobic.006G182500 | 134.7257 | 2.0597 | 0.4071 | 5.0588 | 4.22E-07 | 5.67E-05 | up |
| Sobic.003G029000 | 164.2620 | 1.7256 | 0.3412 | 5.0575 | 4.25E-07 | 5.67E-05 | no |
| Sobic.008G157100 | 7749.8158 | -3.3030 | 0.6544 | -5.0470 | 4.49E-07 | 5.95E-05 | down |
| Sobic.006G026400 | 154.6851 | -5.3995 | 1.0715 | -5.0390 | 4.68E-07 | 6.17E-05 | down |
| Sobic.003G099400 | 339.4665 | -4.4556 | 0.8858 | -5.0302 | 4.90E-07 | 6.42E-05 | down |
| Sobic.003G333800 | 1812.3789 | 1.2135 | 0.2419 | 5.0165 | 5.26E-07 | 6.85E-05 | no |
| Sobic.003G022000 | 98.7080 | 2.7742 | 0.5536 | 5.0109 | 5.42E-07 | 7.01E-05 | up |
| Sobic.004G236550 | 76.1295 | 2.9019 | 0.5813 | 4.9917 | 5.98E-07 | 7.70E-05 | up |
| Sobic.005G018700 | 149.3670 | 2.3434 | 0.4697 | 4.9888 | 6.07E-07 | 7.76E-05 | up |
| Sobic.005G139900 | 8230.7364 | -2.0042 | 0.4021 | -4.9847 | 6.21E-07 | 7.86E-05 | down |

| Sobic.007G107000 | 521.6484 | 2.0137 | 0.4040 | 4.9842 | 6.22E-07 | 7.86E-05 | up |
| --- | --- | --- | --- | --- | --- | --- | --- |
| Sobic.002G411900 | 155.0045 | -5.5160 | 1.1100 | -4.9693 | 6.72E-07 | 8.44E-05 | down |
| Sobic.002G040100 | 3075.0560 | -2.7328 | 0.5511 | -4.9588 | 7.09E-07 | 8.85E-05 | down |
| Sobic.008G186600 | 241.6275 | 1.7391 | 0.3513 | 4.9501 | 7.42E-07 | 9.20E-05 | no |
| Sobic.007G050100 | 8697.5205 | -1.4865 | 0.3009 | -4.9409 | 7.78E-07 | 9.59E-05 | no |
| Sobic.001G524750 | 333.3269 | 2.2433 | 0.4546 | 4.9345 | 8.03E-07 | 9.85E-05 | up |
| Sobic.001G073700 | 4039.7324 | 1.5966 | 0.3249 | 4.9142 | 8.91E-07 | 0.0001085 | no |
| Sobic.002G195900 | 601.3149 | 2.0590 | 0.4190 | 4.9134 | 8.95E-07 | 0.0001085 | up |
| Sobic.001G486100 | 3044.2158 | -1.5700 | 0.3197 | -4.9101 | 9.10E-07 | 0.0001097 | no |
| Sobic.003G041600 | 199.9284 | -1.8085 | 0.3701 | -4.8865 | 1.03E-06 | 0.000123 | no |
| Sobic.010G159000 | 351.8996 | -2.3557 | 0.4834 | -4.8736 | 1.10E-06 | 0.0001305 | down |
| Sobic.007G064250 | 558.3224 | 1.7794 | 0.3652 | 4.8724 | 1.10E-06 | 0.0001306 | no |
| Sobic.006G022200 | 133.3989 | 3.2172 | 0.6643 | 4.8432 | 1.28E-06 | 0.0001505 | up |
| Sobic.004G222100 | 268.0894 | 1.4824 | 0.3070 | 4.8280 | 1.38E-06 | 0.0001616 | no |
| Sobic.003G348700 | 26.0471 | 6.0526 | 1.2560 | 4.8191 | 1.44E-06 | 0.000168 | up |
| Sobic.006G267000 | 327.0326 | 2.8118 | 0.5841 | 4.8143 | 1.48E-06 | 0.0001712 | up |
| Sobic.003G017200 | 3171.0451 | -1.7578 | 0.3661 | -4.8018 | 1.57E-06 | 0.0001812 | no |
| Sobic.001G117300 | 3235.9537 | 1.4192 | 0.2966 | 4.7844 | 1.71E-06 | 0.0001965 | no |
| Sobic.007G158500 | 371.9566 | 1.7955 | 0.3754 | 4.7826 | 1.73E-06 | 0.0001972 | no |
| Sobic.002G255600 | 477.0616 | 1.3742 | 0.2877 | 4.7761 | 1.79E-06 | 0.0002027 | no |
| Sobic.001G275600 | 48.8351 | 3.8427 | 0.8050 | 4.7734 | 1.81E-06 | 0.0002043 | up |
| Sobic.010G267100 | 188.8489 | 2.6159 | 0.5482 | 4.7713 | 1.83E-06 | 0.0002053 | up |
| Sobic.003G136900 | 5264.1166 | -1.4781 | 0.3105 | -4.7605 | 1.93E-06 | 0.0002155 | no |
| Sobic.002G193200 | 13.7082 | 6.9909 | 1.4717 | 4.7502 | 2.03E-06 | 0.0002255 | up |
| Sobic.006G052900 | 38.5663 | 6.6973 | 1.4110 | 4.7465 | 2.07E-06 | 0.0002285 | up |
| Sobic.006G237600 | 23.6899 | -6.3728 | 1.3473 | -4.7301 | 2.24E-06 | 0.0002464 | down |
| Sobic.005G090600 | 195.2297 | -3.3406 | 0.7081 | -4.7174 | 2.39E-06 | 0.000261 | down |
| Sobic.007G162400 | 39.9413 | -4.6620 | 0.9890 | -4.7138 | 2.43E-06 | 0.0002643 | down |
| Sobic.001G022900 | 4949.6374 | 1.4047 | 0.2981 | 4.7115 | 2.46E-06 | 0.000266 | no |
| Sobic.009G016800 | 33.9662 | 3.4377 | 0.7309 | 4.7032 | 2.56E-06 | 0.0002755 | up |
| Sobic.009G183300 | 104.2420 | 2.7424 | 0.5840 | 4.6960 | 2.65E-06 | 0.000284 | up |
| Sobic.006G019600 | 41.1155 | 7.6081 | 1.6205 | 4.6949 | 2.67E-06 | 0.0002842 | up |
| Sobic.010G172200 | 236.5098 | -2.2585 | 0.4818 | -4.6873 | 2.77E-06 | 0.0002933 | down |
| Sobic.005G212700 | 73.7625 | 3.7746 | 0.8057 | 4.6846 | 2.80E-06 | 0.0002957 | up |
| Sobic.006G260300 | 170.5232 | 2.2330 | 0.4769 | 4.6828 | 2.83E-06 | 0.0002968 | up |
| Sobic.001G317800 | 5149.7499 | 2.2293 | 0.4770 | 4.6736 | 2.96E-06 | 0.0003089 | up |
| Sobic.004G310900 | 1222.7439 | 1.4702 | 0.3147 | 4.6714 | 2.99E-06 | 0.0003093 | no |
| Sobic.009G215700 | 16.2502 | 7.2353 | 1.5487 | 4.6718 | 2.99E-06 | 0.0003093 | up |
| Sobic.009G125500 | 101.1352 | -2.9793 | 0.6384 | -4.6667 | 3.06E-06 | 0.0003149 | down |
| Sobic.003G440500 | 776.0231 | -2.3021 | 0.4939 | -4.6611 | 3.14E-06 | 0.0003219 | down |
| Sobic.001G265500 | 124.1993 | 1.7845 | 0.3832 | 4.6564 | 3.22E-06 | 0.0003278 | no |
| Sobic.009G085001 | 92.6649 | -2.8512 | 0.6128 | -4.6528 | 3.28E-06 | 0.0003321 | down |
| Sobic.007G221400 | 54.6724 | -3.0843 | 0.6631 | -4.6512 | 3.30E-06 | 0.000333 | down |
| Sobic.008G191300 | 1949.7632 | 3.0945 | 0.6660 | 4.6466 | 3.37E-06 | 0.0003389 | up |
| Sobic.006G193700 | 40.7673 | 6.7306 | 1.4499 | 4.6423 | 3.45E-06 | 0.0003444 | up |
| Sobic.002G428400 | 113.4187 | 3.1543 | 0.6804 | 4.6358 | 3.56E-06 | 0.000353 | up |
| Sobic.005G155200 | 18.1861 | 7.3989 | 1.5962 | 4.6352 | 3.57E-06 | 0.000353 | up |
| Sobic.002G126400 | 53.2522 | -3.0058 | 0.6504 | -4.6215 | 3.81E-06 | 0.0003754 | down |
| Sobic.002G366600 | 156.7380 | -2.4425 | 0.5291 | -4.6161 | 3.91E-06 | 0.0003767 | down |
| Sobic.005G111200 | 16537.4430 | -1.5903 | 0.3443 | -4.6191 | 3.85E-06 | 0.0003767 | no |
| Sobic.002G187700 | 1672.1954 | -1.1516 | 0.2494 | -4.6170 | 3.89E-06 | 0.0003767 | no |
| Sobic.001G447100 | 318.5035 | 1.4474 | 0.3136 | 4.6160 | 3.91E-06 | 0.0003767 | no |
| Sobic.009G173600 | 136.4966 | 4.9678 | 1.0760 | 4.6169 | 3.89E-06 | 0.0003767 | up |
| Sobic.009G071800 | 1481.7461 | 1.5728 | 0.3419 | 4.5999 | 4.23E-06 | 0.0004052 | no |
| Sobic.005G231600 | 158.1965 | 3.5571 | 0.7745 | 4.5925 | 4.38E-06 | 0.000418 | up |
| Sobic.007G055700 | 59.8082 | -3.4720 | 0.7565 | -4.5896 | 4.44E-06 | 0.0004218 | down |
| Sobic.003G310200 | 897.1196 | -2.0733 | 0.4521 | -4.5859 | 4.52E-06 | 0.0004274 | down |

| Sobic.008G168700 | 60.6087 | -2.1989 | 0.4807 | -4.5741 | 4.78E-06 | 0.0004504 | down |
| --- | --- | --- | --- | --- | --- | --- | --- |
| Sobic.006G026000 | 2502.7054 | -2.8310 | 0.6191 | -4.5727 | 4.81E-06 | 0.0004512 | down |
| Sobic.002G210100 | 62.1433 | 2.5277 | 0.5537 | 4.5653 | 4.99E-06 | 0.0004655 | up |
| Sobic.003G216232 | 228.6507 | 2.8546 | 0.6273 | 4.5509 | 5.34E-06 | 0.0004963 | up |
| Sobic.006G151001 | 113.8342 | 1.7921 | 0.3940 | 4.5485 | 5.40E-06 | 0.0004998 | no |
| Sobic.002G284600 | 84.6567 | -4.0874 | 0.8995 | -4.5442 | 5.51E-06 | 0.0005055 | down |
| Sobic.009G004500 | 5029.3777 | -1.9331 | 0.4254 | -4.5445 | 5.51E-06 | 0.0005055 | no |
| Sobic.008G080400 | 10.1345 | -7.0317 | 1.5514 | -4.5325 | 5.83E-06 | 0.0005298 | down |
| Sobic.004G284700 | 174.6648 | -3.8538 | 0.8502 | -4.5326 | 5.83E-06 | 0.0005298 | down |
| Sobic.001G198500 | 110.5175 | -1.5975 | 0.3529 | -4.5267 | 5.99E-06 | 0.0005399 | no |
| Sobic.001G308500 | 85.2360 | 1.8384 | 0.4061 | 4.5275 | 5.97E-06 | 0.0005399 | no |
| Sobic.006G115300 | 4790.4116 | 2.0614 | 0.4563 | 4.5172 | 6.27E-06 | 0.0005624 | up |
| Sobic.007G224400 | 186.6514 | -3.4108 | 0.7554 | -4.5152 | 6.33E-06 | 0.0005653 | down |
| Sobic.003G022700 | 543.7906 | 1.8223 | 0.4037 | 4.5134 | 6.38E-06 | 0.0005676 | no |
| Sobic.006G026700 | 338.0718 | -2.3556 | 0.5222 | -4.5112 | 6.45E-06 | 0.0005713 | down |
| Sobic.001G314300 | 3177.3376 | 1.9630 | 0.4358 | 4.5050 | 6.64E-06 | 0.0005857 | no |
| Sobic.009G167900 | 220.2019 | 1.8558 | 0.4121 | 4.5038 | 6.68E-06 | 0.0005866 | no |
| Sobic.002G414400 | 36721.8041 | -2.2431 | 0.4982 | -4.5024 | 6.72E-06 | 0.0005879 | down |
| Sobic.008G094300 | 2180.7103 | -2.2809 | 0.5074 | -4.4957 | 6.93E-06 | 0.0006042 | down |
| Sobic.001G115300 | 323.9294 | 3.1900 | 0.7099 | 4.4934 | 7.01E-06 | 0.0006084 | up |
| Sobic.001G148500 | 1298.0150 | 1.4737 | 0.3286 | 4.4846 | 7.30E-06 | 0.0006313 | no |
| Sobic.003G439000 | 6828.3688 | -1.8148 | 0.4050 | -4.4812 | 7.42E-06 | 0.0006389 | no |
| Sobic.010G096300 | 8629.9377 | -1.0255 | 0.2291 | -4.4767 | 7.58E-06 | 0.0006499 | no |
| Sobic.001G168800 | 1996.2054 | -1.3558 | 0.3029 | -4.4754 | 7.63E-06 | 0.0006511 | no |
| Sobic.002G086800 | 1014.4000 | -1.1357 | 0.2542 | -4.4677 | 7.91E-06 | 0.0006724 | no |
| Sobic.010G091400 | 785.7508 | 1.7746 | 0.3973 | 4.4665 | 7.95E-06 | 0.0006734 | no |
| Sobic.010G171000 | 671.9922 | 1.3805 | 0.3093 | 4.4639 | 8.05E-06 | 0.0006789 | no |
| Sobic.002G314200 | 4978.1591 | -1.2040 | 0.2702 | -4.4561 | 8.35E-06 | 0.0007012 | no |
| Sobic.003G313000 | 68303.7739 | -2.3200 | 0.5210 | -4.4529 | 8.47E-06 | 0.000709 | down |
| Sobic.001G455000 | 2046.0760 | 0.9593 | 0.2158 | 4.4459 | 8.75E-06 | 0.0007297 | no |
| Sobic.006G169100 | 18.5669 | -6.9258 | 1.5585 | -4.4438 | 8.84E-06 | 0.0007338 | down |
| Sobic.001G363100 | 1971.6793 | -2.7296 | 0.6158 | -4.4329 | 9.30E-06 | 0.0007689 | down |
| Sobic.003G443400 | 166.5434 | 1.9950 | 0.4502 | 4.4310 | 9.38E-06 | 0.0007727 | no |
| Sobic.003G107800 | 640.4117 | -2.0969 | 0.4735 | -4.4282 | 9.50E-06 | 0.0007799 | down |
| Sobic.009G049000 | 63.8059 | -3.1093 | 0.7027 | -4.4247 | 9.66E-06 | 0.0007893 | down |
| Sobic.004G253500 | 10.6884 | 6.6315 | 1.4993 | 4.4230 | 9.73E-06 | 0.0007926 | up |
| Sobic.004G338200 | 53.4008 | 3.2464 | 0.7346 | 4.4191 | 9.91E-06 | 0.0008041 | up |
| Sobic.004G076100 | 424.8011 | 1.6974 | 0.3844 | 4.4155 | 1.01E-05 | 0.0008143 | no |
| Sobic.006G188000 | 1170.6494 | -1.4676 | 0.3326 | -4.4129 | 1.02E-05 | 0.0008211 | no |
| Sobic.001G235500 | 1442.9133 | 2.0022 | 0.4539 | 4.4112 | 1.03E-05 | 0.0008211 | up |
| Sobic.001G050100 | 289.4355 | 2.0534 | 0.4654 | 4.4120 | 1.02E-05 | 0.0008211 | up |
| Sobic.001G147400 | 793.1933 | -1.5304 | 0.3473 | -4.4064 | 1.05E-05 | 0.0008281 | no |
| Sobic.003G196900 | 1440.7636 | 1.0321 | 0.2341 | 4.4083 | 1.04E-05 | 0.0008281 | no |
| Sobic.001G343000 | 656.9738 | 1.4494 | 0.3289 | 4.4066 | 1.05E-05 | 0.0008281 | no |
| Sobic.006G119600 | 39.8031 | 2.4319 | 0.5519 | 4.4061 | 1.05E-05 | 0.0008281 | up |
| Sobic.001G408100 | 1931.6957 | 1.0585 | 0.2404 | 4.4033 | 1.07E-05 | 0.0008357 | no |
| Sobic.004G251500 | 2084.6702 | -3.1338 | 0.7122 | -4.4001 | 1.08E-05 | 0.000845 | down |
| Sobic.004G299850 | 323.0908 | 2.3236 | 0.5293 | 4.3897 | 1.14E-05 | 0.0008834 | up |
| Sobic.K009800 | 109.5107 | -3.1352 | 0.7178 | -4.3675 | 1.26E-05 | 0.0009745 | down |
| Sobic.008G088860 | 219.2965 | -2.7201 | 0.6235 | -4.3625 | 1.29E-05 | 0.0009895 | down |
| Sobic.008G191200 | 2192.5416 | 2.8444 | 0.6519 | 4.3631 | 1.28E-05 | 0.0009895 | up |
| Sobic.002G196100 | 134.6350 | 3.8753 | 0.8894 | 4.3573 | 1.32E-05 | 0.0010098 | up |
| Sobic.003G347000 | 28.4605 | 3.9233 | 0.9019 | 4.3503 | 1.36E-05 | 0.0010389 | up |
| Sobic.003G290900 | 1084.9609 | 1.3531 | 0.3111 | 4.3493 | 1.37E-05 | 0.0010396 | no |
| Sobic.003G155300 | 2756.5040 | 1.3400 | 0.3086 | 4.3417 | 1.41E-05 | 0.0010727 | no |
| Sobic.001G089000 | 300.7643 | 3.0716 | 0.7077 | 4.3404 | 1.42E-05 | 0.001075 | up |
| Sobic.005G075200 | 159.1826 | 1.4701 | 0.3395 | 4.3299 | 1.49E-05 | 0.0011237 | no |

| Sobic.007G066050 | 87.6300 | -2.3514 | 0.5437 | -4.3250 | 1.53E-05 | 0.0011447 | down |
| --- | --- | --- | --- | --- | --- | --- | --- |
| Sobic.003G417350 | 314.5977 | 1.5976 | 0.3702 | 4.3155 | 1.59E-05 | 0.0011909 | no |
| Sobic.007G210500 | 8555.0873 | 2.4355 | 0.5650 | 4.3108 | 1.63E-05 | 0.001212 | up |
| Sobic.001G483300 | 2552.3681 | -1.3913 | 0.3231 | -4.3061 | 1.66E-05 | 0.0012338 | no |
| Sobic.007G139200 | 2580.0575 | -1.5062 | 0.3499 | -4.3046 | 1.67E-05 | 0.001238 | no |
| Sobic.002G331450 | 15.6996 | 6.2090 | 1.4453 | 4.2959 | 1.74E-05 | 0.001283 | up |
| Sobic.007G004800 | 90690.5745 | -1.9410 | 0.4538 | -4.2771 | 1.89E-05 | 0.0013914 | no |
| Sobic.003G074700 | 2912.0223 | -2.2893 | 0.5358 | -4.2729 | 1.93E-05 | 0.0014128 | down |
| Sobic.002G264800 | 167.2318 | -3.9800 | 0.9328 | -4.2668 | 1.98E-05 | 0.0014451 | down |
| Sobic.001G288000 | 62.4074 | 2.4357 | 0.5709 | 4.2663 | 1.99E-05 | 0.0014451 | up |
| Sobic.006G084201 | 287.9015 | -1.7217 | 0.4039 | -4.2628 | 2.02E-05 | 0.0014583 | no |
| Sobic.003G103701 | 418.9156 | 3.4609 | 0.8118 | 4.2635 | 2.01E-05 | 0.0014583 | up |
| Sobic.003G055300 | 31563.2814 | -2.0690 | 0.4863 | -4.2543 | 2.10E-05 | 0.0015022 | down |
| Sobic.006G182700 | 846.0969 | -1.7278 | 0.4062 | -4.2538 | 2.10E-05 | 0.0015022 | no |
| Sobic.004G304700 | 94.5504 | 1.6865 | 0.3964 | 4.2544 | 2.10E-05 | 0.0015022 | no |
| Sobic.001G317600 | 309.1466 | 3.5800 | 0.8425 | 4.2494 | 2.14E-05 | 0.0015274 | up |
| Sobic.002G088200 | 3248.8062 | -1.8014 | 0.4245 | -4.2433 | 2.20E-05 | 0.0015587 | no |
| Sobic.005G124100 | 104.7320 | 1.8356 | 0.4325 | 4.2437 | 2.20E-05 | 0.0015587 | no |
| Sobic.008G131700 | 1341.9727 | -1.9113 | 0.4513 | -4.2353 | 2.28E-05 | 0.0016102 | no |
| Sobic.001G271500 | 20.0796 | 4.2390 | 1.0019 | 4.2309 | 2.33E-05 | 0.001636 | up |
| Sobic.002G031600 | 706.0205 | -1.6827 | 0.3979 | -4.2290 | 2.35E-05 | 0.0016388 | no |
| Sobic.004G031100 | 2463.4231 | -1.2097 | 0.2860 | -4.2296 | 2.34E-05 | 0.0016388 | no |
| Sobic.002G351300 | 340.5283 | 1.5031 | 0.3557 | 4.2251 | 2.39E-05 | 0.0016621 | no |
| Sobic.005G226600 | 13.7786 | 6.9980 | 1.6596 | 4.2168 | 2.48E-05 | 0.0017192 | up |
| Sobic.003G391100 | 1767.2856 | -1.4326 | 0.3399 | -4.2146 | 2.50E-05 | 0.0017246 | no |
| Sobic.007G080600 | 174.6607 | 1.6422 | 0.3896 | 4.2151 | 2.50E-05 | 0.0017246 | no |
| Sobic.010G066000 | 32.0454 | 3.1731 | 0.7536 | 4.2107 | 2.55E-05 | 0.0017489 | up |
| Sobic.009G236800 | 2674.3325 | -1.8200 | 0.4335 | -4.1986 | 2.69E-05 | 0.0018271 | no |
| Sobic.001G536800 | 873.0247 | -1.7227 | 0.4103 | -4.1987 | 2.68E-05 | 0.0018271 | no |
| Sobic.001G302800 | 3855.1631 | -1.6106 | 0.3836 | -4.1991 | 2.68E-05 | 0.0018271 | no |
| Sobic.004G210500 | 532.3071 | 1.9499 | 0.4647 | 4.1962 | 2.71E-05 | 0.0018408 | no |
| Sobic.003G068800 | 1871.1149 | -1.8724 | 0.4467 | -4.1919 | 2.77E-05 | 0.0018583 | no |
| Sobic.001G520700 | 1006.8949 | -0.9726 | 0.2320 | -4.1929 | 2.75E-05 | 0.0018583 | no |
| Sobic.001G119100 | 135.0375 | 3.0450 | 0.7264 | 4.1919 | 2.77E-05 | 0.0018583 | up |
| Sobic.010G260700 | 2945.2074 | -1.4336 | 0.3422 | -4.1888 | 2.80E-05 | 0.0018778 | no |
| Sobic.004G343100 | 2169.6682 | -1.3943 | 0.3331 | -4.1864 | 2.83E-05 | 0.0018912 | no |
| Sobic.001G403300 | 920.8464 | -2.1591 | 0.5158 | -4.1856 | 2.84E-05 | 0.0018919 | down |
| Sobic.001G381300 | 201.6087 | 2.3330 | 0.5575 | 4.1848 | 2.85E-05 | 0.0018926 | up |
| Sobic.009G044501 | 661.2473 | -2.5758 | 0.6160 | -4.1818 | 2.89E-05 | 0.0019119 | down |
| Sobic.008G019100 | 353.2212 | -2.1343 | 0.5107 | -4.1789 | 2.93E-05 | 0.0019308 | down |
| Sobic.001G305900 | 197.1590 | 1.2987 | 0.3110 | 4.1762 | 2.96E-05 | 0.0019443 | no |
| Sobic.005G186500 | 334.2065 | 4.4792 | 1.0726 | 4.1759 | 2.97E-05 | 0.0019443 | up |
| Sobic.008G024550 | 259.7696 | -1.8990 | 0.4562 | -4.1625 | 3.15E-05 | 0.002055 | no |
| Sobic.006G258800 | 909.7482 | 1.8472 | 0.4440 | 4.1601 | 3.18E-05 | 0.0020708 | no |
| Sobic.001G166800 | 45.3209 | -3.0575 | 0.7361 | -4.1540 | 3.27E-05 | 0.0021202 | down |
| Sobic.001G433200 | 960.9620 | 1.3898 | 0.3348 | 4.1513 | 3.31E-05 | 0.0021389 | no |
| Sobic.001G429200 | 171.8095 | 2.0350 | 0.4905 | 4.1490 | 3.34E-05 | 0.0021536 | up |
| Sobic.010G107000 | 1215.6324 | 1.0514 | 0.2538 | 4.1420 | 3.44E-05 | 0.0022138 | no |
| Sobic.001G393600 | 143.1595 | 2.5077 | 0.6073 | 4.1293 | 3.64E-05 | 0.002332 | up |
| Sobic.002G310900 | 2295.7671 | -1.5660 | 0.3797 | -4.1239 | 3.72E-05 | 0.002366 | no |
| Sobic.006G151100 | 930.3802 | -1.0453 | 0.2534 | -4.1245 | 3.72E-05 | 0.002366 | no |
| Sobic.008G094800 | 116.1206 | 1.6853 | 0.4086 | 4.1245 | 3.72E-05 | 0.002366 | no |
| Sobic.005G124850 | 52.5073 | -2.7857 | 0.6758 | -4.1221 | 3.76E-05 | 0.0023781 | down |
| Sobic.005G042100 | 118.0833 | -2.7195 | 0.6609 | -4.1151 | 3.87E-05 | 0.0024437 | down |
| Sobic.009G244900 | 75.6827 | 3.6504 | 0.8873 | 4.1139 | 3.89E-05 | 0.0024491 | up |
| Sobic.003G374600 | 373.7442 | -1.2435 | 0.3026 | -4.1099 | 3.96E-05 | 0.0024844 | no |
| Sobic.007G180300 | 524.1045 | 1.4745 | 0.3591 | 4.1057 | 4.03E-05 | 0.002523 | no |

| Sobic.001G369401 | 16.9364 | -3.4802 | 0.8481 | -4.1036 | 4.07E-05 | 0.002525 | down |
| --- | --- | --- | --- | --- | --- | --- | --- |
| Sobic.008G079400 | 1528.7295 | -1.9936 | 0.4862 | -4.1007 | 4.12E-05 | 0.002525 | no |
| Sobic.008G157500 | 16270.0759 | -1.5234 | 0.3713 | -4.1031 | 4.08E-05 | 0.002525 | no |
| Sobic.005G224800 | 1161.9643 | -1.3872 | 0.3383 | -4.1000 | 4.13E-05 | 0.002525 | no |
| Sobic.001G319100 | 198.0343 | 1.5493 | 0.3779 | 4.1003 | 4.13E-05 | 0.002525 | no |
| Sobic.002G068800 | 108.7382 | 1.6084 | 0.3922 | 4.1010 | 4.11E-05 | 0.002525 | no |
| Sobic.007G064100 | 52.2605 | 2.2582 | 0.5505 | 4.1019 | 4.10E-05 | 0.002525 | up |
| Sobic.010G088800 | 66.0762 | 4.0392 | 0.9844 | 4.1034 | 4.07E-05 | 0.002525 | up |
| Sobic.003G143700 | 3799.7911 | 1.5898 | 0.3881 | 4.0962 | 4.20E-05 | 0.0025599 | no |
| Sobic.005G037300 | 691.8081 | -6.4462 | 1.5761 | -4.0901 | 4.31E-05 | 0.0026206 | down |
| Sobic.002G049600 | 3244.8100 | -1.5228 | 0.3724 | -4.0888 | 4.34E-05 | 0.0026275 | no |
| Sobic.005G118300 | 91.7488 | -3.2506 | 0.7985 | -4.0709 | 4.68E-05 | 0.0028299 | down |
| Sobic.005G115600 | 9527.4951 | 1.5761 | 0.3875 | 4.0673 | 4.76E-05 | 0.0028657 | no |
| Sobic.002G156200 | 987.7081 | -1.0936 | 0.2690 | -4.0652 | 4.80E-05 | 0.0028839 | no |
| Sobic.005G004800 | 11.3783 | -4.6962 | 1.1570 | -4.0589 | 4.93E-05 | 0.0029538 | down |
| Sobic.007G186000 | 3132.4564 | 1.1854 | 0.2922 | 4.0562 | 4.99E-05 | 0.0029796 | no |
| Sobic.004G004500 | 55.2883 | 4.3758 | 1.0799 | 4.0519 | 5.08E-05 | 0.003027 | up |
| Sobic.004G058000 | 397.7427 | 1.1643 | 0.2877 | 4.0474 | 5.18E-05 | 0.003076 | no |
| Sobic.005G210000 | 1585.9241 | 1.3538 | 0.3350 | 4.0415 | 5.31E-05 | 0.0031467 | no |
| Sobic.007G059200 | 955.2119 | 1.8601 | 0.4605 | 4.0391 | 5.37E-05 | 0.0031701 | no |
| Sobic.001G092600 | 219.7782 | 2.0063 | 0.4974 | 4.0339 | 5.49E-05 | 0.0032317 | up |
| Sobic.004G244600 | 53.7060 | 2.3736 | 0.5892 | 4.0286 | 5.61E-05 | 0.0032962 | up |
| Sobic.009G248000 | 282.4548 | -1.6103 | 0.3998 | -4.0275 | 5.64E-05 | 0.0033019 | no |
| Sobic.K010100 | 62.7795 | -3.9276 | 0.9759 | -4.0247 | 5.71E-05 | 0.003333 | down |
| Sobic.009G170700 | 20.7358 | -3.2580 | 0.8100 | -4.0221 | 5.77E-05 | 0.0033429 | down |
| Sobic.009G080400 | 524.4578 | 1.8957 | 0.4713 | 4.0220 | 5.77E-05 | 0.0033429 | no |
| Sobic.008G126800 | 114.1222 | 2.4646 | 0.6126 | 4.0232 | 5.74E-05 | 0.0033429 | up |
| Sobic.007G179750 | 3284.7991 | -1.0344 | 0.2574 | -4.0183 | 5.86E-05 | 0.0033773 | no |
| Sobic.009G116200 | 45.1939 | 3.1132 | 0.7746 | 4.0188 | 5.85E-05 | 0.0033773 | up |
| Sobic.006G104900 | 414.9005 | 1.2791 | 0.3184 | 4.0170 | 5.90E-05 | 0.0033877 | no |
| Sobic.002G041100 | 2260.3513 | -1.4979 | 0.3730 | -4.0158 | 5.92E-05 | 0.0033952 | no |
| Sobic.005G210100 | 48.4421 | -3.0773 | 0.7670 | -4.0120 | 6.02E-05 | 0.0034403 | down |
| Sobic.005G161100 | 40.9255 | -7.1410 | 1.7803 | -4.0112 | 6.04E-05 | 0.0034407 | down |
| Sobic.010G109500 | 20348.7093 | -2.2492 | 0.5608 | -4.0107 | 6.05E-05 | 0.0034407 | down |
| Sobic.008G139000 | 126.1650 | -1.9874 | 0.4964 | -4.0039 | 6.23E-05 | 0.0035139 | no |
| Sobic.002G320900 | 523.1891 | 1.6663 | 0.4161 | 4.0047 | 6.21E-05 | 0.0035139 | no |
| Sobic.003G129400 | 63.6616 | 2.1852 | 0.5457 | 4.0045 | 6.22E-05 | 0.0035139 | up |
| Sobic.003G312400 | 324.7921 | 1.6894 | 0.4222 | 4.0010 | 6.31E-05 | 0.0035476 | no |
| Sobic.001G448300 | 1560.0864 | -1.8072 | 0.4529 | -3.9900 | 6.61E-05 | 0.0037051 | no |
| Sobic.004G201600 | 67.6553 | 2.0505 | 0.5147 | 3.9839 | 6.78E-05 | 0.0037916 | up |
| Sobic.009G254600 | 205.1630 | -2.5954 | 0.6518 | -3.9818 | 6.84E-05 | 0.0038031 | down |
| Sobic.001G255700 | 339.0705 | -2.0494 | 0.5148 | -3.9813 | 6.85E-05 | 0.0038031 | down |
| Sobic.001G133000 | 2962.8149 | -1.6256 | 0.4082 | -3.9822 | 6.83E-05 | 0.0038031 | no |
| Sobic.010G069900 | 30.1381 | 3.1044 | 0.7800 | 3.9801 | 6.89E-05 | 0.0038133 | up |
| Sobic.001G464900 | 436.5057 | 1.5252 | 0.3835 | 3.9773 | 6.97E-05 | 0.0038479 | no |
| Sobic.003G150700 | 569.5250 | -2.0221 | 0.5098 | -3.9666 | 7.29E-05 | 0.0040136 | down |
| Sobic.010G078100 | 668.2314 | 1.3315 | 0.3358 | 3.9659 | 7.31E-05 | 0.0040162 | no |
| Sobic.001G348100 | 433.7287 | 2.2014 | 0.5553 | 3.9647 | 7.35E-05 | 0.0040251 | up |
| Sobic.004G358400 | 2605.1406 | 1.3921 | 0.3515 | 3.9604 | 7.48E-05 | 0.0040884 | no |
| Sobic.009G179800 | 2627.7067 | -2.1278 | 0.5378 | -3.9563 | 7.61E-05 | 0.0041486 | down |
| Sobic.006G191400 | 100.4370 | -1.9556 | 0.4951 | -3.9503 | 7.81E-05 | 0.0042402 | no |
| Sobic.K028200 | 148.6734 | -1.6740 | 0.4238 | -3.9498 | 7.82E-05 | 0.0042402 | no |
| Sobic.004G144500 | 964.5573 | 1.0080 | 0.2555 | 3.9443 | 8.00E-05 | 0.0043269 | no |
| Sobic.003G381700 | 6040.9144 | 1.4684 | 0.3724 | 3.9430 | 8.05E-05 | 0.0043393 | no |
| Sobic.002G326650 | 63.6280 | 3.1979 | 0.8114 | 3.9413 | 8.10E-05 | 0.0043602 | up |
| Sobic.006G169900 | 14.1671 | 7.0375 | 1.7859 | 3.9406 | 8.13E-05 | 0.0043615 | up |
| Sobic.010G083550 | 11.7868 | 6.7717 | 1.7188 | 3.9398 | 8.15E-05 | 0.0043648 | up |

| Sobic.007G001900 | 136.7015 | -1.9410 | 0.4928 | -3.9386 | 8.20E-05 | 0.0043755 | no |
| --- | --- | --- | --- | --- | --- | --- | --- |
| Sobic.008G124600 | 358.0725 | 1.2744 | 0.3237 | 3.9370 | 8.25E-05 | 0.0043834 | no |
| Sobic.004G236000 | 195.2739 | 4.0527 | 1.0294 | 3.9370 | 8.25E-05 | 0.0043834 | up |
| Sobic.001G486800 | 8.5308 | -6.7807 | 1.7242 | -3.9327 | 8.40E-05 | 0.0044499 | down |
| Sobic.001G295600 | 8721.2084 | -1.7420 | 0.4430 | -3.9319 | 8.43E-05 | 0.0044537 | no |
| Sobic.002G104000 | 1041.2551 | -1.4725 | 0.3747 | -3.9301 | 8.49E-05 | 0.0044537 | no |
| Sobic.009G126000 | 602.6361 | 0.9086 | 0.2312 | 3.9304 | 8.48E-05 | 0.0044537 | no |
| Sobic.002G341200 | 585.2683 | 1.7112 | 0.4354 | 3.9303 | 8.49E-05 | 0.0044537 | no |
| Sobic.003G231700 | 12.2070 | -7.2939 | 1.8566 | -3.9287 | 8.54E-05 | 0.0044687 | down |
| Sobic.002G195200 | 10.9041 | 6.6614 | 1.6962 | 3.9272 | 8.60E-05 | 0.0044869 | up |
| Sobic.002G140300 | 1449.4889 | 1.9208 | 0.4903 | 3.9172 | 8.96E-05 | 0.0046646 | no |
| Sobic.001G459800 | 776.8050 | 1.5521 | 0.3965 | 3.9145 | 9.06E-05 | 0.0047061 | no |
| Sobic.009G142200 | 425.3672 | 2.2790 | 0.5823 | 3.9137 | 9.09E-05 | 0.0047091 | up |
| Sobic.003G052700 | 1567.0184 | -2.1629 | 0.5529 | -3.9116 | 9.17E-05 | 0.0047397 | down |
| Sobic.006G188600 | 259.6475 | 1.8719 | 0.4786 | 3.9109 | 9.20E-05 | 0.0047412 | no |
| Sobic.006G135100 | 32343.5693 | -1.1977 | 0.3070 | -3.9006 | 9.59E-05 | 0.0049299 | no |
| Sobic.004G324950 | 24.3240 | 4.2542 | 1.0907 | 3.9003 | 9.61E-05 | 0.0049299 | up |
| Sobic.003G245600 | 632.2944 | -1.2412 | 0.3185 | -3.8976 | 9.72E-05 | 0.004973 | no |
| Sobic.001G203000 | 3305.0153 | -2.0564 | 0.5278 | -3.8964 | 9.77E-05 | 0.0049865 | down |
| Sobic.009G055550 | 199.1200 | 1.9274 | 0.4948 | 3.8954 | 9.80E-05 | 0.0049935 | no |
| Sobic.003G392700 | 7883.8557 | -1.7953 | 0.4611 | -3.8939 | 9.87E-05 | 0.0050016 | no |
| Sobic.003G445900 | 390.4077 | 2.2837 | 0.5864 | 3.8942 | 9.85E-05 | 0.0050016 | up |
| Sobic.006G116000 | 157.6364 | -1.7749 | 0.4560 | -3.8923 | 9.93E-05 | 0.0050223 | no |
| Sobic.003G432700 | 128.1484 | -2.2122 | 0.5688 | -3.8894 | 0.0001005 | 0.0050709 | down |
| Sobic.006G088000 | 5222.6842 | 0.9051 | 0.2328 | 3.8878 | 0.0001012 | 0.0050918 | no |
| Sobic.001G195800 | 4035.0524 | -1.6978 | 0.4371 | -3.8841 | 0.0001027 | 0.005146 | no |
| Sobic.001G449600 | 93.0570 | 2.4022 | 0.6184 | 3.8845 | 0.0001025 | 0.005146 | up |
| Sobic.010G126600 | 78.4692 | -2.1961 | 0.5657 | -3.8824 | 0.0001034 | 0.0051684 | down |
| Sobic.001G469933 | 2313.9465 | -1.6306 | 0.4201 | -3.8810 | 0.000104 | 0.0051859 | no |
| Sobic.004G276200 | 4400.4746 | -1.7706 | 0.4567 | -3.8767 | 0.0001059 | 0.0052672 | no |
| Sobic.009G205300 | 3340.6385 | 2.6731 | 0.6906 | 3.8705 | 0.0001086 | 0.0053893 | up |
| Sobic.005G188900 | 213.1434 | -3.0907 | 0.7992 | -3.8675 | 0.00011 | 0.0054315 | down |
| Sobic.001G162700 | 86.8590 | 3.3557 | 0.8676 | 3.8678 | 0.0001098 | 0.0054315 | up |
| Sobic.007G129201 | 441.3442 | -1.5128 | 0.3914 | -3.8649 | 0.0001111 | 0.0054762 | no |
| Sobic.009G143500 | 1660.4480 | -1.4771 | 0.3823 | -3.8639 | 0.0001116 | 0.005485 | no |
| Sobic.004G314700 | 424.2546 | 1.3620 | 0.3527 | 3.8619 | 0.0001125 | 0.0055054 | no |
| Sobic.003G288600 | 4776.0865 | 1.8391 | 0.4763 | 3.8613 | 0.0001128 | 0.0055054 | no |
| Sobic.005G205100 | 83.9760 | 3.2291 | 0.8361 | 3.8621 | 0.0001124 | 0.0055054 | up |
| Sobic.008G033200 | ######### | -1.1852 | 0.3071 | -3.8597 | 0.0001135 | 0.0055291 | no |
| Sobic.003G209800 | 41430.3153 | -3.7366 | 0.9694 | -3.8546 | 0.0001159 | 0.0056063 | down |
| Sobic.010G120000 | 487.2347 | -1.1076 | 0.2873 | -3.8556 | 0.0001154 | 0.0056063 | no |
| Sobic.009G020950 | 390.1039 | 1.9424 | 0.5039 | 3.8547 | 0.0001159 | 0.0056063 | no |
| Sobic.003G354500 | 70.3078 | 1.9024 | 0.4937 | 3.8530 | 0.0001167 | 0.00563 | no |
| Sobic.007G014600 | 2252.5156 | -3.1518 | 0.8183 | -3.8517 | 0.0001173 | 0.0056467 | down |
| Sobic.004G068300 | 11656.0307 | -1.8920 | 0.4913 | -3.8511 | 0.0001176 | 0.0056485 | no |
| Sobic.004G166400 | 2168.5248 | -1.9141 | 0.4972 | -3.8496 | 0.0001183 | 0.005671 | no |
| Sobic.003G173116 | 149.2102 | -1.5941 | 0.4143 | -3.8482 | 0.000119 | 0.0056892 | no |
| Sobic.002G152700 | 1488.6327 | -2.0631 | 0.5366 | -3.8451 | 0.0001205 | 0.0057498 | down |
| Sobic.001G123100 | 383.3023 | 1.5272 | 0.3974 | 3.8426 | 0.0001217 | 0.0057953 | no |
| Sobic.005G041200 | 70.2940 | -3.2369 | 0.8427 | -3.8411 | 0.0001225 | 0.0058169 | down |
| Sobic.001G521200 | 641.8249 | -1.4277 | 0.3723 | -3.8344 | 0.0001259 | 0.0059657 | no |
| Sobic.001G344500 | 1317.2062 | -2.9918 | 0.7816 | -3.8280 | 0.0001292 | 0.0061096 | down |
| Sobic.006G088400 | 6.8836 | -6.4769 | 1.6974 | -3.8158 | 0.0001357 | 0.0064031 | down |
| Sobic.003G239200 | 42.4430 | -3.2571 | 0.8538 | -3.8147 | 0.0001363 | 0.0064041 | down |
| Sobic.006G257300 | 209.9271 | 1.2620 | 0.3308 | 3.8149 | 0.0001362 | 0.0064041 | no |
| Sobic.001G318000 | 248.2783 | 3.6255 | 0.9524 | 3.8065 | 0.0001409 | 0.0066048 | up |
| Sobic.006G225200 | 3488.9656 | -1.7796 | 0.4677 | -3.8047 | 0.000142 | 0.0066381 | no |

| Sobic.003G057900 | 17.3642 | -6.8304 | 1.7969 | -3.8013 | 0.0001439 | 0.0067157 | down |
| --- | --- | --- | --- | --- | --- | --- | --- |
| Sobic.007G184300 | 287.1897 | 1.0498 | 0.2767 | 3.7941 | 0.0001482 | 0.0068823 | no |
| Sobic.007G028001 | 72.5986 | 1.5138 | 0.3989 | 3.7945 | 0.0001479 | 0.0068823 | no |
| Sobic.010G251300 | 6148.8097 | 1.7076 | 0.4505 | 3.7908 | 0.0001501 | 0.0069592 | no |
| Sobic.003G221200 | 1446.0631 | -1.9308 | 0.5094 | -3.7900 | 0.0001506 | 0.0069667 | no |
| Sobic.007G168000 | 71.3163 | -5.3323 | 1.4075 | -3.7885 | 0.0001515 | 0.0069818 | down |
| Sobic.004G265000 | 756.1787 | 4.2590 | 1.1242 | 3.7884 | 0.0001516 | 0.0069818 | up |
| Sobic.006G073700 | 32.9020 | -2.0459 | 0.5405 | -3.7855 | 0.0001534 | 0.0070337 | down |
| Sobic.007G107600 | 38.9034 | 2.8996 | 0.7660 | 3.7856 | 0.0001533 | 0.0070337 | up |
| Sobic.008G068400 | 316.2096 | -1.7022 | 0.4498 | -3.7842 | 0.0001542 | 0.0070534 | no |
| Sobic.001G296300 | 2156.0957 | -2.9430 | 0.7789 | -3.7785 | 0.0001577 | 0.0071873 | down |
| Sobic.003G031800 | 3520.0773 | 0.9817 | 0.2598 | 3.7785 | 0.0001578 | 0.0071873 | no |
| Sobic.010G240600 | 8.4879 | -5.7844 | 1.5330 | -3.7732 | 0.0001612 | 0.0073086 | down |
| Sobic.002G079300 | 156.0298 | -1.7911 | 0.4747 | -3.7734 | 0.0001611 | 0.0073086 | no |
| Sobic.001G211800 | 150.2914 | -2.2424 | 0.5946 | -3.7711 | 0.0001625 | 0.0073232 | down |
| Sobic.010G255500 | 173.2556 | 2.4499 | 0.6496 | 3.7712 | 0.0001624 | 0.0073232 | up |
| Sobic.001G284425 | 31.4324 | 5.0681 | 1.3438 | 3.7715 | 0.0001623 | 0.0073232 | up |
| Sobic.001G072300 | 466.0263 | -1.5345 | 0.4071 | -3.7698 | 0.0001634 | 0.0073478 | no |
| Sobic.005G124200 | 51.0032 | 2.6404 | 0.7008 | 3.7676 | 0.0001648 | 0.0073943 | up |
| Sobic.006G100332 | 90.6286 | 2.5862 | 0.6888 | 3.7548 | 0.0001735 | 0.0077677 | up |
| Sobic.006G076201 | 118.2520 | 3.6556 | 0.9739 | 3.7536 | 0.0001743 | 0.0077875 | up |
| Sobic.001G063600 | 229.1139 | 1.5533 | 0.4141 | 3.7512 | 0.000176 | 0.0078472 | no |
| Sobic.002G036100 | 27.8357 | 2.8235 | 0.7532 | 3.7487 | 0.0001778 | 0.0079098 | up |
| Sobic.003G215800 | 170.9817 | 1.8571 | 0.4955 | 3.7479 | 0.0001783 | 0.0079163 | no |
| Sobic.001G374600 | 14.9771 | 4.2713 | 1.1405 | 3.7453 | 0.0001802 | 0.0079841 | up |
| Sobic.009G002500 | 404.8549 | -2.6491 | 0.7079 | -3.7420 | 0.0001826 | 0.0080717 | down |
| Sobic.004G151500 | 3873.3446 | -0.8214 | 0.2196 | -3.7411 | 0.0001832 | 0.0080824 | no |
| Sobic.008G179200 | 32.1187 | 5.4880 | 1.4671 | 3.7406 | 0.0001836 | 0.0080824 | up |
| Sobic.001G003200 | 10197.8778 | -1.2655 | 0.3387 | -3.7357 | 0.0001872 | 0.0082247 | no |
| Sobic.002G112400 | 25640.2780 | -1.2897 | 0.3455 | -3.7330 | 0.0001892 | 0.0082947 | no |
| Sobic.001G508600 | 65.0263 | 2.4095 | 0.6460 | 3.7300 | 0.0001915 | 0.0083775 | up |
| Sobic.010G068300 | 225.7704 | -2.0784 | 0.5577 | -3.7264 | 0.0001942 | 0.0084409 | down |
| Sobic.010G046601 | 130.9762 | -1.8367 | 0.4929 | -3.7264 | 0.0001942 | 0.0084409 | no |
| Sobic.001G454600 | 1187.0088 | -1.4038 | 0.3767 | -3.7261 | 0.0001945 | 0.0084409 | no |
| Sobic.001G414900 | 514.4715 | 1.1553 | 0.3101 | 3.7260 | 0.0001945 | 0.0084409 | no |
| Sobic.010G034600 | 317.4386 | -2.3645 | 0.6348 | -3.7247 | 0.0001956 | 0.008451 | down |
| Sobic.006G097800 | 89.8703 | -1.8921 | 0.5079 | -3.7251 | 0.0001952 | 0.008451 | no |
| Sobic.009G027900 | 122.2892 | 2.0271 | 0.5445 | 3.7227 | 0.0001971 | 0.0085015 | up |
| Sobic.004G279700 | 1302.3553 | -2.5143 | 0.6759 | -3.7197 | 0.0001994 | 0.0085165 | down |
| Sobic.007G071000 | 1475.0877 | -2.5104 | 0.6747 | -3.7207 | 0.0001986 | 0.0085165 | down |
| Sobic.001G067850 | 145.5492 | -1.6426 | 0.4416 | -3.7197 | 0.0001995 | 0.0085165 | no |
| Sobic.003G029400 | 1477.5322 | 2.6958 | 0.7247 | 3.7197 | 0.0001994 | 0.0085165 | up |
| Sobic.003G293700 | 95.8261 | 2.8654 | 0.7701 | 3.7210 | 0.0001984 | 0.0085165 | up |
| Sobic.002G362900 | 1333.3086 | 1.4995 | 0.4034 | 3.7176 | 0.0002012 | 0.0085698 | no |
| Sobic.003G292400 | 10.2420 | 6.5695 | 1.7678 | 3.7162 | 0.0002023 | 0.0085997 | up |
| Sobic.003G324400 | 1456.7542 | -2.5847 | 0.6958 | -3.7148 | 0.0002034 | 0.0086249 | down |
| Sobic.001G032000 | 1642.0359 | -1.3923 | 0.3748 | -3.7144 | 0.0002037 | 0.0086249 | no |
| Sobic.002G067000 | 6926.8217 | 2.5589 | 0.6892 | 3.7128 | 0.0002049 | 0.0086615 | up |
| Sobic.010G165000 | 938.9446 | -2.0027 | 0.5397 | -3.7109 | 0.0002065 | 0.0086923 | down |
| Sobic.003G036000 | 2430.4952 | 2.0048 | 0.5402 | 3.7111 | 0.0002064 | 0.0086923 | up |
| Sobic.001G269000 | 481.8703 | -2.6079 | 0.7031 | -3.7094 | 0.0002078 | 0.0087287 | down |
| Sobic.006G272900 | 249.4878 | -2.9991 | 0.8095 | -3.7048 | 0.0002115 | 0.0088681 | down |
| Sobic.010G214600 | 20781.1829 | -1.8690 | 0.5050 | -3.7007 | 0.000215 | 0.0089969 | no |
| Sobic.006G080600 | 1932.7010 | -1.8829 | 0.5091 | -3.6988 | 0.0002166 | 0.0090293 | no |
| Sobic.003G323500 | 1566.4218 | 3.4968 | 0.9454 | 3.6988 | 0.0002166 | 0.0090293 | up |
| Sobic.006G279200 | 374.7843 | 2.3463 | 0.6347 | 3.6966 | 0.0002185 | 0.0090886 | up |
| Sobic.004G051000 | 36.8818 | -2.0669 | 0.5593 | -3.6955 | 0.0002194 | 0.0091089 | down |

| Sobic.002G211200 | 168.8304 | -1.2894 | 0.3491 | -3.6934 | 0.0002213 | 0.0091674 | no |
| --- | --- | --- | --- | --- | --- | --- | --- |
| Sobic.009G002600 | 333.6033 | -2.5943 | 0.7026 | -3.6926 | 0.000222 | 0.0091783 | down |
| Sobic.004G182300 | 1532.2114 | 1.4612 | 0.3959 | 3.6906 | 0.0002237 | 0.0092333 | no |
| Sobic.002G001200 | 190.3274 | -2.1729 | 0.5889 | -3.6900 | 0.0002243 | 0.0092378 | down |
| Sobic.K030000 | 84.7191 | -2.2442 | 0.6085 | -3.6881 | 0.0002259 | 0.0092694 | down |
| Sobic.003G310800 | 616.0418 | -1.5406 | 0.4177 | -3.6881 | 0.0002259 | 0.0092694 | no |
| Sobic.003G321200 | 400.4651 | -1.9779 | 0.5368 | -3.6848 | 0.0002289 | 0.0093718 | no |
| Sobic.006G260800 | 1240.2589 | -0.9705 | 0.2634 | -3.6843 | 0.0002293 | 0.0093718 | no |
| Sobic.007G051700 | 5555.0340 | 0.9407 | 0.2555 | 3.6820 | 0.0002314 | 0.0094341 | no |
| Sobic.002G227900 | 15.1107 | 4.7004 | 1.2767 | 3.6817 | 0.0002317 | 0.0094341 | up |
| Sobic.002G033800 | 9164.7448 | -1.4229 | 0.3867 | -3.6795 | 0.0002337 | 0.0094957 | no |
| Sobic.006G188700 | 1535.5450 | -1.6185 | 0.4399 | -3.6790 | 0.0002341 | 0.0094961 | no |
| Sobic.006G121800 | 186.0212 | 2.1000 | 0.5711 | 3.6770 | 0.000236 | 0.0095513 | up |
| Sobic.002G383400 | 645.6700 | -1.7347 | 0.4719 | -3.6759 | 0.000237 | 0.0095574 | no |
| Sobic.005G136200 | 752.3257 | 2.7235 | 0.7409 | 3.6760 | 0.0002369 | 0.0095574 | up |
| Sobic.001G042301 | 105.7348 | -1.5635 | 0.4254 | -3.6752 | 0.0002376 | 0.0095642 | no |
| Sobic.003G396100 | 1340.7901 | -0.8633 | 0.2350 | -3.6741 | 0.0002387 | 0.0095873 | no |
| Sobic.003G308300 | 338.2494 | 1.1897 | 0.3240 | 3.6721 | 0.0002405 | 0.0096444 | no |
| Sobic.005G077800 | 468.2890 | -1.3418 | 0.3655 | -3.6713 | 0.0002413 | 0.0096511 | no |
| Sobic.004G203500 | 662.6925 | 2.1966 | 0.5984 | 3.6710 | 0.0002416 | 0.0096511 | up |
| Sobic.006G245500 | 26.6204 | -2.5853 | 0.7046 | -3.6691 | 0.0002434 | 0.0096964 | down |
| Sobic.005G122300 | 264.7756 | 2.2373 | 0.6098 | 3.6688 | 0.0002437 | 0.0096964 | up |
| Sobic.010G248900 | 50.6543 | 1.8049 | 0.4920 | 3.6683 | 0.0002442 | 0.0096969 | no |
| Sobic.008G098700 | 93.1804 | 1.9243 | 0.5249 | 3.6662 | 0.0002462 | 0.0097576 | no |
| Sobic.008G052000 | 227.3568 | 2.5179 | 0.6871 | 3.6645 | 0.0002479 | 0.0098067 | up |
| Sobic.009G222000 | 22.0689 | 3.6642 | 1.0005 | 3.6623 | 0.00025 | 0.0098729 | up |
| Sobic.003G075000 | 641.0614 | -2.6649 | 0.7279 | -3.6609 | 0.0002513 | 0.0099032 | down |
| Sobic.002G033900 | ######### | -1.7326 | 0.4733 | -3.6605 | 0.0002517 | 0.0099032 | no |
| Sobic.001G369500 | 473.0466 | -1.6145 | 0.4411 | -3.6599 | 0.0002523 | 0.0099096 | no |
| Sobic.006G206800 | 793.6557 | 3.0594 | 0.8376 | 3.6527 | 0.0002595 | 0.0101703 | up |
| Sobic.009G002200 | 46.4912 | -2.2491 | 0.6159 | -3.6516 | 0.0002606 | 0.0101949 | down |
| Sobic.006G228400 | 285.0942 | 1.8763 | 0.5144 | 3.6474 | 0.0002649 | 0.0103465 | no |
| Sobic.009G054500 | 74.9453 | 2.3011 | 0.6312 | 3.6458 | 0.0002665 | 0.0103894 | up |
| Sobic.010G011800 | 540.7349 | -1.3978 | 0.3835 | -3.6451 | 0.0002673 | 0.0104003 | no |
| Sobic.001G521500 | 6682.7195 | 1.3205 | 0.3624 | 3.6435 | 0.0002689 | 0.0104369 | no |
| Sobic.002G374600 | 16087.0942 | 1.6924 | 0.4645 | 3.6432 | 0.0002692 | 0.0104369 | no |
| Sobic.002G217700 | 1188.1099 | 1.1349 | 0.3119 | 3.6382 | 0.0002745 | 0.0106224 | no |
| Sobic.005G050200 | 19761.7821 | -1.8305 | 0.5034 | -3.6361 | 0.0002768 | 0.0106913 | no |
| Sobic.004G061400 | 467.3664 | 1.8854 | 0.5186 | 3.6355 | 0.0002774 | 0.0106948 | no |
| Sobic.003G239600 | 138.0716 | -1.3182 | 0.3629 | -3.6325 | 0.0002807 | 0.0108015 | no |
| Sobic.008G090000 | 109.2001 | 1.3545 | 0.3730 | 3.6314 | 0.0002819 | 0.0108277 | no |
| Sobic.008G164000 | 5.9351 | -6.2561 | 1.7232 | -3.6305 | 0.0002829 | 0.0108474 | down |
| Sobic.007G037100 | 5875.0343 | -1.3354 | 0.3680 | -3.6292 | 0.0002843 | 0.0108817 | no |
| Sobic.002G367600 | 332.8756 | -3.1130 | 0.8586 | -3.6259 | 0.000288 | 0.0109325 | down |
| Sobic.002G324400 | 22473.0535 | -1.4763 | 0.4072 | -3.6257 | 0.0002882 | 0.0109325 | no |
| Sobic.002G225300 | 717.1110 | 1.7390 | 0.4796 | 3.6260 | 0.0002879 | 0.0109325 | no |
| Sobic.007G227300 | 100.2107 | 2.2066 | 0.6083 | 3.6274 | 0.0002863 | 0.0109325 | up |
| Sobic.009G111000 | 58.3496 | 2.4104 | 0.6646 | 3.6268 | 0.0002869 | 0.0109325 | up |
| Sobic.003G050300 | 41.3881 | -3.9523 | 1.0904 | -3.6245 | 0.0002895 | 0.0109431 | down |
| Sobic.006G206000 | 6437.4775 | -0.9246 | 0.2551 | -3.6247 | 0.0002893 | 0.0109431 | no |
| Sobic.003G348200 | 5340.5645 | 1.0729 | 0.2961 | 3.6232 | 0.000291 | 0.0109783 | no |
| Sobic.010G189300 | ######### | -2.0946 | 0.5784 | -3.6214 | 0.000293 | 0.0110343 | down |
| Sobic.002G127400 | 531.5952 | 0.9565 | 0.2643 | 3.6187 | 0.0002961 | 0.0111313 | no |
| Sobic.001G524100 | 670.9416 | -1.2322 | 0.3408 | -3.6157 | 0.0002995 | 0.0112185 | no |
| Sobic.001G318200 | 400.5227 | 2.4096 | 0.6663 | 3.6161 | 0.000299 | 0.0112185 | up |
| Sobic.006G219200 | 89.4446 | 2.4880 | 0.6886 | 3.6129 | 0.0003028 | 0.0113229 | up |
| Sobic.009G181300 | 80.3208 | 2.0528 | 0.5690 | 3.6079 | 0.0003086 | 0.0115204 | up |

| Sobic.001G187300 | 6.0912 | -6.2949 | 1.7457 | -3.6060 | 0.000311 | 0.0115377 | down |
| --- | --- | --- | --- | --- | --- | --- | --- |
| Sobic.003G083200 | 354.6328 | 1.8466 | 0.5121 | 3.6057 | 0.0003113 | 0.0115377 | no |
| Sobic.004G273100 | 79.4117 | 2.1729 | 0.6024 | 3.6070 | 0.0003097 | 0.0115377 | up |
| Sobic.004G201100 | 115.7691 | 2.3093 | 0.6404 | 3.6061 | 0.0003108 | 0.0115377 | up |
| Sobic.001G101400 | 67.2400 | 3.6770 | 1.0199 | 3.6052 | 0.000312 | 0.0115418 | up |
| Sobic.004G263500 | 1343.1663 | 1.3810 | 0.3833 | 3.6025 | 0.0003152 | 0.0116413 | no |
| Sobic.008G139900 | 5566.0119 | -1.7455 | 0.4846 | -3.6016 | 0.0003162 | 0.0116592 | no |
| Sobic.001G264400 | 20.2731 | 3.3240 | 0.9233 | 3.6001 | 0.000318 | 0.0117054 | up |
| Sobic.002G210800 | 32.2256 | 3.0654 | 0.8516 | 3.5995 | 0.0003189 | 0.0117158 | up |
| Sobic.006G102400 | 428.2329 | -1.4005 | 0.3893 | -3.5977 | 0.000321 | 0.0117731 | no |
| Sobic.006G011300 | 4179.0777 | -1.0314 | 0.2867 | -3.5970 | 0.000322 | 0.0117876 | no |
| Sobic.003G342100 | 10835.4686 | -1.4899 | 0.4143 | -3.5963 | 0.0003227 | 0.0117959 | no |
| Sobic.001G086400 | 294.6881 | 2.7495 | 0.7651 | 3.5936 | 0.0003262 | 0.0119002 | up |
| Sobic.009G130700 | 6908.5989 | -1.8510 | 0.5156 | -3.5902 | 0.0003304 | 0.0120052 | no |
| Sobic.007G185100 | 270.3855 | -1.7623 | 0.4909 | -3.5898 | 0.0003309 | 0.0120052 | no |
| Sobic.001G281100 | 1910.2433 | 0.8397 | 0.2339 | 3.5899 | 0.0003308 | 0.0120052 | no |
| Sobic.002G308600 | 14.8803 | 6.1272 | 1.7070 | 3.5895 | 0.0003313 | 0.0120052 | up |
| Sobic.001G280000 | 1876.0105 | 2.2136 | 0.6171 | 3.5874 | 0.000334 | 0.0120836 | up |
| Sobic.001G107400 | 1335.7847 | -1.2620 | 0.3522 | -3.5830 | 0.0003397 | 0.0122459 | no |
| Sobic.003G267700 | 55.3842 | 1.9667 | 0.5489 | 3.5834 | 0.0003392 | 0.0122459 | no |
| Sobic.010G157100 | 232.2944 | 2.5706 | 0.7177 | 3.5819 | 0.0003411 | 0.0122771 | up |
| Sobic.003G431700 | 85077.2874 | -1.4880 | 0.4156 | -3.5802 | 0.0003433 | 0.0123324 | no |
| Sobic.002G375701 | 187.1507 | -1.8757 | 0.5245 | -3.5761 | 0.0003487 | 0.0124863 | no |
| Sobic.001G516600 | 1098.5308 | 1.1832 | 0.3308 | 3.5762 | 0.0003486 | 0.0124863 | no |
| Sobic.001G334400 | 13.1155 | -4.1286 | 1.1558 | -3.5721 | 0.0003541 | 0.012658 | down |
| Sobic.001G357000 | 19.9887 | -5.0695 | 1.4207 | -3.5684 | 0.0003592 | 0.0128164 | down |
| Sobic.007G209100 | 15.4135 | -5.1688 | 1.4487 | -3.5679 | 0.0003599 | 0.0128202 | down |
| Sobic.009G046200 | 32.8354 | 3.7675 | 1.0562 | 3.5670 | 0.0003611 | 0.0128404 | up |
| Sobic.008G185600 | 684.9276 | 1.3520 | 0.3791 | 3.5659 | 0.0003626 | 0.0128724 | no |
| Sobic.007G200500 | 1580.8514 | 0.8446 | 0.2369 | 3.5648 | 0.0003641 | 0.0129019 | no |
| Sobic.006G097000 | 133.9637 | 1.9807 | 0.5557 | 3.5644 | 0.0003646 | 0.0129019 | no |
| Sobic.004G071401 | 15.7051 | 4.8151 | 1.3512 | 3.5635 | 0.0003659 | 0.012927 | up |
| Sobic.002G272700 | 57930.5941 | -2.1717 | 0.6098 | -3.5614 | 0.0003689 | 0.012988 | down |
| Sobic.006G249400 | 3750.6273 | -2.1458 | 0.6025 | -3.5615 | 0.0003688 | 0.012988 | down |
| Sobic.002G260600 | 6.5839 | -6.4132 | 1.8012 | -3.5605 | 0.0003702 | 0.0130106 | down |
| Sobic.003G126600 | 721.1635 | 1.2370 | 0.3475 | 3.5599 | 0.0003711 | 0.0130206 | no |
| Sobic.004G141000 | 8541.8141 | -1.2444 | 0.3498 | -3.5575 | 0.0003744 | 0.0130854 | no |
| Sobic.010G182966 | 36.1167 | 2.3239 | 0.6534 | 3.5568 | 0.0003754 | 0.0130854 | up |
| Sobic.004G222000 | 4330.2358 | 2.7628 | 0.7767 | 3.5570 | 0.0003751 | 0.0130854 | up |
| Sobic.004G133600 | 23.1480 | 3.1420 | 0.8832 | 3.5574 | 0.0003746 | 0.0130854 | up |
| Sobic.002G155400 | 140.5136 | 2.1858 | 0.6147 | 3.5562 | 0.0003762 | 0.0130934 | up |
| Sobic.003G124750 | 673.6429 | 1.4451 | 0.4066 | 3.5545 | 0.0003787 | 0.0131563 | no |
| Sobic.010G244500 | 445.4643 | -2.1499 | 0.6051 | -3.5531 | 0.0003807 | 0.0132066 | down |
| Sobic.003G291600 | 729.3936 | 1.7278 | 0.4865 | 3.5516 | 0.0003828 | 0.0132572 | no |
| Sobic.005G014000 | 85.6955 | 2.9213 | 0.8229 | 3.5501 | 0.0003851 | 0.0133136 | up |
| Sobic.004G226600 | 418.7954 | 1.1323 | 0.3195 | 3.5441 | 0.000394 | 0.0135991 | no |
| Sobic.010G229450 | 73774.1411 | -1.5809 | 0.4463 | -3.5426 | 0.0003962 | 0.013653 | no |
| Sobic.003G099500 | 13.9612 | -5.5734 | 1.5747 | -3.5393 | 0.0004013 | 0.0138046 | down |
| Sobic.002G009700 | 571.0624 | 1.6438 | 0.4646 | 3.5377 | 0.0004036 | 0.0138418 | no |
| Sobic.003G275300 | 1293.8073 | 1.8263 | 0.5162 | 3.5377 | 0.0004037 | 0.0138418 | no |
| Sobic.003G018000 | 7.2209 | -5.5481 | 1.5691 | -3.5358 | 0.0004065 | 0.0138461 | down |
| Sobic.006G169200 | 371.9134 | -1.9370 | 0.5477 | -3.5369 | 0.0004049 | 0.0138461 | no |
| Sobic.001G029100 | 6915.8966 | -1.2229 | 0.3459 | -3.5350 | 0.0004077 | 0.0138461 | no |
| Sobic.006G148000 | 138.1517 | 1.2214 | 0.3454 | 3.5363 | 0.0004058 | 0.0138461 | no |
| Sobic.003G216166 | 43.9842 | 2.8573 | 0.8082 | 3.5352 | 0.0004074 | 0.0138461 | up |
| Sobic.009G136100 | 141.5019 | 3.0508 | 0.8630 | 3.5352 | 0.0004075 | 0.0138461 | up |
| Sobic.002G372800 | 286.4389 | 1.4282 | 0.4042 | 3.5335 | 0.0004101 | 0.0138876 | no |

| Sobic.006G226400 | 922.7655 | 2.6689 | 0.7553 | 3.5334 | 0.0004103 | 0.0138876 | up |
| --- | --- | --- | --- | --- | --- | --- | --- |
| Sobic.003G254600 | 3669.3043 | -1.2812 | 0.3628 | -3.5315 | 0.0004132 | 0.0138993 | no |
| Sobic.001G314700 | 348.2294 | 1.1962 | 0.3387 | 3.5319 | 0.0004126 | 0.0138993 | no |
| Sobic.009G252500 | 271.4384 | 1.6134 | 0.4569 | 3.5315 | 0.0004132 | 0.0138993 | no |
| Sobic.006G132500 | 3474.9959 | 1.7980 | 0.5090 | 3.5327 | 0.0004114 | 0.0138993 | no |
| Sobic.003G420201 | 86.9581 | -1.3150 | 0.3724 | -3.5306 | 0.0004146 | 0.0139214 | no |
| Sobic.002G222700 | 198.5348 | -1.8643 | 0.5282 | -3.5294 | 0.0004165 | 0.0139632 | no |
| Sobic.010G249900 | 131.2211 | 1.2285 | 0.3482 | 3.5276 | 0.0004193 | 0.0140373 | no |
| Sobic.001G187000 | 20.7082 | -2.4237 | 0.6873 | -3.5262 | 0.0004215 | 0.0140879 | down |
| Sobic.009G142600 | 738.3271 | -1.8195 | 0.5161 | -3.5258 | 0.0004222 | 0.0140879 | no |
| Sobic.003G365200 | 4341.6634 | -1.7533 | 0.4975 | -3.5240 | 0.0004251 | 0.0141637 | no |
| Sobic.002G421900 | 4343.2672 | -0.9215 | 0.2616 | -3.5220 | 0.0004283 | 0.0142474 | no |
| Sobic.002G328800 | 201.9333 | -2.2890 | 0.6504 | -3.5194 | 0.0004325 | 0.0143397 | down |
| Sobic.008G002700 | 538.5563 | -1.1192 | 0.3180 | -3.5195 | 0.0004324 | 0.0143397 | no |
| Sobic.001G109000 | 149.7341 | 3.5655 | 1.0132 | 3.5190 | 0.0004331 | 0.0143397 | up |
| Sobic.003G345200 | 106.2272 | 1.6910 | 0.4814 | 3.5127 | 0.0004436 | 0.0146389 | no |
| Sobic.006G174000 | 16.2635 | 6.2580 | 1.7813 | 3.5131 | 0.0004429 | 0.0146389 | up |
| Sobic.001G319500 | 853.5450 | 2.2033 | 0.6275 | 3.5109 | 0.0004466 | 0.0147153 | up |
| Sobic.001G119000 | 253.6226 | 2.3050 | 0.6567 | 3.5097 | 0.0004486 | 0.014758 | up |
| Sobic.003G376100 | 1097.4824 | 1.4547 | 0.4146 | 3.5086 | 0.0004505 | 0.0147968 | no |
| Sobic.004G195300 | 20.8612 | 3.5619 | 1.0156 | 3.5072 | 0.0004528 | 0.0148507 | up |
| Sobic.005G110523 | 575.9434 | -1.8061 | 0.5152 | -3.5056 | 0.0004555 | 0.0148941 | no |
| Sobic.004G133500 | 810.5713 | 1.4496 | 0.4135 | 3.5059 | 0.0004551 | 0.0148941 | no |
| Sobic.002G239500 | 7.7088 | -5.6416 | 1.6099 | -3.5044 | 0.0004577 | 0.0149269 | down |
| Sobic.004G042600 | 102.6197 | 2.4284 | 0.6930 | 3.5042 | 0.000458 | 0.0149269 | up |
| Sobic.006G173200 | 556.4531 | 1.1137 | 0.3179 | 3.5036 | 0.0004589 | 0.0149358 | no |
| Sobic.008G063500 | ######### | -1.8100 | 0.5167 | -3.5030 | 0.00046 | 0.0149481 | no |
| Sobic.001G180500 | 74.9684 | -1.5794 | 0.4510 | -3.5019 | 0.0004619 | 0.0149857 | no |
| Sobic.001G401800 | 345.7000 | 1.2578 | 0.3596 | 3.4976 | 0.0004695 | 0.0152089 | no |
| Sobic.001G485901 | 1687.3276 | -1.3937 | 0.3988 | -3.4947 | 0.0004745 | 0.0153253 | no |
| Sobic.003G394500 | 1251.5080 | 0.7992 | 0.2287 | 3.4950 | 0.0004741 | 0.0153253 | no |
| Sobic.009G113700 | 363.6318 | -2.5842 | 0.7399 | -3.4927 | 0.0004782 | 0.0154176 | down |
| Sobic.009G132400 | 21.5511 | 4.0625 | 1.1633 | 3.4923 | 0.0004789 | 0.0154176 | up |
| Sobic.004G027600 | 750.8638 | -1.7415 | 0.4990 | -3.4902 | 0.0004827 | 0.015519 | no |
| Sobic.003G030400 | 679.4911 | -1.0069 | 0.2886 | -3.4892 | 0.0004844 | 0.0155495 | no |
| Sobic.002G021132 | 243.8080 | 1.5154 | 0.4345 | 3.4875 | 0.0004876 | 0.0156283 | no |
| Sobic.002G032900 | 243.5390 | -1.3225 | 0.3794 | -3.4859 | 0.0004905 | 0.015697 | no |
| Sobic.006G081600 | 309.1748 | 1.6424 | 0.4712 | 3.4852 | 0.0004918 | 0.0156998 | no |
| Sobic.001G352700 | 258.3582 | 1.9914 | 0.5714 | 3.4850 | 0.0004921 | 0.0156998 | no |
| Sobic.004G099700 | 17886.2988 | 0.8740 | 0.2511 | 3.4805 | 0.0005005 | 0.0158963 | no |
| Sobic.003G170400 | 30.2523 | 2.2520 | 0.6470 | 3.4806 | 0.0005004 | 0.0158963 | up |
| Sobic.006G174400 | 100.9184 | 2.7819 | 0.7993 | 3.4805 | 0.0005005 | 0.0158963 | up |
| Sobic.007G150100 | 4408.9218 | -1.0317 | 0.2967 | -3.4772 | 0.0005067 | 0.0160691 | no |
| Sobic.008G050800 | 47.7355 | -1.7801 | 0.5122 | -3.4755 | 0.00051 | 0.0161243 | no |
| Sobic.005G102200 | 21.5870 | 5.8874 | 1.6940 | 3.4755 | 0.0005098 | 0.0161243 | up |
| Sobic.002G382100 | 43.2878 | 3.9229 | 1.1295 | 3.4731 | 0.0005146 | 0.0162454 | up |
| Sobic.010G231200 | 1135.1946 | -1.8393 | 0.5299 | -3.4710 | 0.0005186 | 0.0163482 | no |
| Sobic.002G179800 | 38.5741 | 2.4692 | 0.7115 | 3.4705 | 0.0005194 | 0.0163494 | up |
| Sobic.008G108400 | 1482.6043 | -1.0015 | 0.2886 | -3.4699 | 0.0005206 | 0.0163634 | no |
| Sobic.001G533600 | 523.3082 | -1.8048 | 0.5202 | -3.4693 | 0.0005219 | 0.0163769 | no |
| Sobic.004G152000 | 535.5203 | 1.3830 | 0.3987 | 3.4687 | 0.000523 | 0.0163886 | no |
| Sobic.001G102400 | 465.3777 | -1.6120 | 0.4651 | -3.4659 | 0.0005284 | 0.0165344 | no |
| Sobic.004G087000 | 85.4976 | -1.6598 | 0.4795 | -3.4615 | 0.0005372 | 0.0167103 | no |
| Sobic.002G289100 | 135.4363 | -1.6356 | 0.4725 | -3.4615 | 0.0005372 | 0.0167103 | no |
| Sobic.004G113800 | 4695.1957 | -0.8673 | 0.2505 | -3.4619 | 0.0005363 | 0.0167103 | no |
| Sobic.002G186900 | 510.4944 | 1.3415 | 0.3875 | 3.4617 | 0.0005367 | 0.0167103 | no |
| Sobic.003G123500 | 384.6931 | -1.3185 | 0.3810 | -3.4607 | 0.0005389 | 0.0167366 | no |

| Sobic.001G325200 | 2448.9994 | 0.9827 | 0.2843 | 3.4567 | 0.0005468 | 0.0169575 | no |
| --- | --- | --- | --- | --- | --- | --- | --- |
| Sobic.002G323000 | 174.9964 | -1.1956 | 0.3461 | -3.4548 | 0.0005507 | 0.0170338 | no |
| Sobic.004G321200 | 121.0474 | 1.3980 | 0.4047 | 3.4547 | 0.0005509 | 0.0170338 | no |
| Sobic.006G177800 | 9267.7065 | -3.4022 | 0.9851 | -3.4537 | 0.0005529 | 0.0170569 | down |
| Sobic.002G030500 | 20570.1576 | -1.0756 | 0.3114 | -3.4536 | 0.0005532 | 0.0170569 | no |
| Sobic.001G420900 | 254.0094 | -2.1843 | 0.6329 | -3.4512 | 0.000558 | 0.0171769 | down |
| Sobic.006G182000 | 1578.5778 | 2.2921 | 0.6642 | 3.4509 | 0.0005587 | 0.0171769 | up |
| Sobic.009G030600 | 59.2691 | 2.4192 | 0.7015 | 3.4489 | 0.0005629 | 0.017281 | up |
| Sobic.006G024600 | 862.6519 | -1.3494 | 0.3914 | -3.4473 | 0.0005663 | 0.0173208 | no |
| Sobic.004G193300 | 361.5929 | 1.2390 | 0.3594 | 3.4472 | 0.0005665 | 0.0173208 | no |
| Sobic.006G270500 | 59.9681 | 1.8912 | 0.5486 | 3.4471 | 0.0005667 | 0.0173208 | no |
| Sobic.008G187001 | 10.8605 | -3.8402 | 1.1152 | -3.4434 | 0.0005745 | 0.0175085 | down |
| Sobic.009G251400 | 32.8304 | 2.7698 | 0.8044 | 3.4434 | 0.0005745 | 0.0175085 | up |
| Sobic.004G182700 | 1284.3480 | -2.3931 | 0.6952 | -3.4421 | 0.0005772 | 0.0175639 | down |
| Sobic.001G341001 | 75.7461 | -1.7671 | 0.5137 | -3.4401 | 0.0005815 | 0.0175745 | no |
| Sobic.004G341200 | 2808.0810 | 0.9840 | 0.2860 | 3.4400 | 0.0005817 | 0.0175745 | no |
| Sobic.004G340900 | 259.1077 | 2.2516 | 0.6543 | 3.4411 | 0.0005794 | 0.0175745 | up |
| Sobic.008G085350 | 56.2085 | 2.8247 | 0.8208 | 3.4414 | 0.0005788 | 0.0175745 | up |
| Sobic.008G062700 | 12.3515 | 4.4456 | 1.2922 | 3.4404 | 0.0005809 | 0.0175745 | up |
| Sobic.001G520800 | 1498.6925 | -2.5185 | 0.7329 | -3.4365 | 0.0005892 | 0.0177776 | down |
| Sobic.002G228400 | 410.6573 | -1.2306 | 0.3583 | -3.4347 | 0.0005932 | 0.0178706 | no |
| Sobic.004G339800 | 149.3357 | -3.0384 | 0.8858 | -3.4302 | 0.0006031 | 0.0180104 | down |
| Sobic.002G329600 | 68800.8471 | -2.2363 | 0.6518 | -3.4309 | 0.0006015 | 0.0180104 | down |
| Sobic.003G169200 | 33.7096 | -1.9912 | 0.5803 | -3.4315 | 0.0006001 | 0.0180104 | no |
| Sobic.009G187100 | 558.9769 | -1.7931 | 0.5225 | -3.4316 | 0.0005999 | 0.0180104 | no |
| Sobic.007G047300 | 2119.5232 | 1.4471 | 0.4218 | 3.4308 | 0.0006018 | 0.0180104 | no |
| Sobic.006G084701 | 54.6029 | 2.0501 | 0.5977 | 3.4299 | 0.0006038 | 0.0180104 | up |
| Sobic.001G084500 | 33.4028 | 2.0994 | 0.6121 | 3.4300 | 0.0006036 | 0.0180104 | up |
| Sobic.005G087000 | ######### | -2.5039 | 0.7305 | -3.4276 | 0.0006089 | 0.0180773 | down |
| Sobic.009G191300 | 17540.0320 | -1.1883 | 0.3466 | -3.4283 | 0.0006074 | 0.0180773 | no |
| Sobic.006G259401 | 443.1731 | 1.2066 | 0.3521 | 3.4274 | 0.0006095 | 0.0180773 | no |
| Sobic.010G236500 | 1020.8259 | 1.2885 | 0.3759 | 3.4280 | 0.0006082 | 0.0180773 | no |
| Sobic.010G239900 | 857.5919 | -1.3677 | 0.3991 | -3.4270 | 0.0006104 | 0.0180784 | no |
| Sobic.003G012300 | 244.6405 | 1.4707 | 0.4296 | 3.4234 | 0.0006184 | 0.0182917 | no |
| Sobic.005G225700 | 12.2056 | 6.8238 | 1.9937 | 3.4227 | 0.0006201 | 0.0183165 | up |
| Sobic.010G118100 | 519.9734 | 1.3735 | 0.4016 | 3.4204 | 0.0006253 | 0.0184444 | no |
| Sobic.002G128900 | 3359.8743 | -0.8378 | 0.2450 | -3.4198 | 0.0006267 | 0.0184581 | no |
| Sobic.001G158200 | 34.3024 | -5.1100 | 1.4950 | -3.4180 | 0.0006307 | 0.018478 | down |
| Sobic.010G010900 | 334.3812 | -1.2385 | 0.3623 | -3.4181 | 0.0006307 | 0.018478 | no |
| Sobic.005G114800 | 1697.0706 | 0.8404 | 0.2458 | 3.4189 | 0.0006287 | 0.018478 | no |
| Sobic.001G370400 | 84.4617 | 2.6570 | 0.7774 | 3.4180 | 0.0006309 | 0.018478 | up |
| Sobic.005G186800 | 5719.6435 | -2.2374 | 0.6548 | -3.4167 | 0.0006337 | 0.0185368 | down |
| Sobic.001G518800 | 2117.8205 | -2.5613 | 0.7503 | -3.4135 | 0.0006413 | 0.0187317 | down |
| Sobic.010G161201 | 580.0353 | -1.2099 | 0.3550 | -3.4086 | 0.0006531 | 0.0190489 | no |
| Sobic.004G191500 | 5955.7017 | 0.8738 | 0.2564 | 3.4080 | 0.0006544 | 0.0190608 | no |
| Sobic.002G339200 | 51009.3554 | -2.1333 | 0.6265 | -3.4052 | 0.0006612 | 0.0192323 | down |
| Sobic.002G209900 | 58.0938 | -1.5450 | 0.4538 | -3.4046 | 0.0006625 | 0.0192444 | no |
| Sobic.006G146000 | 318.0486 | 1.9222 | 0.5648 | 3.4035 | 0.0006653 | 0.0192988 | no |
| Sobic.003G088450 | 41.6862 | -2.2035 | 0.6478 | -3.4015 | 0.0006703 | 0.0194164 | down |
| Sobic.003G145300 | 6.7798 | -5.4583 | 1.6054 | -3.4001 | 0.0006737 | 0.0194621 | down |
| Sobic.010G135300 | 169.0682 | 1.2803 | 0.3765 | 3.4002 | 0.0006733 | 0.0194621 | no |
| Sobic.004G303500 | 1334.5520 | -1.6038 | 0.4718 | -3.3994 | 0.0006754 | 0.0194838 | no |
| Sobic.003G020600 | 4974.2617 | -1.3443 | 0.3956 | -3.3978 | 0.0006793 | 0.0195426 | no |
| Sobic.006G183600 | 51.5901 | 4.4970 | 1.3234 | 3.3981 | 0.0006785 | 0.0195426 | up |
| Sobic.004G175500 | 10931.0631 | -1.3206 | 0.3889 | -3.3957 | 0.0006847 | 0.0196713 | no |
| Sobic.001G310500 | 2839.5024 | 1.2807 | 0.3773 | 3.3945 | 0.0006876 | 0.0197303 | no |
| Sobic.001G410200 | 7599.4978 | 1.4846 | 0.4375 | 3.3934 | 0.0006903 | 0.0197783 | no |

| Sobic.009G111400 | 155.3454 | 1.7646 | 0.5202 | 3.3922 | 0.0006934 | 0.0198181 | no |
| --- | --- | --- | --- | --- | --- | --- | --- |
| Sobic.002G014200 | 37.2018 | 2.3644 | 0.6970 | 3.3921 | 0.0006935 | 0.0198181 | up |
| Sobic.006G115700 | 65.0120 | -1.6902 | 0.4984 | -3.3910 | 0.0006965 | 0.0198756 | no |
| Sobic.009G140000 | 21819.7659 | -1.4156 | 0.4177 | -3.3887 | 0.0007022 | 0.0199915 | no |
| Sobic.007G076000 | 1150.0864 | 2.4330 | 0.7180 | 3.3885 | 0.0007026 | 0.0199915 | up |
| Sobic.004G320600 | 34.1323 | 3.1297 | 0.9237 | 3.3883 | 0.0007034 | 0.0199915 | up |
| Sobic.003G291700 | 677.2042 | 1.7800 | 0.5255 | 3.3870 | 0.0007067 | 0.0200597 | no |
| Sobic.008G157700 | 387.2469 | -1.5418 | 0.4558 | -3.3825 | 0.0007183 | 0.020361 | no |
| Sobic.004G305800 | 10005.4937 | -1.4771 | 0.4372 | -3.3786 | 0.0007285 | 0.0204856 | no |
| Sobic.004G058050 | 126.0153 | -1.3887 | 0.4110 | -3.3787 | 0.0007283 | 0.0204856 | no |
| Sobic.010G248301 | 4262.1256 | -1.1753 | 0.3478 | -3.3798 | 0.0007253 | 0.0204856 | no |
| Sobic.002G338100 | 10283.3179 | -1.0628 | 0.3146 | -3.3786 | 0.0007284 | 0.0204856 | no |
| Sobic.003G233000 | 694.1493 | 1.9021 | 0.5628 | 3.3795 | 0.0007261 | 0.0204856 | no |
| Sobic.010G162900 | 637.4221 | 1.9290 | 0.5707 | 3.3799 | 0.0007252 | 0.0204856 | no |
| Sobic.008G131200 | 2416.9071 | -1.3387 | 0.3965 | -3.3764 | 0.0007345 | 0.0205705 | no |
| Sobic.006G160700 | 550.4951 | 1.1387 | 0.3373 | 3.3764 | 0.0007343 | 0.0205705 | no |
| Sobic.003G228200 | 558.1083 | 1.3293 | 0.3937 | 3.3766 | 0.0007338 | 0.0205705 | no |
| Sobic.005G192500 | 528.0991 | -1.9176 | 0.5680 | -3.3759 | 0.0007359 | 0.020582 | no |
| Sobic.004G077300 | 175.1620 | 1.5963 | 0.4729 | 3.3753 | 0.0007372 | 0.0205928 | no |
| Sobic.001G073500 | 3594.6444 | -1.5331 | 0.4544 | -3.3739 | 0.0007411 | 0.0206725 | no |
| Sobic.006G176100 | 851.9745 | -1.1935 | 0.3539 | -3.3729 | 0.0007439 | 0.0207232 | no |
| Sobic.003G435800 | 9930.0931 | -1.0496 | 0.3113 | -3.3720 | 0.0007463 | 0.0207352 | no |
| Sobic.003G186300 | 173.7367 | 1.2154 | 0.3605 | 3.3720 | 0.0007462 | 0.0207352 | no |
| Sobic.003G071901 | 6916.1636 | -1.4895 | 0.4428 | -3.3642 | 0.0007677 | 0.0213016 | no |
| Sobic.002G323700 | 66.9924 | -2.6714 | 0.7943 | -3.3633 | 0.0007703 | 0.0213296 | down |
| Sobic.001G497200 | 6304.9654 | -1.3265 | 0.3945 | -3.3627 | 0.0007717 | 0.0213296 | no |
| Sobic.007G011700 | 792.0948 | 1.5737 | 0.4680 | 3.3628 | 0.0007715 | 0.0213296 | no |
| Sobic.008G156900 | 20.5597 | -2.9481 | 0.8769 | -3.3618 | 0.0007744 | 0.0213755 | down |
| Sobic.010G110800 | 8.3538 | 5.2750 | 1.5700 | 3.3598 | 0.0007799 | 0.0214984 | up |
| Sobic.002G190100 | 469.7232 | -2.0392 | 0.6072 | -3.3583 | 0.0007843 | 0.0215922 | down |
| Sobic.010G102800 | 3968.9310 | -1.1623 | 0.3463 | -3.3562 | 0.0007901 | 0.0217248 | no |
| Sobic.008G186300 | 538.4165 | 1.2648 | 0.3770 | 3.3548 | 0.0007942 | 0.0217788 | no |
| Sobic.006G058732 | 47.7173 | 2.2917 | 0.6831 | 3.3549 | 0.0007938 | 0.0217788 | up |
| Sobic.009G000100 | 542.1485 | 1.2038 | 0.3590 | 3.3531 | 0.0007991 | 0.0218852 | no |
| Sobic.004G305200 | 67.5486 | 1.5171 | 0.4526 | 3.3521 | 0.000802 | 0.0219364 | no |
| Sobic.008G159901 | 202.6775 | -1.0994 | 0.3281 | -3.3505 | 0.0008066 | 0.0220337 | no |
| Sobic.005G137300 | 359.1519 | 2.1049 | 0.6284 | 3.3496 | 0.0008093 | 0.0220798 | up |
| Sobic.006G221101 | 239.6857 | -1.2803 | 0.3823 | -3.3486 | 0.0008122 | 0.0221306 | no |
| Sobic.008G047000 | 35999.1426 | -1.1435 | 0.3416 | -3.3476 | 0.000815 | 0.0221785 | no |
| Sobic.009G091850 | 3031.8739 | -1.6107 | 0.4812 | -3.3473 | 0.0008161 | 0.0221797 | no |
| Sobic.001G372600 | 712.7726 | -1.8490 | 0.5532 | -3.3424 | 0.0008307 | 0.022507 | no |
| Sobic.007G009200 | 37514.4078 | -1.4528 | 0.4346 | -3.3426 | 0.0008299 | 0.022507 | no |
| Sobic.008G143100 | 1059.0772 | 1.2632 | 0.3780 | 3.3421 | 0.0008314 | 0.022507 | no |
| Sobic.001G144100 | 11.4630 | -3.0118 | 0.9013 | -3.3417 | 0.0008327 | 0.0225114 | down |
| Sobic.002G117600 | 352.8883 | 0.9765 | 0.2923 | 3.3414 | 0.0008337 | 0.0225114 | no |
| Sobic.003G110700 | 2120.1997 | -1.2658 | 0.3790 | -3.3402 | 0.0008371 | 0.0225754 | no |
| Sobic.001G382100 | 10843.9008 | -2.1391 | 0.6405 | -3.3395 | 0.0008393 | 0.0226048 | down |
| Sobic.004G321800 | 567.1438 | 2.2983 | 0.6885 | 3.3383 | 0.0008431 | 0.0226775 | up |
| Sobic.001G371700 | 481.2826 | -1.2310 | 0.3689 | -3.3367 | 0.0008479 | 0.0227495 | no |
| Sobic.002G311300 | 56.2265 | 2.0553 | 0.6159 | 3.3369 | 0.0008472 | 0.0227495 | up |
| Sobic.001G532200 | 17.7313 | 2.6634 | 0.7991 | 3.3328 | 0.0008597 | 0.0230383 | up |
| Sobic.001G124800 | 633.8717 | -1.3786 | 0.4137 | -3.3323 | 0.0008613 | 0.0230514 | no |
| Sobic.002G297300 | 4827.8148 | -1.9105 | 0.5735 | -3.3314 | 0.0008641 | 0.0230971 | no |
| Sobic.003G232900 | 398.2993 | 1.5849 | 0.4762 | 3.3282 | 0.000874 | 0.023331 | no |
| Sobic.008G024700 | 203.0961 | -1.0820 | 0.3253 | -3.3264 | 0.0008797 | 0.0233956 | no |
| Sobic.007G028200 | 2702.8942 | -0.8679 | 0.2609 | -3.3265 | 0.0008793 | 0.0233956 | no |
| Sobic.004G214000 | 282.9314 | 3.1331 | 0.9417 | 3.3270 | 0.0008778 | 0.0233956 | up |

| Sobic.009G134600 | 7289.5851 | 0.8373 | 0.2518 | 3.3259 | 0.0008814 | 0.0234114 | no |
| --- | --- | --- | --- | --- | --- | --- | --- |
| Sobic.010G080100 | 201.2936 | -2.3579 | 0.7099 | -3.3217 | 0.0008947 | 0.0236574 | down |
| Sobic.002G095400 | 96.9925 | -2.3120 | 0.6963 | -3.3206 | 0.0008983 | 0.0236574 | down |
| Sobic.009G055900 | 244.3849 | -1.6261 | 0.4896 | -3.3209 | 0.0008973 | 0.0236574 | no |
| Sobic.006G201700 | 234.3262 | -0.9714 | 0.2925 | -3.3211 | 0.0008965 | 0.0236574 | no |
| Sobic.008G003700 | 4071.8675 | 1.0103 | 0.3041 | 3.3221 | 0.0008934 | 0.0236574 | no |
| Sobic.005G069900 | 24.8109 | 2.3855 | 0.7184 | 3.3206 | 0.0008984 | 0.0236574 | up |
| Sobic.003G429300 | 76.1178 | 3.0583 | 0.9210 | 3.3205 | 0.0008986 | 0.0236574 | up |
| Sobic.004G235200 | 44449.2761 | -1.5211 | 0.4583 | -3.3194 | 0.0009022 | 0.0237239 | no |
| Sobic.004G000300 | 971.7277 | -1.2961 | 0.3905 | -3.3186 | 0.0009047 | 0.0237607 | no |
| Sobic.003G068200 | 43.5975 | 1.8443 | 0.5559 | 3.3175 | 0.0009083 | 0.0238256 | no |
| Sobic.006G211000 | 579.4520 | -1.3490 | 0.4068 | -3.3164 | 0.0009119 | 0.0238898 | no |
| Sobic.003G257900 | 112.0304 | 3.0653 | 0.9244 | 3.3159 | 0.0009136 | 0.0239042 | up |
| Sobic.004G056900 | ######### | -1.8815 | 0.5678 | -3.3138 | 0.0009203 | 0.0239628 | no |
| Sobic.005G061300 | 203.9991 | -1.7415 | 0.5255 | -3.3139 | 0.0009202 | 0.0239628 | no |
| Sobic.002G287900 | 63.1649 | -1.5673 | 0.4730 | -3.3138 | 0.0009204 | 0.0239628 | no |
| Sobic.006G143100 | 7159.7113 | -1.0275 | 0.3101 | -3.3139 | 0.0009199 | 0.0239628 | no |
| Sobic.010G201444 | 62.0477 | 3.0311 | 0.9149 | 3.3130 | 0.0009231 | 0.024004 | up |
| Sobic.002G137850 | 6.8702 | -6.4629 | 1.9520 | -3.3110 | 0.0009298 | 0.0240683 | down |
| Sobic.003G383800 | 18.9129 | -3.3353 | 1.0072 | -3.3114 | 0.0009283 | 0.0240683 | down |
| Sobic.001G299400 | 765.4112 | -0.9698 | 0.2930 | -3.3105 | 0.0009313 | 0.0240683 | no |
| Sobic.002G311200 | 10856.7464 | 1.4820 | 0.4476 | 3.3106 | 0.0009311 | 0.0240683 | no |
| Sobic.008G034300 | 90.5341 | 2.1792 | 0.6581 | 3.3114 | 0.0009283 | 0.0240683 | up |
| Sobic.010G006800 | 41.6349 | -3.0867 | 0.9332 | -3.3076 | 0.0009409 | 0.0242878 | down |
| Sobic.001G381900 | 164.9282 | -1.1931 | 0.3609 | -3.3059 | 0.0009466 | 0.0243748 | no |
| Sobic.002G377200 | 57.2921 | 1.4039 | 0.4247 | 3.3059 | 0.0009466 | 0.0243748 | no |
| Sobic.003G215600 | 16.4207 | 5.4800 | 1.6583 | 3.3046 | 0.0009511 | 0.0244613 | up |
| Sobic.003G347200 | 36.3703 | -2.3924 | 0.7241 | -3.3040 | 0.0009531 | 0.024482 | down |
| Sobic.003G440900 | 280.1630 | -1.0693 | 0.3237 | -3.3029 | 0.0009568 | 0.0245168 | no |
| Sobic.009G225100 | 238.7447 | 1.2629 | 0.3824 | 3.3031 | 0.0009563 | 0.0245168 | no |
| Sobic.010G179150 | 765.3323 | -1.8679 | 0.5659 | -3.3010 | 0.0009635 | 0.0246023 | no |
| Sobic.009G122000 | 92.5627 | -1.4715 | 0.4457 | -3.3016 | 0.0009614 | 0.0246023 | no |
| Sobic.001G434900 | 1862.9966 | 1.3450 | 0.4074 | 3.3009 | 0.0009636 | 0.0246023 | no |
| Sobic.001G177000 | ######### | -2.0383 | 0.6179 | -3.2987 | 0.0009713 | 0.0247423 | down |
| Sobic.008G091300 | 247.6245 | 0.9058 | 0.2746 | 3.2987 | 0.0009714 | 0.0247423 | no |
| Sobic.010G045833 | 526.1591 | 1.0418 | 0.3160 | 3.2973 | 0.0009763 | 0.0248064 | no |
| Sobic.001G435000 | 122.5044 | 2.4728 | 0.7499 | 3.2975 | 0.0009756 | 0.0248064 | up |
| Sobic.010G016900 | 3687.7073 | -0.9970 | 0.3025 | -3.2959 | 0.0009809 | 0.0248639 | no |
| Sobic.007G165701 | 1425.9553 | 1.7930 | 0.5440 | 3.2962 | 0.0009801 | 0.0248639 | no |
| Sobic.007G120800 | 1458.2798 | 0.7804 | 0.2371 | 3.2911 | 0.000998 | 0.0252658 | no |
| Sobic.001G447700 | 2160.2139 | -1.1657 | 0.3543 | -3.2906 | 0.0009997 | 0.0252801 | no |
| Sobic.001G482300 | 2532.9249 | -1.2744 | 0.3876 | -3.2882 | 0.0010083 | 0.0254652 | no |
| Sobic.006G054900 | 814.9999 | 2.7027 | 0.8225 | 3.2859 | 0.0010166 | 0.0256203 | up |
| Sobic.010G169000 | 587.5598 | 2.8820 | 0.8771 | 3.2858 | 0.0010168 | 0.0256203 | up |
| Sobic.005G079500 | 174.9912 | 1.8025 | 0.5487 | 3.2854 | 0.0010185 | 0.0256311 | no |
| Sobic.004G267000 | 393.7618 | -2.4822 | 0.7557 | -3.2845 | 0.0010217 | 0.0256524 | down |
| Sobic.007G081100 | 23.5121 | 2.2683 | 0.6906 | 3.2845 | 0.0010218 | 0.0256524 | up |
| Sobic.008G169400 | 333.1010 | 2.5815 | 0.7863 | 3.2831 | 0.0010269 | 0.0257495 | up |
| Sobic.002G345300 | 1647.4587 | -0.8809 | 0.2685 | -3.2814 | 0.0010329 | 0.0258528 | no |
| Sobic.006G274400 | 258.4952 | 1.3022 | 0.3969 | 3.2813 | 0.0010334 | 0.0258528 | no |
| Sobic.003G333000 | 5.5026 | -6.1452 | 1.8732 | -3.2806 | 0.0010358 | 0.0258826 | down |
| Sobic.003G037200 | 414.5400 | -1.8363 | 0.5600 | -3.2794 | 0.0010402 | 0.0259615 | no |
| Sobic.006G151500 | 15.6538 | -5.7365 | 1.7502 | -3.2776 | 0.001047 | 0.0260675 | down |
| Sobic.004G211400 | 5110.3874 | -0.9571 | 0.2920 | -3.2779 | 0.0010459 | 0.0260675 | no |
| Sobic.010G001900 | 403.8643 | 1.6399 | 0.5004 | 3.2772 | 0.0010484 | 0.0260719 | no |
| Sobic.008G157600 | ######### | -1.2411 | 0.3788 | -3.2765 | 0.0010512 | 0.0261117 | no |
| Sobic.003G044900 | 2159.1808 | -1.1496 | 0.3511 | -3.2744 | 0.0010589 | 0.0262729 | no |

| Sobic.001G430400 | 175.3887 | 1.2034 | 0.3676 | 3.2737 | 0.0010616 | 0.026307 | no |
| --- | --- | --- | --- | --- | --- | --- | --- |
| Sobic.003G233600 | 1646.1960 | 0.9090 | 0.2778 | 3.2722 | 0.0010672 | 0.0264164 | no |
| Sobic.004G179700 | 590.2118 | -0.9456 | 0.2891 | -3.2710 | 0.0010716 | 0.0264618 | no |
| Sobic.008G072500 | 196.0475 | 1.0035 | 0.3068 | 3.2711 | 0.0010712 | 0.0264618 | no |
| Sobic.009G011600 | 2291.1946 | -0.7899 | 0.2415 | -3.2706 | 0.0010731 | 0.0264687 | no |
| Sobic.003G142900 | 392.8256 | -1.4228 | 0.4351 | -3.2698 | 0.0010762 | 0.0265145 | no |
| Sobic.006G028600 | 19.5482 | 2.7828 | 0.8513 | 3.2689 | 0.0010797 | 0.0265694 | up |
| Sobic.002G198600 | 14411.5646 | -1.2851 | 0.3934 | -3.2669 | 0.0010874 | 0.026728 | no |
| Sobic.003G398800 | 5717.5339 | -1.4803 | 0.4532 | -3.2660 | 0.0010908 | 0.0267642 | no |
| Sobic.003G054400 | 1341.3807 | -1.3829 | 0.4234 | -3.2658 | 0.0010914 | 0.0267642 | no |
| Sobic.006G132700 | 39.6794 | 2.2433 | 0.6872 | 3.2646 | 0.0010963 | 0.0268511 | up |
| Sobic.003G172966 | 2427.9658 | -1.2951 | 0.3970 | -3.2624 | 0.0011046 | 0.0269939 | no |
| Sobic.003G348800 | 1469.4463 | 1.2905 | 0.3955 | 3.2625 | 0.0011043 | 0.0269939 | no |
| Sobic.004G208700 | 1467.1745 | 2.4052 | 0.7378 | 3.2597 | 0.0011152 | 0.0271896 | up |
| Sobic.001G101200 | 15.9704 | 3.0011 | 0.9206 | 3.2599 | 0.0011146 | 0.0271896 | up |
| Sobic.006G030700 | 393.3469 | 1.6349 | 0.5017 | 3.2590 | 0.0011183 | 0.0272318 | no |
| Sobic.007G207800 | 6732.6265 | -1.3743 | 0.4219 | -3.2571 | 0.0011256 | 0.0273796 | no |
| Sobic.003G241900 | 4447.2643 | -1.9006 | 0.5837 | -3.2563 | 0.0011288 | 0.0273947 | no |
| Sobic.007G128100 | 132.8172 | 1.5224 | 0.4675 | 3.2563 | 0.0011287 | 0.0273947 | no |
| Sobic.006G101900 | 140.1959 | -1.5935 | 0.4896 | -3.2546 | 0.0011355 | 0.0275251 | no |
| Sobic.006G258700 | 11446.5507 | -1.1811 | 0.3631 | -3.2532 | 0.0011411 | 0.0275966 | no |
| Sobic.001G464600 | 42.8064 | 2.0393 | 0.6268 | 3.2535 | 0.00114 | 0.0275966 | up |
| Sobic.001G272700 | 113.1451 | 2.3048 | 0.7087 | 3.2523 | 0.0011446 | 0.0276501 | up |
| Sobic.009G242100 | 18403.8194 | -1.0868 | 0.3342 | -3.2519 | 0.0011464 | 0.0276625 | no |
| Sobic.007G204400 | 309.4839 | -1.5476 | 0.4760 | -3.2514 | 0.0011482 | 0.0276745 | no |
| Sobic.001G088900 | 346.3373 | 1.0203 | 0.3141 | 3.2483 | 0.0011608 | 0.0279139 | no |
| Sobic.006G186300 | 10.1234 | 3.9598 | 1.2190 | 3.2484 | 0.0011605 | 0.0279139 | up |
| Sobic.001G366600 | 79.4187 | 1.6673 | 0.5133 | 3.2480 | 0.0011622 | 0.0279164 | no |
| Sobic.004G001300 | 40848.8340 | -1.0540 | 0.3247 | -3.2462 | 0.0011696 | 0.0280304 | no |
| Sobic.004G268300 | 1000.4804 | -0.8213 | 0.2530 | -3.2463 | 0.0011693 | 0.0280304 | no |
| Sobic.004G279150 | 437.1191 | 1.7973 | 0.5539 | 3.2445 | 0.0011765 | 0.0281621 | no |
| Sobic.002G030200 | ######### | -1.9565 | 0.6036 | -3.2411 | 0.0011906 | 0.0284683 | no |
| Sobic.006G036300 | 3008.6385 | -2.6400 | 0.8149 | -3.2395 | 0.0011975 | 0.0285668 | down |
| Sobic.010G267200 | 83.8408 | 2.4295 | 0.7499 | 3.2397 | 0.0011965 | 0.0285668 | up |
| Sobic.010G176600 | 222.3986 | 0.9024 | 0.2787 | 3.2381 | 0.0012034 | 0.0286769 | no |
| Sobic.010G236700 | 92.8913 | -2.4562 | 0.7589 | -3.2365 | 0.00121 | 0.0288015 | down |
| Sobic.003G303700 | 5317.9212 | -1.4341 | 0.4434 | -3.2343 | 0.0012195 | 0.0289934 | no |
| Sobic.005G101500 | 53.2373 | 3.4983 | 1.0824 | 3.2321 | 0.0012289 | 0.0291841 | up |
| Sobic.002G148700 | 1885.5115 | 1.0682 | 0.3306 | 3.2311 | 0.0012334 | 0.0292251 | no |
| Sobic.002G353900 | 325.4066 | 1.6435 | 0.5086 | 3.2312 | 0.0012328 | 0.0292251 | no |
| Sobic.002G119500 | 45503.8370 | -1.6877 | 0.5225 | -3.2303 | 0.0012366 | 0.0292701 | no |
| Sobic.010G177100 | 246.8679 | 1.8720 | 0.5796 | 3.2298 | 0.0012388 | 0.0292874 | no |
| Sobic.007G225600 | 907.4555 | -1.0481 | 0.3246 | -3.2286 | 0.0012441 | 0.029348 | no |
| Sobic.007G158600 | 54.3205 | 2.2715 | 0.7036 | 3.2286 | 0.0012441 | 0.029348 | up |
| Sobic.003G363100 | 6074.3114 | 0.7440 | 0.2306 | 3.2267 | 0.0012524 | 0.0295096 | no |
| Sobic.004G202400 | 56.4069 | 2.8920 | 0.8967 | 3.2253 | 0.0012583 | 0.0296166 | up |
| Sobic.010G175100 | 17.0566 | -2.6152 | 0.8109 | -3.2248 | 0.0012605 | 0.0296352 | down |
| Sobic.003G424400 | 343.3684 | 1.5432 | 0.4787 | 3.2239 | 0.0012644 | 0.0296948 | no |
| Sobic.003G203000 | 17662.0050 | -1.0621 | 0.3296 | -3.2228 | 0.0012696 | 0.0297492 | no |
| Sobic.002G312700 | 289.7054 | 0.9992 | 0.3101 | 3.2228 | 0.0012696 | 0.0297492 | no |
| Sobic.004G357300 | 1535.1927 | -1.4634 | 0.4543 | -3.2210 | 0.0012775 | 0.0298638 | no |
| Sobic.003G185900 | 12693.8110 | -1.2622 | 0.3919 | -3.2204 | 0.0012801 | 0.0298638 | no |
| Sobic.002G076932 | 131.9609 | 1.5053 | 0.4674 | 3.2209 | 0.0012781 | 0.0298638 | no |
| Sobic.002G214200 | 84.6305 | 1.7376 | 0.5395 | 3.2207 | 0.0012789 | 0.0298638 | no |
| Sobic.007G052400 | 12692.5971 | -1.4236 | 0.4422 | -3.2192 | 0.0012855 | 0.0299224 | no |
| Sobic.003G118000 | 317.1461 | 1.2867 | 0.3997 | 3.2193 | 0.0012849 | 0.0299224 | no |
| Sobic.003G286600 | 75.2708 | 2.9262 | 0.9092 | 3.2184 | 0.0012889 | 0.0299514 | up |

| Sobic.010G117100 | 49.6405 | 3.1844 | 0.9895 | 3.2183 | 0.0012896 | 0.0299514 | up |
| --- | --- | --- | --- | --- | --- | --- | --- |
| Sobic.007G077100 | 292.6236 | -3.0374 | 0.9443 | -3.2166 | 0.0012972 | 0.0300949 | down |
| Sobic.004G276600 | 29.1819 | -1.8948 | 0.5892 | -3.2157 | 0.0013014 | 0.0301602 | no |
| Sobic.009G146800 | 1083.7951 | -1.1115 | 0.3457 | -3.2152 | 0.0013038 | 0.030181 | no |
| Sobic.006G154300 | 9.4888 | 3.9071 | 1.2161 | 3.2129 | 0.0013141 | 0.0303861 | up |
| Sobic.002G249600 | 57.4147 | 2.4115 | 0.7509 | 3.2113 | 0.0013212 | 0.0305189 | up |
| Sobic.003G000600 | 895.1985 | 1.2371 | 0.3854 | 3.2098 | 0.0013282 | 0.0306452 | no |
| Sobic.007G151300 | 7.7243 | 6.1639 | 1.9211 | 3.2085 | 0.0013341 | 0.030748 | up |
| Sobic.008G188000 | 39.0195 | 1.9705 | 0.6142 | 3.2082 | 0.0013358 | 0.0307545 | no |
| Sobic.006G211500 | 172.1100 | 1.0481 | 0.3269 | 3.2064 | 0.0013439 | 0.0309075 | no |
| Sobic.006G025900 | 1679.3397 | -4.8700 | 1.5197 | -3.2047 | 0.0013522 | 0.0310633 | down |
| Sobic.003G052500 | 91312.8786 | -1.6964 | 0.5296 | -3.2031 | 0.0013595 | 0.0311982 | no |
| Sobic.003G107100 | 5897.8068 | -1.0071 | 0.3145 | -3.2019 | 0.0013651 | 0.0312913 | no |
| Sobic.010G163900 | 1284.1762 | -1.2310 | 0.3846 | -3.2004 | 0.0013726 | 0.0314099 | no |
| Sobic.010G029000 | 99.9674 | 1.1407 | 0.3564 | 3.2002 | 0.0013732 | 0.0314099 | no |
| Sobic.010G242300 | 234.9142 | 0.9592 | 0.2999 | 3.1985 | 0.0013813 | 0.0315593 | no |
| Sobic.004G047900 | 35.5709 | 3.5418 | 1.1078 | 3.1970 | 0.0013885 | 0.031691 | up |
| Sobic.007G052900 | 2031.9968 | -1.4221 | 0.4449 | -3.1964 | 0.0013915 | 0.0317245 | no |
| Sobic.001G062600 | 24.4719 | 3.6328 | 1.1367 | 3.1959 | 0.0013941 | 0.0317502 | up |
| Sobic.006G241350 | 260.4503 | -1.7008 | 0.5324 | -3.1945 | 0.0014009 | 0.0318711 | no |
| Sobic.010G235500 | 55.3038 | 1.5512 | 0.4859 | 3.1927 | 0.0014094 | 0.0320298 | no |
| Sobic.002G201200 | 24.7132 | -2.9855 | 0.9355 | -3.1914 | 0.0014157 | 0.0321109 | down |
| Sobic.010G246600 | 1894.3802 | -2.0769 | 0.6509 | -3.1910 | 0.0014176 | 0.0321109 | down |
| Sobic.001G353300 | 365.3193 | 2.4968 | 0.7824 | 3.1911 | 0.0014175 | 0.0321109 | up |
| Sobic.001G436000 | 2819.3365 | -0.8758 | 0.2746 | -3.1889 | 0.0014281 | 0.0323154 | no |
| Sobic.010G223100 | 364.9399 | 1.6307 | 0.5116 | 3.1877 | 0.0014339 | 0.0324118 | no |
| Sobic.001G046900 | 96910.1715 | -1.7764 | 0.5576 | -3.1858 | 0.0014437 | 0.0325966 | no |
| Sobic.010G120300 | 265.8361 | 0.8662 | 0.2719 | 3.1853 | 0.001446 | 0.0326138 | no |
| Sobic.010G176700 | 18.8587 | 2.2598 | 0.7096 | 3.1848 | 0.0014486 | 0.0326375 | up |
| Sobic.004G236600 | 204.7251 | 1.7705 | 0.5560 | 3.1844 | 0.0014506 | 0.0326491 | no |
| Sobic.007G002000 | 3694.8904 | -1.0327 | 0.3243 | -3.1840 | 0.0014527 | 0.0326547 | no |
| Sobic.003G195700 | 102.5877 | 2.1025 | 0.6604 | 3.1837 | 0.001454 | 0.0326547 | up |
| Sobic.005G116100 | 554.1342 | -1.3961 | 0.4388 | -3.1814 | 0.0014655 | 0.0328793 | no |
| Sobic.001G523300 | 486.2277 | 1.1646 | 0.3663 | 3.1792 | 0.0014767 | 0.0330956 | no |
| Sobic.004G218700 | 118.6411 | 2.0790 | 0.6541 | 3.1783 | 0.0014815 | 0.0331665 | up |
| Sobic.006G103400 | 5645.7064 | -1.3645 | 0.4294 | -3.1778 | 0.0014839 | 0.0331857 | no |
| Sobic.001G364100 | 3703.8564 | -1.3104 | 0.4124 | -3.1773 | 0.0014866 | 0.0332097 | no |
| Sobic.002G405100 | 11981.1017 | -1.3632 | 0.4291 | -3.1769 | 0.0014885 | 0.0332172 | no |
| Sobic.007G213200 | 24.5209 | -4.2208 | 1.3289 | -3.1760 | 0.001493 | 0.0332482 | down |
| Sobic.004G341100 | 3799.2880 | 1.3151 | 0.4140 | 3.1763 | 0.0014918 | 0.0332482 | no |
| Sobic.003G199200 | 120.6550 | 1.2979 | 0.4089 | 3.1745 | 0.0015011 | 0.0333922 | no |
| Sobic.009G224200 | 37.1810 | -4.4918 | 1.4153 | -3.1736 | 0.0015054 | 0.0334535 | down |
| Sobic.005G075800 | 48.2615 | 2.0622 | 0.6499 | 3.1733 | 0.001507 | 0.0334535 | up |
| Sobic.001G312500 | 412.6898 | -1.0222 | 0.3222 | -3.1724 | 0.0015117 | 0.0335239 | no |
| Sobic.006G180432 | 152.7547 | -1.4332 | 0.4519 | -3.1716 | 0.0015163 | 0.0335536 | no |
| Sobic.002G102200 | 718.0884 | -1.1868 | 0.3742 | -3.1718 | 0.001515 | 0.0335536 | no |
| Sobic.001G070500 | ######### | -1.6667 | 0.5256 | -3.1711 | 0.0015184 | 0.0335652 | no |
| Sobic.003G287400 | 113.9822 | 1.1697 | 0.3689 | 3.1705 | 0.001522 | 0.0336091 | no |
| Sobic.003G059300 | 549.5544 | 1.5600 | 0.4924 | 3.1680 | 0.0015351 | 0.0338639 | no |
| Sobic.006G118000 | 851.1692 | -1.7710 | 0.5592 | -3.1669 | 0.0015409 | 0.0339569 | no |
| Sobic.008G172800 | 1832.7788 | -1.1460 | 0.3620 | -3.1662 | 0.0015446 | 0.0340016 | no |
| Sobic.003G314500 | 20725.6679 | -1.2782 | 0.4039 | -3.1645 | 0.0015533 | 0.0340874 | no |
| Sobic.004G272600 | 5814.9438 | -1.1385 | 0.3597 | -3.1646 | 0.0015527 | 0.0340874 | no |
| Sobic.004G257800 | 72.9296 | 1.9908 | 0.6291 | 3.1647 | 0.0015527 | 0.0340874 | no |
| Sobic.002G376000 | 1734.3403 | -1.5512 | 0.4903 | -3.1639 | 0.0015564 | 0.0340896 | no |
| Sobic.008G038200 | 587.2231 | 0.8025 | 0.2536 | 3.1639 | 0.0015567 | 0.0340896 | no |
| Sobic.008G133200 | 33.9038 | -2.5442 | 0.8043 | -3.1630 | 0.0015613 | 0.0341138 | down |

| Sobic.002G350901 | 234.4074 | 1.1835 | 0.3742 | 3.1631 | 0.0015611 | 0.0341138 | no |
| --- | --- | --- | --- | --- | --- | --- | --- |
| Sobic.005G055300 | 222.3909 | 2.2932 | 0.7251 | 3.1628 | 0.0015628 | 0.0341138 | up |
| Sobic.006G190100 | 202.5931 | 2.9668 | 0.9381 | 3.1625 | 0.0015642 | 0.0341138 | up |
| Sobic.010G241000 | ######### | -1.6101 | 0.5093 | -3.1612 | 0.001571 | 0.0341904 | no |
| Sobic.002G206300 | 1315.9908 | -1.2023 | 0.3803 | -3.1614 | 0.0015703 | 0.0341904 | no |
| Sobic.004G052500 | 49.6268 | 3.5869 | 1.1352 | 3.1596 | 0.0015801 | 0.0343528 | up |
| Sobic.010G163150 | 491.7585 | -1.8368 | 0.5816 | -3.1582 | 0.0015876 | 0.0344094 | no |
| Sobic.001G074200 | 681.0578 | 1.0878 | 0.3444 | 3.1586 | 0.0015854 | 0.0344094 | no |
| Sobic.001G001200 | 109.8536 | 2.2280 | 0.7055 | 3.1579 | 0.0015892 | 0.0344094 | up |
| Sobic.009G207800 | 13.9073 | 3.5232 | 1.1155 | 3.1584 | 0.0015866 | 0.0344094 | up |
| Sobic.004G024600 | 1968.2972 | -1.2588 | 0.3989 | -3.1558 | 0.0016008 | 0.0345896 | no |
| Sobic.003G014300 | 1605.8313 | -1.0665 | 0.3379 | -3.1559 | 0.0016003 | 0.0345896 | no |
| Sobic.007G213700 | 40330.9904 | -0.9695 | 0.3074 | -3.1538 | 0.0016114 | 0.034782 | no |
| Sobic.001G450600 | 4142.4644 | -0.9399 | 0.2983 | -3.1513 | 0.0016256 | 0.0350531 | no |
| Sobic.006G116050 | 3966.1382 | -1.0608 | 0.3368 | -3.1500 | 0.0016326 | 0.0351481 | no |
| Sobic.007G182500 | 186.2162 | 1.2218 | 0.3879 | 3.1499 | 0.0016333 | 0.0351481 | no |
| Sobic.002G184600 | 2440.3437 | -1.2633 | 0.4012 | -3.1489 | 0.0016388 | 0.035194 | no |
| Sobic.007G120000 | 31.4763 | 3.7132 | 1.1791 | 3.1491 | 0.0016376 | 0.035194 | up |
| Sobic.008G134800 | 4086.4772 | -1.6140 | 0.5129 | -3.1470 | 0.0016493 | 0.0352343 | no |
| Sobic.005G118400 | 3781.1904 | -1.2010 | 0.3816 | -3.1473 | 0.0016481 | 0.0352343 | no |
| Sobic.001G441000 | 1849.8772 | 1.0417 | 0.3310 | 3.1468 | 0.0016507 | 0.0352343 | no |
| Sobic.009G195000 | 2150.0843 | 1.1560 | 0.3673 | 3.1471 | 0.0016488 | 0.0352343 | no |
| Sobic.007G060000 | 214.6616 | 1.6653 | 0.5291 | 3.1472 | 0.0016487 | 0.0352343 | no |
| Sobic.007G214300 | 454.0147 | 1.7300 | 0.5495 | 3.1481 | 0.0016431 | 0.0352343 | no |
| Sobic.001G389000 | 40.1616 | 2.3051 | 0.7328 | 3.1458 | 0.0016561 | 0.0353136 | up |
| Sobic.002G401100 | 1867.1460 | -1.2146 | 0.3863 | -3.1444 | 0.0016642 | 0.0354155 | no |
| Sobic.004G186300 | 190.8025 | 1.1755 | 0.3738 | 3.1446 | 0.001663 | 0.0354155 | no |
| Sobic.009G151000 | 306.2282 | -1.5658 | 0.4986 | -3.1406 | 0.0016862 | 0.0357673 | no |
| Sobic.004G227200 | 21539.2530 | -1.2587 | 0.4008 | -3.1403 | 0.0016879 | 0.0357673 | no |
| Sobic.010G254200 | 847.2100 | -1.2134 | 0.3864 | -3.1403 | 0.0016879 | 0.0357673 | no |
| Sobic.003G260100 | 908.1038 | -0.9945 | 0.3167 | -3.1403 | 0.001688 | 0.0357673 | no |
| Sobic.010G162400 | 337.1347 | 1.2057 | 0.3840 | 3.1400 | 0.0016892 | 0.0357673 | no |
| Sobic.006G140000 | 186.1297 | 1.4776 | 0.4707 | 3.1392 | 0.0016943 | 0.0358377 | no |
| Sobic.009G206400 | 312.7942 | -1.8623 | 0.5933 | -3.1387 | 0.001697 | 0.0358598 | no |
| Sobic.006G174500 | 31.7957 | 3.4825 | 1.1097 | 3.1383 | 0.0016993 | 0.0358725 | up |
| Sobic.009G234800 | 3711.4208 | 1.1428 | 0.3642 | 3.1375 | 0.0017039 | 0.035933 | no |
| Sobic.010G201600 | 1874.1247 | -1.6501 | 0.5262 | -3.1361 | 0.0017118 | 0.0359897 | no |
| Sobic.010G079000 | 25793.0946 | -1.4878 | 0.4745 | -3.1356 | 0.0017151 | 0.0359897 | no |
| Sobic.006G109100 | 488.3678 | -1.3766 | 0.4389 | -3.1367 | 0.0017084 | 0.0359897 | no |
| Sobic.004G219900 | 3373.4876 | -0.9337 | 0.2977 | -3.1365 | 0.00171 | 0.0359897 | no |
| Sobic.010G035900 | 19.0266 | 2.1993 | 0.7014 | 3.1357 | 0.0017144 | 0.0359897 | up |
| Sobic.003G081500 | 61.1340 | 2.3709 | 0.7562 | 3.1352 | 0.0017175 | 0.0360052 | up |
| Sobic.001G140101 | 560.4628 | -0.8910 | 0.2842 | -3.1348 | 0.0017195 | 0.0360109 | no |
| Sobic.001G222700 | 596.9412 | 2.2752 | 0.7260 | 3.1337 | 0.0017261 | 0.0361121 | up |
| Sobic.009G240700 | 6714.2034 | 0.8627 | 0.2753 | 3.1333 | 0.0017286 | 0.0361297 | no |
| Sobic.003G435100 | 878.8015 | -2.0885 | 0.6669 | -3.1318 | 0.0017375 | 0.0362309 | down |
| Sobic.003G332500 | 1072.2910 | 0.7936 | 0.2534 | 3.1316 | 0.0017386 | 0.0362309 | no |
| Sobic.001G066000 | 329.1546 | 2.0007 | 0.6388 | 3.1318 | 0.0017376 | 0.0362309 | up |
| Sobic.001G410600 | 268.9549 | -0.9947 | 0.3177 | -3.1307 | 0.0017438 | 0.0363041 | no |
| Sobic.002G127200 | 1347.2329 | -1.1776 | 0.3762 | -3.1302 | 0.001747 | 0.0363343 | no |
| Sobic.004G025700 | 464.8820 | 2.1205 | 0.6776 | 3.1296 | 0.0017505 | 0.0363343 | up |
| Sobic.002G135800 | 8.4510 | 5.3040 | 1.6947 | 3.1299 | 0.0017489 | 0.0363343 | up |
| Sobic.005G019800 | 60.2565 | 1.6449 | 0.5257 | 3.1290 | 0.0017538 | 0.036367 | no |
| Sobic.001G368900 | 452.0411 | -2.7589 | 0.8819 | -3.1286 | 0.0017565 | 0.0363888 | down |
| Sobic.006G189500 | 8.9586 | -4.3176 | 1.3802 | -3.1282 | 0.001759 | 0.036404 | down |
| Sobic.010G265500 | 6486.5251 | -1.2943 | 0.4139 | -3.1272 | 0.0017645 | 0.0364821 | no |
| Sobic.002G342500 | 1541.4153 | 0.9048 | 0.2894 | 3.1263 | 0.0017701 | 0.0365257 | no |

| Sobic.010G148501 | 59.6373 | 1.8360 | 0.5872 | 3.1265 | 0.0017692 | 0.0365257 | no |
| --- | --- | --- | --- | --- | --- | --- | --- |
| Sobic.003G288500 | 137.7034 | 2.1305 | 0.6817 | 3.1251 | 0.0017772 | 0.0366361 | up |
| Sobic.003G284300 | 5278.9933 | -1.9822 | 0.6345 | -3.1241 | 0.0017832 | 0.0367247 | no |
| Sobic.003G122900 | 1658.9318 | -1.0967 | 0.3512 | -3.1231 | 0.0017896 | 0.0368203 | no |
| Sobic.007G112832 | 13.2480 | -2.6529 | 0.8498 | -3.1219 | 0.001797 | 0.0369001 | down |
| Sobic.004G240900 | 437.0074 | -2.4912 | 0.7979 | -3.1221 | 0.0017959 | 0.0369001 | down |
| Sobic.008G077800 | 82.5338 | -2.0582 | 0.6598 | -3.1194 | 0.0018122 | 0.0369206 | down |
| Sobic.001G181300 | 40.2932 | -1.8363 | 0.5884 | -3.1208 | 0.0018035 | 0.0369206 | no |
| Sobic.001G339500 | 5849.1659 | -1.1716 | 0.3756 | -3.1192 | 0.0018137 | 0.0369206 | no |
| Sobic.003G146500 | 2440.4148 | -1.1661 | 0.3738 | -3.1194 | 0.001812 | 0.0369206 | no |
| Sobic.001G426600 | 1281.5878 | 0.7770 | 0.2490 | 3.1202 | 0.0018072 | 0.0369206 | no |
| Sobic.003G413600 | 126.4020 | 1.2816 | 0.4106 | 3.1212 | 0.0018012 | 0.0369206 | no |
| Sobic.007G075100 | 149.5530 | 1.6609 | 0.5325 | 3.1192 | 0.0018133 | 0.0369206 | no |
| Sobic.007G082200 | 62.7991 | 2.1744 | 0.6967 | 3.1210 | 0.0018021 | 0.0369206 | up |
| Sobic.001G104100 | 40.2251 | 3.7636 | 1.2062 | 3.1203 | 0.0018069 | 0.0369206 | up |
| Sobic.001G208000 | 1796.2428 | 1.6187 | 0.5191 | 3.1183 | 0.0018192 | 0.0369964 | no |
| Sobic.006G239300 | 259.4627 | 1.0617 | 0.3406 | 3.1168 | 0.0018285 | 0.0371301 | no |
| Sobic.002G308700 | 60.7785 | 3.2027 | 1.0276 | 3.1166 | 0.0018293 | 0.0371301 | up |
| Sobic.006G073500 | 83118.4778 | -2.3536 | 0.7553 | -3.1163 | 0.0018314 | 0.0371361 | down |
| Sobic.002G030650 | 12.8145 | -7.3664 | 2.3649 | -3.1149 | 0.0018398 | 0.0371966 | down |
| Sobic.001G098800 | 17524.8839 | -1.1260 | 0.3615 | -3.1147 | 0.0018414 | 0.0371966 | no |
| Sobic.002G324700 | 1803.7698 | -0.9209 | 0.2956 | -3.1154 | 0.0018369 | 0.0371966 | no |
| Sobic.007G218900 | 98.8963 | 1.5501 | 0.4976 | 3.1150 | 0.0018397 | 0.0371966 | no |
| Sobic.001G254400 | 257.5570 | -0.9137 | 0.2935 | -3.1133 | 0.0018501 | 0.0373365 | no |
| Sobic.003G349200 | 5251.6012 | -1.2012 | 0.3859 | -3.1126 | 0.0018544 | 0.0373884 | no |
| Sobic.007G131900 | 865.7165 | -1.0995 | 0.3534 | -3.1113 | 0.0018624 | 0.0374781 | no |
| Sobic.001G054400 | 7.9086 | 6.1976 | 1.9919 | 3.1113 | 0.0018624 | 0.0374781 | up |
| Sobic.002G074500 | 45.5184 | 2.5487 | 0.8193 | 3.1107 | 0.0018662 | 0.0375177 | up |
| Sobic.007G119800 | 2004.3750 | 1.0544 | 0.3391 | 3.1096 | 0.0018732 | 0.037623 | no |
| Sobic.001G163600 | 964.7924 | -1.2884 | 0.4144 | -3.1090 | 0.0018769 | 0.037662 | no |
| Sobic.010G063433 | 36.2401 | -3.8535 | 1.2396 | -3.1087 | 0.0018792 | 0.0376709 | down |
| Sobic.008G045900 | 216.2088 | -1.8339 | 0.5900 | -3.1083 | 0.0018816 | 0.0376831 | no |
| Sobic.006G018800 | 422.7050 | 1.0275 | 0.3310 | 3.1044 | 0.0019068 | 0.0381529 | no |
| Sobic.002G030300 | 161.0825 | -3.4582 | 1.1149 | -3.1018 | 0.0019232 | 0.0384053 | down |
| Sobic.006G093100 | 91691.0420 | -1.5840 | 0.5107 | -3.1016 | 0.0019249 | 0.0384053 | no |
| Sobic.007G140300 | 147.2597 | 1.0388 | 0.3349 | 3.1020 | 0.001922 | 0.0384053 | no |
| Sobic.003G001650 | 147.5694 | 2.6747 | 0.8626 | 3.1007 | 0.0019304 | 0.0384781 | up |
| Sobic.010G086400 | 215.5292 | 0.9147 | 0.2951 | 3.1002 | 0.0019338 | 0.0385088 | no |
| Sobic.007G212400 | 2373.3870 | -1.7979 | 0.5800 | -3.0998 | 0.0019365 | 0.0385263 | no |
| Sobic.007G201900 | 29.4889 | 1.7993 | 0.5808 | 3.0982 | 0.0019473 | 0.0387053 | no |
| Sobic.009G178850 | 23.9949 | 2.2438 | 0.7244 | 3.0975 | 0.0019519 | 0.0387603 | up |
| Sobic.008G032300 | 1103.5338 | -0.9991 | 0.3227 | -3.0957 | 0.0019632 | 0.038949 | no |
| Sobic.002G291900 | 324.5272 | -2.2834 | 0.7377 | -3.0952 | 0.0019667 | 0.0389586 | down |
| Sobic.007G029300 | 520.4514 | -2.1705 | 0.7013 | -3.0951 | 0.0019674 | 0.0389586 | down |
| Sobic.003G249600 | 668.7795 | 0.8719 | 0.2817 | 3.0948 | 0.0019697 | 0.0389663 | no |
| Sobic.002G160600 | 6.2398 | 5.8559 | 1.8928 | 3.0938 | 0.001976 | 0.039056 | up |
| Sobic.009G214300 | 898.2381 | -1.3864 | 0.4483 | -3.0927 | 0.0019837 | 0.0391703 | no |
| Sobic.001G435500 | 21237.2919 | -1.4471 | 0.4680 | -3.0918 | 0.0019892 | 0.0392288 | no |
| Sobic.002G376600 | 1579.6755 | -1.1671 | 0.3775 | -3.0917 | 0.0019904 | 0.0392288 | no |
| Sobic.006G020200 | 133.1851 | 1.2302 | 0.3982 | 3.0894 | 0.0020058 | 0.0394968 | no |
| Sobic.009G121900 | 53.7775 | -1.8077 | 0.5855 | -3.0875 | 0.0020183 | 0.0396313 | no |
| Sobic.008G175700 | 268.0779 | -1.2347 | 0.3999 | -3.0876 | 0.0020175 | 0.0396313 | no |
| Sobic.003G189200 | 15.7079 | 5.4273 | 1.7576 | 3.0879 | 0.0020155 | 0.0396313 | up |
| Sobic.010G258700 | 142.3776 | -1.5304 | 0.4958 | -3.0870 | 0.0020222 | 0.0396705 | no |
| Sobic.004G063400 | 75.1709 | 3.1310 | 1.0144 | 3.0866 | 0.0020244 | 0.0396779 | up |
| Sobic.006G054200 | 48.7332 | 1.5971 | 0.5175 | 3.0859 | 0.0020292 | 0.0397333 | no |
| Sobic.005G166900 | 882.3188 | -1.5560 | 0.5044 | -3.0851 | 0.0020345 | 0.039802 | no |

| Sobic.006G113900 | 982.1138 | 1.1305 | 0.3665 | 3.0846 | 0.0020384 | 0.0398404 | no |
| --- | --- | --- | --- | --- | --- | --- | --- |
| Sobic.004G347500 | 64.1163 | 2.2423 | 0.7273 | 3.0829 | 0.00205 | 0.0400292 | up |
| Sobic.001G082400 | 1262.2439 | 2.3182 | 0.7521 | 3.0823 | 0.0020539 | 0.0400696 | up |
| Sobic.009G189000 | 6143.9560 | -2.0326 | 0.6604 | -3.0779 | 0.0020848 | 0.0406337 | down |
| Sobic.007G116950 | 1881.7623 | -1.7273 | 0.5615 | -3.0764 | 0.002095 | 0.0407961 | no |
| Sobic.004G333900 | 132.8893 | -2.5302 | 0.8227 | -3.0756 | 0.0021006 | 0.0408298 | down |
| Sobic.003G258100 | 61.2874 | 2.4749 | 0.8046 | 3.0757 | 0.0021 | 0.0408298 | up |
| Sobic.004G286600 | 8379.0230 | 1.8614 | 0.6053 | 3.0751 | 0.0021041 | 0.0408595 | no |
| Sobic.004G211501 | 3015.6072 | -1.1336 | 0.3690 | -3.0718 | 0.0021274 | 0.0412742 | no |
| Sobic.006G211300 | 399.1033 | 1.0569 | 0.3442 | 3.0708 | 0.0021347 | 0.0413769 | no |
| Sobic.006G273000 | 4532.5398 | -1.3134 | 0.4278 | -3.0703 | 0.0021384 | 0.0414097 | no |
| Sobic.004G278400 | 19.2852 | 2.6388 | 0.8595 | 3.0700 | 0.0021403 | 0.0414097 | up |
| Sobic.004G298900 | 5456.3341 | -1.1554 | 0.3764 | -3.0694 | 0.0021449 | 0.0414606 | no |
| Sobic.001G233200 | 640.6347 | 1.2300 | 0.4008 | 3.0686 | 0.0021506 | 0.041533 | no |
| Sobic.006G268200 | 2372.1076 | -2.1525 | 0.7017 | -3.0676 | 0.0021582 | 0.0416407 | down |
| Sobic.010G005200 | 509.9791 | -1.1680 | 0.3808 | -3.0673 | 0.0021601 | 0.041641 | no |
| Sobic.002G073250 | 341.9192 | 1.1790 | 0.3845 | 3.0663 | 0.0021676 | 0.0417463 | no |
| Sobic.009G132000 | 1316.9627 | -1.3209 | 0.4310 | -3.0649 | 0.0021772 | 0.0418667 | no |
| Sobic.007G198000 | 251.4719 | 1.8803 | 0.6135 | 3.0647 | 0.0021787 | 0.0418667 | no |
| Sobic.001G471100 | 11.2811 | 4.8169 | 1.5718 | 3.0646 | 0.0021798 | 0.0418667 | up |
| Sobic.001G481400 | 62.3112 | -1.7870 | 0.5839 | -3.0605 | 0.0022098 | 0.042404 | no |
| Sobic.003G389300 | 558.3578 | 1.4579 | 0.4765 | 3.0596 | 0.0022165 | 0.0424935 | no |
| Sobic.005G184100 | 465.4384 | -0.9279 | 0.3033 | -3.0592 | 0.0022193 | 0.0425085 | no |
| Sobic.007G071500 | ######### | -1.6503 | 0.5396 | -3.0583 | 0.0022256 | 0.0425918 | no |
| Sobic.007G205900 | 439.9856 | 1.0188 | 0.3332 | 3.0574 | 0.0022329 | 0.0426533 | no |
| Sobic.004G054300 | 432.2419 | 1.2733 | 0.4165 | 3.0574 | 0.0022326 | 0.0426533 | no |
| Sobic.002G318000 | 811.5675 | -1.0521 | 0.3442 | -3.0568 | 0.0022369 | 0.0426916 | no |
| Sobic.005G042900 | 140.9399 | -1.4620 | 0.4784 | -3.0557 | 0.0022455 | 0.0427941 | no |
| Sobic.007G076900 | 1318.8001 | -1.1716 | 0.3835 | -3.0548 | 0.0022524 | 0.0427941 | no |
| Sobic.007G013601 | 387.2425 | 0.8113 | 0.2656 | 3.0548 | 0.0022524 | 0.0427941 | no |
| Sobic.009G009900 | 23.5968 | 3.9777 | 1.3021 | 3.0549 | 0.0022516 | 0.0427941 | up |
| Sobic.009G144600 | 10.5285 | 4.6428 | 1.5198 | 3.0548 | 0.0022519 | 0.0427941 | up |
| Sobic.007G212200 | 5145.0911 | -1.1795 | 0.3864 | -3.0525 | 0.0022698 | 0.0430856 | no |
| Sobic.009G136400 | 492.9824 | 1.3123 | 0.4300 | 3.0521 | 0.0022722 | 0.0430922 | no |
| Sobic.009G000200 | 1567.9636 | 1.1842 | 0.3880 | 3.0516 | 0.002276 | 0.0431252 | no |
| Sobic.003G230400 | 6652.4315 | -1.4477 | 0.4745 | -3.0514 | 0.0022781 | 0.0431264 | no |
| Sobic.001G291200 | 5829.6067 | -1.7322 | 0.5682 | -3.0484 | 0.0023004 | 0.0434059 | no |
| Sobic.007G170400 | 1075.5841 | -1.3981 | 0.4586 | -3.0485 | 0.0022998 | 0.0434059 | no |
| Sobic.009G213400 | 1569.3849 | -1.3398 | 0.4395 | -3.0483 | 0.0023011 | 0.0434059 | no |
| Sobic.006G087900 | 146.8439 | 1.7058 | 0.5595 | 3.0490 | 0.0022957 | 0.0434059 | no |
| Sobic.004G312200 | 63.1525 | 2.6294 | 0.8629 | 3.0471 | 0.002311 | 0.0435534 | up |
| Sobic.001G060401 | 165.1280 | -1.5821 | 0.5194 | -3.0461 | 0.0023186 | 0.0436559 | no |
| Sobic.002G395200 | 3343.6676 | -1.4275 | 0.4687 | -3.0458 | 0.0023206 | 0.0436559 | no |
| Sobic.001G023700 | 116.2944 | -2.5605 | 0.8412 | -3.0439 | 0.0023357 | 0.0438958 | down |
| Sobic.005G110550 | 87.6495 | 1.4990 | 0.4925 | 3.0436 | 0.0023375 | 0.0438958 | no |
| Sobic.008G149400 | 6.5793 | -4.4776 | 1.4715 | -3.0428 | 0.0023437 | 0.0439736 | down |
| Sobic.003G440600 | 142.8770 | 1.2687 | 0.4170 | 3.0423 | 0.0023481 | 0.0440161 | no |
| Sobic.001G512800 | 2791.2133 | 0.8990 | 0.2956 | 3.0414 | 0.0023548 | 0.044064 | no |
| Sobic.010G051200 | 341.2393 | 1.1203 | 0.3683 | 3.0416 | 0.002353 | 0.044064 | no |
| Sobic.005G218800 | 9.6374 | -6.9560 | 2.2876 | -3.0408 | 0.0023595 | 0.0440743 | down |
| Sobic.009G238300 | 358.6478 | -1.2546 | 0.4126 | -3.0409 | 0.0023586 | 0.0440743 | no |
| Sobic.004G186200 | 825.9206 | -1.0976 | 0.3611 | -3.0393 | 0.002371 | 0.0442502 | no |
| Sobic.002G167800 | 1165.7438 | -2.0847 | 0.6863 | -3.0375 | 0.0023854 | 0.0443268 | down |
| Sobic.003G037500 | 2318.2075 | -1.4967 | 0.4926 | -3.0384 | 0.0023785 | 0.0443268 | no |
| Sobic.004G036900 | 1091.0380 | -1.1881 | 0.3912 | -3.0372 | 0.0023878 | 0.0443268 | no |
| Sobic.002G308000 | 59.8089 | 1.3882 | 0.4570 | 3.0379 | 0.0023825 | 0.0443268 | no |
| Sobic.007G155000 | 181.3722 | 2.2217 | 0.7315 | 3.0372 | 0.0023877 | 0.0443268 | up |

| Sobic.006G007800 | 25.6489 | 2.6048 | 0.8574 | 3.0381 | 0.0023805 | 0.0443268 | up |
| --- | --- | --- | --- | --- | --- | --- | --- |
| Sobic.006G185150 | 11.4144 | 5.7443 | 1.8918 | 3.0365 | 0.0023936 | 0.0443959 | up |
| Sobic.006G107600 | 3696.2322 | -1.5151 | 0.4992 | -3.0351 | 0.0024043 | 0.0445547 | no |
| Sobic.009G068600 | 82.4631 | 1.7281 | 0.5699 | 3.0321 | 0.0024287 | 0.0449674 | no |
| Sobic.001G302666 | 337.7094 | -2.7001 | 0.8907 | -3.0314 | 0.0024345 | 0.045037 | down |
| Sobic.003G328200 | 2840.6465 | -1.3011 | 0.4294 | -3.0299 | 0.0024467 | 0.0452228 | no |
| Sobic.009G137300 | 211.2426 | -2.0246 | 0.6688 | -3.0272 | 0.0024681 | 0.0455779 | down |
| Sobic.003G004200 | 6755.9563 | -0.8881 | 0.2934 | -3.0267 | 0.0024724 | 0.0456163 | no |
| Sobic.003G105800 | 52.8056 | 2.8059 | 0.9272 | 3.0261 | 0.0024774 | 0.0456693 | up |
| Sobic.001G069000 | 2118.5541 | -1.0604 | 0.3505 | -3.0252 | 0.002485 | 0.0457235 | no |
| Sobic.001G118300 | 443.9041 | 1.0056 | 0.3324 | 3.0249 | 0.0024868 | 0.0457235 | no |
| Sobic.006G127200 | 69.6925 | 1.1063 | 0.3657 | 3.0252 | 0.002485 | 0.0457235 | no |
| Sobic.002G407400 | 38.3756 | -2.0814 | 0.6886 | -3.0229 | 0.0025038 | 0.0459948 | down |
| Sobic.003G184100 | 376.9622 | -2.0726 | 0.6862 | -3.0203 | 0.0025252 | 0.0461684 | down |
| Sobic.009G229700 | ######### | -1.9074 | 0.6315 | -3.0203 | 0.0025253 | 0.0461684 | no |
| Sobic.007G016300 | 359.4296 | -1.7460 | 0.5779 | -3.0211 | 0.0025184 | 0.0461684 | no |
| Sobic.009G062400 | 4527.2511 | -1.7427 | 0.5769 | -3.0207 | 0.0025216 | 0.0461684 | no |
| Sobic.001G291300 | 28957.3010 | -1.4650 | 0.4849 | -3.0213 | 0.0025169 | 0.0461684 | no |
| Sobic.006G026200 | 90.2496 | -1.0877 | 0.3602 | -3.0199 | 0.0025285 | 0.0461684 | no |
| Sobic.003G336700 | 162.0403 | 1.3807 | 0.4573 | 3.0194 | 0.0025327 | 0.0461684 | no |
| Sobic.008G191600 | 53.2566 | 1.8901 | 0.6259 | 3.0199 | 0.0025286 | 0.0461684 | no |
| Sobic.002G202700 | 286.7557 | 2.4126 | 0.7990 | 3.0194 | 0.0025329 | 0.0461684 | up |
| Sobic.004G001600 | 29.3963 | 1.6486 | 0.5465 | 3.0168 | 0.0025545 | 0.0465212 | no |
| Sobic.002G013700 | 1207.6907 | -1.2730 | 0.4221 | -3.0162 | 0.0025592 | 0.0465676 | no |
| Sobic.007G032300 | 128.4581 | -1.6040 | 0.5320 | -3.0153 | 0.0025674 | 0.0466598 | no |
| Sobic.003G065400 | 2693.8645 | -0.8525 | 0.2828 | -3.0147 | 0.0025722 | 0.0466598 | no |
| Sobic.005G064100 | 208.5080 | 1.2015 | 0.3986 | 3.0147 | 0.0025721 | 0.0466598 | no |
| Sobic.006G260200 | 81.1956 | 2.5130 | 0.8336 | 3.0146 | 0.0025732 | 0.0466598 | up |
| Sobic.003G290300 | 2361.1153 | -1.1356 | 0.3768 | -3.0136 | 0.0025816 | 0.0467729 | no |
| Sobic.001G191200 | 44566.4669 | -2.4728 | 0.8213 | -3.0108 | 0.0026056 | 0.047087 | down |
| Sobic.006G214400 | 1845.5348 | -1.2319 | 0.4091 | -3.0108 | 0.0026056 | 0.047087 | no |
| Sobic.002G079500 | 276.0828 | -1.1256 | 0.3738 | -3.0112 | 0.0026021 | 0.047087 | no |
| Sobic.010G198800 | 228.3558 | 2.8177 | 0.9360 | 3.0103 | 0.0026102 | 0.0471291 | up |
| Sobic.001G136400 | 38.3663 | 2.8473 | 0.9460 | 3.0097 | 0.0026149 | 0.0471738 | up |
| Sobic.001G009500 | 84.0875 | -2.1277 | 0.7073 | -3.0081 | 0.0026291 | 0.0473885 | down |
| Sobic.009G016633 | 4810.9692 | -0.9501 | 0.3161 | -3.0061 | 0.0026461 | 0.0476547 | no |
| Sobic.006G281000 | 557.7714 | 0.9832 | 0.3272 | 3.0051 | 0.0026553 | 0.0477395 | no |
| Sobic.001G214000 | 29.5798 | 3.2213 | 1.0719 | 3.0052 | 0.0026541 | 0.0477395 | up |
| Sobic.001G146000 | 660.6128 | -0.9170 | 0.3053 | -3.0033 | 0.0026708 | 0.0479774 | no |
| Sobic.002G412100 | 2461.4695 | -1.7558 | 0.5852 | -3.0004 | 0.0026965 | 0.0483971 | no |
| Sobic.001G413600 | 1670.3559 | -1.1460 | 0.3820 | -2.9999 | 0.0027009 | 0.0484356 | no |
| Sobic.003G158600 | 1437.3381 | 1.8462 | 0.6155 | 2.9995 | 0.0027047 | 0.0484611 | no |
| Sobic.004G259800 | 176.8793 | 0.8840 | 0.2948 | 2.9990 | 0.0027084 | 0.0484873 | no |
| Sobic.001G305600 | 7038.3065 | -0.9413 | 0.3140 | -2.9975 | 0.0027216 | 0.0486605 | no |
| Sobic.001G490950 | 179.0773 | 2.4901 | 0.8308 | 2.9974 | 0.0027227 | 0.0486605 | up |
| Sobic.002G354000 | 919.3308 | 1.8390 | 0.6141 | 2.9944 | 0.0027498 | 0.0491022 | no |
| Sobic.001G226401 | 149.2441 | -1.0445 | 0.3489 | -2.9936 | 0.0027575 | 0.0491981 | no |
| Sobic.006G179500 | 85.0000 | 2.1529 | 0.7193 | 2.9929 | 0.002763 | 0.0492545 | up |
| Sobic.002G379700 | 976.9790 | -1.2444 | 0.4159 | -2.9924 | 0.0027676 | 0.0492963 | no |
| Sobic.008G050600 | 21.2641 | 3.1030 | 1.0375 | 2.9907 | 0.0027831 | 0.0495294 | up |
| Sobic.006G093900 | 27890.5266 | -1.2668 | 0.4237 | -2.9900 | 0.0027902 | 0.0496139 | no |
| Sobic.001G047200 | 199.5434 | -0.9800 | 0.3280 | -2.9880 | 0.0028085 | 0.0498975 | no |
| Sobic.008G060200 | 68.1429 | 1.6465 | 0.5515 | 2.9857 | 0.0028294 | 0.0502138 | no |
| Sobic.001G276300 | 2203.0772 | 1.1460 | 0.3839 | 2.9855 | 0.0028311 | 0.0502138 | no |
| Sobic.009G038501 | 30.9675 | 2.3370 | 0.7835 | 2.9828 | 0.0028567 | 0.0506253 | no |
| Sobic.004G274100 | 513.7561 | -1.3986 | 0.4690 | -2.9821 | 0.0028629 | 0.050693 | no |
| Sobic.003G242800 | 524.5730 | 1.0236 | 0.3433 | 2.9817 | 0.0028668 | 0.0507192 | no |

| Sobic.008G147101 | 1926.1544 | -1.3678 | 0.4588 | -2.9813 | 0.0028698 | 0.0507306 | no |
| --- | --- | --- | --- | --- | --- | --- | --- |
| Sobic.001G417200 | 45806.7635 | -1.1919 | 0.3998 | -2.9810 | 0.0028727 | 0.0507398 | no |
| Sobic.009G150900 | 804.0537 | 1.0906 | 0.3661 | 2.9793 | 0.0028891 | 0.0509859 | no |
| Sobic.006G236700 | 209.8878 | 3.1740 | 1.0659 | 2.9776 | 0.0029047 | 0.0512187 | no |
| Sobic.001G247300 | 41.0119 | 1.8840 | 0.6329 | 2.9767 | 0.0029133 | 0.0513265 | no |
| Sobic.001G389300 | 701.3597 | -1.9369 | 0.6508 | -2.9762 | 0.0029184 | 0.0513737 | no |
| Sobic.004G129800 | 40.4479 | 2.3997 | 0.8064 | 2.9758 | 0.0029219 | 0.0513934 | no |
| Sobic.006G105900 | ######### | -1.6996 | 0.5714 | -2.9747 | 0.0029324 | 0.0515341 | no |
| Sobic.007G150200 | 1010.9558 | -1.8892 | 0.6355 | -2.9726 | 0.0029529 | 0.0518518 | no |
| Sobic.003G137100 | 6026.1220 | -1.6278 | 0.5477 | -2.9721 | 0.0029575 | 0.0518671 | no |
| Sobic.003G218801 | 1151.2907 | -1.0695 | 0.3599 | -2.9719 | 0.0029595 | 0.0518671 | no |
| Sobic.010G023200 | 1019.6207 | -2.2034 | 0.7415 | -2.9717 | 0.0029612 | 0.0518671 | no |
| Sobic.009G079300 | 519.8252 | 1.4117 | 0.4751 | 2.9712 | 0.0029662 | 0.0518955 | no |
| Sobic.001G057900 | 18.4554 | 2.4234 | 0.8157 | 2.9710 | 0.0029684 | 0.0518955 | no |
| Sobic.003G444200 | 58.5251 | 1.8928 | 0.6371 | 2.9708 | 0.0029702 | 0.0518955 | no |
| Sobic.002G185600 | 23.5351 | 4.5303 | 1.5253 | 2.9701 | 0.0029775 | 0.0519801 | no |
| Sobic.002G327000 | 564.6662 | 1.2352 | 0.4160 | 2.9692 | 0.0029853 | 0.0520322 | no |
| Sobic.002G156700 | 775.0719 | 2.9296 | 0.9867 | 2.9692 | 0.0029854 | 0.0520322 | no |
| Sobic.003G326000 | 480.6917 | 2.3799 | 0.8016 | 2.9689 | 0.002989 | 0.0520514 | no |
| Sobic.003G228100 | 4367.2801 | 1.0167 | 0.3425 | 2.9686 | 0.0029919 | 0.0520589 | no |
| Sobic.009G201500 | 2770.0170 | 2.2223 | 0.7487 | 2.9682 | 0.0029954 | 0.0520768 | no |
| Sobic.010G054100 | 291.7165 | 0.9808 | 0.3305 | 2.9680 | 0.0029978 | 0.0520768 | no |
| Sobic.001G143600 | 701.3690 | 0.9599 | 0.3235 | 2.9675 | 0.0030024 | 0.0521124 | no |
| Sobic.006G127750 | 16.3874 | -2.3473 | 0.7911 | -2.9672 | 0.0030048 | 0.0521124 | no |
| Sobic.001G443800 | 131.9045 | 1.7957 | 0.6053 | 2.9669 | 0.003008 | 0.0521247 | no |
| Sobic.010G060600 | 140.5666 | 2.3064 | 0.7775 | 2.9664 | 0.0030133 | 0.052142 | no |
| Sobic.003G080100 | 1631.9315 | 1.6853 | 0.5682 | 2.9663 | 0.0030139 | 0.052142 | no |
| Sobic.001G437000 | 262.4529 | 1.3658 | 0.4607 | 2.9650 | 0.0030265 | 0.0523166 | no |
| Sobic.002G036150 | 9.4752 | 3.9763 | 1.3417 | 2.9636 | 0.0030402 | 0.0525093 | no |
| Sobic.006G207800 | 5745.9060 | -1.2594 | 0.4251 | -2.9626 | 0.0030502 | 0.0525993 | no |
| Sobic.003G188000 | 255.5712 | 2.0847 | 0.7037 | 2.9625 | 0.0030511 | 0.0525993 | no |
| Sobic.002G227700 | 4633.6339 | -1.3932 | 0.4703 | -2.9624 | 0.0030529 | 0.0525993 | no |
| Sobic.003G195500 | 2985.5975 | 0.8123 | 0.2743 | 2.9618 | 0.0030584 | 0.052652 | no |
| Sobic.005G022200 | 270.6235 | 1.2562 | 0.4242 | 2.9614 | 0.0030626 | 0.0526812 | no |
| Sobic.009G230900 | 84.0717 | 3.9862 | 1.3462 | 2.9610 | 0.0030664 | 0.052703 | no |
| Sobic.001G516800 | 1691.5309 | -1.2863 | 0.4344 | -2.9607 | 0.0030697 | 0.0527168 | no |
| Sobic.003G149000 | 28.4240 | -2.3608 | 0.7975 | -2.9603 | 0.0030738 | 0.0527453 | no |
| Sobic.009G083300 | 1423.4695 | -0.9091 | 0.3072 | -2.9588 | 0.0030887 | 0.052957 | no |
| Sobic.004G037000 | 296.8256 | 0.7799 | 0.2636 | 2.9584 | 0.0030922 | 0.0529737 | no |
| Sobic.004G075800 | 2408.8779 | -2.1213 | 0.7171 | -2.9581 | 0.0030953 | 0.052985 | no |
| Sobic.009G234600 | ######### | -1.7664 | 0.5972 | -2.9577 | 0.0030996 | 0.0530153 | no |
| Sobic.006G000900 | 330.0469 | 1.1991 | 0.4056 | 2.9565 | 0.0031116 | 0.0531776 | no |
| Sobic.006G174600 | 1214.4087 | 1.0716 | 0.3625 | 2.9559 | 0.0031179 | 0.0532199 | no |
| Sobic.002G316700 | ######### | -1.1672 | 0.3949 | -2.9557 | 0.0031192 | 0.0532199 | no |
| Sobic.003G326800 | 23.2212 | 1.9001 | 0.6429 | 2.9555 | 0.0031217 | 0.0532199 | no |
| Sobic.002G232800 | 281.5617 | 1.4814 | 0.5015 | 2.9541 | 0.0031359 | 0.0534119 | no |
| Sobic.005G033100 | 42.9034 | 2.2750 | 0.7702 | 2.9539 | 0.003138 | 0.0534119 | no |
| Sobic.003G227300 | 209.1672 | 2.2081 | 0.7478 | 2.9527 | 0.00315 | 0.0535728 | no |
| Sobic.009G233300 | 3000.3525 | -0.7164 | 0.2427 | -2.9518 | 0.0031591 | 0.0536848 | no |
| Sobic.006G177900 | 7.9050 | -4.7863 | 1.6225 | -2.9499 | 0.0031792 | 0.0539828 | no |
| Sobic.004G004300 | 2977.1575 | 0.9870 | 0.3347 | 2.9492 | 0.0031856 | 0.0540476 | no |
| Sobic.007G109800 | 109.2566 | 2.2568 | 0.7654 | 2.9487 | 0.0031913 | 0.0540999 | no |
| Sobic.001G401300 | 2121.0144 | 1.9428 | 0.6592 | 2.9471 | 0.003208 | 0.0543401 | no |
| Sobic.003G337400 | 72.6778 | 1.4494 | 0.4925 | 2.9429 | 0.0032514 | 0.0550305 | no |
| Sobic.002G281500 | 557.6008 | -0.8200 | 0.2788 | -2.9414 | 0.003267 | 0.0552459 | no |
| Sobic.010G058200 | 2142.8904 | -1.0186 | 0.3463 | -2.9410 | 0.0032711 | 0.0552459 | no |
| Sobic.006G215000 | 590.8295 | 1.4069 | 0.4784 | 2.9410 | 0.003272 | 0.0552459 | no |

| Sobic.006G113000 | 130.6242 | 1.5710 | 0.5344 | 2.9395 | 0.0032871 | 0.0554577 | no |
| --- | --- | --- | --- | --- | --- | --- | --- |
| Sobic.003G023000 | 1067.3063 | 0.8246 | 0.2806 | 2.9390 | 0.0032924 | 0.0554962 | no |
| Sobic.005G054800 | 9.3298 | 6.4361 | 2.1901 | 2.9387 | 0.0032956 | 0.0554962 | no |
| Sobic.009G015400 | 147.4170 | -1.0433 | 0.3550 | -2.9386 | 0.0032973 | 0.0554962 | no |
| Sobic.002G403600 | 50398.5686 | -0.6556 | 0.2232 | -2.9377 | 0.0033065 | 0.0556073 | no |
| Sobic.002G396600 | 36.9247 | -3.6400 | 1.2394 | -2.9370 | 0.0033143 | 0.0556943 | no |
| Sobic.004G052900 | 942.0080 | 0.9249 | 0.3150 | 2.9364 | 0.0033203 | 0.055749 | no |
| Sobic.001G512500 | 3117.2807 | -1.1747 | 0.4001 | -2.9358 | 0.0033265 | 0.0557865 | no |
| Sobic.009G129300 | 3084.3664 | -1.2091 | 0.4119 | -2.9355 | 0.0033296 | 0.0557865 | no |
| Sobic.009G136500 | 171.4647 | 3.3127 | 1.1285 | 2.9355 | 0.0033304 | 0.0557865 | no |
| Sobic.006G241200 | 91.4537 | -2.7913 | 0.9510 | -2.9350 | 0.0033358 | 0.0558329 | no |
| Sobic.003G392550 | 244.2561 | 1.0856 | 0.3699 | 2.9345 | 0.0033412 | 0.0558519 | no |
| Sobic.007G004500 | 4081.8696 | 1.3798 | 0.4702 | 2.9344 | 0.0033423 | 0.0558519 | no |
| Sobic.010G261400 | 525.6159 | -0.8379 | 0.2856 | -2.9340 | 0.003346 | 0.0558578 | no |
| Sobic.001G186850 | 767.1059 | -0.8200 | 0.2795 | -2.9338 | 0.0033479 | 0.0558578 | no |
| Sobic.003G012800 | 25.8845 | -2.2339 | 0.7616 | -2.9333 | 0.0033542 | 0.0559185 | no |
| Sobic.007G135800 | 15945.6732 | -1.0325 | 0.3521 | -2.9325 | 0.0033621 | 0.0560062 | no |
| Sobic.003G015000 | 505.8149 | -1.3173 | 0.4493 | -2.9322 | 0.0033658 | 0.0560232 | no |
| Sobic.005G072500 | 2827.0724 | -1.3875 | 0.4735 | -2.9304 | 0.0033854 | 0.0562992 | no |
| Sobic.003G386300 | 1902.2949 | -1.3455 | 0.4592 | -2.9302 | 0.0033877 | 0.0562992 | no |
| Sobic.003G310500 | 338.7497 | 1.1999 | 0.4096 | 2.9296 | 0.003394 | 0.0563225 | no |
| Sobic.004G013900 | 57.1160 | -1.6339 | 0.5577 | -2.9296 | 0.0033945 | 0.0563225 | no |
| Sobic.004G320300 | 30.0660 | 1.7213 | 0.5878 | 2.9281 | 0.0034101 | 0.0565376 | no |
| Sobic.001G453300 | 3795.2087 | -1.6227 | 0.5543 | -2.9276 | 0.0034158 | 0.056564 | no |
| Sobic.001G475400 | 1407.6976 | 1.7777 | 0.6072 | 2.9275 | 0.0034171 | 0.056564 | no |
| Sobic.001G112200 | 317.6251 | 2.5542 | 0.8727 | 2.9268 | 0.003425 | 0.056651 | no |
| Sobic.002G210901 | 9.8991 | 3.4965 | 1.1948 | 2.9263 | 0.0034298 | 0.0566816 | no |
| Sobic.001G134800 | 15770.3601 | -1.4410 | 0.4924 | -2.9261 | 0.0034322 | 0.0566816 | no |
| Sobic.010G195000 | 227.1636 | 1.5958 | 0.5456 | 2.9250 | 0.0034441 | 0.0568329 | no |
| Sobic.001G478500 | 1585.7305 | -0.9472 | 0.3239 | -2.9247 | 0.003448 | 0.0568533 | no |
| Sobic.004G211600 | 4672.9282 | -1.1569 | 0.3957 | -2.9241 | 0.0034548 | 0.0568948 | no |
| Sobic.001G510100 | 15.1696 | -3.1366 | 1.0728 | -2.9238 | 0.0034576 | 0.0568948 | no |
| Sobic.008G073900 | 2196.1342 | -1.3971 | 0.4779 | -2.9236 | 0.0034597 | 0.0568948 | no |
| Sobic.002G136300 | 507.0463 | 1.0455 | 0.3576 | 2.9234 | 0.0034627 | 0.0568948 | no |
| Sobic.002G120400 | 615.4011 | -1.7861 | 0.6110 | -2.9232 | 0.003464 | 0.0568948 | no |
| Sobic.007G079200 | 153.2065 | 1.1909 | 0.4074 | 2.9229 | 0.003468 | 0.0569161 | no |
| Sobic.002G367400 | 80.6992 | 1.1841 | 0.4052 | 2.9220 | 0.0034775 | 0.0570268 | no |
| Sobic.010G030900 | 62.7673 | 1.9907 | 0.6815 | 2.9211 | 0.0034885 | 0.0571631 | no |
| Sobic.002G329500 | 793.2336 | -1.2112 | 0.4147 | -2.9204 | 0.0034957 | 0.0572371 | no |
| Sobic.008G119100 | 432.2197 | -1.7341 | 0.5939 | -2.9200 | 0.0035002 | 0.057266 | no |
| Sobic.001G133900 | 1075.2019 | -0.8305 | 0.2844 | -2.9198 | 0.0035031 | 0.0572691 | no |
| Sobic.009G243200 | 1264.5564 | -1.1277 | 0.3863 | -2.9191 | 0.003511 | 0.0573532 | no |
| Sobic.003G080900 | 11.1146 | 3.8110 | 1.3063 | 2.9174 | 0.0035301 | 0.0576214 | no |
| Sobic.004G162400 | 1321.0888 | 2.2879 | 0.7847 | 2.9155 | 0.0035508 | 0.0579137 | no |
| Sobic.004G087800 | 274.4614 | 1.9412 | 0.6659 | 2.9152 | 0.0035541 | 0.0579192 | no |
| Sobic.005G187200 | 846.9962 | -0.8651 | 0.2968 | -2.9150 | 0.0035566 | 0.0579192 | no |
| Sobic.004G249400 | 1669.3060 | -1.3329 | 0.4575 | -2.9134 | 0.003575 | 0.0581735 | no |
| Sobic.003G305300 | 388.9373 | -1.6095 | 0.5526 | -2.9125 | 0.0035856 | 0.0582582 | no |
| Sobic.004G010800 | 93.8553 | 1.3749 | 0.4721 | 2.9125 | 0.0035857 | 0.0582582 | no |
| Sobic.010G006300 | 899.9283 | -1.1258 | 0.3867 | -2.9116 | 0.0035954 | 0.0583708 | no |
| Sobic.004G223500 | 44.1792 | 2.7283 | 0.9376 | 2.9100 | 0.0036141 | 0.0586295 | no |
| Sobic.008G055100 | 14.0908 | 2.8197 | 0.9691 | 2.9097 | 0.0036176 | 0.0586402 | no |
| Sobic.010G154600 | 42.7520 | -2.4304 | 0.8355 | -2.9090 | 0.0036257 | 0.0587265 | no |
| Sobic.003G240100 | 163.6596 | 1.1690 | 0.4020 | 2.9079 | 0.0036389 | 0.0588852 | no |
| Sobic.009G237800 | 272.1822 | -1.3784 | 0.4740 | -2.9077 | 0.0036411 | 0.0588852 | no |
| Sobic.001G013800 | 1207.8438 | 1.2253 | 0.4215 | 2.9068 | 0.0036519 | 0.059016 | no |
| Sobic.003G306200 | 5963.2545 | -1.4209 | 0.4889 | -2.9064 | 0.0036565 | 0.059044 | no |

| Sobic.001G000400 | 363.8862 | 0.8455 | 0.2910 | 2.9057 | 0.0036642 | 0.0591232 | no |
| --- | --- | --- | --- | --- | --- | --- | --- |
| Sobic.004G260500 | 7.8342 | 4.3612 | 1.5012 | 2.9052 | 0.0036706 | 0.0591537 | no |
| Sobic.004G059600 | 5747.8357 | -0.8959 | 0.3084 | -2.9050 | 0.0036723 | 0.0591537 | no |
| Sobic.003G332700 | 3911.1193 | -1.2222 | 0.4208 | -2.9044 | 0.0036798 | 0.0591537 | no |
| Sobic.006G106800 | 631.9056 | -0.8956 | 0.3084 | -2.9044 | 0.00368 | 0.0591537 | no |
| Sobic.006G204000 | 2184.3232 | -1.8197 | 0.6265 | -2.9044 | 0.0036801 | 0.0591537 | no |
| Sobic.008G117800 | 1007.9909 | -1.4683 | 0.5056 | -2.9040 | 0.0036841 | 0.0591723 | no |
| Sobic.010G178600 | 653.8186 | 1.4440 | 0.4975 | 2.9028 | 0.003699 | 0.0593429 | no |
| Sobic.002G107800 | 13.6490 | 3.1125 | 1.0723 | 2.9026 | 0.0037003 | 0.0593429 | no |
| Sobic.010G263950 | 15.6763 | 6.2029 | 2.1374 | 2.9021 | 0.0037071 | 0.059364 | no |
| Sobic.003G349700 | 64.9926 | 1.8309 | 0.6309 | 2.9020 | 0.0037079 | 0.059364 | no |
| Sobic.002G285700 | 85.0913 | -2.1077 | 0.7263 | -2.9018 | 0.0037101 | 0.059364 | no |
| Sobic.001G078000 | 33646.4315 | -1.6097 | 0.5548 | -2.9013 | 0.0037166 | 0.0593835 | no |
| Sobic.005G023400 | 18.0600 | -2.6035 | 0.8974 | -2.9012 | 0.0037169 | 0.0593835 | no |
| Sobic.008G037000 | 233.9634 | 1.9296 | 0.6655 | 2.8995 | 0.0037372 | 0.0596343 | no |
| Sobic.003G280200 | 60.4695 | 2.8215 | 0.9731 | 2.8994 | 0.0037383 | 0.0596343 | no |
| Sobic.001G032100 | 4649.1775 | -1.5830 | 0.5461 | -2.8986 | 0.0037486 | 0.0596703 | no |
| Sobic.007G151800 | 214.3130 | -0.9639 | 0.3325 | -2.8985 | 0.0037497 | 0.0596703 | no |
| Sobic.003G422200 | 922.3733 | 1.9845 | 0.6847 | 2.8983 | 0.0037515 | 0.0596703 | no |
| Sobic.001G093600 | 3661.6313 | -2.0227 | 0.6979 | -2.8983 | 0.0037519 | 0.0596703 | no |
| Sobic.001G206300 | 301.8852 | 1.6674 | 0.5755 | 2.8975 | 0.0037621 | 0.059788 | no |
| Sobic.004G253050 | 541.8361 | -1.3656 | 0.4716 | -2.8955 | 0.0037858 | 0.0601201 | no |
| Sobic.001G480550 | 471.1236 | 0.9012 | 0.3113 | 2.8952 | 0.0037895 | 0.060133 | no |
| Sobic.008G061401 | 44.1058 | -1.6086 | 0.5556 | -2.8949 | 0.0037926 | 0.0601369 | no |
| Sobic.008G073200 | 2533.6597 | -1.3333 | 0.4606 | -2.8943 | 0.0037998 | 0.0602056 | no |
| Sobic.001G021500 | 826.8942 | 1.2025 | 0.4156 | 2.8936 | 0.0038088 | 0.0603037 | no |
| Sobic.009G121750 | 16.0356 | 2.9007 | 1.0029 | 2.8923 | 0.0038238 | 0.0604729 | no |
| Sobic.010G208700 | 612.2885 | 1.3122 | 0.4537 | 2.8921 | 0.0038271 | 0.0604729 | no |
| Sobic.010G269400 | 5582.2362 | -0.7048 | 0.2437 | -2.8919 | 0.0038286 | 0.0604729 | no |
| Sobic.009G232700 | 3095.4587 | -0.6836 | 0.2364 | -2.8918 | 0.003831 | 0.0604729 | no |
| Sobic.001G203700 | 40.4978 | 2.0066 | 0.6940 | 2.8913 | 0.003837 | 0.0605231 | no |
| Sobic.007G014200 | 670.1116 | -2.4067 | 0.8331 | -2.8890 | 0.0038647 | 0.0609134 | no |
| Sobic.005G015100 | 175.7134 | 0.8412 | 0.2912 | 2.8885 | 0.0038705 | 0.0609548 | no |
| Sobic.004G034300 | 798.1132 | 1.0878 | 0.3766 | 2.8883 | 0.0038731 | 0.0609548 | no |
| Sobic.005G036700 | 1698.6799 | -1.3038 | 0.4516 | -2.8871 | 0.0038882 | 0.0611475 | no |
| Sobic.004G039300 | 281.2707 | 1.0449 | 0.3620 | 2.8867 | 0.0038926 | 0.0611704 | no |
| Sobic.004G083700 | 992.6765 | -0.9738 | 0.3378 | -2.8828 | 0.0039418 | 0.0618332 | no |
| Sobic.003G252200 | 452.7285 | 1.6545 | 0.5739 | 2.8828 | 0.0039421 | 0.0618332 | no |
| Sobic.003G039400 | 114.7614 | 2.6425 | 0.9167 | 2.8826 | 0.0039436 | 0.0618332 | no |
| Sobic.002G007000 | 137.3212 | -1.2824 | 0.4450 | -2.8819 | 0.0039533 | 0.0619403 | no |
| Sobic.003G264700 | 2589.7894 | -0.8980 | 0.3117 | -2.8813 | 0.0039599 | 0.0619643 | no |
| Sobic.003G060400 | 411.3676 | -1.7903 | 0.6214 | -2.8813 | 0.0039607 | 0.0619643 | no |
| Sobic.001G110600 | 2000.0202 | 1.2282 | 0.4264 | 2.8807 | 0.0039681 | 0.0620333 | no |
| Sobic.006G248200 | 976.5069 | -1.7784 | 0.6177 | -2.8791 | 0.0039882 | 0.0622862 | no |
| Sobic.010G108500 | 192.8186 | -2.9418 | 1.0218 | -2.8789 | 0.0039902 | 0.0622862 | no |
| Sobic.004G012900 | 4073.6773 | -1.4313 | 0.4973 | -2.8780 | 0.0040024 | 0.062431 | no |
| Sobic.002G339900 | 61.6827 | 2.0046 | 0.6966 | 2.8777 | 0.0040055 | 0.0624333 | no |
| Sobic.009G181200 | 41.7007 | 3.2849 | 1.1420 | 2.8764 | 0.004022 | 0.0626439 | no |
| Sobic.008G051000 | 6071.4388 | -1.7079 | 0.5943 | -2.8737 | 0.0040572 | 0.0631358 | no |
| Sobic.003G092400 | 19.7643 | -2.0721 | 0.7211 | -2.8735 | 0.0040599 | 0.0631358 | no |
| Sobic.001G498800 | 65.4635 | 1.6866 | 0.5870 | 2.8733 | 0.0040626 | 0.0631358 | no |
| Sobic.001G360900 | 1585.8759 | 0.7748 | 0.2697 | 2.8723 | 0.0040745 | 0.063237 | no |
| Sobic.003G438800 | 45.2838 | 1.8613 | 0.6480 | 2.8723 | 0.0040751 | 0.063237 | no |
| Sobic.002G338000 | ######### | -2.1325 | 0.7435 | -2.8681 | 0.0041296 | 0.0640086 | no |
| Sobic.004G031300 | 359.8392 | 1.2623 | 0.4401 | 2.8680 | 0.0041309 | 0.0640086 | no |
| Sobic.002G352100 | ######### | -2.0497 | 0.7148 | -2.8676 | 0.0041359 | 0.0640399 | no |
| Sobic.004G071200 | 13342.9473 | 1.5909 | 0.5549 | 2.8668 | 0.0041464 | 0.0641276 | no |

| Sobic.009G066500 | 193.4397 | -2.1868 | 0.7628 | -2.8667 | 0.0041477 | 0.0641276 | no |
| --- | --- | --- | --- | --- | --- | --- | --- |
| Sobic.003G058300 | 338.4790 | -1.7215 | 0.6006 | -2.8661 | 0.0041557 | 0.0641644 | no |
| Sobic.001G528900 | 282.9876 | -1.3232 | 0.4617 | -2.8661 | 0.0041561 | 0.0641644 | no |
| Sobic.002G041800 | 1815.3536 | -1.4380 | 0.5020 | -2.8648 | 0.0041731 | 0.0643788 | no |
| Sobic.006G055800 | 4215.3380 | -1.0147 | 0.3543 | -2.8641 | 0.0041822 | 0.0644733 | no |
| Sobic.004G154000 | 77.3560 | 2.5770 | 0.8999 | 2.8636 | 0.0041881 | 0.064517 | no |
| Sobic.009G192100 | 4355.1412 | -0.9999 | 0.3492 | -2.8632 | 0.0041943 | 0.0645646 | no |
| Sobic.004G357800 | 119.5346 | -1.2759 | 0.4458 | -2.8623 | 0.004206 | 0.0646975 | no |
| Sobic.003G234400 | 34376.2251 | -1.0092 | 0.3526 | -2.8620 | 0.0042094 | 0.064703 | no |
| Sobic.001G441800 | 571.3639 | 1.0951 | 0.3827 | 2.8616 | 0.0042153 | 0.0647454 | no |
| Sobic.008G021300 | 7008.3248 | -1.6481 | 0.5763 | -2.8599 | 0.0042376 | 0.0650416 | no |
| Sobic.005G110499 | 5162.0124 | 0.7022 | 0.2457 | 2.8583 | 0.0042588 | 0.0653188 | no |
| Sobic.004G328700 | 153.9449 | -1.2070 | 0.4224 | -2.8578 | 0.0042659 | 0.0653811 | no |
| Sobic.010G261100 | 105.0108 | 1.8502 | 0.6475 | 2.8574 | 0.0042716 | 0.0654201 | no |
| Sobic.004G165600 | 63.8420 | -1.4602 | 0.5111 | -2.8570 | 0.0042772 | 0.0654543 | no |
| Sobic.002G368100 | 492.4358 | 1.6257 | 0.5691 | 2.8568 | 0.00428 | 0.0654543 | no |
| Sobic.003G350700 | 6004.2096 | 2.3991 | 0.8401 | 2.8559 | 0.0042915 | 0.0655824 | no |
| Sobic.001G519800 | ######### | -1.4043 | 0.4918 | -2.8553 | 0.0042994 | 0.0656558 | no |
| Sobic.010G165500 | 2263.6167 | 2.3803 | 0.8339 | 2.8543 | 0.004313 | 0.065807 | no |
| Sobic.008G090800 | 44.3047 | 3.3364 | 1.1690 | 2.8541 | 0.0043156 | 0.065807 | no |
| Sobic.001G068700 | 243.3783 | -1.0324 | 0.3617 | -2.8538 | 0.0043194 | 0.0658182 | no |
| Sobic.004G276500 | 1477.7872 | -1.2897 | 0.4523 | -2.8516 | 0.0043496 | 0.0662298 | no |
| Sobic.010G202900 | 92.8130 | 1.1530 | 0.4044 | 2.8508 | 0.0043614 | 0.066362 | no |
| Sobic.001G043000 | 399.8141 | -1.2990 | 0.4559 | -2.8493 | 0.0043811 | 0.0665985 | no |
| Sobic.010G238400 | 9.3739 | 4.4667 | 1.5677 | 2.8492 | 0.0043833 | 0.0665985 | no |
| Sobic.001G280900 | 865.6071 | 0.9384 | 0.3294 | 2.8485 | 0.0043932 | 0.0666698 | no |
| Sobic.009G115500 | 7.3046 | 4.2541 | 1.4935 | 2.8484 | 0.0043943 | 0.0666698 | no |
| Sobic.001G530350 | 252.4072 | 2.7428 | 0.9639 | 2.8457 | 0.0044316 | 0.0671877 | no |
| Sobic.001G529700 | 170.0216 | 1.7980 | 0.6319 | 2.8453 | 0.0044372 | 0.0672248 | no |
| Sobic.003G388000 | 309.8734 | 1.0517 | 0.3697 | 2.8446 | 0.0044466 | 0.067318 | no |
| Sobic.010G134700 | 61.4415 | 3.5387 | 1.2443 | 2.8438 | 0.0044578 | 0.0674398 | no |
| Sobic.001G242600 | 1085.3861 | -1.7671 | 0.6215 | -2.8432 | 0.0044661 | 0.0675161 | no |
| Sobic.001G542500 | 2308.4021 | 1.6241 | 0.5714 | 2.8421 | 0.0044822 | 0.0677107 | no |
| Sobic.006G191600 | 188.0525 | -1.9614 | 0.6902 | -2.8416 | 0.0044889 | 0.0677636 | no |
| Sobic.002G107000 | 401.2229 | 1.6718 | 0.5888 | 2.8395 | 0.0045187 | 0.0681655 | no |
| Sobic.007G209900 | 20.0060 | -2.5149 | 0.8858 | -2.8392 | 0.0045221 | 0.0681671 | no |
| Sobic.006G147300 | 16310.2503 | -1.1932 | 0.4205 | -2.8379 | 0.0045417 | 0.0683762 | no |
| Sobic.006G265900 | 1755.3621 | -0.8771 | 0.3091 | -2.8378 | 0.0045424 | 0.0683762 | no |
| Sobic.002G118700 | 355.3316 | 1.0834 | 0.3821 | 2.8356 | 0.0045734 | 0.0687932 | no |
| Sobic.008G046800 | 5483.4515 | -0.8876 | 0.3131 | -2.8349 | 0.0045837 | 0.0688989 | no |
| Sobic.010G245200 | 23754.6567 | -0.9658 | 0.3408 | -2.8339 | 0.0045979 | 0.0690627 | no |
| Sobic.010G123400 | 229.7790 | 1.1244 | 0.3968 | 2.8335 | 0.0046042 | 0.0690971 | no |
| Sobic.002G323900 | 990.9282 | -2.4756 | 0.8737 | -2.8333 | 0.0046067 | 0.0690971 | no |
| Sobic.004G228500 | 94.6613 | -2.0655 | 0.7293 | -2.8321 | 0.004625 | 0.069323 | no |
| Sobic.002G342800 | 200.2184 | -1.0105 | 0.3569 | -2.8317 | 0.0046299 | 0.0693464 | no |
| Sobic.001G436500 | 409.3786 | -1.2367 | 0.4368 | -2.8315 | 0.0046333 | 0.0693481 | no |
| Sobic.005G016400 | 729.9709 | 1.3990 | 0.4942 | 2.8311 | 0.004639 | 0.0693837 | no |
| Sobic.001G203600 | 266.3089 | 1.3146 | 0.4644 | 2.8307 | 0.0046446 | 0.0694029 | no |
| Sobic.K032350 | 105.7534 | -1.5077 | 0.5326 | -2.8305 | 0.0046468 | 0.0694029 | no |
| Sobic.009G184300 | 27.8595 | -2.0462 | 0.7230 | -2.8300 | 0.004655 | 0.0694113 | no |
| Sobic.004G096100 | 3552.9108 | -0.8786 | 0.3105 | -2.8300 | 0.0046551 | 0.0694113 | no |
| Sobic.007G141200 | 191.3339 | 1.8299 | 0.6467 | 2.8298 | 0.0046573 | 0.0694113 | no |
| Sobic.003G387100 | 4500.2551 | -0.9118 | 0.3223 | -2.8294 | 0.0046642 | 0.0694656 | no |
| Sobic.006G084600 | 76.4721 | 1.8259 | 0.6455 | 2.8285 | 0.0046768 | 0.0696046 | no |
| Sobic.003G002300 | ######### | -1.4032 | 0.4962 | -2.8282 | 0.0046815 | 0.0696243 | no |
| Sobic.002G073300 | 85.3357 | 2.0544 | 0.7266 | 2.8273 | 0.0046944 | 0.0697668 | no |
| Sobic.003G407300 | 2496.4634 | 0.8402 | 0.2972 | 2.8271 | 0.0046977 | 0.0697668 | no |

| Sobic.010G167500 | 1846.3944 | 0.7915 | 0.2800 | 2.8268 | 0.0047021 | 0.0697839 | no |
| --- | --- | --- | --- | --- | --- | --- | --- |
| Sobic.002G271700 | 5467.9446 | -1.3506 | 0.4778 | -2.8264 | 0.0047068 | 0.0698041 | no |
| Sobic.002G359700 | 1835.5393 | -1.7974 | 0.6361 | -2.8257 | 0.0047177 | 0.0698957 | no |
| Sobic.008G007100 | 40.9628 | 1.8171 | 0.6431 | 2.8256 | 0.0047196 | 0.0698957 | no |
| Sobic.005G076301 | 65.7989 | 1.3675 | 0.4842 | 2.8243 | 0.0047379 | 0.0701174 | no |
| Sobic.006G134400 | 1510.3656 | -0.9796 | 0.3469 | -2.8237 | 0.0047474 | 0.0702088 | no |
| Sobic.002G111200 | 2666.7113 | -0.8315 | 0.2945 | -2.8233 | 0.0047534 | 0.070249 | no |
| Sobic.009G229600 | 1797.3483 | 0.9752 | 0.3454 | 2.8230 | 0.0047573 | 0.0702563 | no |
| Sobic.006G021700 | 1176.8008 | 0.9879 | 0.3500 | 2.8224 | 0.004766 | 0.0703254 | no |
| Sobic.007G188100 | 863.6500 | 1.2682 | 0.4494 | 2.8223 | 0.0047686 | 0.0703254 | no |
| Sobic.003G154800 | 283.6290 | -2.3123 | 0.8195 | -2.8217 | 0.0047773 | 0.0704047 | no |
| Sobic.001G444400 | 14.6187 | 3.9887 | 1.4138 | 2.8214 | 0.0047821 | 0.0704255 | no |
| Sobic.002G193500 | 114.7445 | 0.9257 | 0.3281 | 2.8211 | 0.0047866 | 0.0704424 | no |
| Sobic.001G504400 | 6843.2752 | -1.1080 | 0.3929 | -2.8200 | 0.0048021 | 0.0706212 | no |
| Sobic.010G210200 | 300.9875 | 1.0720 | 0.3802 | 2.8197 | 0.0048075 | 0.070652 | no |
| Sobic.005G041000 | 42.6222 | 2.6552 | 0.9419 | 2.8190 | 0.004817 | 0.0707415 | no |
| Sobic.003G014700 | 58.2020 | 1.7760 | 0.6300 | 2.8188 | 0.0048205 | 0.0707442 | no |
| Sobic.002G394900 | 5.6316 | 4.6933 | 1.6655 | 2.8180 | 0.0048326 | 0.0708725 | no |
| Sobic.004G315100 | 367.0221 | -0.9764 | 0.3465 | -2.8176 | 0.0048388 | 0.0709149 | no |
| Sobic.003G121700 | 71.0308 | -2.4334 | 0.8638 | -2.8171 | 0.0048456 | 0.0709644 | no |
| Sobic.006G102000 | 16555.7336 | -1.0260 | 0.3643 | -2.8164 | 0.0048571 | 0.0710836 | no |
| Sobic.003G072566 | 286.8738 | 7.8753 | 2.7970 | 2.8156 | 0.0048687 | 0.0712039 | no |
| Sobic.002G034750 | 126.4611 | 1.3513 | 0.4800 | 2.8150 | 0.0048781 | 0.0712914 | no |
| Sobic.001G064700 | 136.2494 | 1.5922 | 0.5657 | 2.8145 | 0.0048853 | 0.0713471 | no |
| Sobic.009G027350 | 176.1881 | 1.7932 | 0.6373 | 2.8136 | 0.0048989 | 0.0714974 | no |
| Sobic.004G257200 | 565.3452 | 1.1618 | 0.4130 | 2.8133 | 0.0049039 | 0.0715197 | no |
| Sobic.001G166500 | 75.3219 | -1.8937 | 0.6733 | -2.8126 | 0.0049145 | 0.0716251 | no |
| Sobic.006G021250 | 40.5229 | 2.4762 | 0.8805 | 2.8123 | 0.0049185 | 0.071634 | no |
| Sobic.002G215700 | 668.9261 | 1.5147 | 0.5387 | 2.8118 | 0.0049273 | 0.0717122 | no |
| Sobic.006G162800 | 3587.3121 | -0.7597 | 0.2703 | -2.8104 | 0.0049477 | 0.0719597 | no |
| Sobic.004G283100 | 3262.6712 | -1.1246 | 0.4003 | -2.8096 | 0.0049601 | 0.0720768 | no |
| Sobic.007G032900 | 27.4575 | 2.2348 | 0.7955 | 2.8095 | 0.0049626 | 0.0720768 | no |
| Sobic.002G010200 | 7145.8194 | -0.9174 | 0.3266 | -2.8088 | 0.0049726 | 0.0721728 | no |
| Sobic.009G020900 | 1790.7865 | 1.0885 | 0.3876 | 2.8082 | 0.0049824 | 0.0722363 | no |
| Sobic.002G080000 | 145.9009 | 2.3194 | 0.8260 | 2.8081 | 0.0049838 | 0.0722363 | no |
| Sobic.003G199800 | 13299.5678 | -1.0496 | 0.3738 | -2.8078 | 0.0049886 | 0.0722409 | no |
| Sobic.008G130800 | 83.5230 | 3.1881 | 1.1356 | 2.8075 | 0.0049929 | 0.0722409 | no |
| Sobic.006G052100 | 705.3096 | -1.0019 | 0.3569 | -2.8074 | 0.0049944 | 0.0722409 | no |
| Sobic.010G223500 | 15.0623 | 2.6644 | 0.9492 | 2.8072 | 0.004998 | 0.0722428 | no |
| Sobic.001G146400 | 279.1741 | -3.0874 | 1.1001 | -2.8064 | 0.0050091 | 0.0723541 | no |
| Sobic.001G135500 | 154.9144 | 1.3620 | 0.4858 | 2.8038 | 0.0050497 | 0.0728091 | no |
| Sobic.005G002400 | 1240.9200 | 2.1932 | 0.7822 | 2.8038 | 0.0050507 | 0.0728091 | no |
| Sobic.004G208600 | 531.9984 | -0.9048 | 0.3227 | -2.8036 | 0.0050531 | 0.0728091 | no |
| Sobic.009G245000 | 3283.0953 | 2.5468 | 0.9085 | 2.8033 | 0.005058 | 0.0728091 | no |
| Sobic.008G094400 | 4547.2128 | -0.7237 | 0.2582 | -2.8032 | 0.0050593 | 0.0728091 | no |
| Sobic.010G126400 | 83.2631 | -2.1766 | 0.7765 | -2.8031 | 0.0050613 | 0.0728091 | no |
| Sobic.001G117200 | 1404.2119 | 0.7865 | 0.2807 | 2.8024 | 0.0050727 | 0.0729226 | no |
| Sobic.003G307801 | 38.6354 | 2.6640 | 0.9508 | 2.8017 | 0.0050827 | 0.0730164 | no |
| Sobic.007G108900 | 8082.0664 | -1.1734 | 0.4190 | -2.8004 | 0.0051043 | 0.0732777 | no |
| Sobic.010G181300 | 8601.6020 | -1.1121 | 0.3973 | -2.7989 | 0.0051283 | 0.0735613 | no |
| Sobic.002G395900 | 13.4231 | 2.2879 | 0.8175 | 2.7987 | 0.005131 | 0.0735613 | no |
| Sobic.003G333400 | 3563.4774 | -1.1484 | 0.4104 | -2.7980 | 0.0051417 | 0.0736636 | no |
| Sobic.007G045500 | 4499.6843 | -1.1478 | 0.4102 | -2.7978 | 0.0051452 | 0.0736636 | no |
| Sobic.001G141800 | 32.2323 | 2.2921 | 0.8193 | 2.7975 | 0.0051504 | 0.0736879 | no |
| Sobic.001G329300 | 5693.5965 | -0.8478 | 0.3031 | -2.7970 | 0.0051576 | 0.0737421 | no |
| Sobic.008G063100 | ######### | -1.2533 | 0.4488 | -2.7926 | 0.005229 | 0.0746393 | no |
| Sobic.001G063400 | 717.8708 | -1.1316 | 0.4052 | -2.7925 | 0.0052299 | 0.0746393 | no |

| Sobic.002G174000 | 6183.7604 | -1.7150 | 0.6142 | -2.7925 | 0.005231 | 0.0746393 | no |
| --- | --- | --- | --- | --- | --- | --- | --- |
| Sobic.004G176900 | 132.6363 | 1.1646 | 0.4172 | 2.7914 | 0.0052477 | 0.0748272 | no |
| Sobic.002G286400 | 27969.4758 | -1.3288 | 0.4764 | -2.7895 | 0.0052796 | 0.0752305 | no |
| Sobic.001G240000 | 18220.1059 | -1.3672 | 0.4902 | -2.7890 | 0.0052872 | 0.0752876 | no |
| Sobic.007G123700 | 7.6330 | 4.2265 | 1.5159 | 2.7882 | 0.0053006 | 0.0754286 | no |
| Sobic.003G037400 | 2698.0825 | -1.9724 | 0.7077 | -2.7870 | 0.0053192 | 0.0756009 | no |
| Sobic.001G505800 | 448.5146 | -1.5373 | 0.5517 | -2.7865 | 0.0053273 | 0.0756009 | no |
| Sobic.004G056300 | 580.4566 | 2.3526 | 0.8443 | 2.7865 | 0.0053282 | 0.0756009 | no |
| Sobic.010G063100 | 23.8457 | -2.0873 | 0.7491 | -2.7865 | 0.0053287 | 0.0756009 | no |
| Sobic.004G319100 | 53.5303 | 2.2695 | 0.8145 | 2.7863 | 0.0053307 | 0.0756009 | no |
| Sobic.001G365700 | 102.0759 | 2.7261 | 0.9786 | 2.7856 | 0.005343 | 0.0757242 | no |
| Sobic.002G097400 | 59.2749 | 1.2144 | 0.4360 | 2.7853 | 0.0053484 | 0.0757432 | no |
| Sobic.004G225600 | 1527.1174 | -0.9033 | 0.3243 | -2.7851 | 0.0053515 | 0.0757432 | no |
| Sobic.010G141966 | 23.1786 | 2.8175 | 1.0121 | 2.7839 | 0.0053704 | 0.0759603 | no |
| Sobic.005G085800 | 59.8700 | -1.6244 | 0.5836 | -2.7835 | 0.0053769 | 0.0760011 | no |
| Sobic.009G063800 | 81.5259 | 1.1179 | 0.4017 | 2.7833 | 0.0053815 | 0.0760144 | no |
| Sobic.002G390500 | 22074.1186 | -1.2514 | 0.4497 | -2.7825 | 0.0053941 | 0.0761418 | no |
| Sobic.001G054600 | 62.7735 | -1.4438 | 0.5190 | -2.7821 | 0.0054007 | 0.0761847 | no |
| Sobic.005G202600 | 72.8128 | -2.1269 | 0.7648 | -2.7809 | 0.0054205 | 0.0763704 | no |
| Sobic.003G289200 | 12.9969 | 4.3703 | 1.5715 | 2.7809 | 0.0054211 | 0.0763704 | no |
| Sobic.010G110000 | 426.3147 | -1.1259 | 0.4051 | -2.7795 | 0.0054448 | 0.0766389 | no |
| Sobic.002G283300 | 1540.9464 | -0.7356 | 0.2647 | -2.7791 | 0.0054505 | 0.0766389 | no |
| Sobic.007G226000 | 66.8939 | 2.4576 | 0.8843 | 2.7791 | 0.0054511 | 0.0766389 | no |
| Sobic.006G159100 | 13.5478 | 2.9110 | 1.0476 | 2.7787 | 0.0054576 | 0.0766792 | no |
| Sobic.002G284800 | 36.3243 | -2.8761 | 1.0354 | -2.7777 | 0.0054745 | 0.0768648 | no |
| Sobic.008G157000 | 53.2517 | -3.4271 | 1.2340 | -2.7773 | 0.005482 | 0.076889 | no |
| Sobic.007G128200 | 18.0424 | 2.4789 | 0.8926 | 2.7772 | 0.0054835 | 0.076889 | no |
| Sobic.004G265600 | 766.7703 | 1.0097 | 0.3637 | 2.7763 | 0.0054979 | 0.0770119 | no |
| Sobic.009G178500 | 97.4377 | 2.6894 | 0.9687 | 2.7762 | 0.0054995 | 0.0770119 | no |
| Sobic.007G077001 | 81.2961 | -2.6776 | 0.9646 | -2.7759 | 0.0055042 | 0.0770258 | no |
| Sobic.002G035700 | 77.5290 | 2.8536 | 1.0282 | 2.7754 | 0.005514 | 0.077029 | no |
| Sobic.004G250700 | 461.1997 | 0.8374 | 0.3017 | 2.7753 | 0.0055143 | 0.077029 | no |
| Sobic.001G463100 | 1920.6503 | -1.0719 | 0.3863 | -2.7750 | 0.0055202 | 0.077029 | no |
| Sobic.010G176200 | 3484.1347 | -0.8351 | 0.3010 | -2.7748 | 0.0055238 | 0.077029 | no |
| Sobic.003G256700 | 1138.8223 | -1.1653 | 0.4200 | -2.7744 | 0.0055303 | 0.077029 | no |
| Sobic.001G198600 | 25.8582 | -1.8895 | 0.6811 | -2.7743 | 0.0055323 | 0.077029 | no |
| Sobic.009G254700 | 17.9170 | -2.0864 | 0.7521 | -2.7742 | 0.0055335 | 0.077029 | no |
| Sobic.007G132500 | 1984.5318 | -1.1544 | 0.4161 | -2.7741 | 0.0055361 | 0.077029 | no |
| Sobic.003G200700 | 9.6484 | 4.0991 | 1.4778 | 2.7739 | 0.0055389 | 0.077029 | no |
| Sobic.009G032000 | 7.0964 | -5.5240 | 1.9915 | -2.7738 | 0.0055413 | 0.077029 | no |
| Sobic.005G010200 | 1561.3450 | -1.3126 | 0.4733 | -2.7736 | 0.0055446 | 0.077029 | no |
| Sobic.001G493800 | 822.0464 | -1.7111 | 0.6171 | -2.7729 | 0.0055553 | 0.0770939 | no |
| Sobic.002G368700 | 3948.8715 | -0.7889 | 0.2845 | -2.7726 | 0.0055614 | 0.0770939 | no |
| Sobic.008G177100 | 52.8182 | 1.2095 | 0.4362 | 2.7726 | 0.0055618 | 0.0770939 | no |
| Sobic.010G143900 | 1092.6322 | -1.0930 | 0.3942 | -2.7724 | 0.0055639 | 0.0770939 | no |
| Sobic.004G254000 | 4720.0756 | -0.8092 | 0.2919 | -2.7720 | 0.0055718 | 0.0771005 | no |
| Sobic.003G424000 | 11.9664 | 3.5398 | 1.2770 | 2.7719 | 0.0055734 | 0.0771005 | no |
| Sobic.003G281500 | 159.9659 | 2.1297 | 0.7684 | 2.7718 | 0.0055753 | 0.0771005 | no |
| Sobic.008G081200 | 1001.9364 | -0.9588 | 0.3460 | -2.7713 | 0.0055827 | 0.0771523 | no |
| Sobic.002G288800 | 36.3947 | -1.7964 | 0.6483 | -2.7709 | 0.0055905 | 0.077209 | no |
| Sobic.009G241000 | 632.4877 | 2.8559 | 1.0309 | 2.7704 | 0.0055983 | 0.0772659 | no |
| Sobic.001G496200 | 2552.2431 | -2.1415 | 0.7731 | -2.7700 | 0.0056051 | 0.077275 | no |
| Sobic.001G261545 | 897.4422 | -2.1783 | 0.7864 | -2.7699 | 0.0056077 | 0.077275 | no |
| Sobic.001G325700 | 3125.8490 | -1.1395 | 0.4114 | -2.7697 | 0.0056099 | 0.077275 | no |
| Sobic.009G236200 | 94.2983 | 1.2141 | 0.4386 | 2.7683 | 0.0056352 | 0.0775412 | no |
| Sobic.002G298800 | 1872.7156 | -1.2936 | 0.4673 | -2.7682 | 0.0056366 | 0.0775412 | no |
| Sobic.007G129100 | 172.7687 | -2.3716 | 0.8571 | -2.7672 | 0.0056546 | 0.0776386 | no |

| Sobic.005G034500 | 11.9112 | -3.0878 | 1.1159 | -2.7671 | 0.0056555 | 0.0776386 | no |
| --- | --- | --- | --- | --- | --- | --- | --- |
| Sobic.002G120300 | 1377.9803 | -1.1772 | 0.4254 | -2.7670 | 0.0056576 | 0.0776386 | no |
| Sobic.001G296500 | 2281.1670 | 0.7244 | 0.2618 | 2.7669 | 0.0056584 | 0.0776386 | no |
| Sobic.003G229500 | 2080.7188 | 1.0331 | 0.3735 | 2.7660 | 0.0056755 | 0.0778227 | no |
| Sobic.001G150200 | 219.8792 | 1.4094 | 0.5096 | 2.7657 | 0.0056809 | 0.0778452 | no |
| Sobic.003G332000 | 833.9684 | 1.3799 | 0.4992 | 2.7642 | 0.0057067 | 0.0780868 | no |
| Sobic.004G204400 | 1260.5214 | 0.7225 | 0.2614 | 2.7641 | 0.0057073 | 0.0780868 | no |
| Sobic.003G071800 | 8.8596 | -2.7861 | 1.0080 | -2.7639 | 0.0057114 | 0.0780868 | no |
| Sobic.004G223400 | 38.5446 | -2.4027 | 0.8694 | -2.7638 | 0.0057133 | 0.0780868 | no |
| Sobic.004G176100 | 7.9607 | -2.8445 | 1.0294 | -2.7633 | 0.0057215 | 0.0780978 | no |
| Sobic.002G085300 | 140.3748 | 1.8599 | 0.6731 | 2.7632 | 0.0057236 | 0.0780978 | no |
| Sobic.004G165400 | 424.9752 | -0.6941 | 0.2512 | -2.7631 | 0.0057252 | 0.0780978 | no |
| Sobic.001G411300 | 4421.0145 | -1.4091 | 0.5101 | -2.7625 | 0.0057361 | 0.0781958 | no |
| Sobic.002G349300 | 18.2687 | 1.9972 | 0.7231 | 2.7620 | 0.005745 | 0.0782664 | no |
| Sobic.009G083800 | 241.6133 | 1.1373 | 0.4118 | 2.7616 | 0.005752 | 0.0783105 | no |
| Sobic.006G231900 | 157.4231 | 1.7924 | 0.6493 | 2.7605 | 0.0057705 | 0.0785129 | no |
| Sobic.004G027900 | 2904.4355 | -0.7779 | 0.2819 | -2.7589 | 0.0057993 | 0.0788532 | no |
| Sobic.006G033200 | 1026.6791 | 0.7199 | 0.2610 | 2.7581 | 0.0058141 | 0.0790039 | no |
| Sobic.003G202800 | 2036.6283 | -1.0076 | 0.3654 | -2.7578 | 0.0058196 | 0.0790271 | no |
| Sobic.001G085700 | 845.4246 | 1.0336 | 0.3749 | 2.7572 | 0.0058291 | 0.0791053 | no |
| Sobic.008G171300 | 15851.7699 | -1.1024 | 0.4001 | -2.7551 | 0.0058677 | 0.0795753 | no |
| Sobic.009G168900 | 176.5208 | -1.5620 | 0.5670 | -2.7549 | 0.0058713 | 0.0795753 | no |
| Sobic.010G100800 | 450.5124 | 1.5779 | 0.5729 | 2.7544 | 0.0058797 | 0.0796383 | no |
| Sobic.010G000500 | ######### | -1.5188 | 0.5519 | -2.7519 | 0.0059256 | 0.0802089 | no |
| Sobic.004G252400 | 75.9726 | 1.0968 | 0.3986 | 2.7516 | 0.0059297 | 0.080213 | no |
| Sobic.009G073400 | 286.3880 | 0.9684 | 0.3520 | 2.7513 | 0.0059358 | 0.0802435 | no |
| Sobic.003G243600 | 9986.8549 | -0.9204 | 0.3346 | -2.7510 | 0.0059417 | 0.0802455 | no |
| Sobic.001G154500 | 1045.2316 | -1.0816 | 0.3932 | -2.7509 | 0.0059435 | 0.0802455 | no |
| Sobic.007G193900 | 314.4921 | 1.1586 | 0.4213 | 2.7498 | 0.0059628 | 0.0804198 | no |
| Sobic.006G273100 | 218.9092 | -1.8676 | 0.6792 | -2.7497 | 0.0059641 | 0.0804198 | no |
| Sobic.003G037300 | 2556.7411 | -1.0030 | 0.3650 | -2.7478 | 0.0059991 | 0.08084 | no |
| Sobic.008G153600 | 617.2417 | 0.7974 | 0.2904 | 2.7461 | 0.00603 | 0.0812044 | no |
| Sobic.001G500600 | 1180.6840 | -0.8940 | 0.3257 | -2.7452 | 0.0060483 | 0.0813995 | no |
| Sobic.008G072400 | 207.9216 | -1.7017 | 0.6201 | -2.7442 | 0.0060665 | 0.0815919 | no |
| Sobic.001G386400 | 558.6829 | 1.3307 | 0.4850 | 2.7437 | 0.0060749 | 0.0815957 | no |
| Sobic.001G416100 | 129.3233 | -1.3908 | 0.5069 | -2.7436 | 0.0060778 | 0.0815957 | no |
| Sobic.002G270000 | 156.5527 | 1.8850 | 0.6871 | 2.7435 | 0.0060787 | 0.0815957 | no |
| Sobic.009G070800 | 1328.5345 | -1.6605 | 0.6053 | -2.7433 | 0.0060822 | 0.0815957 | no |
| Sobic.004G138400 | 20.2206 | 3.5449 | 1.2927 | 2.7422 | 0.0061034 | 0.0818275 | no |
| Sobic.001G050800 | 16.9390 | 2.4193 | 0.8826 | 2.7410 | 0.0061246 | 0.0820536 | no |
| Sobic.006G057300 | 153.2873 | -1.6792 | 0.6127 | -2.7408 | 0.0061286 | 0.0820536 | no |
| Sobic.002G100200 | 7.6513 | 3.5169 | 1.2833 | 2.7405 | 0.0061339 | 0.0820536 | no |
| Sobic.002G306900 | 9221.4478 | -0.8650 | 0.3157 | -2.7404 | 0.0061358 | 0.0820536 | no |
| Sobic.001G387700 | 29.9802 | -1.9623 | 0.7163 | -2.7396 | 0.0061511 | 0.082205 | no |
| Sobic.004G215600 | 58.1278 | 1.7215 | 0.6288 | 2.7378 | 0.0061842 | 0.0825776 | no |
| Sobic.005G019250 | 6.7867 | 4.0763 | 1.4893 | 2.7371 | 0.0061977 | 0.0825776 | no |
| Sobic.010G118000 | 332.6855 | 0.9129 | 0.3335 | 2.7370 | 0.0061994 | 0.0825776 | no |
| Sobic.004G247400 | 1987.2089 | -0.7719 | 0.2820 | -2.7370 | 0.0062 | 0.0825776 | no |
| Sobic.001G407900 | 2015.3589 | -1.2659 | 0.4625 | -2.7370 | 0.0062007 | 0.0825776 | no |
| Sobic.008G045600 | 47.5127 | -1.1294 | 0.4127 | -2.7369 | 0.0062024 | 0.0825776 | no |
| Sobic.004G193200 | 274.3701 | -1.3532 | 0.4946 | -2.7359 | 0.0062202 | 0.0827618 | no |
| Sobic.003G353400 | 188.9134 | 1.7353 | 0.6343 | 2.7357 | 0.0062244 | 0.0827655 | no |
| Sobic.003G246600 | 5493.5386 | -1.2863 | 0.4704 | -2.7348 | 0.0062425 | 0.0829536 | no |
| Sobic.010G253300 | 780.1609 | 0.7748 | 0.2834 | 2.7334 | 0.0062685 | 0.08322 | no |
| Sobic.010G026200 | 67.1117 | 1.5686 | 0.5739 | 2.7333 | 0.0062704 | 0.08322 | no |
| Sobic.008G013600 | 58.6462 | -1.6462 | 0.6024 | -2.7329 | 0.0062773 | 0.0832595 | no |
| Sobic.001G346200 | 27.5969 | 2.7016 | 0.9889 | 2.7319 | 0.0062973 | 0.0834717 | no |

| Sobic.003G393200 | 258.0279 | 0.7575 | 0.2773 | 2.7313 | 0.0063076 | 0.0834719 | no |
| --- | --- | --- | --- | --- | --- | --- | --- |
| Sobic.001G221300 | 508.9407 | -1.2635 | 0.4626 | -2.7313 | 0.0063077 | 0.0834719 | no |
| Sobic.010G194001 | 210.7810 | -1.6184 | 0.5926 | -2.7309 | 0.0063168 | 0.0834719 | no |
| Sobic.009G055800 | 4460.9242 | -1.0896 | 0.3990 | -2.7309 | 0.0063168 | 0.0834719 | no |
| Sobic.001G417500 | 212.0969 | 1.0170 | 0.3724 | 2.7309 | 0.0063171 | 0.0834719 | no |
| Sobic.004G331700 | 20493.1487 | -1.1389 | 0.4172 | -2.7299 | 0.0063354 | 0.083662 | no |
| Sobic.003G438200 | 684.3360 | 0.8530 | 0.3125 | 2.7294 | 0.0063456 | 0.0837433 | no |
| Sobic.001G200900 | 9.8529 | -4.4792 | 1.6413 | -2.7290 | 0.0063534 | 0.083794 | no |
| Sobic.006G281400 | 204.6467 | 0.9880 | 0.3621 | 2.7286 | 0.0063608 | 0.0838393 | no |
| Sobic.004G179400 | 9106.5112 | -0.9710 | 0.3560 | -2.7279 | 0.0063744 | 0.0839163 | no |
| Sobic.001G185400 | 63.7753 | 1.4514 | 0.5321 | 2.7279 | 0.0063746 | 0.0839163 | no |
| Sobic.001G048400 | 6335.1609 | -1.0944 | 0.4012 | -2.7274 | 0.0063843 | 0.0839839 | no |
| Sobic.010G117800 | 90.0817 | 2.3910 | 0.8767 | 2.7272 | 0.0063877 | 0.0839839 | no |
| Sobic.008G112500 | 506.1892 | 0.6770 | 0.2483 | 2.7267 | 0.0063964 | 0.0840455 | no |
| Sobic.003G069950 | 12.9737 | 3.7321 | 1.3692 | 2.7258 | 0.0064139 | 0.0842228 | no |
| Sobic.001G384600 | 58.7831 | 1.4498 | 0.5319 | 2.7255 | 0.0064198 | 0.084249 | no |
| Sobic.009G043000 | 9888.4269 | -0.9592 | 0.3520 | -2.7253 | 0.0064249 | 0.0842625 | no |
| Sobic.005G004100 | 545.1585 | 0.9522 | 0.3495 | 2.7242 | 0.0064452 | 0.0844764 | no |
| Sobic.004G289500 | 618.2168 | 1.1939 | 0.4385 | 2.7231 | 0.0064681 | 0.0847087 | no |
| Sobic.001G440401 | 46.8345 | 2.2978 | 0.8439 | 2.7228 | 0.0064723 | 0.0847087 | no |
| Sobic.002G049200 | 587.7546 | 1.0739 | 0.3944 | 2.7227 | 0.006475 | 0.0847087 | no |
| Sobic.003G362200 | 791.1553 | -1.8848 | 0.6925 | -2.7216 | 0.0064962 | 0.0848879 | no |
| Sobic.004G227600 | 1206.3540 | 1.1478 | 0.4217 | 2.7216 | 0.0064967 | 0.0848879 | no |
| Sobic.009G034600 | 4782.7486 | -0.6983 | 0.2567 | -2.7203 | 0.0065217 | 0.0851622 | no |
| Sobic.003G277500 | 2640.6567 | 0.9987 | 0.3672 | 2.7200 | 0.0065286 | 0.0851987 | no |
| Sobic.009G237350 | 14.7629 | -2.2904 | 0.8424 | -2.7188 | 0.0065511 | 0.0854333 | no |
| Sobic.008G009000 | 34.2783 | 2.1951 | 0.8074 | 2.7187 | 0.0065547 | 0.0854333 | no |
| Sobic.010G061100 | 326.6395 | 0.7454 | 0.2742 | 2.7184 | 0.0065592 | 0.0854395 | no |
| Sobic.008G054100 | 1623.8141 | -0.9524 | 0.3504 | -2.7179 | 0.0065703 | 0.0854815 | no |
| Sobic.001G476600 | 78.4061 | 1.4578 | 0.5364 | 2.7179 | 0.0065705 | 0.0854815 | no |
| Sobic.006G064200 | 45260.7820 | -1.1258 | 0.4143 | -2.7173 | 0.0065821 | 0.0855798 | no |
| Sobic.002G131700 | 4428.1489 | -0.9548 | 0.3514 | -2.7170 | 0.0065883 | 0.085607 | no |
| Sobic.001G348900 | 45.4553 | -1.4833 | 0.5461 | -2.7160 | 0.0066076 | 0.0857923 | no |
| Sobic.004G197700 | 2175.3473 | -1.1745 | 0.4325 | -2.7158 | 0.0066107 | 0.0857923 | no |
| Sobic.003G117600 | 46.3045 | 2.5935 | 0.9553 | 2.7147 | 0.0066328 | 0.0860262 | no |
| Sobic.001G378550 | 1129.7007 | -1.6631 | 0.6127 | -2.7144 | 0.0066388 | 0.0860515 | no |
| Sobic.002G231200 | 5955.6244 | -1.3257 | 0.4885 | -2.7139 | 0.0066494 | 0.0861039 | no |
| Sobic.007G178100 | 272.2856 | 1.1366 | 0.4188 | 2.7138 | 0.006651 | 0.0861039 | no |
| Sobic.007G090406 | 886.2861 | -1.0065 | 0.3711 | -2.7123 | 0.0066809 | 0.0864383 | no |
| Sobic.009G143100 | ######### | -1.1703 | 0.4316 | -2.7118 | 0.0066928 | 0.0865381 | no |
| Sobic.001G282300 | 72.2429 | 1.2183 | 0.4494 | 2.7111 | 0.0067051 | 0.0866445 | no |
| Sobic.001G010800 | 860.7972 | -0.9981 | 0.3684 | -2.7092 | 0.0067456 | 0.0870792 | no |
| Sobic.009G029600 | 1732.5499 | 0.7445 | 0.2748 | 2.7090 | 0.0067482 | 0.0870792 | no |
| Sobic.010G205600 | 390.9363 | 1.0127 | 0.3738 | 2.7089 | 0.0067511 | 0.0870792 | no |
| Sobic.001G171300 | 23.5793 | 3.2745 | 1.2090 | 2.7086 | 0.0067572 | 0.0871048 | no |
| Sobic.004G274800 | 35.4282 | 1.9899 | 0.7350 | 2.7074 | 0.0067807 | 0.0873534 | no |
| Sobic.010G153100 | 2853.0278 | 0.7730 | 0.2856 | 2.7071 | 0.0067872 | 0.0873846 | no |
| Sobic.001G290100 | 2569.0008 | 0.9650 | 0.3565 | 2.7067 | 0.0067949 | 0.0874294 | no |
| Sobic.009G170301 | 93.7429 | 1.2222 | 0.4517 | 2.7058 | 0.0068142 | 0.0876195 | no |
| Sobic.003G269700 | 11.7590 | 2.5179 | 0.9306 | 2.7056 | 0.0068179 | 0.0876195 | no |
| Sobic.001G299300 | 80.7303 | -1.2335 | 0.4561 | -2.7046 | 0.0068377 | 0.0877903 | no |
| Sobic.009G239500 | 777.1189 | 0.8910 | 0.3294 | 2.7045 | 0.0068402 | 0.0877903 | no |
| Sobic.002G191800 | 729.9219 | 1.1465 | 0.4240 | 2.7044 | 0.0068437 | 0.0877903 | no |
| Sobic.004G161200 | 305.1701 | -1.2197 | 0.4511 | -2.7040 | 0.0068512 | 0.0878328 | no |
| Sobic.007G064000 | 193.5807 | 1.3083 | 0.4839 | 2.7036 | 0.0068586 | 0.0878741 | no |
| Sobic.002G300000 | 90.3544 | 1.6780 | 0.6210 | 2.7021 | 0.00689 | 0.0882231 | no |
| Sobic.002G314000 | 46.9544 | -1.8548 | 0.6867 | -2.7009 | 0.0069156 | 0.0884965 | no |

| Sobic.001G452000 | 181.5978 | 0.9401 | 0.3481 | 2.7006 | 0.0069207 | 0.0885085 | no |
| --- | --- | --- | --- | --- | --- | --- | --- |
| Sobic.002G311700 | 27.0013 | 2.9715 | 1.1004 | 2.7003 | 0.0069277 | 0.0885343 | no |
| Sobic.001G532000 | 334.5140 | -0.9005 | 0.3335 | -2.7001 | 0.0069317 | 0.0885343 | no |
| Sobic.004G343800 | 9.8990 | 2.9652 | 1.0982 | 2.6999 | 0.0069353 | 0.0885343 | no |
| Sobic.010G160700 | ######### | -1.2942 | 0.4794 | -2.6993 | 0.0069477 | 0.0886393 | no |
| Sobic.004G124500 | 2174.3773 | 0.8348 | 0.3094 | 2.6986 | 0.0069631 | 0.0887819 | no |
| Sobic.002G403100 | 6.9343 | -3.1629 | 1.1722 | -2.6982 | 0.0069713 | 0.088832 | no |
| Sobic.002G073000 | 4.8750 | -4.9688 | 1.8421 | -2.6974 | 0.0069878 | 0.0889637 | no |
| Sobic.004G128100 | 256.3771 | 0.9384 | 0.3479 | 2.6973 | 0.00699 | 0.0889637 | no |
| Sobic.004G088000 | 25.0176 | -2.7187 | 1.0080 | -2.6970 | 0.0069969 | 0.0889804 | no |
| Sobic.003G322900 | 26.4704 | 4.0398 | 1.4981 | 2.6965 | 0.0070069 | 0.0889804 | no |
| Sobic.003G089600 | 172.2518 | -2.0876 | 0.7742 | -2.6965 | 0.0070072 | 0.0889804 | no |
| Sobic.004G163200 | 817.5152 | 1.2869 | 0.4772 | 2.6965 | 0.0070082 | 0.0889804 | no |
| Sobic.007G110200 | 3853.4071 | -1.8293 | 0.6785 | -2.6960 | 0.0070173 | 0.0890416 | no |
| Sobic.001G079600 | 496.3299 | -2.0142 | 0.7473 | -2.6953 | 0.0070317 | 0.0891652 | no |
| Sobic.001G006000 | 2865.4142 | -1.1006 | 0.4084 | -2.6952 | 0.0070355 | 0.0891652 | no |
| Sobic.001G021300 | 172.0819 | -1.3340 | 0.4950 | -2.6949 | 0.0070414 | 0.0891875 | no |
| Sobic.005G070000 | 9.0465 | 4.3696 | 1.6217 | 2.6945 | 0.0070488 | 0.0892264 | no |
| Sobic.004G178300 | 483.9209 | 0.8911 | 0.3308 | 2.6940 | 0.0070609 | 0.0893268 | no |
| Sobic.008G131400 | 27.4667 | 2.4535 | 0.9108 | 2.6937 | 0.0070672 | 0.0893531 | no |
| Sobic.006G234100 | 16498.6938 | -1.3285 | 0.4933 | -2.6933 | 0.0070751 | 0.0893987 | no |
| Sobic.004G276300 | 16.1522 | 3.2523 | 1.2077 | 2.6930 | 0.0070805 | 0.0894139 | no |
| Sobic.005G113300 | 4170.1675 | 1.1195 | 0.4158 | 2.6922 | 0.0070989 | 0.0895468 | no |
| Sobic.006G001900 | 27.1924 | -1.8093 | 0.6721 | -2.6921 | 0.0070995 | 0.0895468 | no |
| Sobic.010G109800 | 23.9758 | -2.4890 | 0.9250 | -2.6908 | 0.007128 | 0.0898521 | no |
| Sobic.001G494100 | 14.8978 | 2.5102 | 0.9332 | 2.6898 | 0.0071488 | 0.0900305 | no |
| Sobic.006G100600 | 2779.1591 | -0.7996 | 0.2973 | -2.6897 | 0.0071513 | 0.0900305 | no |
| Sobic.008G087900 | 1746.9395 | -0.6285 | 0.2337 | -2.6895 | 0.007155 | 0.0900305 | no |
| Sobic.009G046900 | 1818.8692 | 0.8517 | 0.3167 | 2.6892 | 0.0071615 | 0.0900584 | no |
| Sobic.004G322300 | 169.9020 | 0.9361 | 0.3481 | 2.6890 | 0.0071671 | 0.0900758 | no |
| Sobic.001G024200 | 332.6015 | -0.7360 | 0.2738 | -2.6883 | 0.0071821 | 0.0902103 | no |
| Sobic.002G284901 | 95.7989 | -2.1948 | 0.8165 | -2.6880 | 0.0071878 | 0.0902289 | no |
| Sobic.009G183700 | 35283.3471 | -1.5266 | 0.5680 | -2.6876 | 0.0071962 | 0.0902802 | no |
| Sobic.003G033000 | 262.6917 | 2.0671 | 0.7693 | 2.6872 | 0.0072062 | 0.0902925 | no |
| Sobic.001G231200 | 794.7253 | 1.2131 | 0.4515 | 2.6871 | 0.0072067 | 0.0902925 | no |
| Sobic.006G134500 | 224.7034 | 0.7930 | 0.2951 | 2.6870 | 0.00721 | 0.0902925 | no |
| Sobic.009G233200 | 2229.7939 | 0.8342 | 0.3105 | 2.6864 | 0.0072237 | 0.0903723 | no |
| Sobic.001G494300 | 21.7663 | 2.5436 | 0.9469 | 2.6862 | 0.007228 | 0.0903723 | no |
| Sobic.007G106500 | 958.7912 | 1.4533 | 0.5411 | 2.6858 | 0.0072366 | 0.0903723 | no |
| Sobic.003G030200 | 173.9044 | 1.8612 | 0.6930 | 2.6857 | 0.0072379 | 0.0903723 | no |
| Sobic.006G047000 | 1404.5147 | -1.8849 | 0.7018 | -2.6856 | 0.0072393 | 0.0903723 | no |
| Sobic.009G174700 | 6987.8250 | -1.1148 | 0.4151 | -2.6855 | 0.0072421 | 0.0903723 | no |
| Sobic.001G230400 | 17.8895 | -1.8183 | 0.6772 | -2.6851 | 0.0072499 | 0.0903974 | no |
| Sobic.007G186200 | 471.9775 | 1.3689 | 0.5098 | 2.6850 | 0.0072527 | 0.0903974 | no |
| Sobic.007G068900 | 19055.3410 | -0.6802 | 0.2534 | -2.6845 | 0.0072643 | 0.0904884 | no |
| Sobic.006G003000 | 31.7795 | 1.6203 | 0.6037 | 2.6840 | 0.0072742 | 0.0905587 | no |
| Sobic.006G063900 | 4941.9575 | -0.9302 | 0.3467 | -2.6833 | 0.0072894 | 0.0906407 | no |
| Sobic.009G040800 | 261.1097 | 0.8882 | 0.3310 | 2.6833 | 0.0072894 | 0.0906407 | no |
| Sobic.003G164800 | 264.4319 | 1.4322 | 0.5338 | 2.6828 | 0.0073005 | 0.0907252 | no |
| Sobic.005G050300 | 76.2791 | 2.6046 | 0.9712 | 2.6819 | 0.0073204 | 0.0909192 | no |
| Sobic.001G527000 | 2114.6910 | -1.3699 | 0.5109 | -2.6813 | 0.0073337 | 0.0910199 | no |
| Sobic.004G358700 | 405.5157 | 1.2557 | 0.4684 | 2.6811 | 0.0073372 | 0.0910199 | no |
| Sobic.004G103400 | 46.0928 | 1.6386 | 0.6112 | 2.6808 | 0.0073446 | 0.0910581 | no |
| Sobic.002G290100 | 98.1553 | 1.4141 | 0.5276 | 2.6804 | 0.0073543 | 0.0911252 | no |
| Sobic.001G065900 | 426.3441 | 1.0848 | 0.4049 | 2.6791 | 0.0073818 | 0.0914118 | no |
| Sobic.006G061300 | 575.6173 | 0.8963 | 0.3346 | 2.6785 | 0.0073963 | 0.0915383 | no |
| Sobic.004G279200 | 246.9031 | 2.9731 | 1.1105 | 2.6773 | 0.0074228 | 0.0918119 | no |

| Sobic.001G157200 | 2518.9362 | -1.5365 | 0.5741 | -2.6766 | 0.0074382 | 0.0919482 | no |
| --- | --- | --- | --- | --- | --- | --- | --- |
| Sobic.005G217200 | 311.9992 | 0.9867 | 0.3687 | 2.6760 | 0.0074515 | 0.0920587 | no |
| Sobic.002G402366 | 856.9860 | -0.9016 | 0.3371 | -2.6744 | 0.0074857 | 0.0924068 | no |
| Sobic.004G236200 | 650.6297 | 0.8270 | 0.3092 | 2.6743 | 0.0074884 | 0.0924068 | no |
| Sobic.001G482800 | 20.2791 | 2.4566 | 0.9190 | 2.6732 | 0.0075138 | 0.0926663 | no |
| Sobic.K024000 | 19.3047 | 2.3646 | 0.8846 | 2.6729 | 0.0075197 | 0.0926839 | no |
| Sobic.001G297200 | 410.6430 | -2.1216 | 0.7939 | -2.6724 | 0.0075308 | 0.0927674 | no |
| Sobic.010G168400 | 46.6129 | -1.4235 | 0.5328 | -2.6719 | 0.0075433 | 0.0928666 | no |
| Sobic.001G508466 | 34.9187 | 1.8544 | 0.6942 | 2.6715 | 0.0075517 | 0.0929163 | no |
| Sobic.010G041800 | 131.0087 | 1.1848 | 0.4436 | 2.6710 | 0.0075626 | 0.0929259 | no |
| Sobic.010G246200 | 727.8334 | -0.7986 | 0.2990 | -2.6709 | 0.0075652 | 0.0929259 | no |
| Sobic.001G212000 | 13.2545 | 3.1079 | 1.1637 | 2.6707 | 0.0075686 | 0.0929259 | no |
| Sobic.005G093000 | 29.7718 | 1.6438 | 0.6155 | 2.6707 | 0.0075701 | 0.0929259 | no |
| Sobic.008G124800 | 1095.2209 | 0.6865 | 0.2571 | 2.6704 | 0.007577 | 0.0929559 | no |
| Sobic.001G524900 | 3429.3269 | -1.5045 | 0.5635 | -2.6700 | 0.0075862 | 0.0930142 | no |
| Sobic.008G116300 | 526.7849 | -0.8759 | 0.3281 | -2.6693 | 0.0076011 | 0.0931438 | no |
| Sobic.002G142700 | 297.2971 | 0.8049 | 0.3016 | 2.6689 | 0.0076093 | 0.0931828 | no |
| Sobic.003G011400 | 8.9016 | 4.4160 | 1.6548 | 2.6687 | 0.0076155 | 0.0931828 | no |
| Sobic.001G087700 | 8522.0119 | -1.0977 | 0.4114 | -2.6684 | 0.0076203 | 0.0931828 | no |
| Sobic.010G143500 | 1165.6472 | 2.1301 | 0.7983 | 2.6684 | 0.007622 | 0.0931828 | no |
| Sobic.010G076800 | 11959.2491 | -0.9488 | 0.3556 | -2.6678 | 0.0076349 | 0.0932579 | no |
| Sobic.001G338500 | 1213.8574 | -1.2974 | 0.4863 | -2.6677 | 0.007637 | 0.0932579 | no |
| Sobic.008G072600 | 1477.7240 | -1.2993 | 0.4871 | -2.6673 | 0.0076463 | 0.0933181 | no |
| Sobic.002G319100 | 627.4943 | -1.7729 | 0.6647 | -2.6670 | 0.0076526 | 0.0933408 | no |
| Sobic.003G228400 | 606.3582 | 2.7317 | 1.0245 | 2.6664 | 0.007667 | 0.093405 | no |
| Sobic.004G071800 | 861.4351 | -1.3754 | 0.5159 | -2.6662 | 0.0076712 | 0.093405 | no |
| Sobic.001G531700 | 250.4671 | -1.4319 | 0.5371 | -2.6660 | 0.0076755 | 0.093405 | no |
| Sobic.001G084300 | 43.7624 | -2.0872 | 0.7829 | -2.6658 | 0.0076795 | 0.093405 | no |
| Sobic.007G148000 | 7.6213 | -3.6459 | 1.3676 | -2.6658 | 0.00768 | 0.093405 | no |
| Sobic.001G259850 | 23.2272 | 2.7459 | 1.0302 | 2.6653 | 0.0076929 | 0.0934211 | no |
| Sobic.004G163400 | 150.8765 | 1.2295 | 0.4613 | 2.6653 | 0.007693 | 0.0934211 | no |
| Sobic.010G156000 | 1992.8930 | 1.0596 | 0.3976 | 2.6652 | 0.0076946 | 0.0934211 | no |
| Sobic.001G478800 | 233.6036 | 1.3777 | 0.5170 | 2.6647 | 0.0077049 | 0.0934921 | no |
| Sobic.009G001800 | 816.5897 | -1.4534 | 0.5455 | -2.6644 | 0.0077118 | 0.0935222 | no |
| Sobic.004G127600 | 2260.1027 | -1.8100 | 0.6797 | -2.6630 | 0.0077437 | 0.0938544 | no |
| Sobic.004G270300 | 44.0702 | 1.5213 | 0.5714 | 2.6624 | 0.0077587 | 0.0939829 | no |
| Sobic.003G132100 | 86562.3686 | -1.4712 | 0.5527 | -2.6621 | 0.0077666 | 0.0940239 | no |
| Sobic.005G096101 | 188.4633 | -1.8824 | 0.7072 | -2.6618 | 0.0077728 | 0.0940453 | no |
| Sobic.005G091500 | 53.2210 | 2.1365 | 0.8028 | 2.6612 | 0.0077863 | 0.0941541 | no |
| Sobic.007G089900 | 649.2752 | -1.1489 | 0.4318 | -2.6605 | 0.0078033 | 0.0943054 | no |
| Sobic.010G240300 | 39.7470 | 2.0615 | 0.7752 | 2.6592 | 0.0078326 | 0.0946055 | no |
| Sobic.004G044000 | 1244.3616 | -1.0404 | 0.3913 | -2.6588 | 0.0078421 | 0.0946661 | no |
| Sobic.001G078900 | 5010.6789 | -0.5434 | 0.2044 | -2.6585 | 0.0078493 | 0.0946992 | no |
| Sobic.009G164200 | 9.1785 | 4.0269 | 1.5149 | 2.6583 | 0.0078543 | 0.0947018 | no |
| Sobic.002G380400 | 869.3417 | -0.9575 | 0.3602 | -2.6581 | 0.0078585 | 0.0947018 | no |
| Sobic.002G261700 | 104.3884 | 1.0452 | 0.3932 | 2.6578 | 0.0078659 | 0.0947364 | no |
| Sobic.004G020300 | 15438.4880 | -1.2867 | 0.4845 | -2.6560 | 0.0079081 | 0.0951504 | no |
| Sobic.002G329000 | 44.5472 | -1.5391 | 0.5795 | -2.6559 | 0.0079093 | 0.0951504 | no |
| Sobic.003G284800 | 4881.1997 | -1.1053 | 0.4163 | -2.6547 | 0.0079368 | 0.0954276 | no |
| Sobic.007G076700 | 366.6173 | -1.6465 | 0.6203 | -2.6544 | 0.0079443 | 0.0954632 | no |
| Sobic.006G135300 | 42.5159 | -1.6577 | 0.6247 | -2.6537 | 0.0079608 | 0.0956068 | no |
| Sobic.008G191000 | 2101.6312 | -1.4651 | 0.5522 | -2.6531 | 0.007976 | 0.0957351 | no |
| Sobic.006G087200 | 1096.7733 | -1.0723 | 0.4042 | -2.6528 | 0.0079819 | 0.0957445 | no |
| Sobic.003G442700 | 1945.9806 | -1.2187 | 0.4594 | -2.6527 | 0.0079859 | 0.0957445 | no |
| Sobic.002G005600 | 81446.7941 | -0.6821 | 0.2572 | -2.6522 | 0.0079981 | 0.0958363 | no |
| Sobic.002G401900 | 5799.1801 | -1.0771 | 0.4062 | -2.6516 | 0.0080108 | 0.0958437 | no |
| Sobic.003G104000 | 1374.8911 | 0.8103 | 0.3056 | 2.6514 | 0.0080154 | 0.0958437 | no |

| Sobic.001G089600 | 630.4742 | 0.6194 | 0.2336 | 2.6513 | 0.0080173 | 0.0958437 | no |
| --- | --- | --- | --- | --- | --- | --- | --- |
| Sobic.002G125000 | 297.9756 | -1.3535 | 0.5105 | -2.6511 | 0.0080221 | 0.0958437 | no |
| Sobic.003G067201 | 78.8272 | 0.9912 | 0.3739 | 2.6511 | 0.0080229 | 0.0958437 | no |
| Sobic.003G097701 | 369.8758 | -1.1839 | 0.4466 | -2.6510 | 0.008026 | 0.0958437 | no |
| Sobic.001G235800 | 20.6634 | 4.8035 | 1.8134 | 2.6490 | 0.0080742 | 0.0963652 | no |
| Sobic.002G064100 | 6.3194 | -4.3937 | 1.6589 | -2.6486 | 0.0080835 | 0.0964212 | no |
| Sobic.002G369701 | 261.6459 | 1.0808 | 0.4082 | 2.6481 | 0.0080936 | 0.0964213 | no |
| Sobic.001G226600 | 113.1326 | 2.4628 | 0.9301 | 2.6480 | 0.0080968 | 0.0964213 | no |
| Sobic.009G129500 | 543.3036 | -1.0459 | 0.3950 | -2.6480 | 0.0080972 | 0.0964213 | no |
| Sobic.002G064400 | 441.7678 | -2.1760 | 0.8218 | -2.6477 | 0.0081046 | 0.096455 | no |
| Sobic.004G096300 | 5166.4762 | -1.5620 | 0.5900 | -2.6474 | 0.0081116 | 0.0964763 | no |
| Sobic.002G385800 | 828.2452 | -1.5180 | 0.5734 | -2.6472 | 0.0081156 | 0.0964763 | no |
| Sobic.001G450500 | 4634.3402 | -1.4766 | 0.5579 | -2.6467 | 0.008128 | 0.0965703 | no |
| Sobic.003G132500 | 128.6040 | 1.7701 | 0.6690 | 2.6458 | 0.0081498 | 0.0967429 | no |
| Sobic.010G184600 | 10312.8523 | -0.7729 | 0.2921 | -2.6457 | 0.0081517 | 0.0967429 | no |
| Sobic.003G227900 | 29.0419 | -3.0258 | 1.1438 | -2.6455 | 0.0081572 | 0.0967535 | no |
| Sobic.006G248801 | 106.3784 | -2.0891 | 0.7900 | -2.6444 | 0.0081831 | 0.0970053 | no |
| Sobic.007G169700 | 982.0344 | 0.7354 | 0.2781 | 2.6442 | 0.0081889 | 0.0970197 | no |
| Sobic.004G076000 | 834.0948 | -2.0083 | 0.7598 | -2.6432 | 0.0082114 | 0.0972325 | no |
| Sobic.001G043100 | 752.1277 | 0.8097 | 0.3064 | 2.6426 | 0.0082279 | 0.097373 | no |
| Sobic.001G468700 | 2261.1834 | -1.0141 | 0.3839 | -2.6417 | 0.0082502 | 0.0975824 | no |
| Sobic.010G133400 | 949.6481 | 0.6315 | 0.2391 | 2.6414 | 0.0082561 | 0.0975969 | no |
| Sobic.007G002800 | 85.3648 | 1.4736 | 0.5579 | 2.6411 | 0.0082639 | 0.0976346 | no |
| Sobic.006G228833 | 126.2385 | 0.9629 | 0.3647 | 2.6405 | 0.0082792 | 0.0977476 | no |
| Sobic.003G030300 | 1214.1444 | -1.1240 | 0.4257 | -2.6403 | 0.0082843 | 0.0977476 | no |
| Sobic.001G519700 | 927.1221 | -4.3251 | 1.6382 | -2.6401 | 0.0082874 | 0.0977476 | no |
| Sobic.004G087700 | 1522.6172 | 1.2857 | 0.4871 | 2.6394 | 0.0083063 | 0.0979159 | no |
| Sobic.005G224500 | 616.8648 | 1.9440 | 0.7369 | 2.6382 | 0.0083357 | 0.0981337 | no |
| Sobic.009G197000 | 94.9392 | -3.3727 | 1.2785 | -2.6381 | 0.0083372 | 0.0981337 | no |
| Sobic.004G169600 | 144.7360 | 1.1052 | 0.4189 | 2.6379 | 0.008341 | 0.0981337 | no |
| Sobic.002G420700 | 569.7813 | 2.5569 | 0.9693 | 2.6378 | 0.0083434 | 0.0981337 | no |
| Sobic.001G414650 | 84.4521 | -1.1892 | 0.4509 | -2.6373 | 0.0083574 | 0.0982436 | no |
| Sobic.001G478400 | 4940.6767 | -0.7960 | 0.3019 | -2.6365 | 0.0083757 | 0.0984044 | no |
| Sobic.006G165501 | 202.1280 | -1.1916 | 0.4520 | -2.6362 | 0.008384 | 0.0984461 | no |
| Sobic.004G314500 | 996.3332 | 0.5868 | 0.2226 | 2.6355 | 0.0084002 | 0.098554 | no |
| Sobic.010G255600 | 2356.8837 | -1.3316 | 0.5053 | -2.6354 | 0.0084038 | 0.098554 | no |
| Sobic.001G527100 | 3396.8863 | -1.4064 | 0.5337 | -2.6353 | 0.0084072 | 0.098554 | no |
| Sobic.003G433900 | 8046.1643 | -1.1619 | 0.4410 | -2.6350 | 0.0084148 | 0.098589 | no |
| Sobic.010G262200 | 673.8861 | -1.2834 | 0.4873 | -2.6339 | 0.00844 | 0.0987861 | no |
| Sobic.003G309900 | 1128.2284 | -0.9863 | 0.3745 | -2.6339 | 0.008441 | 0.0987861 | no |
| Sobic.009G237000 | 8896.8860 | -1.1010 | 0.4181 | -2.6333 | 0.0084552 | 0.0988203 | no |
| Sobic.007G043000 | 25.8154 | 2.7547 | 1.0461 | 2.6333 | 0.0084562 | 0.0988203 | no |
| Sobic.008G116400 | 635.2669 | -1.1557 | 0.4389 | -2.6331 | 0.0084621 | 0.0988203 | no |
| Sobic.010G180100 | 28.2985 | 1.8909 | 0.7182 | 2.6330 | 0.0084627 | 0.0988203 | no |
| Sobic.004G341700 | 1274.6365 | 1.1814 | 0.4489 | 2.6320 | 0.0084872 | 0.0990513 | no |
| Sobic.005G171800 | 34.0282 | 1.7959 | 0.6824 | 2.6317 | 0.0084964 | 0.0991021 | no |
| Sobic.001G361700 | 1104.9082 | -1.2103 | 0.4599 | -2.6315 | 0.0085009 | 0.0991021 | no |
| Sobic.008G000600 | 37.6010 | 1.6181 | 0.6152 | 2.6302 | 0.0085329 | 0.0994203 | no |
| Sobic.006G254500 | 816.6470 | 0.9999 | 0.3802 | 2.6300 | 0.0085395 | 0.0994416 | no |
| Sobic.003G080400 | 35.2036 | 4.0653 | 1.5464 | 2.6289 | 0.0085661 | 0.0996965 | no |
| Sobic.001G075600 | 45.1118 | 1.9752 | 0.7515 | 2.6282 | 0.0085829 | 0.0998102 | no |
| Sobic.010G091500 | 128.3797 | 1.0382 | 0.3950 | 2.6281 | 0.0085853 | 0.0998102 | no |
| Sobic.002G380150 | 19.1354 | 2.1508 | 0.8184 | 2.6279 | 0.0085903 | 0.0998128 | no |
| Sobic.010G159400 | 1998.9351 | -1.1648 | 0.4434 | -2.6270 | 0.0086148 | 0.1000078 | no |
| Sobic.010G136500 | 84.3214 | -2.3436 | 0.8921 | -2.6269 | 0.0086165 | 0.1000078 | no |
| Sobic.002G426400 | 352.6420 | 1.2249 | 0.4663 | 2.6267 | 0.0086225 | 0.1000224 | no |
| Sobic.003G114100 | 2700.2832 | -1.2670 | 0.4824 | -2.6263 | 0.0086316 | 0.1000381 | no |

| Sobic.006G151600 | 376.6122 | -1.0350 | 0.3941 | -2.6262 | 0.0086339 | 0.1000381 | no |
| --- | --- | --- | --- | --- | --- | --- | --- |
| Sobic.009G009500 | 16468.9208 | -0.9956 | 0.3791 | -2.6260 | 0.0086399 | 0.1000381 | no |
| Sobic.003G062300 | 1059.6196 | 0.7047 | 0.2684 | 2.6259 | 0.0086429 | 0.1000381 | no |
| Sobic.002G362200 | 83.2212 | 1.1164 | 0.4253 | 2.6251 | 0.0086628 | 0.1002143 | no |
| Sobic.007G181400 | 1750.9125 | -1.3433 | 0.5119 | -2.6242 | 0.0086848 | 0.1004127 | no |
| Sobic.006G218700 | 785.1559 | 0.7934 | 0.3025 | 2.6230 | 0.0087165 | 0.100724 | no |
| Sobic.002G381100 | 200.9998 | -1.2682 | 0.4837 | -2.6217 | 0.0087503 | 0.1010597 | no |
| Sobic.006G060700 | 358.9274 | 1.4767 | 0.5634 | 2.6210 | 0.0087669 | 0.1011877 | no |
| Sobic.001G291100 | 2892.2043 | -0.7944 | 0.3031 | -2.6207 | 0.0087749 | 0.1011877 | no |
| Sobic.001G037100 | 1290.3466 | 1.0501 | 0.4008 | 2.6204 | 0.0087824 | 0.1011877 | no |
| Sobic.010G061300 | 86.1100 | 1.4239 | 0.5434 | 2.6203 | 0.0087853 | 0.1011877 | no |
| Sobic.006G006700 | 93.8198 | 2.5236 | 0.9632 | 2.6201 | 0.0087894 | 0.1011877 | no |
| Sobic.010G090500 | 354.9070 | 1.1572 | 0.4417 | 2.6198 | 0.0087985 | 0.1011877 | no |
| Sobic.003G013600 | 10796.7329 | -2.4447 | 0.9332 | -2.6197 | 0.0088018 | 0.1011877 | no |
| Sobic.001G475600 | 565.2959 | -1.5589 | 0.5951 | -2.6197 | 0.008802 | 0.1011877 | no |
| Sobic.006G161400 | 533.3461 | 0.7173 | 0.2738 | 2.6196 | 0.0088046 | 0.1011877 | no |
| Sobic.002G239450 | 17.3936 | -2.6209 | 1.0007 | -2.6192 | 0.0088141 | 0.1012422 | no |
| Sobic.001G430900 | 85.2760 | 1.7009 | 0.6496 | 2.6183 | 0.0088381 | 0.1014502 | no |
| Sobic.008G067200 | 128.3453 | -1.3555 | 0.5177 | -2.6181 | 0.0088418 | 0.1014502 | no |
| Sobic.004G220300 | 627.6463 | 1.3640 | 0.5211 | 2.6178 | 0.008851 | 0.1015004 | no |
| Sobic.001G072800 | 1506.8385 | -0.6516 | 0.2490 | -2.6169 | 0.0088723 | 0.101663 | no |
| Sobic.006G024000 | 72.3167 | 2.3770 | 0.9084 | 2.6168 | 0.0088748 | 0.101663 | no |
| Sobic.010G132300 | 633.2257 | 0.6425 | 0.2456 | 2.6164 | 0.0088861 | 0.1017364 | no |
| Sobic.007G136900 | ######### | -1.7958 | 0.6865 | -2.6159 | 0.0088985 | 0.1018236 | no |
| Sobic.006G197800 | 3931.3243 | -1.3651 | 0.5219 | -2.6155 | 0.0089095 | 0.1018938 | no |
| Sobic.001G338700 | 705.0670 | -1.6981 | 0.6493 | -2.6152 | 0.0089188 | 0.1019448 | no |
| Sobic.008G055000 | 33.5550 | -1.8133 | 0.6936 | -2.6144 | 0.0089373 | 0.1020109 | no |
| Sobic.006G281600 | 115.8119 | 1.4536 | 0.5560 | 2.6144 | 0.0089389 | 0.1020109 | no |
| Sobic.003G187000 | 16019.3486 | -1.2413 | 0.4748 | -2.6143 | 0.0089403 | 0.1020109 | no |
| Sobic.010G236900 | 5522.3513 | -0.8584 | 0.3283 | -2.6142 | 0.0089439 | 0.1020109 | no |
| Sobic.009G241700 | 562.4282 | 1.4585 | 0.5581 | 2.6134 | 0.0089639 | 0.1021837 | no |
| Sobic.001G121700 | 213.4509 | -1.1374 | 0.4353 | -2.6130 | 0.0089762 | 0.1022205 | no |
| Sobic.002G339600 | 49307.0666 | -1.2388 | 0.4741 | -2.6129 | 0.0089768 | 0.1022205 | no |
| Sobic.007G133900 | 58.7740 | -2.6881 | 1.0291 | -2.6121 | 0.0089976 | 0.1024013 | no |
| Sobic.002G385700 | 225.0516 | -1.1302 | 0.4327 | -2.6119 | 0.0090041 | 0.1024209 | no |
| Sobic.004G102200 | 48.3256 | 2.1570 | 0.8260 | 2.6114 | 0.0090165 | 0.1025064 | no |
| Sobic.003G246400 | 10077.8699 | -1.3189 | 0.5053 | -2.6102 | 0.0090481 | 0.1028096 | no |
| Sobic.009G177400 | 182.0992 | 1.6967 | 0.6501 | 2.6097 | 0.0090609 | 0.1029001 | no |
| Sobic.002G105700 | 121.4706 | 1.4087 | 0.5399 | 2.6093 | 0.0090719 | 0.1029548 | no |
| Sobic.007G143700 | 778.5000 | -1.2426 | 0.4762 | -2.6092 | 0.0090755 | 0.1029548 | no |
| Sobic.002G409700 | 217.4197 | 1.1227 | 0.4306 | 2.6073 | 0.0091262 | 0.1034749 | no |
| Sobic.007G146600 | 543.3377 | 0.8282 | 0.3177 | 2.6070 | 0.0091337 | 0.1035039 | no |
| Sobic.006G149000 | 136.3719 | -1.4622 | 0.5610 | -2.6065 | 0.0091486 | 0.1035825 | no |
| Sobic.001G443200 | 4346.7629 | 0.6224 | 0.2388 | 2.6064 | 0.0091505 | 0.1035825 | no |
| Sobic.003G380600 | 1183.0784 | 1.3247 | 0.5083 | 2.6061 | 0.0091587 | 0.10362 | no |
| Sobic.002G041533 | 2695.7998 | -0.7137 | 0.2739 | -2.6056 | 0.0091702 | 0.1036674 | no |
| Sobic.010G078300 | 182.1109 | -1.0302 | 0.3954 | -2.6055 | 0.0091727 | 0.1036674 | no |
| Sobic.007G163500 | 23.7257 | 2.1524 | 0.8262 | 2.6053 | 0.0091805 | 0.1036995 | no |
| Sobic.002G000400 | 445.6046 | 2.1916 | 0.8416 | 2.6041 | 0.009212 | 0.1039998 | no |
| Sobic.002G267500 | 883.9881 | 0.7553 | 0.2901 | 2.6039 | 0.0092176 | 0.1039998 | no |
| Sobic.009G055600 | 393.0323 | 0.8778 | 0.3371 | 2.6037 | 0.0092219 | 0.1039998 | no |
| Sobic.005G018200 | 230.5248 | 1.4494 | 0.5567 | 2.6034 | 0.0092315 | 0.1040287 | no |
| Sobic.001G063000 | 81.3399 | 1.1724 | 0.4504 | 2.6033 | 0.0092343 | 0.1040287 | no |
| Sobic.003G345500 | 5122.9391 | -0.8674 | 0.3333 | -2.6026 | 0.009251 | 0.1041375 | no |
| Sobic.004G015900 | 5.0607 | 4.5333 | 1.7419 | 2.6025 | 0.0092538 | 0.1041375 | no |
| Sobic.007G019300 | 399.8662 | 0.7297 | 0.2804 | 2.6020 | 0.009269 | 0.1042031 | no |
| Sobic.009G171400 | 935.4130 | -1.7315 | 0.6655 | -2.6019 | 0.0092703 | 0.1042031 | no |

| Sobic.004G006300 | 25084.0082 | -1.0707 | 0.4115 | -2.6018 | 0.0092745 | 0.1042031 | no |
| --- | --- | --- | --- | --- | --- | --- | --- |
| Sobic.002G351400 | 3415.1683 | 1.8204 | 0.6999 | 2.6007 | 0.0093025 | 0.1044622 | no |
| Sobic.003G091200 | 21.1504 | -2.2594 | 0.8689 | -2.6004 | 0.0093125 | 0.1045192 | no |
| Sobic.003G312300 | 111.4930 | 1.2375 | 0.4761 | 2.5992 | 0.0093447 | 0.1048246 | no |
| Sobic.005G099700 | 1607.4383 | -1.7812 | 0.6854 | -2.5986 | 0.0093606 | 0.1049302 | no |
| Sobic.002G321300 | 2361.2981 | 0.9944 | 0.3827 | 2.5985 | 0.0093641 | 0.1049302 | no |
| Sobic.007G152600 | 3381.9102 | -0.6145 | 0.2366 | -2.5977 | 0.0093858 | 0.1051145 | no |
| Sobic.002G147100 | 176.9660 | 1.3314 | 0.5126 | 2.5975 | 0.0093905 | 0.1051145 | no |
| Sobic.002G296800 | 61.8113 | 1.2016 | 0.4627 | 2.5968 | 0.0094101 | 0.1052781 | no |
| Sobic.003G399000 | 440.3948 | -0.9761 | 0.3760 | -2.5960 | 0.009432 | 0.1054198 | no |
| Sobic.007G204100 | 1681.1655 | -2.0508 | 0.7900 | -2.5960 | 0.0094327 | 0.1054198 | no |
| Sobic.010G153900 | 5005.0223 | -1.0554 | 0.4066 | -2.5955 | 0.0094459 | 0.1054777 | no |
| Sobic.008G138600 | 1051.6372 | 0.8958 | 0.3451 | 2.5954 | 0.0094479 | 0.1054777 | no |
| Sobic.002G301700 | 929.4877 | -1.0175 | 0.3922 | -2.5944 | 0.0094746 | 0.1055977 | no |
| Sobic.007G163901 | 33.5966 | -1.4665 | 0.5653 | -2.5943 | 0.009478 | 0.1055977 | no |
| Sobic.004G101400 | 86.8985 | 3.1788 | 1.2253 | 2.5942 | 0.0094803 | 0.1055977 | no |
| Sobic.003G276000 | 49.1229 | 1.8559 | 0.7155 | 2.5939 | 0.0094889 | 0.1055977 | no |
| Sobic.004G249800 | 10360.2447 | -1.0331 | 0.3983 | -2.5939 | 0.0094899 | 0.1055977 | no |
| Sobic.004G329400 | 1385.1680 | 0.9245 | 0.3564 | 2.5937 | 0.0094961 | 0.1055977 | no |
| Sobic.008G127300 | 15.5948 | 3.3808 | 1.3036 | 2.5935 | 0.0095001 | 0.1055977 | no |
| Sobic.005G131700 | 108.7806 | 2.2248 | 0.8578 | 2.5935 | 0.0095002 | 0.1055977 | no |
| Sobic.003G270300 | 29.4490 | 2.9886 | 1.1524 | 2.5934 | 0.0095037 | 0.1055977 | no |
| Sobic.002G311501 | 168.3056 | 1.3896 | 0.5359 | 2.5928 | 0.0095187 | 0.1057079 | no |
| Sobic.010G119500 | 321.3013 | 1.2557 | 0.4843 | 2.5926 | 0.0095264 | 0.1057384 | no |
| Sobic.001G491000 | 17059.7339 | -1.1240 | 0.4336 | -2.5921 | 0.0095387 | 0.1058193 | no |
| Sobic.010G003700 | 164.0434 | -0.8974 | 0.3463 | -2.5916 | 0.0095522 | 0.1059105 | no |
| Sobic.003G116850 | 35.1425 | -1.6016 | 0.6180 | -2.5915 | 0.0095572 | 0.1059105 | no |
| Sobic.003G273700 | 286.3492 | 0.8711 | 0.3362 | 2.5913 | 0.009562 | 0.1059105 | no |
| Sobic.008G036000 | 67.3775 | 2.5957 | 1.0020 | 2.5904 | 0.0095876 | 0.1061384 | no |
| Sobic.003G259200 | 50.9499 | 1.3726 | 0.5299 | 2.5901 | 0.0095934 | 0.1061467 | no |
| Sobic.002G362000 | 98.4389 | 2.1405 | 0.8266 | 2.5896 | 0.0096094 | 0.1062683 | no |
| Sobic.010G178700 | 250.3001 | -1.3813 | 0.5337 | -2.5879 | 0.0096573 | 0.1067417 | no |
| Sobic.004G230900 | 137.2326 | 1.7466 | 0.6752 | 2.5867 | 0.0096911 | 0.1070592 | no |
| Sobic.009G114000 | 1908.9413 | 1.9124 | 0.7397 | 2.5854 | 0.0097268 | 0.1073515 | no |
| Sobic.009G154900 | 445.3269 | 1.0732 | 0.4151 | 2.5854 | 0.0097278 | 0.1073515 | no |
| Sobic.004G344901 | 672.4850 | 0.9354 | 0.3619 | 2.5846 | 0.0097478 | 0.1075163 | no |
| Sobic.001G497500 | 572.9335 | -1.1286 | 0.4367 | -2.5842 | 0.009761 | 0.1075998 | no |
| Sobic.001G025400 | 3659.7519 | -1.0191 | 0.3944 | -2.5840 | 0.0097656 | 0.1075998 | no |
| Sobic.007G035000 | 182.8025 | 5.7732 | 2.2352 | 2.5829 | 0.0097978 | 0.1078989 | no |
| Sobic.003G034400 | 1679.8404 | -1.1470 | 0.4442 | -2.5824 | 0.0098116 | 0.1079941 | no |
| Sobic.003G375000 | 371.3422 | -1.1685 | 0.4526 | -2.5817 | 0.0098305 | 0.1081401 | no |
| Sobic.009G257100 | 45.8125 | 1.4346 | 0.5557 | 2.5814 | 0.0098391 | 0.1081401 | no |
| Sobic.003G403800 | 1728.8736 | -1.6217 | 0.6282 | -2.5814 | 0.0098402 | 0.1081401 | no |
| Sobic.008G095400 | 296.5556 | -0.8499 | 0.3293 | -2.5809 | 0.0098542 | 0.1082369 | no |
| Sobic.007G134700 | 9.6388 | 2.8142 | 1.0911 | 2.5791 | 0.0099047 | 0.1087283 | no |
| Sobic.003G174301 | 62.9418 | 1.6821 | 0.6522 | 2.5790 | 0.0099093 | 0.1087283 | no |
| Sobic.003G130400 | 12.3648 | 3.9866 | 1.5459 | 2.5788 | 0.0099144 | 0.1087283 | no |
| Sobic.008G187200 | 8.0429 | -2.8523 | 1.1064 | -2.5781 | 0.0099339 | 0.1088855 | no |
| Sobic.006G215800 | 930.5156 | -1.3956 | 0.5414 | -2.5776 | 0.0099485 | 0.1089886 | no |
| Sobic.008G029200 | 90.4948 | 1.1174 | 0.4337 | 2.5768 | 0.0099724 | 0.1091942 | no |
| Sobic.002G039800 | 401.8843 | 0.6334 | 0.2459 | 2.5758 | 0.010002 | 0.1094609 | no |
| Sobic.002G134400 | 1049.1553 | 0.8144 | 0.3164 | 2.5739 | 0.010057 | 0.1100058 | no |
| Sobic.008G092600 | 4526.4278 | 1.0597 | 0.4118 | 2.5733 | 0.0100733 | 0.1101274 | no |
| Sobic.009G030800 | 1235.2109 | -0.8298 | 0.3225 | -2.5730 | 0.0100834 | 0.1101804 | no |
| Sobic.002G078800 | 24.5211 | -2.5737 | 1.0004 | -2.5726 | 0.010094 | 0.1102396 | no |
| Sobic.010G005500 | 46036.0155 | -1.2572 | 0.4888 | -2.5722 | 0.0101062 | 0.110316 | no |
| Sobic.010G218500 | 2290.3955 | -1.6393 | 0.6375 | -2.5716 | 0.0101226 | 0.1104376 | no |

| Sobic.007G107100 | 875.8808 | -0.6428 | 0.2500 | -2.5714 | 0.0101283 | 0.1104428 | no |
| --- | --- | --- | --- | --- | --- | --- | --- |
| Sobic.002G241900 | 15.1707 | -1.8097 | 0.7040 | -2.5706 | 0.0101509 | 0.1106177 | no |
| Sobic.009G083100 | 678.1448 | 0.9517 | 0.3702 | 2.5705 | 0.0101549 | 0.1106177 | no |
| Sobic.004G134100 | 354.2378 | 1.1651 | 0.4534 | 2.5696 | 0.0101825 | 0.1108611 | no |
| Sobic.001G499600 | 266.8706 | -1.1787 | 0.4590 | -2.5681 | 0.0102244 | 0.1112163 | no |
| Sobic.003G134400 | 5191.8811 | -1.3023 | 0.5071 | -2.5680 | 0.0102279 | 0.1112163 | no |
| Sobic.003G174700 | 15.5438 | 2.0719 | 0.8068 | 2.5679 | 0.0102309 | 0.1112163 | no |
| Sobic.001G179900 | 403.6604 | 1.0785 | 0.4201 | 2.5670 | 0.0102574 | 0.1114472 | no |
| Sobic.010G225600 | 316.3738 | -2.2374 | 0.8717 | -2.5667 | 0.0102672 | 0.1114631 | no |
| Sobic.001G065800 | 3526.2850 | 0.8835 | 0.3442 | 2.5666 | 0.0102695 | 0.1114631 | no |
| Sobic.007G210801 | 249.4082 | 1.6235 | 0.6328 | 2.5657 | 0.0102955 | 0.1116877 | no |
| Sobic.004G072000 | 8441.1614 | -1.3120 | 0.5114 | -2.5653 | 0.0103099 | 0.1117868 | no |
| Sobic.004G216700 | 1257.1199 | 2.1107 | 0.8232 | 2.5641 | 0.0103432 | 0.1120248 | no |
| Sobic.002G047600 | 8871.1066 | -0.7278 | 0.2839 | -2.5640 | 0.0103472 | 0.1120248 | no |
| Sobic.002G190200 | 163.1603 | -2.5432 | 0.9919 | -2.5640 | 0.0103478 | 0.1120248 | no |
| Sobic.002G066901 | 312.7270 | 0.7915 | 0.3088 | 2.5633 | 0.0103692 | 0.1121475 | no |
| Sobic.001G341900 | 57.1657 | 1.3173 | 0.5139 | 2.5632 | 0.0103697 | 0.1121475 | no |
| Sobic.003G246200 | 1360.2972 | -0.9045 | 0.3529 | -2.5626 | 0.0103903 | 0.1122977 | no |
| Sobic.001G130700 | 228.6615 | -1.1725 | 0.4576 | -2.5623 | 0.0103982 | 0.1122977 | no |
| Sobic.006G138000 | 1052.2979 | 0.9226 | 0.3601 | 2.5620 | 0.0104059 | 0.1122977 | no |
| Sobic.010G066601 | 41.1738 | 2.9529 | 1.1526 | 2.5619 | 0.0104102 | 0.1122977 | no |
| Sobic.002G098800 | 764.2541 | -0.8596 | 0.3355 | -2.5619 | 0.0104103 | 0.1122977 | no |
| Sobic.002G081500 | 662.5680 | -1.1656 | 0.4550 | -2.5615 | 0.0104207 | 0.1123419 | no |
| Sobic.009G132900 | ######### | -1.2296 | 0.4801 | -2.5613 | 0.0104278 | 0.1123419 | no |
| Sobic.004G106900 | 5514.1882 | 1.7925 | 0.6999 | 2.5611 | 0.0104354 | 0.1123419 | no |
| Sobic.006G264201 | 42728.8452 | -1.9419 | 0.7583 | -2.5609 | 0.0104388 | 0.1123419 | no |
| Sobic.006G222500 | 725.8733 | 0.9660 | 0.3772 | 2.5609 | 0.0104412 | 0.1123419 | no |
| Sobic.010G276600 | 313.9891 | -0.9362 | 0.3656 | -2.5607 | 0.0104463 | 0.1123419 | no |
| Sobic.001G078200 | 5972.4834 | -1.5138 | 0.5913 | -2.5601 | 0.0104653 | 0.1124884 | no |
| Sobic.003G107900 | 9.6511 | -3.1371 | 1.2257 | -2.5593 | 0.0104875 | 0.112631 | no |
| Sobic.004G201800 | 1418.8311 | -0.9977 | 0.3898 | -2.5593 | 0.0104896 | 0.112631 | no |
| Sobic.001G058300 | 52.3383 | 1.1177 | 0.4367 | 2.5591 | 0.0104946 | 0.112631 | no |
| Sobic.009G167100 | 386.1927 | 1.0656 | 0.4165 | 2.5585 | 0.0105129 | 0.1127148 | no |
| Sobic.001G116000 | 515.0391 | 1.1534 | 0.4508 | 2.5585 | 0.0105131 | 0.1127148 | no |
| Sobic.003G019700 | 890.6335 | 1.9157 | 0.7489 | 2.5579 | 0.0105319 | 0.1128591 | no |
| Sobic.004G234200 | 7798.4398 | -1.2173 | 0.4760 | -2.5574 | 0.0105451 | 0.1129437 | no |
| Sobic.009G204601 | 3719.0957 | 0.5910 | 0.2311 | 2.5567 | 0.0105665 | 0.1131149 | no |
| Sobic.001G540600 | 3265.2839 | -0.9634 | 0.3768 | -2.5564 | 0.0105752 | 0.1131512 | no |
| Sobic.006G175900 | 224.6116 | -0.9486 | 0.3711 | -2.5559 | 0.0105902 | 0.1132536 | no |
| Sobic.010G077400 | 1547.0344 | -0.7227 | 0.2828 | -2.5557 | 0.010596 | 0.1132582 | no |
| Sobic.010G122200 | 87.6065 | 1.0542 | 0.4126 | 2.5551 | 0.0106172 | 0.1133773 | no |
| Sobic.009G193300 | 264.7925 | 0.6965 | 0.2726 | 2.5548 | 0.0106262 | 0.1133773 | no |
| Sobic.001G038900 | 70.8778 | 1.3517 | 0.5291 | 2.5547 | 0.0106281 | 0.1133773 | no |
| Sobic.005G099000 | 29.3356 | 3.9704 | 1.5542 | 2.5547 | 0.0106286 | 0.1133773 | no |
| Sobic.007G204600 | 510.5423 | 1.4334 | 0.5612 | 2.5542 | 0.0106429 | 0.1134198 | no |
| Sobic.009G126300 | 930.0789 | 0.6986 | 0.2735 | 2.5541 | 0.0106462 | 0.1134198 | no |
| Sobic.001G416600 | 1052.1302 | 0.7612 | 0.2981 | 2.5540 | 0.0106489 | 0.1134198 | no |
| Sobic.006G099900 | 29.5467 | 2.8275 | 1.1072 | 2.5538 | 0.0106541 | 0.1134198 | no |
| Sobic.010G011700 | 1722.3667 | -1.2166 | 0.4766 | -2.5530 | 0.0106807 | 0.1136114 | no |
| Sobic.001G065000 | 1394.4098 | 0.9083 | 0.3558 | 2.5529 | 0.0106829 | 0.1136114 | no |
| Sobic.004G288300 | 140.3739 | -1.5448 | 0.6053 | -2.5522 | 0.0107043 | 0.1137815 | no |
| Sobic.002G214900 | 756.7031 | 0.6160 | 0.2414 | 2.5519 | 0.0107122 | 0.1137904 | no |
| Sobic.003G029700 | 1507.9375 | 0.7159 | 0.2806 | 2.5517 | 0.0107188 | 0.1137904 | no |
| Sobic.001G405700 | 7289.2850 | -1.1311 | 0.4433 | -2.5517 | 0.0107213 | 0.1137904 | no |
| Sobic.002G262800 | 299.6559 | 1.2631 | 0.4954 | 2.5497 | 0.0107825 | 0.1142798 | no |
| Sobic.006G159800 | 406.3643 | 1.2261 | 0.4810 | 2.5493 | 0.010793 | 0.1142798 | no |
| Sobic.002G147600 | 3015.2607 | 1.2990 | 0.5096 | 2.5493 | 0.0107949 | 0.1142798 | no |

| Sobic.002G131900 | 1088.8566 | 0.6495 | 0.2548 | 2.5492 | 0.0107966 | 0.1142798 | no |
| --- | --- | --- | --- | --- | --- | --- | --- |
| Sobic.003G349600 | 53.9608 | 2.1754 | 0.8535 | 2.5489 | 0.0108058 | 0.1142798 | no |
| Sobic.001G515600 | 2276.0382 | -1.7449 | 0.6846 | -2.5488 | 0.0108081 | 0.1142798 | no |
| Sobic.004G142900 | 3201.9753 | -0.8997 | 0.3530 | -2.5488 | 0.0108094 | 0.1142798 | no |
| Sobic.007G171600 | 313.0119 | -0.9154 | 0.3591 | -2.5488 | 0.0108108 | 0.1142798 | no |
| Sobic.006G280050 | 13476.0548 | -1.2023 | 0.4718 | -2.5484 | 0.010822 | 0.1143408 | no |
| Sobic.003G223900 | 402.3295 | 0.7614 | 0.2988 | 2.5477 | 0.0108427 | 0.1145023 | no |
| Sobic.001G012500 | 7255.2610 | -1.4167 | 0.5562 | -2.5469 | 0.0108683 | 0.1147157 | no |
| Sobic.007G188200 | 202.9003 | 0.8770 | 0.3444 | 2.5466 | 0.0108763 | 0.1147424 | no |
| Sobic.005G008400 | 23.6446 | 1.8717 | 0.7351 | 2.5463 | 0.0108877 | 0.1148017 | no |
| Sobic.008G062500 | 1192.9490 | -1.0542 | 0.4140 | -2.5461 | 0.0108928 | 0.1148017 | no |
| Sobic.004G050200 | 350.5196 | 2.7952 | 1.0980 | 2.5457 | 0.0109055 | 0.1148779 | no |
| Sobic.005G192600 | 1111.3779 | -1.6435 | 0.6459 | -2.5445 | 0.0109445 | 0.1151641 | no |
| Sobic.006G105600 | 7.4610 | 3.5232 | 1.3848 | 2.5443 | 0.0109499 | 0.1151641 | no |
| Sobic.002G293000 | 9741.0347 | 0.6227 | 0.2447 | 2.5442 | 0.0109538 | 0.1151641 | no |
| Sobic.001G131900 | 59.1155 | 1.4608 | 0.5742 | 2.5441 | 0.0109569 | 0.1151641 | no |
| Sobic.004G235700 | 2098.2984 | -0.6884 | 0.2706 | -2.5440 | 0.01096 | 0.1151641 | no |
| Sobic.004G213100 | 7945.2705 | 1.5413 | 0.6059 | 2.5437 | 0.0109695 | 0.1152066 | no |
| Sobic.002G315100 | 162.0363 | 0.9672 | 0.3803 | 2.5434 | 0.0109777 | 0.1152358 | no |
| Sobic.006G025400 | 417.4591 | -2.3023 | 0.9053 | -2.5432 | 0.0109838 | 0.1152429 | no |
| Sobic.009G022200 | 6107.9751 | -0.7587 | 0.2984 | -2.5429 | 0.0109921 | 0.1152724 | no |
| Sobic.010G130601 | 591.7743 | -1.0665 | 0.4195 | -2.5423 | 0.0110124 | 0.1153079 | no |
| Sobic.004G268000 | 4644.9997 | -1.1474 | 0.4514 | -2.5422 | 0.0110171 | 0.1153079 | no |
| Sobic.003G156600 | 300.1739 | -1.6143 | 0.6351 | -2.5419 | 0.0110244 | 0.1153079 | no |
| Sobic.005G169200 | 5195.4101 | 1.3306 | 0.5235 | 2.5417 | 0.0110305 | 0.1153079 | no |
| Sobic.007G149000 | 90.2715 | -2.3813 | 0.9369 | -2.5417 | 0.0110315 | 0.1153079 | no |
| Sobic.003G274700 | 1935.8453 | -1.3245 | 0.5212 | -2.5414 | 0.0110399 | 0.1153079 | no |
| Sobic.007G186400 | 22.1632 | 1.7840 | 0.7020 | 2.5414 | 0.0110409 | 0.1153079 | no |
| Sobic.002G189100 | 1201.3230 | 0.8965 | 0.3527 | 2.5414 | 0.0110416 | 0.1153079 | no |
| Sobic.003G005900 | 1481.5789 | 0.6294 | 0.2477 | 2.5413 | 0.0110447 | 0.1153079 | no |
| Sobic.008G060300 | 3404.7338 | 1.6184 | 0.6371 | 2.5405 | 0.0110707 | 0.1155225 | no |
| Sobic.006G220800 | 7257.8403 | -0.8298 | 0.3267 | -2.5401 | 0.0110816 | 0.1155784 | no |
| Sobic.009G030900 | 2334.7847 | -0.9243 | 0.3639 | -2.5398 | 0.0110931 | 0.1156409 | no |
| Sobic.002G324500 | 6337.8743 | -1.5848 | 0.6242 | -2.5390 | 0.0111164 | 0.1158275 | no |
| Sobic.010G150800 | 3410.1658 | -1.0001 | 0.3940 | -2.5385 | 0.0111328 | 0.1159408 | no |
| Sobic.010G249600 | 271.5366 | -1.5390 | 0.6064 | -2.5378 | 0.0111537 | 0.1161009 | no |
| Sobic.009G104800 | 7205.1255 | -1.7330 | 0.6830 | -2.5373 | 0.0111712 | 0.1162256 | no |
| Sobic.003G234200 | ######### | -1.2208 | 0.4813 | -2.5366 | 0.0111928 | 0.1163695 | no |
| Sobic.004G314000 | 229.3588 | 1.0400 | 0.4100 | 2.5365 | 0.0111961 | 0.1163695 | no |
| Sobic.003G408400 | 2962.4879 | -1.2583 | 0.4962 | -2.5360 | 0.0112124 | 0.1164815 | no |
| Sobic.010G028600 | 5382.7661 | 1.1937 | 0.4707 | 2.5358 | 0.0112204 | 0.1165076 | no |
| Sobic.006G037900 | 44.1431 | -1.4192 | 0.5597 | -2.5356 | 0.0112265 | 0.1165141 | no |
| Sobic.002G095900 | 694.7386 | -0.8149 | 0.3214 | -2.5353 | 0.0112349 | 0.1165429 | no |
| Sobic.005G015200 | 10354.2767 | -1.2772 | 0.5038 | -2.5350 | 0.0112435 | 0.1165751 | no |
| Sobic.003G370600 | 23.6726 | 3.1640 | 1.2484 | 2.5345 | 0.0112605 | 0.1166891 | no |
| Sobic.003G202200 | 1858.1733 | -1.3119 | 0.5177 | -2.5342 | 0.0112708 | 0.1166891 | no |
| Sobic.003G152800 | 601.5181 | -0.8012 | 0.3162 | -2.5340 | 0.0112755 | 0.1166891 | no |
| Sobic.009G247200 | 3043.8943 | 1.1326 | 0.4470 | 2.5340 | 0.0112766 | 0.1166891 | no |
| Sobic.003G256200 | 4700.5763 | -1.1341 | 0.4478 | -2.5327 | 0.0113185 | 0.1170484 | no |
| Sobic.004G237200 | 164.6767 | -1.1525 | 0.4551 | -2.5325 | 0.0113261 | 0.1170484 | no |
| Sobic.007G171800 | 3209.6891 | -1.4666 | 0.5791 | -2.5324 | 0.011328 | 0.1170484 | no |
| Sobic.010G147833 | 356.0975 | 1.0200 | 0.4029 | 2.5317 | 0.0113505 | 0.117215 | no |
| Sobic.001G464500 | 897.9852 | 0.6345 | 0.2506 | 2.5314 | 0.0113592 | 0.117215 | no |
| Sobic.003G328100 | 18202.4566 | -0.9493 | 0.3750 | -2.5312 | 0.0113662 | 0.117215 | no |
| Sobic.003G101400 | 4591.0391 | 0.9428 | 0.3725 | 2.5312 | 0.0113663 | 0.117215 | no |
| Sobic.001G416200 | 22.2328 | -1.8143 | 0.7169 | -2.5307 | 0.0113851 | 0.1173508 | no |
| Sobic.006G279800 | 275.4384 | -1.1932 | 0.4715 | -2.5305 | 0.011391 | 0.1173545 | no |

| Sobic.001G287500 | 1647.3144 | 0.8720 | 0.3447 | 2.5296 | 0.0114188 | 0.1175837 | no |
| --- | --- | --- | --- | --- | --- | --- | --- |
| Sobic.003G019200 | 777.5198 | 1.1474 | 0.4537 | 2.5287 | 0.0114471 | 0.117817 | no |
| Sobic.008G183400 | 226.1427 | 2.5061 | 0.9912 | 2.5284 | 0.0114589 | 0.1178817 | no |
| Sobic.005G068800 | 51.7932 | 1.4237 | 0.5631 | 2.5282 | 0.0114659 | 0.1178954 | no |
| Sobic.001G509800 | 73.6477 | 1.2467 | 0.4931 | 2.5280 | 0.0114715 | 0.1178958 | no |
| Sobic.009G135400 | 18.3251 | 5.3830 | 2.1296 | 2.5278 | 0.0114795 | 0.1179209 | no |
| Sobic.001G484800 | 1900.8137 | -1.1105 | 0.4394 | -2.5273 | 0.0114958 | 0.1180304 | no |
| Sobic.006G220600 | 1297.8945 | -1.8790 | 0.7435 | -2.5270 | 0.0115026 | 0.118043 | no |
| Sobic.002G191000 | 12.2759 | 3.0000 | 1.1873 | 2.5267 | 0.0115146 | 0.1181085 | no |
| Sobic.004G099300 | 2732.6797 | 1.0092 | 0.3995 | 2.5263 | 0.011527 | 0.1181774 | no |
| Sobic.003G440300 | 2724.4988 | 1.7378 | 0.6879 | 2.5260 | 0.0115354 | 0.1181774 | no |
| Sobic.001G409400 | 83.9399 | 1.6434 | 0.6506 | 2.5260 | 0.0115381 | 0.1181774 | no |
| Sobic.001G310800 | 1302.4435 | -1.2716 | 0.5035 | -2.5252 | 0.0115631 | 0.1183714 | no |
| Sobic.004G107500 | 1271.5829 | -0.8731 | 0.3458 | -2.5246 | 0.0115832 | 0.1183714 | no |
| Sobic.002G360301 | 662.3133 | 1.3383 | 0.5301 | 2.5245 | 0.0115853 | 0.1183714 | no |
| Sobic.003G042400 | 527.2132 | 0.6689 | 0.2650 | 2.5244 | 0.0115911 | 0.1183714 | no |
| Sobic.002G202600 | 324.7605 | 0.8432 | 0.3340 | 2.5241 | 0.0115988 | 0.1183714 | no |
| Sobic.002G051000 | 59.0932 | -1.9894 | 0.7882 | -2.5239 | 0.0116046 | 0.1183714 | no |
| Sobic.001G401100 | 53.7205 | 1.9281 | 0.7640 | 2.5237 | 0.0116118 | 0.1183714 | no |
| Sobic.004G185900 | 517.5652 | 1.4810 | 0.5868 | 2.5237 | 0.0116119 | 0.1183714 | no |
| Sobic.001G035800 | 5032.0124 | -0.6970 | 0.2762 | -2.5237 | 0.0116127 | 0.1183714 | no |
| Sobic.005G214300 | 10.0292 | -4.0266 | 1.5955 | -2.5237 | 0.0116132 | 0.1183714 | no |
| Sobic.001G122400 | 5805.4253 | -1.0942 | 0.4338 | -2.5225 | 0.0116534 | 0.1187236 | no |
| Sobic.006G152600 | 4085.9447 | -1.7105 | 0.6782 | -2.5222 | 0.0116608 | 0.1187419 | no |
| Sobic.006G047500 | 145.7658 | -0.8170 | 0.3241 | -2.5205 | 0.0117177 | 0.1192633 | no |
| Sobic.005G122400 | 19.2986 | 1.8763 | 0.7445 | 2.5202 | 0.0117284 | 0.1193149 | no |
| Sobic.003G380000 | 1882.3360 | -1.0122 | 0.4017 | -2.5198 | 0.0117431 | 0.1193726 | no |
| Sobic.009G252200 | 22.5637 | 1.6740 | 0.6644 | 2.5196 | 0.011748 | 0.1193726 | no |
| Sobic.009G002100 | 45.4419 | -1.3697 | 0.5436 | -2.5195 | 0.011751 | 0.1193726 | no |
| Sobic.004G022900 | 24.5468 | 3.7294 | 1.4807 | 2.5187 | 0.0117781 | 0.1195895 | no |
| Sobic.002G001900 | 6837.9164 | -1.7556 | 0.6972 | -2.5180 | 0.0118029 | 0.1197418 | no |
| Sobic.004G337900 | 3573.9317 | 1.1318 | 0.4495 | 2.5179 | 0.0118044 | 0.1197418 | no |
| Sobic.003G241600 | 182.3115 | 1.0642 | 0.4228 | 2.5167 | 0.011846 | 0.1201055 | no |
| Sobic.004G028500 | 3733.6073 | -1.0383 | 0.4127 | -2.5161 | 0.0118657 | 0.1202481 | no |
| Sobic.001G383900 | 1868.7369 | -1.1236 | 0.4467 | -2.5154 | 0.01189 | 0.1204151 | no |
| Sobic.005G029000 | 94.5537 | 1.0184 | 0.4049 | 2.5151 | 0.0119006 | 0.1204151 | no |
| Sobic.010G277900 | 682.7723 | 0.6015 | 0.2392 | 2.5150 | 0.0119037 | 0.1204151 | no |
| Sobic.003G371000 | 1581.4331 | -4.2020 | 1.6710 | -2.5146 | 0.0119154 | 0.1204151 | no |
| Sobic.004G252200 | 124.3513 | 1.3452 | 0.5349 | 2.5146 | 0.0119158 | 0.1204151 | no |
| Sobic.002G024100 | 449.8859 | 1.6222 | 0.6451 | 2.5145 | 0.0119216 | 0.1204151 | no |
| Sobic.010G067900 | 67.3993 | 1.1257 | 0.4477 | 2.5144 | 0.0119222 | 0.1204151 | no |
| Sobic.007G001300 | 16.5756 | -1.8139 | 0.7216 | -2.5136 | 0.0119499 | 0.1206372 | no |
| Sobic.001G439200 | 22333.9550 | -1.7623 | 0.7013 | -2.5129 | 0.0119727 | 0.1206942 | no |
| Sobic.008G027200 | 71.9828 | 2.1342 | 0.8493 | 2.5129 | 0.0119759 | 0.1206942 | no |
| Sobic.006G240300 | 107.1379 | 1.3913 | 0.5537 | 2.5128 | 0.0119763 | 0.1206942 | no |
| Sobic.005G218500 | 67.2981 | -6.5075 | 2.5898 | -2.5128 | 0.0119784 | 0.1206942 | no |
| Sobic.003G397100 | 189.4462 | 0.8945 | 0.3560 | 2.5126 | 0.0119846 | 0.1206987 | no |
| Sobic.002G283500 | 7.1280 | 6.0473 | 2.4074 | 2.5120 | 0.0120057 | 0.1208536 | no |
| Sobic.002G367700 | 1403.7841 | -1.6184 | 0.6444 | -2.5116 | 0.012018 | 0.1208873 | no |
| Sobic.009G248300 | 606.6354 | 1.1405 | 0.4541 | 2.5115 | 0.0120205 | 0.1208873 | no |
| Sobic.005G060700 | 249.5344 | -1.3760 | 0.5480 | -2.5111 | 0.0120366 | 0.1209909 | no |
| Sobic.001G104000 | 717.2653 | 0.9954 | 0.3965 | 2.5107 | 0.0120489 | 0.1210571 | no |
| Sobic.006G163100 | 160.3394 | -1.2001 | 0.4781 | -2.5102 | 0.0120661 | 0.1211724 | no |
| Sobic.003G439100 | 1642.6777 | -0.8895 | 0.3544 | -2.5099 | 0.0120757 | 0.1212116 | no |
| Sobic.008G044000 | 315.5415 | 1.4211 | 0.5663 | 2.5092 | 0.0120995 | 0.1212471 | no |
| Sobic.004G217600 | 2720.5931 | -0.9516 | 0.3793 | -2.5091 | 0.0121029 | 0.1212471 | no |
| Sobic.003G368901 | 214.6521 | 0.9523 | 0.3795 | 2.5091 | 0.0121045 | 0.1212471 | no |

| Sobic.010G248200 | 2508.0002 | -1.5514 | 0.6183 | -2.5091 | 0.0121047 | 0.1212471 | no |
| --- | --- | --- | --- | --- | --- | --- | --- |
| Sobic.002G091600 | 319.6706 | -0.8021 | 0.3197 | -2.5090 | 0.012108 | 0.1212471 | no |
| Sobic.009G071600 | 13.8740 | 2.9723 | 1.1849 | 2.5086 | 0.0121217 | 0.1213264 | no |
| Sobic.001G384800 | 1529.5679 | -0.9573 | 0.3816 | -2.5083 | 0.0121321 | 0.1213727 | no |
| Sobic.009G105900 | 883.2625 | 1.2478 | 0.4978 | 2.5065 | 0.0121938 | 0.121932 | no |
| Sobic.009G029100 | 165.8501 | -1.0959 | 0.4373 | -2.5061 | 0.0122069 | 0.1220056 | no |
| Sobic.009G000400 | 1396.6295 | 0.7108 | 0.2837 | 2.5058 | 0.0122167 | 0.1220455 | no |
| Sobic.002G000500 | 3740.7948 | -1.5127 | 0.6041 | -2.5040 | 0.0122784 | 0.1226038 | no |
| Sobic.002G001800 | 8093.8500 | -1.0837 | 0.4331 | -2.5023 | 0.0123401 | 0.1231612 | no |
| Sobic.010G139600 | 2416.7164 | 0.5700 | 0.2278 | 2.5018 | 0.0123562 | 0.1232641 | no |
| Sobic.001G320200 | 2468.2616 | 1.2406 | 0.4959 | 2.5014 | 0.0123697 | 0.1233404 | no |
| Sobic.001G093500 | 36879.8980 | -1.6957 | 0.6781 | -2.5005 | 0.0124013 | 0.1235965 | no |
| Sobic.001G076500 | 2035.6417 | -1.3932 | 0.5573 | -2.5001 | 0.0124143 | 0.1236685 | no |
| Sobic.010G236600 | 134.4290 | 0.9507 | 0.3803 | 2.4999 | 0.0124212 | 0.1236787 | no |
| Sobic.002G084700 | 10.9308 | -3.3750 | 1.3504 | -2.4993 | 0.0124433 | 0.1238396 | no |
| Sobic.010G067100 | 7033.7402 | -1.1833 | 0.4738 | -2.4975 | 0.0125063 | 0.1243563 | no |
| Sobic.002G043700 | 164.9935 | 1.0759 | 0.4308 | 2.4975 | 0.012507 | 0.1243563 | no |
| Sobic.003G018800 | 1511.8274 | 1.1451 | 0.4586 | 2.4967 | 0.0125354 | 0.1245805 | no |
| Sobic.001G505100 | 10097.7946 | 1.3319 | 0.5336 | 2.4961 | 0.0125557 | 0.1246619 | no |
| Sobic.002G405600 | 13417.4340 | -0.8778 | 0.3517 | -2.4960 | 0.0125604 | 0.1246619 | no |
| Sobic.004G023200 | 12650.5918 | -1.2791 | 0.5125 | -2.4959 | 0.0125652 | 0.1246619 | no |
| Sobic.001G169200 | 3678.1950 | -0.9793 | 0.3924 | -2.4958 | 0.0125673 | 0.1246619 | no |
| Sobic.001G344700 | 328.9734 | 1.8339 | 0.7349 | 2.4953 | 0.0125851 | 0.1246795 | no |
| Sobic.008G042300 | 61.1632 | 1.6791 | 0.6729 | 2.4953 | 0.0125857 | 0.1246795 | no |
| Sobic.003G112700 | 335.2969 | -1.5204 | 0.6093 | -2.4951 | 0.012591 | 0.1246795 | no |
| Sobic.009G222200 | 2293.5198 | -1.7839 | 0.7150 | -2.4951 | 0.0125927 | 0.1246795 | no |
| Sobic.009G004200 | 199.6690 | 1.0084 | 0.4042 | 2.4947 | 0.0126051 | 0.1247316 | no |
| Sobic.009G011400 | 1139.1545 | 0.7953 | 0.3188 | 2.4946 | 0.0126098 | 0.1247316 | no |
| Sobic.007G151900 | 1382.2758 | -1.2335 | 0.4945 | -2.4944 | 0.0126169 | 0.1247434 | no |
| Sobic.002G412001 | 111.9683 | -2.6507 | 1.0629 | -2.4939 | 0.0126345 | 0.1248586 | no |
| Sobic.009G128901 | 250.8827 | -1.3806 | 0.5537 | -2.4937 | 0.0126418 | 0.1248722 | no |
| Sobic.004G266800 | 183.6329 | -0.9488 | 0.3807 | -2.4926 | 0.012681 | 0.125201 | no |
| Sobic.004G308700 | 19261.7305 | -1.1562 | 0.4639 | -2.4923 | 0.012692 | 0.1252514 | no |
| Sobic.003G441900 | 238.2554 | 0.8316 | 0.3337 | 2.4921 | 0.0126997 | 0.1252681 | no |
| Sobic.001G117900 | 55.3688 | 1.5415 | 0.6186 | 2.4918 | 0.0127094 | 0.1253051 | no |
| Sobic.001G050600 | 546.6931 | 1.0950 | 0.4395 | 2.4915 | 0.0127216 | 0.1253668 | no |
| Sobic.001G354700 | 1551.1750 | -2.1587 | 0.8666 | -2.4910 | 0.0127383 | 0.1254732 | no |
| Sobic.007G036750 | 23.8560 | 1.5748 | 0.6324 | 2.4903 | 0.0127652 | 0.1256792 | no |
| Sobic.004G268500 | ######### | -1.4639 | 0.5879 | -2.4901 | 0.0127713 | 0.1256804 | no |
| Sobic.002G190800 | 442.6692 | -0.8408 | 0.3381 | -2.4871 | 0.0128803 | 0.1266941 | no |
| Sobic.002G065700 | 43.7524 | 2.2913 | 0.9215 | 2.4866 | 0.0128987 | 0.1267865 | no |
| Sobic.001G293700 | 1045.5335 | 1.0455 | 0.4205 | 2.4864 | 0.0129034 | 0.1267865 | no |
| Sobic.010G277600 | 6961.3661 | -1.1838 | 0.4761 | -2.4863 | 0.0129077 | 0.1267865 | no |
| Sobic.008G188100 | 411.4566 | 0.9867 | 0.3970 | 2.4852 | 0.0129462 | 0.1271051 | no |
| Sobic.005G187300 | 2001.4009 | 0.5675 | 0.2284 | 2.4850 | 0.0129538 | 0.1271204 | no |
| Sobic.001G012700 | 282.6602 | -0.8078 | 0.3251 | -2.4849 | 0.01296 | 0.127123 | no |
| Sobic.002G245300 | 188.7056 | 2.0481 | 0.8243 | 2.4846 | 0.0129694 | 0.1271553 | no |
| Sobic.009G209900 | 1161.7558 | -0.6205 | 0.2498 | -2.4837 | 0.0130027 | 0.1273747 | no |
| Sobic.010G009200 | 209.1568 | 1.1512 | 0.4635 | 2.4836 | 0.0130074 | 0.1273747 | no |
| Sobic.003G269400 | 26.8493 | 2.7323 | 1.1002 | 2.4835 | 0.0130099 | 0.1273747 | no |
| Sobic.003G314300 | 239.7042 | 0.9996 | 0.4025 | 2.4833 | 0.0130161 | 0.1273761 | no |
| Sobic.003G138400 | 2521.6052 | 0.7906 | 0.3185 | 2.4824 | 0.0130494 | 0.1276036 | no |
| Sobic.003G424200 | 3167.5259 | -0.7319 | 0.2949 | -2.4823 | 0.0130548 | 0.1276036 | no |
| Sobic.010G109100 | 49.5491 | 2.6255 | 1.0577 | 2.4822 | 0.0130574 | 0.1276036 | no |
| Sobic.001G509300 | 4395.3407 | 1.9060 | 0.7680 | 2.4816 | 0.0130782 | 0.1277309 | no |
| Sobic.003G334400 | 78.2056 | 1.2711 | 0.5122 | 2.4815 | 0.0130842 | 0.1277309 | no |
| Sobic.009G062800 | 4181.4476 | 2.3501 | 0.9471 | 2.4813 | 0.0130886 | 0.1277309 | no |

| Sobic.003G151300 | 332.1682 | 0.7736 | 0.3118 | 2.4811 | 0.0130971 | 0.1277541 | no |
| --- | --- | --- | --- | --- | --- | --- | --- |
| Sobic.002G322800 | 1085.0336 | -1.2961 | 0.5226 | -2.4800 | 0.0131377 | 0.1280914 | no |
| Sobic.001G082300 | 90.6865 | -1.6863 | 0.6803 | -2.4788 | 0.0131829 | 0.1284725 | no |
| Sobic.003G331000 | 132.9889 | 0.9478 | 0.3824 | 2.4784 | 0.0131958 | 0.1285386 | no |
| Sobic.001G183000 | 5123.4618 | -0.6605 | 0.2665 | -2.4781 | 0.0132074 | 0.1285921 | no |
| Sobic.010G063200 | 11942.1138 | -0.7238 | 0.2921 | -2.4780 | 0.0132137 | 0.1285921 | no |
| Sobic.001G407600 | 13263.7806 | -1.2701 | 0.5126 | -2.4778 | 0.0132196 | 0.1285921 | no |
| Sobic.008G131500 | 1441.7173 | -0.7361 | 0.2971 | -2.4772 | 0.0132424 | 0.1287546 | no |
| Sobic.002G356500 | 18.2886 | 1.9715 | 0.7960 | 2.4768 | 0.0132561 | 0.1288187 | no |
| Sobic.002G378700 | 90.1749 | 1.6594 | 0.6700 | 2.4767 | 0.0132612 | 0.1288187 | no |
| Sobic.009G031300 | 18.7103 | 3.0145 | 1.2173 | 2.4763 | 0.0132744 | 0.1288877 | no |
| Sobic.006G118400 | 168.5799 | 1.2996 | 0.5250 | 2.4756 | 0.0133013 | 0.1290894 | no |
| Sobic.002G251900 | 219.0193 | 1.1978 | 0.4840 | 2.4749 | 0.0133274 | 0.1292829 | no |
| Sobic.003G140000 | 420.8673 | -0.8161 | 0.3298 | -2.4747 | 0.0133348 | 0.1292951 | no |
| Sobic.002G212300 | 531.7921 | -1.1266 | 0.4554 | -2.4741 | 0.0133572 | 0.129453 | no |
| Sobic.007G198200 | 91.9028 | -1.6988 | 0.6867 | -2.4737 | 0.0133721 | 0.1295376 | no |
| Sobic.001G297000 | 861.5645 | 1.3050 | 0.5277 | 2.4731 | 0.0133965 | 0.129715 | no |
| Sobic.002G269400 | 55.8781 | -2.6313 | 1.0641 | -2.4727 | 0.0134086 | 0.1297273 | no |
| Sobic.008G006300 | 32.4473 | 1.5010 | 0.6070 | 2.4727 | 0.0134101 | 0.1297273 | no |
| Sobic.008G052200 | 707.6100 | 0.7764 | 0.3140 | 2.4725 | 0.0134163 | 0.1297274 | no |
| Sobic.006G184900 | 1398.5393 | 0.6663 | 0.2695 | 2.4722 | 0.0134272 | 0.1297738 | no |
| Sobic.001G448400 | 2180.1156 | -1.7408 | 0.7042 | -2.4720 | 0.0134354 | 0.1297793 | no |
| Sobic.004G191000 | 74.8711 | 2.6710 | 1.0806 | 2.4717 | 0.0134462 | 0.1297793 | no |
| Sobic.001G393900 | 446.6707 | 0.9178 | 0.3713 | 2.4717 | 0.0134464 | 0.1297793 | no |
| Sobic.003G205200 | 2047.1073 | 1.1468 | 0.4640 | 2.4715 | 0.0134536 | 0.1297793 | no |
| Sobic.010G105100 | 941.1201 | 0.8297 | 0.3357 | 2.4714 | 0.0134585 | 0.1297793 | no |
| Sobic.003G326100 | 7.5523 | 4.3365 | 1.7552 | 2.4706 | 0.0134887 | 0.1300105 | no |
| Sobic.002G386000 | 2086.0840 | -1.1775 | 0.4767 | -2.4703 | 0.0134987 | 0.130048 | no |
| Sobic.003G231800 | 550.2873 | -1.5693 | 0.6353 | -2.4700 | 0.013513 | 0.1301258 | no |
| Sobic.002G361700 | 41.2468 | 1.2223 | 0.4949 | 2.4697 | 0.0135221 | 0.1301543 | no |
| Sobic.007G113900 | 117.1621 | 0.8812 | 0.3569 | 2.4695 | 0.013531 | 0.1301804 | no |
| Sobic.006G057866 | 804.8878 | -1.7733 | 0.7182 | -2.4692 | 0.0135422 | 0.130226 | no |
| Sobic.007G015500 | 1105.3341 | -0.9400 | 0.3807 | -2.4690 | 0.0135499 | 0.130226 | no |
| Sobic.003G041900 | 125.7472 | 0.9146 | 0.3705 | 2.4688 | 0.0135584 | 0.130226 | no |
| Sobic.001G543100 | 13.7432 | 2.8632 | 1.1598 | 2.4687 | 0.0135604 | 0.130226 | no |
| Sobic.006G267500 | 129.3112 | 1.3306 | 0.5391 | 2.4681 | 0.0135826 | 0.1303271 | no |
| Sobic.004G235100 | 377.1153 | -1.3249 | 0.5368 | -2.4681 | 0.0135833 | 0.1303271 | no |
| Sobic.006G208400 | 485.1537 | 0.5756 | 0.2333 | 2.4675 | 0.0136079 | 0.1304247 | no |
| Sobic.001G354501 | 622.5444 | -0.9239 | 0.3744 | -2.4675 | 0.0136079 | 0.1304247 | no |
| Sobic.006G131900 | 542.9495 | 1.9206 | 0.7784 | 2.4673 | 0.0136121 | 0.1304247 | no |
| Sobic.001G457700 | 380.5459 | 0.7882 | 0.3195 | 2.4668 | 0.0136317 | 0.1304611 | no |
| Sobic.010G101900 | 18.1427 | 2.1904 | 0.8880 | 2.4668 | 0.0136334 | 0.1304611 | no |
| Sobic.001G314800 | 210.5810 | 0.9968 | 0.4041 | 2.4668 | 0.0136344 | 0.1304611 | no |
| Sobic.001G273875 | 9.0463 | -2.6208 | 1.0626 | -2.4664 | 0.0136489 | 0.1305408 | no |
| Sobic.007G023300 | 26744.6661 | -1.0495 | 0.4257 | -2.4654 | 0.0136871 | 0.1308467 | no |
| Sobic.010G073600 | 12.2845 | -2.2826 | 0.9260 | -2.4650 | 0.0137021 | 0.1308873 | no |
| Sobic.005G116500 | 338.6867 | -1.0540 | 0.4276 | -2.4649 | 0.0137038 | 0.1308873 | no |
| Sobic.004G231800 | 1705.3218 | -1.2928 | 0.5245 | -2.4646 | 0.013718 | 0.1309633 | no |
| Sobic.002G346250 | 207.0446 | 2.0199 | 0.8199 | 2.4636 | 0.0137567 | 0.1312486 | no |
| Sobic.004G281200 | 193.3863 | 0.9918 | 0.4026 | 2.4635 | 0.0137603 | 0.1312486 | no |
| Sobic.006G140700 | 9505.6606 | -1.0962 | 0.4451 | -2.4630 | 0.0137775 | 0.1313532 | no |
| Sobic.003G289900 | 162.3043 | 0.9342 | 0.3795 | 2.4618 | 0.0138244 | 0.131741 | no |
| Sobic.004G221000 | 308.6852 | -1.0953 | 0.4450 | -2.4615 | 0.0138351 | 0.1317836 | no |
| Sobic.006G230900 | 15.0225 | -2.0230 | 0.8221 | -2.4607 | 0.0138678 | 0.1320352 | no |
| Sobic.004G006000 | 262.6132 | -0.8209 | 0.3337 | -2.4600 | 0.0138931 | 0.1321005 | no |
| Sobic.010G229600 | 501.1339 | -1.2691 | 0.5159 | -2.4600 | 0.0138947 | 0.1321005 | no |
| Sobic.007G092400 | 4715.5273 | 1.0040 | 0.4082 | 2.4599 | 0.0138982 | 0.1321005 | no |

| Sobic.001G236900 | 125.7422 | 1.5287 | 0.6215 | 2.4597 | 0.0139058 | 0.1321005 | no |
| --- | --- | --- | --- | --- | --- | --- | --- |
| Sobic.007G088400 | 50.7731 | 1.4589 | 0.5931 | 2.4597 | 0.013906 | 0.1321005 | no |
| Sobic.001G278800 | 3178.1569 | -0.6791 | 0.2762 | -2.4591 | 0.0139272 | 0.1322423 | no |
| Sobic.003G010900 | 156.5792 | 0.8181 | 0.3327 | 2.4589 | 0.0139367 | 0.132273 | no |
| Sobic.003G379500 | 4715.6134 | -1.6306 | 0.6633 | -2.4585 | 0.0139518 | 0.1323296 | no |
| Sobic.004G182900 | 4552.4205 | -0.8691 | 0.3535 | -2.4584 | 0.0139575 | 0.1323296 | no |
| Sobic.001G284400 | 206.9468 | 1.8477 | 0.7516 | 2.4583 | 0.0139615 | 0.1323296 | no |
| Sobic.008G144500 | 107.6198 | 1.1540 | 0.4696 | 2.4573 | 0.0140003 | 0.1325996 | no |
| Sobic.006G028200 | 504.6892 | 0.9150 | 0.3724 | 2.4572 | 0.0140025 | 0.1325996 | no |
| Sobic.004G184400 | 560.8627 | -0.7031 | 0.2863 | -2.4560 | 0.014048 | 0.1329705 | no |
| Sobic.002G218600 | 33.7668 | -1.6671 | 0.6790 | -2.4552 | 0.0140818 | 0.1332003 | no |
| Sobic.010G082700 | 1458.0358 | -0.6709 | 0.2733 | -2.4551 | 0.0140849 | 0.1332003 | no |
| Sobic.006G187600 | 122.9434 | -0.9163 | 0.3734 | -2.4542 | 0.0141181 | 0.1334548 | no |
| Sobic.002G133900 | 5356.6480 | -1.1011 | 0.4487 | -2.4540 | 0.0141273 | 0.1334815 | no |
| Sobic.003G029600 | 1177.5952 | 0.5939 | 0.2421 | 2.4531 | 0.0141615 | 0.1337338 | no |
| Sobic.002G152800 | 3989.1610 | -0.9806 | 0.3998 | -2.4530 | 0.0141667 | 0.1337338 | no |
| Sobic.007G082000 | 43.8474 | 1.3847 | 0.5646 | 2.4524 | 0.0141898 | 0.1338919 | no |
| Sobic.005G010900 | 557.8982 | 0.7052 | 0.2876 | 2.4521 | 0.0142011 | 0.1339386 | no |
| Sobic.003G160800 | 2397.0254 | -1.2100 | 0.4936 | -2.4515 | 0.0142247 | 0.1341013 | no |
| Sobic.004G010101 | 98.5497 | 0.9635 | 0.3932 | 2.4506 | 0.014262 | 0.1343934 | no |
| Sobic.010G053900 | 60.4877 | 1.0195 | 0.4161 | 2.4500 | 0.0142874 | 0.1345722 | no |
| Sobic.004G336200 | 3373.6104 | -1.0420 | 0.4254 | -2.4496 | 0.014303 | 0.1346513 | no |
| Sobic.002G215200 | 61.6812 | 1.2896 | 0.5265 | 2.4494 | 0.0143086 | 0.1346513 | no |
| Sobic.001G125900 | 10894.3502 | 1.0886 | 0.4445 | 2.4492 | 0.0143185 | 0.1346843 | no |
| Sobic.002G306000 | 1123.1116 | 0.8041 | 0.3285 | 2.4480 | 0.0143634 | 0.1349281 | no |
| Sobic.004G262300 | 1406.9884 | 0.6467 | 0.2642 | 2.4480 | 0.0143641 | 0.1349281 | no |
| Sobic.001G452600 | 352.5175 | 0.9174 | 0.3748 | 2.4480 | 0.0143669 | 0.1349281 | no |
| Sobic.005G208350 | 7.7672 | 5.1677 | 2.1111 | 2.4479 | 0.0143708 | 0.1349281 | no |
| Sobic.004G143400 | 55.4884 | -1.3080 | 0.5344 | -2.4477 | 0.0143789 | 0.1349281 | no |
| Sobic.002G282000 | 30.3123 | 1.7175 | 0.7017 | 2.4476 | 0.0143828 | 0.1349281 | no |
| Sobic.010G013700 | 687.9942 | -1.3246 | 0.5413 | -2.4472 | 0.014396 | 0.1349917 | no |
| Sobic.008G038100 | 56.0607 | 1.3117 | 0.5360 | 2.4470 | 0.0144041 | 0.1350077 | no |
| Sobic.003G362600 | 710.1161 | 0.7106 | 0.2904 | 2.4468 | 0.0144123 | 0.135025 | no |
| Sobic.008G186100 | 43.6323 | -1.2558 | 0.5133 | -2.4466 | 0.0144218 | 0.1350543 | no |
| Sobic.006G093200 | 192.1382 | -1.9690 | 0.8052 | -2.4452 | 0.0144761 | 0.1355022 | no |
| Sobic.001G287600 | 4091.2440 | -0.8503 | 0.3479 | -2.4444 | 0.0145076 | 0.1357369 | no |
| Sobic.005G017200 | 1476.3063 | 1.0160 | 0.4157 | 2.4441 | 0.0145208 | 0.1357996 | no |
| Sobic.005G217900 | 420.7911 | 1.0727 | 0.4389 | 2.4439 | 0.014531 | 0.1358357 | no |
| Sobic.007G116100 | 562.7998 | -1.2379 | 0.5067 | -2.4432 | 0.0145574 | 0.1360213 | no |
| Sobic.006G251000 | 1729.3281 | 0.8213 | 0.3363 | 2.4422 | 0.0145981 | 0.1362701 | no |
| Sobic.008G007200 | 190.5155 | 1.2024 | 0.4924 | 2.4422 | 0.0145987 | 0.1362701 | no |
| Sobic.002G042900 | 20.0659 | 1.9041 | 0.7797 | 2.4421 | 0.0146034 | 0.1362701 | no |
| Sobic.004G227500 | 167.2594 | 0.9217 | 0.3778 | 2.4398 | 0.0146945 | 0.1370596 | no |
| Sobic.002G249900 | 4301.6872 | -1.3448 | 0.5513 | -2.4392 | 0.0147203 | 0.1372399 | no |
| Sobic.010G236800 | 192.2157 | -1.1066 | 0.4538 | -2.4387 | 0.0147419 | 0.1373803 | no |
| Sobic.004G183300 | 641.8046 | -0.7397 | 0.3033 | -2.4384 | 0.0147519 | 0.1374126 | no |
| Sobic.004G165200 | 2647.6584 | 1.1334 | 0.4650 | 2.4375 | 0.0147889 | 0.1376964 | no |
| Sobic.010G000800 | 869.8956 | 0.8406 | 0.3449 | 2.4373 | 0.0147981 | 0.1377212 | no |
| Sobic.001G431000 | 1647.9244 | 0.5210 | 0.2138 | 2.4370 | 0.0148111 | 0.1377822 | no |
| Sobic.009G024133 | 55.9865 | -1.6294 | 0.6687 | -2.4367 | 0.0148229 | 0.1377847 | no |
| Sobic.006G197700 | 1008.0128 | -1.1589 | 0.4756 | -2.4366 | 0.0148245 | 0.1377847 | no |
| Sobic.010G090700 | 47.6613 | -1.2899 | 0.5295 | -2.4362 | 0.0148434 | 0.1378489 | no |
| Sobic.009G254000 | 106.6732 | 1.2731 | 0.5226 | 2.4362 | 0.0148445 | 0.1378489 | no |
| Sobic.005G017000 | 536.2746 | 0.8545 | 0.3509 | 2.4354 | 0.0148767 | 0.1380874 | no |
| Sobic.010G161400 | 21.6930 | -2.1955 | 0.9021 | -2.4337 | 0.0149466 | 0.1386751 | no |
| Sobic.003G373500 | 541.6901 | 0.9873 | 0.4057 | 2.4335 | 0.0149535 | 0.1386782 | no |
| Sobic.002G361500 | 222.5263 | 1.5239 | 0.6264 | 2.4327 | 0.014986 | 0.1389183 | no |

| Sobic.002G361100 | 63.7100 | 2.5599 | 1.0526 | 2.4319 | 0.0150184 | 0.1391532 | no |
| --- | --- | --- | --- | --- | --- | --- | --- |
| Sobic.003G085100 | 1229.1909 | -0.8841 | 0.3636 | -2.4318 | 0.0150248 | 0.1391532 | no |
| Sobic.009G245600 | 29.3908 | 1.5460 | 0.6358 | 2.4316 | 0.0150311 | 0.1391532 | no |
| Sobic.010G100200 | 792.1460 | -0.7141 | 0.2937 | -2.4312 | 0.0150487 | 0.139255 | no |
| Sobic.002G272500 | 7192.4669 | -1.0560 | 0.4344 | -2.4308 | 0.0150666 | 0.1393597 | no |
| Sobic.001G196200 | 1499.6154 | -0.9825 | 0.4043 | -2.4302 | 0.0150895 | 0.1394327 | no |
| Sobic.003G146000 | 149.0076 | 1.6990 | 0.6991 | 2.4302 | 0.0150918 | 0.1394327 | no |
| Sobic.009G122500 | 690.0026 | 0.6166 | 0.2538 | 2.4300 | 0.0150999 | 0.1394327 | no |
| Sobic.002G326800 | 131.4613 | 1.8186 | 0.7484 | 2.4299 | 0.015101 | 0.1394327 | no |
| Sobic.004G054500 | 3578.3560 | -1.1520 | 0.4741 | -2.4297 | 0.0151099 | 0.1394543 | no |
| Sobic.004G166700 | 1173.8757 | 0.7780 | 0.3203 | 2.4295 | 0.0151217 | 0.1395023 | no |
| Sobic.002G140100 | 84.9271 | 1.0411 | 0.4286 | 2.4292 | 0.0151316 | 0.1395323 | no |
| Sobic.004G213500 | 422.0664 | -1.0061 | 0.4142 | -2.4288 | 0.0151485 | 0.1396269 | no |
| Sobic.007G036550 | 188.5139 | 1.1716 | 0.4824 | 2.4286 | 0.0151563 | 0.1396376 | no |
| Sobic.009G121000 | 6756.1157 | -0.8129 | 0.3347 | -2.4284 | 0.0151639 | 0.1396465 | no |
| Sobic.001G268400 | 6.6273 | -2.5234 | 1.0392 | -2.4282 | 0.0151722 | 0.1396622 | no |
| Sobic.008G171100 | 1004.1574 | -1.0354 | 0.4265 | -2.4277 | 0.0151933 | 0.1397518 | no |
| Sobic.001G300201 | 9339.6046 | -0.8677 | 0.3574 | -2.4277 | 0.0151952 | 0.1397518 | no |
| Sobic.008G182300 | 112.9879 | 2.3972 | 0.9876 | 2.4271 | 0.0152187 | 0.1398808 | no |
| Sobic.007G223500 | 21457.7500 | -1.0313 | 0.4249 | -2.4270 | 0.0152254 | 0.1398808 | no |
| Sobic.010G183100 | 1391.1983 | -1.1724 | 0.4831 | -2.4269 | 0.0152291 | 0.1398808 | no |
| Sobic.004G231200 | 166.7062 | 0.8252 | 0.3401 | 2.4264 | 0.015248 | 0.1399935 | no |
| Sobic.001G460100 | 737.3968 | 0.7642 | 0.3150 | 2.4261 | 0.0152609 | 0.1400511 | no |
| Sobic.003G312100 | 437.1085 | 0.8444 | 0.3481 | 2.4253 | 0.0152939 | 0.1402924 | no |
| Sobic.002G017750 | 1029.5211 | -1.2726 | 0.5248 | -2.4251 | 0.0153055 | 0.1403378 | no |
| Sobic.005G053200 | 70.5759 | 1.2294 | 0.5072 | 2.4240 | 0.0153513 | 0.1406973 | no |
| Sobic.002G180400 | 672.1262 | -1.1196 | 0.4620 | -2.4235 | 0.0153719 | 0.1408245 | no |
| Sobic.002G295100 | 3283.2438 | 1.7209 | 0.7103 | 2.4230 | 0.0153943 | 0.1409687 | no |
| Sobic.010G260900 | 15.3851 | -2.0914 | 0.8633 | -2.4226 | 0.0154113 | 0.1410421 | no |
| Sobic.007G089101 | 11.9057 | 4.8451 | 2.0001 | 2.4225 | 0.0154157 | 0.1410421 | no |
| Sobic.004G146300 | 95.1446 | 1.3090 | 0.5404 | 2.4222 | 0.0154269 | 0.1410834 | no |
| Sobic.001G178250 | 497.6418 | -1.1609 | 0.4793 | -2.4220 | 0.0154346 | 0.1410925 | no |
| Sobic.008G172700 | 377.0150 | -1.0167 | 0.4200 | -2.4210 | 0.0154783 | 0.1413781 | no |
| Sobic.009G207900 | 23390.2874 | -0.7732 | 0.3194 | -2.4208 | 0.0154857 | 0.1413781 | no |
| Sobic.010G252700 | 51.9045 | 1.9514 | 0.8061 | 2.4208 | 0.015486 | 0.1413781 | no |
| Sobic.005G213700 | 9.5396 | -3.1277 | 1.2923 | -2.4202 | 0.0155105 | 0.1415413 | no |
| Sobic.007G098500 | 2389.1064 | 0.8519 | 0.3520 | 2.4200 | 0.0155191 | 0.1415455 | no |
| Sobic.009G109000 | 9.5974 | -2.1030 | 0.8690 | -2.4199 | 0.0155244 | 0.1415455 | no |
| Sobic.006G019400 | 9.5562 | 2.7973 | 1.1564 | 2.4191 | 0.0155591 | 0.1417006 | no |
| Sobic.003G128100 | 88.4385 | 0.9349 | 0.3865 | 2.4189 | 0.015567 | 0.1417006 | no |
| Sobic.003G369100 | 65.2469 | 1.9871 | 0.8215 | 2.4189 | 0.0155683 | 0.1417006 | no |
| Sobic.003G261632 | 138.3775 | 1.2249 | 0.5064 | 2.4189 | 0.0155683 | 0.1417006 | no |
| Sobic.001G098500 | 6359.8914 | -1.2421 | 0.5137 | -2.4182 | 0.0155965 | 0.1418957 | no |
| Sobic.006G216100 | 735.2153 | -0.5598 | 0.2315 | -2.4180 | 0.0156078 | 0.141937 | no |
| Sobic.001G337600 | 605.7340 | -1.1584 | 0.4792 | -2.4173 | 0.015635 | 0.1421032 | no |
| Sobic.004G163300 | 445.7345 | 1.0558 | 0.4368 | 2.4172 | 0.0156413 | 0.1421032 | no |
| Sobic.008G126500 | 437.5633 | -0.7325 | 0.3031 | -2.4169 | 0.0156523 | 0.1421032 | no |
| Sobic.002G193900 | 22.7109 | 1.9573 | 0.8099 | 2.4168 | 0.0156579 | 0.1421032 | no |
| Sobic.005G021600 | 4126.8138 | -0.7922 | 0.3278 | -2.4166 | 0.0156647 | 0.1421032 | no |
| Sobic.005G154700 | 130.7791 | 1.2101 | 0.5008 | 2.4166 | 0.0156665 | 0.1421032 | no |
| Sobic.002G354600 | 1650.5089 | -1.1941 | 0.4943 | -2.4156 | 0.0157094 | 0.1424312 | no |
| Sobic.002G301600 | 2255.9725 | -0.8334 | 0.3450 | -2.4153 | 0.0157215 | 0.1424681 | no |
| Sobic.004G304475 | 10.5108 | -3.5548 | 1.4719 | -2.4152 | 0.015727 | 0.1424681 | no |
| Sobic.002G416250 | 197.3107 | -0.8384 | 0.3471 | -2.4150 | 0.0157349 | 0.1424715 | no |
| Sobic.004G016400 | 1612.7757 | -0.9322 | 0.3860 | -2.4149 | 0.0157408 | 0.1424715 | no |
| Sobic.004G290000 | 1409.9718 | 0.8803 | 0.3646 | 2.4142 | 0.0157685 | 0.1426608 | no |
| Sobic.004G128900 | 25825.7373 | -1.2559 | 0.5203 | -2.4140 | 0.0157763 | 0.1426703 | no |

| Sobic.002G320600 | 260.8647 | 0.6999 | 0.2901 | 2.4128 | 0.0158299 | 0.1430934 | no |
| --- | --- | --- | --- | --- | --- | --- | --- |
| Sobic.004G281400 | 438.6144 | -1.9703 | 0.8167 | -2.4124 | 0.0158482 | 0.1431518 | no |
| Sobic.009G131100 | 9707.1442 | -0.5346 | 0.2216 | -2.4124 | 0.0158499 | 0.1431518 | no |
| Sobic.008G017900 | 577.5073 | 1.4731 | 0.6108 | 2.4117 | 0.0158776 | 0.1432984 | no |
| Sobic.009G113400 | 1870.1418 | -0.6484 | 0.2689 | -2.4116 | 0.0158836 | 0.1432984 | no |
| Sobic.006G216400 | 43.1096 | -1.2640 | 0.5242 | -2.4115 | 0.0158866 | 0.1432984 | no |
| Sobic.009G103900 | 7.0398 | -2.6157 | 1.0849 | -2.4111 | 0.0159066 | 0.1434174 | no |
| Sobic.006G157200 | 749.9200 | -1.0655 | 0.4420 | -2.4106 | 0.0159261 | 0.1434787 | no |
| Sobic.007G187300 | 7.2135 | 6.0646 | 2.5158 | 2.4106 | 0.015927 | 0.1434787 | no |
| Sobic.010G033800 | 23.0869 | 2.0084 | 0.8333 | 2.4103 | 0.015941 | 0.143544 | no |
| Sobic.009G050600 | 1227.6448 | -0.6883 | 0.2857 | -2.4097 | 0.0159651 | 0.1437 | no |
| Sobic.007G110300 | 22.7372 | 2.6614 | 1.1046 | 2.4093 | 0.0159813 | 0.1437842 | no |
| Sobic.010G107200 | 19.2184 | 1.7814 | 0.7395 | 2.4089 | 0.015999 | 0.1438355 | no |
| Sobic.002G227800 | 347.4668 | -1.3524 | 0.5614 | -2.4089 | 0.0160007 | 0.1438355 | no |
| Sobic.002G083200 | 3226.1983 | -1.1509 | 0.4781 | -2.4071 | 0.0160777 | 0.1444046 | no |
| Sobic.009G022900 | 10.7433 | -3.5832 | 1.4886 | -2.4070 | 0.0160843 | 0.1444046 | no |
| Sobic.004G123400 | 1714.2167 | 0.5168 | 0.2147 | 2.4068 | 0.0160941 | 0.1444046 | no |
| Sobic.002G399300 | 24.4046 | 1.5694 | 0.6521 | 2.4067 | 0.0160959 | 0.1444046 | no |
| Sobic.002G353600 | 1216.8505 | 0.6778 | 0.2816 | 2.4067 | 0.0160982 | 0.1444046 | no |
| Sobic.006G222700 | 450.1344 | -1.0156 | 0.4221 | -2.4063 | 0.0161129 | 0.1444752 | no |
| Sobic.010G068600 | 98.8930 | 1.2038 | 0.5003 | 2.4059 | 0.0161321 | 0.1445853 | no |
| Sobic.007G015600 | 704.9052 | -0.6891 | 0.2864 | -2.4056 | 0.016147 | 0.1446578 | no |
| Sobic.002G237700 | 78.3779 | 1.2752 | 0.5302 | 2.4050 | 0.0161716 | 0.1448164 | no |
| Sobic.010G004100 | 1543.4863 | 0.6366 | 0.2648 | 2.4043 | 0.0162021 | 0.1449841 | no |
| Sobic.002G082300 | 39.5507 | -1.2207 | 0.5077 | -2.4043 | 0.0162041 | 0.1449841 | no |
| Sobic.007G077200 | 911.4528 | -2.0932 | 0.8708 | -2.4038 | 0.0162275 | 0.1451324 | no |
| Sobic.003G440400 | 1400.1171 | 1.0342 | 0.4304 | 2.4031 | 0.0162586 | 0.1453046 | no |
| Sobic.007G008100 | 14.0899 | 3.4345 | 1.4292 | 2.4030 | 0.0162606 | 0.1453046 | no |
| Sobic.003G194800 | 502.3872 | 0.7185 | 0.2991 | 2.4025 | 0.0162831 | 0.1454445 | no |
| Sobic.004G070200 | 1558.3916 | -0.6085 | 0.2533 | -2.4022 | 0.0162975 | 0.1455118 | no |
| Sobic.004G103200 | 16.0549 | 1.9667 | 0.8188 | 2.4020 | 0.016305 | 0.1455171 | no |
| Sobic.003G227700 | 55.3192 | 1.1938 | 0.4970 | 2.4018 | 0.0163126 | 0.1455233 | no |
| Sobic.007G073700 | 4985.6545 | -0.8618 | 0.3589 | -2.4013 | 0.0163377 | 0.145674 | no |
| Sobic.010G279900 | 1540.2865 | 0.9065 | 0.3775 | 2.4012 | 0.0163433 | 0.145674 | no |
| Sobic.010G030600 | 93.6358 | 0.8462 | 0.3525 | 2.4009 | 0.0163548 | 0.1457146 | no |
| Sobic.004G319600 | 117.7795 | 0.7926 | 0.3302 | 2.4007 | 0.0163638 | 0.1457336 | no |
| Sobic.004G125700 | 227.7305 | -0.8563 | 0.3568 | -2.4003 | 0.0163813 | 0.1458275 | no |
| Sobic.010G008700 | 808.9886 | -0.8559 | 0.3567 | -2.3999 | 0.0163992 | 0.1459251 | no |
| Sobic.003G109300 | 6.6065 | -3.0722 | 1.2805 | -2.3992 | 0.016429 | 0.146129 | no |
| Sobic.006G209700 | 2218.9728 | -0.6443 | 0.2685 | -2.3991 | 0.0164376 | 0.1461434 | no |
| Sobic.001G422600 | 632.7965 | -0.7696 | 0.3208 | -2.3986 | 0.0164592 | 0.1462301 | no |
| Sobic.007G128600 | 69.7364 | 1.0786 | 0.4497 | 2.3985 | 0.0164612 | 0.1462301 | no |
| Sobic.001G103300 | 209.3999 | 0.8166 | 0.3405 | 2.3983 | 0.0164734 | 0.1462769 | no |
| Sobic.004G134700 | 1040.0040 | -0.7185 | 0.2996 | -2.3978 | 0.0164917 | 0.1463781 | no |
| Sobic.003G079700 | 97.8706 | 2.1133 | 0.8814 | 2.3976 | 0.0165029 | 0.1464152 | no |
| Sobic.003G223000 | 79.4942 | -1.0454 | 0.4361 | -2.3972 | 0.0165199 | 0.146505 | no |
| Sobic.007G188701 | 407.7498 | 1.9229 | 0.8022 | 2.3969 | 0.0165348 | 0.1465398 | no |
| Sobic.003G121200 | 43.7009 | 1.7250 | 0.7197 | 2.3967 | 0.0165448 | 0.1465398 | no |
| Sobic.007G139950 | 211.4406 | -1.5219 | 0.6350 | -2.3965 | 0.0165514 | 0.1465398 | no |
| Sobic.002G423400 | 48.4941 | 1.6337 | 0.6817 | 2.3965 | 0.0165517 | 0.1465398 | no |
| Sobic.004G354700 | 1265.2273 | -1.0358 | 0.4323 | -2.3962 | 0.0165647 | 0.1465628 | no |
| Sobic.009G056600 | 13.5306 | -2.5723 | 1.0735 | -2.3962 | 0.0165682 | 0.1465628 | no |
| Sobic.007G103400 | 2528.5688 | -0.9486 | 0.3960 | -2.3953 | 0.016607 | 0.1468445 | no |
| Sobic.004G116100 | 4401.2060 | -1.2831 | 0.5357 | -2.3950 | 0.0166184 | 0.1468838 | no |
| Sobic.004G232400 | 9.8052 | 3.6286 | 1.5153 | 2.3947 | 0.0166336 | 0.1469565 | no |
| Sobic.004G165500 | 2769.4732 | -1.0669 | 0.4456 | -2.3944 | 0.0166468 | 0.1470123 | no |
| Sobic.003G387800 | 13813.8372 | -1.1468 | 0.4792 | -2.3932 | 0.0167004 | 0.1474238 | no |

| Sobic.008G082900 | 293.3528 | -1.4189 | 0.5932 | -2.3921 | 0.0167509 | 0.1478072 | no |
| --- | --- | --- | --- | --- | --- | --- | --- |
| Sobic.003G147000 | 374.1207 | 0.7524 | 0.3146 | 2.3912 | 0.016792 | 0.1481079 | no |
| Sobic.006G152500 | 155.3458 | -1.7878 | 0.7478 | -2.3907 | 0.0168169 | 0.1482656 | no |
| Sobic.007G095300 | 1832.9216 | -0.7648 | 0.3200 | -2.3903 | 0.0168359 | 0.1483708 | no |
| Sobic.005G000300 | 94.0998 | 1.2318 | 0.5154 | 2.3898 | 0.0168587 | 0.148492 | no |
| Sobic.003G319500 | 46.1269 | 1.8099 | 0.7574 | 2.3897 | 0.0168637 | 0.148492 | no |
| Sobic.001G425400 | 10647.5854 | -1.1920 | 0.4989 | -2.3892 | 0.0168874 | 0.1486113 | no |
| Sobic.009G210200 | 958.9317 | -0.9019 | 0.3775 | -2.3889 | 0.0168972 | 0.1486113 | no |
| Sobic.006G239900 | 40.7826 | 2.2898 | 0.9585 | 2.3889 | 0.0168984 | 0.1486113 | no |
| Sobic.001G469900 | 505.3543 | -1.1265 | 0.4718 | -2.3875 | 0.0169644 | 0.1491038 | no |
| Sobic.010G181500 | 110.4778 | -1.3273 | 0.5560 | -2.3871 | 0.0169826 | 0.1491038 | no |
| Sobic.007G003500 | 41.2357 | 2.2321 | 0.9351 | 2.3871 | 0.0169827 | 0.1491038 | no |
| Sobic.009G249100 | 2826.8108 | 0.8806 | 0.3689 | 2.3871 | 0.016983 | 0.1491038 | no |
| Sobic.001G267100 | 9.0637 | -3.5891 | 1.5036 | -2.3869 | 0.0169897 | 0.1491038 | no |
| Sobic.002G038300 | 777.9900 | -1.1894 | 0.4983 | -2.3868 | 0.0169971 | 0.1491042 | no |
| Sobic.002G044700 | 547.2505 | -2.3608 | 0.9892 | -2.3865 | 0.01701 | 0.1491042 | no |
| Sobic.009G036400 | 1213.1265 | 1.7503 | 0.7334 | 2.3865 | 0.017011 | 0.1491042 | no |
| Sobic.003G252400 | 86.8935 | -1.2271 | 0.5144 | -2.3856 | 0.0170495 | 0.1493794 | no |
| Sobic.001G122000 | 862.9730 | 0.7352 | 0.3083 | 2.3846 | 0.0170961 | 0.149726 | no |
| Sobic.003G330500 | 1544.1068 | 0.8688 | 0.3644 | 2.3843 | 0.0171114 | 0.1497977 | no |
| Sobic.010G220600 | 181.5322 | 1.0238 | 0.4295 | 2.3839 | 0.0171309 | 0.149804 | no |
| Sobic.010G076200 | 89.8530 | -1.6650 | 0.6985 | -2.3839 | 0.0171324 | 0.149804 | no |
| Sobic.003G158500 | 76.6233 | 1.9130 | 0.8025 | 2.3838 | 0.0171334 | 0.149804 | no |
| Sobic.002G430200 | 332.3548 | 0.7226 | 0.3031 | 2.3837 | 0.0171416 | 0.1498135 | no |
| Sobic.008G066100 | 5723.5388 | -1.0101 | 0.4240 | -2.3825 | 0.0171947 | 0.1502146 | no |
| Sobic.010G105200 | 5709.2802 | 1.0713 | 0.4498 | 2.3819 | 0.017224 | 0.1504089 | no |
| Sobic.007G206500 | 329.9679 | -1.0433 | 0.4381 | -2.3816 | 0.0172387 | 0.1504747 | no |
| Sobic.006G015600 | 298.7147 | -1.1001 | 0.4620 | -2.3811 | 0.0172627 | 0.1506216 | no |
| Sobic.001G137600 | 21.0222 | -2.5768 | 1.0826 | -2.3802 | 0.0173039 | 0.1509193 | no |
| Sobic.008G040201 | 12.5676 | -3.1486 | 1.3231 | -2.3796 | 0.01733 | 0.1510449 | no |
| Sobic.009G081700 | 148.6310 | 1.2751 | 0.5359 | 2.3794 | 0.0173396 | 0.1510449 | no |
| Sobic.006G262501 | 50.6388 | 1.1696 | 0.4915 | 2.3794 | 0.0173398 | 0.1510449 | no |
| Sobic.001G057500 | 19.9245 | 2.8855 | 1.2129 | 2.3791 | 0.0173559 | 0.1511228 | no |
| Sobic.010G186000 | 1632.6893 | -0.6277 | 0.2639 | -2.3788 | 0.0173705 | 0.1511868 | no |
| Sobic.009G194500 | 4396.5400 | -0.9375 | 0.3941 | -2.3785 | 0.0173831 | 0.1512348 | no |
| Sobic.009G038300 | 689.1671 | 1.1499 | 0.4835 | 2.3782 | 0.0173992 | 0.1513124 | no |
| Sobic.006G280200 | 670.9940 | 0.9825 | 0.4132 | 2.3775 | 0.0174281 | 0.1515008 | no |
| Sobic.002G052600 | 67.2883 | -1.0913 | 0.4591 | -2.3770 | 0.0174523 | 0.1516485 | no |
| Sobic.008G078400 | 1091.6602 | -0.8934 | 0.3759 | -2.3768 | 0.0174613 | 0.1516642 | no |
| Sobic.004G177500 | 6170.2430 | -0.7524 | 0.3166 | -2.3767 | 0.0174685 | 0.1516643 | no |
| Sobic.004G317900 | 1261.7553 | -1.2025 | 0.5060 | -2.3765 | 0.0174764 | 0.1516708 | no |
| Sobic.003G057100 | 69.3958 | -1.2816 | 0.5394 | -2.3761 | 0.017496 | 0.1517625 | no |
| Sobic.001G466000 | 13.7030 | 3.3909 | 1.4271 | 2.3760 | 0.0175014 | 0.1517625 | no |
| Sobic.008G081150 | 4206.8337 | -0.7699 | 0.3241 | -2.3758 | 0.0175111 | 0.151784 | no |
| Sobic.003G219600 | 472.7780 | 0.7905 | 0.3328 | 2.3756 | 0.0175198 | 0.1517973 | no |
| Sobic.010G095500 | 600.4888 | -1.3026 | 0.5484 | -2.3753 | 0.0175339 | 0.1518571 | no |
| Sobic.004G303433 | 165.1893 | -1.0791 | 0.4544 | -2.3747 | 0.017562 | 0.1520134 | no |
| Sobic.001G097200 | 472.3909 | 1.0110 | 0.4257 | 2.3746 | 0.0175663 | 0.1520134 | no |
| Sobic.010G005900 | 8.4528 | -3.1584 | 1.3303 | -2.3742 | 0.0175856 | 0.1521174 | no |
| Sobic.002G265600 | 30.6122 | 2.8408 | 1.1966 | 2.3739 | 0.0176002 | 0.1521817 | no |
| Sobic.003G189600 | 1163.2850 | 0.9579 | 0.4037 | 2.3731 | 0.0176405 | 0.1524675 | no |
| Sobic.008G018600 | 332.5456 | -1.9568 | 0.8247 | -2.3727 | 0.0176585 | 0.152519 | no |
| Sobic.009G127400 | 1118.1071 | -0.8479 | 0.3574 | -2.3726 | 0.0176652 | 0.152519 | no |
| Sobic.005G063000 | 7935.7517 | -1.3172 | 0.5552 | -2.3725 | 0.0176682 | 0.152519 | no |
| Sobic.001G322300 | 636.6453 | 0.6298 | 0.2655 | 2.3722 | 0.017683 | 0.1525602 | no |
| Sobic.004G221500 | 21.2729 | 2.4838 | 1.0471 | 2.3720 | 0.0176903 | 0.1525602 | no |
| Sobic.001G024500 | 6535.6757 | -0.7938 | 0.3347 | -2.3719 | 0.0176946 | 0.1525602 | no |

| Sobic.002G237600 | 41.5339 | 1.7789 | 0.7501 | 2.3714 | 0.0177216 | 0.1527304 | no |
| --- | --- | --- | --- | --- | --- | --- | --- |
| Sobic.010G120600 | 283.1688 | -1.3565 | 0.5725 | -2.3696 | 0.0178093 | 0.1533995 | no |
| Sobic.002G422100 | 645.8849 | 0.8349 | 0.3523 | 2.3695 | 0.0178138 | 0.1533995 | no |
| Sobic.004G070500 | 849.3241 | -0.8714 | 0.3679 | -2.3689 | 0.0178432 | 0.1535898 | no |
| Sobic.003G056500 | 659.0925 | 1.7291 | 0.7301 | 2.3684 | 0.0178649 | 0.1537026 | no |
| Sobic.009G161100 | 77.8875 | -1.3469 | 0.5687 | -2.3683 | 0.0178713 | 0.1537026 | no |
| Sobic.010G256433 | 87.7962 | 0.8892 | 0.3755 | 2.3681 | 0.0178782 | 0.1537026 | no |
| Sobic.008G000100 | 24.4112 | -1.7078 | 0.7216 | -2.3668 | 0.0179435 | 0.1541971 | no |
| Sobic.006G131800 | 30375.9298 | -0.9097 | 0.3844 | -2.3666 | 0.0179507 | 0.1541971 | no |
| Sobic.009G125900 | 478.2224 | 1.0246 | 0.4330 | 2.3665 | 0.0179577 | 0.1541971 | no |
| Sobic.009G076900 | 55.4274 | 1.2946 | 0.5471 | 2.3663 | 0.0179649 | 0.1541971 | no |
| Sobic.003G434900 | 1063.5235 | -0.5768 | 0.2438 | -2.3661 | 0.0179777 | 0.1542426 | no |
| Sobic.009G161401 | 26.2727 | 2.9578 | 1.2502 | 2.3659 | 0.0179849 | 0.1542426 | no |
| Sobic.006G234300 | 125.2914 | -1.0551 | 0.4460 | -2.3657 | 0.0179953 | 0.1542697 | no |
| Sobic.007G022200 | 3481.5925 | -1.2315 | 0.5207 | -2.3652 | 0.0180202 | 0.1543682 | no |
| Sobic.004G272100 | 31478.2721 | -1.0285 | 0.4348 | -2.3652 | 0.0180215 | 0.1543682 | no |
| Sobic.006G117400 | 19.7690 | 2.7813 | 1.1763 | 2.3644 | 0.0180593 | 0.1544775 | no |
| Sobic.003G393800 | 8177.4657 | -1.3942 | 0.5897 | -2.3643 | 0.0180649 | 0.1544775 | no |
| Sobic.010G077250 | 49.5814 | 3.0217 | 1.2781 | 2.3643 | 0.018066 | 0.1544775 | no |
| Sobic.009G083900 | 734.3136 | 0.7138 | 0.3019 | 2.3642 | 0.0180712 | 0.1544775 | no |
| Sobic.005G110469 | 555.2777 | -1.0401 | 0.4400 | -2.3640 | 0.0180777 | 0.1544775 | no |
| Sobic.010G042400 | 1848.8999 | -0.9992 | 0.4227 | -2.3640 | 0.0180794 | 0.1544775 | no |
| Sobic.001G043500 | 49.1740 | -1.2601 | 0.5331 | -2.3637 | 0.0180933 | 0.1544775 | no |
| Sobic.008G137400 | 91.2313 | -0.9193 | 0.3889 | -2.3636 | 0.0180981 | 0.1544775 | no |
| Sobic.003G135800 | 465.7377 | -0.6663 | 0.2819 | -2.3635 | 0.0181046 | 0.1544775 | no |
| Sobic.003G368300 | 172.9971 | 1.2071 | 0.5108 | 2.3631 | 0.0181233 | 0.1544775 | no |
| Sobic.001G453200 | 2162.6644 | -0.5292 | 0.2240 | -2.3630 | 0.0181263 | 0.1544775 | no |
| Sobic.001G090700 | 746.3861 | -1.3343 | 0.5647 | -2.3630 | 0.0181277 | 0.1544775 | no |
| Sobic.001G432300 | 126.6484 | 0.8605 | 0.3642 | 2.3630 | 0.0181294 | 0.1544775 | no |
| Sobic.001G484300 | 3593.2466 | -1.2117 | 0.5129 | -2.3627 | 0.0181416 | 0.1544837 | no |
| Sobic.002G335600 | 4757.1558 | 0.9809 | 0.4152 | 2.3626 | 0.0181448 | 0.1544837 | no |
| Sobic.002G278800 | 15557.7125 | -1.0731 | 0.4543 | -2.3620 | 0.0181791 | 0.1547004 | no |
| Sobic.004G241000 | 11.2518 | 2.2140 | 0.9374 | 2.3618 | 0.0181849 | 0.1547004 | no |
| Sobic.007G000700 | 26.7300 | 1.3376 | 0.5664 | 2.3615 | 0.0182004 | 0.1547692 | no |
| Sobic.004G131900 | 115.9758 | 1.0209 | 0.4324 | 2.3612 | 0.0182151 | 0.1547891 | no |
| Sobic.006G076601 | 436.9391 | -0.7174 | 0.3038 | -2.3612 | 0.0182174 | 0.1547891 | no |
| Sobic.009G163900 | 3526.9354 | 1.3817 | 0.5854 | 2.3603 | 0.0182581 | 0.1550605 | no |
| Sobic.006G167600 | 922.7613 | 0.7974 | 0.3378 | 2.3602 | 0.018264 | 0.1550605 | no |
| Sobic.003G088100 | 83.4189 | 1.1470 | 0.4860 | 2.3599 | 0.0182801 | 0.1551341 | no |
| Sobic.001G206600 | 12.1735 | -1.8275 | 0.7747 | -2.3589 | 0.01833 | 0.1554222 | no |
| Sobic.003G382300 | 1686.6887 | 0.9077 | 0.3848 | 2.3588 | 0.018333 | 0.1554222 | no |
| Sobic.001G040000 | 3117.3535 | -1.2574 | 0.5331 | -2.3586 | 0.0183419 | 0.1554222 | no |
| Sobic.009G118600 | 300.7093 | 1.3690 | 0.5804 | 2.3586 | 0.0183435 | 0.1554222 | no |
| Sobic.001G300000 | 185.7423 | 0.6727 | 0.2852 | 2.3583 | 0.0183588 | 0.1554892 | no |
| Sobic.006G224800 | 437.6066 | 0.6656 | 0.2823 | 2.3578 | 0.0183823 | 0.1555901 | no |
| Sobic.003G108600 | 26159.7849 | -0.8706 | 0.3693 | -2.3578 | 0.0183854 | 0.1555901 | no |
| Sobic.004G338300 | 1116.3966 | -0.6367 | 0.2701 | -2.3576 | 0.0183955 | 0.155613 | no |
| Sobic.007G219100 | 7.2920 | 3.1511 | 1.3370 | 2.3569 | 0.0184274 | 0.1558204 | no |
| Sobic.006G126900 | 2152.7286 | 0.8478 | 0.3599 | 2.3553 | 0.0185081 | 0.1564399 | no |
| Sobic.002G209600 | 1841.6450 | -0.9987 | 0.4241 | -2.3551 | 0.0185183 | 0.1564634 | no |
| Sobic.001G397500 | 1244.0401 | -0.6342 | 0.2694 | -2.3543 | 0.0185562 | 0.1567209 | no |
| Sobic.006G037700 | 470.3548 | 1.0015 | 0.4255 | 2.3538 | 0.0185805 | 0.156863 | no |
| Sobic.001G342600 | 11456.4404 | 0.5087 | 0.2162 | 2.3532 | 0.0186145 | 0.157087 | no |
| Sobic.008G149500 | 114.3951 | -2.1409 | 0.9101 | -2.3522 | 0.0186616 | 0.1574218 | no |
| Sobic.006G279400 | 404.1593 | 0.7918 | 0.3366 | 2.3520 | 0.0186703 | 0.1574304 | no |
| Sobic.002G037700 | 144.8410 | 1.6486 | 0.7010 | 2.3519 | 0.0186775 | 0.1574304 | no |
| Sobic.003G414600 | 81.4855 | 1.5943 | 0.6780 | 2.3514 | 0.0187031 | 0.1575825 | no |

| Sobic.001G128600 | 5395.2755 | -1.2355 | 0.5255 | -2.3509 | 0.0187265 | 0.1577169 | no |
| --- | --- | --- | --- | --- | --- | --- | --- |
| Sobic.003G224700 | 178.1798 | 1.0586 | 0.4506 | 2.3494 | 0.0188046 | 0.1583118 | no |
| Sobic.004G297100 | 892.0863 | -1.5309 | 0.6523 | -2.3468 | 0.0189354 | 0.1593494 | no |
| Sobic.005G168800 | 673.6791 | 1.2343 | 0.5262 | 2.3459 | 0.0189811 | 0.1596698 | no |
| Sobic.004G247800 | 668.4555 | -0.5860 | 0.2499 | -2.3446 | 0.0190464 | 0.1601555 | no |
| Sobic.004G342100 | 260.3788 | -1.0234 | 0.4366 | -2.3439 | 0.0190819 | 0.1603895 | no |
| Sobic.002G228200 | 311.3914 | -0.8499 | 0.3627 | -2.3436 | 0.0190999 | 0.1604349 | no |
| Sobic.001G282600 | 1687.1348 | 1.3931 | 0.5945 | 2.3435 | 0.0191025 | 0.1604349 | no |
| Sobic.001G517600 | 3249.5043 | -0.9326 | 0.3980 | -2.3432 | 0.0191192 | 0.1604731 | no |
| Sobic.005G184300 | 2186.6468 | -0.9935 | 0.4240 | -2.3431 | 0.0191222 | 0.1604731 | no |
| Sobic.006G021600 | 653.9900 | -1.1418 | 0.4874 | -2.3427 | 0.0191473 | 0.1606196 | no |
| Sobic.003G114400 | 307.0435 | 0.7480 | 0.3194 | 2.3421 | 0.0191777 | 0.1608101 | no |
| Sobic.003G293400 | 41.0297 | 1.1551 | 0.4933 | 2.3415 | 0.0192049 | 0.160961 | no |
| Sobic.005G208000 | 96.8056 | -1.2548 | 0.5359 | -2.3414 | 0.0192109 | 0.160961 | no |
| Sobic.009G090800 | 559.1289 | -0.6260 | 0.2675 | -2.3407 | 0.0192502 | 0.161226 | no |
| Sobic.006G177300 | 215.6750 | 1.0172 | 0.4347 | 2.3397 | 0.0192977 | 0.1615383 | no |
| Sobic.004G279100 | 1346.1925 | 1.3166 | 0.5628 | 2.3395 | 0.0193119 | 0.1615383 | no |
| Sobic.007G224600 | 12.6681 | 2.4028 | 1.0271 | 2.3394 | 0.0193128 | 0.1615383 | no |
| Sobic.001G418700 | 8041.5308 | -0.6987 | 0.2987 | -2.3393 | 0.0193181 | 0.1615383 | no |
| Sobic.002G133000 | 208.4602 | 1.2457 | 0.5325 | 2.3392 | 0.0193265 | 0.1615444 | no |
| Sobic.003G108200 | 1899.1792 | -0.8778 | 0.3753 | -2.3389 | 0.0193408 | 0.1615465 | no |
| Sobic.001G099000 | 1004.0364 | -0.9087 | 0.3885 | -2.3389 | 0.0193421 | 0.1615465 | no |
| Sobic.001G500000 | 10.0894 | 3.2258 | 1.3808 | 2.3362 | 0.0194813 | 0.1626081 | no |
| Sobic.004G097000 | 208.9741 | -1.1668 | 0.4995 | -2.3360 | 0.0194891 | 0.1626081 | no |
| Sobic.002G340900 | 1693.8209 | 0.7508 | 0.3214 | 2.3358 | 0.0194999 | 0.1626081 | no |
| Sobic.002G192101 | 1137.8459 | 0.9409 | 0.4028 | 2.3357 | 0.0195054 | 0.1626081 | no |
| Sobic.004G060300 | 454.6234 | 0.6989 | 0.2992 | 2.3357 | 0.0195077 | 0.1626081 | no |
| Sobic.002G334900 | 1504.1380 | 0.6741 | 0.2887 | 2.3348 | 0.0195556 | 0.1629038 | no |
| Sobic.008G026700 | 1004.8531 | -1.2605 | 0.5399 | -2.3347 | 0.0195587 | 0.1629038 | no |
| Sobic.010G018100 | 1108.7694 | -1.4121 | 0.6050 | -2.3343 | 0.0195812 | 0.1630023 | no |
| Sobic.006G004400 | 172.8905 | -1.5415 | 0.6604 | -2.3340 | 0.0195946 | 0.1630023 | no |
| Sobic.003G408000 | 13066.3169 | -0.9779 | 0.4190 | -2.3339 | 0.0196009 | 0.1630023 | no |
| Sobic.001G373700 | 281.1147 | 1.0859 | 0.4653 | 2.3339 | 0.0196014 | 0.1630023 | no |
| Sobic.003G341200 | 187.3549 | -1.2437 | 0.5330 | -2.3335 | 0.0196195 | 0.1630254 | no |
| Sobic.004G146600 | 680.7375 | 0.8254 | 0.3537 | 2.3335 | 0.0196196 | 0.1630254 | no |
| Sobic.010G036200 | 26.3692 | 2.4401 | 1.0460 | 2.3328 | 0.0196602 | 0.1632984 | no |
| Sobic.005G053900 | 44.9155 | 1.5297 | 0.6561 | 2.3313 | 0.019735 | 0.1637585 | no |
| Sobic.005G094332 | 276.9075 | -1.2902 | 0.5534 | -2.3313 | 0.0197358 | 0.1637585 | no |
| Sobic.001G438800 | 836.8751 | 0.6728 | 0.2886 | 2.3313 | 0.0197389 | 0.1637585 | no |
| Sobic.007G035700 | 363.2130 | 1.5906 | 0.6824 | 2.3309 | 0.0197586 | 0.1638574 | no |
| Sobic.001G386100 | 650.6551 | 0.6263 | 0.2687 | 2.3307 | 0.0197681 | 0.1638717 | no |
| Sobic.002G144800 | 143.3521 | 0.8431 | 0.3618 | 2.3304 | 0.0197873 | 0.1639666 | no |
| Sobic.001G445500 | 1107.4141 | 0.8313 | 0.3568 | 2.3298 | 0.0198191 | 0.1641254 | no |
| Sobic.002G359000 | 1718.1282 | 0.8947 | 0.3840 | 2.3297 | 0.0198221 | 0.1641254 | no |
| Sobic.009G051500 | 410.8727 | -0.6430 | 0.2761 | -2.3289 | 0.0198637 | 0.1641808 | no |
| Sobic.001G009400 | 43.8937 | 1.8649 | 0.8007 | 2.3289 | 0.0198644 | 0.1641808 | no |
| Sobic.008G126600 | 32.9320 | 1.7703 | 0.7601 | 2.3289 | 0.0198644 | 0.1641808 | no |
| Sobic.001G302732 | 834.1416 | -0.6313 | 0.2711 | -2.3289 | 0.0198651 | 0.1641808 | no |
| Sobic.001G034500 | 560.0661 | 0.7392 | 0.3174 | 2.3288 | 0.0198677 | 0.1641808 | no |
| Sobic.005G222100 | 819.9116 | 0.8279 | 0.3556 | 2.3284 | 0.0198909 | 0.1643084 | no |
| Sobic.006G265300 | 891.2626 | -0.8086 | 0.3473 | -2.3279 | 0.0199174 | 0.1644262 | no |
| Sobic.001G350300 | 313.4284 | -0.8787 | 0.3775 | -2.3278 | 0.0199208 | 0.1644262 | no |
| Sobic.003G275700 | 890.0318 | 0.8751 | 0.3760 | 2.3273 | 0.019947 | 0.1645783 | no |
| Sobic.004G204500 | 6248.8837 | -1.1377 | 0.4893 | -2.3252 | 0.0200625 | 0.1654663 | no |
| Sobic.008G112600 | 181.0138 | 1.2885 | 0.5542 | 2.3248 | 0.0200836 | 0.1654675 | no |
| Sobic.010G160900 | 41.1107 | -1.2739 | 0.5480 | -2.3247 | 0.0200865 | 0.1654675 | no |
| Sobic.002G125900 | 6166.5137 | -0.9116 | 0.3922 | -2.3245 | 0.0200963 | 0.1654675 | no |

| Sobic.003G257100 | 1044.1625 | 1.2150 | 0.5227 | 2.3244 | 0.0201015 | 0.1654675 | no |
| --- | --- | --- | --- | --- | --- | --- | --- |
| Sobic.003G141100 | 638.7492 | -0.8739 | 0.3759 | -2.3244 | 0.0201018 | 0.1654675 | no |
| Sobic.005G102350 | 9.6027 | -6.9557 | 2.9928 | -2.3242 | 0.0201167 | 0.1655249 | no |
| Sobic.002G343901 | 1114.8152 | -0.7992 | 0.3440 | -2.3235 | 0.0201523 | 0.1657536 | no |
| Sobic.010G256466 | 4.9806 | 3.6047 | 1.5516 | 2.3231 | 0.0201716 | 0.1657717 | no |
| Sobic.004G005400 | 870.0355 | 0.9113 | 0.3923 | 2.3230 | 0.0201796 | 0.1657717 | no |
| Sobic.005G128100 | 4341.2582 | -0.9318 | 0.4011 | -2.3229 | 0.0201843 | 0.1657717 | no |
| Sobic.004G327500 | 1071.5393 | 0.8380 | 0.3608 | 2.3229 | 0.020186 | 0.1657717 | no |
| Sobic.002G413900 | 68.5529 | 1.3993 | 0.6025 | 2.3227 | 0.0201955 | 0.1657854 | no |
| Sobic.006G161500 | 32793.8018 | -1.1237 | 0.4840 | -2.3216 | 0.0202523 | 0.1661867 | no |
| Sobic.006G218600 | 35.4548 | 1.5301 | 0.6591 | 2.3214 | 0.020264 | 0.1661886 | no |
| Sobic.009G128400 | 187.3684 | 0.8290 | 0.3571 | 2.3213 | 0.0202683 | 0.1661886 | no |
| Sobic.002G313100 | 809.4029 | 0.6763 | 0.2914 | 2.3209 | 0.0202927 | 0.1662331 | no |
| Sobic.006G143400 | 978.6488 | 0.7301 | 0.3146 | 2.3208 | 0.0202966 | 0.1662331 | no |
| Sobic.009G028400 | 3711.5785 | -0.9104 | 0.3923 | -2.3205 | 0.0203155 | 0.1662331 | no |
| Sobic.009G104900 | 184.1392 | 0.7503 | 0.3233 | 2.3204 | 0.0203215 | 0.1662331 | no |
| Sobic.006G109400 | 32.1223 | 1.5951 | 0.6875 | 2.3203 | 0.0203241 | 0.1662331 | no |
| Sobic.006G196000 | 252.3951 | -0.9332 | 0.4022 | -2.3202 | 0.0203289 | 0.1662331 | no |
| Sobic.003G104200 | 821.7140 | 1.8807 | 0.8106 | 2.3201 | 0.0203345 | 0.1662331 | no |
| Sobic.001G074800 | 229.0756 | 0.9132 | 0.3936 | 2.3201 | 0.0203367 | 0.1662331 | no |
| Sobic.002G229600 | 32.2794 | 1.4094 | 0.6077 | 2.3193 | 0.0203795 | 0.1665183 | no |
| Sobic.002G107400 | 34.8126 | 2.0253 | 0.8736 | 2.3182 | 0.0204379 | 0.166883 | no |
| Sobic.001G340600 | 1218.9006 | -1.0780 | 0.4650 | -2.3182 | 0.02044 | 0.166883 | no |
| Sobic.001G038500 | 2451.6514 | -1.2580 | 0.5428 | -2.3178 | 0.0204611 | 0.1669378 | no |
| Sobic.001G143000 | 1118.6499 | 0.9262 | 0.3996 | 2.3178 | 0.0204625 | 0.1669378 | no |
| Sobic.003G267300 | 31.5437 | 1.8801 | 0.8114 | 2.3172 | 0.0204932 | 0.1670744 | no |
| Sobic.010G112300 | 751.9366 | 1.1183 | 0.4827 | 2.3171 | 0.0204997 | 0.1670744 | no |
| Sobic.010G060000 | 773.4398 | -1.1012 | 0.4753 | -2.3168 | 0.020512 | 0.1670744 | no |
| Sobic.005G086300 | 556.9299 | -1.4112 | 0.6091 | -2.3167 | 0.020518 | 0.1670744 | no |
| Sobic.008G159100 | 175.6097 | 2.0744 | 0.8954 | 2.3167 | 0.0205189 | 0.1670744 | no |
| Sobic.003G132600 | 3222.1493 | -1.1493 | 0.4962 | -2.3163 | 0.020541 | 0.1671568 | no |
| Sobic.002G090900 | 34.4422 | -2.0578 | 0.8884 | -2.3162 | 0.0205449 | 0.1671568 | no |
| Sobic.002G421800 | 9.3957 | 2.1694 | 0.9368 | 2.3159 | 0.0205656 | 0.1672614 | no |
| Sobic.007G057100 | 42.4147 | -1.9435 | 0.8393 | -2.3156 | 0.0205801 | 0.1673144 | no |
| Sobic.001G463200 | 6123.3837 | -1.0445 | 0.4512 | -2.3152 | 0.0206012 | 0.1674218 | no |
| Sobic.002G155900 | 5396.9526 | 1.7416 | 0.7524 | 2.3148 | 0.020622 | 0.1675259 | no |
| Sobic.003G072400 | 44.8325 | -1.3371 | 0.5777 | -2.3145 | 0.0206406 | 0.1676124 | no |
| Sobic.005G104200 | 5.4996 | 3.6462 | 1.5755 | 2.3143 | 0.0206537 | 0.1676168 | no |
| Sobic.003G003600 | 5563.2990 | -0.9536 | 0.4121 | -2.3142 | 0.0206579 | 0.1676168 | no |
| Sobic.007G173000 | 937.4287 | 0.6478 | 0.2799 | 2.3141 | 0.020665 | 0.1676168 | no |
| Sobic.005G013600 | 88.6019 | 0.9038 | 0.3908 | 2.3127 | 0.0207364 | 0.1681316 | no |
| Sobic.002G220300 | 907.2508 | -0.9993 | 0.4322 | -2.3123 | 0.0207611 | 0.168184 | no |
| Sobic.004G238600 | 1777.8172 | -1.1324 | 0.4897 | -2.3123 | 0.0207624 | 0.168184 | no |
| Sobic.001G527800 | 831.8319 | 0.9489 | 0.4104 | 2.3122 | 0.0207668 | 0.168184 | no |
| Sobic.003G158400 | 151.1790 | -0.7044 | 0.3047 | -2.3117 | 0.0207944 | 0.1683425 | no |
| Sobic.002G376800 | 608.1668 | -2.2077 | 0.9553 | -2.3110 | 0.0208333 | 0.1685928 | no |
| Sobic.009G165200 | 2469.3230 | -1.1788 | 0.5102 | -2.3105 | 0.0208596 | 0.1686946 | no |
| Sobic.001G105200 | 969.6636 | -1.1259 | 0.4873 | -2.3105 | 0.0208625 | 0.1686946 | no |
| Sobic.003G118100 | 22.9891 | 1.4880 | 0.6441 | 2.3103 | 0.0208718 | 0.1686946 | no |
| Sobic.001G477100 | 130.9688 | -0.9031 | 0.3909 | -2.3102 | 0.0208778 | 0.1686946 | no |
| Sobic.004G269200 | 3588.3981 | 0.6638 | 0.2874 | 2.3098 | 0.0209001 | 0.1687726 | no |
| Sobic.004G065300 | 4182.8676 | -0.8185 | 0.3544 | -2.3097 | 0.0209041 | 0.1687726 | no |
| Sobic.001G106700 | 2714.5790 | -0.6729 | 0.2914 | -2.3096 | 0.0209122 | 0.1687726 | no |
| Sobic.003G369700 | 255.7895 | 1.0003 | 0.4331 | 2.3094 | 0.0209195 | 0.1687726 | no |
| Sobic.001G107600 | 244.8731 | 0.7300 | 0.3161 | 2.3092 | 0.0209336 | 0.1688219 | no |
| Sobic.008G172600 | 11.6365 | 2.3496 | 1.0178 | 2.3085 | 0.0209713 | 0.1690614 | no |
| Sobic.001G005500 | 17605.3881 | -1.3998 | 0.6065 | -2.3079 | 0.0210041 | 0.169201 | no |

| Sobic.009G139400 | 176.8767 | -0.7076 | 0.3066 | -2.3079 | 0.0210062 | 0.169201 | no |
| --- | --- | --- | --- | --- | --- | --- | --- |
| Sobic.006G213100 | 880.4921 | -1.3710 | 0.5941 | -2.3078 | 0.0210127 | 0.169201 | no |
| Sobic.006G093000 | 958.7500 | -1.3155 | 0.5703 | -2.3065 | 0.0210821 | 0.1696951 | no |
| Sobic.003G371100 | 56.2179 | -5.7989 | 2.5146 | -2.3061 | 0.0211053 | 0.1697174 | no |
| Sobic.003G254700 | 298.1568 | 1.2964 | 0.5622 | 2.3061 | 0.0211063 | 0.1697174 | no |
| Sobic.004G017200 | 2317.8806 | -1.0316 | 0.4473 | -2.3060 | 0.021109 | 0.1697174 | no |
| Sobic.006G160400 | 164.3656 | -1.0115 | 0.4388 | -2.3051 | 0.0211584 | 0.1700306 | no |
| Sobic.007G169100 | 6419.7445 | -0.9908 | 0.4299 | -2.3050 | 0.0211641 | 0.1700306 | no |
| Sobic.001G357700 | 5.7113 | -3.1830 | 1.3811 | -2.3048 | 0.0211791 | 0.1700602 | no |
| Sobic.003G380200 | 5036.0105 | -0.7975 | 0.3460 | -2.3047 | 0.0211839 | 0.1700602 | no |
| Sobic.001G340200 | 428.3099 | -0.7837 | 0.3402 | -2.3040 | 0.0212238 | 0.1703153 | no |
| Sobic.004G325600 | 819.7280 | 0.6574 | 0.2854 | 2.3034 | 0.0212544 | 0.170496 | no |
| Sobic.001G248500 | 19.2911 | 2.3484 | 1.0199 | 2.3027 | 0.021296 | 0.1707472 | no |
| Sobic.004G142500 | 471.2784 | 1.7471 | 0.7587 | 2.3026 | 0.0213019 | 0.1707472 | no |
| Sobic.010G008600 | 292.5118 | 1.5762 | 0.6846 | 2.3023 | 0.0213157 | 0.1707928 | no |
| Sobic.004G259900 | 407.7246 | 1.0205 | 0.4433 | 2.3021 | 0.0213295 | 0.1708383 | no |
| Sobic.002G140600 | 1826.5547 | 0.7428 | 0.3227 | 2.3017 | 0.0213527 | 0.1709593 | no |
| Sobic.008G114300 | 3144.6020 | 0.9972 | 0.4333 | 2.3014 | 0.0213674 | 0.1709956 | no |
| Sobic.006G101800 | 1257.6159 | 1.2565 | 0.5460 | 2.3013 | 0.0213734 | 0.1709956 | no |
| Sobic.008G157200 | 2153.7956 | 0.5530 | 0.2403 | 2.3011 | 0.0213832 | 0.1710087 | no |
| Sobic.002G158800 | 928.2118 | -1.0591 | 0.4603 | -2.3010 | 0.0213944 | 0.1710092 | no |
| Sobic.001G393700 | 563.2715 | 0.6509 | 0.2829 | 2.3009 | 0.0213995 | 0.1710092 | no |
| Sobic.001G072700 | 3734.7449 | -0.5644 | 0.2453 | -2.3006 | 0.021412 | 0.171045 | no |
| Sobic.003G336200 | 982.8540 | -0.5652 | 0.2457 | -2.3003 | 0.0214316 | 0.1710885 | no |
| Sobic.007G000900 | 1983.0251 | -1.1073 | 0.4814 | -2.3003 | 0.0214337 | 0.1710885 | no |
| Sobic.008G076500 | 8.2799 | 4.4945 | 1.9541 | 2.3000 | 0.0214477 | 0.1711352 | no |
| Sobic.002G127900 | 233.2063 | 0.7091 | 0.3084 | 2.2996 | 0.0214716 | 0.1712613 | no |
| Sobic.003G106500 | 25995.9998 | -1.1039 | 0.4801 | -2.2994 | 0.0214841 | 0.1712961 | no |
| Sobic.001G302400 | 174.7081 | -0.8651 | 0.3763 | -2.2991 | 0.0214988 | 0.1713487 | no |
| Sobic.008G073600 | 1052.4336 | -0.8411 | 0.3659 | -2.2989 | 0.0215118 | 0.1713875 | no |
| Sobic.001G479200 | 523.6689 | -0.8918 | 0.3880 | -2.2984 | 0.0215414 | 0.1714765 | no |
| Sobic.009G135300 | 274.7338 | 1.0012 | 0.4357 | 2.2982 | 0.0215527 | 0.1714765 | no |
| Sobic.010G227400 | 6308.6964 | 1.1112 | 0.4835 | 2.2980 | 0.0215596 | 0.1714765 | no |
| Sobic.001G264300 | 9.8589 | 2.1974 | 0.9562 | 2.2980 | 0.0215599 | 0.1714765 | no |
| Sobic.010G075000 | 88.3560 | 1.3465 | 0.5859 | 2.2980 | 0.0215636 | 0.1714765 | no |
| Sobic.007G188500 | 66.0525 | 1.8987 | 0.8268 | 2.2965 | 0.0216489 | 0.1720896 | no |
| Sobic.001G137100 | 4859.7705 | -0.6347 | 0.2765 | -2.2954 | 0.0217103 | 0.1724332 | no |
| Sobic.003G253400 | 1801.0074 | 0.7336 | 0.3196 | 2.2952 | 0.0217216 | 0.1724332 | no |
| Sobic.008G088842 | 242.2917 | 0.8079 | 0.3520 | 2.2952 | 0.0217217 | 0.1724332 | no |
| Sobic.010G089200 | 1693.6149 | -1.0931 | 0.4763 | -2.2951 | 0.0217248 | 0.1724332 | no |
| Sobic.001G219900 | 783.3416 | 0.5564 | 0.2425 | 2.2948 | 0.0217456 | 0.1725331 | no |
| Sobic.009G004400 | 355.9564 | -0.7474 | 0.3258 | -2.2943 | 0.0217731 | 0.1726417 | no |
| Sobic.004G153501 | 551.0786 | -0.8367 | 0.3647 | -2.2943 | 0.0217757 | 0.1726417 | no |
| Sobic.007G030900 | 268.1611 | 0.9254 | 0.4034 | 2.2938 | 0.0217994 | 0.1727304 | no |
| Sobic.005G024700 | 64.4651 | 2.5740 | 1.1222 | 2.2938 | 0.0218032 | 0.1727304 | no |
| Sobic.003G132200 | 57.3403 | 1.4584 | 0.6359 | 2.2934 | 0.0218222 | 0.1728155 | no |
| Sobic.008G064200 | 5.0333 | 4.5189 | 1.9706 | 2.2931 | 0.0218432 | 0.1728267 | no |
| Sobic.006G153700 | 330.4594 | -0.9948 | 0.4339 | -2.2929 | 0.0218521 | 0.1728267 | no |
| Sobic.003G100200 | 44.4963 | -1.7330 | 0.7558 | -2.2929 | 0.0218526 | 0.1728267 | no |
| Sobic.007G006800 | 33.2153 | 1.9590 | 0.8544 | 2.2929 | 0.0218564 | 0.1728267 | no |
| Sobic.009G004700 | 447.0389 | 0.6144 | 0.2680 | 2.2926 | 0.0218688 | 0.17286 | no |
| Sobic.004G192800 | 780.0758 | 1.2912 | 0.5635 | 2.2915 | 0.0219358 | 0.1733247 | no |
| Sobic.010G208000 | 10794.8466 | -1.2174 | 0.5313 | -2.2913 | 0.0219475 | 0.1733523 | no |
| Sobic.003G375700 | 847.5452 | -1.0953 | 0.4781 | -2.2910 | 0.0219658 | 0.173432 | no |
| Sobic.010G012400 | 7.0566 | 3.4919 | 1.5243 | 2.2908 | 0.0219767 | 0.1734531 | no |
| Sobic.004G269500 | 9.6405 | 2.3390 | 1.0213 | 2.2902 | 0.0220108 | 0.1736416 | no |
| Sobic.010G083300 | 16.5925 | 1.6768 | 0.7322 | 2.2901 | 0.022017 | 0.1736416 | no |

| Sobic.003G252500 | 755.3562 | 1.3601 | 0.5940 | 2.2897 | 0.0220387 | 0.1737472 | no |
| --- | --- | --- | --- | --- | --- | --- | --- |
| Sobic.004G030400 | 54.5660 | 0.9834 | 0.4296 | 2.2892 | 0.022065 | 0.1738896 | no |
| Sobic.001G434100 | 115.1795 | -1.0379 | 0.4535 | -2.2885 | 0.022106 | 0.174146 | no |
| Sobic.004G203900 | 1736.8280 | 0.5658 | 0.2472 | 2.2884 | 0.022114 | 0.174146 | no |
| Sobic.004G328900 | 20.3800 | -2.3532 | 1.0284 | -2.2882 | 0.0221237 | 0.1741568 | no |
| Sobic.007G149550 | 137.2909 | 0.9295 | 0.4063 | 2.2878 | 0.0221503 | 0.1743012 | no |
| Sobic.010G221600 | 1031.4160 | -0.6476 | 0.2831 | -2.2876 | 0.0221623 | 0.1743311 | no |
| Sobic.001G060100 | 301.7916 | 1.1011 | 0.4815 | 2.2871 | 0.0221914 | 0.1744384 | no |
| Sobic.002G355500 | 4091.5197 | 0.6502 | 0.2843 | 2.2871 | 0.0221925 | 0.1744384 | no |
| Sobic.003G043800 | 540.1579 | 1.0143 | 0.4436 | 2.2867 | 0.0222105 | 0.1745147 | no |
| Sobic.009G088575 | 26.1500 | 1.4503 | 0.6344 | 2.2861 | 0.0222458 | 0.1747266 | no |
| Sobic.001G229200 | 93.0305 | -1.0010 | 0.4380 | -2.2856 | 0.0222772 | 0.1749081 | no |
| Sobic.001G368500 | 7238.9272 | -1.0815 | 0.4734 | -2.2847 | 0.0223295 | 0.1752538 | no |
| Sobic.004G339000 | 122.5565 | 1.0616 | 0.4648 | 2.2842 | 0.0223577 | 0.1753445 | no |
| Sobic.009G000300 | 485.4978 | 0.7556 | 0.3308 | 2.2842 | 0.0223623 | 0.1753445 | no |
| Sobic.004G047100 | 30.3988 | 2.3578 | 1.0322 | 2.2841 | 0.022366 | 0.1753445 | no |
| Sobic.001G115900 | 5.0966 | 3.7219 | 1.6296 | 2.2839 | 0.0223746 | 0.1753464 | no |
| Sobic.007G149700 | 533.5583 | -1.6752 | 0.7341 | -2.2821 | 0.0224833 | 0.1761237 | no |
| Sobic.004G246800 | 104.0037 | -0.8764 | 0.3841 | -2.2818 | 0.022502 | 0.1761237 | no |
| Sobic.002G395801 | 1180.9965 | -0.5521 | 0.2419 | -2.2818 | 0.0225031 | 0.1761237 | no |
| Sobic.010G245100 | 4299.0916 | -1.4645 | 0.6418 | -2.2817 | 0.0225071 | 0.1761237 | no |
| Sobic.002G072500 | 4485.1861 | -0.9862 | 0.4323 | -2.2815 | 0.02252 | 0.1761586 | no |
| Sobic.006G079800 | 268.6341 | -1.2672 | 0.5555 | -2.2811 | 0.0225404 | 0.1762533 | no |
| Sobic.002G065800 | 13.9820 | 3.1023 | 1.3606 | 2.2800 | 0.0226051 | 0.1766937 | no |
| Sobic.003G186500 | 11815.8252 | 0.9195 | 0.4033 | 2.2798 | 0.0226177 | 0.1767263 | no |
| Sobic.002G376700 | 2145.8986 | -0.9800 | 0.4299 | -2.2795 | 0.0226344 | 0.1767325 | no |
| Sobic.009G143000 | 478.1565 | 1.0571 | 0.4637 | 2.2795 | 0.0226352 | 0.1767325 | no |
| Sobic.010G082500 | 527.1099 | 0.8604 | 0.3776 | 2.2788 | 0.0226811 | 0.1770075 | no |
| Sobic.007G171000 | 1539.5833 | 0.7989 | 0.3506 | 2.2786 | 0.022688 | 0.1770075 | no |
| Sobic.007G126900 | 1861.2291 | -0.9659 | 0.4239 | -2.2784 | 0.0227023 | 0.1770075 | no |
| Sobic.001G217400 | 2574.6384 | 1.3555 | 0.5950 | 2.2783 | 0.0227109 | 0.1770075 | no |
| Sobic.006G265200 | 800.3245 | 0.8286 | 0.3637 | 2.2782 | 0.0227145 | 0.1770075 | no |
| Sobic.008G082400 | 545.4213 | 1.2029 | 0.5280 | 2.2781 | 0.0227208 | 0.1770075 | no |
| Sobic.006G117100 | 7.9901 | -3.7410 | 1.6427 | -2.2774 | 0.0227633 | 0.1772699 | no |
| Sobic.004G091200 | 1902.7609 | 0.6755 | 0.2966 | 2.2772 | 0.0227713 | 0.1772699 | no |
| Sobic.001G156800 | 19.7280 | -2.2685 | 0.9964 | -2.2768 | 0.0227963 | 0.1773987 | no |
| Sobic.001G361900 | 9305.4447 | -0.7764 | 0.3411 | -2.2764 | 0.0228239 | 0.1775483 | no |
| Sobic.001G346000 | 22.0082 | -2.3498 | 1.0324 | -2.2760 | 0.0228462 | 0.1776018 | no |
| Sobic.001G357900 | 186.4396 | 1.0364 | 0.4554 | 2.2760 | 0.0228476 | 0.1776018 | no |
| Sobic.006G029100 | 365.3364 | 0.7833 | 0.3442 | 2.2756 | 0.0228714 | 0.177721 | no |
| Sobic.009G204900 | 115.8720 | -2.6361 | 1.1588 | -2.2749 | 0.0229107 | 0.177958 | no |
| Sobic.010G201700 | 6661.2856 | -1.0755 | 0.4728 | -2.2748 | 0.0229188 | 0.177958 | no |
| Sobic.004G357100 | 988.5977 | 0.7224 | 0.3176 | 2.2746 | 0.0229296 | 0.1779769 | no |
| Sobic.010G257000 | 61.2293 | 0.9343 | 0.4109 | 2.2741 | 0.022962 | 0.1781626 | no |
| Sobic.008G136700 | 68.6892 | 0.9831 | 0.4324 | 2.2737 | 0.0229842 | 0.1782691 | no |
| Sobic.001G106400 | 188.7764 | 0.6952 | 0.3058 | 2.2735 | 0.0229939 | 0.1782792 | no |
| Sobic.001G040100 | 990.4164 | 0.6181 | 0.2719 | 2.2729 | 0.0230298 | 0.1784281 | no |
| Sobic.010G231300 | 5701.0244 | -0.8486 | 0.3734 | -2.2727 | 0.0230435 | 0.1784281 | no |
| Sobic.004G244500 | 667.1047 | 1.0442 | 0.4595 | 2.2727 | 0.0230454 | 0.1784281 | no |
| Sobic.010G009532 | 315.2113 | -1.0781 | 0.4744 | -2.2725 | 0.0230554 | 0.1784281 | no |
| Sobic.004G280401 | 73.9439 | -1.9286 | 0.8487 | -2.2725 | 0.0230555 | 0.1784281 | no |
| Sobic.001G281300 | 924.0764 | 0.7332 | 0.3227 | 2.2724 | 0.0230648 | 0.178435 | no |
| Sobic.003G393001 | 145.9225 | -0.8190 | 0.3605 | -2.2720 | 0.0230892 | 0.1785581 | no |
| Sobic.010G198500 | 11.0705 | 3.2332 | 1.4232 | 2.2717 | 0.0231055 | 0.1786188 | no |
| Sobic.004G177400 | 23.6832 | 1.7493 | 0.7701 | 2.2715 | 0.023119 | 0.1786518 | no |
| Sobic.001G021900 | 11.0950 | 2.1394 | 0.9420 | 2.2711 | 0.0231391 | 0.1786518 | no |
| Sobic.002G350700 | 8.9659 | 2.9447 | 1.2966 | 2.2710 | 0.0231452 | 0.1786518 | no |

| Sobic.003G222500 | 4204.7468 | -0.9940 | 0.4377 | -2.2710 | 0.0231483 | 0.1786518 | no |
| --- | --- | --- | --- | --- | --- | --- | --- |
| Sobic.005G165500 | 22538.6864 | -1.0498 | 0.4623 | -2.2709 | 0.0231521 | 0.1786518 | no |
| Sobic.006G231266 | 1191.4723 | -1.0940 | 0.4818 | -2.2706 | 0.0231705 | 0.1787278 | no |
| Sobic.008G141800 | 464.0956 | 0.8778 | 0.3867 | 2.2698 | 0.0232226 | 0.1790649 | no |
| Sobic.005G197300 | 725.1596 | -1.2890 | 0.5683 | -2.2680 | 0.023327 | 0.1797803 | no |
| Sobic.001G249300 | 17.2467 | 1.7619 | 0.7769 | 2.2679 | 0.0233325 | 0.1797803 | no |
| Sobic.003G265400 | 2648.3726 | -1.0120 | 0.4463 | -2.2674 | 0.0233637 | 0.1799551 | no |
| Sobic.003G214600 | 2209.7246 | 0.5083 | 0.2242 | 2.2669 | 0.0233948 | 0.1799703 | no |
| Sobic.009G038800 | 13.1855 | 1.9709 | 0.8694 | 2.2669 | 0.0233959 | 0.1799703 | no |
| Sobic.009G172500 | 52.2766 | 2.2298 | 0.9837 | 2.2668 | 0.0234052 | 0.1799703 | no |
| Sobic.003G095900 | 1912.5851 | 0.8786 | 0.3877 | 2.2666 | 0.0234177 | 0.1799703 | no |
| Sobic.003G135700 | 1415.8008 | -1.1566 | 0.5103 | -2.2665 | 0.023422 | 0.1799703 | no |
| Sobic.001G083000 | 40.6413 | 1.1236 | 0.4958 | 2.2661 | 0.0234433 | 0.1799703 | no |
| Sobic.007G047600 | 275.7121 | 0.9526 | 0.4204 | 2.2661 | 0.0234454 | 0.1799703 | no |
| Sobic.009G148500 | 1950.4364 | -0.8168 | 0.3604 | -2.2661 | 0.023447 | 0.1799703 | no |
| Sobic.006G145700 | 224.9927 | -1.3759 | 0.6072 | -2.2660 | 0.0234494 | 0.1799703 | no |
| Sobic.009G136000 | 25.0475 | 2.0595 | 0.9088 | 2.2660 | 0.023451 | 0.1799703 | no |
| Sobic.009G050400 | 300.8855 | 0.7915 | 0.3494 | 2.2655 | 0.0234839 | 0.1801569 | no |
| Sobic.002G131800 | 73.1489 | 1.4349 | 0.6337 | 2.2643 | 0.0235574 | 0.1806135 | no |
| Sobic.003G341300 | 21893.2069 | -1.2029 | 0.5313 | -2.2642 | 0.0235623 | 0.1806135 | no |
| Sobic.007G202000 | 631.9948 | 1.0990 | 0.4854 | 2.2641 | 0.0235695 | 0.1806135 | no |
| Sobic.002G404400 | 2596.7893 | -0.8491 | 0.3751 | -2.2639 | 0.0235776 | 0.1806135 | no |
| Sobic.010G026100 | 353.0650 | 1.1075 | 0.4893 | 2.2636 | 0.0235987 | 0.1806797 | no |
| Sobic.009G183101 | 252.4005 | 1.6811 | 0.7427 | 2.2635 | 0.0236034 | 0.1806797 | no |
| Sobic.008G123500 | 2393.3025 | 1.0345 | 0.4571 | 2.2632 | 0.0236255 | 0.1807833 | no |
| Sobic.005G002900 | 3135.5089 | -0.9160 | 0.4049 | -2.2626 | 0.0236623 | 0.180999 | no |
| Sobic.004G188800 | 295.1260 | 1.4187 | 0.6271 | 2.2622 | 0.0236856 | 0.1810873 | no |
| Sobic.006G097400 | 202.5820 | -0.7035 | 0.3110 | -2.2620 | 0.023695 | 0.1810873 | no |
| Sobic.009G139800 | 2196.5866 | 0.6238 | 0.2758 | 2.2618 | 0.0237097 | 0.1810873 | no |
| Sobic.003G314000 | 481.5162 | -1.0573 | 0.4675 | -2.2616 | 0.0237249 | 0.1810873 | no |
| Sobic.006G127100 | 82.5718 | 2.6709 | 1.1810 | 2.2615 | 0.0237305 | 0.1810873 | no |
| Sobic.002G136200 | 568.2137 | 0.9491 | 0.4197 | 2.2614 | 0.0237367 | 0.1810873 | no |
| Sobic.001G456800 | 250.2558 | 0.6574 | 0.2907 | 2.2613 | 0.0237429 | 0.1810873 | no |
| Sobic.004G282000 | 143.1556 | 0.9160 | 0.4051 | 2.2612 | 0.0237498 | 0.1810873 | no |
| Sobic.001G356200 | 627.8215 | 0.6594 | 0.2916 | 2.2611 | 0.0237511 | 0.1810873 | no |
| Sobic.005G013901 | 859.0370 | -1.0588 | 0.4683 | -2.2609 | 0.0237631 | 0.1811131 | no |
| Sobic.002G013600 | 374.7570 | -1.1949 | 0.5285 | -2.2607 | 0.0237804 | 0.1811793 | no |
| Sobic.001G428900 | 41.1076 | 1.9549 | 0.8648 | 2.2604 | 0.0237934 | 0.1812129 | no |
| Sobic.003G263300 | 32.2220 | 1.5328 | 0.6783 | 2.2600 | 0.0238243 | 0.1813361 | no |
| Sobic.003G148200 | 159.4303 | -1.5056 | 0.6663 | -2.2598 | 0.0238332 | 0.1813361 | no |
| Sobic.001G508800 | 2891.7676 | 1.2901 | 0.5709 | 2.2598 | 0.0238353 | 0.1813361 | no |
| Sobic.010G093100 | 346.1630 | 0.8783 | 0.3887 | 2.2595 | 0.0238523 | 0.1813994 | no |
| Sobic.001G510400 | 1254.7810 | -1.0096 | 0.4469 | -2.2591 | 0.0238801 | 0.1815456 | no |
| Sobic.001G259100 | 38.3230 | 2.6636 | 1.1796 | 2.2580 | 0.0239446 | 0.1819703 | no |
| Sobic.006G171900 | 139.7533 | 0.9988 | 0.4424 | 2.2578 | 0.023961 | 0.182029 | no |
| Sobic.003G178700 | 3289.3145 | -1.4658 | 0.6493 | -2.2574 | 0.023981 | 0.182033 | no |
| Sobic.001G069200 | 150.3424 | 1.3640 | 0.6043 | 2.2572 | 0.0239931 | 0.182033 | no |
| Sobic.001G469500 | 2895.8257 | 0.7092 | 0.3142 | 2.2571 | 0.0239994 | 0.182033 | no |
| Sobic.001G012900 | 701.8120 | 0.8043 | 0.3563 | 2.2571 | 0.0239995 | 0.182033 | no |
| Sobic.010G095700 | 7567.1534 | -0.6284 | 0.2784 | -2.2571 | 0.0240046 | 0.182033 | no |
| Sobic.004G325400 | 678.7199 | 0.7965 | 0.3530 | 2.2565 | 0.0240395 | 0.1822315 | no |
| Sobic.005G158100 | 502.2482 | -0.7230 | 0.3205 | -2.2560 | 0.0240686 | 0.1823869 | no |
| Sobic.004G179000 | 31.3463 | -2.1855 | 0.9689 | -2.2557 | 0.0240894 | 0.1824786 | no |
| Sobic.003G385500 | 184.7563 | 2.2821 | 1.0120 | 2.2550 | 0.0241341 | 0.182752 | no |
| Sobic.002G166700 | 241.6172 | -0.6001 | 0.2662 | -2.2544 | 0.0241719 | 0.1829725 | no |
| Sobic.003G391500 | 138.9583 | 0.9211 | 0.4087 | 2.2540 | 0.0241988 | 0.1830262 | no |
| Sobic.007G002900 | 3853.7059 | -0.7553 | 0.3351 | -2.2539 | 0.0242035 | 0.1830262 | no |

| Sobic.006G140100 | 10.2589 | 2.2810 | 1.0120 | 2.2539 | 0.024205 | 0.1830262 | no |
| --- | --- | --- | --- | --- | --- | --- | --- |
| Sobic.004G001400 | 4978.3654 | -0.5720 | 0.2538 | -2.2536 | 0.024223 | 0.1830944 | no |
| Sobic.010G225500 | 108.3020 | 1.2128 | 0.5382 | 2.2534 | 0.0242314 | 0.1830944 | no |
| Sobic.007G207100 | 37.8946 | -1.5615 | 0.6931 | -2.2531 | 0.0242538 | 0.1831975 | no |
| Sobic.001G454401 | 70.1672 | 1.8253 | 0.8104 | 2.2525 | 0.0242909 | 0.1833347 | no |
| Sobic.003G202300 | 317.4990 | -1.2078 | 0.5362 | -2.2524 | 0.0242969 | 0.1833347 | no |
| Sobic.010G239600 | 25445.6254 | -1.0164 | 0.4512 | -2.2524 | 0.0242993 | 0.1833347 | no |
| Sobic.009G237200 | 1108.8956 | -0.8260 | 0.3667 | -2.2522 | 0.0243071 | 0.1833347 | no |
| Sobic.002G112300 | 6930.3992 | -1.1541 | 0.5125 | -2.2520 | 0.0243204 | 0.1833347 | no |
| Sobic.004G269100 | 96.4963 | 1.0009 | 0.4445 | 2.2520 | 0.0243241 | 0.1833347 | no |
| Sobic.002G041200 | 1737.4560 | -4.2723 | 1.8973 | -2.2518 | 0.0243344 | 0.1833468 | no |
| Sobic.009G022500 | 169.2259 | 1.9280 | 0.8565 | 2.2511 | 0.0243817 | 0.1835893 | no |
| Sobic.003G297700 | 92.4222 | 0.9665 | 0.4294 | 2.2510 | 0.024384 | 0.1835893 | no |
| Sobic.001G192800 | 1761.3049 | -1.1265 | 0.5006 | -2.2505 | 0.0244184 | 0.1837826 | no |
| Sobic.001G037800 | 86.9094 | -1.1755 | 0.5226 | -2.2496 | 0.0244771 | 0.1840742 | no |
| Sobic.005G086700 | 6.2191 | -2.9837 | 1.3265 | -2.2494 | 0.0244888 | 0.1840742 | no |
| Sobic.004G311100 | 22970.8648 | -0.9560 | 0.4250 | -2.2494 | 0.0244902 | 0.1840742 | no |
| Sobic.003G311200 | 16.1946 | 2.4232 | 1.0774 | 2.2492 | 0.0244997 | 0.1840742 | no |
| Sobic.009G213300 | 919.8051 | -0.7085 | 0.3150 | -2.2492 | 0.0245007 | 0.1840742 | no |
| Sobic.002G201400 | 2456.6197 | -1.0662 | 0.4741 | -2.2488 | 0.0245265 | 0.1841712 | no |
| Sobic.002G254000 | 109.6096 | -1.5861 | 0.7054 | -2.2485 | 0.0245455 | 0.1841712 | no |
| Sobic.005G055150 | 1022.5122 | 0.8921 | 0.3968 | 2.2483 | 0.0245552 | 0.1841712 | no |
| Sobic.001G303000 | 69.0822 | -2.2693 | 1.0093 | -2.2483 | 0.0245583 | 0.1841712 | no |
| Sobic.003G245201 | 64.4385 | 1.6130 | 0.7175 | 2.2481 | 0.024571 | 0.1841712 | no |
| Sobic.009G042300 | 470.0605 | 0.6183 | 0.2750 | 2.2481 | 0.0245711 | 0.1841712 | no |
| Sobic.008G032100 | 1699.7481 | -0.7288 | 0.3242 | -2.2480 | 0.0245748 | 0.1841712 | no |
| Sobic.007G013000 | 352.3279 | -0.5948 | 0.2646 | -2.2478 | 0.0245883 | 0.1841922 | no |
| Sobic.008G015200 | 650.2802 | 0.6220 | 0.2767 | 2.2477 | 0.0245951 | 0.1841922 | no |
| Sobic.001G111000 | 237.7981 | 1.4112 | 0.6279 | 2.2474 | 0.0246176 | 0.1842898 | no |
| Sobic.001G523000 | 226.8427 | -0.7638 | 0.3399 | -2.2472 | 0.0246256 | 0.1842898 | no |
| Sobic.010G095100 | 130.6034 | -1.0022 | 0.4461 | -2.2466 | 0.0246688 | 0.1845365 | no |
| Sobic.004G034500 | 4835.6726 | -0.9853 | 0.4386 | -2.2464 | 0.024676 | 0.1845365 | no |
| Sobic.002G216500 | 407.4962 | 0.9056 | 0.4032 | 2.2458 | 0.0247164 | 0.1846609 | no |
| Sobic.006G139300 | 18.6819 | -1.7295 | 0.7701 | -2.2457 | 0.0247225 | 0.1846609 | no |
| Sobic.010G003900 | 93.1506 | 1.0015 | 0.4460 | 2.2457 | 0.0247238 | 0.1846609 | no |
| Sobic.004G178400 | 290.2606 | -0.7723 | 0.3439 | -2.2456 | 0.0247277 | 0.1846609 | no |
| Sobic.002G337800 | 148.1247 | 1.8501 | 0.8241 | 2.2450 | 0.0247697 | 0.184836 | no |
| Sobic.005G084850 | 41.8491 | -1.2144 | 0.5410 | -2.2449 | 0.0247776 | 0.184836 | no |
| Sobic.008G113000 | 6036.9348 | 0.5042 | 0.2246 | 2.2446 | 0.0247913 | 0.184836 | no |
| Sobic.008G062400 | 1326.3805 | -0.7830 | 0.3488 | -2.2446 | 0.0247938 | 0.184836 | no |
| Sobic.001G127200 | 1838.8515 | 0.5321 | 0.2370 | 2.2446 | 0.024795 | 0.184836 | no |
| Sobic.002G044500 | 66.4222 | -0.9274 | 0.4133 | -2.2440 | 0.024835 | 0.185069 | no |
| Sobic.005G112566 | 687.4678 | -0.7463 | 0.3327 | -2.2432 | 0.0248821 | 0.1853544 | no |
| Sobic.002G341500 | 2003.0055 | -0.9511 | 0.4240 | -2.2430 | 0.0248997 | 0.1853575 | no |
| Sobic.009G049900 | 993.2359 | -0.8689 | 0.3874 | -2.2428 | 0.0249111 | 0.1853575 | no |
| Sobic.003G337000 | 13634.8836 | -0.8174 | 0.3645 | -2.2426 | 0.0249224 | 0.1853575 | no |
| Sobic.010G176800 | 17.2333 | 2.5534 | 1.1386 | 2.2426 | 0.0249236 | 0.1853575 | no |
| Sobic.002G251400 | 937.2803 | -0.9848 | 0.4392 | -2.2425 | 0.0249315 | 0.1853575 | no |
| Sobic.001G184900 | 528.9277 | -0.8240 | 0.3675 | -2.2424 | 0.0249352 | 0.1853575 | no |
| Sobic.010G222800 | 23.7139 | 2.0695 | 0.9230 | 2.2422 | 0.0249514 | 0.1853913 | no |
| Sobic.002G047400 | 59204.6706 | -1.0473 | 0.4671 | -2.2421 | 0.0249574 | 0.1853913 | no |
| Sobic.002G186600 | 91.5698 | -1.3016 | 0.5806 | -2.2418 | 0.0249722 | 0.1854362 | no |
| Sobic.005G126401 | 70.4788 | -1.5635 | 0.6976 | -2.2414 | 0.025001 | 0.1855847 | no |
| Sobic.002G213300 | 808.0387 | 1.3580 | 0.6059 | 2.2412 | 0.0250106 | 0.1855909 | no |
| Sobic.001G114400 | 1079.4002 | 0.7435 | 0.3318 | 2.2409 | 0.0250302 | 0.185671 | no |
| Sobic.010G250100 | 550.8016 | 1.3709 | 0.6118 | 2.2407 | 0.0250485 | 0.1857411 | no |
| Sobic.003G264100 | 2034.8591 | 0.6099 | 0.2722 | 2.2403 | 0.0250724 | 0.1858533 | no |

| Sobic.006G193900 | 1936.9412 | 0.8397 | 0.3748 | 2.2401 | 0.0250824 | 0.1858615 | no |
| --- | --- | --- | --- | --- | --- | --- | --- |
| Sobic.006G157600 | 103.6562 | 1.3756 | 0.6141 | 2.2400 | 0.0250917 | 0.1858652 | no |
| Sobic.002G252400 | 284.2055 | 0.8174 | 0.3650 | 2.2394 | 0.0251296 | 0.1860655 | no |
| Sobic.003G359400 | 43.4432 | 1.0876 | 0.4857 | 2.2391 | 0.0251511 | 0.1860655 | no |
| Sobic.006G046900 | 5.0767 | 3.7201 | 1.6615 | 2.2390 | 0.025153 | 0.1860655 | no |
| Sobic.001G483400 | 718.6636 | -0.5255 | 0.2347 | -2.2390 | 0.0251578 | 0.1860655 | no |
| Sobic.001G274900 | 185.7630 | 0.9133 | 0.4079 | 2.2389 | 0.0251628 | 0.1860655 | no |
| Sobic.010G046801 | 6.9283 | -2.5997 | 1.1612 | -2.2387 | 0.0251733 | 0.1860779 | no |
| Sobic.006G026600 | 1755.9824 | -1.0983 | 0.4907 | -2.2381 | 0.0252133 | 0.1863078 | no |
| Sobic.003G134300 | 9455.2580 | -0.8487 | 0.3793 | -2.2377 | 0.0252436 | 0.1864664 | no |
| Sobic.003G107300 | 96523.3387 | -1.2525 | 0.5599 | -2.2372 | 0.0252727 | 0.1865773 | no |
| Sobic.004G058500 | 470.5517 | 0.7847 | 0.3508 | 2.2370 | 0.0252834 | 0.1865773 | no |
| Sobic.006G032300 | 3344.1603 | 0.7641 | 0.3416 | 2.2370 | 0.0252891 | 0.1865773 | no |
| Sobic.010G085100 | 2814.5290 | -0.8588 | 0.3839 | -2.2369 | 0.025294 | 0.1865773 | no |
| Sobic.001G466300 | 120.7459 | -0.8030 | 0.3591 | -2.2365 | 0.0253174 | 0.1866034 | no |
| Sobic.005G018500 | 65.0864 | 2.4198 | 1.0820 | 2.2365 | 0.0253202 | 0.1866034 | no |
| Sobic.002G077100 | 1270.7277 | -1.0539 | 0.4713 | -2.2364 | 0.0253241 | 0.1866034 | no |
| Sobic.003G135500 | 55.4473 | 1.1356 | 0.5080 | 2.2353 | 0.0253977 | 0.1870162 | no |
| Sobic.001G039300 | 102.5797 | 1.1703 | 0.5236 | 2.2353 | 0.0253978 | 0.1870162 | no |
| Sobic.003G355500 | 2156.3178 | 1.5304 | 0.6849 | 2.2346 | 0.025442 | 0.1872766 | no |
| Sobic.007G116500 | 35.2653 | 2.0631 | 0.9235 | 2.2341 | 0.025475 | 0.1873235 | no |
| Sobic.001G275200 | 817.1260 | -0.8280 | 0.3706 | -2.2341 | 0.0254787 | 0.1873235 | no |
| Sobic.010G220700 | 133.5511 | -0.8681 | 0.3886 | -2.2340 | 0.0254828 | 0.1873235 | no |
| Sobic.002G397600 | 1660.3520 | 1.0517 | 0.4708 | 2.2340 | 0.0254839 | 0.1873235 | no |
| Sobic.006G140400 | 144.8643 | 1.0143 | 0.4542 | 2.2332 | 0.025538 | 0.1876105 | no |
| Sobic.004G268600 | 256.7031 | -0.9282 | 0.4156 | -2.2331 | 0.0255408 | 0.1876105 | no |
| Sobic.001G394400 | 5552.0291 | -0.8432 | 0.3778 | -2.2321 | 0.0256055 | 0.1880201 | no |
| Sobic.003G092500 | 1508.4223 | -1.2140 | 0.5440 | -2.2317 | 0.025634 | 0.1881316 | no |
| Sobic.006G155500 | 1347.7993 | -0.7566 | 0.3390 | -2.2316 | 0.025643 | 0.1881316 | no |
| Sobic.006G201900 | 23.7489 | 2.1138 | 0.9472 | 2.2315 | 0.0256474 | 0.1881316 | no |
| Sobic.005G123200 | 179.2251 | -1.4976 | 0.6713 | -2.2309 | 0.0256878 | 0.1883622 | no |
| Sobic.007G206100 | 291.7255 | -1.0942 | 0.4906 | -2.2305 | 0.025717 | 0.1885109 | no |
| Sobic.003G141000 | 1216.2396 | -0.7173 | 0.3217 | -2.2298 | 0.0257581 | 0.1887466 | no |
| Sobic.010G033300 | 494.6155 | 1.1813 | 0.5298 | 2.2296 | 0.025774 | 0.1887973 | no |
| Sobic.007G144300 | 28.7183 | 2.3137 | 1.0378 | 2.2294 | 0.0257891 | 0.1888425 | no |
| Sobic.009G073800 | 32.3018 | 1.4991 | 0.6725 | 2.2292 | 0.0258014 | 0.188867 | no |
| Sobic.002G008800 | 818.3103 | 0.7619 | 0.3419 | 2.2287 | 0.0258306 | 0.1890076 | no |
| Sobic.008G165200 | 28.4189 | 1.3887 | 0.6231 | 2.2286 | 0.0258385 | 0.1890076 | no |
| Sobic.007G198100 | 4513.1397 | 0.7969 | 0.3576 | 2.2281 | 0.0258766 | 0.1891598 | no |
| Sobic.009G112600 | 7440.4453 | 0.8102 | 0.3636 | 2.2280 | 0.0258773 | 0.1891598 | no |
| Sobic.010G267000 | 5896.3730 | -1.8303 | 0.8216 | -2.2278 | 0.0258968 | 0.1892367 | no |
| Sobic.009G069000 | 548.4984 | 0.7564 | 0.3396 | 2.2273 | 0.0259257 | 0.1892459 | no |
| Sobic.009G211100 | 33.3039 | 1.9852 | 0.8914 | 2.2272 | 0.0259346 | 0.1892459 | no |
| Sobic.005G034700 | 849.9463 | 0.8743 | 0.3926 | 2.2271 | 0.0259437 | 0.1892459 | no |
| Sobic.002G398100 | 141.2536 | 0.7678 | 0.3448 | 2.2270 | 0.0259498 | 0.1892459 | no |
| Sobic.001G040600 | 379.3558 | 1.6012 | 0.7190 | 2.2269 | 0.0259548 | 0.1892459 | no |
| Sobic.005G220200 | 1703.8428 | -0.8069 | 0.3624 | -2.2268 | 0.0259606 | 0.1892459 | no |
| Sobic.004G033900 | 1401.4595 | -1.2570 | 0.5645 | -2.2268 | 0.0259609 | 0.1892459 | no |
| Sobic.001G465900 | 220.1558 | -0.9227 | 0.4147 | -2.2246 | 0.0261056 | 0.1902356 | no |
| Sobic.006G213700 | 1404.3528 | -0.8922 | 0.4011 | -2.2241 | 0.0261395 | 0.1904163 | no |
| Sobic.006G002600 | 460.9227 | 0.6445 | 0.2898 | 2.2239 | 0.0261557 | 0.1904689 | no |
| Sobic.002G212000 | 386.2053 | 1.2534 | 0.5637 | 2.2236 | 0.0261761 | 0.1905515 | no |
| Sobic.004G211866 | 8414.4555 | 1.5607 | 0.7020 | 2.2231 | 0.0262087 | 0.1907228 | no |
| Sobic.005G195200 | 367.1278 | 1.5972 | 0.7185 | 2.2229 | 0.0262221 | 0.1907544 | no |
| Sobic.007G140800 | 3652.4193 | -1.0564 | 0.4758 | -2.2204 | 0.0263886 | 0.1918345 | no |
| Sobic.002G403700 | 555.2218 | -0.7869 | 0.3544 | -2.2203 | 0.0263956 | 0.1918345 | no |
| Sobic.004G306100 | 124.0740 | 1.4086 | 0.6344 | 2.2203 | 0.0263978 | 0.1918345 | no |

| Sobic.003G222200 | 380.7878 | 0.8945 | 0.4030 | 2.2199 | 0.0264267 | 0.1919778 | no |
| --- | --- | --- | --- | --- | --- | --- | --- |
| Sobic.010G151801 | 570.7376 | -1.0420 | 0.4695 | -2.2196 | 0.026449 | 0.192074 | no |
| Sobic.002G360000 | 228.7452 | -1.1965 | 0.5391 | -2.2193 | 0.0264633 | 0.1921116 | no |
| Sobic.003G152500 | 45.1674 | -1.0557 | 0.4757 | -2.2191 | 0.026479 | 0.1921597 | no |
| Sobic.010G076100 | 54.5551 | -1.5076 | 0.6794 | -2.2188 | 0.0264983 | 0.1922337 | no |
| Sobic.001G010300 | 496.4976 | 0.9300 | 0.4192 | 2.2185 | 0.0265241 | 0.1923546 | no |
| Sobic.001G079500 | 4503.8669 | -1.6606 | 0.7486 | -2.2181 | 0.0265452 | 0.1924412 | no |
| Sobic.006G226601 | 1102.8426 | -0.8374 | 0.3776 | -2.2174 | 0.0265948 | 0.192671 | no |
| Sobic.003G276800 | 19.6676 | 2.0519 | 0.9254 | 2.2173 | 0.026602 | 0.192671 | no |
| Sobic.004G322400 | 940.9826 | 0.7701 | 0.3473 | 2.2172 | 0.0266093 | 0.192671 | no |
| Sobic.006G059700 | 29.2478 | 2.8102 | 1.2675 | 2.2171 | 0.0266134 | 0.192671 | no |
| Sobic.010G125300 | 24.6019 | 2.8437 | 1.2830 | 2.2165 | 0.0266569 | 0.1928551 | no |
| Sobic.002G224100 | 95.2539 | 1.9235 | 0.8678 | 2.2164 | 0.0266626 | 0.1928551 | no |
| Sobic.001G451901 | 346.1851 | 0.8281 | 0.3736 | 2.2164 | 0.0266663 | 0.1928551 | no |
| Sobic.002G306300 | 55.3640 | 1.3721 | 0.6192 | 2.2159 | 0.0266974 | 0.1930035 | no |
| Sobic.007G080900 | 32.7177 | -1.8752 | 0.8463 | -2.2157 | 0.0267122 | 0.1930035 | no |
| Sobic.001G481201 | 2720.9018 | -1.4387 | 0.6494 | -2.2155 | 0.026726 | 0.1930035 | no |
| Sobic.001G122500 | 367.9061 | 0.8848 | 0.3994 | 2.2155 | 0.026728 | 0.1930035 | no |
| Sobic.010G247800 | 1498.3921 | 0.5890 | 0.2659 | 2.2154 | 0.0267326 | 0.1930035 | no |
| Sobic.007G226400 | 13.1875 | 2.2472 | 1.0144 | 2.2152 | 0.0267462 | 0.1930357 | no |
| Sobic.010G076700 | 292.0635 | 1.0015 | 0.4523 | 2.2145 | 0.026795 | 0.1932778 | no |
| Sobic.010G214100 | 1213.0572 | -0.6889 | 0.3111 | -2.2143 | 0.0268052 | 0.1932778 | no |
| Sobic.003G334000 | 2754.9192 | -0.7193 | 0.3248 | -2.2143 | 0.0268072 | 0.1932778 | no |
| Sobic.006G098500 | 4173.8757 | -0.6283 | 0.2838 | -2.2140 | 0.0268254 | 0.1933424 | no |
| Sobic.002G116500 | 147.7756 | 0.6766 | 0.3057 | 2.2136 | 0.0268538 | 0.1934815 | no |
| Sobic.003G253700 | 127.2209 | 1.1896 | 0.5375 | 2.2133 | 0.026876 | 0.1935753 | no |
| Sobic.004G180500 | 132.3390 | 1.1774 | 0.5321 | 2.2128 | 0.0269094 | 0.1937199 | no |
| Sobic.003G356200 | 1477.5555 | -0.7438 | 0.3362 | -2.2128 | 0.0269145 | 0.1937199 | no |
| Sobic.010G216400 | 25.3516 | 1.4320 | 0.6472 | 2.2124 | 0.0269404 | 0.1937766 | no |
| Sobic.005G228900 | 1641.4028 | 0.8891 | 0.4019 | 2.2123 | 0.0269437 | 0.1937766 | no |
| Sobic.003G037800 | 667.3028 | 0.9506 | 0.4297 | 2.2122 | 0.0269504 | 0.1937766 | no |
| Sobic.006G192200 | 184.4033 | 1.3176 | 0.5956 | 2.2121 | 0.0269591 | 0.1937766 | no |
| Sobic.006G094000 | 7.2151 | -2.6462 | 1.1963 | -2.2119 | 0.0269703 | 0.1937909 | no |
| Sobic.004G234500 | 34.8758 | 2.0788 | 0.9400 | 2.2114 | 0.0270048 | 0.1939731 | no |
| Sobic.006G264300 | 2837.7427 | 1.0409 | 0.4708 | 2.2111 | 0.0270291 | 0.1940228 | no |
| Sobic.003G197400 | 1160.7138 | -1.5896 | 0.7189 | -2.2110 | 0.0270388 | 0.1940228 | no |
| Sobic.002G056200 | 2695.8897 | -0.6786 | 0.3069 | -2.2109 | 0.0270394 | 0.1940228 | no |
| Sobic.002G116800 | 599.2631 | 0.6924 | 0.3132 | 2.2105 | 0.0270723 | 0.1941934 | no |
| Sobic.009G240000 | 110.7331 | -0.9138 | 0.4135 | -2.2096 | 0.0271315 | 0.1944448 | no |
| Sobic.006G194300 | 551.3061 | 1.7857 | 0.8082 | 2.2096 | 0.0271355 | 0.1944448 | no |
| Sobic.001G056601 | 741.7564 | -0.8752 | 0.3961 | -2.2095 | 0.027138 | 0.1944448 | no |
| Sobic.004G201700 | 13860.8190 | -0.7176 | 0.3248 | -2.2094 | 0.0271443 | 0.1944448 | no |
| Sobic.010G087500 | 15.7892 | 2.4627 | 1.1149 | 2.2090 | 0.0271774 | 0.1946161 | no |
| Sobic.007G225500 | 1441.2995 | -0.8937 | 0.4046 | -2.2087 | 0.0271939 | 0.1946681 | no |
| Sobic.001G111366 | 1669.3370 | -1.0825 | 0.4902 | -2.2082 | 0.02723 | 0.1948604 | no |
| Sobic.010G064600 | 1211.4367 | 0.6057 | 0.2743 | 2.2081 | 0.0272396 | 0.194863 | no |
| Sobic.001G437400 | 1256.1744 | 0.7937 | 0.3595 | 2.2076 | 0.0272706 | 0.1950188 | no |
| Sobic.001G309200 | 1018.6389 | 0.8504 | 0.3853 | 2.2074 | 0.0272875 | 0.1950336 | no |
| Sobic.001G001900 | 1637.3556 | 0.9212 | 0.4174 | 2.2073 | 0.0272912 | 0.1950336 | no |
| Sobic.010G064100 | 249.6495 | -1.2598 | 0.5708 | -2.2071 | 0.0273102 | 0.1951031 | no |
| Sobic.010G100300 | 252.2003 | -1.3675 | 0.6197 | -2.2067 | 0.0273366 | 0.1952258 | no |
| Sobic.007G149500 | 34.3363 | -1.8515 | 0.8391 | -2.2065 | 0.0273496 | 0.1952526 | no |
| Sobic.003G237201 | 157.7641 | 1.3345 | 0.6049 | 2.2063 | 0.0273613 | 0.1952699 | no |
| Sobic.004G035400 | 219.1960 | 0.8077 | 0.3662 | 2.2060 | 0.0273863 | 0.1953519 | no |
| Sobic.003G316350 | 55.5035 | -1.8425 | 0.8353 | -2.2057 | 0.027405 | 0.1953519 | no |
| Sobic.009G113800 | 2997.7523 | -0.9125 | 0.4137 | -2.2057 | 0.0274078 | 0.1953519 | no |
| Sobic.006G001800 | 197.1077 | 0.8338 | 0.3781 | 2.2053 | 0.0274309 | 0.1953519 | no |

| Sobic.008G078700 | 26.8251 | 2.6411 | 1.1976 | 2.2053 | 0.0274339 | 0.1953519 | no |
| --- | --- | --- | --- | --- | --- | --- | --- |
| Sobic.008G082100 | 340.4694 | -0.7586 | 0.3440 | -2.2053 | 0.0274357 | 0.1953519 | no |
| Sobic.003G435500 | 742.0669 | 0.6899 | 0.3128 | 2.2052 | 0.0274376 | 0.1953519 | no |
| Sobic.003G397200 | 2946.2624 | -1.0268 | 0.4657 | -2.2051 | 0.027448 | 0.1953599 | no |
| Sobic.009G016600 | 22666.7705 | -0.7491 | 0.3398 | -2.2048 | 0.0274659 | 0.1954212 | no |
| Sobic.008G088857 | 838.4238 | -0.6157 | 0.2793 | -2.2044 | 0.0274996 | 0.1955953 | no |
| Sobic.009G219100 | 49473.1498 | -0.9210 | 0.4178 | -2.2041 | 0.0275156 | 0.1956426 | no |
| Sobic.006G063800 | 50.6360 | 1.8317 | 0.8311 | 2.2040 | 0.0275276 | 0.1956606 | no |
| Sobic.004G247200 | 9684.5131 | 0.7701 | 0.3495 | 2.2038 | 0.0275367 | 0.1956606 | no |
| Sobic.004G298100 | 9044.0719 | -1.2314 | 0.5589 | -2.2033 | 0.0275759 | 0.1958734 | no |
| Sobic.004G062500 | 2135.6259 | 0.6510 | 0.2956 | 2.2027 | 0.0276194 | 0.1961161 | no |
| Sobic.001G145900 | 598.0292 | 0.5905 | 0.2681 | 2.2022 | 0.0276531 | 0.1962897 | no |
| Sobic.002G260000 | 268.0010 | 1.6602 | 0.7540 | 2.2019 | 0.0276732 | 0.1963664 | no |
| Sobic.001G050200 | 1159.9909 | 0.8139 | 0.3697 | 2.2017 | 0.0276875 | 0.1964013 | no |
| Sobic.001G324900 | 610.5562 | 0.9923 | 0.4508 | 2.2013 | 0.0277126 | 0.1965137 | no |
| Sobic.010G121300 | 4915.7220 | -0.5455 | 0.2479 | -2.2007 | 0.0277576 | 0.1967667 | no |
| Sobic.003G406300 | 620.7716 | 1.0016 | 0.4553 | 2.2000 | 0.0278071 | 0.1970508 | no |
| Sobic.001G536901 | 1743.4072 | -0.9011 | 0.4097 | -2.1992 | 0.0278646 | 0.1973922 | no |
| Sobic.008G122400 | 17.0875 | 2.2923 | 1.0425 | 2.1988 | 0.027895 | 0.197454 | no |
| Sobic.003G106600 | 943.2870 | -0.8771 | 0.3989 | -2.1987 | 0.0279025 | 0.197454 | no |
| Sobic.001G484500 | 5.1872 | 3.7565 | 1.7086 | 2.1986 | 0.0279062 | 0.197454 | no |
| Sobic.009G180800 | 2064.2236 | -1.1773 | 0.5355 | -2.1985 | 0.0279108 | 0.197454 | no |
| Sobic.008G081400 | 619.3423 | -1.1184 | 0.5088 | -2.1982 | 0.0279338 | 0.1975507 | no |
| Sobic.006G133800 | 1353.6936 | -0.6941 | 0.3158 | -2.1979 | 0.0279573 | 0.1976324 | no |
| Sobic.003G404000 | 19290.1775 | 0.6907 | 0.3142 | 2.1978 | 0.0279641 | 0.1976324 | no |
| Sobic.003G070025 | 10.3401 | -2.6925 | 1.2252 | -2.1976 | 0.0279774 | 0.1976602 | no |
| Sobic.005G111550 | 77.0518 | -0.9048 | 0.4118 | -2.1970 | 0.0280192 | 0.1978853 | no |
| Sobic.009G069800 | 478.2819 | -0.9411 | 0.4284 | -2.1968 | 0.0280354 | 0.1978853 | no |
| Sobic.009G055200 | 222.5857 | 0.6411 | 0.2918 | 2.1968 | 0.0280374 | 0.1978853 | no |
| Sobic.004G121900 | 5.4943 | -3.6096 | 1.6435 | -2.1964 | 0.0280666 | 0.1980249 | no |
| Sobic.003G235500 | 5957.4478 | -0.9953 | 0.4533 | -2.1958 | 0.0281036 | 0.1982199 | no |
| Sobic.002G221700 | 156.3495 | -1.0191 | 0.4642 | -2.1953 | 0.0281409 | 0.1984166 | no |
| Sobic.003G176600 | 450.9823 | -0.7852 | 0.3577 | -2.1949 | 0.0281683 | 0.19854 | no |
| Sobic.001G323701 | 3050.5211 | -1.3462 | 0.6134 | -2.1948 | 0.0281773 | 0.19854 | no |
| Sobic.007G130800 | 3783.6865 | 0.7146 | 0.3257 | 2.1944 | 0.0282081 | 0.1986909 | no |
| Sobic.001G210900 | 6.2903 | 4.8522 | 2.2118 | 2.1938 | 0.0282506 | 0.1989241 | no |
| Sobic.001G245000 | 93.9369 | 0.9527 | 0.4345 | 2.1928 | 0.0283192 | 0.1993402 | no |
| Sobic.004G130932 | 711.6167 | -0.6176 | 0.2817 | -2.1927 | 0.0283287 | 0.1993407 | no |
| Sobic.001G398300 | 235.5438 | -0.7592 | 0.3463 | -2.1925 | 0.0283407 | 0.1993588 | no |
| Sobic.008G078900 | 2286.2854 | -0.9574 | 0.4369 | -2.1914 | 0.0284252 | 0.1998863 | no |
| Sobic.003G285500 | 400.2446 | -1.2438 | 0.5678 | -2.1907 | 0.0284713 | 0.2001439 | no |
| Sobic.004G197600 | 862.9599 | 1.1208 | 0.5117 | 2.1904 | 0.028494 | 0.2002367 | no |
| Sobic.002G327300 | 88.9709 | 1.2050 | 0.5503 | 2.1897 | 0.0285456 | 0.2004427 | no |
| Sobic.003G060300 | 134.3060 | 0.9794 | 0.4473 | 2.1897 | 0.0285475 | 0.2004427 | no |
| Sobic.006G264900 | 34570.4719 | -1.2079 | 0.5517 | -2.1894 | 0.0285673 | 0.2004427 | no |
| Sobic.010G038200 | 29.8906 | 1.3956 | 0.6374 | 2.1894 | 0.028569 | 0.2004427 | no |
| Sobic.002G252900 | 493.3204 | 1.0126 | 0.4625 | 2.1894 | 0.0285709 | 0.2004427 | no |
| Sobic.010G053800 | 1597.0154 | 0.6085 | 0.2780 | 2.1890 | 0.0285993 | 0.2005738 | no |
| Sobic.007G179400 | 75.1356 | -1.5776 | 0.7208 | -2.1888 | 0.0286086 | 0.2005738 | no |
| Sobic.008G033900 | 591.3019 | 0.7788 | 0.3559 | 2.1886 | 0.0286286 | 0.2006478 | no |
| Sobic.003G423600 | 83.1737 | 0.9177 | 0.4194 | 2.1881 | 0.028663 | 0.2008222 | no |
| Sobic.002G426700 | 99.2916 | -1.0370 | 0.4740 | -2.1879 | 0.0286782 | 0.2008617 | no |
| Sobic.002G125300 | 261.9709 | -1.0154 | 0.4643 | -2.1870 | 0.0287433 | 0.2012506 | no |
| Sobic.002G302500 | 62.6305 | 1.6246 | 0.7429 | 2.1868 | 0.0287542 | 0.20126 | no |
| Sobic.002G141100 | 20.5878 | -2.1006 | 0.9607 | -2.1866 | 0.0287687 | 0.2012756 | no |
| Sobic.005G160400 | 3601.8816 | -0.5398 | 0.2469 | -2.1865 | 0.0287785 | 0.2012756 | no |
| Sobic.008G081500 | 652.8307 | 1.1816 | 0.5404 | 2.1864 | 0.028786 | 0.2012756 | no |

| Sobic.009G014100 | 258.1001 | -0.9467 | 0.4330 | -2.1863 | 0.0287946 | 0.2012756 | no |
| --- | --- | --- | --- | --- | --- | --- | --- |
| Sobic.008G137800 | 47.9338 | 1.1823 | 0.5408 | 2.1860 | 0.0288176 | 0.2013184 | no |
| Sobic.010G072500 | 35.7402 | 1.6172 | 0.7398 | 2.1859 | 0.0288198 | 0.2013184 | no |
| Sobic.001G001300 | 1471.1041 | -1.0192 | 0.4663 | -2.1857 | 0.0288412 | 0.2013724 | no |
| Sobic.004G317600 | 3244.1175 | -0.8948 | 0.4094 | -2.1856 | 0.0288466 | 0.2013724 | no |
| Sobic.002G416500 | 12.3962 | -1.7893 | 0.8190 | -2.1849 | 0.0288965 | 0.2016543 | no |
| Sobic.003G412900 | 20412.5282 | -0.7809 | 0.3575 | -2.1844 | 0.028933 | 0.2018416 | no |
| Sobic.007G051400 | 1228.3756 | 0.6024 | 0.2759 | 2.1837 | 0.0289842 | 0.202071 | no |
| Sobic.008G088863 | 56.4397 | -1.9098 | 0.8746 | -2.1836 | 0.0289899 | 0.202071 | no |
| Sobic.002G016300 | 831.7531 | -0.7213 | 0.3304 | -2.1835 | 0.0290013 | 0.202071 | no |
| Sobic.003G170200 | 7850.1162 | -0.6173 | 0.2827 | -2.1834 | 0.0290042 | 0.202071 | no |
| Sobic.006G038500 | 1425.8990 | -0.5975 | 0.2737 | -2.1827 | 0.0290611 | 0.2023085 | no |
| Sobic.001G191000 | 6324.3390 | -1.1085 | 0.5079 | -2.1827 | 0.0290616 | 0.2023085 | no |
| Sobic.003G314700 | 901.8136 | 0.6927 | 0.3174 | 2.1826 | 0.029067 | 0.2023085 | no |
| Sobic.003G370900 | 1076.8928 | -0.5842 | 0.2677 | -2.1824 | 0.0290777 | 0.2023159 | no |
| Sobic.006G077300 | 1989.6563 | 0.6928 | 0.3175 | 2.1822 | 0.0290931 | 0.2023562 | no |
| Sobic.008G095000 | 1302.4819 | 1.4236 | 0.6525 | 2.1819 | 0.0291174 | 0.2024046 | no |
| Sobic.001G487000 | 11.6548 | 2.6549 | 1.2168 | 2.1819 | 0.0291192 | 0.2024046 | no |
| Sobic.008G161700 | 3008.0277 | -1.1843 | 0.5429 | -2.1816 | 0.0291357 | 0.2024234 | no |
| Sobic.009G155200 | 754.9150 | 0.9026 | 0.4138 | 2.1815 | 0.0291455 | 0.2024234 | no |
| Sobic.006G204200 | 639.9969 | -0.6267 | 0.2873 | -2.1814 | 0.0291507 | 0.2024234 | no |
| Sobic.004G244300 | 274.8237 | 1.3373 | 0.6131 | 2.1811 | 0.0291755 | 0.2024682 | no |
| Sobic.001G392400 | 268.4685 | -1.0070 | 0.4617 | -2.1811 | 0.0291764 | 0.2024682 | no |
| Sobic.010G131000 | 1541.6997 | -0.7010 | 0.3214 | -2.1809 | 0.0291911 | 0.2025033 | no |
| Sobic.002G166400 | 77.0462 | -1.0537 | 0.4833 | -2.1803 | 0.0292319 | 0.2027203 | no |
| Sobic.009G038701 | 6619.5888 | -0.7568 | 0.3472 | -2.1801 | 0.0292527 | 0.2027977 | no |
| Sobic.007G023100 | 20.2127 | -1.6037 | 0.7358 | -2.1796 | 0.0292887 | 0.2029803 | no |
| Sobic.006G048600 | 702.6538 | -0.7115 | 0.3265 | -2.1794 | 0.0293053 | 0.2030144 | no |
| Sobic.001G237000 | 1241.0457 | 0.7529 | 0.3455 | 2.1793 | 0.0293129 | 0.2030144 | no |
| Sobic.009G242700 | 68.1721 | -2.6305 | 1.2073 | -2.1789 | 0.029338 | 0.2031004 | no |
| Sobic.006G130100 | 143.7176 | 1.1287 | 0.5180 | 2.1788 | 0.0293445 | 0.2031004 | no |
| Sobic.004G012400 | 518.7957 | 1.0481 | 0.4811 | 2.1787 | 0.0293564 | 0.2031155 | no |
| Sobic.002G274800 | 1106.5886 | 1.2653 | 0.5808 | 2.1785 | 0.0293688 | 0.203135 | no |
| Sobic.003G404900 | 98.8482 | -0.9418 | 0.4325 | -2.1774 | 0.029452 | 0.2036439 | no |
| Sobic.004G300300 | 221.0327 | 2.6941 | 1.2374 | 2.1772 | 0.0294648 | 0.2036655 | no |
| Sobic.003G024400 | 2536.4221 | -1.0205 | 0.4688 | -2.1770 | 0.0294837 | 0.2036936 | no |
| Sobic.001G046800 | 46.1546 | -1.4813 | 0.6805 | -2.1768 | 0.0294958 | 0.2036936 | no |
| Sobic.005G039700 | 723.3734 | -0.8454 | 0.3884 | -2.1768 | 0.0294979 | 0.2036936 | no |
| Sobic.005G131600 | 160.2986 | 1.4435 | 0.6632 | 2.1766 | 0.0295109 | 0.2037107 | no |
| Sobic.004G343300 | 6988.7142 | -1.1635 | 0.5346 | -2.1765 | 0.0295197 | 0.2037107 | no |
| Sobic.007G037900 | 3744.4416 | -0.8357 | 0.3841 | -2.1758 | 0.0295672 | 0.2038631 | no |
| Sobic.007G178300 | 824.9808 | -0.6581 | 0.3024 | -2.1758 | 0.0295699 | 0.2038631 | no |
| Sobic.001G007400 | 678.2319 | -0.5128 | 0.2357 | -2.1757 | 0.0295758 | 0.2038631 | no |
| Sobic.008G095900 | 36.0043 | 2.4100 | 1.1078 | 2.1755 | 0.0295905 | 0.2038631 | no |
| Sobic.003G440700 | 389.8766 | -0.7096 | 0.3262 | -2.1755 | 0.0295955 | 0.2038631 | no |
| Sobic.004G272500 | 323.4056 | -0.9044 | 0.4158 | -2.1753 | 0.0296078 | 0.2038631 | no |
| Sobic.009G190900 | 32.3382 | -1.5746 | 0.7238 | -2.1753 | 0.0296094 | 0.2038631 | no |
| Sobic.003G196400 | 2347.1253 | 1.2163 | 0.5592 | 2.1750 | 0.0296318 | 0.2039017 | no |
| Sobic.001G342400 | 58.0329 | -1.7000 | 0.7816 | -2.1749 | 0.0296343 | 0.2039017 | no |
| Sobic.008G003300 | 166.1326 | -0.6810 | 0.3131 | -2.1748 | 0.0296441 | 0.2039024 | no |
| Sobic.010G190600 | 27115.7010 | -1.0193 | 0.4687 | -2.1745 | 0.0296698 | 0.2039928 | no |
| Sobic.002G299900 | 1594.2189 | -0.7016 | 0.3227 | -2.1744 | 0.0296766 | 0.2039928 | no |
| Sobic.009G067000 | 4283.9342 | 0.5601 | 0.2576 | 2.1740 | 0.0297048 | 0.2041203 | no |
| Sobic.002G421600 | 527.3377 | -1.8337 | 0.8440 | -2.1726 | 0.0298093 | 0.2046908 | no |
| Sobic.001G270200 | 15.9012 | 2.1799 | 1.0034 | 2.1726 | 0.0298097 | 0.2046908 | no |
| Sobic.003G425500 | 3841.3183 | -1.1462 | 0.5276 | -2.1725 | 0.029817 | 0.2046908 | no |
| Sobic.004G211200 | 5299.3507 | -1.1319 | 0.5211 | -2.1722 | 0.0298376 | 0.2047661 | no |

| Sobic.003G348400 | 277.6976 | 1.0892 | 0.5015 | 2.1717 | 0.0298772 | 0.2049705 | no |
| --- | --- | --- | --- | --- | --- | --- | --- |
| Sobic.003G012400 | 162.5132 | -2.2726 | 1.0466 | -2.1714 | 0.0299025 | 0.205078 | no |
| Sobic.004G192500 | 79.1382 | 1.1867 | 0.5467 | 2.1707 | 0.029953 | 0.205357 | no |
| Sobic.004G296200 | 216.7863 | 1.0259 | 0.4727 | 2.1701 | 0.0299965 | 0.2055001 | no |
| Sobic.001G052000 | 7252.8694 | -1.8099 | 0.8341 | -2.1697 | 0.0300288 | 0.2055001 | no |
| Sobic.003G286000 | 10.5299 | -1.7782 | 0.8196 | -2.1695 | 0.0300437 | 0.2055001 | no |
| Sobic.005G152100 | 101.4350 | -1.3975 | 0.6442 | -2.1694 | 0.0300488 | 0.2055001 | no |
| Sobic.010G168900 | 316.5097 | 1.3580 | 0.6259 | 2.1694 | 0.0300489 | 0.2055001 | no |
| Sobic.005G179500 | 512.7590 | -0.9433 | 0.4348 | -2.1694 | 0.0300493 | 0.2055001 | no |
| Sobic.002G386400 | 22291.2316 | -0.8092 | 0.3730 | -2.1694 | 0.0300496 | 0.2055001 | no |
| Sobic.006G148900 | 119.1712 | 1.7085 | 0.7875 | 2.1694 | 0.0300518 | 0.2055001 | no |
| Sobic.009G176800 | 779.8969 | -1.0032 | 0.4625 | -2.1690 | 0.0300799 | 0.2056257 | no |
| Sobic.001G318900 | 1693.9486 | 1.1020 | 0.5081 | 2.1689 | 0.030091 | 0.2056348 | no |
| Sobic.001G158900 | 1801.3609 | -0.6994 | 0.3226 | -2.1681 | 0.0301514 | 0.205981 | no |
| Sobic.002G183200 | 132.0895 | 2.0892 | 0.9637 | 2.1679 | 0.030165 | 0.2060068 | no |
| Sobic.001G058500 | 844.5419 | -0.9851 | 0.4545 | -2.1677 | 0.0301813 | 0.2060519 | no |
| Sobic.002G140800 | 53.6355 | 1.1709 | 0.5402 | 2.1675 | 0.0301986 | 0.2060644 | no |
| Sobic.002G347900 | 34.8700 | 1.7756 | 0.8192 | 2.1674 | 0.0302027 | 0.2060644 | no |
| Sobic.002G211000 | 61725.9393 | -1.1174 | 0.5156 | -2.1673 | 0.0302141 | 0.2060754 | no |
| Sobic.001G084100 | 2495.3007 | 0.8142 | 0.3757 | 2.1670 | 0.0302357 | 0.2061559 | no |
| Sobic.003G270600 | 3254.9043 | 0.7083 | 0.3269 | 2.1667 | 0.0302563 | 0.2062298 | no |
| Sobic.001G388800 | 64.5022 | 1.5366 | 0.7093 | 2.1665 | 0.0302744 | 0.2062863 | no |
| Sobic.001G261400 | 8.6794 | 2.7981 | 1.2918 | 2.1661 | 0.0303068 | 0.2064365 | no |
| Sobic.007G136200 | 2568.9377 | -0.7492 | 0.3459 | -2.1659 | 0.030316 | 0.2064365 | no |
| Sobic.009G118900 | 615.6093 | 0.5717 | 0.2640 | 2.1654 | 0.0303558 | 0.2066406 | no |
| Sobic.002G349900 | 151.1410 | 0.8016 | 0.3703 | 2.1649 | 0.030397 | 0.2068547 | no |
| Sobic.004G261600 | 657.3097 | 0.7622 | 0.3521 | 2.1646 | 0.0304216 | 0.2068989 | no |
| Sobic.010G278700 | 332.7345 | 0.7953 | 0.3674 | 2.1645 | 0.0304231 | 0.2068989 | no |
| Sobic.001G251100 | 2093.4524 | 0.5160 | 0.2384 | 2.1642 | 0.0304524 | 0.2070052 | no |
| Sobic.002G070200 | 1268.9619 | -1.3429 | 0.6205 | -2.1641 | 0.0304584 | 0.2070052 | no |
| Sobic.004G289400 | 151.3299 | 0.9841 | 0.4548 | 2.1637 | 0.0304855 | 0.2070832 | no |
| Sobic.001G207500 | 2026.8409 | -0.5602 | 0.2589 | -2.1636 | 0.0304921 | 0.2070832 | no |
| Sobic.002G328500 | 484.7966 | 0.9628 | 0.4450 | 2.1635 | 0.0304993 | 0.2070832 | no |
| Sobic.006G169400 | 32.5426 | -2.1657 | 1.0013 | -2.1629 | 0.0305484 | 0.2072586 | no |
| Sobic.005G068700 | 7053.9693 | -0.8423 | 0.3895 | -2.1629 | 0.03055 | 0.2072586 | no |
| Sobic.003G172250 | 96.3580 | -0.9911 | 0.4582 | -2.1627 | 0.0305606 | 0.2072586 | no |
| Sobic.006G106300 | 192.3892 | 1.0143 | 0.4690 | 2.1627 | 0.0305669 | 0.2072586 | no |
| Sobic.002G220000 | 8.9394 | -3.2645 | 1.5095 | -2.1626 | 0.0305743 | 0.2072586 | no |
| Sobic.003G055200 | 80.8000 | 1.3913 | 0.6436 | 2.1618 | 0.0306322 | 0.2075845 | no |
| Sobic.006G245000 | 897.7705 | 0.7005 | 0.3241 | 2.1615 | 0.0306545 | 0.2076347 | no |
| Sobic.004G312800 | 2785.6970 | -0.8999 | 0.4164 | -2.1615 | 0.0306594 | 0.2076347 | no |
| Sobic.005G150350 | 98.6014 | 0.7814 | 0.3615 | 2.1613 | 0.0306692 | 0.2076347 | no |
| Sobic.003G136500 | 71.8425 | 1.8122 | 0.8386 | 2.1611 | 0.0306891 | 0.2076571 | no |
| Sobic.006G167800 | 5937.7199 | -1.2944 | 0.5990 | -2.1610 | 0.0306922 | 0.2076571 | no |
| Sobic.002G146300 | 3207.0669 | 0.6485 | 0.3001 | 2.1607 | 0.0307168 | 0.2077574 | no |
| Sobic.002G314400 | 1171.7677 | -1.1345 | 0.5252 | -2.1600 | 0.0307719 | 0.2080628 | no |
| Sobic.005G133700 | 5393.6025 | -0.8009 | 0.3709 | -2.1595 | 0.0308149 | 0.2081594 | no |
| Sobic.009G242800 | 1176.5684 | -1.1700 | 0.5418 | -2.1594 | 0.0308186 | 0.2081594 | no |
| Sobic.009G116900 | 746.5768 | 0.8539 | 0.3954 | 2.1594 | 0.0308209 | 0.2081594 | no |
| Sobic.010G169300 | 635.9258 | 0.9409 | 0.4358 | 2.1593 | 0.0308256 | 0.2081594 | no |
| Sobic.004G006400 | 7952.4137 | -1.0161 | 0.4706 | -2.1590 | 0.0308513 | 0.2082662 | no |
| Sobic.004G353100 | 27067.6034 | -1.0122 | 0.4689 | -2.1588 | 0.0308628 | 0.2082769 | no |
| Sobic.003G106000 | 440.3918 | 0.6654 | 0.3083 | 2.1584 | 0.0309004 | 0.2083621 | no |
| Sobic.009G234900 | 40.9433 | 1.3322 | 0.6172 | 2.1583 | 0.0309051 | 0.2083621 | no |
| Sobic.001G273700 | 1213.7637 | 0.9016 | 0.4177 | 2.1581 | 0.0309173 | 0.2083621 | no |
| Sobic.002G216400 | 47.4566 | 1.2413 | 0.5752 | 2.1581 | 0.03092 | 0.2083621 | no |
| Sobic.009G092060 | 350.5832 | 1.3730 | 0.6362 | 2.1580 | 0.0309248 | 0.2083621 | no |

| Sobic.009G091800 | 2875.7536 | -1.1775 | 0.5457 | -2.1578 | 0.0309407 | 0.2084027 | no |
| --- | --- | --- | --- | --- | --- | --- | --- |
| Sobic.001G425500 | 43.9206 | 2.0331 | 0.9423 | 2.1577 | 0.0309533 | 0.2084208 | no |
| Sobic.010G167800 | 31.7577 | -2.3432 | 1.0862 | -2.1573 | 0.0309792 | 0.2085159 | no |
| Sobic.010G028200 | 451.8174 | 0.9135 | 0.4235 | 2.1572 | 0.0309884 | 0.2085159 | no |
| Sobic.002G284200 | 1192.9955 | -0.5934 | 0.2751 | -2.1571 | 0.0309971 | 0.2085159 | no |
| Sobic.003G395600 | 1883.0501 | -0.6490 | 0.3009 | -2.1567 | 0.0310256 | 0.2086413 | no |
| Sobic.002G373950 | 256.4437 | 0.9139 | 0.4238 | 2.1564 | 0.0310494 | 0.2087348 | no |
| Sobic.004G071600 | 23.8240 | 1.9555 | 0.9069 | 2.1562 | 0.0310695 | 0.2088035 | no |
| Sobic.002G235200 | 280.1653 | 0.6907 | 0.3204 | 2.1558 | 0.0311028 | 0.2089264 | no |
| Sobic.006G030300 | 235.6819 | -0.7286 | 0.3380 | -2.1557 | 0.0311076 | 0.2089264 | no |
| Sobic.002G274200 | 1143.5511 | -0.7047 | 0.3270 | -2.1552 | 0.03115 | 0.2091445 | no |
| Sobic.005G152600 | 22.1209 | -2.8813 | 1.3371 | -2.1549 | 0.0311722 | 0.2091969 | no |
| Sobic.001G237100 | 339.9823 | 0.8867 | 0.4115 | 2.1548 | 0.0311777 | 0.2091969 | no |
| Sobic.004G119100 | 725.3399 | 0.6624 | 0.3074 | 2.1545 | 0.0312012 | 0.2092769 | no |
| Sobic.001G066600 | 3802.6698 | -1.5616 | 0.7249 | -2.1544 | 0.0312094 | 0.2092769 | no |
| Sobic.001G264700 | 42.2075 | 1.6304 | 0.7571 | 2.1533 | 0.0312916 | 0.2096397 | no |
| Sobic.010G273300 | 367.6119 | 0.7071 | 0.3284 | 2.1533 | 0.0312947 | 0.2096397 | no |
| Sobic.001G161700 | 1304.1351 | 0.6917 | 0.3212 | 2.1532 | 0.0313057 | 0.2096397 | no |
| Sobic.003G345600 | 46.3201 | 1.2877 | 0.5981 | 2.1530 | 0.0313148 | 0.2096397 | no |
| Sobic.004G207900 | 1031.0054 | 0.8178 | 0.3799 | 2.1529 | 0.0313288 | 0.2096397 | no |
| Sobic.001G009000 | 33.0356 | -1.2866 | 0.5976 | -2.1528 | 0.0313311 | 0.2096397 | no |
| Sobic.001G299200 | 31.9123 | -1.5506 | 0.7203 | -2.1528 | 0.0313331 | 0.2096397 | no |
| Sobic.004G301200 | 1255.4888 | 0.5406 | 0.2511 | 2.1525 | 0.0313567 | 0.2097309 | no |
| Sobic.007G146500 | 12.0284 | 2.1709 | 1.0086 | 2.1524 | 0.0313681 | 0.2097403 | no |
| Sobic.009G122375 | 43.1280 | -1.3686 | 0.6359 | -2.1521 | 0.0313931 | 0.2098416 | no |
| Sobic.004G049800 | 11.8494 | 2.4135 | 1.1216 | 2.1518 | 0.0314106 | 0.2098625 | no |
| Sobic.002G098400 | 5.6345 | -3.6482 | 1.6956 | -2.1516 | 0.0314296 | 0.2098625 | no |
| Sobic.007G032100 | 515.5241 | -0.8597 | 0.3996 | -2.1516 | 0.03143 | 0.2098625 | no |
| Sobic.004G118800 | 575.4431 | 1.1365 | 0.5283 | 2.1514 | 0.0314478 | 0.2098625 | no |
| Sobic.001G256400 | 11.6037 | 2.2360 | 1.0393 | 2.1514 | 0.0314486 | 0.2098625 | no |
| Sobic.003G341600 | 1813.3610 | -0.7475 | 0.3475 | -2.1512 | 0.0314573 | 0.2098625 | no |
| Sobic.002G089100 | 28.2955 | 1.8900 | 0.8787 | 2.1509 | 0.0314846 | 0.2098625 | no |
| Sobic.008G104500 | 2946.0953 | 1.0522 | 0.4892 | 2.1507 | 0.031496 | 0.2098625 | no |
| Sobic.010G156800 | 27.4130 | -1.8528 | 0.8615 | -2.1506 | 0.0315039 | 0.2098625 | no |
| Sobic.008G190700 | 433.4955 | 1.1748 | 0.5463 | 2.1504 | 0.0315204 | 0.2098625 | no |
| Sobic.001G020350 | 387.6278 | 1.3041 | 0.6064 | 2.1504 | 0.0315213 | 0.2098625 | no |
| Sobic.009G099000 | 83.1117 | -1.7212 | 0.8004 | -2.1504 | 0.0315235 | 0.2098625 | no |
| Sobic.008G037700 | 155.3891 | 1.0861 | 0.5051 | 2.1504 | 0.0315257 | 0.2098625 | no |
| Sobic.008G146700 | 1454.9800 | 1.7808 | 0.8282 | 2.1501 | 0.0315436 | 0.2099159 | no |
| Sobic.002G000700 | 5833.5496 | -0.7274 | 0.3384 | -2.1497 | 0.0315753 | 0.2100605 | no |
| Sobic.002G224300 | 504.0599 | -1.3249 | 0.6165 | -2.1492 | 0.0316223 | 0.2103069 | no |
| Sobic.001G289500 | 6442.5133 | -0.9529 | 0.4435 | -2.1485 | 0.0316767 | 0.2106022 | no |
| Sobic.005G045000 | 4141.8610 | -0.9832 | 0.4576 | -2.1483 | 0.0316894 | 0.2106199 | no |
| Sobic.008G116100 | 37.3263 | -1.8395 | 0.8564 | -2.1479 | 0.0317193 | 0.2107522 | no |
| Sobic.005G113533 | 51.4194 | -0.8854 | 0.4123 | -2.1474 | 0.0317588 | 0.2108286 | no |
| Sobic.008G172300 | 966.4427 | -0.5979 | 0.2784 | -2.1474 | 0.0317606 | 0.2108286 | no |
| Sobic.001G454900 | 1054.7773 | 1.1526 | 0.5367 | 2.1474 | 0.0317641 | 0.2108286 | no |
| Sobic.003G404400 | 1928.3469 | -1.3386 | 0.6234 | -2.1472 | 0.0317746 | 0.2108286 | no |
| Sobic.009G069700 | 12.7771 | -1.8966 | 0.8834 | -2.1471 | 0.0317864 | 0.2108286 | no |
| Sobic.002G414800 | 108.6091 | 1.2058 | 0.5616 | 2.1470 | 0.0317907 | 0.2108286 | no |
| Sobic.005G208900 | 790.6838 | 0.8936 | 0.4162 | 2.1469 | 0.0318017 | 0.2108347 | no |
| Sobic.009G054900 | 5619.4428 | -1.2778 | 0.5953 | -2.1467 | 0.0318175 | 0.2108733 | no |
| Sobic.001G505600 | 163.4421 | 0.8729 | 0.4067 | 2.1465 | 0.031836 | 0.2109301 | no |
| Sobic.010G246500 | 43.8493 | -2.1493 | 1.0015 | -2.1462 | 0.0318607 | 0.211027 | no |
| Sobic.003G345100 | 23.2959 | 2.3702 | 1.1044 | 2.1460 | 0.0318715 | 0.2110322 | no |
| Sobic.001G477700 | 688.5210 | 0.5560 | 0.2591 | 2.1457 | 0.0318946 | 0.2111187 | no |
| Sobic.005G003300 | 6919.3683 | 0.5743 | 0.2677 | 2.1456 | 0.0319079 | 0.2111405 | no |

| Sobic.003G104500 | 260.9207 | -0.7400 | 0.3450 | -2.1449 | 0.0319609 | 0.2113688 | no |
| --- | --- | --- | --- | --- | --- | --- | --- |
| Sobic.008G124766 | 21.6955 | -1.3692 | 0.6384 | -2.1449 | 0.0319624 | 0.2113688 | no |
| Sobic.008G134300 | 346.4738 | -1.1033 | 0.5144 | -2.1447 | 0.0319766 | 0.2113965 | no |
| Sobic.003G036500 | 875.9286 | 0.6369 | 0.2970 | 2.1444 | 0.0320046 | 0.2114611 | no |
| Sobic.002G424700 | 708.6917 | -0.6942 | 0.3237 | -2.1443 | 0.0320064 | 0.2114611 | no |
| Sobic.003G330100 | 1679.1546 | -1.3303 | 0.6205 | -2.1440 | 0.0320353 | 0.2115857 | no |
| Sobic.003G346400 | 935.1772 | 0.5315 | 0.2480 | 2.1432 | 0.032101 | 0.2119529 | no |
| Sobic.001G433600 | 46.2425 | 1.0875 | 0.5075 | 2.1429 | 0.0321249 | 0.2120446 | no |
| Sobic.002G225200 | 15.9242 | -1.9025 | 0.8880 | -2.1425 | 0.0321571 | 0.2121905 | no |
| Sobic.006G131000 | 2176.5621 | 0.5956 | 0.2780 | 2.1420 | 0.0321943 | 0.2123699 | no |
| Sobic.004G085700 | 24.6863 | -1.6412 | 0.7663 | -2.1416 | 0.0322288 | 0.212531 | no |
| Sobic.004G143475 | 1136.7902 | 0.9207 | 0.4300 | 2.1412 | 0.0322578 | 0.2126551 | no |
| Sobic.009G114500 | 303.4938 | 1.5996 | 0.7472 | 2.1408 | 0.0322907 | 0.2128059 | no |
| Sobic.009G052600 | 3636.4983 | -0.6995 | 0.3268 | -2.1405 | 0.0323158 | 0.2128565 | no |
| Sobic.010G230600 | 1467.2615 | 0.6757 | 0.3157 | 2.1404 | 0.0323236 | 0.2128565 | no |
| Sobic.003G079300 | 9.3363 | 3.3811 | 1.5797 | 2.1403 | 0.0323287 | 0.2128565 | no |
| Sobic.006G219300 | 3573.3521 | 0.7762 | 0.3628 | 2.1397 | 0.0323779 | 0.2130674 | no |
| Sobic.004G133201 | 21.5065 | 1.7032 | 0.7960 | 2.1397 | 0.0323809 | 0.2130674 | no |
| Sobic.002G195300 | 34.2648 | 1.4719 | 0.6881 | 2.1392 | 0.0324196 | 0.2132153 | no |
| Sobic.004G007900 | 428.1782 | 0.7431 | 0.3474 | 2.1391 | 0.0324236 | 0.2132153 | no |
| Sobic.003G177800 | 188.3777 | 1.1074 | 0.5177 | 2.1389 | 0.0324435 | 0.2132796 | no |
| Sobic.003G276300 | 957.0166 | -0.7393 | 0.3457 | -2.1387 | 0.0324634 | 0.2133442 | no |
| Sobic.001G522100 | 400.3455 | 0.8731 | 0.4084 | 2.1378 | 0.0325305 | 0.2136327 | no |
| Sobic.005G078600 | 795.8283 | -0.9221 | 0.4313 | -2.1378 | 0.0325332 | 0.2136327 | no |
| Sobic.001G379700 | 2677.2110 | -0.9496 | 0.4442 | -2.1376 | 0.0325465 | 0.2136327 | no |
| Sobic.004G040200 | 4960.0185 | -0.5819 | 0.2722 | -2.1376 | 0.0325479 | 0.2136327 | no |
| Sobic.010G088300 | 1048.4822 | 0.6252 | 0.2925 | 2.1374 | 0.0325644 | 0.213675 | no |
| Sobic.009G145900 | 8542.4381 | -0.6345 | 0.2969 | -2.1372 | 0.0325829 | 0.2137297 | no |
| Sobic.004G224700 | 159.9684 | 1.2244 | 0.5731 | 2.1364 | 0.0326439 | 0.2140634 | no |
| Sobic.004G095800 | 37.9781 | -1.8418 | 0.8623 | -2.1358 | 0.0326922 | 0.2141518 | no |
| Sobic.001G027600 | 4820.4211 | -1.0904 | 0.5105 | -2.1358 | 0.0326924 | 0.2141518 | no |
| Sobic.004G035601 | 120.8230 | -1.6394 | 0.7676 | -2.1356 | 0.0327139 | 0.2141518 | no |
| Sobic.K044403 | 5.5002 | 5.6737 | 2.6568 | 2.1355 | 0.0327162 | 0.2141518 | no |
| Sobic.002G057400 | 74.1372 | -0.8316 | 0.3894 | -2.1355 | 0.0327204 | 0.2141518 | no |
| Sobic.010G134600 | 19.7673 | 2.1561 | 1.0097 | 2.1354 | 0.032731 | 0.2141518 | no |
| Sobic.003G137200 | 228.6997 | -1.5454 | 0.7238 | -2.1352 | 0.0327422 | 0.2141518 | no |
| Sobic.003G409900 | 268.3383 | 0.7659 | 0.3587 | 2.1352 | 0.0327477 | 0.2141518 | no |
| Sobic.001G290400 | 516.7585 | 1.1155 | 0.5224 | 2.1351 | 0.0327488 | 0.2141518 | no |
| Sobic.009G243500 | 5.6817 | -3.6528 | 1.7110 | -2.1348 | 0.0327752 | 0.2141825 | no |
| Sobic.009G140600 | 2723.2201 | -0.8571 | 0.4015 | -2.1348 | 0.0327767 | 0.2141825 | no |
| Sobic.004G154400 | 18607.6714 | -0.6636 | 0.3109 | -2.1346 | 0.0327937 | 0.2141825 | no |
| Sobic.004G357700 | 772.8944 | 0.8204 | 0.3843 | 2.1346 | 0.0327941 | 0.2141825 | no |
| Sobic.003G372800 | 2412.0634 | 0.8546 | 0.4005 | 2.1341 | 0.0328345 | 0.2143796 | no |
| Sobic.004G015500 | 220.7951 | 0.9470 | 0.4438 | 2.1340 | 0.0328462 | 0.2143899 | no |
| Sobic.006G017600 | 145.6053 | 1.0852 | 0.5086 | 2.1337 | 0.0328641 | 0.21444 | no |
| Sobic.003G426700 | 324.4922 | -0.6404 | 0.3002 | -2.1331 | 0.0329203 | 0.2147404 | no |
| Sobic.002G302900 | 356.8849 | 0.9406 | 0.4410 | 2.1328 | 0.0329442 | 0.21483 | no |
| Sobic.008G157900 | 18.5929 | -2.4022 | 1.1269 | -2.1316 | 0.0330366 | 0.2152969 | no |
| Sobic.001G090200 | 2191.1715 | -0.5539 | 0.2598 | -2.1316 | 0.0330382 | 0.2152969 | no |
| Sobic.006G037300 | 5077.5967 | -0.7029 | 0.3298 | -2.1315 | 0.0330464 | 0.2152969 | no |
| Sobic.010G042700 | 225.4024 | 0.7627 | 0.3579 | 2.1312 | 0.0330745 | 0.2153926 | no |
| Sobic.001G068800 | 12.4443 | -1.7932 | 0.8414 | -2.1311 | 0.0330815 | 0.2153926 | no |
| Sobic.003G412300 | 2426.4859 | 0.6392 | 0.3000 | 2.1307 | 0.0331146 | 0.2155411 | no |
| Sobic.010G091300 | 325.7560 | 1.0169 | 0.4774 | 2.1302 | 0.0331524 | 0.2157208 | no |
| Sobic.008G125100 | 158.8542 | 0.7894 | 0.3708 | 2.1292 | 0.0332406 | 0.2162279 | no |
| Sobic.002G258600 | 3516.8912 | -0.8116 | 0.3813 | -2.1287 | 0.0332773 | 0.2163067 | no |
| Sobic.003G219700 | 912.6773 | 0.8865 | 0.4165 | 2.1286 | 0.0332889 | 0.2163067 | no |

| Sobic.006G083300 | 424.4304 | 1.0397 | 0.4885 | 2.1286 | 0.0332906 | 0.2163067 | no |
| --- | --- | --- | --- | --- | --- | --- | --- |
| Sobic.010G266000 | 5169.5297 | -0.7965 | 0.3742 | -2.1285 | 0.0332937 | 0.2163067 | no |
| Sobic.001G081100 | 15.2030 | 1.6277 | 0.7649 | 2.1281 | 0.0333311 | 0.2164828 | no |
| Sobic.006G048100 | 5.8875 | -3.7210 | 1.7488 | -2.1278 | 0.0333538 | 0.216512 | no |
| Sobic.007G169300 | 1838.1925 | -0.4669 | 0.2194 | -2.1276 | 0.0333673 | 0.216512 | no |
| Sobic.007G196201 | 521.1895 | -0.8861 | 0.4165 | -2.1276 | 0.0333701 | 0.216512 | no |
| Sobic.007G217800 | 19.9365 | -1.4949 | 0.7026 | -2.1275 | 0.0333767 | 0.216512 | no |
| Sobic.003G312550 | 209.6488 | -1.8170 | 0.8542 | -2.1271 | 0.0334089 | 0.2166545 | no |
| Sobic.003G029500 | 487.5319 | 0.8313 | 0.3909 | 2.1268 | 0.0334341 | 0.216751 | no |
| Sobic.004G217700 | 1445.1051 | -1.0366 | 0.4875 | -2.1265 | 0.0334632 | 0.2168303 | no |
| Sobic.003G123300 | 2024.7365 | -1.6620 | 0.7816 | -2.1264 | 0.0334669 | 0.2168303 | no |
| Sobic.002G338700 | 95.9347 | -1.3634 | 0.6413 | -2.1260 | 0.0335022 | 0.2169558 | no |
| Sobic.009G070400 | 5913.2333 | -1.3773 | 0.6479 | -2.1260 | 0.0335068 | 0.2169558 | no |
| Sobic.002G258500 | 22.1218 | 3.7234 | 1.7515 | 2.1258 | 0.0335196 | 0.2169722 | no |
| Sobic.003G404100 | 1060.9822 | 0.8112 | 0.3817 | 2.1252 | 0.0335729 | 0.2172502 | no |
| Sobic.001G151300 | 13.6802 | 2.1483 | 1.0110 | 2.1249 | 0.0335945 | 0.2173044 | no |
| Sobic.003G329400 | 2508.9551 | 0.7238 | 0.3407 | 2.1248 | 0.0336019 | 0.2173044 | no |
| Sobic.009G171600 | 81.8262 | 1.3242 | 0.6233 | 2.1247 | 0.0336148 | 0.2173214 | no |
| Sobic.004G315800 | 3605.6677 | -0.8748 | 0.4118 | -2.1243 | 0.033648 | 0.2174694 | no |
| Sobic.005G061033 | 21.4484 | 1.5759 | 0.7420 | 2.1239 | 0.0336805 | 0.2176131 | no |
| Sobic.007G217000 | 2609.1977 | 0.4752 | 0.2238 | 2.1237 | 0.0336957 | 0.217644 | no |
| Sobic.003G044100 | 524.6645 | -0.6455 | 0.3040 | -2.1235 | 0.0337087 | 0.2176618 | no |
| Sobic.006G161800 | 60.3682 | -2.5064 | 1.1804 | -2.1233 | 0.0337303 | 0.2177341 | no |
| Sobic.010G022100 | 5846.4598 | -1.4574 | 0.6865 | -2.1230 | 0.0337574 | 0.2177418 | no |
| Sobic.002G141700 | 552.6115 | 0.8517 | 0.4012 | 2.1229 | 0.0337586 | 0.2177418 | no |
| Sobic.001G335100 | 386.2214 | 0.6761 | 0.3185 | 2.1229 | 0.0337638 | 0.2177418 | no |
| Sobic.003G252900 | 181.3478 | 1.2661 | 0.5964 | 2.1228 | 0.0337727 | 0.2177418 | no |
| Sobic.006G005600 | 431.1834 | 2.1818 | 1.0282 | 2.1220 | 0.0338385 | 0.2180989 | no |
| Sobic.002G398200 | 172.4632 | 0.8572 | 0.4040 | 2.1218 | 0.0338542 | 0.2181337 | no |
| Sobic.002G429300 | 431.9587 | 0.6139 | 0.2894 | 2.1216 | 0.033871 | 0.2181752 | no |
| Sobic.001G486900 | 123.6375 | 0.8509 | 0.4011 | 2.1213 | 0.0338996 | 0.218293 | no |
| Sobic.010G216000 | 1086.8961 | 0.6392 | 0.3014 | 2.1207 | 0.0339462 | 0.2184167 | no |
| Sobic.006G257900 | 42574.2592 | -0.6710 | 0.3164 | -2.1206 | 0.0339534 | 0.2184167 | no |
| Sobic.008G082800 | 20.7871 | 1.6127 | 0.7605 | 2.1206 | 0.0339585 | 0.2184167 | no |
| Sobic.003G190800 | 3377.0316 | 0.4674 | 0.2204 | 2.1205 | 0.0339603 | 0.2184167 | no |
| Sobic.007G126300 | 7.9869 | -2.2073 | 1.0411 | -2.1202 | 0.0339914 | 0.21855 | no |
| Sobic.006G082500 | 1155.4046 | -0.6668 | 0.3146 | -2.1194 | 0.0340525 | 0.2186812 | no |
| Sobic.001G161400 | 763.0350 | -1.0289 | 0.4855 | -2.1193 | 0.0340644 | 0.2186812 | no |
| Sobic.010G052500 | 1042.4109 | -1.0872 | 0.5130 | -2.1192 | 0.0340724 | 0.2186812 | no |
| Sobic.001G289100 | 570.5520 | -0.7113 | 0.3357 | -2.1191 | 0.034078 | 0.2186812 | no |
| Sobic.002G069050 | 328.8684 | -0.8220 | 0.3879 | -2.1191 | 0.0340797 | 0.2186812 | no |
| Sobic.007G020300 | 612.6129 | -1.1987 | 0.5657 | -2.1191 | 0.0340799 | 0.2186812 | no |
| Sobic.005G154400 | 319.1670 | -0.7978 | 0.3765 | -2.1191 | 0.0340844 | 0.2186812 | no |
| Sobic.001G041800 | 2748.5358 | -1.0127 | 0.4779 | -2.1189 | 0.0340966 | 0.2186933 | no |
| Sobic.001G173300 | 14766.6163 | 0.9082 | 0.4287 | 2.1186 | 0.0341267 | 0.2188195 | no |
| Sobic.003G413700 | 147.0659 | 0.8955 | 0.4228 | 2.1183 | 0.034148 | 0.2188898 | no |
| Sobic.007G163700 | 1446.9019 | -0.5136 | 0.2426 | -2.1172 | 0.0342427 | 0.2194064 | no |
| Sobic.002G306400 | 1516.9612 | -0.5115 | 0.2416 | -2.1170 | 0.0342563 | 0.2194064 | no |
| Sobic.001G125400 | 106.5701 | 1.1863 | 0.5603 | 2.1170 | 0.0342598 | 0.2194064 | no |
| Sobic.006G083000 | 61.7749 | 1.5373 | 0.7263 | 2.1166 | 0.0342947 | 0.2194175 | no |
| Sobic.002G119900 | 31.4969 | -2.1274 | 1.0051 | -2.1166 | 0.0342963 | 0.2194175 | no |
| Sobic.005G128800 | 1068.8249 | -0.8213 | 0.3880 | -2.1165 | 0.0343023 | 0.2194175 | no |
| Sobic.001G529400 | 314.9885 | -1.3656 | 0.6452 | -2.1165 | 0.0343032 | 0.2194175 | no |
| Sobic.005G110442 | 365.2729 | -0.7969 | 0.3767 | -2.1156 | 0.0343758 | 0.2198156 | no |
| Sobic.001G454100 | 4885.0793 | -0.8596 | 0.4064 | -2.1152 | 0.0344108 | 0.2199694 | no |
| Sobic.003G125100 | 179.4318 | 0.9329 | 0.4411 | 2.1151 | 0.034425 | 0.2199694 | no |
| Sobic.006G224300 | 155.7096 | 0.6296 | 0.2977 | 2.1148 | 0.0344458 | 0.2199694 | no |

| Sobic.008G189600 | 192.9522 | -1.1641 | 0.5504 | -2.1148 | 0.0344475 | 0.2199694 | no |
| --- | --- | --- | --- | --- | --- | --- | --- |
| Sobic.009G226800 | 142.9909 | 0.9757 | 0.4614 | 2.1147 | 0.034452 | 0.2199694 | no |
| Sobic.006G048775 | 3548.6352 | -1.0330 | 0.4887 | -2.1138 | 0.0345364 | 0.2204415 | no |
| Sobic.004G058300 | 13.4055 | 2.7204 | 1.2871 | 2.1136 | 0.0345493 | 0.2204568 | no |
| Sobic.004G035300 | 105.2346 | 1.5818 | 0.7484 | 2.1134 | 0.0345626 | 0.2204753 | no |
| Sobic.006G270000 | 383.7082 | -0.8128 | 0.3847 | -2.1127 | 0.0346243 | 0.2208018 | no |
| Sobic.004G243800 | 25.3442 | 1.9411 | 0.9190 | 2.1122 | 0.0346686 | 0.2209991 | no |
| Sobic.006G218300 | 11.4704 | 2.6674 | 1.2630 | 2.1120 | 0.0346858 | 0.2209991 | no |
| Sobic.003G256000 | 2550.3898 | 0.9443 | 0.4471 | 2.1120 | 0.0346867 | 0.2209991 | no |
| Sobic.004G346200 | 8.9987 | 2.1236 | 1.0057 | 2.1115 | 0.0347264 | 0.2211857 | no |
| Sobic.005G137000 | 46.8161 | 2.0038 | 0.9492 | 2.1112 | 0.0347584 | 0.2213223 | no |
| Sobic.004G252900 | 3186.2898 | 0.6695 | 0.3171 | 2.1110 | 0.0347765 | 0.2213592 | no |
| Sobic.010G113900 | 34.0834 | 1.2496 | 0.5920 | 2.1108 | 0.034791 | 0.2213592 | no |
| Sobic.010G150000 | 423.7832 | -0.7613 | 0.3607 | -2.1107 | 0.0347957 | 0.2213592 | no |
| Sobic.002G248100 | 28.0030 | 1.4863 | 0.7044 | 2.1101 | 0.0348516 | 0.2214397 | no |
| Sobic.001G470400 | 108.3235 | -0.9717 | 0.4605 | -2.1100 | 0.0348546 | 0.2214397 | no |
| Sobic.007G178500 | 4964.9694 | -0.7573 | 0.3589 | -2.1100 | 0.0348559 | 0.2214397 | no |
| Sobic.003G358150 | 69.5151 | 1.5728 | 0.7454 | 2.1100 | 0.0348595 | 0.2214397 | no |
| Sobic.007G153500 | 1773.2456 | 0.5340 | 0.2531 | 2.1099 | 0.0348665 | 0.2214397 | no |
| Sobic.001G368600 | 2078.0641 | -1.1309 | 0.5360 | -2.1098 | 0.0348765 | 0.2214397 | no |
| Sobic.009G068400 | 47.7986 | 1.0556 | 0.5003 | 2.1097 | 0.0348839 | 0.2214397 | no |
| Sobic.003G094100 | 2993.5389 | -0.9373 | 0.4443 | -2.1096 | 0.0348942 | 0.2214397 | no |
| Sobic.001G048100 | 1641.6278 | -0.4799 | 0.2275 | -2.1095 | 0.0349028 | 0.2214397 | no |
| Sobic.006G235800 | 4681.5176 | 0.7926 | 0.3757 | 2.1094 | 0.0349136 | 0.2214416 | no |
| Sobic.002G162000 | 51.9097 | 1.2436 | 0.5896 | 2.1091 | 0.0349343 | 0.221506 | no |
| Sobic.010G208900 | 272.0786 | -1.0957 | 0.5196 | -2.1088 | 0.0349653 | 0.2216359 | no |
| Sobic.009G239600 | 104.1354 | 0.7821 | 0.3710 | 2.1082 | 0.0350176 | 0.2219007 | no |
| Sobic.003G316200 | 1531.1186 | 0.5189 | 0.2462 | 2.1079 | 0.0350372 | 0.2219586 | no |
| Sobic.008G117000 | 64.9232 | -1.2269 | 0.5821 | -2.1077 | 0.0350611 | 0.222043 | no |
| Sobic.001G252401 | 2557.5861 | -0.8294 | 0.3935 | -2.1075 | 0.0350737 | 0.2220562 | no |
| Sobic.002G076866 | 909.8133 | 1.1110 | 0.5272 | 2.1074 | 0.0350859 | 0.2220667 | no |
| Sobic.003G338400 | 770.8637 | 0.7019 | 0.3331 | 2.1072 | 0.0350964 | 0.2220667 | no |
| Sobic.003G214400 | 661.4228 | 0.7092 | 0.3367 | 2.1065 | 0.0351606 | 0.2223967 | no |
| Sobic.003G010800 | 7155.2173 | -0.7384 | 0.3505 | -2.1064 | 0.0351697 | 0.2223967 | no |
| Sobic.002G061900 | 1812.9748 | -1.4023 | 0.6658 | -2.1062 | 0.0351865 | 0.2224364 | no |
| Sobic.004G350300 | 4810.6048 | 0.4938 | 0.2346 | 2.1046 | 0.035323 | 0.2232322 | no |
| Sobic.003G184765 | 354.1358 | 1.1782 | 0.5598 | 2.1045 | 0.035337 | 0.2232407 | no |
| Sobic.004G018500 | 843.3280 | -0.6067 | 0.2883 | -2.1044 | 0.0353455 | 0.2232407 | no |
| Sobic.008G176000 | 574.0264 | 0.6897 | 0.3278 | 2.1039 | 0.0353891 | 0.2234493 | no |
| Sobic.010G232700 | 37.4975 | 1.7218 | 0.8185 | 2.1036 | 0.0354108 | 0.2235195 | no |
| Sobic.006G078400 | 4378.0635 | 0.9152 | 0.4352 | 2.1030 | 0.0354678 | 0.223812 | no |
| Sobic.009G175600 | 376.8208 | 1.2712 | 0.6045 | 2.1028 | 0.0354849 | 0.2238533 | no |
| Sobic.006G239800 | 2136.0446 | 0.8694 | 0.4135 | 2.1026 | 0.0355051 | 0.2239135 | no |
| Sobic.002G294100 | 492.5253 | 0.7809 | 0.3715 | 2.1019 | 0.0355577 | 0.2241395 | no |
| Sobic.010G258100 | 14073.7295 | -0.7361 | 0.3502 | -2.1019 | 0.0355622 | 0.2241395 | no |
| Sobic.003G258400 | 12916.4001 | -0.6967 | 0.3315 | -2.1017 | 0.03558 | 0.2241475 | no |
| Sobic.008G042100 | 7.7574 | -2.8068 | 1.3355 | -2.1016 | 0.0355847 | 0.2241475 | no |
| Sobic.008G105300 | 914.5827 | -1.0691 | 0.5087 | -2.1014 | 0.0356069 | 0.2242186 | no |
| Sobic.002G277100 | 12177.6716 | -1.0280 | 0.4892 | -2.1013 | 0.0356172 | 0.2242186 | no |
| Sobic.003G150200 | 806.4686 | -0.6848 | 0.3260 | -2.1007 | 0.0356641 | 0.2244468 | no |
| Sobic.002G085200 | 15.5016 | 3.2086 | 1.5274 | 2.1006 | 0.0356763 | 0.2244564 | no |
| Sobic.007G105900 | 880.9778 | -0.5125 | 0.2441 | -2.1001 | 0.0357239 | 0.2246497 | no |
| Sobic.005G215900 | 230.4419 | 1.9024 | 0.9059 | 2.0999 | 0.0357352 | 0.2246497 | no |
| Sobic.004G269700 | 283.0228 | 0.8304 | 0.3954 | 2.0999 | 0.035739 | 0.2246497 | no |
| Sobic.010G233800 | 2733.0966 | -1.0339 | 0.4924 | -2.0997 | 0.035755 | 0.2246833 | no |
| Sobic.009G166650 | 43.2954 | 1.0446 | 0.4977 | 2.0988 | 0.0358336 | 0.2250937 | no |
| Sobic.003G346500 | 535.9830 | 0.6030 | 0.2873 | 2.0987 | 0.0358417 | 0.2250937 | no |

| Sobic.003G372900 | 1729.6348 | -0.9190 | 0.4380 | -2.0983 | 0.0358825 | 0.225283 | no |
| --- | --- | --- | --- | --- | --- | --- | --- |
| Sobic.002G007800 | 26.4826 | 1.7069 | 0.8137 | 2.0978 | 0.0359231 | 0.2254669 | no |
| Sobic.001G009200 | 2107.8258 | -0.9484 | 0.4521 | -2.0977 | 0.0359332 | 0.2254669 | no |
| Sobic.003G143800 | 1107.3755 | 0.6531 | 0.3114 | 2.0972 | 0.0359734 | 0.2256521 | no |
| Sobic.004G228200 | 3502.7278 | -0.8281 | 0.3949 | -2.0971 | 0.035986 | 0.2256645 | no |
| Sobic.005G178100 | 697.6044 | 0.8785 | 0.4191 | 2.0963 | 0.0360513 | 0.2260065 | no |
| Sobic.005G128550 | 6.5459 | -2.7343 | 1.3049 | -2.0955 | 0.0361281 | 0.2264091 | no |
| Sobic.010G128500 | 151.8636 | 0.6359 | 0.3035 | 2.0954 | 0.036137 | 0.2264091 | no |
| Sobic.003G041200 | 267.5547 | -0.7755 | 0.3702 | -2.0945 | 0.0362166 | 0.2267721 | no |
| Sobic.002G132600 | 312.5264 | 1.0477 | 0.5002 | 2.0944 | 0.0362237 | 0.2267721 | no |
| Sobic.004G204600 | 11.9248 | 2.2870 | 1.0920 | 2.0944 | 0.0362272 | 0.2267721 | no |
| Sobic.002G251800 | 41.1788 | 1.4539 | 0.6943 | 2.0942 | 0.0362421 | 0.226798 | no |
| Sobic.002G335100 | 410.2410 | -3.6032 | 1.7214 | -2.0932 | 0.036329 | 0.2272581 | no |
| Sobic.002G038600 | 6316.0293 | -0.6167 | 0.2946 | -2.0931 | 0.0363396 | 0.2272581 | no |
| Sobic.001G156200 | 150.1796 | -1.0238 | 0.4892 | -2.0930 | 0.0363479 | 0.2272581 | no |
| Sobic.004G162200 | 524.2722 | 1.5477 | 0.7396 | 2.0928 | 0.0363712 | 0.2273363 | no |
| Sobic.004G323700 | 2920.5294 | 0.5342 | 0.2553 | 2.0925 | 0.0363963 | 0.2273578 | no |
| Sobic.001G333000 | 268.8511 | -1.4183 | 0.6778 | -2.0924 | 0.0364048 | 0.2273578 | no |
| Sobic.001G533100 | 207.2618 | 0.6306 | 0.3014 | 2.0924 | 0.036407 | 0.2273578 | no |
| Sobic.001G347100 | 11.0312 | 1.9910 | 0.9516 | 2.0922 | 0.0364189 | 0.2273648 | no |
| Sobic.008G152100 | 474.7118 | -0.8148 | 0.3895 | -2.0917 | 0.0364617 | 0.2275644 | no |
| Sobic.004G198800 | 116.3735 | 1.7051 | 0.8152 | 2.0916 | 0.0364756 | 0.2275837 | no |
| Sobic.001G139700 | 7.5196 | 2.4540 | 1.1735 | 2.0913 | 0.0365029 | 0.2276749 | no |
| Sobic.001G182850 | 107.9054 | -0.8603 | 0.4114 | -2.0912 | 0.0365118 | 0.2276749 | no |
| Sobic.008G016300 | 97.4671 | 1.0739 | 0.5138 | 2.0904 | 0.0365843 | 0.2280154 | no |
| Sobic.009G123900 | 49408.3093 | -0.9234 | 0.4418 | -2.0903 | 0.036588 | 0.2280154 | no |
| Sobic.010G091366 | 6.0128 | 4.7890 | 2.2913 | 2.0901 | 0.0366097 | 0.2280835 | no |
| Sobic.003G313800 | 9.7950 | 2.3312 | 1.1160 | 2.0889 | 0.0367201 | 0.2287035 | no |
| Sobic.001G450800 | 1229.2519 | 0.7128 | 0.3413 | 2.0886 | 0.0367407 | 0.2287408 | no |
| Sobic.003G204200 | 43.9233 | 1.2386 | 0.5930 | 2.0886 | 0.0367478 | 0.2287408 | no |
| Sobic.007G125900 | 482.7216 | 1.2400 | 0.5939 | 2.0879 | 0.036806 | 0.2289768 | no |
| Sobic.003G077400 | 1163.9120 | -0.8342 | 0.3996 | -2.0878 | 0.0368199 | 0.2289768 | no |
| Sobic.008G020800 | 22.2615 | -1.6356 | 0.7835 | -2.0876 | 0.0368301 | 0.2289768 | no |
| Sobic.007G047400 | 36133.3223 | -1.3945 | 0.6680 | -2.0876 | 0.0368313 | 0.2289768 | no |
| Sobic.010G067700 | 3887.9883 | -0.6874 | 0.3293 | -2.0875 | 0.03684 | 0.2289768 | no |
| Sobic.006G075801 | 343.1884 | 0.7985 | 0.3826 | 2.0873 | 0.0368582 | 0.2290223 | no |
| Sobic.005G096700 | 175.0412 | 0.8080 | 0.3872 | 2.0869 | 0.0368964 | 0.2291781 | no |
| Sobic.003G264901 | 323.7028 | -0.6779 | 0.3249 | -2.0868 | 0.0369082 | 0.2291781 | no |
| Sobic.006G190500 | 153.5989 | -1.1434 | 0.5480 | -2.0866 | 0.0369202 | 0.2291781 | no |
| Sobic.002G412300 | 14.4414 | 2.1890 | 1.0491 | 2.0866 | 0.0369267 | 0.2291781 | no |
| Sobic.006G207000 | 18.6210 | -2.0493 | 0.9825 | -2.0858 | 0.0369961 | 0.2295412 | no |
| Sobic.005G182300 | 8.0784 | 2.0738 | 0.9944 | 2.0855 | 0.0370251 | 0.2296536 | no |
| Sobic.005G094500 | 1955.1302 | -0.8236 | 0.3950 | -2.0853 | 0.0370433 | 0.2296989 | no |
| Sobic.005G182400 | 3932.8315 | -0.7385 | 0.3542 | -2.0851 | 0.0370636 | 0.2297072 | no |
| Sobic.003G389700 | 716.7689 | -1.1263 | 0.5402 | -2.0850 | 0.0370721 | 0.2297072 | no |
| Sobic.009G183000 | 799.2964 | 1.4243 | 0.6832 | 2.0849 | 0.0370773 | 0.2297072 | no |
| Sobic.001G336500 | 140.2194 | -1.8060 | 0.8663 | -2.0847 | 0.0370984 | 0.2297623 | no |
| Sobic.004G261300 | 29.9524 | 1.5135 | 0.7261 | 2.0845 | 0.0371127 | 0.2297623 | no |
| Sobic.009G161500 | 24.6088 | -2.1443 | 1.0287 | -2.0845 | 0.0371189 | 0.2297623 | no |
| Sobic.001G175800 | 1506.7800 | -0.6521 | 0.3129 | -2.0842 | 0.0371441 | 0.2298209 | no |
| Sobic.001G501500 | 9.6034 | -2.5030 | 1.2010 | -2.0841 | 0.0371501 | 0.2298209 | no |
| Sobic.004G067900 | 13.4902 | 2.2044 | 1.0581 | 2.0833 | 0.0372257 | 0.2302206 | no |
| Sobic.004G171700 | 55.6973 | -1.2078 | 0.5799 | -2.0827 | 0.0372749 | 0.2304423 | no |
| Sobic.007G180600 | 1404.1816 | 0.6949 | 0.3337 | 2.0826 | 0.0372834 | 0.2304423 | no |
| Sobic.008G104801 | 94.2476 | -1.0174 | 0.4887 | -2.0819 | 0.0373502 | 0.2307879 | no |
| Sobic.010G042600 | 51.9568 | 1.6532 | 0.7942 | 2.0817 | 0.037371 | 0.2308484 | no |
| Sobic.003G008100 | 1220.7180 | -0.5094 | 0.2447 | -2.0815 | 0.0373843 | 0.2308488 | no |

| Sobic.006G210400 | 1299.1404 | -0.9119 | 0.4381 | -2.0814 | 0.0373929 | 0.2308488 | no |
| --- | --- | --- | --- | --- | --- | --- | --- |
| Sobic.007G022600 | 1292.6350 | -0.7370 | 0.3541 | -2.0812 | 0.0374117 | 0.2308713 | no |
| Sobic.006G021900 | 186.1601 | 1.0186 | 0.4895 | 2.0811 | 0.0374292 | 0.2308713 | no |
| Sobic.003G316400 | 352.6299 | 0.6109 | 0.2936 | 2.0810 | 0.0374367 | 0.2308713 | no |
| Sobic.001G361300 | 1250.0891 | 0.7242 | 0.3480 | 2.0809 | 0.0374404 | 0.2308713 | no |
| Sobic.005G093300 | 2158.7541 | -1.0769 | 0.5176 | -2.0808 | 0.0374521 | 0.2308762 | no |
| Sobic.006G187401 | 818.5127 | -0.6145 | 0.2954 | -2.0806 | 0.0374688 | 0.2309118 | no |
| Sobic.001G054800 | 6210.1338 | 1.3880 | 0.6673 | 2.0801 | 0.0375194 | 0.230937 | no |
| Sobic.006G166000 | 1163.1915 | -1.0562 | 0.5078 | -2.0801 | 0.0375208 | 0.230937 | no |
| Sobic.005G064200 | 178.5337 | 1.0024 | 0.4819 | 2.0800 | 0.0375215 | 0.230937 | no |
| Sobic.001G331301 | 12.4229 | 3.7311 | 1.7938 | 2.0800 | 0.0375265 | 0.230937 | no |
| Sobic.007G017200 | 88.7447 | 1.1188 | 0.5379 | 2.0800 | 0.0375277 | 0.230937 | no |
| Sobic.003G421400 | 4052.1945 | -0.7505 | 0.3609 | -2.0795 | 0.0375707 | 0.2311342 | no |
| Sobic.009G037700 | 107.0642 | 1.0287 | 0.4948 | 2.0791 | 0.0376075 | 0.2312935 | no |
| Sobic.004G206200 | 670.3000 | -0.8596 | 0.4135 | -2.0790 | 0.0376199 | 0.2313018 | no |
| Sobic.002G379500 | 3782.6226 | -0.6457 | 0.3106 | -2.0787 | 0.0376468 | 0.2313999 | no |
| Sobic.009G225700 | 31.7795 | 1.9990 | 0.9619 | 2.0781 | 0.0376981 | 0.2316477 | no |
| Sobic.010G088950 | 19.4754 | 3.5393 | 1.7038 | 2.0773 | 0.0377774 | 0.2320675 | no |
| Sobic.010G276400 | 8965.5410 | -1.3737 | 0.6613 | -2.0771 | 0.0377884 | 0.2320675 | no |
| Sobic.001G222000 | 230.9601 | 1.0159 | 0.4891 | 2.0770 | 0.0378008 | 0.2320761 | no |
| Sobic.009G217100 | 5203.6468 | -0.4766 | 0.2295 | -2.0765 | 0.0378453 | 0.2322814 | no |
| Sobic.001G062300 | 11799.2390 | 1.0313 | 0.4969 | 2.0755 | 0.0379415 | 0.2328042 | no |
| Sobic.006G078100 | 113.6489 | 0.9785 | 0.4716 | 2.0749 | 0.037998 | 0.2330832 | no |
| Sobic.009G100800 | 11635.5722 | -1.5583 | 0.7511 | -2.0746 | 0.038024 | 0.2331747 | no |
| Sobic.002G217300 | 191.9812 | 2.0108 | 0.9694 | 2.0742 | 0.0380574 | 0.2332687 | no |
| Sobic.001G030100 | 156.2095 | -0.9805 | 0.4727 | -2.0742 | 0.0380614 | 0.2332687 | no |
| Sobic.001G132400 | 4262.1535 | -0.5775 | 0.2785 | -2.0738 | 0.0381015 | 0.2333397 | no |
| Sobic.009G004100 | 8027.9987 | -1.2578 | 0.6066 | -2.0737 | 0.0381066 | 0.2333397 | no |
| Sobic.004G328100 | 156.9067 | 0.8720 | 0.4205 | 2.0737 | 0.0381084 | 0.2333397 | no |
| Sobic.009G097250 | 5.1834 | -2.6721 | 1.2886 | -2.0736 | 0.0381173 | 0.2333397 | no |
| Sobic.001G138200 | 941.3184 | -3.3821 | 1.6315 | -2.0730 | 0.0381736 | 0.2335496 | no |
| Sobic.006G060200 | 7610.3242 | -0.8555 | 0.4127 | -2.0729 | 0.0381793 | 0.2335496 | no |
| Sobic.009G168800 | 11.7038 | 2.0496 | 0.9888 | 2.0729 | 0.0381848 | 0.2335496 | no |
| Sobic.003G052300 | 2584.6892 | -0.7280 | 0.3512 | -2.0727 | 0.0382003 | 0.2335674 | no |
| Sobic.004G116300 | 2311.8353 | 1.2213 | 0.5893 | 2.0726 | 0.0382098 | 0.2335674 | no |
| Sobic.009G130300 | 6.0356 | 2.8474 | 1.3742 | 2.0721 | 0.0382561 | 0.2337825 | no |
| Sobic.006G129000 | 294.2696 | 1.0486 | 0.5061 | 2.0719 | 0.0382794 | 0.233821 | no |
| Sobic.004G137801 | 510.2545 | -0.8532 | 0.4118 | -2.0717 | 0.0382907 | 0.233821 | no |
| Sobic.001G355600 | 822.3787 | -1.3813 | 0.6668 | -2.0716 | 0.0383011 | 0.233821 | no |
| Sobic.003G328300 | 307.9307 | 0.9004 | 0.4347 | 2.0716 | 0.0383068 | 0.233821 | no |
| Sobic.007G213100 | 10731.8719 | -0.5612 | 0.2709 | -2.0714 | 0.0383252 | 0.2338657 | no |
| Sobic.007G062800 | 2128.2461 | 0.8646 | 0.4174 | 2.0712 | 0.0383379 | 0.2338722 | no |
| Sobic.001G371300 | 2211.8167 | -0.5371 | 0.2593 | -2.0711 | 0.0383484 | 0.2338722 | no |
| Sobic.009G106601 | 9.6704 | -2.1127 | 1.0204 | -2.0704 | 0.0384135 | 0.2340051 | no |
| Sobic.001G449900 | 819.7918 | 0.6761 | 0.3266 | 2.0704 | 0.038414 | 0.2340051 | no |
| Sobic.010G173900 | 11.5910 | 2.4887 | 1.2022 | 2.0701 | 0.038441 | 0.2340051 | no |
| Sobic.002G012800 | 34.8195 | 3.0512 | 1.4740 | 2.0701 | 0.038445 | 0.2340051 | no |
| Sobic.001G192200 | 676.3556 | 0.9189 | 0.4439 | 2.0699 | 0.0384596 | 0.2340051 | no |
| Sobic.001G506300 | 47.8755 | 1.0955 | 0.5293 | 2.0698 | 0.0384751 | 0.2340051 | no |
| Sobic.004G257000 | 936.1920 | 1.0733 | 0.5186 | 2.0697 | 0.0384786 | 0.2340051 | no |
| Sobic.007G166300 | 31188.4236 | -1.0577 | 0.5110 | -2.0697 | 0.0384839 | 0.2340051 | no |
| Sobic.001G382000 | 4346.2758 | 0.8198 | 0.3961 | 2.0694 | 0.0385073 | 0.2340051 | no |
| Sobic.002G329100 | 382.1770 | -0.8381 | 0.4050 | -2.0694 | 0.0385111 | 0.2340051 | no |
| Sobic.003G255100 | 3449.4607 | -1.1415 | 0.5516 | -2.0694 | 0.0385132 | 0.2340051 | no |
| Sobic.001G309900 | 626.3876 | 1.1680 | 0.5644 | 2.0693 | 0.0385205 | 0.2340051 | no |
| Sobic.010G064200 | 10.9808 | -1.7390 | 0.8404 | -2.0693 | 0.0385209 | 0.2340051 | no |
| Sobic.006G259000 | 1610.3269 | -1.2458 | 0.6021 | -2.0692 | 0.0385256 | 0.2340051 | no |

| Sobic.002G153000 | 120.8010 | 0.7619 | 0.3683 | 2.0688 | 0.0385647 | 0.2341753 | no |
| --- | --- | --- | --- | --- | --- | --- | --- |
| Sobic.002G401500 | 5566.7244 | 0.5446 | 0.2633 | 2.0685 | 0.0385942 | 0.2342868 | no |
| Sobic.010G249700 | 3566.5831 | -0.8502 | 0.4111 | -2.0683 | 0.0386084 | 0.2343058 | no |
| Sobic.006G259900 | 2181.6768 | 0.5790 | 0.2800 | 2.0681 | 0.0386314 | 0.2343761 | no |
| Sobic.001G236700 | 166.8653 | 1.3694 | 0.6622 | 2.0680 | 0.0386422 | 0.2343761 | no |
| Sobic.004G338100 | 301.7401 | 1.0606 | 0.5130 | 2.0674 | 0.0386967 | 0.2345979 | no |
| Sobic.003G378600 | 23369.5429 | -0.8575 | 0.4148 | -2.0674 | 0.038701 | 0.2345979 | no |
| Sobic.003G310300 | 278.0448 | 1.0458 | 0.5059 | 2.0670 | 0.0387346 | 0.2347339 | no |
| Sobic.001G127800 | 1130.6967 | -0.5168 | 0.2501 | -2.0661 | 0.0388221 | 0.2351966 | no |
| Sobic.001G541166 | 1749.3511 | -0.7349 | 0.3558 | -2.0656 | 0.0388685 | 0.235381 | no |
| Sobic.002G219300 | 653.5737 | -1.9701 | 0.9538 | -2.0655 | 0.0388749 | 0.235381 | no |
| Sobic.006G056400 | 81.2626 | 1.4330 | 0.6939 | 2.0652 | 0.0389008 | 0.2354705 | no |
| Sobic.010G185200 | 723.6412 | -0.7712 | 0.3735 | -2.0650 | 0.0389262 | 0.2355098 | no |
| Sobic.003G255800 | 279.4117 | 1.3360 | 0.6470 | 2.0649 | 0.0389296 | 0.2355098 | no |
| Sobic.005G059500 | 1550.4430 | 0.5872 | 0.2844 | 2.0646 | 0.0389576 | 0.2356113 | no |
| Sobic.010G214000 | 95525.9727 | -0.6233 | 0.3019 | -2.0644 | 0.0389799 | 0.2356203 | no |
| Sobic.003G188300 | 258.7825 | 0.9962 | 0.4826 | 2.0644 | 0.0389814 | 0.2356203 | no |
| Sobic.001G178600 | 1339.6937 | 0.7700 | 0.3731 | 2.0639 | 0.0390268 | 0.235809 | no |
| Sobic.009G182000 | 1612.8956 | 1.1513 | 0.5579 | 2.0638 | 0.0390356 | 0.235809 | no |
| Sobic.001G026900 | 1360.5826 | 1.6697 | 0.8091 | 2.0637 | 0.0390476 | 0.235809 | no |
| Sobic.007G151700 | 39.1645 | 2.5869 | 1.2536 | 2.0636 | 0.0390574 | 0.235809 | no |
| Sobic.009G036900 | 1125.0752 | -0.5534 | 0.2683 | -2.0626 | 0.0391495 | 0.2361976 | no |
| Sobic.001G159400 | 8784.7220 | -0.5892 | 0.2857 | -2.0626 | 0.0391544 | 0.2361976 | no |
| Sobic.004G147800 | 1521.5297 | -0.8059 | 0.3907 | -2.0626 | 0.0391553 | 0.2361976 | no |
| Sobic.009G055500 | 56.3789 | 1.2223 | 0.5927 | 2.0623 | 0.0391768 | 0.2361977 | no |
| Sobic.003G085300 | 115.4784 | 2.1186 | 1.0273 | 2.0622 | 0.0391841 | 0.2361977 | no |
| Sobic.002G004200 | 47.0676 | 1.4648 | 0.7103 | 2.0621 | 0.0392 | 0.2361977 | no |
| Sobic.006G256600 | 2982.7239 | -0.5982 | 0.2901 | -2.0620 | 0.039203 | 0.2361977 | no |
| Sobic.006G121500 | 26.0151 | 1.8781 | 0.9108 | 2.0619 | 0.0392177 | 0.2361977 | no |
| Sobic.007G033800 | 9532.6915 | -0.7499 | 0.3637 | -2.0618 | 0.0392269 | 0.2361977 | no |
| Sobic.003G362900 | 1386.3444 | 0.5940 | 0.2881 | 2.0617 | 0.0392337 | 0.2361977 | no |
| Sobic.001G195000 | 154.1734 | 1.4604 | 0.7084 | 2.0615 | 0.0392563 | 0.2362659 | no |
| Sobic.002G343500 | 529.2886 | 0.7136 | 0.3463 | 2.0610 | 0.0393056 | 0.2364949 | no |
| Sobic.001G134700 | 1556.1955 | -0.7481 | 0.3630 | -2.0605 | 0.0393466 | 0.2366742 | no |
| Sobic.006G125000 | 11407.9418 | -0.6099 | 0.2960 | -2.0604 | 0.0393641 | 0.2367124 | no |
| Sobic.001G456400 | 12.2607 | 2.7828 | 1.3507 | 2.0602 | 0.0393813 | 0.2367437 | no |
| Sobic.010G021200 | 937.4854 | -0.5412 | 0.2627 | -2.0601 | 0.0393918 | 0.2367437 | no |
| Sobic.002G417500 | 4523.6855 | 0.6331 | 0.3074 | 2.0595 | 0.0394459 | 0.2369465 | no |
| Sobic.003G074600 | 49.3739 | -1.7269 | 0.8385 | -2.0595 | 0.039448 | 0.2369465 | no |
| Sobic.001G316300 | 12.7228 | 2.6632 | 1.2934 | 2.0591 | 0.0394889 | 0.2369977 | no |
| Sobic.009G034200 | 3296.1380 | -0.9279 | 0.4506 | -2.0591 | 0.0394891 | 0.2369977 | no |
| Sobic.001G354800 | 36.5264 | -1.5361 | 0.7460 | -2.0590 | 0.0394902 | 0.2369977 | no |
| Sobic.002G294200 | 1405.3939 | 0.5324 | 0.2586 | 2.0584 | 0.0395525 | 0.2373039 | no |
| Sobic.001G161300 | 2328.0684 | -0.7269 | 0.3532 | -2.0582 | 0.0395744 | 0.2373676 | no |
| Sobic.001G368300 | 392.7720 | -0.9884 | 0.4803 | -2.0580 | 0.0395862 | 0.2373707 | no |
| Sobic.003G157400 | 364.5489 | -0.8624 | 0.4192 | -2.0573 | 0.0396593 | 0.2376338 | no |
| Sobic.010G056200 | 2758.8224 | -0.6824 | 0.3317 | -2.0573 | 0.0396602 | 0.2376338 | no |
| Sobic.005G193300 | 1276.6664 | -0.8887 | 0.4320 | -2.0572 | 0.0396639 | 0.2376338 | no |
| Sobic.010G256700 | 1206.6117 | -0.8282 | 0.4026 | -2.0570 | 0.0396831 | 0.2376818 | no |
| Sobic.005G194100 | 3849.5360 | -0.9834 | 0.4782 | -2.0564 | 0.0397426 | 0.2379703 | no |
| Sobic.001G015200 | 3488.2308 | -1.1255 | 0.5473 | -2.0562 | 0.0397596 | 0.2380046 | no |
| Sobic.001G400000 | 293.2517 | 0.9781 | 0.4758 | 2.0559 | 0.0397969 | 0.2381238 | no |
| Sobic.003G126800 | 5509.2308 | 0.9075 | 0.4415 | 2.0558 | 0.0398021 | 0.2381238 | no |
| Sobic.003G128800 | 75.7614 | -0.8397 | 0.4086 | -2.0551 | 0.0398668 | 0.2384435 | no |
| Sobic.008G159250 | 491.6351 | -0.7244 | 0.3526 | -2.0545 | 0.0399226 | 0.2387094 | no |
| Sobic.004G181000 | 87.3913 | 2.2368 | 1.0889 | 2.0541 | 0.0399644 | 0.2388809 | no |
| Sobic.010G024400 | 154.2843 | -0.9217 | 0.4487 | -2.0540 | 0.039974 | 0.2388809 | no |

| Sobic.003G156700 | 46.0247 | -1.6012 | 0.7796 | -2.0537 | 0.0400017 | 0.2389789 | no |
| --- | --- | --- | --- | --- | --- | --- | --- |
| Sobic.004G203600 | 1205.9771 | 0.6141 | 0.2991 | 2.0534 | 0.0400351 | 0.239047 | no |
| Sobic.003G432500 | 2064.2688 | -0.8166 | 0.3977 | -2.0534 | 0.0400358 | 0.239047 | no |
| Sobic.006G271300 | 161.4569 | -0.8523 | 0.4152 | -2.0530 | 0.0400739 | 0.2391662 | no |
| Sobic.006G224200 | 10.9774 | 2.5563 | 1.2452 | 2.0529 | 0.0400784 | 0.2391662 | no |
| Sobic.006G235900 | 61.5387 | 1.1546 | 0.5624 | 2.0528 | 0.0400912 | 0.2391748 | no |
| Sobic.003G027800 | 522.5447 | 0.6669 | 0.3249 | 2.0526 | 0.0401153 | 0.2392513 | no |
| Sobic.001G149000 | 576.5331 | -0.7149 | 0.3484 | -2.0522 | 0.0401496 | 0.239388 | no |
| Sobic.009G155900 | 391.2451 | 1.2488 | 0.6088 | 2.0511 | 0.0402567 | 0.2399583 | no |
| Sobic.003G043200 | 874.3824 | 0.8458 | 0.4124 | 2.0507 | 0.0402943 | 0.240115 | no |
| Sobic.002G060600 | 14.8521 | 1.6457 | 0.8026 | 2.0505 | 0.0403144 | 0.2401347 | no |
| Sobic.009G155050 | 183.6766 | -1.0687 | 0.5212 | -2.0505 | 0.0403204 | 0.2401347 | no |
| Sobic.010G231500 | 3004.1321 | -0.7911 | 0.3859 | -2.0499 | 0.0403709 | 0.2402803 | no |
| Sobic.003G007800 | 50430.0324 | -1.4412 | 0.7032 | -2.0495 | 0.0404087 | 0.2402803 | no |
| Sobic.007G033400 | 64.5055 | -1.9793 | 0.9658 | -2.0494 | 0.0404241 | 0.2402803 | no |
| Sobic.006G136900 | 269.1599 | -0.6655 | 0.3248 | -2.0494 | 0.0404251 | 0.2402803 | no |
| Sobic.003G254800 | 1337.5640 | -0.5078 | 0.2478 | -2.0494 | 0.0404269 | 0.2402803 | no |
| Sobic.006G132000 | 543.3063 | -0.6934 | 0.3384 | -2.0493 | 0.0404318 | 0.2402803 | no |
| Sobic.010G028400 | 229.9315 | 0.8424 | 0.4111 | 2.0493 | 0.040435 | 0.2402803 | no |
| Sobic.006G217900 | 115.1896 | 2.7911 | 1.3621 | 2.0491 | 0.0404498 | 0.2402803 | no |
| Sobic.004G095200 | 14.6420 | 2.0116 | 0.9818 | 2.0490 | 0.0404639 | 0.2402803 | no |
| Sobic.004G082200 | 76.7433 | -1.0085 | 0.4922 | -2.0489 | 0.0404723 | 0.2402803 | no |
| Sobic.003G157000 | 28.4668 | -1.8620 | 0.9088 | -2.0488 | 0.0404849 | 0.2402803 | no |
| Sobic.008G019400 | 234.5231 | 2.4823 | 1.2117 | 2.0487 | 0.0404909 | 0.2402803 | no |
| Sobic.003G388400 | 57.8860 | -1.2731 | 0.6215 | -2.0485 | 0.0405118 | 0.2402803 | no |
| Sobic.009G184600 | 81.8521 | 0.8110 | 0.3959 | 2.0484 | 0.0405257 | 0.2402803 | no |
| Sobic.010G244100 | 2312.9317 | -1.5561 | 0.7597 | -2.0483 | 0.04053 | 0.2402803 | no |
| Sobic.001G006900 | 74.5925 | -1.3092 | 0.6392 | -2.0482 | 0.0405413 | 0.2402803 | no |
| Sobic.004G344400 | 198.6688 | -0.8491 | 0.4146 | -2.0481 | 0.0405542 | 0.2402803 | no |
| Sobic.001G060666 | 5.6294 | -2.2439 | 1.0957 | -2.0480 | 0.0405636 | 0.2402803 | no |
| Sobic.002G336800 | 128.2552 | 1.2076 | 0.5897 | 2.0479 | 0.0405713 | 0.2402803 | no |
| Sobic.004G318200 | 75.8426 | 0.8421 | 0.4112 | 2.0478 | 0.0405787 | 0.2402803 | no |
| Sobic.005G137200 | 18.9460 | 2.2214 | 1.0848 | 2.0478 | 0.0405841 | 0.2402803 | no |
| Sobic.004G092700 | 12.8713 | -2.3244 | 1.1352 | -2.0476 | 0.0405978 | 0.2402937 | no |
| Sobic.002G220900 | 1935.0477 | -0.7523 | 0.3675 | -2.0473 | 0.0406296 | 0.2403546 | no |
| Sobic.003G290200 | 82.8821 | 0.9484 | 0.4632 | 2.0473 | 0.0406326 | 0.2403546 | no |
| Sobic.003G110800 | 1687.6772 | -0.4937 | 0.2411 | -2.0472 | 0.0406423 | 0.2403546 | no |
| Sobic.006G138200 | 580.0042 | -0.4593 | 0.2244 | -2.0468 | 0.0406789 | 0.2404639 | no |
| Sobic.008G167400 | 29.4138 | -1.1430 | 0.5584 | -2.0467 | 0.0406835 | 0.2404639 | no |
| Sobic.004G264000 | 22.5060 | 1.6634 | 0.8128 | 2.0464 | 0.0407196 | 0.2406094 | no |
| Sobic.001G261541 | 728.2264 | 0.5239 | 0.2560 | 2.0462 | 0.0407413 | 0.2406703 | no |
| Sobic.002G259900 | 42.1786 | 1.1202 | 0.5476 | 2.0459 | 0.0407636 | 0.2407199 | no |
| Sobic.009G235600 | 114.4605 | 0.7177 | 0.3508 | 2.0458 | 0.0407725 | 0.2407199 | no |
| Sobic.005G192200 | 263.6082 | 1.4593 | 0.7134 | 2.0457 | 0.0407892 | 0.2407513 | no |
| Sobic.001G321200 | 121.0469 | 0.9941 | 0.4861 | 2.0452 | 0.0408317 | 0.2409346 | no |
| Sobic.010G256000 | 27.6886 | 1.7040 | 0.8333 | 2.0451 | 0.0408493 | 0.2409706 | no |
| Sobic.004G034100 | 9.1973 | 3.2702 | 1.5993 | 2.0448 | 0.0408787 | 0.2410769 | no |
| Sobic.006G146800 | 1488.1582 | 0.6365 | 0.3113 | 2.0446 | 0.0408939 | 0.2410871 | no |
| Sobic.003G015600 | 6118.7795 | -1.0270 | 0.5024 | -2.0443 | 0.0409266 | 0.2410871 | no |
| Sobic.003G100700 | 46.5506 | 1.1043 | 0.5402 | 2.0442 | 0.0409312 | 0.2410871 | no |
| Sobic.003G186000 | 700.2940 | 0.8151 | 0.3987 | 2.0442 | 0.0409326 | 0.2410871 | no |
| Sobic.002G288300 | ######### | -1.1038 | 0.5400 | -2.0442 | 0.0409376 | 0.2410871 | no |
| Sobic.003G137400 | 352.6084 | -0.6427 | 0.3145 | -2.0439 | 0.0409658 | 0.241102 | no |
| Sobic.008G098900 | 1968.1863 | 0.5363 | 0.2624 | 2.0438 | 0.0409735 | 0.241102 | no |
| Sobic.002G041666 | 58.0207 | 1.5248 | 0.7461 | 2.0437 | 0.04098 | 0.241102 | no |
| Sobic.010G163100 | 146.4400 | -0.6816 | 0.3335 | -2.0437 | 0.0409858 | 0.241102 | no |
| Sobic.001G007300 | 861.5889 | -0.6431 | 0.3147 | -2.0434 | 0.0410174 | 0.2412202 | no |

| Sobic.008G101000 | 54.9844 | 0.9970 | 0.4880 | 2.0429 | 0.0410625 | 0.2414184 | no |
| --- | --- | --- | --- | --- | --- | --- | --- |
| Sobic.004G125100 | 1478.4152 | -0.5828 | 0.2854 | -2.0423 | 0.0411172 | 0.2416727 | no |
| Sobic.001G172700 | 104.6721 | 1.1793 | 0.5775 | 2.0421 | 0.0411406 | 0.2417425 | no |
| Sobic.009G026650 | 73.6129 | -1.2212 | 0.5981 | -2.0420 | 0.0411556 | 0.2417636 | no |
| Sobic.002G284500 | 12.7292 | -2.6008 | 1.2738 | -2.0418 | 0.0411728 | 0.241797 | no |
| Sobic.009G179600 | 225.2888 | 0.9957 | 0.4877 | 2.0415 | 0.0411982 | 0.2418127 | no |
| Sobic.001G458000 | 237.9096 | 0.5675 | 0.2780 | 2.0415 | 0.0411984 | 0.2418127 | no |
| Sobic.004G111700 | 651.2814 | -0.5022 | 0.2461 | -2.0411 | 0.0412437 | 0.2420105 | no |
| Sobic.001G311800 | 11.5896 | 1.8343 | 0.8988 | 2.0410 | 0.041255 | 0.2420105 | no |
| Sobic.001G104800 | 2116.6307 | -0.8828 | 0.4326 | -2.0407 | 0.0412791 | 0.2420733 | no |
| Sobic.003G169300 | 27.6384 | -1.4645 | 0.7177 | -2.0405 | 0.0412967 | 0.2420733 | no |
| Sobic.001G347700 | 253.8503 | 0.9051 | 0.4436 | 2.0405 | 0.0413002 | 0.2420733 | no |
| Sobic.005G012700 | 84.2388 | 0.7092 | 0.3476 | 2.0402 | 0.0413339 | 0.2422036 | no |
| Sobic.001G139600 | 11.4413 | -2.0509 | 1.0054 | -2.0399 | 0.0413557 | 0.242264 | no |
| Sobic.009G098100 | 2406.2686 | -0.9913 | 0.4861 | -2.0394 | 0.0414146 | 0.2425415 | no |
| Sobic.003G419700 | 5.5784 | 5.6924 | 2.7915 | 2.0392 | 0.0414313 | 0.2425719 | no |
| Sobic.009G257500 | 172.3930 | 0.7402 | 0.3630 | 2.0389 | 0.0414559 | 0.2426485 | no |
| Sobic.010G267650 | 37.5937 | 1.0235 | 0.5021 | 2.0387 | 0.0414837 | 0.2427438 | no |
| Sobic.001G317700 | 4.9234 | 3.6892 | 1.8098 | 2.0384 | 0.0415104 | 0.2428328 | no |
| Sobic.001G533000 | 69.6980 | 1.4099 | 0.6918 | 2.0381 | 0.0415353 | 0.2429071 | no |
| Sobic.008G080500 | 488.5056 | 0.7809 | 0.3832 | 2.0380 | 0.0415461 | 0.2429071 | no |
| Sobic.001G397200 | 12.1515 | -1.8297 | 0.8980 | -2.0376 | 0.0415917 | 0.2430894 | no |
| Sobic.007G059600 | 1454.5517 | 0.9828 | 0.4823 | 2.0375 | 0.0416003 | 0.2430894 | no |
| Sobic.005G063100 | 27.3073 | 1.8175 | 0.8924 | 2.0368 | 0.0416721 | 0.2434411 | no |
| Sobic.009G125400 | 1702.7419 | -1.0200 | 0.5010 | -2.0360 | 0.0417548 | 0.2437614 | no |
| Sobic.004G288200 | 95.5689 | 1.3735 | 0.6747 | 2.0359 | 0.0417588 | 0.2437614 | no |
| Sobic.002G249300 | 368.0029 | -0.6114 | 0.3003 | -2.0359 | 0.0417616 | 0.2437614 | no |
| Sobic.002G343400 | 1890.3061 | -0.6865 | 0.3373 | -2.0357 | 0.0417854 | 0.2438331 | no |
| Sobic.007G137700 | 588.2539 | 1.5286 | 0.7511 | 2.0352 | 0.04183 | 0.2438736 | no |
| Sobic.002G020900 | 1152.5420 | 1.2432 | 0.6109 | 2.0352 | 0.0418332 | 0.2438736 | no |
| Sobic.001G384400 | 234.9475 | 0.6117 | 0.3006 | 2.0352 | 0.0418353 | 0.2438736 | no |
| Sobic.001G433000 | 1183.4364 | -0.8278 | 0.4068 | -2.0351 | 0.04184 | 0.2438736 | no |
| Sobic.006G083201 | 10.9315 | 2.4394 | 1.1988 | 2.0349 | 0.0418592 | 0.2438736 | no |
| Sobic.001G286600 | 4859.9469 | -0.6544 | 0.3216 | -2.0349 | 0.0418617 | 0.2438736 | no |
| Sobic.007G164101 | 10.9867 | 2.0478 | 1.0065 | 2.0346 | 0.04189 | 0.2439705 | no |
| Sobic.002G379200 | 65.7320 | 1.6033 | 0.7882 | 2.0342 | 0.0419337 | 0.2441579 | no |
| Sobic.008G117100 | 617.0981 | -0.5983 | 0.2941 | -2.0340 | 0.0419522 | 0.2441982 | no |
| Sobic.006G252200 | 2255.2878 | 0.5796 | 0.2850 | 2.0337 | 0.041984 | 0.2443157 | no |
| Sobic.003G287900 | 3426.4151 | -0.9611 | 0.4726 | -2.0335 | 0.0419995 | 0.2443388 | no |
| Sobic.007G086600 | 169.2327 | 0.7008 | 0.3447 | 2.0332 | 0.0420373 | 0.2444379 | no |
| Sobic.006G276700 | 4137.8513 | -0.6501 | 0.3198 | -2.0331 | 0.0420398 | 0.2444379 | no |
| Sobic.002G241250 | 169.1337 | 1.7227 | 0.8474 | 2.0330 | 0.0420519 | 0.2444413 | no |
| Sobic.004G253100 | 1101.9767 | -0.4898 | 0.2410 | -2.0324 | 0.0421159 | 0.2447456 | no |
| Sobic.009G008200 | 219.0220 | 1.0749 | 0.5290 | 2.0319 | 0.0421648 | 0.2449625 | no |
| Sobic.004G208400 | 21494.4138 | -0.8361 | 0.4116 | -2.0312 | 0.042231 | 0.2451669 | no |
| Sobic.003G227100 | 1200.1987 | 0.6564 | 0.3232 | 2.0312 | 0.0422347 | 0.2451669 | no |
| Sobic.002G116300 | 68.6394 | 0.8639 | 0.4253 | 2.0312 | 0.0422349 | 0.2451669 | no |
| Sobic.010G074600 | 1080.3664 | -0.8269 | 0.4072 | -2.0306 | 0.0422981 | 0.2454664 | no |
| Sobic.001G300400 | 8.7734 | 3.7616 | 1.8527 | 2.0303 | 0.0423297 | 0.24549 | no |
| Sobic.001G491700 | 142.5674 | 0.8606 | 0.4239 | 2.0302 | 0.042335 | 0.24549 | no |
| Sobic.005G210700 | 13.1421 | 1.8940 | 0.9329 | 2.0302 | 0.0423371 | 0.24549 | no |
| Sobic.001G082500 | 93.1394 | 1.7184 | 0.8468 | 2.0294 | 0.04242 | 0.2459025 | no |
| Sobic.010G237000 | 3807.6652 | -0.5049 | 0.2488 | -2.0293 | 0.0424316 | 0.2459025 | no |
| Sobic.003G421100 | 1549.1391 | -1.0081 | 0.4968 | -2.0291 | 0.0424475 | 0.2459275 | no |
| Sobic.008G119800 | 410.3338 | -0.6267 | 0.3089 | -2.0285 | 0.042514 | 0.2461144 | no |
| Sobic.002G181200 | 84.8785 | 0.7716 | 0.3804 | 2.0284 | 0.0425224 | 0.2461144 | no |
| Sobic.005G028300 | 28.4424 | 1.1986 | 0.5910 | 2.0281 | 0.0425505 | 0.2461144 | no |

| Sobic.004G324000 | 261.0549 | -1.4360 | 0.7081 | -2.0280 | 0.0425631 | 0.2461144 | no |
| --- | --- | --- | --- | --- | --- | --- | --- |
| Sobic.007G173300 | 234.7544 | 0.7820 | 0.3856 | 2.0279 | 0.0425707 | 0.2461144 | no |
| Sobic.010G206000 | 364.6105 | 1.0959 | 0.5404 | 2.0278 | 0.0425759 | 0.2461144 | no |
| Sobic.010G117500 | 29.9865 | 2.3673 | 1.1674 | 2.0278 | 0.0425785 | 0.2461144 | no |
| Sobic.004G023000 | 447.4393 | 0.8713 | 0.4297 | 2.0278 | 0.0425792 | 0.2461144 | no |
| Sobic.006G173000 | 5954.3011 | -0.9308 | 0.4590 | -2.0277 | 0.0425941 | 0.2461144 | no |
| Sobic.002G300600 | 1085.7832 | -0.8823 | 0.4351 | -2.0276 | 0.0425965 | 0.2461144 | no |
| Sobic.007G025500 | 1997.7967 | 0.7018 | 0.3462 | 2.0273 | 0.0426293 | 0.2462367 | no |
| Sobic.008G156700 | 285.3194 | 0.6465 | 0.3189 | 2.0270 | 0.0426673 | 0.2463368 | no |
| Sobic.001G514100 | 1764.5511 | -0.9085 | 0.4482 | -2.0268 | 0.0426795 | 0.2463368 | no |
| Sobic.001G474700 | 315.5623 | 0.5804 | 0.2864 | 2.0268 | 0.0426817 | 0.2463368 | no |
| Sobic.004G238100 | 29935.9885 | 1.2138 | 0.5991 | 2.0260 | 0.0427642 | 0.2467034 | no |
| Sobic.001G466500 | 47.6472 | 1.0206 | 0.5038 | 2.0260 | 0.0427686 | 0.2467034 | no |
| Sobic.009G135900 | 11943.0356 | -0.4474 | 0.2209 | -2.0256 | 0.0428095 | 0.2468714 | no |
| Sobic.006G148400 | 782.5890 | -0.6680 | 0.3299 | -2.0247 | 0.0428978 | 0.2473132 | no |
| Sobic.004G194100 | 200.0537 | -0.7863 | 0.3885 | -2.0241 | 0.0429566 | 0.2475248 | no |
| Sobic.004G100000 | 63.8035 | -0.9333 | 0.4611 | -2.0240 | 0.0429688 | 0.2475248 | no |
| Sobic.001G465100 | 56907.1027 | -1.0239 | 0.5059 | -2.0240 | 0.0429697 | 0.2475248 | no |
| Sobic.005G038500 | 301.4870 | 1.2229 | 0.6043 | 2.0236 | 0.0430072 | 0.2476728 | no |
| Sobic.006G221600 | 152.1573 | 1.1402 | 0.5636 | 2.0232 | 0.0430507 | 0.2478559 | no |
| Sobic.001G541700 | 277.7607 | -0.8733 | 0.4317 | -2.0230 | 0.043072 | 0.2479109 | no |
| Sobic.010G086600 | 473.3521 | 0.6420 | 0.3174 | 2.0228 | 0.0430914 | 0.2479323 | no |
| Sobic.010G065800 | 2829.5607 | -1.0290 | 0.5087 | -2.0227 | 0.0430992 | 0.2479323 | no |
| Sobic.007G157800 | 15.8900 | -1.8510 | 0.9153 | -2.0223 | 0.0431412 | 0.248106 | no |
| Sobic.002G215800 | 356.6798 | -1.2399 | 0.6132 | -2.0220 | 0.0431763 | 0.2482011 | no |
| Sobic.001G457900 | 2115.0175 | 0.7215 | 0.3568 | 2.0220 | 0.0431813 | 0.2482011 | no |
| Sobic.002G312200 | 202.6261 | -0.9748 | 0.4823 | -2.0213 | 0.0432488 | 0.2485214 | no |
| Sobic.001G463800 | 65.7128 | 1.7675 | 0.8746 | 2.0210 | 0.0432763 | 0.248559 | no |
| Sobic.006G252466 | 64.4041 | -1.1647 | 0.5763 | -2.0210 | 0.0432789 | 0.248559 | no |
| Sobic.007G201500 | 4066.4498 | -0.8268 | 0.4091 | -2.0208 | 0.0432956 | 0.2485873 | no |
| Sobic.009G159900 | 982.7312 | 1.0167 | 0.5032 | 2.0206 | 0.0433245 | 0.2486855 | no |
| Sobic.005G206300 | 3961.8598 | -0.8059 | 0.3989 | -2.0201 | 0.0433707 | 0.2487845 | no |
| Sobic.009G094700 | 1321.3009 | 0.9227 | 0.4568 | 2.0201 | 0.043377 | 0.2487845 | no |
| Sobic.004G171800 | 961.1175 | -0.8551 | 0.4233 | -2.0201 | 0.0433771 | 0.2487845 | no |
| Sobic.001G223400 | 13.4772 | -2.1399 | 1.0595 | -2.0197 | 0.0434133 | 0.2488733 | no |
| Sobic.001G489900 | 8418.0680 | -0.8430 | 0.4174 | -2.0197 | 0.0434162 | 0.2488733 | no |
| Sobic.001G400500 | 765.2496 | 0.7381 | 0.3655 | 2.0194 | 0.0434479 | 0.2489872 | no |
| Sobic.004G298800 | 132.4463 | -0.9571 | 0.4741 | -2.0188 | 0.0435038 | 0.2492399 | no |
| Sobic.009G033300 | 5.5130 | 2.9726 | 1.4726 | 2.0186 | 0.0435283 | 0.2493127 | no |
| Sobic.006G128300 | 736.9312 | -0.6314 | 0.3129 | -2.0180 | 0.0435955 | 0.2495942 | no |
| Sobic.005G175600 | 5817.6931 | 0.9794 | 0.4854 | 2.0177 | 0.0436184 | 0.2495942 | no |
| Sobic.010G203400 | 19.7654 | -2.0300 | 1.0061 | -2.0177 | 0.0436232 | 0.2495942 | no |
| Sobic.002G107900 | 6.9882 | 2.5110 | 1.2445 | 2.0177 | 0.0436248 | 0.2495942 | no |
| Sobic.007G113800 | 1128.6948 | -0.8237 | 0.4084 | -2.0168 | 0.0437117 | 0.2500234 | no |
| Sobic.004G312700 | 143.5543 | -0.9828 | 0.4874 | -2.0166 | 0.0437415 | 0.2501258 | no |
| Sobic.002G159400 | 30.1630 | 1.6604 | 0.8235 | 2.0162 | 0.0437765 | 0.2502582 | no |
| Sobic.003G328700 | 2509.5023 | -0.6452 | 0.3200 | -2.0160 | 0.0437977 | 0.2502984 | no |
| Sobic.006G078350 | 46.1510 | 1.2799 | 0.6349 | 2.0159 | 0.0438073 | 0.2502984 | no |
| Sobic.004G042700 | 17.8842 | 1.7313 | 0.8589 | 2.0158 | 0.0438206 | 0.2503069 | no |
| Sobic.002G407200 | 1256.3927 | -1.2756 | 0.6328 | -2.0157 | 0.0438336 | 0.2503131 | no |
| Sobic.001G125200 | 1293.5279 | 0.8020 | 0.3979 | 2.0155 | 0.0438545 | 0.2503651 | no |
| Sobic.006G191500 | 14366.7663 | -0.6983 | 0.3465 | -2.0152 | 0.0438796 | 0.25044 | no |
| Sobic.010G171300 | 220.2689 | -0.7595 | 0.3770 | -2.0147 | 0.043936 | 0.2506942 | no |
| Sobic.004G177000 | 90.2010 | 1.0312 | 0.5119 | 2.0145 | 0.0439556 | 0.2507385 | no |
| Sobic.001G018400 | 7.4642 | -3.2738 | 1.6254 | -2.0142 | 0.0439868 | 0.2508483 | no |
| Sobic.008G011200 | 163.8791 | -1.1800 | 0.5861 | -2.0133 | 0.0440875 | 0.251355 | no |
| Sobic.006G156801 | 246.9945 | -0.6365 | 0.3163 | -2.0126 | 0.0441578 | 0.2516873 | no |

| Sobic.007G093900 | 436.9646 | -0.7962 | 0.3957 | -2.0122 | 0.0441965 | 0.2518399 | no |
| --- | --- | --- | --- | --- | --- | --- | --- |
| Sobic.010G197500 | 2253.2679 | 0.9931 | 0.4936 | 2.0121 | 0.0442094 | 0.2518454 | no |
| Sobic.002G177600 | 25.4304 | 1.7941 | 0.8919 | 2.0115 | 0.0442693 | 0.2521186 | no |
| Sobic.001G456200 | 1422.0102 | -0.8519 | 0.4236 | -2.0112 | 0.0443009 | 0.2522305 | no |
| Sobic.004G189800 | 21078.3816 | -1.0450 | 0.5196 | -2.0111 | 0.0443185 | 0.2522628 | no |
| Sobic.003G222700 | 318.6125 | 0.5285 | 0.2628 | 2.0108 | 0.0443487 | 0.252305 | no |
| Sobic.007G072100 | 52.0322 | 1.7297 | 0.8602 | 2.0108 | 0.0443499 | 0.252305 | no |
| Sobic.010G255700 | 765.6856 | -0.5932 | 0.2950 | -2.0106 | 0.0443672 | 0.2523092 | no |
| Sobic.001G131400 | 3516.8457 | -0.8934 | 0.4444 | -2.0104 | 0.0443889 | 0.2523092 | no |
| Sobic.003G137300 | 15.0671 | 1.7758 | 0.8833 | 2.0104 | 0.0443895 | 0.2523092 | no |
| Sobic.001G213200 | 6.5001 | 2.5518 | 1.2694 | 2.0102 | 0.0444078 | 0.2523092 | no |
| Sobic.001G195100 | 2994.0967 | 1.0359 | 0.5153 | 2.0102 | 0.0444104 | 0.2523092 | no |
| Sobic.006G212400 | 13.2246 | 2.3208 | 1.1546 | 2.0101 | 0.0444239 | 0.2523178 | no |
| Sobic.008G047300 | 797.2610 | 0.5497 | 0.2735 | 2.0099 | 0.044441 | 0.2523469 | no |
| Sobic.005G148500 | 7.9653 | 2.2413 | 1.1155 | 2.0093 | 0.0445055 | 0.2526452 | no |
| Sobic.002G297400 | 1652.9741 | -0.8854 | 0.4407 | -2.0091 | 0.0445216 | 0.2526687 | no |
| Sobic.004G199900 | 243.7757 | -1.2478 | 0.6211 | -2.0090 | 0.044542 | 0.2527163 | no |
| Sobic.009G000900 | 374.8337 | -1.4386 | 0.7161 | -2.0088 | 0.0445557 | 0.2527259 | no |
| Sobic.004G071500 | 730.7917 | 1.0006 | 0.4982 | 2.0086 | 0.0445843 | 0.2528203 | no |
| Sobic.002G258400 | 1153.3045 | 1.6058 | 0.7997 | 2.0081 | 0.0446294 | 0.2529546 | no |
| Sobic.008G106900 | 73.1122 | 1.2212 | 0.6081 | 2.0081 | 0.044632 | 0.2529546 | no |
| Sobic.001G180200 | 72159.7496 | -0.7528 | 0.3749 | -2.0079 | 0.0446492 | 0.252984 | no |
| Sobic.002G330400 | 717.6656 | -0.9080 | 0.4523 | -2.0075 | 0.0447012 | 0.253175 | no |
| Sobic.003G198150 | 145.5160 | 1.5970 | 0.7956 | 2.0074 | 0.0447069 | 0.253175 | no |
| Sobic.002G316500 | 71.7043 | 0.9878 | 0.4921 | 2.0073 | 0.0447194 | 0.2531777 | no |
| Sobic.002G272100 | 444.6465 | -0.8231 | 0.4101 | -2.0069 | 0.044761 | 0.2533189 | no |
| Sobic.001G335700 | 10.4984 | 2.1310 | 1.0619 | 2.0068 | 0.0447704 | 0.2533189 | no |
| Sobic.003G442500 | 1524.0425 | 1.4760 | 0.7355 | 2.0067 | 0.0447804 | 0.2533189 | no |
| Sobic.006G215400 | 609.4688 | -0.9865 | 0.4917 | -2.0064 | 0.0448106 | 0.253422 | no |
| Sobic.003G445500 | 624.9671 | 0.7462 | 0.3719 | 2.0063 | 0.0448283 | 0.2534302 | no |
| Sobic.002G034700 | 492.3575 | 0.8283 | 0.4129 | 2.0061 | 0.0448448 | 0.2534302 | no |
| Sobic.002G198100 | 193.8256 | 1.0526 | 0.5247 | 2.0060 | 0.0448564 | 0.2534302 | no |
| Sobic.006G229800 | 27.2304 | -1.4741 | 0.7348 | -2.0060 | 0.0448601 | 0.2534302 | no |
| Sobic.002G309800 | 7373.7336 | -0.4795 | 0.2391 | -2.0057 | 0.0448892 | 0.2534724 | no |
| Sobic.004G110500 | 5.7108 | 2.3481 | 1.1708 | 2.0057 | 0.0448916 | 0.2534724 | no |
| Sobic.004G004200 | 3107.5923 | 0.5441 | 0.2714 | 2.0051 | 0.0449556 | 0.2537657 | no |
| Sobic.001G411900 | 329.2091 | 0.5231 | 0.2609 | 2.0048 | 0.0449798 | 0.2538341 | no |
| Sobic.002G315400 | 17.4795 | 2.1054 | 1.0503 | 2.0046 | 0.0450026 | 0.2538652 | no |
| Sobic.007G084200 | 72.8614 | 1.1265 | 0.5620 | 2.0046 | 0.0450093 | 0.2538652 | no |
| Sobic.002G316800 | 201.7701 | 0.9648 | 0.4815 | 2.0037 | 0.0450997 | 0.2543066 | no |
| Sobic.009G053201 | 204.4882 | -1.0935 | 0.5459 | -2.0033 | 0.0451464 | 0.254502 | no |
| Sobic.007G118600 | 859.5950 | 0.5738 | 0.2865 | 2.0029 | 0.0451854 | 0.2546541 | no |
| Sobic.006G035600 | 11.5310 | 2.3963 | 1.1965 | 2.0027 | 0.0452068 | 0.2547066 | no |
| Sobic.001G329600 | 26.2320 | 1.5302 | 0.7643 | 2.0021 | 0.0452769 | 0.2550334 | no |
| Sobic.004G287500 | 14.4589 | -2.5679 | 1.2827 | -2.0020 | 0.0452891 | 0.2550339 | no |
| Sobic.002G143300 | 72.3872 | 2.3866 | 1.1923 | 2.0016 | 0.0453283 | 0.2551007 | no |
| Sobic.002G243000 | 215.7204 | 0.9197 | 0.4595 | 2.0016 | 0.0453327 | 0.2551007 | no |
| Sobic.001G268300 | 49.5160 | 1.1767 | 0.5879 | 2.0015 | 0.0453373 | 0.2551007 | no |
| Sobic.003G111900 | 14.7499 | 2.1092 | 1.0539 | 2.0014 | 0.0453516 | 0.2551133 | no |
| Sobic.001G046500 | 21.0167 | 1.9018 | 0.9504 | 2.0010 | 0.0453921 | 0.2552732 | no |
| Sobic.006G208300 | 46.8868 | 1.3483 | 0.6739 | 2.0009 | 0.0454072 | 0.2552901 | no |
| Sobic.005G014400 | 17.0789 | 1.9242 | 0.9621 | 2.0001 | 0.0454933 | 0.2557061 | no |
| Sobic.004G001800 | 7.9641 | -3.0288 | 1.5154 | -1.9987 | 0.0456382 | 0.2564293 | no |
| Sobic.003G406900 | 346.9727 | -1.0946 | 0.5477 | -1.9986 | 0.0456463 | 0.2564293 | no |
| Sobic.002G111700 | 53.8865 | 1.0835 | 0.5422 | 1.9985 | 0.0456631 | 0.2564554 | no |
| Sobic.007G161100 | 18.3098 | 2.1457 | 1.0738 | 1.9982 | 0.045691 | 0.2565437 | no |
| Sobic.007G146900 | 1701.4414 | 0.6056 | 0.3031 | 1.9981 | 0.0457089 | 0.2565756 | no |

| Sobic.006G115100 | 791.8119 | -0.5195 | 0.2600 | -1.9980 | 0.0457215 | 0.2565781 | no |
| --- | --- | --- | --- | --- | --- | --- | --- |
| Sobic.002G198000 | 2410.2965 | -0.9747 | 0.4881 | -1.9971 | 0.0458154 | 0.2569169 | no |
| Sobic.007G060100 | 53.0463 | 1.6848 | 0.8436 | 1.9971 | 0.0458165 | 0.2569169 | no |
| Sobic.002G413600 | 249.3872 | 0.7225 | 0.3618 | 1.9971 | 0.0458184 | 0.2569169 | no |
| Sobic.003G308800 | 161.7908 | 0.7582 | 0.3797 | 1.9969 | 0.0458343 | 0.2569377 | no |
| Sobic.001G313900 | 366.4431 | -0.6771 | 0.3391 | -1.9965 | 0.0458826 | 0.2571133 | no |
| Sobic.003G370500 | 703.2590 | 0.7621 | 0.3818 | 1.9963 | 0.0459034 | 0.2571133 | no |
| Sobic.001G533300 | 2362.9703 | -0.5605 | 0.2808 | -1.9962 | 0.0459106 | 0.2571133 | no |
| Sobic.007G192000 | 18.0059 | -1.9992 | 1.0015 | -1.9962 | 0.0459171 | 0.2571133 | no |
| Sobic.008G086300 | 5.9253 | 2.7355 | 1.3704 | 1.9961 | 0.0459266 | 0.2571133 | no |
| Sobic.004G164100 | 134.2337 | -1.1928 | 0.5977 | -1.9955 | 0.0459838 | 0.2573276 | no |
| Sobic.003G125200 | 143.0510 | -1.0134 | 0.5079 | -1.9954 | 0.0460014 | 0.2573276 | no |
| Sobic.005G047100 | 13.4435 | 2.9773 | 1.4921 | 1.9953 | 0.0460096 | 0.2573276 | no |
| Sobic.001G261408 | 5031.3713 | -1.5304 | 0.7671 | -1.9951 | 0.0460363 | 0.2573276 | no |
| Sobic.006G157800 | 18.4634 | -1.7061 | 0.8552 | -1.9950 | 0.0460385 | 0.2573276 | no |
| Sobic.004G082500 | 1001.2615 | -0.7772 | 0.3896 | -1.9949 | 0.0460569 | 0.2573276 | no |
| Sobic.002G353000 | 710.6239 | -0.7301 | 0.3660 | -1.9948 | 0.0460617 | 0.2573276 | no |
| Sobic.003G265900 | 177.4305 | 1.3698 | 0.6867 | 1.9948 | 0.0460661 | 0.2573276 | no |
| Sobic.001G196700 | 1981.4681 | 0.4844 | 0.2428 | 1.9947 | 0.0460747 | 0.2573276 | no |
| Sobic.001G213500 | 26.4613 | 2.1034 | 1.0547 | 1.9944 | 0.0461085 | 0.2573694 | no |
| Sobic.003G111600 | 53.6238 | -1.2450 | 0.6243 | -1.9943 | 0.0461173 | 0.2573694 | no |
| Sobic.007G192300 | 67.0643 | -1.1704 | 0.5869 | -1.9943 | 0.0461188 | 0.2573694 | no |
| Sobic.003G161800 | 434.6203 | 0.9379 | 0.4705 | 1.9935 | 0.0462056 | 0.257786 | no |
| Sobic.004G297600 | 1185.0469 | -0.9299 | 0.4666 | -1.9931 | 0.0462486 | 0.2579575 | no |
| Sobic.002G023300 | 37877.4217 | 1.4224 | 0.7138 | 1.9927 | 0.046299 | 0.2581392 | no |
| Sobic.002G018500 | 23.4896 | -3.3297 | 1.6711 | -1.9926 | 0.0463057 | 0.2581392 | no |
| Sobic.001G462100 | 994.6848 | -0.6602 | 0.3315 | -1.9917 | 0.0464038 | 0.2586034 | no |
| Sobic.003G070800 | 1071.5218 | 0.5608 | 0.2816 | 1.9915 | 0.0464236 | 0.2586034 | no |
| Sobic.002G266200 | 98.4416 | 1.0966 | 0.5506 | 1.9915 | 0.0464257 | 0.2586034 | no |
| Sobic.002G254600 | 60.4440 | 1.3436 | 0.6748 | 1.9913 | 0.0464501 | 0.2586706 | no |
| Sobic.001G433900 | 181.0814 | 1.1854 | 0.5955 | 1.9907 | 0.0465158 | 0.2589685 | no |
| Sobic.007G102700 | 969.5617 | -0.6455 | 0.3243 | -1.9906 | 0.0465299 | 0.2589783 | no |
| Sobic.001G272900 | 24.1046 | 1.9142 | 0.9620 | 1.9897 | 0.0466207 | 0.2592634 | no |
| Sobic.002G145000 | 4848.2578 | -0.6603 | 0.3318 | -1.9897 | 0.0466212 | 0.2592634 | no |
| Sobic.010G078600 | 86.7535 | 1.2333 | 0.6199 | 1.9897 | 0.0466222 | 0.2592634 | no |
| Sobic.002G037200 | 182.5496 | -1.0418 | 0.5236 | -1.9896 | 0.0466303 | 0.2592634 | no |
| Sobic.K010300 | 5.1969 | 5.5920 | 2.8107 | 1.9895 | 0.0466455 | 0.2592794 | no |
| Sobic.003G288300 | 108.3636 | 1.4466 | 0.7272 | 1.9892 | 0.0466801 | 0.2593427 | no |
| Sobic.003G092900 | 343.7283 | -0.5695 | 0.2863 | -1.9892 | 0.0466814 | 0.2593427 | no |
| Sobic.002G339000 | 80.4456 | 1.6323 | 0.8207 | 1.9889 | 0.0467177 | 0.2593787 | no |
| Sobic.007G090463 | 412.2933 | -0.9198 | 0.4625 | -1.9888 | 0.0467236 | 0.2593787 | no |
| Sobic.002G319400 | 54.8474 | 2.1613 | 1.0867 | 1.9888 | 0.0467248 | 0.2593787 | no |
| Sobic.005G130200 | 785.2759 | -0.9209 | 0.4631 | -1.9885 | 0.0467542 | 0.2594737 | no |
| Sobic.001G233900 | 20.0041 | 1.7564 | 0.8834 | 1.9881 | 0.0467979 | 0.2595859 | no |
| Sobic.001G037200 | 17.8022 | -1.9095 | 0.9604 | -1.9881 | 0.0467991 | 0.2595859 | no |
| Sobic.007G221100 | 211.2438 | 0.6875 | 0.3458 | 1.9878 | 0.0468363 | 0.259697 | no |
| Sobic.003G344200 | 1250.1864 | -1.1196 | 0.5633 | -1.9876 | 0.0468517 | 0.259697 | no |
| Sobic.010G036000 | 2429.5979 | -0.7518 | 0.3782 | -1.9876 | 0.046856 | 0.259697 | no |
| Sobic.001G366300 | 128.6307 | 1.2622 | 0.6352 | 1.9873 | 0.0468886 | 0.2598021 | no |
| Sobic.003G076600 | 1038.8970 | -0.8300 | 0.4177 | -1.9871 | 0.0469068 | 0.2598021 | no |
| Sobic.002G199500 | 2809.8119 | -1.8512 | 0.9316 | -1.9871 | 0.046912 | 0.2598021 | no |
| Sobic.003G304200 | 342.4315 | 0.5662 | 0.2850 | 1.9870 | 0.0469252 | 0.2598073 | no |
| Sobic.004G305733 | 414.5754 | 1.0718 | 0.5395 | 1.9868 | 0.0469475 | 0.2598265 | no |
| Sobic.001G073900 | 19748.9299 | -0.6937 | 0.3492 | -1.9867 | 0.0469533 | 0.2598265 | no |
| Sobic.004G012100 | 2394.3745 | -0.4880 | 0.2457 | -1.9864 | 0.0469924 | 0.2599745 | no |
| Sobic.001G489800 | 1171.1356 | -0.7647 | 0.3850 | -1.9862 | 0.0470115 | 0.2600121 | no |
| Sobic.001G266500 | 114.6825 | -0.8876 | 0.4470 | -1.9857 | 0.047066 | 0.2601119 | no |

| Sobic.001G097900 | 715.6669 | 0.7383 | 0.3718 | 1.9857 | 0.0470662 | 0.2601119 | no |
| --- | --- | --- | --- | --- | --- | --- | --- |
| Sobic.010G249200 | 1662.6558 | -0.6024 | 0.3034 | -1.9856 | 0.0470767 | 0.2601119 | no |
| Sobic.005G008001 | 15.1947 | -2.4589 | 1.2384 | -1.9855 | 0.0470905 | 0.2601119 | no |
| Sobic.009G010150 | 8.1323 | 6.2375 | 3.1417 | 1.9854 | 0.0470993 | 0.2601119 | no |
| Sobic.004G008000 | 105.2193 | -0.7544 | 0.3800 | -1.9853 | 0.0471079 | 0.2601119 | no |
| Sobic.004G242500 | 499.4583 | 0.8540 | 0.4302 | 1.9853 | 0.0471159 | 0.2601119 | no |
| Sobic.007G214900 | 10.7528 | -1.6027 | 0.8074 | -1.9850 | 0.0471488 | 0.2602255 | no |
| Sobic.004G035750 | 28.7512 | -2.6358 | 1.3280 | -1.9847 | 0.0471785 | 0.2602615 | no |
| Sobic.004G261900 | 2681.0538 | 1.1109 | 0.5597 | 1.9846 | 0.0471845 | 0.2602615 | no |
| Sobic.003G428300 | 2911.6719 | 1.3616 | 0.6861 | 1.9846 | 0.0471924 | 0.2602615 | no |
| Sobic.010G238100 | 181.5696 | -0.8917 | 0.4494 | -1.9841 | 0.0472489 | 0.260505 | no |
| Sobic.002G354501 | 2667.5722 | -1.0611 | 0.5348 | -1.9839 | 0.0472642 | 0.2605214 | no |
| Sobic.004G349175 | 464.9338 | 1.3228 | 0.6669 | 1.9835 | 0.0473133 | 0.2607237 | no |
| Sobic.002G375800 | 1823.2292 | 1.0004 | 0.5046 | 1.9826 | 0.0474124 | 0.2611517 | no |
| Sobic.003G262600 | 1440.2865 | 0.4649 | 0.2345 | 1.9826 | 0.0474157 | 0.2611517 | no |
| Sobic.010G034000 | 328.1647 | 0.6837 | 0.3450 | 1.9815 | 0.047531 | 0.2616399 | no |
| Sobic.003G269800 | 459.4742 | 1.0089 | 0.5092 | 1.9815 | 0.0475398 | 0.2616399 | no |
| Sobic.003G363800 | 1065.3838 | -0.6504 | 0.3282 | -1.9814 | 0.0475416 | 0.2616399 | no |
| Sobic.002G375900 | 69.0300 | -1.2838 | 0.6481 | -1.9809 | 0.0476042 | 0.2618869 | no |
| Sobic.001G114200 | 564.4160 | 0.5945 | 0.3001 | 1.9808 | 0.0476113 | 0.2618869 | no |
| Sobic.001G473500 | 5.7323 | 3.0296 | 1.5298 | 1.9804 | 0.0476635 | 0.2621058 | no |
| Sobic.006G098100 | 4557.7047 | 0.5285 | 0.2669 | 1.9797 | 0.0477347 | 0.2623869 | no |
| Sobic.004G228800 | 646.4361 | 0.7629 | 0.3854 | 1.9797 | 0.0477395 | 0.2623869 | no |
| Sobic.002G104600 | 87.6785 | 0.7720 | 0.3900 | 1.9794 | 0.0477696 | 0.2624837 | no |
| Sobic.003G075800 | 641.2275 | -0.6946 | 0.3510 | -1.9791 | 0.0478071 | 0.2626214 | no |
| Sobic.001G062500 | 25.5469 | 2.7924 | 1.4111 | 1.9789 | 0.0478281 | 0.2626686 | no |
| Sobic.003G002900 | 25.5786 | -1.3897 | 0.7025 | -1.9783 | 0.0478955 | 0.2629701 | no |
| Sobic.010G092900 | 986.2300 | 1.5165 | 0.7667 | 1.9779 | 0.0479449 | 0.263071 | no |
| Sobic.001G207400 | 294.0735 | 0.5063 | 0.2560 | 1.9778 | 0.0479493 | 0.263071 | no |
| Sobic.007G092700 | 1262.5413 | 0.6012 | 0.3040 | 1.9778 | 0.0479513 | 0.263071 | no |
| Sobic.006G229300 | 698.8684 | -0.7998 | 0.4046 | -1.9770 | 0.0480452 | 0.2635174 | no |
| Sobic.002G136800 | 26.4112 | 1.2891 | 0.6521 | 1.9768 | 0.0480697 | 0.2635834 | no |
| Sobic.009G135100 | 2487.6647 | 1.1395 | 0.5765 | 1.9766 | 0.0480869 | 0.2636094 | no |
| Sobic.001G431400 | 160.9150 | -0.7157 | 0.3622 | -1.9762 | 0.0481341 | 0.2637996 | no |
| Sobic.004G245100 | 74.2375 | 1.7588 | 0.8902 | 1.9757 | 0.0481888 | 0.2640306 | no |
| Sobic.003G127800 | 13968.9107 | -1.1644 | 0.5895 | -1.9753 | 0.0482333 | 0.2641075 | no |
| Sobic.002G427300 | 1517.2706 | 0.6716 | 0.3400 | 1.9752 | 0.0482398 | 0.2641075 | no |
| Sobic.001G511900 | 444.6866 | 0.9328 | 0.4722 | 1.9752 | 0.0482407 | 0.2641075 | no |
| Sobic.004G017100 | 28.0248 | 1.0229 | 0.5179 | 1.9751 | 0.0482544 | 0.2641075 | no |
| Sobic.008G156400 | 6445.0579 | -0.5052 | 0.2558 | -1.9750 | 0.0482658 | 0.2641075 | no |
| Sobic.001G185450 | 5.5387 | 3.1090 | 1.5744 | 1.9747 | 0.0483003 | 0.2641075 | no |
| Sobic.010G278600 | 3762.9438 | 0.5139 | 0.2602 | 1.9746 | 0.0483114 | 0.2641075 | no |
| Sobic.005G059600 | 1143.3189 | 0.5616 | 0.2844 | 1.9745 | 0.048321 | 0.2641075 | no |
| Sobic.003G028000 | 8.7742 | 2.0374 | 1.0319 | 1.9745 | 0.048321 | 0.2641075 | no |
| Sobic.002G355400 | 1086.5917 | 0.9279 | 0.4699 | 1.9745 | 0.0483281 | 0.2641075 | no |
| Sobic.001G319000 | 23.9011 | 1.3883 | 0.7032 | 1.9742 | 0.0483617 | 0.2641384 | no |
| Sobic.004G206600 | 11.4264 | 2.4653 | 1.2488 | 1.9741 | 0.0483689 | 0.2641384 | no |
| Sobic.004G284600 | 159.4839 | 1.1940 | 0.6049 | 1.9741 | 0.0483713 | 0.2641384 | no |
| Sobic.002G430000 | 1314.3252 | 0.6121 | 0.3101 | 1.9739 | 0.0483938 | 0.2641926 | no |
| Sobic.003G017100 | 3916.3827 | -0.7344 | 0.3721 | -1.9738 | 0.04841 | 0.264213 | no |
| Sobic.003G078100 | 143.7373 | -0.8017 | 0.4063 | -1.9733 | 0.0484652 | 0.264379 | no |
| Sobic.010G008100 | 1464.9473 | 0.6592 | 0.3341 | 1.9733 | 0.0484655 | 0.264379 | no |
| Sobic.003G036900 | 1515.2800 | 1.0904 | 0.5529 | 1.9722 | 0.048583 | 0.2649516 | no |
| Sobic.002G395300 | 1827.6925 | -1.0236 | 0.5191 | -1.9720 | 0.0486147 | 0.2650556 | no |
| Sobic.003G301400 | 322.4001 | 0.8361 | 0.4241 | 1.9712 | 0.0486972 | 0.2654369 | no |
| Sobic.009G134400 | 7.6477 | 2.2216 | 1.1273 | 1.9707 | 0.0487548 | 0.2656629 | no |
| Sobic.006G181800 | 213.7793 | 0.5879 | 0.2983 | 1.9707 | 0.0487639 | 0.2656629 | no |

| Sobic.010G134400 | 202.8303 | 0.6521 | 0.3310 | 1.9703 | 0.0488096 | 0.2658304 | no |
| --- | --- | --- | --- | --- | --- | --- | --- |
| Sobic.007G155300 | 162.6257 | -1.7734 | 0.9002 | -1.9699 | 0.0488486 | 0.2658304 | no |
| Sobic.003G097800 | 432.4022 | -1.3980 | 0.7097 | -1.9699 | 0.0488505 | 0.2658304 | no |
| Sobic.005G079201 | 45.6728 | 1.4339 | 0.7280 | 1.9696 | 0.0488815 | 0.2658304 | no |
| Sobic.010G050900 | 1693.7020 | -0.4989 | 0.2533 | -1.9696 | 0.0488877 | 0.2658304 | no |
| Sobic.010G041000 | 88.3261 | 0.8124 | 0.4125 | 1.9694 | 0.0489066 | 0.2658304 | no |
| Sobic.001G122200 | 101.6180 | 1.4658 | 0.7443 | 1.9694 | 0.0489072 | 0.2658304 | no |
| Sobic.005G051600 | 22.4155 | 1.6900 | 0.8582 | 1.9693 | 0.0489172 | 0.2658304 | no |
| Sobic.004G227400 | 399.5881 | 0.7839 | 0.3981 | 1.9693 | 0.0489189 | 0.2658304 | no |
| Sobic.006G210800 | 3000.7352 | -0.8014 | 0.4069 | -1.9693 | 0.0489207 | 0.2658304 | no |
| Sobic.005G213200 | 51.4298 | 1.6008 | 0.8130 | 1.9691 | 0.0489383 | 0.265858 | no |
| Sobic.003G149800 | 730.4414 | 0.6581 | 0.3343 | 1.9688 | 0.0489716 | 0.2659704 | no |
| Sobic.002G232200 | 2315.6239 | -0.9034 | 0.4589 | -1.9687 | 0.0489863 | 0.2659817 | no |
| Sobic.008G040800 | 173.2056 | -1.2835 | 0.6520 | -1.9686 | 0.0490018 | 0.2659972 | no |
| Sobic.002G103900 | 166.2272 | -1.5832 | 0.8048 | -1.9672 | 0.0491568 | 0.2667699 | no |
| Sobic.008G078966 | 78.1890 | 2.1053 | 1.0706 | 1.9666 | 0.0492323 | 0.2671107 | no |
| Sobic.004G032800 | 351.7673 | 0.7802 | 0.3968 | 1.9663 | 0.0492594 | 0.2671893 | no |
| Sobic.004G132300 | 1369.9133 | 0.6228 | 0.3168 | 1.9662 | 0.0492794 | 0.2672292 | no |
| Sobic.010G106900 | 1789.5048 | 0.4822 | 0.2453 | 1.9660 | 0.0492933 | 0.2672358 | no |
| Sobic.004G072100 | 961.7397 | 0.5569 | 0.2834 | 1.9651 | 0.0494075 | 0.2677743 | no |
| Sobic.003G172850 | 8.9677 | -1.8086 | 0.9204 | -1.9650 | 0.0494181 | 0.2677743 | no |
| Sobic.001G420000 | 618.1760 | -0.7648 | 0.3894 | -1.9641 | 0.0495141 | 0.2682256 | no |
| Sobic.005G019300 | 143.9583 | 0.8581 | 0.4370 | 1.9634 | 0.0495952 | 0.2685389 | no |
| Sobic.003G111000 | 48.6443 | -1.2783 | 0.6511 | -1.9634 | 0.0495974 | 0.2685389 | no |
| Sobic.004G231501 | 1005.1746 | -0.6561 | 0.3342 | -1.9631 | 0.04964 | 0.2687005 | no |
| Sobic.006G272700 | 68.1881 | 1.5789 | 0.8044 | 1.9628 | 0.049668 | 0.2687574 | no |
| Sobic.002G184200 | 18.1226 | -2.9159 | 1.4857 | -1.9627 | 0.0496825 | 0.2687574 | no |
| Sobic.006G263600 | 1774.7687 | -1.2351 | 0.6294 | -1.9624 | 0.0497213 | 0.2687574 | no |
| Sobic.003G001100 | 1116.0685 | -0.6338 | 0.3230 | -1.9624 | 0.0497214 | 0.2687574 | no |
| Sobic.004G332900 | 444.1366 | 0.9993 | 0.5093 | 1.9623 | 0.0497319 | 0.2687574 | no |
| Sobic.002G338500 | 1515.5708 | -1.0826 | 0.5517 | -1.9622 | 0.0497381 | 0.2687574 | no |
| Sobic.004G306800 | 160.9919 | 0.9426 | 0.4804 | 1.9622 | 0.0497397 | 0.2687574 | no |
| Sobic.008G123400 | 37.9511 | 1.5160 | 0.7727 | 1.9620 | 0.0497623 | 0.2688107 | no |
| Sobic.002G125800 | 1013.9208 | 0.5722 | 0.2917 | 1.9616 | 0.0498068 | 0.2689557 | no |
| Sobic.001G504200 | 379.0319 | 0.6693 | 0.3412 | 1.9616 | 0.0498146 | 0.2689557 | no |
| Sobic.002G172000 | 396.9517 | 1.5347 | 0.7827 | 1.9609 | 0.0498898 | 0.2691926 | no |
| Sobic.004G315000 | 7.0697 | 2.4538 | 1.2514 | 1.9608 | 0.0498972 | 0.2691926 | no |
| Sobic.004G199400 | 35.5879 | 1.8638 | 0.9507 | 1.9606 | 0.0499301 | 0.2691926 | no |
| Sobic.010G179800 | 5774.5812 | -1.8263 | 0.9316 | -1.9604 | 0.0499466 | 0.2691926 | no |
| Sobic.007G031800 | 101.1049 | 0.7075 | 0.3609 | 1.9603 | 0.0499553 | 0.2691926 | no |
| Sobic.009G195400 | 349.1065 | -0.4678 | 0.2386 | -1.9603 | 0.0499602 | 0.2691926 | no |
| Sobic.004G209800 | 495.6477 | 0.6690 | 0.3413 | 1.9602 | 0.0499676 | 0.2691926 | no |
| Sobic.002G050400 | 50.1119 | 1.7260 | 0.8805 | 1.9602 | 0.0499706 | 0.2691926 | no |
| Sobic.001G536500 | 9334.8086 | -0.5997 | 0.3059 | -1.9601 | 0.0499799 | 0.2691926 | no |
| Sobic.009G011500 | 170.4131 | 0.5597 | 0.2856 | 1.9600 | 0.0499947 | 0.2691926 | no |
| Sobic.006G131200 | 333.5756 | 0.6441 | 0.3287 | 1.9598 | 0.0500192 | 0.2691926 | no |
| Sobic.001G097400 | 29.0513 | 1.5152 | 0.7732 | 1.9595 | 0.0500509 | 0.2691926 | no |
| Sobic.001G409800 | 509.2860 | -0.6557 | 0.3346 | -1.9595 | 0.0500533 | 0.2691926 | no |
| Sobic.001G178200 | 192.7724 | -0.8501 | 0.4338 | -1.9595 | 0.0500571 | 0.2691926 | no |
| Sobic.003G371600 | 317.9250 | -0.7310 | 0.3731 | -1.9594 | 0.050067 | 0.2691926 | no |
| Sobic.004G107100 | 2349.4151 | 0.9217 | 0.4704 | 1.9594 | 0.0500698 | 0.2691926 | no |
| Sobic.006G019901 | 321.7897 | -0.6885 | 0.3514 | -1.9593 | 0.050081 | 0.2691926 | no |
| Sobic.009G044700 | 710.7546 | -0.9366 | 0.4780 | -1.9592 | 0.0500883 | 0.2691926 | no |
| Sobic.002G200700 | 7727.3010 | 0.8259 | 0.4217 | 1.9586 | 0.0501547 | 0.2694809 | no |
| Sobic.001G214600 | 447.1072 | 0.5349 | 0.2731 | 1.9583 | 0.0501948 | 0.2695988 | no |
| Sobic.010G072700 | 441.2150 | 1.7027 | 0.8695 | 1.9582 | 0.0502022 | 0.2695988 | no |
| Sobic.001G065300 | 23.2181 | -2.2649 | 1.1568 | -1.9580 | 0.0502335 | 0.2696979 | no |

| Sobic.001G118000 | 874.9621 | 0.9218 | 0.4709 | 1.9576 | 0.0502821 | 0.2698661 | no |
| --- | --- | --- | --- | --- | --- | --- | --- |
| Sobic.007G186100 | 78.7286 | -0.7366 | 0.3763 | -1.9575 | 0.0502904 | 0.2698661 | no |
| Sobic.003G122400 | 281.6847 | -0.6646 | 0.3395 | -1.9573 | 0.0503066 | 0.2698844 | no |
| Sobic.010G015400 | 170.7499 | 1.0142 | 0.5182 | 1.9570 | 0.0503419 | 0.2700028 | no |
| Sobic.001G085300 | 1265.7936 | 0.7672 | 0.3921 | 1.9568 | 0.0503662 | 0.2700028 | no |
| Sobic.003G253800 | 360.9382 | 0.7511 | 0.3838 | 1.9568 | 0.0503671 | 0.2700028 | no |
| Sobic.002G239700 | 78.6631 | -1.4861 | 0.7595 | -1.9566 | 0.0503907 | 0.270061 | no |
| Sobic.008G174650 | 13.5731 | -1.8311 | 0.9360 | -1.9563 | 0.0504349 | 0.2701633 | no |
| Sobic.002G396500 | 2175.3936 | 1.0262 | 0.5246 | 1.9562 | 0.0504439 | 0.2701633 | no |
| Sobic.009G209200 | 5589.6161 | -0.5792 | 0.2961 | -1.9561 | 0.0504482 | 0.2701633 | no |
| Sobic.003G119100 | 20.2421 | 1.7919 | 0.9161 | 1.9560 | 0.0504615 | 0.2701658 | no |
| Sobic.003G271201 | 1552.4663 | -0.6982 | 0.3570 | -1.9558 | 0.0504829 | 0.2702056 | no |
| Sobic.006G183200 | 14.2772 | 2.0156 | 1.0306 | 1.9558 | 0.0504946 | 0.2702056 | no |
| Sobic.006G090000 | 387.7795 | -0.7734 | 0.3956 | -1.9550 | 0.0505826 | 0.2705167 | no |
| Sobic.001G418200 | 386.7981 | -1.4378 | 0.7355 | -1.9550 | 0.0505855 | 0.2705167 | no |
| Sobic.001G185200 | 98.4706 | -1.2177 | 0.6229 | -1.9549 | 0.0505912 | 0.2705167 | no |
| Sobic.007G121300 | 8150.0149 | -0.7325 | 0.3747 | -1.9548 | 0.0506054 | 0.270524 | no |
| Sobic.006G153000 | 729.2150 | -0.8670 | 0.4436 | -1.9546 | 0.0506361 | 0.2705746 | no |
| Sobic.006G210200 | 546.0445 | -0.6400 | 0.3274 | -1.9545 | 0.0506405 | 0.2705746 | no |
| Sobic.004G116500 | 115.5489 | 0.8442 | 0.4320 | 1.9544 | 0.0506568 | 0.2705931 | no |
| Sobic.005G169900 | 380.7870 | -0.8831 | 0.4520 | -1.9538 | 0.050727 | 0.2707065 | no |
| Sobic.001G012000 | 137.1307 | 1.3523 | 0.6922 | 1.9538 | 0.0507278 | 0.2707065 | no |
| Sobic.006G186500 | 1137.3943 | -0.7349 | 0.3761 | -1.9538 | 0.0507283 | 0.2707065 | no |
| Sobic.002G301100 | 1600.7414 | 0.5273 | 0.2699 | 1.9537 | 0.050734 | 0.2707065 | no |
| Sobic.003G367500 | 26722.5518 | -0.8877 | 0.4544 | -1.9537 | 0.0507422 | 0.2707065 | no |
| Sobic.004G010151 | 256.9832 | 0.5798 | 0.2968 | 1.9533 | 0.050781 | 0.2707887 | no |
| Sobic.007G008000 | 100.0461 | 0.9564 | 0.4896 | 1.9533 | 0.0507833 | 0.2707887 | no |
| Sobic.010G220500 | 4234.7284 | -0.6238 | 0.3194 | -1.9531 | 0.0508027 | 0.2707939 | no |
| Sobic.002G084900 | 1538.5434 | -0.5902 | 0.3022 | -1.9531 | 0.0508131 | 0.2707939 | no |
| Sobic.006G141800 | 37.8098 | -0.8937 | 0.4577 | -1.9528 | 0.050845 | 0.2707939 | no |
| Sobic.005G133800 | 1838.6612 | 0.7920 | 0.4056 | 1.9528 | 0.0508468 | 0.2707939 | no |
| Sobic.001G260300 | 553.0281 | 0.4611 | 0.2362 | 1.9527 | 0.050854 | 0.2707939 | no |
| Sobic.006G243600 | 7.5398 | 4.1538 | 2.1273 | 1.9526 | 0.0508613 | 0.2707939 | no |
| Sobic.004G026700 | 1066.1668 | -0.8570 | 0.4390 | -1.9519 | 0.0509471 | 0.2711822 | no |
| Sobic.010G084900 | 176.6467 | 1.5524 | 0.7954 | 1.9517 | 0.0509725 | 0.2712476 | no |
| Sobic.009G173800 | 648.7495 | -1.3796 | 0.7069 | -1.9516 | 0.0509897 | 0.2712476 | no |
| Sobic.003G226600 | 60.6918 | -1.0067 | 0.5159 | -1.9513 | 0.0510202 | 0.2712476 | no |
| Sobic.005G223000 | 8.3045 | -2.4856 | 1.2738 | -1.9513 | 0.0510203 | 0.2712476 | no |
| Sobic.002G056800 | 3246.1391 | -0.7931 | 0.4064 | -1.9513 | 0.0510237 | 0.2712476 | no |
| Sobic.004G188400 | 3369.8238 | -0.4884 | 0.2503 | -1.9511 | 0.0510409 | 0.2712704 | no |
| Sobic.001G298100 | 18.8060 | 3.2654 | 1.6738 | 1.9509 | 0.0510736 | 0.2713132 | no |
| Sobic.001G116600 | 142.0963 | 1.1390 | 0.5839 | 1.9508 | 0.0510838 | 0.2713132 | no |
| Sobic.003G268800 | 32.5279 | 1.9874 | 1.0188 | 1.9507 | 0.0510886 | 0.2713132 | no |
| Sobic.005G100300 | 1411.0254 | -1.0228 | 0.5244 | -1.9506 | 0.0511086 | 0.2713132 | no |
| Sobic.009G034800 | 963.8509 | 0.9016 | 0.4622 | 1.9505 | 0.0511133 | 0.2713132 | no |
| Sobic.002G336000 | 701.7419 | -0.6996 | 0.3588 | -1.9497 | 0.0512106 | 0.2717615 | no |
| Sobic.010G049300 | 33.2508 | 1.7166 | 0.8806 | 1.9495 | 0.0512359 | 0.2717827 | no |
| Sobic.003G017300 | 88.9323 | -1.0287 | 0.5277 | -1.9494 | 0.0512467 | 0.2717827 | no |
| Sobic.004G229400 | 7.7932 | 6.1753 | 3.1678 | 1.9494 | 0.0512532 | 0.2717827 | no |
| Sobic.004G013400 | 43.1702 | -1.9433 | 0.9970 | -1.9492 | 0.0512739 | 0.2718128 | no |
| Sobic.005G211500 | 37.4192 | -1.2422 | 0.6373 | -1.9491 | 0.0512847 | 0.2718128 | no |
| Sobic.001G020200 | 20.0554 | 2.1526 | 1.1046 | 1.9488 | 0.0513167 | 0.2718775 | no |
| Sobic.004G306700 | 80.8573 | 0.8286 | 0.4252 | 1.9488 | 0.0513227 | 0.2718775 | no |
| Sobic.001G028500 | 2174.2673 | -0.5386 | 0.2765 | -1.9479 | 0.0514232 | 0.2723413 | no |
| Sobic.007G038700 | 47.0770 | -1.2074 | 0.6199 | -1.9477 | 0.0514458 | 0.2723658 | no |
| Sobic.006G140900 | 221.5717 | -0.7971 | 0.4093 | -1.9477 | 0.0514536 | 0.2723658 | no |
| Sobic.009G137500 | 9.0545 | 2.5962 | 1.3331 | 1.9475 | 0.0514727 | 0.2723984 | no |

| Sobic.003G235300 | 510.9022 | 0.6525 | 0.3351 | 1.9472 | 0.0515079 | 0.2725164 | no |
| --- | --- | --- | --- | --- | --- | --- | --- |
| Sobic.001G308800 | 1322.9321 | -0.6741 | 0.3462 | -1.9471 | 0.0515237 | 0.2725314 | no |
| Sobic.010G101200 | 17007.5266 | -0.8960 | 0.4602 | -1.9469 | 0.0515495 | 0.2725484 | no |
| Sobic.001G358300 | 785.5332 | 0.8340 | 0.4284 | 1.9468 | 0.0515527 | 0.2725484 | no |
| Sobic.002G345400 | 7.4163 | -2.4480 | 1.2576 | -1.9466 | 0.0515827 | 0.2725754 | no |
| Sobic.003G012000 | 208.7260 | 0.5602 | 0.2878 | 1.9466 | 0.0515837 | 0.2725754 | no |
| Sobic.001G057200 | 691.8762 | 0.7278 | 0.3739 | 1.9463 | 0.0516147 | 0.2726176 | no |
| Sobic.010G186700 | 1474.6993 | 0.7720 | 0.3967 | 1.9463 | 0.0516219 | 0.2726176 | no |
| Sobic.001G307200 | 614.2623 | 1.0417 | 0.5353 | 1.9462 | 0.0516304 | 0.2726176 | no |
| Sobic.001G336900 | 5.7722 | -2.2659 | 1.1646 | -1.9457 | 0.0516933 | 0.2728813 | no |
| Sobic.001G259700 | 128.7842 | -2.5411 | 1.3062 | -1.9453 | 0.051733 | 0.2730224 | no |
| Sobic.001G050500 | 398.3230 | 0.9184 | 0.4722 | 1.9450 | 0.0517748 | 0.2731747 | no |
| Sobic.002G275200 | 3890.8802 | 0.7875 | 0.4050 | 1.9447 | 0.0518168 | 0.2732247 | no |
| Sobic.003G285600 | 1752.1332 | -0.8719 | 0.4484 | -1.9446 | 0.051822 | 0.2732247 | no |
| Sobic.010G250400 | 475.6877 | 1.8726 | 0.9630 | 1.9446 | 0.0518269 | 0.2732247 | no |
| Sobic.001G475500 | 54.6991 | 1.2352 | 0.6352 | 1.9445 | 0.0518361 | 0.2732247 | no |
| Sobic.003G188600 | 107.7571 | 1.0574 | 0.5439 | 1.9443 | 0.0518631 | 0.2732847 | no |
| Sobic.001G082200 | 556.7448 | -1.0390 | 0.5344 | -1.9442 | 0.0518734 | 0.2732847 | no |
| Sobic.003G092700 | 459.4497 | 0.9699 | 0.4990 | 1.9436 | 0.0519417 | 0.2735758 | no |
| Sobic.009G077600 | 764.1966 | 0.6878 | 0.3539 | 1.9434 | 0.0519707 | 0.2736607 | no |
| Sobic.001G137000 | 1022.3673 | 0.6652 | 0.3423 | 1.9431 | 0.0520059 | 0.2737194 | no |
| Sobic.004G119000 | 638.2062 | -0.4700 | 0.2419 | -1.9431 | 0.0520079 | 0.2737194 | no |
| Sobic.007G196900 | 492.8812 | 0.6690 | 0.3444 | 1.9428 | 0.0520435 | 0.2738386 | no |
| Sobic.004G094100 | 506.9472 | -0.7686 | 0.3956 | -1.9426 | 0.0520667 | 0.2738835 | no |
| Sobic.001G333100 | 1898.4129 | 0.6107 | 0.3144 | 1.9423 | 0.0520949 | 0.2738835 | no |
| Sobic.002G411700 | 53.6321 | 1.7282 | 0.8898 | 1.9423 | 0.0521032 | 0.2738835 | no |
| Sobic.010G279100 | 1363.5951 | -0.8367 | 0.4308 | -1.9423 | 0.052104 | 0.2738835 | no |
| Sobic.001G334700 | 572.6802 | 0.7189 | 0.3703 | 1.9416 | 0.0521829 | 0.274136 | no |
| Sobic.005G002500 | 6.0852 | -2.1542 | 1.1095 | -1.9415 | 0.0521951 | 0.274136 | no |
| Sobic.002G406300 | 683.3778 | 0.5583 | 0.2876 | 1.9413 | 0.0522179 | 0.274136 | no |
| Sobic.009G028200 | 904.0486 | -1.1719 | 0.6038 | -1.9410 | 0.0522526 | 0.274136 | no |
| Sobic.001G205500 | 102.7962 | 1.0281 | 0.5297 | 1.9410 | 0.0522542 | 0.274136 | no |
| Sobic.004G011700 | 4259.2098 | 0.7141 | 0.3679 | 1.9410 | 0.052259 | 0.274136 | no |
| Sobic.008G021800 | 963.9591 | 1.6990 | 0.8754 | 1.9410 | 0.0522606 | 0.274136 | no |
| Sobic.004G132600 | 249.2566 | -1.0284 | 0.5298 | -1.9408 | 0.0522776 | 0.274136 | no |
| Sobic.002G161900 | 544.1872 | 0.8327 | 0.4291 | 1.9408 | 0.0522791 | 0.274136 | no |
| Sobic.K043400 | 350.2033 | 0.9854 | 0.5077 | 1.9408 | 0.052282 | 0.274136 | no |
| Sobic.005G200200 | 2713.6320 | -1.0762 | 0.5546 | -1.9405 | 0.0523141 | 0.2742361 | no |
| Sobic.009G148600 | 248.1999 | -0.8320 | 0.4288 | -1.9402 | 0.0523544 | 0.2742481 | no |
| Sobic.007G002600 | 471.2845 | 0.5276 | 0.2720 | 1.9401 | 0.0523623 | 0.2742481 | no |
| Sobic.002G370700 | 12146.0228 | -0.6853 | 0.3533 | -1.9400 | 0.0523782 | 0.2742481 | no |
| Sobic.006G208050 | 302.8424 | 0.7384 | 0.3806 | 1.9400 | 0.0523788 | 0.2742481 | no |
| Sobic.002G370200 | 2479.8735 | 0.4085 | 0.2106 | 1.9400 | 0.0523814 | 0.2742481 | no |
| Sobic.006G115466 | 353.8294 | 0.8710 | 0.4490 | 1.9398 | 0.0524049 | 0.2743032 | no |
| Sobic.001G481100 | 41.6283 | -1.2134 | 0.6256 | -1.9396 | 0.0524271 | 0.2743513 | no |
| Sobic.010G026925 | 8594.2203 | -1.3390 | 0.6905 | -1.9392 | 0.0524821 | 0.2745707 | no |
| Sobic.010G128700 | 129.8239 | -1.5089 | 0.7783 | -1.9388 | 0.0525229 | 0.2746044 | no |
| Sobic.006G065800 | 668.9966 | 0.4992 | 0.2575 | 1.9388 | 0.0525272 | 0.2746044 | no |
| Sobic.005G038900 | 426.7057 | 0.8804 | 0.4541 | 1.9388 | 0.0525276 | 0.2746044 | no |
| Sobic.001G313100 | 460.4742 | -0.8646 | 0.4460 | -1.9385 | 0.0525629 | 0.2747212 | no |
| Sobic.003G321400 | 5.8436 | 3.6715 | 1.8942 | 1.9383 | 0.0525918 | 0.2748039 | no |
| Sobic.002G242600 | 204.1006 | -1.0902 | 0.5625 | -1.9381 | 0.0526065 | 0.2748128 | no |
| Sobic.004G152300 | 8.1774 | 1.8641 | 0.9619 | 1.9380 | 0.0526251 | 0.2748416 | no |
| Sobic.005G093200 | 45.4453 | -2.0043 | 1.0343 | -1.9378 | 0.0526426 | 0.2748651 | no |
| Sobic.001G160200 | 142.1260 | -1.2862 | 0.6640 | -1.9371 | 0.0527276 | 0.2752407 | no |
| Sobic.001G097100 | 5296.6503 | -0.6363 | 0.3285 | -1.9368 | 0.0527732 | 0.2754104 | no |
| Sobic.004G071000 | 168.6617 | 1.2825 | 0.6623 | 1.9365 | 0.0528117 | 0.2754873 | no |

| Sobic.008G111100 | 2774.2549 | -1.0278 | 0.5308 | -1.9364 | 0.052814 | 0.2754873 | no |
| --- | --- | --- | --- | --- | --- | --- | --- |
| Sobic.009G127800 | 23.5229 | 1.3119 | 0.6776 | 1.9362 | 0.0528412 | 0.2755608 | no |
| Sobic.003G126300 | 1258.9136 | 0.7076 | 0.3655 | 1.9360 | 0.0528684 | 0.2756346 | no |
| Sobic.004G245900 | 83.4396 | 1.2362 | 0.6386 | 1.9358 | 0.0528978 | 0.2757199 | no |
| Sobic.010G187100 | 3085.6746 | 1.0507 | 0.5429 | 1.9353 | 0.0529509 | 0.2759281 | no |
| Sobic.004G105200 | 701.5099 | -0.8520 | 0.4403 | -1.9351 | 0.0529743 | 0.2759819 | no |
| Sobic.004G058400 | 705.2422 | -0.5380 | 0.2781 | -1.9349 | 0.0530055 | 0.2760762 | no |
| Sobic.007G181300 | 1413.9567 | -0.6399 | 0.3308 | -1.9346 | 0.0530415 | 0.2761958 | no |
| Sobic.009G217600 | 165.3445 | -1.7385 | 0.8987 | -1.9344 | 0.0530664 | 0.2762569 | no |
| Sobic.003G219800 | 1177.0890 | 0.9392 | 0.4855 | 1.9342 | 0.0530867 | 0.2762664 | no |
| Sobic.006G155800 | 31.8326 | 2.2722 | 1.1749 | 1.9339 | 0.0531232 | 0.2762664 | no |
| Sobic.002G357400 | 60.3979 | 1.2674 | 0.6554 | 1.9339 | 0.0531264 | 0.2762664 | no |
| Sobic.001G140000 | 58.8670 | 0.9755 | 0.5045 | 1.9337 | 0.053146 | 0.2762664 | no |
| Sobic.004G238801 | 7.7832 | -2.0276 | 1.0486 | -1.9337 | 0.0531475 | 0.2762664 | no |
| Sobic.003G432900 | 47.8208 | 1.0159 | 0.5254 | 1.9337 | 0.0531527 | 0.2762664 | no |
| Sobic.010G257100 | 1340.6612 | 0.6168 | 0.3190 | 1.9336 | 0.0531626 | 0.2762664 | no |
| Sobic.001G295700 | 1010.1862 | -2.0400 | 1.0551 | -1.9335 | 0.053173 | 0.2762664 | no |
| Sobic.009G186600 | 39.9515 | 0.9850 | 0.5096 | 1.9331 | 0.0532196 | 0.2764406 | no |
| Sobic.002G117100 | 15.3046 | 3.3816 | 1.7508 | 1.9314 | 0.0534304 | 0.2774288 | no |
| Sobic.001G188000 | 162.8043 | 0.9840 | 0.5095 | 1.9313 | 0.0534516 | 0.2774288 | no |
| Sobic.009G165400 | 1190.7740 | 0.6745 | 0.3493 | 1.9312 | 0.0534613 | 0.2774288 | no |
| Sobic.001G151800 | 123.7453 | 1.0745 | 0.5564 | 1.9310 | 0.0534818 | 0.2774288 | no |
| Sobic.001G408200 | 1155.3602 | 0.6125 | 0.3172 | 1.9310 | 0.0534869 | 0.2774288 | no |
| Sobic.006G069100 | 10.6448 | 2.2571 | 1.1690 | 1.9309 | 0.0535009 | 0.2774288 | no |
| Sobic.008G124700 | 792.6824 | -0.5616 | 0.2909 | -1.9308 | 0.053502 | 0.2774288 | no |
| Sobic.004G219300 | 524.5579 | -0.7747 | 0.4013 | -1.9303 | 0.0535679 | 0.2777025 | no |
| Sobic.003G182800 | 225.7952 | 1.0388 | 0.5384 | 1.9295 | 0.0536643 | 0.2781336 | no |
| Sobic.010G047800 | 39.7250 | 1.4075 | 0.7296 | 1.9292 | 0.0537072 | 0.2782876 | no |
| Sobic.005G169800 | 1020.8799 | -0.8557 | 0.4436 | -1.9290 | 0.0537353 | 0.278365 | no |
| Sobic.005G050900 | 5.8593 | -6.2440 | 3.2376 | -1.9286 | 0.0537831 | 0.278544 | no |
| Sobic.003G389000 | 707.6149 | 0.5656 | 0.2933 | 1.9284 | 0.0538032 | 0.2785801 | no |
| Sobic.006G232000 | 452.9710 | 0.8938 | 0.4636 | 1.9280 | 0.0538506 | 0.278757 | no |
| Sobic.001G253300 | 696.7958 | 0.6152 | 0.3192 | 1.9278 | 0.0538856 | 0.2788695 | no |
| Sobic.008G102000 | 6559.7461 | -0.9633 | 0.4999 | -1.9270 | 0.0539796 | 0.2792873 | no |
| Sobic.003G221600 | 744.3851 | -0.5804 | 0.3012 | -1.9266 | 0.0540273 | 0.2794368 | no |
| Sobic.005G202000 | 1030.6096 | 0.9693 | 0.5031 | 1.9266 | 0.054035 | 0.2794368 | no |
| Sobic.003G247400 | 618.4828 | -0.8251 | 0.4283 | -1.9264 | 0.0540591 | 0.2794597 | no |
| Sobic.007G201100 | 865.2593 | 1.2093 | 0.6278 | 1.9263 | 0.0540663 | 0.2794597 | no |
| Sobic.002G304000 | 2213.7468 | 0.9093 | 0.4721 | 1.9262 | 0.0540791 | 0.2794597 | no |
| Sobic.001G488100 | 314.8745 | 0.5790 | 0.3006 | 1.9260 | 0.0541086 | 0.2795432 | no |
| Sobic.001G415700 | 444.4072 | 1.1709 | 0.6080 | 1.9258 | 0.0541351 | 0.2795665 | no |
| Sobic.003G228600 | 5.1983 | 3.4936 | 1.8142 | 1.9257 | 0.0541396 | 0.2795665 | no |
| Sobic.007G190100 | 1500.9079 | -1.1610 | 0.6029 | -1.9256 | 0.0541595 | 0.279601 | no |
| Sobic.003G437600 | 8992.9713 | -1.1127 | 0.5779 | -1.9253 | 0.0541895 | 0.2796871 | no |
| Sobic.007G154300 | 10.0813 | -2.3172 | 1.2038 | -1.9250 | 0.0542351 | 0.2798543 | no |
| Sobic.002G238300 | 344.0352 | -1.0532 | 0.5472 | -1.9248 | 0.054253 | 0.2798778 | no |
| Sobic.004G002100 | 1646.1383 | 0.6379 | 0.3315 | 1.9244 | 0.0542989 | 0.28002 | no |
| Sobic.003G155000 | 9.6836 | 1.9011 | 0.9880 | 1.9243 | 0.0543187 | 0.28002 | no |
| Sobic.001G034900 | 27.1270 | 2.3398 | 1.2160 | 1.9243 | 0.0543204 | 0.28002 | no |
| Sobic.003G377900 | 1093.2638 | 0.6797 | 0.3532 | 1.9241 | 0.0543383 | 0.2800441 | no |
| Sobic.010G165400 | 7.7046 | -2.5137 | 1.3066 | -1.9239 | 0.0543691 | 0.2800922 | no |
| Sobic.002G020400 | 780.5849 | -0.6339 | 0.3295 | -1.9238 | 0.0543742 | 0.2800922 | no |
| Sobic.007G072600 | 161.0380 | 0.7371 | 0.3832 | 1.9236 | 0.0544082 | 0.280199 | no |
| Sobic.004G304900 | 219.9081 | 0.8598 | 0.4470 | 1.9234 | 0.0544264 | 0.2802243 | no |
| Sobic.008G131800 | 1037.5073 | 0.5900 | 0.3068 | 1.9232 | 0.054449 | 0.2802722 | no |
| Sobic.003G346200 | 127.7339 | 1.3250 | 0.6890 | 1.9231 | 0.0544715 | 0.2803197 | no |
| Sobic.001G212350 | 67.6871 | 1.5827 | 0.8231 | 1.9229 | 0.0544915 | 0.2803539 | no |

| Sobic.004G333100 | 840.0566 | 1.1962 | 0.6222 | 1.9225 | 0.0545444 | 0.28049 | no |
| --- | --- | --- | --- | --- | --- | --- | --- |
| Sobic.001G482100 | 1526.0657 | -0.9732 | 0.5062 | -1.9225 | 0.0545445 | 0.28049 | no |
| Sobic.005G167300 | 2796.4238 | -0.6882 | 0.3580 | -1.9220 | 0.0546051 | 0.280733 | no |
| Sobic.003G081900 | 60.0640 | 2.1723 | 1.1304 | 1.9217 | 0.0546437 | 0.2808631 | no |
| Sobic.001G395100 | 1113.5280 | 0.5160 | 0.2686 | 1.9211 | 0.0547129 | 0.2811196 | no |
| Sobic.004G184800 | 76.8149 | -1.4753 | 0.7679 | -1.9211 | 0.0547203 | 0.2811196 | no |
| Sobic.001G230700 | 580.1229 | 0.7629 | 0.3972 | 1.9209 | 0.054739 | 0.2811196 | no |
| Sobic.010G241500 | 305.9480 | 0.9129 | 0.4753 | 1.9209 | 0.0547469 | 0.2811196 | no |
| Sobic.001G528400 | 814.1751 | -0.8526 | 0.4439 | -1.9206 | 0.0547786 | 0.2812137 | no |
| Sobic.003G428400 | 2349.5631 | 0.6683 | 0.3481 | 1.9200 | 0.0548583 | 0.2815545 | no |
| Sobic.003G367000 | 103.1575 | -1.5008 | 0.7818 | -1.9198 | 0.0548827 | 0.2816113 | no |
| Sobic.001G225900 | 20.0795 | -2.1827 | 1.1372 | -1.9195 | 0.0549249 | 0.2816323 | no |
| Sobic.006G024400 | 648.1420 | 1.0310 | 0.5372 | 1.9193 | 0.0549524 | 0.2816323 | no |
| Sobic.002G387200 | 417.7156 | 0.9352 | 0.4873 | 1.9192 | 0.0549644 | 0.2816323 | no |
| Sobic.006G195400 | 132.0398 | -0.7463 | 0.3889 | -1.9191 | 0.0549685 | 0.2816323 | no |
| Sobic.002G299700 | 834.2510 | 0.5650 | 0.2944 | 1.9190 | 0.0549795 | 0.2816323 | no |
| Sobic.001G206200 | 16103.6750 | -1.4876 | 0.7752 | -1.9190 | 0.0549797 | 0.2816323 | no |
| Sobic.008G009100 | 36.6733 | 1.6921 | 0.8817 | 1.9190 | 0.0549803 | 0.2816323 | no |
| Sobic.003G038300 | 251.7704 | 0.6180 | 0.3221 | 1.9188 | 0.0550119 | 0.2817257 | no |
| Sobic.003G149300 | 728.8225 | -0.7726 | 0.4027 | -1.9186 | 0.0550369 | 0.2817853 | no |
| Sobic.001G529600 | 2743.7622 | 0.7751 | 0.4041 | 1.9183 | 0.0550715 | 0.2818611 | no |
| Sobic.007G026600 | 22.8782 | 1.3409 | 0.6990 | 1.9183 | 0.0550785 | 0.2818611 | no |
| Sobic.003G425600 | 171.3777 | -1.5409 | 0.8034 | -1.9179 | 0.0551188 | 0.2819989 | no |
| Sobic.009G092400 | 1641.1306 | 0.5551 | 0.2895 | 1.9175 | 0.0551712 | 0.2821357 | no |
| Sobic.001G356100 | 133.2347 | 0.7856 | 0.4097 | 1.9175 | 0.0551722 | 0.2821357 | no |
| Sobic.010G148932 | 848.0703 | 0.6418 | 0.3348 | 1.9170 | 0.0552349 | 0.2823752 | no |
| Sobic.002G110600 | 360.7494 | -0.7260 | 0.3787 | -1.9169 | 0.0552539 | 0.2823752 | no |
| Sobic.002G035200 | 1650.1112 | 1.0314 | 0.5381 | 1.9168 | 0.05526 | 0.2823752 | no |
| Sobic.003G439200 | 194.3587 | -1.0722 | 0.5594 | -1.9167 | 0.0552726 | 0.2823752 | no |
| Sobic.003G435900 | 547.1236 | -0.9512 | 0.4963 | -1.9166 | 0.0552901 | 0.2823961 | no |
| Sobic.001G455500 | 1265.1470 | -0.7029 | 0.3669 | -1.9160 | 0.0553651 | 0.2826474 | no |
| Sobic.010G005600 | 1432.7072 | -0.6903 | 0.3603 | -1.9160 | 0.0553661 | 0.2826474 | no |
| Sobic.001G056400 | 5.5046 | 3.7228 | 1.9434 | 1.9156 | 0.0554186 | 0.2828466 | no |
| Sobic.004G135400 | 2699.0232 | 0.5954 | 0.3108 | 1.9154 | 0.055441 | 0.282847 | no |
| Sobic.004G240500 | 333.7566 | 1.1241 | 0.5869 | 1.9152 | 0.0554672 | 0.282847 | no |
| Sobic.008G105900 | 449.7928 | -1.3087 | 0.6834 | -1.9151 | 0.0554757 | 0.282847 | no |
| Sobic.002G081900 | 117.2866 | 0.8744 | 0.4566 | 1.9151 | 0.0554816 | 0.282847 | no |
| Sobic.001G124600 | 952.9998 | -0.8306 | 0.4337 | -1.9151 | 0.0554857 | 0.282847 | no |
| Sobic.006G193200 | 9478.7029 | -1.1121 | 0.5808 | -1.9147 | 0.0555336 | 0.2830225 | no |
| Sobic.004G156200 | 49.9545 | 1.0727 | 0.5604 | 1.9141 | 0.0556017 | 0.2831646 | no |
| Sobic.006G082000 | 682.5765 | -1.0373 | 0.5419 | -1.9141 | 0.0556022 | 0.2831646 | no |
| Sobic.003G168425 | 17.6539 | -1.4706 | 0.7683 | -1.9141 | 0.0556126 | 0.2831646 | no |
| Sobic.009G045600 | 237.1583 | -1.1204 | 0.5854 | -1.9140 | 0.0556151 | 0.2831646 | no |
| Sobic.007G044800 | 5.9158 | 4.7769 | 2.4967 | 1.9133 | 0.055708 | 0.2834339 | no |
| Sobic.006G254700 | 273.1580 | -0.5279 | 0.2759 | -1.9133 | 0.0557113 | 0.2834339 | no |
| Sobic.005G110532 | 183.6034 | -1.0676 | 0.5580 | -1.9132 | 0.0557191 | 0.2834339 | no |
| Sobic.003G076200 | 2374.2978 | 0.9541 | 0.4987 | 1.9132 | 0.0557273 | 0.2834339 | no |
| Sobic.003G353000 | 63.5040 | -1.0584 | 0.5532 | -1.9131 | 0.0557352 | 0.2834339 | no |
| Sobic.001G079200 | 2336.3015 | 0.6375 | 0.3333 | 1.9129 | 0.0557556 | 0.2834679 | no |
| Sobic.003G196800 | 5341.3301 | -0.6163 | 0.3222 | -1.9128 | 0.0557688 | 0.2834679 | no |
| Sobic.010G084700 | 247.2241 | 1.3994 | 0.7317 | 1.9127 | 0.0557898 | 0.2835063 | no |
| Sobic.001G231400 | 282.2981 | 1.0942 | 0.5722 | 1.9125 | 0.0558181 | 0.2835581 | no |
| Sobic.010G143300 | 59.0820 | 1.0330 | 0.5402 | 1.9124 | 0.0558269 | 0.2835581 | no |
| Sobic.004G078000 | 978.4803 | 0.6903 | 0.3610 | 1.9121 | 0.0558623 | 0.2836696 | no |
| Sobic.002G335200 | 109.0345 | -1.2638 | 0.6610 | -1.9118 | 0.0558998 | 0.283724 | no |
| Sobic.001G378150 | 66.8465 | 1.3198 | 0.6904 | 1.9117 | 0.0559126 | 0.283724 | no |
| Sobic.003G409300 | 290.1906 | -0.6610 | 0.3458 | -1.9117 | 0.0559134 | 0.283724 | no |

| Sobic.001G454000 | 295.0345 | 0.8937 | 0.4675 | 1.9115 | 0.0559351 | 0.2837244 | no |
| --- | --- | --- | --- | --- | --- | --- | --- |
| Sobic.003G091850 | 230.1440 | -1.6227 | 0.8489 | -1.9115 | 0.055945 | 0.2837244 | no |
| Sobic.010G056300 | 343.7038 | -0.5968 | 0.3122 | -1.9114 | 0.0559538 | 0.2837244 | no |
| Sobic.002G430300 | 840.6502 | 0.6058 | 0.3170 | 1.9111 | 0.0559977 | 0.2838785 | no |
| Sobic.004G063200 | 7280.7474 | -0.6762 | 0.3539 | -1.9108 | 0.0560277 | 0.2839623 | no |
| Sobic.001G534800 | 2622.1382 | 0.4042 | 0.2116 | 1.9106 | 0.0560535 | 0.2839732 | no |
| Sobic.006G134300 | 53.7974 | -1.2027 | 0.6295 | -1.9105 | 0.0560659 | 0.2839732 | no |
| Sobic.004G276133 | 7.3570 | 1.8898 | 0.9892 | 1.9104 | 0.0560778 | 0.2839732 | no |
| Sobic.001G298600 | 1235.6347 | -0.4859 | 0.2544 | -1.9102 | 0.0561094 | 0.2839732 | no |
| Sobic.008G189200 | 1156.9300 | 1.1896 | 0.6228 | 1.9102 | 0.0561124 | 0.2839732 | no |
| Sobic.007G172200 | 578.6068 | 0.6678 | 0.3496 | 1.9101 | 0.0561214 | 0.2839732 | no |
| Sobic.002G338300 | 1077.9164 | -1.4138 | 0.7402 | -1.9101 | 0.0561245 | 0.2839732 | no |
| Sobic.001G291900 | 3358.2749 | -0.4911 | 0.2571 | -1.9100 | 0.0561375 | 0.2839732 | no |
| Sobic.010G212100 | 1407.0402 | -0.6277 | 0.3287 | -1.9098 | 0.056155 | 0.2839937 | no |
| Sobic.003G160500 | 327.5432 | 0.9301 | 0.4872 | 1.9092 | 0.0562357 | 0.284247 | no |
| Sobic.010G139500 | 566.2459 | 0.7349 | 0.3849 | 1.9092 | 0.0562386 | 0.284247 | no |
| Sobic.004G336300 | 541.8869 | 1.0074 | 0.5277 | 1.9090 | 0.0562593 | 0.284247 | no |
| Sobic.001G211500 | 265.7245 | 0.9546 | 0.5001 | 1.9088 | 0.056288 | 0.284247 | no |
| Sobic.010G066700 | 16.7938 | 2.5390 | 1.3302 | 1.9088 | 0.0562934 | 0.284247 | no |
| Sobic.003G302300 | 244.6637 | 0.7160 | 0.3752 | 1.9086 | 0.0563081 | 0.284247 | no |
| Sobic.003G384300 | 917.5707 | 0.9910 | 0.5192 | 1.9086 | 0.0563124 | 0.284247 | no |
| Sobic.003G144300 | 616.6566 | 0.6920 | 0.3626 | 1.9085 | 0.0563255 | 0.284247 | no |
| Sobic.006G113800 | 350.8216 | 1.2660 | 0.6634 | 1.9085 | 0.0563264 | 0.284247 | no |
| Sobic.008G024000 | 24.5237 | 1.0878 | 0.5700 | 1.9084 | 0.0563455 | 0.2842752 | no |
| Sobic.007G156101 | 104.6556 | -1.3845 | 0.7256 | -1.9081 | 0.0563743 | 0.2843524 | no |
| Sobic.006G274700 | 3416.0779 | -0.7468 | 0.3915 | -1.9077 | 0.0564287 | 0.284519 | no |
| Sobic.003G397600 | ######### | -0.8102 | 0.4247 | -1.9077 | 0.0564343 | 0.284519 | no |
| Sobic.006G071362 | 43.7265 | 1.6058 | 0.8418 | 1.9075 | 0.0564612 | 0.2845721 | no |
| Sobic.003G175500 | 5.0372 | 2.4323 | 1.2752 | 1.9073 | 0.0564763 | 0.2845721 | no |
| Sobic.005G154500 | 488.0472 | 0.4755 | 0.2493 | 1.9072 | 0.0564971 | 0.2845721 | no |
| Sobic.010G263700 | 15.7125 | -1.5908 | 0.8342 | -1.9071 | 0.0565122 | 0.2845721 | no |
| Sobic.003G241100 | 323.1339 | 0.5585 | 0.2928 | 1.9070 | 0.0565142 | 0.2845721 | no |
| Sobic.002G252300 | 2824.4006 | 0.6065 | 0.3180 | 1.9070 | 0.0565258 | 0.2845721 | no |
| Sobic.003G306600 | 8392.3710 | -0.9063 | 0.4754 | -1.9065 | 0.0565855 | 0.2848048 | no |
| Sobic.010G242000 | 15.0702 | 1.4438 | 0.7575 | 1.9059 | 0.05666 | 0.2850308 | no |
| Sobic.003G394900 | 29.3174 | 1.8417 | 0.9663 | 1.9059 | 0.056666 | 0.2850308 | no |
| Sobic.007G218600 | 603.9676 | 0.6129 | 0.3216 | 1.9058 | 0.056671 | 0.2850308 | no |
| Sobic.007G078200 | 65.6428 | 0.8754 | 0.4593 | 1.9057 | 0.0566855 | 0.285036 | no |
| Sobic.002G198900 | 16.6096 | 1.6531 | 0.8676 | 1.9054 | 0.0567225 | 0.2850968 | no |
| Sobic.002G149000 | 33.4768 | 1.1241 | 0.5900 | 1.9053 | 0.0567377 | 0.2850968 | no |
| Sobic.003G198800 | 2945.9800 | 0.7930 | 0.4163 | 1.9051 | 0.056768 | 0.2850968 | no |
| Sobic.003G160200 | 16371.4604 | 0.5654 | 0.2968 | 1.9050 | 0.0567851 | 0.2850968 | no |
| Sobic.003G295300 | 857.4747 | -3.3388 | 1.7527 | -1.9049 | 0.0567886 | 0.2850968 | no |
| Sobic.009G119400 | 53.8812 | -1.5905 | 0.8350 | -1.9049 | 0.05679 | 0.2850968 | no |
| Sobic.009G222400 | 12.6918 | -2.0265 | 1.0639 | -1.9048 | 0.056805 | 0.2850968 | no |
| Sobic.001G351400 | 19491.8537 | -0.9447 | 0.4959 | -1.9048 | 0.0568058 | 0.2850968 | no |
| Sobic.004G141600 | 21.0331 | 1.4866 | 0.7806 | 1.9044 | 0.0568596 | 0.2852518 | no |
| Sobic.003G233400 | 489.6096 | 0.6868 | 0.3607 | 1.9042 | 0.0568794 | 0.2852518 | no |
| Sobic.001G017000 | 1051.3270 | -1.3605 | 0.7145 | -1.9042 | 0.0568844 | 0.2852518 | no |
| Sobic.009G237500 | 526.6402 | -0.8205 | 0.4309 | -1.9041 | 0.0568908 | 0.2852518 | no |
| Sobic.009G133000 | 15.3312 | -1.6437 | 0.8633 | -1.9040 | 0.0569119 | 0.2852802 | no |
| Sobic.006G265000 | 877.6351 | 0.6976 | 0.3664 | 1.9039 | 0.0569235 | 0.2852802 | no |
| Sobic.010G186200 | 849.0653 | -0.6043 | 0.3175 | -1.9033 | 0.0570067 | 0.2856295 | no |
| Sobic.001G219800 | 707.0632 | 0.6279 | 0.3299 | 1.9032 | 0.0570207 | 0.2856319 | no |
| Sobic.005G021200 | 95.4450 | 0.6935 | 0.3644 | 1.9030 | 0.0570454 | 0.2856812 | no |
| Sobic.006G101500 | 17907.3445 | -0.7719 | 0.4057 | -1.9028 | 0.0570663 | 0.2856812 | no |
| Sobic.001G408300 | 365.4969 | 0.7347 | 0.3861 | 1.9028 | 0.0570712 | 0.2856812 | no |

| Sobic.003G168300 | 5.7371 | -2.2604 | 1.1881 | -1.9025 | 0.0571038 | 0.2857763 | no |
| --- | --- | --- | --- | --- | --- | --- | --- |
| Sobic.004G238500 | 11451.6139 | -1.4818 | 0.7791 | -1.9021 | 0.0571618 | 0.285999 | no |
| Sobic.001G361200 | 53.2222 | 0.8552 | 0.4497 | 1.9019 | 0.0571859 | 0.2860515 | no |
| Sobic.008G018400 | 108.7682 | -1.9954 | 1.0493 | -1.9017 | 0.057215 | 0.2861289 | no |
| Sobic.007G227700 | 2749.5587 | -0.5825 | 0.3063 | -1.9015 | 0.0572401 | 0.2861289 | no |
| Sobic.001G100700 | 56.2242 | 1.9204 | 1.0100 | 1.9015 | 0.0572426 | 0.2861289 | no |
| Sobic.003G087600 | 7.9146 | 2.3640 | 1.2434 | 1.9013 | 0.0572667 | 0.2861289 | no |
| Sobic.010G061800 | 2366.3308 | 0.6640 | 0.3492 | 1.9013 | 0.0572692 | 0.2861289 | no |
| Sobic.001G260800 | 4775.5710 | -0.9503 | 0.5000 | -1.9008 | 0.0573276 | 0.2863234 | no |
| Sobic.003G284100 | 187.9111 | -0.7895 | 0.4153 | -1.9007 | 0.0573353 | 0.2863234 | no |
| Sobic.003G320800 | 135.6704 | 0.9337 | 0.4913 | 1.9005 | 0.0573731 | 0.286376 | no |
| Sobic.002G261600 | 3558.3563 | 0.8404 | 0.4422 | 1.9004 | 0.0573755 | 0.286376 | no |
| Sobic.002G062600 | 31.2111 | 1.4088 | 0.7413 | 1.9004 | 0.0573865 | 0.286376 | no |
| Sobic.001G005600 | 1930.5193 | 0.5607 | 0.2951 | 1.8999 | 0.0574521 | 0.2865683 | no |
| Sobic.003G398900 | 16.4983 | 1.8507 | 0.9741 | 1.8999 | 0.0574523 | 0.2865683 | no |
| Sobic.009G127000 | 331.7497 | 0.6808 | 0.3584 | 1.8997 | 0.0574683 | 0.2865804 | no |
| Sobic.002G274700 | 322.5797 | 0.7344 | 0.3867 | 1.8994 | 0.0575117 | 0.2867291 | no |
| Sobic.009G053600 | 401.6109 | -1.4873 | 0.7832 | -1.8990 | 0.0575633 | 0.2868965 | no |
| Sobic.003G031900 | 82.4240 | -1.7133 | 0.9023 | -1.8989 | 0.0575725 | 0.2868965 | no |
| Sobic.004G170200 | 880.4421 | 0.7587 | 0.3996 | 1.8987 | 0.0576026 | 0.2869318 | no |
| Sobic.003G213000 | 437.5613 | 1.5933 | 0.8392 | 1.8987 | 0.0576068 | 0.2869318 | no |
| Sobic.003G088400 | 206.5451 | 1.8117 | 0.9543 | 1.8984 | 0.0576484 | 0.2870309 | no |
| Sobic.002G379866 | 707.2402 | 0.7874 | 0.4148 | 1.8983 | 0.0576539 | 0.2870309 | no |
| Sobic.005G060300 | 851.2293 | -0.7535 | 0.3970 | -1.8981 | 0.057686 | 0.2871232 | no |
| Sobic.005G122900 | 622.7392 | -0.6573 | 0.3464 | -1.8977 | 0.0577377 | 0.2872838 | no |
| Sobic.009G241200 | 885.6956 | 0.8756 | 0.4614 | 1.8976 | 0.0577456 | 0.2872838 | no |
| Sobic.003G401100 | 876.2137 | 0.5194 | 0.2738 | 1.8970 | 0.057822 | 0.2875434 | no |
| Sobic.001G427000 | 5332.4575 | -1.0224 | 0.5390 | -1.8970 | 0.057825 | 0.2875434 | no |
| Sobic.001G375650 | 20.7436 | 1.4187 | 0.7479 | 1.8969 | 0.057842 | 0.28756 | no |
| Sobic.002G291700 | 2999.4605 | -0.7404 | 0.3904 | -1.8966 | 0.0578826 | 0.2876471 | no |
| Sobic.001G273500 | 9.2324 | 2.3110 | 1.2185 | 1.8966 | 0.0578868 | 0.2876471 | no |
| Sobic.010G028000 | 1333.5889 | -0.7546 | 0.3979 | -1.8962 | 0.0579302 | 0.2877949 | no |
| Sobic.003G040900 | 37007.9682 | 0.7066 | 0.3727 | 1.8961 | 0.0579494 | 0.2878228 | no |
| Sobic.004G053200 | 5.9536 | 2.3278 | 1.2279 | 1.8957 | 0.0580044 | 0.2880281 | no |
| Sobic.009G065400 | 3238.0531 | -0.6013 | 0.3172 | -1.8955 | 0.0580231 | 0.288053 | no |
| Sobic.009G140300 | 4383.8778 | -0.5471 | 0.2887 | -1.8953 | 0.058058 | 0.2880833 | no |
| Sobic.008G118400 | 290.3063 | 0.8479 | 0.4474 | 1.8953 | 0.0580588 | 0.2880833 | no |
| Sobic.010G035400 | 949.8768 | -0.7508 | 0.3962 | -1.8952 | 0.0580702 | 0.2880833 | no |
| Sobic.003G261900 | 340.0259 | -0.8957 | 0.4727 | -1.8949 | 0.05811 | 0.2882128 | no |
| Sobic.002G090200 | 163.2876 | 0.7707 | 0.4068 | 1.8947 | 0.0581329 | 0.2882586 | no |
| Sobic.001G467600 | 82.9211 | 1.6864 | 0.8903 | 1.8943 | 0.0581825 | 0.2884368 | no |
| Sobic.003G194200 | 19.3586 | -1.2472 | 0.6585 | -1.8941 | 0.058207 | 0.2884458 | no |
| Sobic.003G396800 | 1532.3113 | 0.5730 | 0.3025 | 1.8941 | 0.0582116 | 0.2884458 | no |
| Sobic.004G160700 | 4194.4027 | 0.6064 | 0.3201 | 1.8940 | 0.0582302 | 0.2884698 | no |
| Sobic.004G317500 | 2027.5435 | -0.7199 | 0.3802 | -1.8934 | 0.0583095 | 0.2887951 | no |
| Sobic.009G003700 | 2680.4651 | 0.7535 | 0.3980 | 1.8930 | 0.0583539 | 0.288827 | no |
| Sobic.002G004300 | 13.5115 | 2.3208 | 1.2260 | 1.8930 | 0.0583545 | 0.288827 | no |
| Sobic.007G062200 | 400.2218 | -0.6102 | 0.3223 | -1.8930 | 0.0583571 | 0.288827 | no |
| Sobic.003G298900 | 229.3932 | 1.0214 | 0.5396 | 1.8927 | 0.0584023 | 0.2889233 | no |
| Sobic.009G069200 | 268.2069 | 0.8777 | 0.4637 | 1.8927 | 0.0584039 | 0.2889233 | no |
| Sobic.004G075000 | 383.7536 | -1.0568 | 0.5586 | -1.8921 | 0.0584809 | 0.2891421 | no |
| Sobic.002G124700 | 15720.5732 | 0.8170 | 0.4318 | 1.8920 | 0.0584882 | 0.2891421 | no |
| Sobic.001G252700 | 230.8944 | 1.3426 | 0.7096 | 1.8920 | 0.0584893 | 0.2891421 | no |
| Sobic.002G373600 | 115.0760 | 0.9092 | 0.4808 | 1.8911 | 0.0586062 | 0.2896523 | no |
| Sobic.003G387500 | 9.4736 | -2.0490 | 1.0836 | -1.8909 | 0.0586391 | 0.289747 | no |
| Sobic.001G067000 | 188.6677 | 0.9978 | 0.5278 | 1.8907 | 0.0586705 | 0.2897983 | no |
| Sobic.007G213600 | 710.7585 | 0.6867 | 0.3632 | 1.8905 | 0.0586904 | 0.2897983 | no |

| Sobic.001G539016 | 825.5973 | 0.5972 | 0.3159 | 1.8905 | 0.0586907 | 0.2897983 | no |
| --- | --- | --- | --- | --- | --- | --- | --- |
| Sobic.009G243800 | 595.6882 | 0.4987 | 0.2639 | 1.8899 | 0.0587695 | 0.2901192 | no |
| Sobic.001G030000 | 2526.0153 | 0.3980 | 0.2106 | 1.8898 | 0.0587867 | 0.2901362 | no |
| Sobic.002G121000 | 362.3475 | 0.6803 | 0.3601 | 1.8892 | 0.0588597 | 0.2904224 | no |
| Sobic.003G193800 | 3028.6539 | -1.4905 | 0.7890 | -1.8891 | 0.0588784 | 0.2904224 | no |
| Sobic.001G216600 | 350.4380 | -0.5960 | 0.3155 | -1.8889 | 0.0588994 | 0.2904224 | no |
| Sobic.009G212100 | 917.1694 | 1.0585 | 0.5604 | 1.8889 | 0.0588997 | 0.2904224 | no |
| Sobic.001G415000 | 308.7801 | 0.6291 | 0.3330 | 1.8888 | 0.0589168 | 0.2904386 | no |
| Sobic.010G042100 | 7608.7980 | -0.4463 | 0.2363 | -1.8885 | 0.0589539 | 0.2905534 | no |
| Sobic.010G015900 | 5732.3803 | 0.6834 | 0.3621 | 1.8871 | 0.059151 | 0.2914251 | no |
| Sobic.001G406000 | 2039.1222 | -1.1732 | 0.6217 | -1.8870 | 0.0591584 | 0.2914251 | no |
| Sobic.004G063300 | 337.0719 | 0.7741 | 0.4103 | 1.8866 | 0.0592162 | 0.2916418 | no |
| Sobic.003G269500 | 119.0691 | 1.3725 | 0.7277 | 1.8861 | 0.0592848 | 0.2918471 | no |
| Sobic.001G440800 | 434.5333 | 0.7411 | 0.3930 | 1.8860 | 0.0592988 | 0.2918471 | no |
| Sobic.010G171900 | 120.4760 | -1.5116 | 0.8015 | -1.8860 | 0.0592994 | 0.2918471 | no |
| Sobic.001G412500 | 452.3295 | 1.1329 | 0.6008 | 1.8855 | 0.0593668 | 0.2920713 | no |
| Sobic.002G013400 | 37.4285 | 1.8353 | 0.9734 | 1.8854 | 0.0593726 | 0.2920713 | no |
| Sobic.002G153100 | 105.1690 | -1.2416 | 0.6586 | -1.8851 | 0.0594106 | 0.292083 | no |
| Sobic.003G203300 | 1532.6027 | -0.5922 | 0.3141 | -1.8850 | 0.0594237 | 0.292083 | no |
| Sobic.007G118500 | 577.8697 | 0.5199 | 0.2758 | 1.8850 | 0.059427 | 0.292083 | no |
| Sobic.002G413800 | 14.1780 | 1.9509 | 1.0350 | 1.8849 | 0.059441 | 0.292083 | no |
| Sobic.001G486600 | 43.6142 | 0.8648 | 0.4588 | 1.8849 | 0.0594443 | 0.292083 | no |
| Sobic.007G226700 | 12.6139 | 1.6005 | 0.8492 | 1.8846 | 0.0594807 | 0.2921489 | no |
| Sobic.009G163100 | 40.8202 | 1.3190 | 0.6999 | 1.8846 | 0.0594854 | 0.2921489 | no |
| Sobic.007G087900 | 30.6614 | 1.2198 | 0.6474 | 1.8841 | 0.0595471 | 0.2923837 | no |
| Sobic.010G002500 | 193.4934 | -0.9670 | 0.5133 | -1.8838 | 0.0595867 | 0.2924647 | no |
| Sobic.006G095000 | 13.4502 | 1.6210 | 0.8605 | 1.8838 | 0.0595913 | 0.2924647 | no |
| Sobic.003G070100 | 269.5966 | 0.8702 | 0.4620 | 1.8835 | 0.0596275 | 0.2925743 | no |
| Sobic.001G353200 | 1230.8813 | -0.6680 | 0.3547 | -1.8834 | 0.0596495 | 0.2926144 | no |
| Sobic.009G070600 | 3032.2731 | -0.7801 | 0.4143 | -1.8830 | 0.0597002 | 0.2926786 | no |
| Sobic.006G002100 | 95.3783 | -1.0890 | 0.5783 | -1.8830 | 0.0597017 | 0.2926786 | no |
| Sobic.010G140600 | 5320.8262 | -0.5389 | 0.2862 | -1.8830 | 0.0597043 | 0.2926786 | no |
| Sobic.001G359500 | 1090.0821 | 1.0380 | 0.5513 | 1.8827 | 0.0597358 | 0.2927651 | no |
| Sobic.003G072300 | 4170.7966 | -0.5520 | 0.2932 | -1.8825 | 0.059766 | 0.2927976 | no |
| Sobic.007G183700 | 2102.6556 | 0.4281 | 0.2274 | 1.8824 | 0.0597812 | 0.2927976 | no |
| Sobic.007G070200 | 26.4302 | -1.3361 | 0.7098 | -1.8823 | 0.0597939 | 0.2927976 | no |
| Sobic.004G288900 | 83.3072 | 0.7468 | 0.3968 | 1.8823 | 0.0598009 | 0.2927976 | no |
| Sobic.004G179600 | 737.9163 | -0.6564 | 0.3487 | -1.8822 | 0.0598118 | 0.2927976 | no |
| Sobic.008G029500 | 222.4652 | 1.3852 | 0.7361 | 1.8817 | 0.0598802 | 0.2930644 | no |
| Sobic.005G163600 | 75.1375 | 1.2505 | 0.6646 | 1.8815 | 0.0598982 | 0.2930844 | no |
| Sobic.005G043000 | 40.2815 | 0.9532 | 0.5067 | 1.8813 | 0.0599335 | 0.2931448 | no |
| Sobic.001G020500 | 1076.7017 | 0.7847 | 0.4171 | 1.8812 | 0.059942 | 0.2931448 | no |
| Sobic.010G255300 | 7564.0706 | -0.9188 | 0.4884 | -1.8811 | 0.0599612 | 0.2931448 | no |
| Sobic.003G172800 | 586.7918 | -1.0422 | 0.5540 | -1.8810 | 0.0599662 | 0.2931448 | no |
| Sobic.007G146100 | 1358.1239 | 0.7426 | 0.3949 | 1.8806 | 0.0600324 | 0.2934007 | no |
| Sobic.006G067150 | 50.0683 | -0.8099 | 0.4307 | -1.8803 | 0.0600632 | 0.2934826 | no |
| Sobic.002G205800 | 7.5379 | 2.5664 | 1.3650 | 1.8802 | 0.060077 | 0.2934826 | no |
| Sobic.009G250100 | 13.7328 | 2.8678 | 1.5254 | 1.8800 | 0.060103 | 0.2935413 | no |
| Sobic.004G043750 | 1364.7769 | 0.5414 | 0.2880 | 1.8798 | 0.0601296 | 0.2936034 | no |
| Sobic.006G212700 | 11.5442 | -1.7151 | 0.9125 | -1.8796 | 0.0601653 | 0.2937097 | no |
| Sobic.006G138100 | 936.1255 | -0.7952 | 0.4231 | -1.8792 | 0.0602145 | 0.2937314 | no |
| Sobic.009G031100 | 66.0644 | 1.1564 | 0.6154 | 1.8791 | 0.0602241 | 0.2937314 | no |
| Sobic.002G283100 | 24.9336 | 1.4150 | 0.7530 | 1.8791 | 0.0602334 | 0.2937314 | no |
| Sobic.003G316500 | 1448.4031 | 0.7101 | 0.3779 | 1.8790 | 0.0602384 | 0.2937314 | no |
| Sobic.009G074700 | 3452.0329 | 0.5329 | 0.2836 | 1.8790 | 0.0602394 | 0.2937314 | no |
| Sobic.009G152600 | 649.7799 | -0.8644 | 0.4601 | -1.8788 | 0.0602694 | 0.2937788 | no |
| Sobic.003G334700 | 1648.5194 | 0.5549 | 0.2954 | 1.8786 | 0.0603023 | 0.2937788 | no |

| Sobic.008G164800 | 1398.7612 | 1.7144 | 0.9126 | 1.8786 | 0.060305 | 0.2937788 | no |
| --- | --- | --- | --- | --- | --- | --- | --- |
| Sobic.008G130900 | 442.9240 | -0.7421 | 0.3950 | -1.8784 | 0.0603285 | 0.2937788 | no |
| Sobic.001G077100 | 562.6296 | -0.8512 | 0.4532 | -1.8784 | 0.0603293 | 0.2937788 | no |
| Sobic.001G536200 | 27.4410 | -1.4537 | 0.7739 | -1.8784 | 0.0603327 | 0.2937788 | no |
| Sobic.009G184400 | 1200.3347 | -1.0398 | 0.5537 | -1.8781 | 0.0603671 | 0.2938784 | no |
| Sobic.004G219700 | 32.0226 | 1.9103 | 1.0172 | 1.8780 | 0.0603826 | 0.2938861 | no |
| Sobic.001G245900 | 4967.0582 | 0.5754 | 0.3064 | 1.8776 | 0.0604304 | 0.2940154 | no |
| Sobic.001G269300 | 97.1806 | -1.4782 | 0.7873 | -1.8776 | 0.060437 | 0.2940154 | no |
| Sobic.008G088818 | 42.1731 | 1.6596 | 0.8842 | 1.8769 | 0.0605267 | 0.2943836 | no |
| Sobic.004G199200 | 43.4034 | 1.0730 | 0.5718 | 1.8767 | 0.0605604 | 0.2944798 | no |
| Sobic.009G171300 | 801.3000 | -1.0227 | 0.5450 | -1.8766 | 0.0605765 | 0.2944902 | no |
| Sobic.004G073100 | 1306.4859 | -0.5225 | 0.2785 | -1.8764 | 0.0606043 | 0.2945571 | no |
| Sobic.005G194800 | 634.6733 | -1.0176 | 0.5424 | -1.8760 | 0.0606607 | 0.2947637 | no |
| Sobic.009G165700 | 1635.8923 | 0.6250 | 0.3332 | 1.8757 | 0.0606931 | 0.294853 | no |
| Sobic.002G348600 | 1961.1336 | 0.5068 | 0.2702 | 1.8753 | 0.0607466 | 0.2950111 | no |
| Sobic.003G074400 | 1127.3713 | -0.5335 | 0.2845 | -1.8753 | 0.0607536 | 0.2950111 | no |
| Sobic.004G267600 | 167.5131 | 0.6862 | 0.3660 | 1.8750 | 0.0607934 | 0.2951362 | no |
| Sobic.006G065600 | 1831.1615 | -0.4545 | 0.2424 | -1.8747 | 0.0608375 | 0.2951655 | no |
| Sobic.009G070100 | 1229.5682 | -0.7350 | 0.3921 | -1.8746 | 0.0608441 | 0.2951655 | no |
| Sobic.002G226800 | 5.8780 | 3.7228 | 1.9862 | 1.8743 | 0.0608833 | 0.2951655 | no |
| Sobic.006G111300 | 76.5424 | 0.9374 | 0.5002 | 1.8742 | 0.0608995 | 0.2951655 | no |
| Sobic.006G228500 | 2627.1302 | -0.5316 | 0.2836 | -1.8742 | 0.0608999 | 0.2951655 | no |
| Sobic.001G484400 | 3935.4935 | -0.7365 | 0.3930 | -1.8741 | 0.0609161 | 0.2951655 | no |
| Sobic.002G408800 | 1518.5454 | 0.5604 | 0.2990 | 1.8740 | 0.060924 | 0.2951655 | no |
| Sobic.002G383100 | 71.2902 | 1.6863 | 0.8998 | 1.8740 | 0.0609351 | 0.2951655 | no |
| Sobic.007G023800 | 43.5675 | -1.1369 | 0.6067 | -1.8740 | 0.0609365 | 0.2951655 | no |
| Sobic.006G162200 | 3050.1217 | -0.9814 | 0.5237 | -1.8739 | 0.0609509 | 0.2951655 | no |
| Sobic.006G209500 | 2870.8000 | -0.4533 | 0.2419 | -1.8738 | 0.0609534 | 0.2951655 | no |
| Sobic.006G028100 | 559.5393 | 0.8178 | 0.4365 | 1.8734 | 0.0610123 | 0.2953829 | no |
| Sobic.001G006400 | 21645.1357 | -0.5958 | 0.3181 | -1.8733 | 0.0610289 | 0.2953956 | no |
| Sobic.005G150900 | 766.8677 | 0.4382 | 0.2339 | 1.8730 | 0.061068 | 0.2955172 | no |
| Sobic.007G038100 | 78.0928 | -1.3233 | 0.7066 | -1.8729 | 0.0610886 | 0.2955488 | no |
| Sobic.004G052100 | 196.0268 | -0.8176 | 0.4366 | -1.8726 | 0.0611194 | 0.2956293 | no |
| Sobic.010G058650 | 29.7032 | 1.1870 | 0.6339 | 1.8725 | 0.0611333 | 0.2956293 | no |
| Sobic.005G051200 | 43.6649 | 1.2640 | 0.6753 | 1.8719 | 0.0612219 | 0.2957908 | no |
| Sobic.010G164400 | 220.5289 | 2.3272 | 1.2432 | 1.8719 | 0.0612224 | 0.2957908 | no |
| Sobic.002G145600 | 36.8704 | -1.3118 | 0.7008 | -1.8718 | 0.0612331 | 0.2957908 | no |
| Sobic.002G406400 | 118.6185 | -1.1549 | 0.6170 | -1.8718 | 0.0612373 | 0.2957908 | no |
| Sobic.001G457500 | 20.6303 | 1.7120 | 0.9147 | 1.8717 | 0.061252 | 0.2957908 | no |
| Sobic.002G419000 | 435.9002 | 0.6046 | 0.3230 | 1.8716 | 0.0612586 | 0.2957908 | no |
| Sobic.006G208800 | 338.1859 | 1.2030 | 0.6428 | 1.8716 | 0.0612648 | 0.2957908 | no |
| Sobic.004G235600 | 2400.7240 | -0.5312 | 0.2839 | -1.8714 | 0.0612865 | 0.2958276 | no |
| Sobic.004G291200 | 161.9800 | -0.8112 | 0.4336 | -1.8708 | 0.0613673 | 0.2961501 | no |
| Sobic.001G149800 | 7.3559 | 4.3408 | 2.3205 | 1.8707 | 0.0613901 | 0.2961821 | no |
| Sobic.006G032200 | 47.8436 | 1.2999 | 0.6949 | 1.8706 | 0.0614065 | 0.2961821 | no |
| Sobic.004G037900 | 763.2588 | 0.6259 | 0.3346 | 1.8705 | 0.0614161 | 0.2961821 | no |
| Sobic.001G369200 | 92.9474 | -4.2464 | 2.2708 | -1.8700 | 0.0614882 | 0.2964621 | no |
| Sobic.001G219200 | 5.1399 | 2.6117 | 1.3968 | 1.8697 | 0.0615215 | 0.296555 | no |
| Sobic.003G223300 | 117.3172 | 0.7084 | 0.3789 | 1.8695 | 0.061549 | 0.2966192 | no |
| Sobic.003G274300 | 1978.7535 | -1.1672 | 0.6244 | -1.8694 | 0.0615632 | 0.2966201 | no |
| Sobic.001G225700 | 2804.7710 | -0.5566 | 0.2978 | -1.8691 | 0.0616047 | 0.2966911 | no |
| Sobic.001G100000 | 8196.5015 | -0.8150 | 0.4360 | -1.8691 | 0.0616061 | 0.2966911 | no |
| Sobic.010G222400 | 228.3142 | 0.8569 | 0.4586 | 1.8687 | 0.0616615 | 0.2968522 | no |
| Sobic.001G538866 | 1793.6267 | -0.8999 | 0.4816 | -1.8687 | 0.0616677 | 0.2968522 | no |
| Sobic.006G207300 | 403.4934 | 0.6545 | 0.3503 | 1.8685 | 0.0616905 | 0.2968942 | no |
| Sobic.001G543400 | 84.8680 | 1.0158 | 0.5437 | 1.8681 | 0.0617474 | 0.2971005 | no |
| Sobic.001G391400 | 7985.0198 | -0.8576 | 0.4591 | -1.8680 | 0.0617638 | 0.2971115 | no |

| Sobic.010G212000 | 8.6534 | 2.3061 | 1.2347 | 1.8677 | 0.0617993 | 0.2972145 | no |
| --- | --- | --- | --- | --- | --- | --- | --- |
| Sobic.003G324500 | 50.0518 | -1.4772 | 0.7910 | -1.8675 | 0.0618293 | 0.2972876 | no |
| Sobic.007G162600 | 1614.7821 | -0.5936 | 0.3179 | -1.8673 | 0.0618589 | 0.2972876 | no |
| Sobic.007G189500 | 4987.9614 | -0.6909 | 0.3700 | -1.8673 | 0.0618612 | 0.2972876 | no |
| Sobic.003G044700 | 6.9282 | 2.2074 | 1.1823 | 1.8670 | 0.0618968 | 0.2972876 | no |
| Sobic.010G204100 | 114.2525 | 1.5857 | 0.8493 | 1.8670 | 0.0618975 | 0.2972876 | no |
| Sobic.007G034500 | 2480.5340 | -0.7341 | 0.3932 | -1.8670 | 0.0618991 | 0.2972876 | no |
| Sobic.001G106800 | 9.2375 | -2.8850 | 1.5455 | -1.8668 | 0.0619343 | 0.2973888 | no |
| Sobic.010G265600 | 3542.9748 | 1.2253 | 0.6565 | 1.8665 | 0.0619767 | 0.297483 | no |
| Sobic.009G215500 | 2975.1992 | -0.5638 | 0.3021 | -1.8664 | 0.0619821 | 0.297483 | no |
| Sobic.001G362601 | 303.3527 | -0.8864 | 0.4751 | -1.8655 | 0.062109 | 0.2980245 | no |
| Sobic.007G133000 | 5839.9397 | -1.0098 | 0.5414 | -1.8652 | 0.0621537 | 0.2981709 | no |
| Sobic.004G152501 | 66.0772 | 0.8374 | 0.4491 | 1.8648 | 0.062204 | 0.2983441 | no |
| Sobic.002G318300 | 428.3444 | 0.9768 | 0.5239 | 1.8643 | 0.0622783 | 0.2986162 | no |
| Sobic.001G193200 | 3736.0956 | -0.7250 | 0.3889 | -1.8642 | 0.062289 | 0.2986162 | no |
| Sobic.001G527200 | 143.1715 | -0.9582 | 0.5140 | -1.8641 | 0.0623138 | 0.2986673 | no |
| Sobic.003G408700 | 820.8118 | -0.7812 | 0.4192 | -1.8638 | 0.0623515 | 0.2987395 | no |
| Sobic.005G120800 | 7.3496 | -1.7547 | 0.9415 | -1.8637 | 0.0623572 | 0.2987395 | no |
| Sobic.004G106300 | 130.4979 | -0.9828 | 0.5275 | -1.8633 | 0.0624238 | 0.2989907 | no |
| Sobic.009G107400 | 3502.2482 | 0.6466 | 0.3471 | 1.8630 | 0.0624626 | 0.2991087 | no |
| Sobic.002G055200 | 34.3856 | -1.3552 | 0.7275 | -1.8629 | 0.0624827 | 0.2991367 | no |
| Sobic.003G404600 | 10.9071 | 1.9560 | 1.0501 | 1.8627 | 0.0624988 | 0.2991427 | no |
| Sobic.002G245000 | 1407.8636 | 0.7636 | 0.4099 | 1.8626 | 0.0625123 | 0.2991427 | no |
| Sobic.002G427800 | 79.3528 | 0.7452 | 0.4001 | 1.8625 | 0.062535 | 0.2991835 | no |
| Sobic.003G324000 | 3069.2757 | -0.9567 | 0.5137 | -1.8623 | 0.0625623 | 0.2992288 | no |
| Sobic.001G028100 | 16242.8246 | -0.6188 | 0.3323 | -1.8622 | 0.0625792 | 0.2992288 | no |
| Sobic.004G010600 | 352.7253 | 0.7997 | 0.4294 | 1.8621 | 0.062587 | 0.2992288 | no |
| Sobic.004G174700 | 554.3365 | 0.6544 | 0.3515 | 1.8617 | 0.0626429 | 0.2993589 | no |
| Sobic.010G072900 | 3507.6847 | -1.4619 | 0.7853 | -1.8616 | 0.062655 | 0.2993589 | no |
| Sobic.009G085500 | 1462.4835 | -0.6234 | 0.3349 | -1.8616 | 0.0626569 | 0.2993589 | no |
| Sobic.009G030400 | 510.4342 | 0.6793 | 0.3649 | 1.8614 | 0.0626835 | 0.2994182 | no |
| Sobic.001G373000 | 412.2569 | 0.5796 | 0.3114 | 1.8612 | 0.0627142 | 0.2994973 | no |
| Sobic.009G180200 | 1640.1572 | -1.0652 | 0.5724 | -1.8610 | 0.0627448 | 0.2995217 | no |
| Sobic.002G383900 | 153.2776 | 0.6368 | 0.3422 | 1.8609 | 0.0627582 | 0.2995217 | no |
| Sobic.010G054600 | 432.2809 | 1.3778 | 0.7404 | 1.8609 | 0.0627619 | 0.2995217 | no |
| Sobic.010G170000 | 497.3200 | 1.0560 | 0.5675 | 1.8606 | 0.0628069 | 0.2996685 | no |
| Sobic.004G031501 | 68.4211 | -0.7322 | 0.3936 | -1.8604 | 0.0628345 | 0.2997324 | no |
| Sobic.007G003200 | 30.7934 | 2.4466 | 1.3153 | 1.8602 | 0.0628598 | 0.2997851 | no |
| Sobic.003G239000 | 14.8468 | 1.9052 | 1.0249 | 1.8590 | 0.0630338 | 0.3005461 | no |
| Sobic.006G230400 | 670.1322 | 0.7108 | 0.3824 | 1.8589 | 0.0630478 | 0.3005461 | no |
| Sobic.001G370900 | 2076.3926 | 0.6480 | 0.3486 | 1.8586 | 0.0630782 | 0.3006228 | no |
| Sobic.010G214400 | 176.8925 | 0.9772 | 0.5258 | 1.8585 | 0.0630932 | 0.3006265 | no |
| Sobic.010G065900 | 127.0463 | 0.7206 | 0.3877 | 1.8583 | 0.0631265 | 0.3007173 | no |
| Sobic.003G086300 | 2273.3783 | 0.6514 | 0.3506 | 1.8578 | 0.0631908 | 0.3008889 | no |
| Sobic.006G142775 | 721.2891 | 0.6975 | 0.3754 | 1.8578 | 0.0631911 | 0.3008889 | no |
| Sobic.003G167632 | 176.1227 | -0.7980 | 0.4296 | -1.8576 | 0.0632249 | 0.3008983 | no |
| Sobic.005G090200 | 195.1277 | 0.5811 | 0.3128 | 1.8575 | 0.0632368 | 0.3008983 | no |
| Sobic.003G437800 | 58983.0239 | -0.9786 | 0.5269 | -1.8573 | 0.063266 | 0.3008983 | no |
| Sobic.003G054200 | 1125.7573 | -0.6979 | 0.3757 | -1.8573 | 0.063267 | 0.3008983 | no |
| Sobic.004G147600 | 9.5693 | 1.7699 | 0.9529 | 1.8573 | 0.0632726 | 0.3008983 | no |
| Sobic.003G329000 | 725.9133 | 1.5594 | 0.8396 | 1.8572 | 0.0632787 | 0.3008983 | no |
| Sobic.005G135000 | 15.7076 | 1.5773 | 0.8497 | 1.8564 | 0.0633931 | 0.3012794 | no |
| Sobic.003G198600 | 99.1135 | -0.7317 | 0.3942 | -1.8562 | 0.0634261 | 0.3012794 | no |
| Sobic.004G188100 | 50.8563 | 0.9451 | 0.5091 | 1.8562 | 0.0634316 | 0.3012794 | no |
| Sobic.004G153400 | 2761.6238 | -0.9120 | 0.4914 | -1.8560 | 0.0634522 | 0.3012794 | no |
| Sobic.001G089800 | 856.7108 | 0.6629 | 0.3572 | 1.8560 | 0.0634584 | 0.3012794 | no |
| Sobic.008G073100 | 448.8995 | -0.8358 | 0.4503 | -1.8560 | 0.0634599 | 0.3012794 | no |

| Sobic.004G063100 | 2950.4082 | -0.4266 | 0.2299 | -1.8559 | 0.0634626 | 0.3012794 | no |
| --- | --- | --- | --- | --- | --- | --- | --- |
| Sobic.003G359500 | 2624.0197 | -0.8002 | 0.4312 | -1.8559 | 0.0634731 | 0.3012794 | no |
| Sobic.001G321800 | 96.7620 | 1.8975 | 1.0228 | 1.8551 | 0.0635756 | 0.3016066 | no |
| Sobic.004G286000 | 87.9845 | 0.9596 | 0.5173 | 1.8551 | 0.0635885 | 0.3016066 | no |
| Sobic.003G339900 | 1336.7338 | 0.6106 | 0.3292 | 1.8550 | 0.0635955 | 0.3016066 | no |
| Sobic.005G005800 | 18.7595 | 2.9984 | 1.6164 | 1.8550 | 0.0635994 | 0.3016066 | no |
| Sobic.006G090450 | 41.9905 | 1.6453 | 0.8870 | 1.8548 | 0.0636221 | 0.3016066 | no |
| Sobic.002G231800 | 15.0664 | -1.9580 | 1.0557 | -1.8547 | 0.0636381 | 0.3016066 | no |
| Sobic.003G133900 | 7838.7721 | -0.4842 | 0.2611 | -1.8546 | 0.0636536 | 0.3016066 | no |
| Sobic.002G206600 | 34.7561 | 1.7735 | 0.9563 | 1.8546 | 0.0636564 | 0.3016066 | no |
| Sobic.003G231100 | 502.9980 | 0.5839 | 0.3149 | 1.8542 | 0.0637164 | 0.3017826 | no |
| Sobic.009G109700 | 4871.0492 | 0.6041 | 0.3258 | 1.8541 | 0.0637222 | 0.3017826 | no |
| Sobic.004G042300 | 1739.7409 | 0.5265 | 0.2840 | 1.8538 | 0.0637723 | 0.3019305 | no |
[truncated: 1,642,898 more chars]
