# Supplementary material for: Development of vegetative oil sorghum: From lab‐to‐field
Source: Plant Biotechnol J. 2024 Nov 30;23(2):660–73. doi: 10.1111/pbi.14527 (PMC11772366; doi:10.1111/pbi.14527)
Supplement: Supplementary file 4 — Table S3 GO analysis on differentially expressed genes. [file PBI-23-660-s001.docx]

Table S3. GO analysis on differentially expressed genes. RNAseq was performed on mature *Sorghum bicolor* leaves and differentially expressed genes were identified with DEseq2. Genes that were significantly up- or down-regulated (|log2FC| > 2, padj < 0.05) in the transgenic high-oil line compared to the wildtype were analyzed with GO to estimate molecular characteristics. Listed are the GO terms that match to the genes with a false discovery rate (FDR) less than 5%.

| Biological process (BP)  GO Term | Expression | pvalue | Fold Enrichment | # of Genes | % of Enriched Genes |
| --- | --- | --- | --- | --- | --- |
| ~glycolytic process | Up-regulated in transgenic | 9.67E-09 | 16.00548992 | 10 | 2.906976744 |
| ~glutathione metabolic process | Up-regulated in transgenic | 1.04E-05 | 8.4647591 | 9 | 2.61627907 |
| ~fatty acid biosynthetic process | Up-regulated in transgenic | 6.81E-05 | 7.84785312 | 8 | 2.325581395 |
| ~photosynthesis, light harvesting in ph | Down-regulated in transgenic | 5.03E-08 | 55.87916667 | 6 | 2.790697674 |
| ~protein-chromophore linkage | Down-regulated in transgenic | 8.76E-06 | 20.9546875 | 6 | 2.790697674 |
| ~response to light stimulus | Down-regulated in transgenic | 3.63E-05 | 15.71601563 | 6 | 2.790697674 |
|  |  |  |  |  |  |
| Molecular Function (MF) |  |  |  |  |  |
| GO Term | Expression | pvalue | Fold Enrichment | # of Genes | % of Enriched Genes |
| ~glutathione transferase activity | Up-regulated in transgenic | 3.99E-05 | 7.066204 | 9 | 2.61627907 |
| ~ACP phosphopantetheine attachment | Up-regulated in transgenic | 6.69E-05 | 21.70663974 | 5 | 1.453488372 |
| ~chlorophyll binding | Down-regulated in transgenic | 1.46E-06 | 30.04542807 | 6 | 2.790697674 |
|  |  |  |  |  |  |
| Cellular Component (CC) |  |  |  |  |  |
| GO Term | Expression | pvalue | Fold Enrichment | # of Genes | % of Enriched Genes |
| ~photosystem II | Down-regulated in transgenic | 1.31E-12 | 42.03953954 | 10 | 4.651162791 |
| ~chloroplast thylakoid membrane | Down-regulated in transgenic | 4.45E-10 | 9.601623228 | 15 | 6.976744186 |
| ~photosystem I | Down-regulated in transgenic | 1.54E-08 | 40.32681756 | 7 | 3.255813953 |
| ~plastoglobule | Down-regulated in transgenic | 1.20E-06 | 31.10925926 | 6 | 2.790697674 |
| ~chloroplast thylakoid lumen | Down-regulated in transgenic | 1.10E-03 | 58.32986111 | 3 | 1.395348837 |
| ~integral component of membrane | Down-regulated in transgenic | 7.12E-03 | 1.341134489 | 53 | 24.65116279 |
