# Supplementary material for: Development of vegetative oil sorghum: From lab‐to‐field
Source: Plant Biotechnol J. 2024 Nov 30;23(2):660–73. doi: 10.1111/pbi.14527 (PMC11772366; doi:10.1111/pbi.14527)
Supplement: Supplementary file 5 — Table S4 Sorghum bicolor candidate homologues to Arabidopsis thaliana central metabolism genes. [file PBI-23-660-s006.docx]

Table S4. *Sorghum bicolor* candidate homologs to*Arabidopsis thaliana* central metabolism genes.*A. thaliana* genes with accepted metabolic functions were obtained from Kuczynski et al. (2022), ARALIP (http://aralip.plantbiology.msu.edu; Li-Beisson et al., Arabidopsis Book 11: e0161), PlantCyc, or UniProt and candidate homologs were identified by amino acid sequence homology (using BLAST and Clustal2.1 alignm*A*e*.* n*th*t)*a*.*l*T*ia*h*n*e*a* and *S. bicolor* genes were further

characterized for subcellular location prediction (using TargetP 2.0). cTP = chloroplast location, mTP = mitochondrial location, SP = ecretory pathway. Also, information on homology/orthology relations was obtained from PANTHER 16.0 via TAIR. For easy reference, gene expression parameters (base mean, log2FC, and adj p-value) are copied from Table S#. DEseq2). Significant gene expression (Sig) was indicated for genes that were expressed at least |log2FC| = 2 more (up) or less (down) in the transgenic line with padj < 0.05. Also indicated is whether th*A*e*. thaliana* gene is a WRI1 target (Kuczynski et al 2022).

**Abbreviations:** cTP, chloroplast location; FC, fold change; LDO, least-diverged orthologs; mTP, mitochondrial location; nd, expression not determined for this gene; SP, secretory pat

|  | | | | | BLAST - Clustal2.1 | TargetP 2.0 TargetP 2.0 PANTHER 16.0 Expected |  |
| --- | --- | --- | --- | --- | --- | --- | --- |
| Subsystem | Abbreviation | Name | Ath Gene | Ath Source | Sbi Homolog top BLAST hit? Percent Identical Percent | Ath subcellular location Sbi subcellular via TAIR - WRI1 | baseMe |

acetyl-CoA carboxylase comple ACC1 acetyl-CoA carboxylase AT1G3616 Manuscript (Kuczynski; C.; McCorkle; S.; Keereetaweep; J.; Shanklin; J.; & Schwender; J. (2022). An expanded role for the transcription factor WRINKLED1 in the biosynthesis of triacylglycerols during seed development. Frontiers in Plant Scienc Sobic.001G26240 no 64.81 68.42 OTHER 0.9950 OTHER 0.9924 ortholo no 6.0

acetyl-CoA carboxylase comple ACC1 acetyl-CoA carboxylase AT1G3616 Manuscript (Kuczynski; C.; McCorkle; S.; Keereetaweep; J.; Shanklin; J.; & Schwender; J. (2022). An expanded role for the transcription factor WRINKLED1 in the biosynthesis of triacylglycerols during seed development. Frontiers in Plant Scienc Sobic.006G03010 no 67.4 68.71 OTHER 0.7312 cTP 0.1636 ortholo no 22439.6

acetyl-CoA carboxylase comple ACC1 acetyl-CoA carboxylase AT1G3616 Manuscript (Kuczynski; C.; McCorkle; S.; Keereetaweep; J.; Shanklin; J.; & Schwender; J. (2022). An expanded role for the transcription factor WRINKLED1 in the biosynthesis of triacylglycerols during seed development. Frontiers in Plant Scienc Sobic.008G11430 bidirectiona 72.16 72.88 OTHER 0.7312 OTHER 0.9998 LDO no 3144.6

acetyl-CoA carboxylase comple ACC2 (plastid homomeric acetyl-CoA carboxylas AT1G3618 Manuscript (Kuczynski; C.; McCorkle; S.; Keereetaweep; J.; Shanklin; J.; & Schwender; J. (2022). An expanded role for the transcription factor WRINKLED1 in the biosynthesis of triacylglycerols during seed development. Frontiers in Plant Scienc Sobic.001G26240 no 63.42 66.83 OTHER 0.7312 OTHER 0.9924 ortholo no 6.0

acetyl-CoA carboxylase comple ACC2 (plastid homomeric acetyl-CoA carboxylas AT1G3618 Manuscript (Kuczynski; C.; McCorkle; S.; Keereetaweep; J.; Shanklin; J.; & Schwender; J. (2022). An expanded role for the transcription factor WRINKLED1 in the biosynthesis of triacylglycerols during seed development. Frontiers in Plant Scienc Sobic.006G03010 no 65.77 66.84 OTHER 0.7312 cTP 0.1636 ortholo no 22439.6

acetyl-CoA carboxylase comple ACC2 (plastid homomeric acetyl-CoA carboxylas AT1G3618 Manuscript (Kuczynski; C.; McCorkle; S.; Keereetaweep; J.; Shanklin; J.; & Schwender; J. (2022). An expanded role for the transcription factor WRINKLED1 in the biosynthesis of triacylglycerols during seed development. Frontiers in Plant Scienc Sobic.008G11430 yes 63.42 71.3 OTHER 0.7312 OTHER 0.9998 ortholo no 3144.6

| acetyl-CoA carboxylase comple ?-CT | | acetyl Co-enzyme a carboxylase carboxyltransferase alpha subun | AT2G3804 Manuscript (Kuczynski; C.; McCorkle; S.; Keereetaweep; J.; Shanklin; J.; & Schwender; J. (2022). An expanded role for the transcription factor WRINKLED1 in the biosynthesis of triacylglycerols during seed development. Frontiers in Plant Scienc | N/A | N/A | 31.58 | 22.82 | cTP | 0.9939 OTHER 0.9998 | N/A | yes | nd |
| --- | --- | --- | --- | --- | --- | --- | --- | --- | --- | --- | --- | --- |
| acetyl-CoA carboxylase comple BCCP2 | | Biotin Carboxyl Carrier Protei | AT5G1553 Manuscript (Kuczynski; C.; McCorkle; S.; Keereetaweep; J.; Shanklin; J.; & Schwender; J. (2022). An expanded role for the transcription factor WRINKLED1 in the biosynthesis of triacylglycerols during seed development. Frontiers in Plant Scienc | N/A | N/A | 38 | 21.85 | cTP | 0.8185 OTHER 0.9998 | N/A | yes | nd |
| acetyl-CoA carboxylase comple BCCP1 | | Biotin Carboxyl Carrier Protei | AT5G1639 Manuscript (Kuczynski; C.; McCorkle; S.; Keereetaweep; J.; Shanklin; J.; & Schwender; J. (2022). An expanded role for the transcription factor WRINKLED1 in the biosynthesis of triacylglycerols during seed development. Frontiers in Plant Scienc | N/A | N/A | 35.14 | 18.7 | cTP | 0.9907 OTHER 0.9998 | N/A | yes | nd |
| acetyl-CoA carboxylase comple BC | | Biotin Carboxylase of Heteromeric ACCas | AT5G3536 Manuscript (Kuczynski; C.; McCorkle; S.; Keereetaweep; J.; Shanklin; J.; & Schwender; J. (2022). An expanded role for the transcription factor WRINKLED1 in the biosynthesis of triacylglycerols during seed development. Frontiers in Plant Scienc | N/A | N/A | 49.33 | 43.84 | cTP | 0.7011 N/A N/A | N/A | yes | nd |
| acetyl-CoA carboxylase comple β-CT | | acetyl Co-enzyme a carboxylase carboxyltransferase beta subun | ATCG00500 Manuscript (Kuczynski; C.; McCorkle; S.; Keereetaweep; J.; Shanklin; J.; & Schwender; J. (2022). An expanded role for the transcription factor WRINKLED1 in the biosynthesis of triacylglycerols during seed development. Frontiers in Plant Scienc | N/A | N/A | 44.12 | 22.95 | OTHER | 0.9961 OTHER 0.9998 | N/A | no | nd |
| acyl Lipid Metabolis | ACBP6 | Acyl CoA Binding Protei | AT1G3181 Manuscript (Kuczynski; C.; McCorkle; S.; Keereetaweep; J.; Shanklin; J.; & Schwender; J. (2022). An expanded role for the transcription factor WRINKLED1 in the biosynthesis of triacylglycerols during seed development. Frontiers in Plant Scienc | Sobic.007G05270 | yes | 72.53 | 72.53 | OTHER | 0.9877 OTHER 0.9915 | LDO | yes | 2726.8 |
| acyl Lipid Metabolis | ACBP6 | Acyl CoA Binding Protei | AT1G3181 Manuscript (Kuczynski; C.; McCorkle; S.; Keereetaweep; J.; Shanklin; J.; & Schwender; J. (2022). An expanded role for the transcription factor WRINKLED1 in the biosynthesis of triacylglycerols during seed development. Frontiers in Plant Scienc | Sobic.010G01280 | bidirectiona | 72.53 | 72.53 | OTHER | 0.9877 OTHER 0.9881 | O | yes | 10365.7 |
| acyl Lipid Metabolis | ACBP4 | Acyl CoA Binding Protei | AT3G0542 Manuscript (Kuczynski; C.; McCorkle; S.; Keereetaweep; J.; Shanklin; J.; & Schwender; J. (2022). An expanded role for the transcription factor WRINKLED1 in the biosynthesis of triacylglycerols during seed development. Frontiers in Plant Scienc | Sobic.004G35070 | no | 44.91 | 43.89 | OTHER | 0.9972 OTHER 0.9990 | no | no | 361.4 |
| acyl Lipid Metabolis | ACBP4 | Acyl CoA Binding Protei | AT3G0542 Manuscript (Kuczynski; C.; McCorkle; S.; Keereetaweep; J.; Shanklin; J.; & Schwender; J. (2022). An expanded role for the transcription factor WRINKLED1 in the biosynthesis of triacylglycerols during seed development. Frontiers in Plant Scienc | Sobic.008G11840 | yes | 50.48 | 50.19 | OTHER | 0.9972 OTHER 0.9363 | O | no | 290.3 |
| acyl Lipid Metabolis | ACBP4 | Acyl CoA Binding Protei | AT3G0542 Manuscript (Kuczynski; C.; McCorkle; S.; Keereetaweep; J.; Shanklin; J.; & Schwender; J. (2022). An expanded role for the transcription factor WRINKLED1 in the biosynthesis of triacylglycerols during seed development. Frontiers in Plant Scienc | Sobic.001G02310 | bidirectiona | 65.31 | 64.93 | OTHER | 0.9972 OTHER 0.9607 | LDO | no | 1971.3 |
| acyl Lipid Metabolis | PII | regulatory subunit of acetyl-CoA carboxylas | AT4G0190 Manuscript (Kuczynski; C.; McCorkle; S.; Keereetaweep; J.; Shanklin; J.; & Schwender; J. (2022). An expanded role for the transcription factor WRINKLED1 in the biosynthesis of triacylglycerols during seed development. Frontiers in Plant Scienc | Sobic.009G03210 | bidirectiona | 71.53 | 59.04 | cTP | 0.8790 cTP 0.8790 | LDO | yes | 632.7 |
| acyl Lipid Metabolis | ACBP3 | Acyl CoA Binding Protei | AT4G2423 Manuscript (Kuczynski; C.; McCorkle; S.; Keereetaweep; J.; Shanklin; J.; & Schwender; J. (2022). An expanded role for the transcription factor WRINKLED1 in the biosynthesis of triacylglycerols during seed development. Frontiers in Plant Scienc | Sobic.001G43950 | yes | 45.1 | 30.14 | SP | 0.8360 SP 0.6105 | O | no | 619.2 |
| acyl Lipid Metabolis | ACBP2 | Acyl CoA Binding Protei | AT4G2778 Manuscript (Kuczynski; C.; McCorkle; S.; Keereetaweep; J.; Shanklin; J.; & Schwender; J. (2022). An expanded role for the transcription factor WRINKLED1 in the biosynthesis of triacylglycerols during seed development. Frontiers in Plant Scienc | Sobic.001G09280 | no | 50 | 49.29 | OTHER | 0.8204 OTHER 0.9999 | no | no | 12.1 |
| acyl Lipid Metabolis | ACBP2 | Acyl CoA Binding Protei | AT4G2778 Manuscript (Kuczynski; C.; McCorkle; S.; Keereetaweep; J.; Shanklin; J.; & Schwender; J. (2022). An expanded role for the transcription factor WRINKLED1 in the biosynthesis of triacylglycerols during seed development. Frontiers in Plant Scienc | Sobic.006G27090 | bidirectiona | 54.76 | 54.82 | OTHER | 0.8204 OTHER 0.7874 | O | no | 1696.6 |
| acyl Lipid Metabolis | ACBP5 | Acyl CoA Binding Protei | AT5G2763 Manuscript (Kuczynski; C.; McCorkle; S.; Keereetaweep; J.; Shanklin; J.; & Schwender; J. (2022). An expanded role for the transcription factor WRINKLED1 in the biosynthesis of triacylglycerols during seed development. Frontiers in Plant Scienc | Sobic.004G35070 | no | 43.54 | 43.97 | OTHER | 0.7821 OTHER 0.9990 | no | no | 361.4 |
| acyl Lipid Metabolis | ACBP5 | Acyl CoA Binding Protei | AT5G2763 Manuscript (Kuczynski; C.; McCorkle; S.; Keereetaweep; J.; Shanklin; J.; & Schwender; J. (2022). An expanded role for the transcription factor WRINKLED1 in the biosynthesis of triacylglycerols during seed development. Frontiers in Plant Scienc | Sobic.008G11840 | no | 51.18 | 49.52 | OTHER | 0.7821 OTHER 0.9363 | O | no | 290.3 |
| acyl Lipid Metabolis | ACBP5 | Acyl CoA Binding Protei | AT5G2763 Manuscript (Kuczynski; C.; McCorkle; S.; Keereetaweep; J.; Shanklin; J.; & Schwender; J. (2022). An expanded role for the transcription factor WRINKLED1 in the biosynthesis of triacylglycerols during seed development. Frontiers in Plant Scienc | Sobic.001G02310 | yes | 60.66 | 62.72 | OTHER | 0.7821 OTHER 0.9607 | O | no | 1971.3 |
| acyl Lipid Metabolis | ACBP1 | Acyl CoA Binding Protei | AT5G5347 Manuscript (Kuczynski; C.; McCorkle; S.; Keereetaweep; J.; Shanklin; J.; & Schwender; J. (2022). An expanded role for the transcription factor WRINKLED1 in the biosynthesis of triacylglycerols during seed development. Frontiers in Plant Scienc | Sobic.001G09280 | yes | 51.08 | 51.09 | OTHER | 0.8881 OTHER 0.9999 | no | no | 12.1 |
| acyl Lipid Metabolis | ACBP1 | Acyl CoA Binding Protei | AT5G5347 Manuscript (Kuczynski; C.; McCorkle; S.; Keereetaweep; J.; Shanklin; J.; & Schwender; J. (2022). An expanded role for the transcription factor WRINKLED1 in the biosynthesis of triacylglycerols during seed development. Frontiers in Plant Scienc | Sobic.006G27090 | yes | 55.33 | 58.04 | OTHER | 0.8881 OTHER 0.7874 | LDO | no | 1696.6 |
| acylglycerol lipas | TAGL | Triacylglycerol Lipas | AT1G1074 ARALIP (<http://aralip.plantbiology.msu.edu/pathways/triacylglycerol_fatty_acid_degradati> | Sobic.002G11370 | yes | 63.71 | 64.47 | SP | 0.0142 SP 0.8447 | ortholo | no | 2691.2 |
| acylglycerol lipas | TAGL | Triacylglycerol Lipas | AT1G1074 ARALIP (<http://aralip.plantbiology.msu.edu/pathways/triacylglycerol_fatty_acid_degradati> | Sobic.009G13510 | bidirectiona | 68.74 | 70.42 | SP | 0.0142 SP 0.7293 | LDO | no | 2487.6 |
| acylglycerol lipas | MAGL | Monoacylglycerol Lipas | AT1G1109 ARALIP (<http://aralip.plantbiology.msu.edu/pathways/triacylglycerol_fatty_acid_degradati> | Sobic.002G34160 | bidirectiona | 61.41 | 59.26 | OTHER | 0.9998 OTHER 1.0000 | LDO | no | 1101.7 |
| acylglycerol lipas | MAGL | Monoacylglycerol Lipas | AT1G1109 ARALIP (<http://aralip.plantbiology.msu.edu/pathways/triacylglycerol_fatty_acid_degradati> | Sobic.001G27040 | no | 43.55 | 40.19 | OTHER | 0.9998 OTHER 0.9983 | no | no | 894.0 |
| acylglycerol lipas | MAGL | Monoacylglycerol Lipas | AT1G1109 ARALIP (<http://aralip.plantbiology.msu.edu/pathways/triacylglycerol_fatty_acid_degradati> | Sobic.005G00020 | no | 44.72 | 42.21 | OTHER | 0.9998 SP 0.7266 | no | no | nd |
| acylglycerol lipas | MAGL | Monoacylglycerol Lipas | AT1G1109 ARALIP (<http://aralip.plantbiology.msu.edu/pathways/triacylglycerol_fatty_acid_degradati> | Sobic.008G00020 | no | 33.54 | 41.77 | OTHER | 0.9998 OTHER 0.8272 | no | no | nd |
| acylglycerol lipas | MAGL | Monoacylglycerol Lipas | AT1G1109 ARALIP (<http://aralip.plantbiology.msu.edu/pathways/triacylglycerol_fatty_acid_degradati> | Sobic.003G34870 | no | 39.66 | 40.21 | OTHER | 0.9998 OTHER 0.9964 | no | no | 26.0 |
| acylglycerol lipas | MAGL | Monoacylglycerol Lipas | AT1G1109 ARALIP (<http://aralip.plantbiology.msu.edu/pathways/triacylglycerol_fatty_acid_degradati> | Sobic.003G14940 | no | 47.55 | 45.15 | OTHER | 0.9998 OTHER 0.9955 | no | no | 36.7 |
| acylglycerol lipas | MAGL | Monoacylglycerol Lipas | AT1G1109 ARALIP (<http://aralip.plantbiology.msu.edu/pathways/triacylglycerol_fatty_acid_degradati> | Sobic.009G11250 | no | 47.4 | 45.18 | OTHER | 0.9998 OTHER 0.9896 | no | no | 350.2 |
| acylglycerol lipas | MAGL | Monoacylglycerol Lipas | AT1G1109 ARALIP (<http://aralip.plantbiology.msu.edu/pathways/triacylglycerol_fatty_acid_degradati> | Sobic.005G00010 | no | 44.33 | 43.67 | OTHER | 0.9998 OTHER 0.9988 | no | no | 1163.2 |
| acylglycerol lipas | MAGL | Monoacylglycerol Lipas | AT1G1109 ARALIP (<http://aralip.plantbiology.msu.edu/pathways/triacylglycerol_fatty_acid_degradati> | Sobic.008G00010 | no | 44.3 | 42.63 | OTHER | 0.9998 OTHER 0.9997 | no | no | 24.4 |
| acylglycerol lipas | MAGL | Monoacylglycerol Lipas | AT1G1836 ARALIP (<http://aralip.plantbiology.msu.edu/pathways/triacylglycerol_fatty_acid_degradati> | Sobic.003G02890 | no | 53.8 | 49.85 | cTP | 0.4645 OTHER 0.9936 | no | no | 11746.5 |
| acylglycerol lipas | MAGL | Monoacylglycerol Lipas | AT1G1836 ARALIP (<http://aralip.plantbiology.msu.edu/pathways/triacylglycerol_fatty_acid_degradati> | Sobic.002G19400 | bidirectiona | 61.08 | 58.61 | cTP | 0.4645 cTP 0.6087 | ortholo | no | 532.6 |
| acylglycerol lipas | MAGL | Monoacylglycerol Lipas | AT1G1836 ARALIP (<http://aralip.plantbiology.msu.edu/pathways/triacylglycerol_fatty_acid_degradati> | Sobic.001G11170 | no | 60.26 | 57.98 | cTP | 0.4645 OTHER 0.6885 | ortholo | no | 37493.1 |
| acylglycerol lipas | TAGL | Triacylglycerol Lipas | AT1G2333 ARALIP (<http://aralip.plantbiology.msu.edu/pathways/triacylglycerol_fatty_acid_degradati> | Sobic.002G11370 | no | 64.15 | 65.43 | OTHER | 0.7269 SP 0.8447 | ortholo | no | 2691.2 |
| acylglycerol lipas | TAGL | Triacylglycerol Lipas | AT1G2333 ARALIP (<http://aralip.plantbiology.msu.edu/pathways/triacylglycerol_fatty_acid_degradati> | Sobic.009G13510 | yes | 68.51 | 69.96 | OTHER | 0.7269 SP 0.7293 | ortholo | no | 2487.6 |
| acylglycerol lipas acylglycerol lipas | TAGL TAGL | Triacylglycerol Lipas Triacylglycerol Lipas | AT1G4520 ARALIP (<http://aralip.plantbiology.msu.edu/pathways/triacylglycerol_fatty_acid_degradati> AT1G4520 ARALIP (<http://aralip.plantbiology.msu.edu/pathways/triacylglycerol_fatty_acid_degradati> | Sobic.009G04810 Sobic.006G24810 | no  no | 35.92  37.08 | 39.09  37.83 | OTHER OTHER | 0.8908 OTHER 0.9985  0.8908 OTHER 0.9270 | no no | no no | 11.1  nd |
| acylglycerol lipas | TAGL | Triacylglycerol Lipas | AT1G4520 ARALIP (<http://aralip.plantbiology.msu.edu/pathways/triacylglycerol_fatty_acid_degradati> | Sobic.004G31000 | no | 45 | 43.22 | OTHER | 0.8908 OTHER 0.9270 | no | no | 2.4 |
| acylglycerol lipas | TAGL | Triacylglycerol Lipas | AT1G4520 ARALIP (<http://aralip.plantbiology.msu.edu/pathways/triacylglycerol_fatty_acid_degradati> | Sobic.010G08110 | yes | 44.59 | 44.28 | OTHER | 0.8908 OTHER 0.9983 | no | no | 4.1 |
| acylglycerol lipas | MAGL | Monoacylglycerol Lipas | AT1G5276 ARALIP (<http://aralip.plantbiology.msu.edu/pathways/triacylglycerol_fatty_acid_degradati> | Sobic.002G34160 | yes | 38.33 | 41.72 | OTHER | 0.9985 OTHER 1.0000 | no | no | 1101.7 |
| acylglycerol lipas acylglycerol lipas | TAGL TAGL | Triacylglycerol Lipas Triacylglycerol Lipas | AT1G5663 ARALIP (<http://aralip.plantbiology.msu.edu/pathways/triacylglycerol_fatty_acid_degradati> AT1G5663 ARALIP (<http://aralip.plantbiology.msu.edu/pathways/triacylglycerol_fatty_acid_degradati> | Sobic.009G04810 Sobic.006G24810 | no  no | 33.47  38.22 | 34.12  38.27 | OTHER OTHER | 0.9996 OTHER 0.9985  0.9996 OTHER 0.9270 | no ortholo | no no | 11.1  nd |
| acylglycerol lipas | TAGL | Triacylglycerol Lipas | AT1G5663 ARALIP (<http://aralip.plantbiology.msu.edu/pathways/triacylglycerol_fatty_acid_degradati> | Sobic.004G31000 | yes | 46.62 | 45.86 | OTHER | 0.9996 OTHER 0.9995 | no | no | 2.4 |
| acylglycerol lipas | TAGL | Triacylglycerol Lipas | AT1G5663 ARALIP (<http://aralip.plantbiology.msu.edu/pathways/triacylglycerol_fatty_acid_degradati> | Sobic.010G08110 | no | 45.65 | 45.38 | OTHER | 0.9996 OTHER 0.9983 | ortholo | no | 4.1 |
| acylglycerol lipas | MAGL | Monoacylglycerol Lipas | AT1G7348 ARALIP (<http://aralip.plantbiology.msu.edu/pathways/triacylglycerol_fatty_acid_degradati> | Sobic.003G02890 | no | 53.8 | 49.74 | OTHER | 0.4828 OTHER 0.9936 | no | no | 11746.5 |
| acylglycerol lipas | MAGL | Monoacylglycerol Lipas | AT1G7348 ARALIP (<http://aralip.plantbiology.msu.edu/pathways/triacylglycerol_fatty_acid_degradati> | Sobic.002G19400 | yes | 61.08 | 56.78 | OTHER | 0.4828 cTP 0.6087 | LDO | no | 532.6 |
| acylglycerol lipas | MAGL | Monoacylglycerol Lipas | AT1G7348 ARALIP (<http://aralip.plantbiology.msu.edu/pathways/triacylglycerol_fatty_acid_degradati> | Sobic.001G11170 | yes | 60.26 | 55.30 | OTHER | 0.4828 OTHER 0.6885 | ortholo | no | 37493.1 |
| acylglycerol lipas | MAGL | Monoacylglycerol Lipas | AT1G7742 ARALIP (<http://aralip.plantbiology.msu.edu/pathways/triacylglycerol_fatty_acid_degradati> | Sobic.001G27040 | yes | 57.86 | 54.02 | OTHER | 0.7624 OTHER 0.9983 | no | no | 894.0 |
| acylglycerol lipas | MAGL | Monoacylglycerol Lipas | AT1G7742 ARALIP (<http://aralip.plantbiology.msu.edu/pathways/triacylglycerol_fatty_acid_degradati> | Sobic.003G26150 | yes | 59.28 | 56.74 | OTHER | 0.7624 cTP 0.7075 | LDO | no | 667.3 |
| acylglycerol lipas | TAGL | Triacylglycerol Lipas | AT2G1523 ARALIP (<http://aralip.plantbiology.msu.edu/pathways/triacylglycerol_fatty_acid_degradati> | Sobic.002G14070 | yes | 62.09 | 59.54 | SP | 0.0001 SP 1.0000 | LDO | no | 2727.3 |
| acylglycerol lipas | TAGL | Triacylglycerol Lipas | AT2G1523 ARALIP (<http://aralip.plantbiology.msu.edu/pathways/triacylglycerol_fatty_acid_degradati> | Sobic.010G20000 | no | 39.13 | 36.95 | SP | 0.0001 SP 0.9888 | no | no | nd |
| acylglycerol lipas | TAGL | Triacylglycerol Lipas | AT2G1523 ARALIP (<http://aralip.plantbiology.msu.edu/pathways/triacylglycerol_fatty_acid_degradati> | Sobic.006G03750 | no | 41.81 | 42.12 | SP | 0.0001 SP 0.9984 | no | no | nd |
| acylglycerol lipas | TAGL | Triacylglycerol Lipas | AT2G1523 ARALIP (<http://aralip.plantbiology.msu.edu/pathways/triacylglycerol_fatty_acid_degradati> | Sobic.007G19480 | no | 38.54 | 39.72 | SP | 0.0001 OTHER 0.7751 | no | no | 7.3 |
| acylglycerol lipas acylglycerol lipas | TAGL TAGL | Triacylglycerol Lipas Triacylglycerol Lipas | AT2G3169 ARALIP (<http://aralip.plantbiology.msu.edu/pathways/triacylglycerol_fatty_acid_degradati> AT2G3169 ARALIP (<http://aralip.plantbiology.msu.edu/pathways/triacylglycerol_fatty_acid_degradati> | Sobic.009G12530 Sobic.003G39090 | no  no | 43.22  46.15 | 36.89  42.73 | OTHER OTHER | 0.4964 OTHER 0.8051  0.4964 cTP 0.5825 | no ortholo | no no | 478.3  nd |
| acylglycerol lipas | TAGL | Triacylglycerol Lipas | AT2G3169 ARALIP (<http://aralip.plantbiology.msu.edu/pathways/triacylglycerol_fatty_acid_degradati> | Sobic.003G39100 | no | 49.31 | 44.26 | OTHER | 0.4964 cTP 0.4977 | ortholo | no | 7.9 |
| acylglycerol lipas | TAGL | Triacylglycerol Lipas | AT2G3169 ARALIP (<http://aralip.plantbiology.msu.edu/pathways/triacylglycerol_fatty_acid_degradati> | Sobic.005G11140 | yes | 63.31 | 53.83 | OTHER | 0.4964 cTP 0.9131 | ortholo | no | 6.3 |
| acylglycerol lipas | TAGL | Triacylglycerol Lipas | AT2G3169 ARALIP (<http://aralip.plantbiology.msu.edu/pathways/triacylglycerol_fatty_acid_degradati> | Sobic.007G18700 | yes | 58.71 | 50.66 | OTHER | 0.4964 cTP 0.4658 | LDO | no | nd |
| acylglycerol lipas | MAGL | Monoacylglycerol Lipas | AT2G3940 ARALIP (<http://aralip.plantbiology.msu.edu/pathways/triacylglycerol_fatty_acid_degradati> | Sobic.005G00020 | yes | 61.27 | 59.59 | OTHER | 0.9991 SP 0.7266 | ortholo | no | nd |
| acylglycerol lipas | MAGL | Monoacylglycerol Lipas | AT2G3940 ARALIP (<http://aralip.plantbiology.msu.edu/pathways/triacylglycerol_fatty_acid_degradati> | Sobic.008G00020 | no | 43.3 | 54.44 | OTHER | 0.9991 OTHER 0.8272 | ortholo | no | nd |
| acylglycerol lipas | MAGL | Monoacylglycerol Lipas | AT2G3940 ARALIP (<http://aralip.plantbiology.msu.edu/pathways/triacylglycerol_fatty_acid_degradati> | Sobic.003G34860 | no | 57.29 | 54.97 | OTHER | 0.9991 OTHER 0.9972 | ortholo | no | nd |
| acylglycerol lipas | MAGL | Monoacylglycerol Lipas | AT2G3940 ARALIP (<http://aralip.plantbiology.msu.edu/pathways/triacylglycerol_fatty_acid_degradati> | Sobic.003G34870 | no | 51.04 | 50.52 | OTHER | 0.9991 OTHER 0.9964 | ortholo | no | 26.0 |
| acylglycerol lipas | MAGL | Monoacylglycerol Lipas | AT2G3940 ARALIP (<http://aralip.plantbiology.msu.edu/pathways/triacylglycerol_fatty_acid_degradati> | Sobic.003G14940 | no | 58.48 | 54.84 | OTHER | 0.9991 OTHER 0.9955 | no | no | 36.7 |
| acylglycerol lipas | MAGL | Monoacylglycerol Lipas | AT2G3940 ARALIP (<http://aralip.plantbiology.msu.edu/pathways/triacylglycerol_fatty_acid_degradati> | Sobic.009G11250 | no | 54.83 | 51.13 | OTHER | 0.9991 OTHER 0.9896 | no | no | 350.2 |
| acylglycerol lipas | MAGL | Monoacylglycerol Lipas | AT2G3940 ARALIP (<http://aralip.plantbiology.msu.edu/pathways/triacylglycerol_fatty_acid_degradati> | Sobic.005G00010 | no | 55.27 | 54.90 | OTHER | 0.9991 OTHER 0.9988 | ortholo | no | 1163.2 |
| acylglycerol lipas | MAGL | Monoacylglycerol Lipas | AT2G3940 ARALIP (<http://aralip.plantbiology.msu.edu/pathways/triacylglycerol_fatty_acid_degradati> | Sobic.008G00010 | no | 55.96 | 53.87 | OTHER | 0.9991 OTHER 0.9997 | ortholo | no | 24.4 |
| acylglycerol lipas | MAGL | Monoacylglycerol Lipas | AT2G3941 ARALIP (<http://aralip.plantbiology.msu.edu/pathways/triacylglycerol_fatty_acid_degradati> | Sobic.005G00020 | yes | 58.5 | 57.38 | OTHER | 0.9996 SP 0.7266 | ortholo | no | nd |
| acylglycerol lipas | MAGL | Monoacylglycerol Lipas | AT2G3941 ARALIP (<http://aralip.plantbiology.msu.edu/pathways/triacylglycerol_fatty_acid_degradati> | Sobic.008G00020 | no | 42.14 | 52.82 | OTHER | 0.9996 OTHER 0.8272 | ortholo | no | nd |
| acylglycerol lipas | MAGL | Monoacylglycerol Lipas | AT2G3941 ARALIP (<http://aralip.plantbiology.msu.edu/pathways/triacylglycerol_fatty_acid_degradati> | Sobic.003G34860 | no | 57.29 | 54.22 | OTHER | 0.9996 OTHER 0.9972 | ortholo | no | nd |
| acylglycerol lipas | MAGL | Monoacylglycerol Lipas | AT2G3941 ARALIP (<http://aralip.plantbiology.msu.edu/pathways/triacylglycerol_fatty_acid_degradati> | Sobic.003G34870 | no | 50 | 49.49 | OTHER | 0.9996 OTHER 0.9964 | ortholo | no | 26.0 |
| acylglycerol lipas | MAGL | Monoacylglycerol Lipas | AT2G3941 ARALIP (<http://aralip.plantbiology.msu.edu/pathways/triacylglycerol_fatty_acid_degradati> | Sobic.003G14940 | no | 58.48 | 54.02 | OTHER | 0.9996 OTHER 0.9955 | no | no | 36.7 |
| acylglycerol lipas | MAGL | Monoacylglycerol Lipas | AT2G3941 ARALIP (<http://aralip.plantbiology.msu.edu/pathways/triacylglycerol_fatty_acid_degradati> | Sobic.009G11250 | no | 55.97 | 52.40 | OTHER | 0.9996 OTHER 0.9896 | no | no | 350.2 |
| acylglycerol lipas | MAGL | Monoacylglycerol Lipas | AT2G3941 ARALIP (<http://aralip.plantbiology.msu.edu/pathways/triacylglycerol_fatty_acid_degradati> | Sobic.005G00010 | no | 53.42 | 53.53 | OTHER | 0.9996 OTHER 0.9988 | ortholo | no | 1163.2 |
| acylglycerol lipas | MAGL | Monoacylglycerol Lipas | AT2G3941 ARALIP (<http://aralip.plantbiology.msu.edu/pathways/triacylglycerol_fatty_acid_degradati> | Sobic.008G00010 | no | 55.08 | 52.85 | OTHER | 0.9996 OTHER 0.9997 | ortholo | no | 24.4 |
| acylglycerol lipas | MAGL | Monoacylglycerol Lipas | AT2G3942 ARALIP (<http://aralip.plantbiology.msu.edu/pathways/triacylglycerol_fatty_acid_degradati> | Sobic.005G00020 | no | 61.27 | 59.06 | OTHER | 0.9997 SP 0.7266 | ortholo | no | nd |
| acylglycerol lipas | MAGL | Monoacylglycerol Lipas | AT2G3942 ARALIP (<http://aralip.plantbiology.msu.edu/pathways/triacylglycerol_fatty_acid_degradati> | Sobic.008G00020 | no | 42.41 | 53.63 | OTHER | 0.9997 OTHER 0.8272 | ortholo | no | nd |
| acylglycerol lipas | MAGL | Monoacylglycerol Lipas | AT2G3942 ARALIP (<http://aralip.plantbiology.msu.edu/pathways/triacylglycerol_fatty_acid_degradati> | Sobic.003G34860 | yes | 60.88 | 58.12 | OTHER | 0.9997 OTHER 0.9972 | ortholo | no | nd |
| acylglycerol lipas | MAGL | Monoacylglycerol Lipas | AT2G3942 ARALIP (<http://aralip.plantbiology.msu.edu/pathways/triacylglycerol_fatty_acid_degradati> | Sobic.003G34870 | bidirectiona | 53.04 | 52.19 | OTHER | 0.9997 OTHER 0.9964 | ortholo | no | 26.0 |
| acylglycerol lipas | MAGL | Monoacylglycerol Lipas | AT2G3942 ARALIP (<http://aralip.plantbiology.msu.edu/pathways/triacylglycerol_fatty_acid_degradati> | Sobic.003G14940 | no | 59.38 | 54.98 | OTHER | 0.9997 OTHER 0.9955 | no | no | 36.7 |
| acylglycerol lipas | MAGL | Monoacylglycerol Lipas | AT2G3942 ARALIP (<http://aralip.plantbiology.msu.edu/pathways/triacylglycerol_fatty_acid_degradati> | Sobic.009G11250 | no | 57.24 | 53.04 | OTHER | 0.9997 OTHER 0.9896 | no | no | 350.2 |
| acylglycerol lipas | MAGL | Monoacylglycerol Lipas | AT2G3942 ARALIP (<http://aralip.plantbiology.msu.edu/pathways/triacylglycerol_fatty_acid_degradati> | Sobic.005G00010 | yes | 57.42 | 55.77 | OTHER | 0.9997 OTHER 0.9988 | ortholo | no | 1163.2 |
| acylglycerol lipas | MAGL | Monoacylglycerol Lipas | AT2G3942 ARALIP (<http://aralip.plantbiology.msu.edu/pathways/triacylglycerol_fatty_acid_degradati> | Sobic.008G00010 | yes | 56.77 | 54.11 | OTHER | 0.9997 OTHER 0.9997 | ortholo | no | 24.4 |
| acylglycerol lipas | MAGL | Monoacylglycerol Lipas | AT2G4763 ARALIP (<http://aralip.plantbiology.msu.edu/pathways/triacylglycerol_fatty_acid_degradati> | Sobic.005G00020 | no | 57.89 | 55.89 | OTHER | 0.9851 SP 0.7266 | no | no | nd |
| acylglycerol lipas | MAGL | Monoacylglycerol Lipas | AT2G4763 ARALIP (<http://aralip.plantbiology.msu.edu/pathways/triacylglycerol_fatty_acid_degradati> | Sobic.008G00020 | no | 39.75 | 50.60 | OTHER | 0.9851 OTHER 0.8272 | no | no | nd |
| acylglycerol lipas | MAGL | Monoacylglycerol Lipas | AT2G4763 ARALIP (<http://aralip.plantbiology.msu.edu/pathways/triacylglycerol_fatty_acid_degradati> | Sobic.003G34860 | no | 55.33 | 54.61 | OTHER | 0.9851 OTHER 0.9972 | no | no | nd |
| acylglycerol lipas | MAGL | Monoacylglycerol Lipas | AT2G4763 ARALIP (<http://aralip.plantbiology.msu.edu/pathways/triacylglycerol_fatty_acid_degradati> | Sobic.003G34870 | no | 45.42 | 45.27 | OTHER | 0.9851 OTHER 0.9964 | no | no | 26.0 |
| acylglycerol lipas | MAGL | Monoacylglycerol Lipas | AT2G4763 ARALIP (<http://aralip.plantbiology.msu.edu/pathways/triacylglycerol_fatty_acid_degradati> | Sobic.003G14940 | no | 74.48 | 66.08 | OTHER | 0.9851 OTHER 0.9955 | ortholo | no | 36.7 |
| acylglycerol lipas | MAGL | Monoacylglycerol Lipas | AT2G4763 ARALIP (<http://aralip.plantbiology.msu.edu/pathways/triacylglycerol_fatty_acid_degradati> | Sobic.009G11250 | bidirectiona | 68.8 | 65.13 | OTHER | 0.9851 OTHER 0.9896 | ortholo | no | 350.2 |
| acylglycerol lipas | MAGL | Monoacylglycerol Lipas | AT2G4763 ARALIP (<http://aralip.plantbiology.msu.edu/pathways/triacylglycerol_fatty_acid_degradati> | Sobic.005G00010 | no | 57.72 | 52.31 | OTHER | 0.9851 OTHER 0.9988 | no | no | 1163.2 |
| acylglycerol lipas | MAGL | Monoacylglycerol Lipas | AT2G4763 ARALIP (<http://aralip.plantbiology.msu.edu/pathways/triacylglycerol_fatty_acid_degradati> | Sobic.008G00010 | no | 57.91 | 52.15 | OTHER | 0.9851 OTHER 0.9997 | no | no | 24.4 |
| acylglycerol lipas acylglycerol lipas | TAGL TAGL | Triacylglycerol Lipas Triacylglycerol Lipas | AT3G1436 ARALIP (<http://aralip.plantbiology.msu.edu/pathways/triacylglycerol_fatty_acid_degradati> AT3G1436 ARALIP (<http://aralip.plantbiology.msu.edu/pathways/triacylglycerol_fatty_acid_degradati> | Sobic.009G04810 Sobic.006G24810 | yes  no | 34.15  36.46 | 35.07  33.62 | OTHER OTHER | 1.0000 OTHER 0.9985  1.0000 OTHER 0.9270 | no no | no no | 11.1  nd |
| acylglycerol lipas | TAGL | Triacylglycerol Lipas | AT3G1436 ARALIP (<http://aralip.plantbiology.msu.edu/pathways/triacylglycerol_fatty_acid_degradati> | Sobic.004G31000 | no | 41.65 | 42.41 | OTHER | 1.0000 OTHER 0.9995 | no | no | 2.4 |
| acylglycerol lipas | TAGL | Triacylglycerol Lipas | AT3G1436 ARALIP (<http://aralip.plantbiology.msu.edu/pathways/triacylglycerol_fatty_acid_degradati> | Sobic.010G08110 | yes | 41.21 | 42.34 | OTHER | 1.0000 OTHER 0.9983 | no | no | 4.1 |
| acylglycerol lipas | MAGL | Monoacylglycerol Lipas | AT3G5518 ARALIP (<http://aralip.plantbiology.msu.edu/pathways/triacylglycerol_fatty_acid_degradati> | Sobic.005G00020 | bidirectiona | 61.75 | 60.07 | OTHER | 0.9988 SP 0.7266 | ortholo | no | nd |
| acylglycerol lipas | MAGL | Monoacylglycerol Lipas | AT3G5518 ARALIP (<http://aralip.plantbiology.msu.edu/pathways/triacylglycerol_fatty_acid_degradati> | Sobic.008G00020 | yes | 44.72 | 55.42 | OTHER | 0.9988 OTHER 0.8272 | ortholo | no | nd |
| acylglycerol lipas | MAGL | Monoacylglycerol Lipas | AT3G5518 ARALIP (<http://aralip.plantbiology.msu.edu/pathways/triacylglycerol_fatty_acid_degradati> | Sobic.003G34860 | no | 56.44 | 56.44 | OTHER | 0.9988 OTHER 0.9972 | ortholo | no | nd |
| acylglycerol lipas | MAGL | Monoacylglycerol Lipas | AT3G5518 ARALIP (<http://aralip.plantbiology.msu.edu/pathways/triacylglycerol_fatty_acid_degradati> | Sobic.003G34870 | no | 49.66 | 49.32 | OTHER | 0.9988 OTHER 0.9964 | ortholo | no | 26.0 |
| acylglycerol lipas | MAGL | Monoacylglycerol Lipas | AT3G5518 ARALIP (<http://aralip.plantbiology.msu.edu/pathways/triacylglycerol_fatty_acid_degradati> | Sobic.003G14940 | no | 59.66 | 55.63 | OTHER | 0.9988 OTHER 0.9955 | no | no | 36.7 |
| acylglycerol lipas | MAGL | Monoacylglycerol Lipas | AT3G5518 ARALIP (<http://aralip.plantbiology.msu.edu/pathways/triacylglycerol_fatty_acid_degradati> | Sobic.009G11250 | no | 57.59 | 54.17 | OTHER | 0.9988 OTHER 0.9896 | no | no | 350.2 |
| acylglycerol lipas | MAGL | Monoacylglycerol Lipas | AT3G5518 ARALIP (<http://aralip.plantbiology.msu.edu/pathways/triacylglycerol_fatty_acid_degradati> | Sobic.005G00010 | no | 57.14 | 55.70 | OTHER | 0.9988 OTHER 0.9988 | ortholo | no | 1163.2 |
| acylglycerol lipas | MAGL | Monoacylglycerol Lipas | AT3G5518 ARALIP (<http://aralip.plantbiology.msu.edu/pathways/triacylglycerol_fatty_acid_degradati> | Sobic.008G00010 | no | 56.95 | 54.66 | OTHER | 0.9988 OTHER 0.9997 | ortholo | no | 24.4 |
| acylglycerol lipas | MAGL | Monoacylglycerol Lipas | AT3G5519 ARALIP (<http://aralip.plantbiology.msu.edu/pathways/triacylglycerol_fatty_acid_degradati> | Sobic.005G00020 | yes | 52.94 | 51.01 | OTHER | 0.9993 SP 0.7266 | ortholo | no | nd |
| acylglycerol lipas | MAGL | Monoacylglycerol Lipas | AT3G5519 ARALIP (<http://aralip.plantbiology.msu.edu/pathways/triacylglycerol_fatty_acid_degradati> | Sobic.008G00020 | no | 39.81 | 50.00 | OTHER | 0.9993 OTHER 0.8272 | ortholo | no | nd |
| acylglycerol lipas | MAGL | Monoacylglycerol Lipas | AT3G5519 ARALIP (<http://aralip.plantbiology.msu.edu/pathways/triacylglycerol_fatty_acid_degradati> | Sobic.003G34860 | no | 52.41 | 51.62 | OTHER | 0.9993 OTHER 0.9972 | ortholo | no | nd |
| acylglycerol lipas | MAGL | Monoacylglycerol Lipas | AT3G5519 ARALIP (<http://aralip.plantbiology.msu.edu/pathways/triacylglycerol_fatty_acid_degradati> | Sobic.003G34870 | no | 45.95 | 46.64 | OTHER | 0.9993 OTHER 0.9964 | ortholo | no | 26.0 |
| acylglycerol lipas | MAGL | Monoacylglycerol Lipas | AT3G5519 ARALIP (<http://aralip.plantbiology.msu.edu/pathways/triacylglycerol_fatty_acid_degradati> | Sobic.003G14940 | no | 50 | 47.27 | OTHER | 0.9993 OTHER 0.9955 | no | no | 36.7 |
| acylglycerol lipas | MAGL | Monoacylglycerol Lipas | AT3G5519 ARALIP (<http://aralip.plantbiology.msu.edu/pathways/triacylglycerol_fatty_acid_degradati> | Sobic.009G11250 | no | 51.72 | 48.09 | OTHER | 0.9993 OTHER 0.9896 | no | no | 350.2 |
| acylglycerol lipas | MAGL | Monoacylglycerol Lipas | AT3G5519 ARALIP (<http://aralip.plantbiology.msu.edu/pathways/triacylglycerol_fatty_acid_degradati> | Sobic.005G00010 | no | 49.15 | 45.69 | OTHER | 0.9993 OTHER 0.9988 | ortholo | no | 1163.2 |
| acylglycerol lipas | MAGL | Monoacylglycerol Lipas | AT3G5519 ARALIP (<http://aralip.plantbiology.msu.edu/pathways/triacylglycerol_fatty_acid_degradati> | Sobic.008G00010 | no | 48.63 | 44.48 | OTHER | 0.9993 OTHER 0.9997 | ortholo | no | 24.4 |
| acylglycerol lipas | TAGL | Triacylglycerol Lipas | AT3G5714 ARALIP (<http://aralip.plantbiology.msu.edu/pathways/triacylglycerol_fatty_acid_degradati> | Sobic.001G04190 | no | 54.83 | 57.90 | OTHER | 0.9969 OTHER 0.9779 | ortholo | no | 603.1 |
| acylglycerol lipas | TAGL | Triacylglycerol Lipas | AT3G5714 ARALIP (<http://aralip.plantbiology.msu.edu/pathways/triacylglycerol_fatty_acid_degradati> | Sobic.003G30420 | yes | 59.86 | 64.55 | OTHER | 0.9969 OTHER 0.9904 | ortholo | no | 342.4 |

| acylglycerol lipas | MAGL | Monoacylglycerol Lipas | AT3G6286 ARALIP (<http://aralip.plantbiology.msu.edu/pathways/triacylglycerol_fatty_acid_degradati> | Sobic.005G00020 | no | 55.52 | 53.04 | OTHER | 0.9959 SP 0.7266 | | | no | no | nd | |
| --- | --- | --- | --- | --- | --- | --- | --- | --- | --- | --- | --- | --- | --- | --- | --- |
| acylglycerol lipas | MAGL | Monoacylglycerol Lipas | AT3G6286 ARALIP (<http://aralip.plantbiology.msu.edu/pathways/triacylglycerol_fatty_acid_degradati> | Sobic.008G00020 | no | 38.32 | 48.79 | OTHER | 0.9959 OTHER 0.8272 | | | no | no | nd | |
| acylglycerol lipas | MAGL | Monoacylglycerol Lipas | AT3G6286 ARALIP (<http://aralip.plantbiology.msu.edu/pathways/triacylglycerol_fatty_acid_degradati> | Sobic.003G34860 | no | 54.52 | 53.47 | OTHER | 0.9959 OTHER 0.9972 | | | no | no | nd | |
| acylglycerol lipas | MAGL | Monoacylglycerol Lipas | AT3G6286 ARALIP (<http://aralip.plantbiology.msu.edu/pathways/triacylglycerol_fatty_acid_degradati> | Sobic.003G34870 | no | 46.26 | 46.92 | OTHER | 0.9959 OTHER 0.9964 | | | no | no | 26.0 | |
| acylglycerol lipas | MAGL | Monoacylglycerol Lipas | AT3G6286 ARALIP (<http://aralip.plantbiology.msu.edu/pathways/triacylglycerol_fatty_acid_degradati> | Sobic.003G14940 | yes | 67.05 | 67.65 | OTHER | 0.9959 OTHER 0.9955 | | | ortholo | no | 36.7 | |
| acylglycerol lipas | MAGL | Monoacylglycerol Lipas | AT3G6286 ARALIP (<http://aralip.plantbiology.msu.edu/pathways/triacylglycerol_fatty_acid_degradati> | Sobic.009G11250 | yes | 68.14 | 66.28 | OTHER | 0.9959 OTHER 0.9896 | | | LDO | no | 350.2 | |
| acylglycerol lipas | MAGL | Monoacylglycerol Lipas | AT3G6286 ARALIP (<http://aralip.plantbiology.msu.edu/pathways/triacylglycerol_fatty_acid_degradati> | Sobic.005G00010 | no | 56.76 | 52.50 | OTHER | 0.9959 OTHER 0.9988 | | | no | no | 1163.2 | |
| acylglycerol lipas | MAGL | Monoacylglycerol Lipas | AT3G6286 ARALIP (<http://aralip.plantbiology.msu.edu/pathways/triacylglycerol_fatty_acid_degradati> | Sobic.008G00010 | no | 53.61 | 52.65 | OTHER | 0.9959 OTHER 0.9997 | | | no | no | 24.4 | |
| acylglycerol lipas | TAGL | Triacylglycerol Lipas | AT5G0404 ARALIP (<http://aralip.plantbiology.msu.edu/pathways/triacylglycerol_fatty_acid_degradati> | Sobic.001G04190 | yes | 58.42 | 60.15 | OTHER | 0.9986 OTHER 0.9779 | | | ortholo | no | 603.1 | |
| acylglycerol lipas | TAGL | Triacylglycerol Lipas | AT5G0404 ARALIP (<http://aralip.plantbiology.msu.edu/pathways/triacylglycerol_fatty_acid_degradati> | Sobic.003G30420 | bidirectiona | 65.07 | 66.04 | OTHER | 0.9986 OTHER 0.9904 | | | LDO | no | 342.4 | |
| acylglycerol lipas | MAGL | Monoacylglycerol Lipas | AT5G1165 ARALIP (<http://aralip.plantbiology.msu.edu/pathways/triacylglycerol_fatty_acid_degradati> | Sobic.003G02890 | bidirectiona | 72.33 | 62.34 | OTHER | 0.9839 OTHER 0.9936 | | | LDO | no | 11746.5 | |
| acylglycerol lipas | MAGL | Monoacylglycerol Lipas | AT5G1165 ARALIP (<http://aralip.plantbiology.msu.edu/pathways/triacylglycerol_fatty_acid_degradati> | Sobic.002G19400 | no | 61.84 | 55.46 | OTHER | 0.9839 cTP 0.6087 | | | no | no | 532.6 | |
| acylglycerol lipas | MAGL | Monoacylglycerol Lipas | AT5G1165 ARALIP (<http://aralip.plantbiology.msu.edu/pathways/triacylglycerol_fatty_acid_degradati> | Sobic.001G11170 | no | 57.04 | 47.77 | OTHER | 0.9839 OTHER 0.6885 | | | no | no | 37493.1 | |
| acylglycerol lipas | TAGL | Triacylglycerol Lipas | AT5G1418 ARALIP (<http://aralip.plantbiology.msu.edu/pathways/triacylglycerol_fatty_acid_degradati> | Sobic.002G14070 | no | 41.51 | 40.10 | SP | 0.0013 SP 1.0000 | | | no | no | 2727.3 | |
| acylglycerol lipas | TAGL | Triacylglycerol Lipas | AT5G1418 ARALIP (<http://aralip.plantbiology.msu.edu/pathways/triacylglycerol_fatty_acid_degradati> | Sobic.010G20000 | yes | 49.74 | 47.87 | SP | 0.0013 SP 0.9888 | | | no | no | nd | |
| acylglycerol lipas | TAGL | Triacylglycerol Lipas | AT5G1418 ARALIP (<http://aralip.plantbiology.msu.edu/pathways/triacylglycerol_fatty_acid_degradati> | Sobic.006G03750 | bidirectiona | 50.8 | 48.40 | SP | 0.0013 SP 0.9984 | | | no | no | nd | |
| acylglycerol lipas | TAGL | Triacylglycerol Lipas | AT5G1418 ARALIP (<http://aralip.plantbiology.msu.edu/pathways/triacylglycerol_fatty_acid_degradati> | Sobic.007G19480 | yes | 55.41 | 55.74 | SP | 0.0013 OTHER 0.7751 | | | LDO | no | 7.3 | |
| acylglycerol lipas | TAGL | Triacylglycerol Lipas | AT5G1493 ARALIP (<http://aralip.plantbiology.msu.edu/pathways/triacylglycerol_fatty_acid_degradati> | N/A | N/A | ≤ 42.5 | all < 20 | OTHER | 0.8545 N/A N/A | | | no | no | nd | |
| acylglycerol lipas | MAGL; LPLA | Monoacylglycerol Lipase; Lipoprotein Lipa | AT5G1498 ARALIP (<http://aralip.plantbiology.msu.edu/pathways/triacylglycerol_fatty_acid_degradati> | Sobic.003G15020 | yes | 57.91 | 60.92 | OTHER | 0.9535 | OTHER | 0.9888 | ortholo | no |  | 806.4 |
| acylglycerol lipas | MAGL; LPLA | Monoacylglycerol Lipase; Lipoprotein Lipa | AT5G1498 ARALIP (<http://aralip.plantbiology.msu.edu/pathways/triacylglycerol_fatty_acid_degradati> | Sobic.004G01260 | yes | 46.43 | 49.31 | OTHER | 0.9535 | OTHER | 0.9507 | no | no | nd |  |
| acylglycerol lipas | MAGL | Monoacylglycerol Lipas | AT5G1612 ARALIP (<http://aralip.plantbiology.msu.edu/pathways/triacylglycerol_fatty_acid_degradati> | Sobic.001G27040 | bidirectiona | 65.66 | 61.39 | OTHER | 0.9046 | OTHER | 0.9983 | no | no |  | 894.0 |
| acylglycerol lipas | MAGL | Monoacylglycerol Lipas | AT5G1612 ARALIP (<http://aralip.plantbiology.msu.edu/pathways/triacylglycerol_fatty_acid_degradati> | Sobic.003G26150 | no | 57.48 | 54.25 | OTHER | 0.9046 | cTP | 0.7075 | no | no |  | 667.3 |
| acylglycerol lipas | MAGL | Monoacylglycerol Lipas | AT5G1612 ARALIP (<http://aralip.plantbiology.msu.edu/pathways/triacylglycerol_fatty_acid_degradati> | Sobic.003G14940 | no | 50 | 47.67 | OTHER | 0.9046 | OTHER | 0.9955 | no | no |  | 36.7 |
| acylglycerol lipas | MAGL | Monoacylglycerol Lipas | AT5G1612 ARALIP (<http://aralip.plantbiology.msu.edu/pathways/triacylglycerol_fatty_acid_degradati> | Sobic.009G11250 | no | 48.28 | 46.36 | OTHER | 0.9046 | OTHER | 0.9896 | no | no |  | 350.2 |
| acylglycerol lipas | MAGL | Monoacylglycerol Lipas | AT5G1612 ARALIP (<http://aralip.plantbiology.msu.edu/pathways/triacylglycerol_fatty_acid_degradati> | Sobic.005G00010 | no | 46.23 | 42.45 | OTHER | 0.9046 | OTHER | 0.9988 | no | no |  | 1163.2 |
| acylglycerol lipas | MAGL | Monoacylglycerol Lipas | AT5G1612 ARALIP (<http://aralip.plantbiology.msu.edu/pathways/triacylglycerol_fatty_acid_degradati> | Sobic.008G00010 | no | 45.7 | 42.17 | OTHER | 0.9046 | OTHER | 0.9997 | no | no |  | 24.4 |
| acylglycerol lipas | TAGL | Triacylglycerol Lipas | AT5G1863 ARALIP (<http://aralip.plantbiology.msu.edu/pathways/triacylglycerol_fatty_acid_degradati> | Sobic.001G37320 | no | 56.21 | 49.57 | SP | 0.0000 | SP | 0.9826 | ortholo | no |  | 219.7 |
| acylglycerol lipas | TAGL | Triacylglycerol Lipas | AT5G1863 ARALIP (<http://aralip.plantbiology.msu.edu/pathways/triacylglycerol_fatty_acid_degradati> | Sobic.002G40950 | no | 56.39 | 52.59 | SP | 0.0000 | SP | 0.6420 | ortholo | no |  | 308.0 |
| acylglycerol lipas | TAGL | Triacylglycerol Lipas | AT5G1863 ARALIP (<http://aralip.plantbiology.msu.edu/pathways/triacylglycerol_fatty_acid_degradati> | Sobic.005G20380 | no | 59.55 | 55.49 | SP | 0.0000 | SP | 0.9997 | ortholo | no |  | 740.0 |
| acylglycerol lipas | TAGL | Triacylglycerol Lipas | AT5G1863 ARALIP (<http://aralip.plantbiology.msu.edu/pathways/triacylglycerol_fatty_acid_degradati> | Sobic.003G41870 | no | 58.75 | 55.94 | SP | 0.0000 | SP | 0.9997 | ortholo | no |  | 500.8 |
| acylglycerol lipas | TAGL | Triacylglycerol Lipas | AT5G1863 ARALIP (<http://aralip.plantbiology.msu.edu/pathways/triacylglycerol_fatty_acid_degradati> | Sobic.009G11750 | no | 57.1 | 55.23 | SP | 0.0000 | SP | 0.9992 | ortholo | no |  | 644.7 |
| acylglycerol lipas | TAGL | Triacylglycerol Lipas | AT5G1864 ARALIP (<http://aralip.plantbiology.msu.edu/pathways/triacylglycerol_fatty_acid_degradati> | Sobic.001G37320 | yes | 57.77 | 51.16 | SP | 0.0000 | SP | 0.9826 | no | no |  | 219.7 |
| acylglycerol lipas | TAGL | Triacylglycerol Lipas | AT5G1864 ARALIP (<http://aralip.plantbiology.msu.edu/pathways/triacylglycerol_fatty_acid_degradati> | Sobic.002G40950 | yes | 57.19 | 52.16 | SP | 0.0000 | SP | 0.6420 | no | no |  | 308.0 |
| acylglycerol lipas | TAGL | Triacylglycerol Lipas | AT5G1864 ARALIP (<http://aralip.plantbiology.msu.edu/pathways/triacylglycerol_fatty_acid_degradati> | Sobic.005G20380 | yes | 59.38 | 56.23 | SP | 0.0000 | SP | 0.9997 | no | no |  | 740.0 |
| acylglycerol lipas acylglycerol lipas | TAGL TAGL | Triacylglycerol Lipas Triacylglycerol Lipas | AT5G1864 ARALIP (<http://aralip.plantbiology.msu.edu/pathways/triacylglycerol_fatty_acid_degradati> AT5G1864 ARALIP (<http://aralip.plantbiology.msu.edu/pathways/triacylglycerol_fatty_acid_degradati> | Sobic.003G41870 Sobic.009G11750 | bidirectiona  yes | 58.26  59.64 | 56.10  58.60 | SP SP | 0.0000  0.0000 | SP SP | 0.9997  0.9992 | no no | no no |  | 500.8  644.7 |
| acylglycerol lipas | MAGL; LPLA | Monoacylglycerol Lipase; Lipoprotein Lipa | AT5G1929 ARALIP (<http://aralip.plantbiology.msu.edu/pathways/triacylglycerol_fatty_acid_degradati> | Sobic.003G15020 | bidirectiona | 62.03 | 60.92 | OTHER | 0.9953 OTHER 0.9888 | | | LDO | no |  | 806.4 |
| acylglycerol lipas | MAGL; LPLA | Monoacylglycerol Lipase; Lipoprotein Lipa | AT5G1929 ARALIP (<http://aralip.plantbiology.msu.edu/pathways/triacylglycerol_fatty_acid_degradati> | Sobic.004G01260 | no | 47.86 | 49.31 | OTHER | 0.9953 OTHER 0.9507 | | | no | no | nd |  |
| acylglycerol lipas | TAGL | Triacylglycerol Lipas | AT5G4293 ARALIP (<http://aralip.plantbiology.msu.edu/pathways/triacylglycerol_fatty_acid_degradati> | Sobic.009G04810 | no | 31.42 | 34.92 | OTHER | 0.9997 OTHER 0.9985 | | | no | no |  | 11.1 |
| acylglycerol lipas | TAGL | Triacylglycerol Lipas | AT5G4293 ARALIP (<http://aralip.plantbiology.msu.edu/pathways/triacylglycerol_fatty_acid_degradati> | Sobic.006G24810 | yes | 37.53 | 38.05 | OTHER | 0.9997 OTHER 0.9270 | | | ortholo | no | nd |  |
| acylglycerol lipas | TAGL | Triacylglycerol Lipas | AT5G4293 ARALIP (<http://aralip.plantbiology.msu.edu/pathways/triacylglycerol_fatty_acid_degradati> | Sobic.004G31000 | bidirectiona | 47.38 | 47.49 | OTHER | 0.9997 OTHER 0.9995 | | | LDO | no |  | 2.4 |
| acylglycerol lipas | TAGL | Triacylglycerol Lipas | AT5G4293 ARALIP (<http://aralip.plantbiology.msu.edu/pathways/triacylglycerol_fatty_acid_degradati> | Sobic.010G08110 | yes | 46.72 | 46.22 | OTHER | 0.9997 OTHER 0.9983 | | | ortholo | no |  | 4.1 |
| acylglycerol lipas | TAGL | Triacylglycerol Lipas | AT5G6705 ARALIP (<http://aralip.plantbiology.msu.edu/pathways/triacylglycerol_fatty_acid_degradati> | Sobic.009G04810 | no | 31.99 | 34.91 | OTHER | 1.0000 OTHER 0.9985 | | | no | no |  | 11.1 |
| acylglycerol lipas | TAGL | Triacylglycerol Lipas | AT5G6705 ARALIP (<http://aralip.plantbiology.msu.edu/pathways/triacylglycerol_fatty_acid_degradati> | Sobic.006G24810 | no | 38.24 | 38.29 | OTHER | 1.0000 OTHER 0.9270 | | | ortholo | no | nd |  |
| acylglycerol lipas | TAGL | Triacylglycerol Lipas | AT5G6705 ARALIP (<http://aralip.plantbiology.msu.edu/pathways/triacylglycerol_fatty_acid_degradati> | Sobic.004G31000 | yes | 45.61 | 44.04 | OTHER | 1.0000 OTHER 0.9995 | | | ortholo | no |  | 2.4 |
| acylglycerol lipas | TAGL | Triacylglycerol Lipas | AT5G6705 ARALIP (<http://aralip.plantbiology.msu.edu/pathways/triacylglycerol_fatty_acid_degradati> | Sobic.010G08110 | no | 45.17 | 45.81 | OTHER | 1.0000 OTHER 0.9983 | | | ortholo | no |  | 4.1 |
| beta-oxidatio | KAT1 | 3-ketoacyl-CoA thiolas | AT1G0471 PlantCyc (https://pmn.plantcyc.org/PLANT/NEW-IMAGE?type=PATHWAY&object=PWY-5138; https://pmn.plantcyc.org/PLANT/NEW-IMAGE?type=PATHWAY&object=PWY-51 | Sobic.004G34580 | yes | 75.56 | 76.75 | OTHER | 0.98551 OTHER 0.959741 | | | O | no |  | 9663.1 |
| beta-oxidatio | KAT1 | 3-ketoacyl-CoA thiolas | AT1G0471 PlantCyc (https://pmn.plantcyc.org/PLANT/NEW-IMAGE?type=PATHWAY&object=PWY-5138; https://pmn.plantcyc.org/PLANT/NEW-IMAGE?type=PATHWAY&object=PWY-51 | Sobic.001G23010 | no | 73.84 | 75.17 | OTHER | 0.98551 OTHER 0.985453 | | | O | no |  | 840.8 |
| beta-oxidatio | ACX | acyl-CoA oxidase | AT1G0629 PlantCyc (https://pmn.plantcyc.org/PLANT/NEW-IMAGE?type=PATHWAY&object=PWY-5138; https://pmn.plantcyc.org/PLANT/NEW-IMAGE?type=PATHWAY&object=PWY-51 | Sobic.003G35440 | bidirectiona | 66.41 | 65.19 | OTHER | 0.933914 OTHER 0.960818 | | | LDO | no |  | 1510.6 |
| beta-oxidatio | ACX | acyl-CoA oxidase | AT1G0631 PlantCyc (https://pmn.plantcyc.org/PLANT/NEW-IMAGE?type=PATHWAY&object=PWY-5138; https://pmn.plantcyc.org/PLANT/NEW-IMAGE?type=PATHWAY&object=PWY-51 | Sobic.003G35440 | yes | 63.98 | 62.52 | OTHER | 0.979317 OTHER 0.960818 | | | O | no |  | 1510.6 |
| beta-oxidatio | ACX | acyl-CoA oxidase | AT1G0631 PlantCyc (https://pmn.plantcyc.org/PLANT/NEW-IMAGE?type=PATHWAY&object=PWY-5138; https://pmn.plantcyc.org/PLANT/NEW-IMAGE?type=PATHWAY&object=PWY-51 | Sobic.005G18100 | no | 31.45 | 35.59 | OTHER | 0.979317 OTHER 0.960989 | | | no | no |  | 61.6 |
| beta-oxidatio | ECI | enoyl-CoA isomeras | AT1G6552 PlantCyc (https://pmn.plantcyc.org/PLANT/NEW-IMAGE?type=PATHWAY&object=PWY-5138; https://pmn.plantcyc.org/PLANT/NEW-IMAGE?type=PATHWAY&object=PWY-51 | Sobic.009G20710 | yes | 50.68 | 48.95 | OTHER | 0.998426 OTHER 0.884428 | | | O | no |  | 3436.4 |
| beta-oxidatio | ECI | enoyl-CoA isomeras | AT1G6552 PlantCyc (https://pmn.plantcyc.org/PLANT/NEW-IMAGE?type=PATHWAY&object=PWY-5138; https://pmn.plantcyc.org/PLANT/NEW-IMAGE?type=PATHWAY&object=PWY-51 | Sobic.003G02500 | yes | 42.24 | 42.67 | OTHER | 0.998426 OTHER 0.989532 | | | O | no |  | 98.6 |
| beta-oxidatio | ECI | enoyl-CoA isomeras | AT1G6552 PlantCyc (https://pmn.plantcyc.org/PLANT/NEW-IMAGE?type=PATHWAY&object=PWY-5138; https://pmn.plantcyc.org/PLANT/NEW-IMAGE?type=PATHWAY&object=PWY-51 | Sobic.003G02490 | yes | 44.26 | 40.93 | OTHER | 0.998426 OTHER 0.596359 | | | O | no |  | 22.9 |
| beta-oxidatio | ECHS | D-specific enoyl-CoA hydratase | AT1G7615 PlantCyc (https://pmn.plantcyc.org/PLANT/NEW-IMAGE?type=PATHWAY&object=PWY-5138; https://pmn.plantcyc.org/PLANT/NEW-IMAGE?type=PATHWAY&object=PWY-51 | Sobic.002G28420 | bidirectiona | 62.86 | 62.14 | OTHER | 0.999932 OTHER 0.998397 | | | LDO | no |  | 1192.9 |
| beta-oxidatio | ECHS | D-specific enoyl-CoA hydratase | AT1G7615 PlantCyc (https://pmn.plantcyc.org/PLANT/NEW-IMAGE?type=PATHWAY&object=PWY-5138; https://pmn.plantcyc.org/PLANT/NEW-IMAGE?type=PATHWAY&object=PWY-51 | Sobic.002G28440 | yes | 60.73 | 59.09 | OTHER | 0.999932 OTHER 0.887259 | | | O | no |  | 8.1 |
| beta-oxidatio | KAT2 | 3-ketoacyl-CoA thiolas | AT2G3315 PlantCyc (https://pmn.plantcyc.org/PLANT/NEW-IMAGE?type=PATHWAY&object=PWY-5138; https://pmn.plantcyc.org/PLANT/NEW-IMAGE?type=PATHWAY&object=PWY-51 | Sobic.004G34580 | bidirectiona | 76.72 | 76.84 | OTHER | 0.992267 OTHER 0.959741 | | | LDO | no |  | 9663.1 |
| beta-oxidatio | KAT2 | 3-ketoacyl-CoA thiolas | AT2G3315 PlantCyc (https://pmn.plantcyc.org/PLANT/NEW-IMAGE?type=PATHWAY&object=PWY-5138; https://pmn.plantcyc.org/PLANT/NEW-IMAGE?type=PATHWAY&object=PWY-51 | Sobic.001G23010 | yes | 76.36 | 76.36 | OTHER | 0.992267 OTHER 0.985453 | | | O | no |  | 840.8 |
| beta-oxidatio | LACS | 2;3;4-saturated fatty acyl-CoA synthetas | AT3G0597 PlantCyc (https://pmn.plantcyc.org/PLANT/NEW-IMAGE?type=PATHWAY&object=PWY-5138; https://pmn.plantcyc.org/PLANT/NEW-IMAGE?type=PATHWAY&object=PWY-51 | Sobic.008G01080 | yes | 75.15 | 73.34 | OTHER | 0.932893 OTHER 0.99399 | | | O | no |  | 1066.1 |
| beta-oxidatio | LACS | 2;3;4-saturated fatty acyl-CoA synthetas | AT3G0597 PlantCyc (https://pmn.plantcyc.org/PLANT/NEW-IMAGE?type=PATHWAY&object=PWY-5138; https://pmn.plantcyc.org/PLANT/NEW-IMAGE?type=PATHWAY&object=PWY-51 | Sobic.005G03310 | no | 73.72 | 70.75 | OTHER | 0.932893 OTHER 0.998167 | | | LDO | no |  | 42.9 |
| beta-oxidatio | ACX | acyl-CoA oxidase | AT3G0669 PlantCyc (https://pmn.plantcyc.org/PLANT/NEW-IMAGE?type=PATHWAY&object=PWY-5138; https://pmn.plantcyc.org/PLANT/NEW-IMAGE?type=PATHWAY&object=PWY-51 | Sobic.003G35440 | yes | 60.24 | 55.14 | OTHER | 0.963124 OTHER 0.960818 | | | no | no |  | 1510.6 |
| beta-oxidatio | HADH | 3-hydroxyacyl-CoA dehydrogenase / enoyl-CoA hydratas | AT3G0686 PlantCyc (https://pmn.plantcyc.org/PLANT/NEW-IMAGE?type=PATHWAY&object=PWY-5138; https://pmn.plantcyc.org/PLANT/NEW-IMAGE?type=PATHWAY&object=PWY-51 | Sobic.003G15830 | bidirectiona | 73.65 | 73.31 | OTHER | 0.999806 OTHER 0.999475 | | | LDO | no |  | 2416.7 |
| beta-oxidatio | HADH | 3-hydroxyacyl-CoA dehydrogenase / enoyl-CoA hydratas | AT3G0686 PlantCyc (https://pmn.plantcyc.org/PLANT/NEW-IMAGE?type=PATHWAY&object=PWY-5138; https://pmn.plantcyc.org/PLANT/NEW-IMAGE?type=PATHWAY&object=PWY-51 | Sobic.009G11140 | yes | 65.74 | 65.88 | OTHER | 0.999806 OTHER 0.937282 | | | O | no |  | 155.3 |
| beta-oxidatio | HADH | 3-hydroxyacyl-CoA dehydrogenase / enoyl-CoA hydratas | AT3G0686 PlantCyc (https://pmn.plantcyc.org/PLANT/NEW-IMAGE?type=PATHWAY&object=PWY-5138; https://pmn.plantcyc.org/PLANT/NEW-IMAGE?type=PATHWAY&object=PWY-51 | Sobic.004G12420 | no | 57.56 | 56.43 | OTHER | 0.999806 OTHER 0.980323 | | | no | no |  | 3678.0 |
| beta-oxidatio | HADH | 3-hydroxyacyl-CoA dehydrogenas | AT3G1529 PlantCyc (https://pmn.plantcyc.org/PLANT/NEW-IMAGE?type=PATHWAY&object=PWY-5138; https://pmn.plantcyc.org/PLANT/NEW-IMAGE?type=PATHWAY&object=PWY-51 | Sobic.003G32400 | bidirectiona | 70.55 | 70.07 | OTHER | 0.999578 OTHER 0.949587 | | | LDO | no |  | 3069.2 |
| beta-oxidatio | ACX | acyl-CoA oxidase | AT3G5184 PlantCyc (https://pmn.plantcyc.org/PLANT/NEW-IMAGE?type=PATHWAY&object=PWY-5138; https://pmn.plantcyc.org/PLANT/NEW-IMAGE?type=PATHWAY&object=PWY-51 | Sobic.003G06180 | yes | 78.42 | 74.42 | OTHER | 0.99753 OTHER 0.999783 | | | LDO | no |  | 618.1 |
| beta-oxidatio | ACX | acyl-CoA oxidase | AT3G5184 PlantCyc (https://pmn.plantcyc.org/PLANT/NEW-IMAGE?type=PATHWAY&object=PWY-5138; https://pmn.plantcyc.org/PLANT/NEW-IMAGE?type=PATHWAY&object=PWY-51 | Sobic.009G05730 | bidirectiona | 77.46 | 75.58 | OTHER | 0.99753 OTHER 0.997776 | | | O | no |  | 2688.7 |
| beta-oxidatio | ACX | acyl-CoA oxidase | AT3G5184 PlantCyc (https://pmn.plantcyc.org/PLANT/NEW-IMAGE?type=PATHWAY&object=PWY-5138; https://pmn.plantcyc.org/PLANT/NEW-IMAGE?type=PATHWAY&object=PWY-51 | Sobic.009G05720 | yes | 78.43 | 74.42 | OTHER | 0.99753 OTHER 0.9991 | | | O | no |  | 151.6 |
| beta-oxidatio | ACX | acyl-CoA oxidase | AT3G5184 PlantCyc (https://pmn.plantcyc.org/PLANT/NEW-IMAGE?type=PATHWAY&object=PWY-5138; https://pmn.plantcyc.org/PLANT/NEW-IMAGE?type=PATHWAY&object=PWY-51 | Sobic.006G01280 | yes | 63.68 | 60.83 | OTHER | 0.99753 cTP 0.055698 | | | O | no | nd |  |
| beta-oxidatio | ECI | enoyl-CoA isomeras | AT4G1443 PlantCyc (https://pmn.plantcyc.org/PLANT/NEW-IMAGE?type=PATHWAY&object=PWY-5138; https://pmn.plantcyc.org/PLANT/NEW-IMAGE?type=PATHWAY&object=PWY-51 | Sobic.009G20710 | yes | 49.36 | 50.86 | OTHER | 0.999348 OTHER 0.884428 | | | LDO | no |  | 3436.4 |
| beta-oxidatio | ECI | enoyl-CoA isomeras | AT4G1443 PlantCyc (https://pmn.plantcyc.org/PLANT/NEW-IMAGE?type=PATHWAY&object=PWY-5138; https://pmn.plantcyc.org/PLANT/NEW-IMAGE?type=PATHWAY&object=PWY-51 | Sobic.003G02500 | no | 44.49 | 46.75 | OTHER | 0.999348 OTHER 0.989532 | | | O | no |  | 98.6 |
| beta-oxidatio | ECI | enoyl-CoA isomeras | AT4G1443 PlantCyc (https://pmn.plantcyc.org/PLANT/NEW-IMAGE?type=PATHWAY&object=PWY-5138; https://pmn.plantcyc.org/PLANT/NEW-IMAGE?type=PATHWAY&object=PWY-51 | Sobic.003G02490 | no | 47.58 | 43.1 | OTHER | 0.999348 OTHER 0.596359 | | | O | no |  | 22.9 |
| beta-oxidatio | ECI | enoyl-CoA isomeras | AT4G1444 PlantCyc (https://pmn.plantcyc.org/PLANT/NEW-IMAGE?type=PATHWAY&object=PWY-5138; https://pmn.plantcyc.org/PLANT/NEW-IMAGE?type=PATHWAY&object=PWY-51 | Sobic.009G20710 | bidirectiona | 47.66 | 47.62 | OTHER | 0.999667 OTHER 0.884428 | | | O | no |  | 3436.4 |
| beta-oxidatio | ECI | enoyl-CoA isomeras | AT4G1444 PlantCyc (https://pmn.plantcyc.org/PLANT/NEW-IMAGE?type=PATHWAY&object=PWY-5138; https://pmn.plantcyc.org/PLANT/NEW-IMAGE?type=PATHWAY&object=PWY-51 | Sobic.003G02500 | no | 42.11 | 44.16 | OTHER | 0.999667 OTHER 0.989532 | | | O | no |  | 98.6 |
| beta-oxidatio | ECI | enoyl-CoA isomeras | AT4G1444 PlantCyc (https://pmn.plantcyc.org/PLANT/NEW-IMAGE?type=PATHWAY&object=PWY-5138; https://pmn.plantcyc.org/PLANT/NEW-IMAGE?type=PATHWAY&object=PWY-51 | Sobic.003G02490 | no | 22.97 | 40.76 | OTHER | 0.999667 OTHER 0.596359 | | | O | no |  | 22.9 |
| beta-oxidatio | ACX1 | acyl-CoA oxidase | AT4G1676 PlantCyc (https://pmn.plantcyc.org/PLANT/NEW-IMAGE?type=PATHWAY&object=PWY-5138; https://pmn.plantcyc.org/PLANT/NEW-IMAGE?type=PATHWAY&object=PWY-51 | Sobic.010G04910 | bidirectiona | 77.95 | 77.53 | OTHER | 0.999977 OTHER 0.999955 | | | LDO | no |  | 8201.2 |
| beta-oxidatio | ACX1 | acyl-CoA oxidase | AT4G1676 PlantCyc (https://pmn.plantcyc.org/PLANT/NEW-IMAGE?type=PATHWAY&object=PWY-5138; https://pmn.plantcyc.org/PLANT/NEW-IMAGE?type=PATHWAY&object=PWY-51 | Sobic.010G00190 | yes | 77.51 | 77.41 | OTHER | 0.999977 OTHER 0.999876 | | | O | no |  | 403.8 |
| beta-oxidatio | ACX1 | acyl-CoA oxidase | AT4G1676 PlantCyc (https://pmn.plantcyc.org/PLANT/NEW-IMAGE?type=PATHWAY&object=PWY-5138; https://pmn.plantcyc.org/PLANT/NEW-IMAGE?type=PATHWAY&object=PWY-51 | Sobic.005G18100 | no | 24.71 | 33.45 | OTHER | 0.999977 OTHER 0.960989 | | | no | no |  | 61.6 |
| beta-oxidatio | ECHS | enoyl-CoA hydratase | AT4G2901 PlantCyc (https://pmn.plantcyc.org/PLANT/NEW-IMAGE?type=PATHWAY&object=PWY-5138; https://pmn.plantcyc.org/PLANT/NEW-IMAGE?type=PATHWAY&object=PWY-51 | Sobic.004G12420 | bidirectiona | 67.63 | 67.82 | OTHER | 0.998067 OTHER 0.980323 | | | LDO | no |  | 3678.0 |
| beta-oxidatio | ECHS | enoyl-CoA hydratase | AT4G2901 PlantCyc (https://pmn.plantcyc.org/PLANT/NEW-IMAGE?type=PATHWAY&object=PWY-5138; https://pmn.plantcyc.org/PLANT/NEW-IMAGE?type=PATHWAY&object=PWY-51 | Sobic.003G15830 | no | 55.92 | 55.62 | OTHER | 0.998067 OTHER 0.999475 | | | no | no |  | 2416.7 |
| beta-oxidatio | ECHS | enoyl-CoA hydratase | AT4G2901 PlantCyc (https://pmn.plantcyc.org/PLANT/NEW-IMAGE?type=PATHWAY&object=PWY-5138; https://pmn.plantcyc.org/PLANT/NEW-IMAGE?type=PATHWAY&object=PWY-51 | Sobic.009G11140 | no | 54.39 | 53.77 | OTHER | 0.998067 OTHER 0.937282 | | | no | no |  | 155.3 |
| beta-oxidatio | LACS | 2;3;4-saturated fatty acyl-CoA synthetas | AT5G2760 PlantCyc (https://pmn.plantcyc.org/PLANT/NEW-IMAGE?type=PATHWAY&object=PWY-5138; https://pmn.plantcyc.org/PLANT/NEW-IMAGE?type=PATHWAY&object=PWY-51 | Sobic.008G01080 | yes | 72.99 | 71.85 | OTHER | 0.998743 OTHER 0.99399 | | | O | no |  | 1066.1 |
| beta-oxidatio | LACS | 2;3;4-saturated fatty acyl-CoA synthetas | AT5G2760 PlantCyc (https://pmn.plantcyc.org/PLANT/NEW-IMAGE?type=PATHWAY&object=PWY-5138; https://pmn.plantcyc.org/PLANT/NEW-IMAGE?type=PATHWAY&object=PWY-51 | Sobic.005G03310 | yes | 72.4 | 69.8 | OTHER | 0.998743 OTHER 0.998167 | | | O | no |  | 42.9 |
| beta-oxidatio | ECHS | D-specific enoyl-CoA hydratase | AT5G4289 PlantCyc (https://pmn.plantcyc.org/PLANT/NEW-IMAGE?type=PATHWAY&object=PWY-5138; https://pmn.plantcyc.org/PLANT/NEW-IMAGE?type=PATHWAY&object=PWY-51 | Sobic.010G08190 | bidirectiona | 68.55 | 68.85 | OTHER | 0.998951 OTHER 0.991067 | | | LDO | no |  | 845.0 |
| beta-oxidatio | KAT5 | 3-keto-acyl-CoA thiolas | AT5G4888 PlantCyc (https://pmn.plantcyc.org/PLANT/NEW-IMAGE?type=PATHWAY&object=PWY-5138; https://pmn.plantcyc.org/PLANT/NEW-IMAGE?type=PATHWAY&object=PWY-51 | Sobic.004G34580 | yes | 70.26 | 70.2 | OTHER | 0.942628 OTHER 0.959741 | | | O | no |  | 9663.1 |
| beta-oxidatio | KAT5 | 3-keto-acyl-CoA thiolas | AT5G4888 PlantCyc (https://pmn.plantcyc.org/PLANT/NEW-IMAGE?type=PATHWAY&object=PWY-5138; https://pmn.plantcyc.org/PLANT/NEW-IMAGE?type=PATHWAY&object=PWY-51 | Sobic.001G23010 | no | 69.82 | 70.13 | OTHER | 0.942628 OTHER 0.985453 | | | O | no |  | 840.8 |
| beta-oxidatio | ECHS | D-specific enoyl-CoA hydratase | AT5G6033 PlantCyc (https://pmn.plantcyc.org/PLANT/NEW-IMAGE?type=PATHWAY&object=PWY-5138; https://pmn.plantcyc.org/PLANT/NEW-IMAGE?type=PATHWAY&object=PWY-51 | Sobic.006G01650 | bidirectiona | 49.34 | 46.39 | mTP | 0.994336 mTP 0.036327 | | | LDO | no |  | 52.4 |
| beta-oxidatio | ACX | acyl-CoA oxidase | AT5G6511 PlantCyc (https://pmn.plantcyc.org/PLANT/NEW-IMAGE?type=PATHWAY&object=PWY-5138; https://pmn.plantcyc.org/PLANT/NEW-IMAGE?type=PATHWAY&object=PWY-51 | Sobic.005G18100 | bidirectiona | 81.55 | 81.52 | OTHER | 0.999728 OTHER 0.960989 | | | LDO | no |  | 61.6 |
| beta-oxidatio | ACX | acyl-CoA oxidase | AT5G6511 PlantCyc (https://pmn.plantcyc.org/PLANT/NEW-IMAGE?type=PATHWAY&object=PWY-5138; https://pmn.plantcyc.org/PLANT/NEW-IMAGE?type=PATHWAY&object=PWY-51 | Sobic.010G00190 | no | 25.36 | 32.95 | OTHER | 0.999728 OTHER 0.999876 | | | no | no |  | 403.8 |
| beta-oxidatio | ACX | acyl-CoA oxidase | AT5G6511 PlantCyc (https://pmn.plantcyc.org/PLANT/NEW-IMAGE?type=PATHWAY&object=PWY-5138; https://pmn.plantcyc.org/PLANT/NEW-IMAGE?type=PATHWAY&object=PWY-51 | Sobic.010G04910 | no | 23.69 | 32.95 | OTHER | 0.999728 OTHER 0.999955 | | | no | no |  | 8201.2 |
| carbohydrate synthesi | SPS | sucrose phosphate synthase | AT1G0492 Manuscript (Kuczynski; C.; McCorkle; S.; Keereetaweep; J.; Shanklin; J.; & Schwender; J. (2022). An expanded role for the transcription factor WRINKLED1 in the biosynthesis of triacylglycerols during seed development. Frontiers in Plant Scienc | Sobic.004G06840 | no | 62.57 | 53.82 | OTHER | 1.0000 OTHER 1.0000 | | | no | no |  | 2249.5 |
| carbohydrate synthesi | SPS | sucrose phosphate synthase | AT1G0492 Manuscript (Kuczynski; C.; McCorkle; S.; Keereetaweep; J.; Shanklin; J.; & Schwender; J. (2022). An expanded role for the transcription factor WRINKLED1 in the biosynthesis of triacylglycerols during seed development. Frontiers in Plant Scienc | Sobic.010G20510 | no | 59.13 | 53.48 | OTHER | 1.0000 OTHER 1.0000 | | | no | no |  | 1205.4 |
| carbohydrate synthesi | SPS | sucrose phosphate synthase | AT1G0492 Manuscript (Kuczynski; C.; McCorkle; S.; Keereetaweep; J.; Shanklin; J.; & Schwender; J. (2022). An expanded role for the transcription factor WRINKLED1 in the biosynthesis of triacylglycerols during seed development. Frontiers in Plant Scienc | Sobic.009G23320 | no | 55.15 | 55.47 | OTHER | 1.0000 OTHER 1.0000 | | | no | no |  | 2229.7 |
| carbohydrate synthesi | SPS | sucrose phosphate synthase | AT1G0492 Manuscript (Kuczynski; C.; McCorkle; S.; Keereetaweep; J.; Shanklin; J.; & Schwender; J. (2022). An expanded role for the transcription factor WRINKLED1 in the biosynthesis of triacylglycerols during seed development. Frontiers in Plant Scienc | Sobic.003G40330 | bidirectiona | 67.12 | 68.89 | OTHER | 1.0000 OTHER 1.0000 | | | LDO | no | 15729.0 | |
| carbohydrate synthesi | SPS | sucrose phosphate synthase | AT1G0492 Manuscript (Kuczynski; C.; McCorkle; S.; Keereetaweep; J.; Shanklin; J.; & Schwender; J. (2022). An expanded role for the transcription factor WRINKLED1 in the biosynthesis of triacylglycerols during seed development. Frontiers in Plant Scienc | Sobic.005G08960 | no | 51.04 | 53.46 | OTHER | 1.0000 OTHER 0.9995 | | | no | no | 31292.3 | |
| carbohydrate synthesi | INV | invertase | AT1G3558 Manuscript (Kuczynski; C.; McCorkle; S.; Keereetaweep; J.; Shanklin; J.; & Schwender; J. (2022). An expanded role for the transcription factor WRINKLED1 in the biosynthesis of triacylglycerols during seed development. Frontiers in Plant Scienc | Sobic.004G02450 | no | 67.07 | 62.89 | OTHER | 0.9997 OTHER 0.9998 | | | no | no | nd | |
| carbohydrate synthesi | INV | invertase | AT1G3558 Manuscript (Kuczynski; C.; McCorkle; S.; Keereetaweep; J.; Shanklin; J.; & Schwender; J. (2022). An expanded role for the transcription factor WRINKLED1 in the biosynthesis of triacylglycerols during seed development. Frontiers in Plant Scienc | Sobic.005G05880 | no | 72.3 | 72.56 | OTHER | 0.9997 OTHER 0.9983 | | | ortholo | no | 1004.3 | |
| carbohydrate synthesi | INV | invertase | AT1G3558 Manuscript (Kuczynski; C.; McCorkle; S.; Keereetaweep; J.; Shanklin; J.; & Schwender; J. (2022). An expanded role for the transcription factor WRINKLED1 in the biosynthesis of triacylglycerols during seed development. Frontiers in Plant Scienc | Sobic.004G17270 | yes | 77.9 | 77.15 | OTHER | 0.9997 OTHER 0.9962 | | | ortholo | no | 798.0 | |
| carbohydrate synthesi | INV | invertase | AT1G3558 Manuscript (Kuczynski; C.; McCorkle; S.; Keereetaweep; J.; Shanklin; J.; & Schwender; J. (2022). An expanded role for the transcription factor WRINKLED1 in the biosynthesis of triacylglycerols during seed development. Frontiers in Plant Scienc | Sobic.004G25560 | no | 74.87 | 76 | OTHER | 0.9997 OTHER 0.9989 | | | ortholo | no | 3004.9 | |
| carbohydrate synthesi | INV | invertase | AT1G3558 Manuscript (Kuczynski; C.; McCorkle; S.; Keereetaweep; J.; Shanklin; J.; & Schwender; J. (2022). An expanded role for the transcription factor WRINKLED1 in the biosynthesis of triacylglycerols during seed development. Frontiers in Plant Scienc | Sobic.004G16380 | no | 59.91 | 53.21 | OTHER | 0.9997 OTHER 0.6917 | | | no | no | 3099.2 | |
| carbohydrate synthesi | INV | invertase | AT1G3558 Manuscript (Kuczynski; C.; McCorkle; S.; Keereetaweep; J.; Shanklin; J.; & Schwender; J. (2022). An expanded role for the transcription factor WRINKLED1 in the biosynthesis of triacylglycerols during seed development. Frontiers in Plant Scienc | Sobic.001G39160 | no | 59.01 | 52.53 | OTHER | 0.9997 mTP 0.9153 | | | no | no | 637.3 | |
| carbohydrate synthesi | INV | invertase | AT1G3558 Manuscript (Kuczynski; C.; McCorkle; S.; Keereetaweep; J.; Shanklin; J.; & Schwender; J. (2022). An expanded role for the transcription factor WRINKLED1 in the biosynthesis of triacylglycerols during seed development. Frontiers in Plant Scienc | Sobic.003G15380 | no | 57.17 | 52.24 | OTHER | 0.9997 cTP 0.5389 | | | no | no | 1159.7 | |
| carbohydrate synthesi | HXK | hexokinase | AT1G4784 Manuscript (Kuczynski; C.; McCorkle; S.; Keereetaweep; J.; Shanklin; J.; & Schwender; J. (2022). An expanded role for the transcription factor WRINKLED1 in the biosynthesis of triacylglycerols during seed development. Frontiers in Plant Scienc | Sobic.003G42120 | no | 46.58 | 47.36 | OTHER | 0.1582 OTHER 0.3410 | | | no | no | 649.4 | |
| carbohydrate synthesi | HXK | hexokinase | AT1G4784 Manuscript (Kuczynski; C.; McCorkle; S.; Keereetaweep; J.; Shanklin; J.; & Schwender; J. (2022). An expanded role for the transcription factor WRINKLED1 in the biosynthesis of triacylglycerols during seed development. Frontiers in Plant Scienc | Sobic.009G11910 | no | 46.2 | 44.63 | OTHER | 0.1582 SP 0.5806 | | | no | no | nd | |
| carbohydrate synthesi | HXK | hexokinase | AT1G4784 Manuscript (Kuczynski; C.; McCorkle; S.; Keereetaweep; J.; Shanklin; J.; & Schwender; J. (2022). An expanded role for the transcription factor WRINKLED1 in the biosynthesis of triacylglycerols during seed development. Frontiers in Plant Scienc | Sobic.003G03550 | no | 52.09 | 51.42 | OTHER | 0.1582 OTHER 0.9967 | | | no | no | 59.7 | |
| carbohydrate synthesi | HXK | hexokinase | AT1G4784 Manuscript (Kuczynski; C.; McCorkle; S.; Keereetaweep; J.; Shanklin; J.; & Schwender; J. (2022). An expanded role for the transcription factor WRINKLED1 in the biosynthesis of triacylglycerols during seed development. Frontiers in Plant Scienc | Sobic.009G06980 | no | 52.75 | 52.19 | OTHER | 0.1582 OTHER 0.9985 | | | no | no | 478.2 | |
| carbohydrate synthesi | HXK | hexokinase | AT1G4784 Manuscript (Kuczynski; C.; McCorkle; S.; Keereetaweep; J.; Shanklin; J.; & Schwender; J. (2022). An expanded role for the transcription factor WRINKLED1 in the biosynthesis of triacylglycerols during seed development. Frontiers in Plant Scienc | Sobic.003G28040 | no | 52.07 | 50.21 | OTHER | 0.1582 SP 0.4947 | | | no | no | 40.1 | |
| carbohydrate synthesi | HXK | hexokinase | AT1G4784 Manuscript (Kuczynski; C.; McCorkle; S.; Keereetaweep; J.; Shanklin; J.; & Schwender; J. (2022). An expanded role for the transcription factor WRINKLED1 in the biosynthesis of triacylglycerols during seed development. Frontiers in Plant Scienc | Sobic.003G29180 | no | 52.53 | 50.21 | OTHER | 0.1582 OTHER 0.8975 | | | no | no | 1074.2 | |
| carbohydrate synthesi | HXK | hexokinase | AT1G4784 Manuscript (Kuczynski; C.; McCorkle; S.; Keereetaweep; J.; Shanklin; J.; & Schwender; J. (2022). An expanded role for the transcription factor WRINKLED1 in the biosynthesis of triacylglycerols during seed development. Frontiers in Plant Scienc | Sobic.009G20350 | yes | 51.36 | 50.1 | OTHER | 0.1582 OTHER 0.5645 | | | no | no | 405.5 | |
| carbohydrate synthesi | HXK | hexokinase | AT1G5046 Manuscript (Kuczynski; C.; McCorkle; S.; Keereetaweep; J.; Shanklin; J.; & Schwender; J. (2022). An expanded role for the transcription factor WRINKLED1 in the biosynthesis of triacylglycerols during seed development. Frontiers in Plant Scienc | Sobic.003G42120 | yes | 51.35 | 60.78 | SP | 0.6359 OTHER 0.3410 | | | ortholo | no | 649.4 | |
| carbohydrate synthesi | HXK | hexokinase | AT1G5046 Manuscript (Kuczynski; C.; McCorkle; S.; Keereetaweep; J.; Shanklin; J.; & Schwender; J. (2022). An expanded role for the transcription factor WRINKLED1 in the biosynthesis of triacylglycerols during seed development. Frontiers in Plant Scienc | Sobic.009G11910 | bidirectiona | 52.38 | 51.83 | SP | 0.6359 SP 0.5806 | | | ortholo | no | nd | |
| carbohydrate synthesi | HXK | hexokinase | AT1G5046 Manuscript (Kuczynski; C.; McCorkle; S.; Keereetaweep; J.; Shanklin; J.; & Schwender; J. (2022). An expanded role for the transcription factor WRINKLED1 in the biosynthesis of triacylglycerols during seed development. Frontiers in Plant Scienc | Sobic.003G03550 | no | 49.78 | 50.11 | SP | 0.6359 OTHER 0.9967 | | | no | no | 59.7 | |
| carbohydrate synthesi | HXK | hexokinase | AT1G5046 Manuscript (Kuczynski; C.; McCorkle; S.; Keereetaweep; J.; Shanklin; J.; & Schwender; J. (2022). An expanded role for the transcription factor WRINKLED1 in the biosynthesis of triacylglycerols during seed development. Frontiers in Plant Scienc | Sobic.009G06980 | no | 50.9 | 50.55 | SP | 0.6359 OTHER 0.9985 | | | no | no | 478.2 | |
| carbohydrate synthesi | HXK | hexokinase | AT1G5046 Manuscript (Kuczynski; C.; McCorkle; S.; Keereetaweep; J.; Shanklin; J.; & Schwender; J. (2022). An expanded role for the transcription factor WRINKLED1 in the biosynthesis of triacylglycerols during seed development. Frontiers in Plant Scienc | Sobic.003G28040 | no | 50.43 | 50.1 | SP | 0.6359 SP 0.4947 | | | no | no | 40.1 | |
| carbohydrate synthesi | HXK | hexokinase | AT1G5046 Manuscript (Kuczynski; C.; McCorkle; S.; Keereetaweep; J.; Shanklin; J.; & Schwender; J. (2022). An expanded role for the transcription factor WRINKLED1 in the biosynthesis of triacylglycerols during seed development. Frontiers in Plant Scienc | Sobic.003G29180 | no | 50.98 | 49.59 | SP | 0.6359 OTHER 0.8975 | | | no | no | 1074.2 | |
| carbohydrate synthesi | HXK | hexokinase | AT1G5046 Manuscript (Kuczynski; C.; McCorkle; S.; Keereetaweep; J.; Shanklin; J.; & Schwender; J. (2022). An expanded role for the transcription factor WRINKLED1 in the biosynthesis of triacylglycerols during seed development. Frontiers in Plant Scienc | Sobic.009G20350 | no | 49.89 | 48.66 | SP | 0.6359 OTHER 0.5645 | | | no | no | 405.5 | |
| carbohydrate synthesi | SPP | sucrose-6-phosphate phosphohydrolas | AT1G5142 Manuscript (Kuczynski; C.; McCorkle; S.; Keereetaweep; J.; Shanklin; J.; & Schwender; J. (2022). An expanded role for the transcription factor WRINKLED1 in the biosynthesis of triacylglycerols during seed development. Frontiers in Plant Scienc | Sobic.004G15180 | yes | 60.1 | 58.43 | OTHER | 0.9995 OTHER 0.9945 | | | ortholo | no | 4765.5 | |
| carbohydrate synthesi | SPP | sucrose-6-phosphate phosphohydrolas | AT1G5142 Manuscript (Kuczynski; C.; McCorkle; S.; Keereetaweep; J.; Shanklin; J.; & Schwender; J. (2022). An expanded role for the transcription factor WRINKLED1 in the biosynthesis of triacylglycerols during seed development. Frontiers in Plant Scienc | Sobic.009G04090 | no | 53.52 | 53.68 | OTHER | 0.9995 OTHER 0.9999 | | | ortholo | no | 73.2 | |
| carbohydrate synthesi | SPP | sucrose-6-phosphate phosphohydrolas | AT1G5142 Manuscript (Kuczynski; C.; McCorkle; S.; Keereetaweep; J.; Shanklin; J.; & Schwender; J. (2022). An expanded role for the transcription factor WRINKLED1 in the biosynthesis of triacylglycerols during seed development. Frontiers in Plant Scienc | Sobic.009G04100 | no | 53.73 | 52.73 | OTHER | 0.9995 OTHER 1.0000 | | | ortholo | no | 3.5 | |
| carbohydrate synthesi | SUS6 | sucrose synthase | AT1G7337 Manuscript (Kuczynski; C.; McCorkle; S.; Keereetaweep; J.; Shanklin; J.; & Schwender; J. (2022). An expanded role for the transcription factor WRINKLED1 in the biosynthesis of triacylglycerols during seed development. Frontiers in Plant Scienc | Sobic.004G35760 | yes | 63.03 | 61.41 | OTHER | 0.9919 OTHER 0.9928 | | | ortholo | no | 13.6 | |
| carbohydrate synthesi | SUS6 | sucrose synthase | AT1G7337 Manuscript (Kuczynski; C.; McCorkle; S.; Keereetaweep; J.; Shanklin; J.; & Schwender; J. (2022). An expanded role for the transcription factor WRINKLED1 in the biosynthesis of triacylglycerols during seed development. Frontiers in Plant Scienc | Sobic.010G27670 | bidirectiona | 67.87 | 64.33 | OTHER | 0.9919 OTHER 0.8679 | | | ortholo | no | 7.2 | |
| carbohydrate synthesi | HXK | hexokinase | AT2G1986 Manuscript (Kuczynski; C.; McCorkle; S.; Keereetaweep; J.; Shanklin; J.; & Schwender; J. (2022). An expanded role for the transcription factor WRINKLED1 in the biosynthesis of triacylglycerols during seed development. Frontiers in Plant Scienc | Sobic.003G42120 | no | 50.99 | 52.57 | OTHER | 0.6653 OTHER 0.3410 | | | no | no | 649.4 | |
| carbohydrate synthesi | HXK | hexokinase | AT2G1986 Manuscript (Kuczynski; C.; McCorkle; S.; Keereetaweep; J.; Shanklin; J.; & Schwender; J. (2022). An expanded role for the transcription factor WRINKLED1 in the biosynthesis of triacylglycerols during seed development. Frontiers in Plant Scienc | Sobic.009G11910 | no | 46.71 | 47.24 | OTHER | 0.6653 SP 0.5806 | | | ortholo | no | nd | |
| carbohydrate synthesi | HXK | hexokinase | AT2G1986 Manuscript (Kuczynski; C.; McCorkle; S.; Keereetaweep; J.; Shanklin; J.; & Schwender; J. (2022). An expanded role for the transcription factor WRINKLED1 in the biosynthesis of triacylglycerols during seed development. Frontiers in Plant Scienc | Sobic.003G03550 | no | 61.12 | 58.42 | OTHER | 0.6653 OTHER 0.9967 | | | ortholo | no | 59.7 | |
| carbohydrate synthesi | HXK | hexokinase | AT2G1986 Manuscript (Kuczynski; C.; McCorkle; S.; Keereetaweep; J.; Shanklin; J.; & Schwender; J. (2022). An expanded role for the transcription factor WRINKLED1 in the biosynthesis of triacylglycerols during seed development. Frontiers in Plant Scienc | Sobic.009G06980 | no | 62.81 | 61.35 | OTHER | 0.6653 OTHER 0.9985 | | | ortholo | no | 478.2 | |
| carbohydrate synthesi | HXK | hexokinase | AT2G1986 Manuscript (Kuczynski; C.; McCorkle; S.; Keereetaweep; J.; Shanklin; J.; & Schwender; J. (2022). An expanded role for the transcription factor WRINKLED1 in the biosynthesis of triacylglycerols during seed development. Frontiers in Plant Scienc | Sobic.003G28040 | no | 63.3 | 62.04 | OTHER | 0.6653 SP 0.4947 | | | ortholo | no | 40.1 | |

| carbohydrate synthesi | HXK | hexokinase | AT2G1986 Manuscript (Kuczynski; C.; McCorkle; S.; Keereetaweep; J.; Shanklin; J.; & Schwender; J. (2022). An expanded role for the transcription factor WRINKLED1 in the biosynthesis of triacylglycerols during seed development. Frontiers in Plant Scienc | Sobic.003G29180 | no | 67.18 | 65.25 | OTHER | 0.6653 OTHER 0.8975 | | | ortholo | no | 1074.2 |
| --- | --- | --- | --- | --- | --- | --- | --- | --- | --- | --- | --- | --- | --- | --- |
| carbohydrate synthesi | HXK | hexokinase | AT2G1986 Manuscript (Kuczynski; C.; McCorkle; S.; Keereetaweep; J.; Shanklin; J.; & Schwender; J. (2022). An expanded role for the transcription factor WRINKLED1 in the biosynthesis of triacylglycerols during seed development. Frontiers in Plant Scienc | Sobic.009G20350 | yes | 66.59 | 65.24 | OTHER | 0.6653 OTHER 0.5645 | | | ortholo | no | 405.5 |
| carbohydrate synthesi | SPP | sucrose-6-phosphate phosphohydrolas | AT2G3584 Manuscript (Kuczynski; C.; McCorkle; S.; Keereetaweep; J.; Shanklin; J.; & Schwender; J. (2022). An expanded role for the transcription factor WRINKLED1 in the biosynthesis of triacylglycerols during seed development. Frontiers in Plant Scienc | Sobic.004G15180 | bidirectiona | 65.85 | 63.9 | OTHER | 0.9993 OTHER 0.9945 | | | LDO | no | 4765.5 |
| carbohydrate synthesi | SPP | sucrose-6-phosphate phosphohydrolas | AT2G3584 Manuscript (Kuczynski; C.; McCorkle; S.; Keereetaweep; J.; Shanklin; J.; & Schwender; J. (2022). An expanded role for the transcription factor WRINKLED1 in the biosynthesis of triacylglycerols during seed development. Frontiers in Plant Scienc | Sobic.009G04090 | yes | 56.94 | 57.01 | OTHER | 0.9993 OTHER 0.9999 | | | ortholo | no | 73.2 |
| carbohydrate synthesi | SPP | sucrose-6-phosphate phosphohydrolas | AT2G3584 Manuscript (Kuczynski; C.; McCorkle; S.; Keereetaweep; J.; Shanklin; J.; & Schwender; J. (2022). An expanded role for the transcription factor WRINKLED1 in the biosynthesis of triacylglycerols during seed development. Frontiers in Plant Scienc | Sobic.009G04100 | yes | 57.04 | 55.58 | OTHER | 0.9993 OTHER 1.0000 | | | ortholo | no | 3.5 |
| carbohydrate synthesi | UGP | UDP-glucose pyrophosphorylas | AT3G0325 Manuscript (Kuczynski; C.; McCorkle; S.; Keereetaweep; J.; Shanklin; J.; & Schwender; J. (2022). An expanded role for the transcription factor WRINKLED1 in the biosynthesis of triacylglycerols during seed development. Frontiers in Plant Scienc | Sobic.002G29120 | no | 83.01 | 82.09 | OTHER | 0.9991 OTHER 0.9994 | | | LDO | no | 54425.2 |
| carbohydrate synthesi | UGP | UDP-glucose pyrophosphorylas | AT3G0325 Manuscript (Kuczynski; C.; McCorkle; S.; Keereetaweep; J.; Shanklin; J.; & Schwender; J. (2022). An expanded role for the transcription factor WRINKLED1 in the biosynthesis of triacylglycerols during seed development. Frontiers in Plant Scienc | Sobic.004G01350 | yes | 83.44 | 82.87 | OTHER | 0.9991 OTHER 0.9998 | | | ortholo | no | nd |
| carbohydrate synthesi | HXK | hexokinase | AT3G2004 Manuscript (Kuczynski; C.; McCorkle; S.; Keereetaweep; J.; Shanklin; J.; & Schwender; J. (2022). An expanded role for the transcription factor WRINKLED1 in the biosynthesis of triacylglycerols during seed development. Frontiers in Plant Scienc | Sobic.003G42120 | no | 57.68 | 57.23 | SP | 0.4374 OTHER 0.3410 | | | LDO | no | 649.4 |
| carbohydrate synthesi | HXK | hexokinase | AT3G2004 Manuscript (Kuczynski; C.; McCorkle; S.; Keereetaweep; J.; Shanklin; J.; & Schwender; J. (2022). An expanded role for the transcription factor WRINKLED1 in the biosynthesis of triacylglycerols during seed development. Frontiers in Plant Scienc | Sobic.009G11910 | yes | 50.1 | 50.81 | SP | 0.4374 SP 0.5806 | | | ortholo | no | nd |
| carbohydrate synthesi | HXK | hexokinase | AT3G2004 Manuscript (Kuczynski; C.; McCorkle; S.; Keereetaweep; J.; Shanklin; J.; & Schwender; J. (2022). An expanded role for the transcription factor WRINKLED1 in the biosynthesis of triacylglycerols during seed development. Frontiers in Plant Scienc | Sobic.003G03550 | no | 48.8 | 47.95 | SP | 0.4374 OTHER 0.9967 | | | no | no | 59.7 |
| carbohydrate synthesi | HXK | hexokinase | AT3G2004 Manuscript (Kuczynski; C.; McCorkle; S.; Keereetaweep; J.; Shanklin; J.; & Schwender; J. (2022). An expanded role for the transcription factor WRINKLED1 in the biosynthesis of triacylglycerols during seed development. Frontiers in Plant Scienc | Sobic.009G06980 | no | 49.77 | 48.46 | SP | 0.4374 OTHER 0.9985 | | | no | no | 478.2 |
| carbohydrate synthesi | HXK | hexokinase | AT3G2004 Manuscript (Kuczynski; C.; McCorkle; S.; Keereetaweep; J.; Shanklin; J.; & Schwender; J. (2022). An expanded role for the transcription factor WRINKLED1 in the biosynthesis of triacylglycerols during seed development. Frontiers in Plant Scienc | Sobic.003G28040 | no | 50 | 48.47 | SP | 0.4374 SP 0.4947 | | | no | no | 40.1 |
| carbohydrate synthesi | HXK | hexokinase | AT3G2004 Manuscript (Kuczynski; C.; McCorkle; S.; Keereetaweep; J.; Shanklin; J.; & Schwender; J. (2022). An expanded role for the transcription factor WRINKLED1 in the biosynthesis of triacylglycerols during seed development. Frontiers in Plant Scienc | Sobic.003G29180 | no | 49.02 | 47.37 | SP | 0.4374 OTHER 0.8975 | | | no | no | 1074.2 |
| carbohydrate synthesi | HXK | hexokinase | AT3G2004 Manuscript (Kuczynski; C.; McCorkle; S.; Keereetaweep; J.; Shanklin; J.; & Schwender; J. (2022). An expanded role for the transcription factor WRINKLED1 in the biosynthesis of triacylglycerols during seed development. Frontiers in Plant Scienc | Sobic.009G20350 | no | 48.92 | 47.05 | SP | 0.4374 OTHER 0.5645 | | | no | no | 405.5 |
| carbohydrate synthesi | SUS4 | sucrose synthase | AT3G4319 Manuscript (Kuczynski; C.; McCorkle; S.; Keereetaweep; J.; Shanklin; J.; & Schwender; J. (2022). An expanded role for the transcription factor WRINKLED1 in the biosynthesis of triacylglycerols during seed development. Frontiers in Plant Scienc | Sobic.001G37830 | no | 68.96 | 68.53 | OTHER | 0.9993 OTHER 0.9998 | | | no | no | 748.1 |
| carbohydrate synthesi | SUS4 | sucrose synthase | AT3G4319 Manuscript (Kuczynski; C.; McCorkle; S.; Keereetaweep; J.; Shanklin; J.; & Schwender; J. (2022). An expanded role for the transcription factor WRINKLED1 in the biosynthesis of triacylglycerols during seed development. Frontiers in Plant Scienc | Sobic.001G34450 | yes | 74.35 | 74.13 | OTHER | 0.9993 OTHER 0.9997 | | | ortholo | no | 1317.2 |
| carbohydrate synthesi | SUS4 | sucrose synthase | AT3G4319 Manuscript (Kuczynski; C.; McCorkle; S.; Keereetaweep; J.; Shanklin; J.; & Schwender; J. (2022). An expanded role for the transcription factor WRINKLED1 in the biosynthesis of triacylglycerols during seed development. Frontiers in Plant Scienc | Sobic.010G07230 | bidirectiona | 75.5 | 75.28 | OTHER | 0.9993 mTP 0.6921 | | | ortholo | no | 2515.0 |
| carbohydrate synthesi | SPP | sucrose-6-phosphate phosphohydrolas | AT3G5234 Manuscript (Kuczynski; C.; McCorkle; S.; Keereetaweep; J.; Shanklin; J.; & Schwender; J. (2022). An expanded role for the transcription factor WRINKLED1 in the biosynthesis of triacylglycerols during seed development. Frontiers in Plant Scienc | Sobic.004G15180 | yes | 58.08 | 55.24 | OTHER | 0.9995 OTHER 0.9945 | | | ortholo | no | 4765.5 |
| carbohydrate synthesi | SPP | sucrose-6-phosphate phosphohydrolas | AT3G5234 Manuscript (Kuczynski; C.; McCorkle; S.; Keereetaweep; J.; Shanklin; J.; & Schwender; J. (2022). An expanded role for the transcription factor WRINKLED1 in the biosynthesis of triacylglycerols during seed development. Frontiers in Plant Scienc | Sobic.009G04090 | no | 54.87 | 52.14 | OTHER | 0.9995 OTHER 0.9999 | | | ortholo | no | 73.2 |
| carbohydrate synthesi | SPP | sucrose-6-phosphate phosphohydrolas | AT3G5234 Manuscript (Kuczynski; C.; McCorkle; S.; Keereetaweep; J.; Shanklin; J.; & Schwender; J. (2022). An expanded role for the transcription factor WRINKLED1 in the biosynthesis of triacylglycerols during seed development. Frontiers in Plant Scienc | Sobic.009G04100 | no | 52.39 | 50.48 | OTHER | 0.9995 OTHER 1.0000 | | | ortholo | no | 3.5 |
| carbohydrate synthesi | INV | invertase | AT3G5260 Manuscript (Kuczynski; C.; McCorkle; S.; Keereetaweep; J.; Shanklin; J.; & Schwender; J. (2022). An expanded role for the transcription factor WRINKLED1 in the biosynthesis of triacylglycerols during seed development. Frontiers in Plant Scienc | Sobic.004G16670 | bidirectiona | 60.73 | 58.27 | SP | 0.9967 SP 0.9991 | | | ortholo | no | 1173.8 |
| carbohydrate synthesi | INV | invertase | AT3G5260 Manuscript (Kuczynski; C.; McCorkle; S.; Keereetaweep; J.; Shanklin; J.; & Schwender; J. (2022). An expanded role for the transcription factor WRINKLED1 in the biosynthesis of triacylglycerols during seed development. Frontiers in Plant Scienc | Sobic.006G07056 | yes | 55.01 | 55.69 | SP | 0.9967 SP 0.9999 | | | ortholo | no | nd |
| carbohydrate synthesi | INV | invertase | AT3G5260 Manuscript (Kuczynski; C.; McCorkle; S.; Keereetaweep; J.; Shanklin; J.; & Schwender; J. (2022). An expanded role for the transcription factor WRINKLED1 in the biosynthesis of triacylglycerols during seed development. Frontiers in Plant Scienc | Sobic.006G07029 | yes | 55.33 | 56.99 | SP | 0.9967 SP 0.9990 | | | ortholo | no | nd |
| carbohydrate synthesi | INV | invertase | AT3G5260 Manuscript (Kuczynski; C.; McCorkle; S.; Keereetaweep; J.; Shanklin; J.; & Schwender; J. (2022). An expanded role for the transcription factor WRINKLED1 in the biosynthesis of triacylglycerols during seed development. Frontiers in Plant Scienc | Sobic.006G07083 | yes | 55.69 | 55.46 | SP | 0.9967 SP 0.9999 | | | ortholo | no | nd |
| carbohydrate synthesi | INV | invertase | AT3G5260 Manuscript (Kuczynski; C.; McCorkle; S.; Keereetaweep; J.; Shanklin; J.; & Schwender; J. (2022). An expanded role for the transcription factor WRINKLED1 in the biosynthesis of triacylglycerols during seed development. Frontiers in Plant Scienc | Sobic.001G09970 | yes | 56.36 | 56.54 | SP | 0.9967 SP 1.0000 | | | ortholo | no | nd |
| carbohydrate synthesi | INV | invertase | AT3G5260 Manuscript (Kuczynski; C.; McCorkle; S.; Keereetaweep; J.; Shanklin; J.; & Schwender; J. (2022). An expanded role for the transcription factor WRINKLED1 in the biosynthesis of triacylglycerols during seed development. Frontiers in Plant Scienc | Sobic.006G07003 | yes | 55.89 | 55.48 | SP | 0.9967 SP 0.9500 | | | LDO | no | nd |
| carbohydrate synthesi | SPP | sucrose-6-phosphate phosphohydrolas | AT3G5427 Manuscript (Kuczynski; C.; McCorkle; S.; Keereetaweep; J.; Shanklin; J.; & Schwender; J. (2022). An expanded role for the transcription factor WRINKLED1 in the biosynthesis of triacylglycerols during seed development. Frontiers in Plant Scienc | Sobic.004G15180 | no | 51.79 | 49.53 | OTHER | 0.9999 OTHER 0.9945 | | | ortholo | no | 4765.5 |
| carbohydrate synthesi | SPP | sucrose-6-phosphate phosphohydrolas | AT3G5427 Manuscript (Kuczynski; C.; McCorkle; S.; Keereetaweep; J.; Shanklin; J.; & Schwender; J. (2022). An expanded role for the transcription factor WRINKLED1 in the biosynthesis of triacylglycerols during seed development. Frontiers in Plant Scienc | Sobic.009G04090 | yes | 52.34 | 51.66 | OTHER | 0.9999 OTHER 0.9999 | | | ortholo | no | 73.2 |
| carbohydrate synthesi | SPP | sucrose-6-phosphate phosphohydrolas | AT3G5427 Manuscript (Kuczynski; C.; McCorkle; S.; Keereetaweep; J.; Shanklin; J.; & Schwender; J. (2022). An expanded role for the transcription factor WRINKLED1 in the biosynthesis of triacylglycerols during seed development. Frontiers in Plant Scienc | Sobic.009G04100 | no | 50.23 | 50 | OTHER | 0.9999 OTHER 1.0000 | | | ortholo | no | 3.5 |
| carbohydrate synthesi | SUS3 | sucrose synthase | AT4G0228 Manuscript (Kuczynski; C.; McCorkle; S.; Keereetaweep; J.; Shanklin; J.; & Schwender; J. (2022). An expanded role for the transcription factor WRINKLED1 in the biosynthesis of triacylglycerols during seed development. Frontiers in Plant Scienc | Sobic.001G37830 | bidirectiona | 79.83 | 79.93 | OTHER | 0.9939 OTHER 0.9998 | | | LDO | no | 748.1 |
| carbohydrate synthesi | SUS3 | sucrose synthase | AT4G0228 Manuscript (Kuczynski; C.; McCorkle; S.; Keereetaweep; J.; Shanklin; J.; & Schwender; J. (2022). An expanded role for the transcription factor WRINKLED1 in the biosynthesis of triacylglycerols during seed development. Frontiers in Plant Scienc | Sobic.001G34450 | no | 70.25 | 69.02 | OTHER | 0.9939 OTHER 0.9997 | | | no | no | 1317.2 |
| carbohydrate synthesi | SUS3 | sucrose synthase | AT4G0228 Manuscript (Kuczynski; C.; McCorkle; S.; Keereetaweep; J.; Shanklin; J.; & Schwender; J. (2022). An expanded role for the transcription factor WRINKLED1 in the biosynthesis of triacylglycerols during seed development. Frontiers in Plant Scienc | Sobic.010G07230 | no | 69.4 | 69.33 | OTHER | 0.9939 mTP 0.6921 | | | no | no | 2515.0 |
| carbohydrate synthesi | INV | invertase | AT4G0951 Manuscript (Kuczynski; C.; McCorkle; S.; Keereetaweep; J.; Shanklin; J.; & Schwender; J. (2022). An expanded role for the transcription factor WRINKLED1 in the biosynthesis of triacylglycerols during seed development. Frontiers in Plant Scienc | Sobic.004G02450 | yes | 64.55 | 64.17 | OTHER | 1.0000 OTHER 0.9998 | | | no | no | nd |
| carbohydrate synthesi | INV | invertase | AT4G0951 Manuscript (Kuczynski; C.; McCorkle; S.; Keereetaweep; J.; Shanklin; J.; & Schwender; J. (2022). An expanded role for the transcription factor WRINKLED1 in the biosynthesis of triacylglycerols during seed development. Frontiers in Plant Scienc | Sobic.005G05880 | yes | 74.23 | 73.99 | OTHER | 1.0000 OTHER 0.9983 | | | ortholo | no | 1004.3 |
| carbohydrate synthesi | INV | invertase | AT4G0951 Manuscript (Kuczynski; C.; McCorkle; S.; Keereetaweep; J.; Shanklin; J.; & Schwender; J. (2022). An expanded role for the transcription factor WRINKLED1 in the biosynthesis of triacylglycerols during seed development. Frontiers in Plant Scienc | Sobic.004G17270 | bidirectiona | 80.58 | 79.2 | OTHER | 1.0000 OTHER 0.9962 | | | ortholo | no | 798.0 |
| carbohydrate synthesi | INV | invertase | AT4G0951 Manuscript (Kuczynski; C.; McCorkle; S.; Keereetaweep; J.; Shanklin; J.; & Schwender; J. (2022). An expanded role for the transcription factor WRINKLED1 in the biosynthesis of triacylglycerols during seed development. Frontiers in Plant Scienc | Sobic.004G25560 | yes | 75.92 | 77.03 | OTHER | 1.0000 OTHER 0.9989 | | | ortholo | no | 3004.9 |
| carbohydrate synthesi | INV | invertase | AT4G0951 Manuscript (Kuczynski; C.; McCorkle; S.; Keereetaweep; J.; Shanklin; J.; & Schwender; J. (2022). An expanded role for the transcription factor WRINKLED1 in the biosynthesis of triacylglycerols during seed development. Frontiers in Plant Scienc | Sobic.004G16380 | no | 59.52 | 52.33 | OTHER | 1.0000 OTHER 0.6917 | | | no | no | 3099.2 |
| carbohydrate synthesi | INV | invertase | AT4G0951 Manuscript (Kuczynski; C.; McCorkle; S.; Keereetaweep; J.; Shanklin; J.; & Schwender; J. (2022). An expanded role for the transcription factor WRINKLED1 in the biosynthesis of triacylglycerols during seed development. Frontiers in Plant Scienc | Sobic.001G39160 | no | 58.58 | 51.76 | OTHER | 1.0000 mTP 0.9153 | | | no | no | 637.3 |
| carbohydrate synthesi | INV | invertase | AT4G0951 Manuscript (Kuczynski; C.; McCorkle; S.; Keereetaweep; J.; Shanklin; J.; & Schwender; J. (2022). An expanded role for the transcription factor WRINKLED1 in the biosynthesis of triacylglycerols during seed development. Frontiers in Plant Scienc | Sobic.003G15380 | no | 59.43 | 52.31 | OTHER | 1.0000 cTP 0.5389 | | | no | no | 1159.7 |
| carbohydrate synthesi | SPS | sucrose phosphate synthase | AT4G1012 Manuscript (Kuczynski; C.; McCorkle; S.; Keereetaweep; J.; Shanklin; J.; & Schwender; J. (2022). An expanded role for the transcription factor WRINKLED1 in the biosynthesis of triacylglycerols during seed development. Frontiers in Plant Scienc | Sobic.004G06840 | no | 47.96 | 52.19 | OTHER | 1.0000 OTHER 1.0000 | | | no | no | 2249.5 |
| carbohydrate synthesi | SPS | sucrose phosphate synthase | AT4G1012 Manuscript (Kuczynski; C.; McCorkle; S.; Keereetaweep; J.; Shanklin; J.; & Schwender; J. (2022). An expanded role for the transcription factor WRINKLED1 in the biosynthesis of triacylglycerols during seed development. Frontiers in Plant Scienc | Sobic.010G20510 | no | 46.12 | 51 | OTHER | 1.0000 OTHER 1.0000 | | | no | no | 1205.4 |
| carbohydrate synthesi | SPS | sucrose phosphate synthase | AT4G1012 Manuscript (Kuczynski; C.; McCorkle; S.; Keereetaweep; J.; Shanklin; J.; & Schwender; J. (2022). An expanded role for the transcription factor WRINKLED1 in the biosynthesis of triacylglycerols during seed development. Frontiers in Plant Scienc | Sobic.009G23320 | no | 51.94 | 53.62 | OTHER | 1.0000 OTHER 1.0000 | | | no | no | 2229.7 |
| carbohydrate synthesi | SPS | sucrose phosphate synthase | AT4G1012 Manuscript (Kuczynski; C.; McCorkle; S.; Keereetaweep; J.; Shanklin; J.; & Schwender; J. (2022). An expanded role for the transcription factor WRINKLED1 in the biosynthesis of triacylglycerols during seed development. Frontiers in Plant Scienc | Sobic.003G40330 | yes | 55.24 | 56.2 | OTHER | 1.0000 OTHER 1.0000 | | | no | no 15729.0 | |
| carbohydrate synthesi | SPS | sucrose phosphate synthase | AT4G1012 Manuscript (Kuczynski; C.; McCorkle; S.; Keereetaweep; J.; Shanklin; J.; & Schwender; J. (2022). An expanded role for the transcription factor WRINKLED1 in the biosynthesis of triacylglycerols during seed development. Frontiers in Plant Scienc | Sobic.005G08960 | yes | 55.17 | 57.23 | OTHER | 1.0000 OTHER 0.9995 | | | LDO | no 31292.3 | |
| carbohydrate synthesi | HXK | hexokinase | AT4G2913 Manuscript (Kuczynski; C.; McCorkle; S.; Keereetaweep; J.; Shanklin; J.; & Schwender; J. (2022). An expanded role for the transcription factor WRINKLED1 in the biosynthesis of triacylglycerols during seed development. Frontiers in Plant Scienc | Sobic.003G42120 | no | 52.46 | 54.64 | OTHER | 0.6880 OTHER 0.3410 | | | no | no 649.4 | |
| carbohydrate synthesi | HXK | hexokinase | AT4G2913 Manuscript (Kuczynski; C.; McCorkle; S.; Keereetaweep; J.; Shanklin; J.; & Schwender; J. (2022). An expanded role for the transcription factor WRINKLED1 in the biosynthesis of triacylglycerols during seed development. Frontiers in Plant Scienc | Sobic.009G11910 | no | 49.08 | 48.77 | OTHER | 0.6880 SP 0.5806 | | | no | no nd | |
| carbohydrate synthesi | HXK | hexokinase | AT4G2913 Manuscript (Kuczynski; C.; McCorkle; S.; Keereetaweep; J.; Shanklin; J.; & Schwender; J. (2022). An expanded role for the transcription factor WRINKLED1 in the biosynthesis of triacylglycerols during seed development. Frontiers in Plant Scienc | Sobic.003G03550 | yes | 62.39 | 61.56 | OTHER | 0.6880 OTHER 0.9967 | | | ortholo | no | 59.7 |
| carbohydrate synthesi | HXK | hexokinase | AT4G2913 Manuscript (Kuczynski; C.; McCorkle; S.; Keereetaweep; J.; Shanklin; J.; & Schwender; J. (2022). An expanded role for the transcription factor WRINKLED1 in the biosynthesis of triacylglycerols during seed development. Frontiers in Plant Scienc | Sobic.009G06980 | yes | 62.28 | 60.7 | OTHER | 0.6880 OTHER 0.9985 | | | ortholo | no | 478.2 |
| carbohydrate synthesi | HXK | hexokinase | AT4G2913 Manuscript (Kuczynski; C.; McCorkle; S.; Keereetaweep; J.; Shanklin; J.; & Schwender; J. (2022). An expanded role for the transcription factor WRINKLED1 in the biosynthesis of triacylglycerols during seed development. Frontiers in Plant Scienc | Sobic.003G28040 | yes | 65.33 | 64.34 | OTHER | 0.6880 SP 0.4947 | | | ortholo | no | 40.1 |
| carbohydrate synthesi | HXK | hexokinase | AT4G2913 Manuscript (Kuczynski; C.; McCorkle; S.; Keereetaweep; J.; Shanklin; J.; & Schwender; J. (2022). An expanded role for the transcription factor WRINKLED1 in the biosynthesis of triacylglycerols during seed development. Frontiers in Plant Scienc | Sobic.003G29180 | yes | 67.3 | 66.46 | OTHER | 0.6880 OTHER 0.8975 | | | ortholo | no | 1074.2 |
| carbohydrate synthesi | HXK | hexokinase | AT4G2913 Manuscript (Kuczynski; C.; McCorkle; S.; Keereetaweep; J.; Shanklin; J.; & Schwender; J. (2022). An expanded role for the transcription factor WRINKLED1 in the biosynthesis of triacylglycerols during seed development. Frontiers in Plant Scienc | Sobic.009G20350 | bidirectiona | 68.55 | 66.87 | OTHER | 0.6880 OTHER 0.5645 | | | ortholo | no | 405.5 |
| carbohydrate synthesi | INV | invertase | AT4G3486 Manuscript (Kuczynski; C.; McCorkle; S.; Keereetaweep; J.; Shanklin; J.; & Schwender; J. (2022). An expanded role for the transcription factor WRINKLED1 in the biosynthesis of triacylglycerols during seed development. Frontiers in Plant Scienc | Sobic.004G02450 | no | 69.45 | 64.3 | OTHER | 0.9999 OTHER 0.9998 | | | ortholo | no | nd |
| carbohydrate synthesi | INV | invertase | AT4G3486 Manuscript (Kuczynski; C.; McCorkle; S.; Keereetaweep; J.; Shanklin; J.; & Schwender; J. (2022). An expanded role for the transcription factor WRINKLED1 in the biosynthesis of triacylglycerols during seed development. Frontiers in Plant Scienc | Sobic.005G05880 | no | 72.68 | 73.04 | OTHER | 0.9999 OTHER 0.9983 | | | no | no | 1004.3 |
| carbohydrate synthesi | INV | invertase | AT4G3486 Manuscript (Kuczynski; C.; McCorkle; S.; Keereetaweep; J.; Shanklin; J.; & Schwender; J. (2022). An expanded role for the transcription factor WRINKLED1 in the biosynthesis of triacylglycerols during seed development. Frontiers in Plant Scienc | Sobic.004G17270 | yes | 76.19 | 76.94 | OTHER | 0.9999 OTHER 0.9962 | | | no | no | 798.0 |
| carbohydrate synthesi | INV | invertase | AT4G3486 Manuscript (Kuczynski; C.; McCorkle; S.; Keereetaweep; J.; Shanklin; J.; & Schwender; J. (2022). An expanded role for the transcription factor WRINKLED1 in the biosynthesis of triacylglycerols during seed development. Frontiers in Plant Scienc | Sobic.004G25560 | no | 72.46 | 72.73 | OTHER | 0.9999 OTHER 0.9989 | | | no | no | 3004.9 |
| carbohydrate synthesi | INV | invertase | AT4G3486 Manuscript (Kuczynski; C.; McCorkle; S.; Keereetaweep; J.; Shanklin; J.; & Schwender; J. (2022). An expanded role for the transcription factor WRINKLED1 in the biosynthesis of triacylglycerols during seed development. Frontiers in Plant Scienc | Sobic.004G16380 | no | 52.71 | 50.36 | OTHER | 0.9999 OTHER 0.6917 | | | no | no | 3099.2 |
| carbohydrate synthesi | INV | invertase | AT4G3486 Manuscript (Kuczynski; C.; McCorkle; S.; Keereetaweep; J.; Shanklin; J.; & Schwender; J. (2022). An expanded role for the transcription factor WRINKLED1 in the biosynthesis of triacylglycerols during seed development. Frontiers in Plant Scienc | Sobic.001G39160 | no | 58.25 | 51.64 | OTHER | 0.9999 mTP 0.9153 | | | no | no | 637.3 |
| carbohydrate synthesi | INV | invertase | AT4G3486 Manuscript (Kuczynski; C.; McCorkle; S.; Keereetaweep; J.; Shanklin; J.; & Schwender; J. (2022). An expanded role for the transcription factor WRINKLED1 in the biosynthesis of triacylglycerols during seed development. Frontiers in Plant Scienc | Sobic.003G15380 | no | 60.09 | 51.81 | OTHER | 0.9999 cTP 0.5389 | | | no | no | 1159.7 |
| carbohydrate synthesi | HXK | hexokinase | AT4G3784 Manuscript (Kuczynski; C.; McCorkle; S.; Keereetaweep; J.; Shanklin; J.; & Schwender; J. (2022). An expanded role for the transcription factor WRINKLED1 in the biosynthesis of triacylglycerols during seed development. Frontiers in Plant Scienc | Sobic.003G42120 | no | 40.85 | 40.42 | OTHER | 0.8118 OTHER 0.3410 | | | no | no | 649.4 |
| carbohydrate synthesi | HXK | hexokinase | AT4G3784 Manuscript (Kuczynski; C.; McCorkle; S.; Keereetaweep; J.; Shanklin; J.; & Schwender; J. (2022). An expanded role for the transcription factor WRINKLED1 in the biosynthesis of triacylglycerols during seed development. Frontiers in Plant Scienc | Sobic.009G11910 | no | 38.49 | 37.55 | OTHER | 0.8118 SP 0.5806 | | | no | no | nd |
| carbohydrate synthesi | HXK | hexokinase | AT4G3784 Manuscript (Kuczynski; C.; McCorkle; S.; Keereetaweep; J.; Shanklin; J.; & Schwender; J. (2022). An expanded role for the transcription factor WRINKLED1 in the biosynthesis of triacylglycerols during seed development. Frontiers in Plant Scienc | Sobic.003G03550 | no | 40.96 | 40 | OTHER | 0.8118 OTHER 0.9967 | | | ortholo | no | 59.7 |
| carbohydrate synthesi | HXK | hexokinase | AT4G3784 Manuscript (Kuczynski; C.; McCorkle; S.; Keereetaweep; J.; Shanklin; J.; & Schwender; J. (2022). An expanded role for the transcription factor WRINKLED1 in the biosynthesis of triacylglycerols during seed development. Frontiers in Plant Scienc | Sobic.009G06980 | no | 40.05 | 39.6 | OTHER | 0.8118 OTHER 0.9985 | | | ortholo | no | 478.2 |
| carbohydrate synthesi | HXK | hexokinase | AT4G3784 Manuscript (Kuczynski; C.; McCorkle; S.; Keereetaweep; J.; Shanklin; J.; & Schwender; J. (2022). An expanded role for the transcription factor WRINKLED1 in the biosynthesis of triacylglycerols during seed development. Frontiers in Plant Scienc | Sobic.003G28040 | yes | 44.13 | 42.98 | OTHER | 0.8118 SP 0.4947 | | | ortholo | no | 40.1 |
| carbohydrate synthesi | HXK | hexokinase | AT4G3784 Manuscript (Kuczynski; C.; McCorkle; S.; Keereetaweep; J.; Shanklin; J.; & Schwender; J. (2022). An expanded role for the transcription factor WRINKLED1 in the biosynthesis of triacylglycerols during seed development. Frontiers in Plant Scienc | Sobic.003G29180 | no | 41.1 | 40.37 | OTHER | 0.8118 OTHER 0.8975 | | | ortholo | no | 1074.2 |
| carbohydrate synthesi | HXK | hexokinase | AT4G3784 Manuscript (Kuczynski; C.; McCorkle; S.; Keereetaweep; J.; Shanklin; J.; & Schwender; J. (2022). An expanded role for the transcription factor WRINKLED1 in the biosynthesis of triacylglycerols during seed development. Frontiers in Plant Scienc | Sobic.009G20350 | no | 40.64 | 39.67 | OTHER | 0.8118 OTHER 0.5645 | | | ortholo | no | 405.5 |
| carbohydrate synthesi | SPS | sucrose phosphate synthase | AT5G1111 Manuscript (Kuczynski; C.; McCorkle; S.; Keereetaweep; J.; Shanklin; J.; & Schwender; J. (2022). An expanded role for the transcription factor WRINKLED1 in the biosynthesis of triacylglycerols during seed development. Frontiers in Plant Scienc | Sobic.004G06840 | no | 64.46 | 61.37 | OTHER | 1.0000 OTHER 1.0000 | | | ortholo | no | 2249.5 |
| carbohydrate synthesi | SPS | sucrose phosphate synthase | AT5G1111 Manuscript (Kuczynski; C.; McCorkle; S.; Keereetaweep; J.; Shanklin; J.; & Schwender; J. (2022). An expanded role for the transcription factor WRINKLED1 in the biosynthesis of triacylglycerols during seed development. Frontiers in Plant Scienc | Sobic.010G20510 | no | 63.73 | 60.04 | OTHER | 1.0000 OTHER 1.0000 | | | ortholo | no | 1205.4 |
| carbohydrate synthesi | SPS | sucrose phosphate synthase | AT5G1111 Manuscript (Kuczynski; C.; McCorkle; S.; Keereetaweep; J.; Shanklin; J.; & Schwender; J. (2022). An expanded role for the transcription factor WRINKLED1 in the biosynthesis of triacylglycerols during seed development. Frontiers in Plant Scienc | Sobic.009G23320 | yes | 65.26 | 66.54 | OTHER | 1.0000 OTHER 1.0000 | | | ortholo | no | 2229.7 |
| carbohydrate synthesi | UGP | UDP-glucose pyrophosphorylas | AT5G1731 Manuscript (Kuczynski; C.; McCorkle; S.; Keereetaweep; J.; Shanklin; J.; & Schwender; J. (2022). An expanded role for the transcription factor WRINKLED1 in the biosynthesis of triacylglycerols during seed development. Frontiers in Plant Scienc | Sobic.002G29120 | yes | 83.19 | 82.98 | cTP | 0.0042 OTHER 0.9994 | | | ortholo | no | 54425.2 |
| carbohydrate synthesi | UGP | UDP-glucose pyrophosphorylas | AT5G1731 Manuscript (Kuczynski; C.; McCorkle; S.; Keereetaweep; J.; Shanklin; J.; & Schwender; J. (2022). An expanded role for the transcription factor WRINKLED1 in the biosynthesis of triacylglycerols during seed development. Frontiers in Plant Scienc | Sobic.004G01350 | bidirectiona | 83.73 | 83.51 | cTP | 0.0042 OTHER 0.9998 | | | ortholo | no | nd |
| carbohydrate synthesi | SPS | sucrose phosphate synthase | AT5G2028 Manuscript (Kuczynski; C.; McCorkle; S.; Keereetaweep; J.; Shanklin; J.; & Schwender; J. (2022). An expanded role for the transcription factor WRINKLED1 in the biosynthesis of triacylglycerols during seed development. Frontiers in Plant Scienc | Sobic.004G06840 | yes | 55.85 | 61.43 | OTHER | 1.0000 OTHER 1.0000 | | | ortholo | no | 2249.5 |
| carbohydrate synthesi | SPS | sucrose phosphate synthase | AT5G2028 Manuscript (Kuczynski; C.; McCorkle; S.; Keereetaweep; J.; Shanklin; J.; & Schwender; J. (2022). An expanded role for the transcription factor WRINKLED1 in the biosynthesis of triacylglycerols during seed development. Frontiers in Plant Scienc | Sobic.010G20510 | yes | 64.13 | 61.09 | OTHER | 1.0000 OTHER 1.0000 | | | ortholo | no | 1205.4 |
| carbohydrate synthesi | SPS | sucrose phosphate synthase | AT5G2028 Manuscript (Kuczynski; C.; McCorkle; S.; Keereetaweep; J.; Shanklin; J.; & Schwender; J. (2022). An expanded role for the transcription factor WRINKLED1 in the biosynthesis of triacylglycerols during seed development. Frontiers in Plant Scienc | Sobic.009G23320 | bidirectiona | 65.64 | 67.32 | OTHER | 1.0000 OTHER 1.0000 | | | LDO | no | 2229.7 |
| carbohydrate synthesi | SUS1 | sucrose synthase | AT5G2083 Manuscript (Kuczynski; C.; McCorkle; S.; Keereetaweep; J.; Shanklin; J.; & Schwender; J. (2022). An expanded role for the transcription factor WRINKLED1 in the biosynthesis of triacylglycerols during seed development. Frontiers in Plant Scienc | Sobic.001G37830 | no | 67.67 | 67.41 | OTHER | 0.9996 OTHER 0.9998 | | | no | no | 748.1 |
| carbohydrate synthesi | SUS1 | sucrose synthase | AT5G2083 Manuscript (Kuczynski; C.; McCorkle; S.; Keereetaweep; J.; Shanklin; J.; & Schwender; J. (2022). An expanded role for the transcription factor WRINKLED1 in the biosynthesis of triacylglycerols during seed development. Frontiers in Plant Scienc | Sobic.001G34450 | yes | 72.3 | 71.91 | OTHER | 0.9996 OTHER 0.9997 | | | LDO | no | 1317.2 |
| carbohydrate synthesi | SUS1 | sucrose synthase | AT5G2083 Manuscript (Kuczynski; C.; McCorkle; S.; Keereetaweep; J.; Shanklin; J.; & Schwender; J. (2022). An expanded role for the transcription factor WRINKLED1 in the biosynthesis of triacylglycerols during seed development. Frontiers in Plant Scienc | Sobic.010G07230 | no | 73.75 | 73.66 | OTHER | 0.9996 mTP 0.6921 | | | ortholo | no | 2515.0 |
| carbohydrate synthesi | SUS5 | sucrose synthase | AT5G3718 Manuscript (Kuczynski; C.; McCorkle; S.; Keereetaweep; J.; Shanklin; J.; & Schwender; J. (2022). An expanded role for the transcription factor WRINKLED1 in the biosynthesis of triacylglycerols during seed development. Frontiers in Plant Scienc | Sobic.004G35760 | no | 58.71 | 60.55 | OTHER | 0.9919 OTHER 0.9928 | | | ortholo | no | 13.6 |
| carbohydrate synthesi | SUS5 | sucrose synthase | AT5G3718 Manuscript (Kuczynski; C.; McCorkle; S.; Keereetaweep; J.; Shanklin; J.; & Schwender; J. (2022). An expanded role for the transcription factor WRINKLED1 in the biosynthesis of triacylglycerols during seed development. Frontiers in Plant Scienc | Sobic.010G27670 | bidirectiona | 66.23 | 65.58 | OTHER | 0.9919 OTHER 0.8679 | | | LDO | no | 7.2 |
| carbohydrate synthesi | SUS2 | sucrose synthase | AT5G4919 Manuscript (Kuczynski; C.; McCorkle; S.; Keereetaweep; J.; Shanklin; J.; & Schwender; J. (2022). An expanded role for the transcription factor WRINKLED1 in the biosynthesis of triacylglycerols during seed development. Frontiers in Plant Scienc | Sobic.001G37830 | yes | 74.19 | 73.54 | OTHER | 0.9996 OTHER 0.9998 | | | ortholo | yes | 748.1 |
| carbohydrate synthesi | SUS2 | sucrose synthase | AT5G4919 Manuscript (Kuczynski; C.; McCorkle; S.; Keereetaweep; J.; Shanklin; J.; & Schwender; J. (2022). An expanded role for the transcription factor WRINKLED1 in the biosynthesis of triacylglycerols during seed development. Frontiers in Plant Scienc | Sobic.001G34450 | no | 68.66 | 68.32 | OTHER | 0.9996 OTHER 0.9997 | | | no | yes | 1317.2 |
| carbohydrate synthesi | SUS2 | sucrose synthase | AT5G4919 Manuscript (Kuczynski; C.; McCorkle; S.; Keereetaweep; J.; Shanklin; J.; & Schwender; J. (2022). An expanded role for the transcription factor WRINKLED1 in the biosynthesis of triacylglycerols during seed development. Frontiers in Plant Scienc | Sobic.010G07230 | no | 68.91 | 68.88 | OTHER | 0.9996 | mTP | 0.6921 | no | yes | 2515.0 |
| de novo FA synthesi | FAT-A/B | Acyl-ACP Thioesterase | AT1G0851 Manuscript (Kuczynski; C.; McCorkle; S.; Keereetaweep; J.; Shanklin; J.; & Schwender; J. (2022). An expanded role for the transcription factor WRINKLED1 in the biosynthesis of triacylglycerols during seed development. Frontiers in Plant Scienc | Sobic.010G03330 | bidirectiona | 61.76 | 61.33 | cTP | 0.8572 | cTP | 0.9055 | LDO | no | 494.6 |
| de novo FA synthesi | FAT-A/B | Acyl-ACP Thioesterase | AT1G0851 Manuscript (Kuczynski; C.; McCorkle; S.; Keereetaweep; J.; Shanklin; J.; & Schwender; J. (2022). An expanded role for the transcription factor WRINKLED1 in the biosynthesis of triacylglycerols during seed development. Frontiers in Plant Scienc | Sobic.004G08810 | no | 57.42 | 61.33 | cTP | 0.8572 | cTP | 0.9649 | ortholo | no | 1376.0 |
| de novo FA synthesi | FAT-A/B | Acyl-ACP Thioesterase | AT1G0851 Manuscript (Kuczynski; C.; McCorkle; S.; Keereetaweep; J.; Shanklin; J.; & Schwender; J. (2022). An expanded role for the transcription factor WRINKLED1 in the biosynthesis of triacylglycerols during seed development. Frontiers in Plant Scienc | Sobic.010G18040 | no | 59.18 | 57 | cTP | 0.8572 | cTP | 0.9483 | ortholo | no | 1481.3 |
| de novo FA synthesi | KAR | Ketoacyl-ACP Reductase | AT1G2436 Manuscript (Kuczynski; C.; McCorkle; S.; Keereetaweep; J.; Shanklin; J.; & Schwender; J. (2022). An expanded role for the transcription factor WRINKLED1 in the biosynthesis of triacylglycerols during seed development. Frontiers in Plant Scienc | Sobic.008G08730 | yes | 66.94 | 58.5 | cTP | 0.8658 | cTP | 0.9304 | ortholo | no | nd |
| de novo FA synthesi | KAR | Ketoacyl-ACP Reductase | AT1G2436 Manuscript (Kuczynski; C.; McCorkle; S.; Keereetaweep; J.; Shanklin; J.; & Schwender; J. (2022). An expanded role for the transcription factor WRINKLED1 in the biosynthesis of triacylglycerols during seed development. Frontiers in Plant Scienc | Sobic.004G15280 | bidirectiona | 76.52 | 69.87 | cTP | 0.8658 | cTP | 0.9984 | ortholo | no | 234.8 |
| de novo FA synthesi | KAR | Ketoacyl-ACP Reductase | AT1G2436 Manuscript (Kuczynski; C.; McCorkle; S.; Keereetaweep; J.; Shanklin; J.; & Schwender; J. (2022). An expanded role for the transcription factor WRINKLED1 in the biosynthesis of triacylglycerols during seed development. Frontiers in Plant Scienc | Sobic.006G04370 | yes | 75.47 | 70.2 | cTP | 0.8658 | cTP | 0.9999 | LDO | no | 13217.4 |
| de novo FA synthesi | FAB2 / SAD1-6 | Stearoyl-ACP Desaturase | AT1G4380 Manuscript (Kuczynski; C.; McCorkle; S.; Keereetaweep; J.; Shanklin; J.; & Schwender; J. (2022). An expanded role for the transcription factor WRINKLED1 in the biosynthesis of triacylglycerols during seed development. Frontiers in Plant Scienc | Sobic.003G37710 | bidirectiona | 75.37 | 70.45 | cTP | 0.9815 | cTP | 0.9065 | LDO | no | nd |
| de novo FA synthesi | FAB2 / SAD1-6 | Stearoyl-ACP Desaturase | AT1G4380 Manuscript (Kuczynski; C.; McCorkle; S.; Keereetaweep; J.; Shanklin; J.; & Schwender; J. (2022). An expanded role for the transcription factor WRINKLED1 in the biosynthesis of triacylglycerols during seed development. Frontiers in Plant Scienc | Sobic.006G04870 | no | 68.33 | 66.04 | cTP | 0.9815 | cTP | 0.9972 | no | no | 5481.2 |
| de novo FA synthesi | FAB2 / SAD1-6 | Stearoyl-ACP Desaturase | AT1G4380 Manuscript (Kuczynski; C.; McCorkle; S.; Keereetaweep; J.; Shanklin; J.; & Schwender; J. (2022). An expanded role for the transcription factor WRINKLED1 in the biosynthesis of triacylglycerols during seed development. Frontiers in Plant Scienc | Sobic.003G40400 | no | 68.57 | 64.71 | cTP | 0.9815 | cTP | 0.8477 | no | no | 19290.1 |
| de novo FA synthesi | FAB2 / SAD1-6 | Stearoyl-ACP Desaturase | AT1G4380 Manuscript (Kuczynski; C.; McCorkle; S.; Keereetaweep; J.; Shanklin; J.; & Schwender; J. (2022). An expanded role for the transcription factor WRINKLED1 in the biosynthesis of triacylglycerols during seed development. Frontiers in Plant Scienc | Sobic.004G15330 | no | 64.21 | 60 | cTP | 0.9815 | cTP | 0.9992 | no | no | 14.8 |
| de novo FA synthesi | FAB2 / SAD1-6 | Stearoyl-ACP Desaturase | AT1G4380 Manuscript (Kuczynski; C.; McCorkle; S.; Keereetaweep; J.; Shanklin; J.; & Schwender; J. (2022). An expanded role for the transcription factor WRINKLED1 in the biosynthesis of triacylglycerols during seed development. Frontiers in Plant Scienc | Sobic.007G07160 | no | 56.76 | 51.47 | cTP | 0.9815 | cTP | 0.9867 | no | no | nd |
| de novo FA synthesi | FAB2 / SAD1-6 | Stearoyl-ACP Desaturase | AT1G4380 Manuscript (Kuczynski; C.; McCorkle; S.; Keereetaweep; J.; Shanklin; J.; & Schwender; J. (2022). An expanded role for the transcription factor WRINKLED1 in the biosynthesis of triacylglycerols during seed development. Frontiers in Plant Scienc | Sobic.001G28070 | no | 54.44 | 49.47 | cTP | 0.9815 | cTP | 0.9972 | no | no | 1806.0 |
| de novo FA synthesi | FAB2 / SAD1-6 | Stearoyl-ACP Desaturase | AT1G4380 Manuscript (Kuczynski; C.; McCorkle; S.; Keereetaweep; J.; Shanklin; J.; & Schwender; J. (2022). An expanded role for the transcription factor WRINKLED1 in the biosynthesis of triacylglycerols during seed development. Frontiers in Plant Scienc | Sobic.010G23530 | no | 53.82 | 49.18 | cTP | 0.9815 | cTP | 0.8511 | no | no | nd |
| de novo FA synthesi | FAB2 / SAD1-6 | Stearoyl-ACP Desaturase | AT1G4380 Manuscript (Kuczynski; C.; McCorkle; S.; Keereetaweep; J.; Shanklin; J.; & Schwender; J. (2022). An expanded role for the transcription factor WRINKLED1 in the biosynthesis of triacylglycerols during seed development. Frontiers in Plant Scienc | Sobic.002G25850 | no | 55.21 | 48.39 | cTP | 0.9815 | cTP | 0.7060 | no | no | 22.1 |
| de novo FA synthesi | FAB2 / SAD1-6 | Stearoyl-ACP Desaturase | AT1G4380 Manuscript (Kuczynski; C.; McCorkle; S.; Keereetaweep; J.; Shanklin; J.; & Schwender; J. (2022). An expanded role for the transcription factor WRINKLED1 in the biosynthesis of triacylglycerols during seed development. Frontiers in Plant Scienc | Sobic.001G33450 | no | 50.71 | 47.88 | cTP | 0.9815 | cTP | 0.8479 | no | no | nd |
| de novo FA synthesi | FAB2 / SAD1-6 | Stearoyl-ACP Desaturase | AT1G4380 Manuscript (Kuczynski; C.; McCorkle; S.; Keereetaweep; J.; Shanklin; J.; & Schwender; J. (2022). An expanded role for the transcription factor WRINKLED1 in the biosynthesis of triacylglycerols during seed development. Frontiers in Plant Scienc | Sobic.001G26530 | no | 50.87 | 45.85 | cTP | 0.9815 | cTP | 0.7420 | no | no | nd |
| de novo FA synthesi FAB2 / SAD1-6 Stearoyl-ACP Desaturase AT1G4380 Manuscript (Kuczynski; C.; McCorkle; S.; Keereetaweep; J.; Shanklin; J.; & Schwender; J. (2022). An expanded role for the transcription factor WRINKLED1 in the biosynthesis of triacylglycerols during seed development. Frontiers in Plant Scienc Sobic.004G15755 no 54.29 31.32 cTP 0.9815 OTHER 0.8453 no no nd | | | | | | | | | | | | | | |
| de novo FA synthesi | ACP | Acyl carrier protein | AT1G5458 Manuscript (Kuczynski; C.; McCorkle; S.; Keereetaweep; J.; Shanklin; J.; & Schwender; J. (2022). An expanded role for the transcription factor WRINKLED1 in the biosynthesis of triacylglycerols during seed development. Frontiers in Plant Scienc | Sobic.002G28040 | bidirectiona | 53.79 | 50.78 | cTP | 0.9995 cTP 0.9986 | | | ortholo | no | 14213.7 |
| de novo FA synthesi | ACP | Acyl carrier protein | AT1G5458 Manuscript (Kuczynski; C.; McCorkle; S.; Keereetaweep; J.; Shanklin; J.; & Schwender; J. (2022). An expanded role for the transcription factor WRINKLED1 in the biosynthesis of triacylglycerols during seed development. Frontiers in Plant Scienc | Sobic.001G00570 | no | 56.07 | 48.89 | cTP | 0.9995 cTP 0.9996 | | | ortholo | no | 186.6 |
| de novo FA synthesi | ACP | Acyl carrier protein | AT1G5458 Manuscript (Kuczynski; C.; McCorkle; S.; Keereetaweep; J.; Shanklin; J.; & Schwender; J. (2022). An expanded role for the transcription factor WRINKLED1 in the biosynthesis of triacylglycerols during seed development. Frontiers in Plant Scienc | Sobic.007G17680 | no | 51.85 | 51.18 | cTP | 0.9995 cTP 0.9998 | | | ortholo | no | 23607.5 |
| de novo FA synthesi | ACP | Acyl carrier protein | AT1G5458 Manuscript (Kuczynski; C.; McCorkle; S.; Keereetaweep; J.; Shanklin; J.; & Schwender; J. (2022). An expanded role for the transcription factor WRINKLED1 in the biosynthesis of triacylglycerols during seed development. Frontiers in Plant Scienc | Sobic.008G11640 | no | 55.45 | 46.62 | cTP | 0.9995 cTP 0.9995 | | | ortholo | no | 635.2 |
| de novo FA synthesi | ACP | Acyl carrier protein | AT1G5463 Manuscript (Kuczynski; C.; McCorkle; S.; Keereetaweep; J.; Shanklin; J.; & Schwender; J. (2022). An expanded role for the transcription factor WRINKLED1 in the biosynthesis of triacylglycerols during seed development. Frontiers in Plant Scienc | Sobic.007G17680 | yes | 50.37 | 51.18 | cTP | 0.9990 cTP 0.9998 | | | ortholo | no | 23607.5 |
| de novo FA synthesi | ACP | Acyl carrier protein | AT1G5463 Manuscript (Kuczynski; C.; McCorkle; S.; Keereetaweep; J.; Shanklin; J.; & Schwender; J. (2022). An expanded role for the transcription factor WRINKLED1 in the biosynthesis of triacylglycerols during seed development. Frontiers in Plant Scienc | Sobic.001G00570 | no | 54.55 | 46.67 | cTP | 0.9990 cTP 0.9996 | | | ortholo | no | 186.6 |
| de novo FA synthesi | ACP | Acyl carrier protein | AT1G5463 Manuscript (Kuczynski; C.; McCorkle; S.; Keereetaweep; J.; Shanklin; J.; & Schwender; J. (2022). An expanded role for the transcription factor WRINKLED1 in the biosynthesis of triacylglycerols during seed development. Frontiers in Plant Scienc | Sobic.002G28040 | yes | 51.88 | 50.78 | cTP | 0.9990 cTP 0.9986 | | | ortholo | no | 14213.7 |
| de novo FA synthesi | ACP | Acyl carrier protein | AT1G5463 Manuscript (Kuczynski; C.; McCorkle; S.; Keereetaweep; J.; Shanklin; J.; & Schwender; J. (2022). An expanded role for the transcription factor WRINKLED1 in the biosynthesis of triacylglycerols during seed development. Frontiers in Plant Scienc | Sobic.008G11640 | yes | 56.12 | 45.86 | cTP | 0.9990 cTP 0.9995 | | | ortholo | no | 635.2 |
| de novo FA synthesi | KAR | Ketoacyl-ACP Reductase | AT1G6261 Manuscript (Kuczynski; C.; McCorkle; S.; Keereetaweep; J.; Shanklin; J.; & Schwender; J. (2022). An expanded role for the transcription factor WRINKLED1 in the biosynthesis of triacylglycerols during seed development. Frontiers in Plant Scienc | Sobic.007G20980 | yes | 61.76 | 60.29 | OTHER | 0.9999 OTHER 0.9648 | | | ortholo | no | 1109.8 |
| de novo FA synthesi | KAS III | Ketoacyl-ACP Synthase III | AT1G6264 Manuscript (Kuczynski; C.; McCorkle; S.; Keereetaweep; J.; Shanklin; J.; & Schwender; J. (2022). An expanded role for the transcription factor WRINKLED1 in the biosynthesis of triacylglycerols during seed development. Frontiers in Plant Scienc | Sobic.006G23700 | bidirectiona | 66.3 | 63.45 | cTP | 0.6242 | cTP | 0.9540 | LDO | yes | 2436.4 |
| de novo FA synthesi | KAS II | Ketoacyl-ACP Synthase II | AT1G7496 Manuscript (Kuczynski; C.; McCorkle; S.; Keereetaweep; J.; Shanklin; J.; & Schwender; J. (2022). An expanded role for the transcription factor WRINKLED1 in the biosynthesis of triacylglycerols during seed development. Frontiers in Plant Scienc | Sobic.001G34440 | bidirectiona | 67.47 | 74.13 | OTHER | 0.3533 | cTP | 0.5286 | LDO | no | 6033.6 |
| de novo FA synthesi | KAS II | Ketoacyl-ACP Synthase II | AT1G7496 Manuscript (Kuczynski; C.; McCorkle; S.; Keereetaweep; J.; Shanklin; J.; & Schwender; J. (2022). An expanded role for the transcription factor WRINKLED1 in the biosynthesis of triacylglycerols during seed development. Frontiers in Plant Scienc | Sobic.002G37460 | yes | 79.68 | 73.74 | OTHER | 0.3533 | SP | 0.7689 | ortholo | no | 16087.0 |
| de novo FA synthesi | ENR | Enoyl-ACP Reductase | AT2G0599 Manuscript (Kuczynski; C.; McCorkle; S.; Keereetaweep; J.; Shanklin; J.; & Schwender; J. (2022). An expanded role for the transcription factor WRINKLED1 in the biosynthesis of triacylglycerols during seed development. Frontiers in Plant Scienc | Sobic.002G15100 | bidirectiona | 72.75 | 74.19 | cTP | 0.9519 | cTP | 0.9751 | ortholo | no | 523.1 |
| de novo FA synthesi | ENR | Enoyl-ACP Reductase | AT2G0599 Manuscript (Kuczynski; C.; McCorkle; S.; Keereetaweep; J.; Shanklin; J.; & Schwender; J. (2022). An expanded role for the transcription factor WRINKLED1 in the biosynthesis of triacylglycerols during seed development. Frontiers in Plant Scienc | Sobic.007G11210 | yes | 72.28 | 73.44 | cTP | 0.9519 | cTP | 0.9967 | LDO | no | 4304.5 |
| de novo FA synthesi | HAD | Hydroxyacyl-ACP Dehydratase | AT2G2223 Manuscript (Kuczynski; C.; McCorkle; S.; Keereetaweep; J.; Shanklin; J.; & Schwender; J. (2022). An expanded role for the transcription factor WRINKLED1 in the biosynthesis of triacylglycerols during seed development. Frontiers in Plant Scienc | Sobic.007G07960 | bidirectiona | 80 | 68.4 | cTP | 0.9927 | cTP | 0.9826 | ortholo | no | 1021.1 |
| de novo FA synthesi | HAD | Hydroxyacyl-ACP Dehydratase | AT2G2223 Manuscript (Kuczynski; C.; McCorkle; S.; Keereetaweep; J.; Shanklin; J.; & Schwender; J. (2022). An expanded role for the transcription factor WRINKLED1 in the biosynthesis of triacylglycerols during seed development. Frontiers in Plant Scienc | Sobic.009G15090 | no | 73.96 | 66.05 | cTP | 0.9927 | cTP | 0.9927 | ortholo | no | 804.0 |
| de novo FA synthesi | MCMT | Malonyl-CoA : ACP Malonyltransferas | AT2G3020 Manuscript (Kuczynski; C.; McCorkle; S.; Keereetaweep; J.; Shanklin; J.; & Schwender; J. (2022). An expanded role for the transcription factor WRINKLED1 in the biosynthesis of triacylglycerols during seed development. Frontiers in Plant Scienc | Sobic.001G40340 | bidirectiona | 83.54 | 74.93 | mitochondio | 0.8881 | cTP | 0.8307 | no | no | 14094.4 |
| de novo FA synthesi | FAB2 / SAD1-6 | Stearoyl-ACP Desaturase | AT2G4371 Manuscript (Kuczynski; C.; McCorkle; S.; Keereetaweep; J.; Shanklin; J.; & Schwender; J. (2022). An expanded role for the transcription factor WRINKLED1 in the biosynthesis of triacylglycerols during seed development. Frontiers in Plant Scienc | Sobic.003G40400 | bidirectiona | 75.87 | 76.92 | cTP | 0.9637 | cTP | 0.8477 | ortholo | no | 19290.1 |
| de novo FA synthesi | FAB2 / SAD1-6 | Stearoyl-ACP Desaturase | AT2G4371 Manuscript (Kuczynski; C.; McCorkle; S.; Keereetaweep; J.; Shanklin; J.; & Schwender; J. (2022). An expanded role for the transcription factor WRINKLED1 in the biosynthesis of triacylglycerols during seed development. Frontiers in Plant Scienc | Sobic.006G04870 | yes | 81.22 | 76.92 | cTP | 0.9637 | cTP | 0.9972 | ortholo | no | 5481.2 |
| de novo FA synthesi | FAB2 / SAD1-6 | Stearoyl-ACP Desaturase | AT2G4371 Manuscript (Kuczynski; C.; McCorkle; S.; Keereetaweep; J.; Shanklin; J.; & Schwender; J. (2022). An expanded role for the transcription factor WRINKLED1 in the biosynthesis of triacylglycerols during seed development. Frontiers in Plant Scienc | Sobic.004G15330 | yes | 72.88 | 68.75 | cTP | 0.9637 | cTP | 0.9992 | ortholo | no | 14.8 |
| de novo FA synthesi | FAB2 / SAD1-6 | Stearoyl-ACP Desaturase | AT2G4371 Manuscript (Kuczynski; C.; McCorkle; S.; Keereetaweep; J.; Shanklin; J.; & Schwender; J. (2022). An expanded role for the transcription factor WRINKLED1 in the biosynthesis of triacylglycerols during seed development. Frontiers in Plant Scienc | Sobic.003G37710 | no | 71.3 | 65.07 | cTP | 0.9637 | cTP | 0.9065 | no | no | nd |
| de novo FA synthesi | FAB2 / SAD1-6 | Stearoyl-ACP Desaturase | AT2G4371 Manuscript (Kuczynski; C.; McCorkle; S.; Keereetaweep; J.; Shanklin; J.; & Schwender; J. (2022). An expanded role for the transcription factor WRINKLED1 in the biosynthesis of triacylglycerols during seed development. Frontiers in Plant Scienc | Sobic.001G28070 | no | 57.27 | 51.85 | cTP | 0.9637 | cTP | 0.9931 | ortholo | no | 1806.0 |
| de novo FA synthesi | FAB2 / SAD1-6 | Stearoyl-ACP Desaturase | AT2G4371 Manuscript (Kuczynski; C.; McCorkle; S.; Keereetaweep; J.; Shanklin; J.; & Schwender; J. (2022). An expanded role for the transcription factor WRINKLED1 in the biosynthesis of triacylglycerols during seed development. Frontiers in Plant Scienc | Sobic.001G33450 | no | 52.29 | 49.74 | cTP | 0.9637 | cTP | 0.8479 | ortholo | no | nd |
| de novo FA synthesi | FAB2 / SAD1-6 | Stearoyl-ACP Desaturase | AT2G4371 Manuscript (Kuczynski; C.; McCorkle; S.; Keereetaweep; J.; Shanklin; J.; & Schwender; J. (2022). An expanded role for the transcription factor WRINKLED1 in the biosynthesis of triacylglycerols during seed development. Frontiers in Plant Scienc | Sobic.007G07160 | no | 48.99 | 48.7 | cTP | 0.9637 | cTP | 0.9867 | ortholo | no | nd |
| de novo FA synthesi | FAB2 / SAD1-6 | Stearoyl-ACP Desaturase | AT2G4371 Manuscript (Kuczynski; C.; McCorkle; S.; Keereetaweep; J.; Shanklin; J.; & Schwender; J. (2022). An expanded role for the transcription factor WRINKLED1 in the biosynthesis of triacylglycerols during seed development. Frontiers in Plant Scienc | Sobic.002G25850 | no | 54.43 | 47.89 | cTP | 0.9637 | cTP | 0.7060 | ortholo | no | 22.1 |
| de novo FA synthesi | FAB2 / SAD1-6 | Stearoyl-ACP Desaturase | AT2G4371 Manuscript (Kuczynski; C.; McCorkle; S.; Keereetaweep; J.; Shanklin; J.; & Schwender; J. (2022). An expanded role for the transcription factor WRINKLED1 in the biosynthesis of triacylglycerols during seed development. Frontiers in Plant Scienc | Sobic.010G23530 | no | 54.29 | 47.87 | cTP | 0.9637 | cTP | 0.8511 | ortholo | no | nd |
| de novo FA synthesi | FAB2 / SAD1-6 | Stearoyl-ACP Desaturase | AT2G4371 Manuscript (Kuczynski; C.; McCorkle; S.; Keereetaweep; J.; Shanklin; J.; & Schwender; J. (2022). An expanded role for the transcription factor WRINKLED1 in the biosynthesis of triacylglycerols during seed development. Frontiers in Plant Scienc | Sobic.001G26530 | no | 52.03 | 45.94 | cTP | 0.9637 | cTP | 0.7420 | ortholo | no | nd |
| de novo FA synthesi FAB2 / SAD1-6 Stearoyl-ACP Desaturase AT2G4371 Manuscript (Kuczynski; C.; McCorkle; S.; Keereetaweep; J.; Shanklin; J.; & Schwender; J. (2022). An expanded role for the transcription factor WRINKLED1 in the biosynthesis of triacylglycerols during seed development. Frontiers in Plant Scienc Sobic.004G15755 no 53.25 33.33 cTP 0.9637 OTHER 0.8453 ortholo no nd | | | | | | | | | | | | | | |

| de novo FA synthesi | FAB2 / SAD1-6 Stearoyl-ACP Desaturase | | AT3G0261 Manuscript (Kuczynski; C.; McCorkle; S.; Keereetaweep; J.; Shanklin; J.; & Schwender; J. (2022). An expanded role for the transcription factor WRINKLED1 in the biosynthesis of triacylglycerols during seed development. Frontiers in Plant Scienc | Sobic.003G40400 | yes | 65.86 | 66.41 | cTP | 0.9277 | cTP | 0.8477 | ortholo | no | 19290.1 |
| --- | --- | --- | --- | --- | --- | --- | --- | --- | --- | --- | --- | --- | --- | --- |
| de novo FA synthesi | FAB2 / SAD1-6 Stearoyl-ACP Desaturase | | AT3G0261 Manuscript (Kuczynski; C.; McCorkle; S.; Keereetaweep; J.; Shanklin; J.; & Schwender; J. (2022). An expanded role for the transcription factor WRINKLED1 in the biosynthesis of triacylglycerols during seed development. Frontiers in Plant Scienc | Sobic.001G33450 | yes | 54 | 48.73 | cTP | 0.9277 | cTP | 0.8479 | ortholo | no | nd |
| de novo FA synthesi | FAB2 / SAD1-6 Stearoyl-ACP Desaturase | | AT3G0261 Manuscript (Kuczynski; C.; McCorkle; S.; Keereetaweep; J.; Shanklin; J.; & Schwender; J. (2022). An expanded role for the transcription factor WRINKLED1 in the biosynthesis of triacylglycerols during seed development. Frontiers in Plant Scienc | Sobic.002G25850 | no | 56.02 | 49.48 | cTP | 0.9277 | cTP | 0.7060 | ortholo | no | 22.1 |
| de novo FA synthesi | FAB2 / SAD1-6 Stearoyl-ACP Desaturase | | AT3G0261 Manuscript (Kuczynski; C.; McCorkle; S.; Keereetaweep; J.; Shanklin; J.; & Schwender; J. (2022). An expanded role for the transcription factor WRINKLED1 in the biosynthesis of triacylglycerols during seed development. Frontiers in Plant Scienc | Sobic.010G23530 | no | 54.47 | 48.95 | cTP | 0.9277 | cTP | 0.8511 | ortholo | no | nd |
| de novo FA synthesi | FAB2 / SAD1-6 Stearoyl-ACP Desaturase | | AT3G0261 Manuscript (Kuczynski; C.; McCorkle; S.; Keereetaweep; J.; Shanklin; J.; & Schwender; J. (2022). An expanded role for the transcription factor WRINKLED1 in the biosynthesis of triacylglycerols during seed development. Frontiers in Plant Scienc | Sobic.007G07160 | yes | 57.06 | 50 | cTP | 0.9277 | cTP | 0.9867 | ortholo | no | nd |
| de novo FA synthesi | FAB2 / SAD1-6 Stearoyl-ACP Desaturase | | AT3G0261 Manuscript (Kuczynski; C.; McCorkle; S.; Keereetaweep; J.; Shanklin; J.; & Schwender; J. (2022). An expanded role for the transcription factor WRINKLED1 in the biosynthesis of triacylglycerols during seed development. Frontiers in Plant Scienc | Sobic.001G26530 | yes | 52.34 | 46.19 | cTP | 0.9277 | cTP | 0.7420 | ortholo | no | nd |
| de novo FA synthesi | FAB2 / SAD1-6 Stearoyl-ACP Desaturase | | AT3G0261 Manuscript (Kuczynski; C.; McCorkle; S.; Keereetaweep; J.; Shanklin; J.; & Schwender; J. (2022). An expanded role for the transcription factor WRINKLED1 in the biosynthesis of triacylglycerols during seed development. Frontiers in Plant Scienc | Sobic.001G28070 | yes | 57.52 | 53.33 | cTP | 0.9277 | cTP | 0.9931 | ortholo | no | 1806.0 |
| de novo FA synthesi | FAB2 / SAD1-6 Stearoyl-ACP Desaturase | | AT3G0261 Manuscript (Kuczynski; C.; McCorkle; S.; Keereetaweep; J.; Shanklin; J.; & Schwender; J. (2022). An expanded role for the transcription factor WRINKLED1 in the biosynthesis of triacylglycerols during seed development. Frontiers in Plant Scienc | Sobic.003G37710 | no | 68.44 | 63.61 | cTP | 0.9277 | cTP | 0.9065 | no | no | nd |
| de novo FA synthesi | FAB2 / SAD1-6 Stearoyl-ACP Desaturase | | AT3G0261 Manuscript (Kuczynski; C.; McCorkle; S.; Keereetaweep; J.; Shanklin; J.; & Schwender; J. (2022). An expanded role for the transcription factor WRINKLED1 in the biosynthesis of triacylglycerols during seed development. Frontiers in Plant Scienc | Sobic.004G15330 | no | 66.67 | 60.96 | cTP | 0.9277 | cTP | 0.9992 | ortholo | no | 14.8 |
| de novo FA synthesi de novo FA synthesi | FAB2 / SAD1-6 Stearoyl-ACP Desaturase FAB2 / SAD1-6 Stearoyl-ACP Desaturase | | AT3G0261 Manuscript (Kuczynski; C.; McCorkle; S.; Keereetaweep; J.; Shanklin; J.; & Schwender; J. (2022). An expanded role for the transcription factor WRINKLED1 in the biosynthesis of triacylglycerols during seed development. Frontiers in Plant Scienc AT3G0261 Manuscript (Kuczynski; C.; McCorkle; S.; Keereetaweep; J.; Shanklin; J.; & Schwender; J. (2022). An expanded role for the transcription factor WRINKLED1 in the biosynthesis of triacylglycerols during seed development. Frontiers in Plant Scienc | Sobic.006G04870 Sobic.004G15755 | no yes | 68.88  47.92 | 67.54  32.06 | cTP cTP | 0.9277  0.9277 | cTP OTHER | 0.9972  0.8453 | ortholo ortholo | no no | 5481.2  nd |
| de novo FA synthesi | FAB2 / SAD1-6 Stearoyl-ACP Desaturase | | AT3G0262 Manuscript (Kuczynski; C.; McCorkle; S.; Keereetaweep; J.; Shanklin; J.; & Schwender; J. (2022). An expanded role for the transcription factor WRINKLED1 in the biosynthesis of triacylglycerols during seed development. Frontiers in Plant Scienc | Sobic.006G04870 | no | 69.97 | 66.84 | cTP | 0.9982 | cTP | 0.9972 | ortholo | no 5481.2 | |
| de novo FA synthesi | FAB2 / SAD1-6 Stearoyl-ACP Desaturase | | AT3G0262 Manuscript (Kuczynski; C.; McCorkle; S.; Keereetaweep; J.; Shanklin; J.; & Schwender; J. (2022). An expanded role for the transcription factor WRINKLED1 in the biosynthesis of triacylglycerols during seed development. Frontiers in Plant Scienc | Sobic.003G40400 | yes | 69.58 | 65.18 | cTP | 0.9982 | cTP | 0.8477 | ortholo | no 19290.1 | |
| de novo FA synthesi | FAB2 / SAD1-6 Stearoyl-ACP Desaturase | | AT3G0262 Manuscript (Kuczynski; C.; McCorkle; S.; Keereetaweep; J.; Shanklin; J.; & Schwender; J. (2022). An expanded role for the transcription factor WRINKLED1 in the biosynthesis of triacylglycerols during seed development. Frontiers in Plant Scienc | Sobic.003G37710 | no | 66.96 | 62.5 | cTP | 0.9982 | cTP | 0.9065 | no | no nd | |
| de novo FA synthesi | FAB2 / SAD1-6 Stearoyl-ACP Desaturase | | AT3G0262 Manuscript (Kuczynski; C.; McCorkle; S.; Keereetaweep; J.; Shanklin; J.; & Schwender; J. (2022). An expanded role for the transcription factor WRINKLED1 in the biosynthesis of triacylglycerols during seed development. Frontiers in Plant Scienc | Sobic.004G15330 | no | 65.17 | 60.15 | cTP | 0.9982 | cTP | 0.9992 | ortholo | no 14.8 | |
| de novo FA synthesi | FAB2 / SAD1-6 Stearoyl-ACP Desaturase | | AT3G0262 Manuscript (Kuczynski; C.; McCorkle; S.; Keereetaweep; J.; Shanklin; J.; & Schwender; J. (2022). An expanded role for the transcription factor WRINKLED1 in the biosynthesis of triacylglycerols during seed development. Frontiers in Plant Scienc | Sobic.001G28070 | no | 56.25 | 52.15 | cTP | 0.9982 | cTP | 0.9972 | ortholo | no 1806.0 | |
| de novo FA synthesi | FAB2 / SAD1-6 Stearoyl-ACP Desaturase | | AT3G0262 Manuscript (Kuczynski; C.; McCorkle; S.; Keereetaweep; J.; Shanklin; J.; & Schwender; J. (2022). An expanded role for the transcription factor WRINKLED1 in the biosynthesis of triacylglycerols during seed development. Frontiers in Plant Scienc | Sobic.010G23530 | no | 54.76 | 50.54 | cTP | 0.9982 | cTP | 0.8511 | ortholo | no nd | |
| de novo FA synthesi | FAB2 / SAD1-6 Stearoyl-ACP Desaturase | | AT3G0262 Manuscript (Kuczynski; C.; McCorkle; S.; Keereetaweep; J.; Shanklin; J.; & Schwender; J. (2022). An expanded role for the transcription factor WRINKLED1 in the biosynthesis of triacylglycerols during seed development. Frontiers in Plant Scienc | Sobic.002G25850 | yes | 56.33 | 50.53 | cTP | 0.9982 | cTP | 0.7060 | ortholo | no 22.1 | |
| de novo FA synthesi | FAB2 / SAD1-6 Stearoyl-ACP Desaturase | | AT3G0262 Manuscript (Kuczynski; C.; McCorkle; S.; Keereetaweep; J.; Shanklin; J.; & Schwender; J. (2022). An expanded role for the transcription factor WRINKLED1 in the biosynthesis of triacylglycerols during seed development. Frontiers in Plant Scienc | Sobic.007G07160 | no | 55.88 | 50.26 | cTP | 0.9982 | cTP | 0.9867 | ortholo | no nd | |
| de novo FA synthesi | FAB2 / SAD1-6 Stearoyl-ACP Desaturase | | AT3G0262 Manuscript (Kuczynski; C.; McCorkle; S.; Keereetaweep; J.; Shanklin; J.; & Schwender; J. (2022). An expanded role for the transcription factor WRINKLED1 in the biosynthesis of triacylglycerols during seed development. Frontiers in Plant Scienc | Sobic.001G33450 | no | 52.86 | 48.58 | cTP | 0.9982 | cTP | 0.8479 | ortholo | no nd | |
| de novo FA synthesi | FAB2 / SAD1-6 Stearoyl-ACP Desaturase | | AT3G0262 Manuscript (Kuczynski; C.; McCorkle; S.; Keereetaweep; J.; Shanklin; J.; & Schwender; J. (2022). An expanded role for the transcription factor WRINKLED1 in the biosynthesis of triacylglycerols during seed development. Frontiers in Plant Scienc | Sobic.001G26530 | no | 50.29 | 45.34 | cTP | 0.9982 | cTP | 0.7420 | ortholo | no nd | |
| de novo FA synthesi  de novo FA synthesi | FAB2 / SAD1-6 Stearoyl-ACP Desaturase  FAB2 / SAD1-6 Stearoyl-ACP Desaturase | | AT3G0262 Manuscript (Kuczynski; C.; McCorkle; S.; Keereetaweep; J.; Shanklin; J.; & Schwender; J. (2022). An expanded role for the transcription factor WRINKLED1 in the biosynthesis of triacylglycerols during seed development. Frontiers in Plant Scienc  AT3G0263 Manuscript (Kuczynski; C.; McCorkle; S.; Keereetaweep; J.; Shanklin; J.; & Schwender; J. (2022). An expanded role for the transcription factor WRINKLED1 in the biosynthesis of triacylglycerols during seed development. Frontiers in Plant Scienc | Sobic.004G15755  Sobic.003G40400 | no  bidirectiona | 45.83  75 | 32.32  75 | cTP  cTP | 0.9982  0.8342 | OTHER  cTP | 0.8453  0.8477 | ortholo  ortholo | no  no | nd  19290.1 |
| de novo FA synthesi | FAB2 / SAD1-6 Stearoyl-ACP Desaturase | | AT3G0263 Manuscript (Kuczynski; C.; McCorkle; S.; Keereetaweep; J.; Shanklin; J.; & Schwender; J. (2022). An expanded role for the transcription factor WRINKLED1 in the biosynthesis of triacylglycerols during seed development. Frontiers in Plant Scienc | Sobic.006G04870 | no | 77.21 | 74.35 | cTP | 0.8342 | cTP | 0.9972 | ortholo | no | 5481.2 |
| de novo FA synthesi | FAB2 / SAD1-6 Stearoyl-ACP Desaturase | | AT3G0263 Manuscript (Kuczynski; C.; McCorkle; S.; Keereetaweep; J.; Shanklin; J.; & Schwender; J. (2022). An expanded role for the transcription factor WRINKLED1 in the biosynthesis of triacylglycerols during seed development. Frontiers in Plant Scienc | Sobic.003G37710 | no | 71.77 | 66.58 | cTP | 0.8342 | cTP | 0.9065 | no | no | nd |
| de novo FA synthesi | FAB2 / SAD1-6 Stearoyl-ACP Desaturase | | AT3G0263 Manuscript (Kuczynski; C.; McCorkle; S.; Keereetaweep; J.; Shanklin; J.; & Schwender; J. (2022). An expanded role for the transcription factor WRINKLED1 in the biosynthesis of triacylglycerols during seed development. Frontiers in Plant Scienc | Sobic.004G15330 | no | 70.28 | 66.07 | cTP | 0.8342 | cTP | 0.9992 | ortholo | no | 14.8 |
| de novo FA synthesi | FAB2 / SAD1-6 Stearoyl-ACP Desaturase | | AT3G0263 Manuscript (Kuczynski; C.; McCorkle; S.; Keereetaweep; J.; Shanklin; J.; & Schwender; J. (2022). An expanded role for the transcription factor WRINKLED1 in the biosynthesis of triacylglycerols during seed development. Frontiers in Plant Scienc | Sobic.001G33450 | no | 51.71 | 50.52 | cTP | 0.8342 | cTP | 0.8479 | ortholo | no | nd |
| de novo FA synthesi | FAB2 / SAD1-6 Stearoyl-ACP Desaturase | | AT3G0263 Manuscript (Kuczynski; C.; McCorkle; S.; Keereetaweep; J.; Shanklin; J.; & Schwender; J. (2022). An expanded role for the transcription factor WRINKLED1 in the biosynthesis of triacylglycerols during seed development. Frontiers in Plant Scienc | Sobic.001G28070 | no | 55.09 | 50.41 | cTP | 0.8342 | cTP | 0.9972 | ortholo | no | 1806.0 |
| de novo FA synthesi | FAB2 / SAD1-6 Stearoyl-ACP Desaturase | | AT3G0263 Manuscript (Kuczynski; C.; McCorkle; S.; Keereetaweep; J.; Shanklin; J.; & Schwender; J. (2022). An expanded role for the transcription factor WRINKLED1 in the biosynthesis of triacylglycerols during seed development. Frontiers in Plant Scienc | Sobic.010G23530 | no | 54.46 | 48.67 | cTP | 0.8342 | cTP | 0.8511 | ortholo | no | nd |
| de novo FA synthesi | FAB2 / SAD1-6 Stearoyl-ACP Desaturase | | AT3G0263 Manuscript (Kuczynski; C.; McCorkle; S.; Keereetaweep; J.; Shanklin; J.; & Schwender; J. (2022). An expanded role for the transcription factor WRINKLED1 in the biosynthesis of triacylglycerols during seed development. Frontiers in Plant Scienc | Sobic.007G07160 | no | 53.85 | 47.93 | cTP | 0.8342 | cTP | 0.9867 | ortholo | no | nd |
| de novo FA synthesi | FAB2 / SAD1-6 Stearoyl-ACP Desaturase | | AT3G0263 Manuscript (Kuczynski; C.; McCorkle; S.; Keereetaweep; J.; Shanklin; J.; & Schwender; J. (2022). An expanded role for the transcription factor WRINKLED1 in the biosynthesis of triacylglycerols during seed development. Frontiers in Plant Scienc | Sobic.002G25850 | no | 53.07 | 47.09 | cTP | 0.8342 | cTP | 0.7060 | ortholo | no | 22.1 |
| de novo FA synthesi | FAB2 / SAD1-6 Stearoyl-ACP Desaturase | | AT3G0263 Manuscript (Kuczynski; C.; McCorkle; S.; Keereetaweep; J.; Shanklin; J.; & Schwender; J. (2022). An expanded role for the transcription factor WRINKLED1 in the biosynthesis of triacylglycerols during seed development. Frontiers in Plant Scienc | Sobic.001G26530 | no | 50.88 | 46.02 | cTP | 0.8342 | cTP | 0.7420 | ortholo | no | nd |
| de novo FA synthesi FAB2 / SAD1-6 Stearoyl-ACP Desaturase AT3G0263 Manuscript (Kuczynski; C.; McCorkle; S.; Keereetaweep; J.; Shanklin; J.; & Schwender; J. (2022). An expanded role for the transcription factor WRINKLED1 in the biosynthesis of triacylglycerols during seed development. Frontiers in Plant Scienc | | | | Sobic.004G15755 | no | 55.71 | 33.98 | cTP | 0.8342 OTHER 0.8453 | | | ortholo | no nd | |
| de novo FA synthesi | ACP | Acyl carrier protein | AT3G0502 Manuscript (Kuczynski; C.; McCorkle; S.; Keereetaweep; J.; Shanklin; J.; & Schwender; J. (2022). An expanded role for the transcription factor WRINKLED1 in the biosynthesis of triacylglycerols during seed development. Frontiers in Plant Scienc | Sobic.001G00570 | bidirectiona | 59.26 | 47.76 | cTP | 0.9998 cTP 0.9996 | | | ortholo | no | 186.6 |
| de novo FA synthesi | ACP | Acyl carrier protein | AT3G0502 Manuscript (Kuczynski; C.; McCorkle; S.; Keereetaweep; J.; Shanklin; J.; & Schwender; J. (2022). An expanded role for the transcription factor WRINKLED1 in the biosynthesis of triacylglycerols during seed development. Frontiers in Plant Scienc | Sobic.002G28040 | no | 48.41 | 46.46 | cTP | 0.9998 cTP 0.9986 | | | ortholo | no | 14213.7 |
| de novo FA synthesi | ACP | Acyl carrier protein | AT3G0502 Manuscript (Kuczynski; C.; McCorkle; S.; Keereetaweep; J.; Shanklin; J.; & Schwender; J. (2022). An expanded role for the transcription factor WRINKLED1 in the biosynthesis of triacylglycerols during seed development. Frontiers in Plant Scienc | Sobic.007G17680 | no | 48.82 | 48.82 | cTP | 0.9998 cTP 0.9998 | | | ortholo | no | 23607.5 |
| de novo FA synthesi | ACP | Acyl carrier protein | AT3G0502 Manuscript (Kuczynski; C.; McCorkle; S.; Keereetaweep; J.; Shanklin; J.; & Schwender; J. (2022). An expanded role for the transcription factor WRINKLED1 in the biosynthesis of triacylglycerols during seed development. Frontiers in Plant Scienc | Sobic.008G11640 | no | 55.77 | 45.86 | cTP | 0.9998 cTP 0.9995 | | | ortholo | no | 635.2 |
| de novo FA synthesi | HACPS | Holo-ACP Synthase | AT3G1147 Manuscript (Kuczynski; C.; McCorkle; S.; Keereetaweep; J.; Shanklin; J.; & Schwender; J. (2022). An expanded role for the transcription factor WRINKLED1 in the biosynthesis of triacylglycerols during seed development. Frontiers in Plant Scienc | Sobic.001G53710 | bidirectiona | 50.17 | 48.15 | mitochondio | 0.7610 OTHER 0.6491 | | | no | no | 1031.1 |
| de novo FA synthesi | AAE15/16 | Acyl-ACP Synthetase | AT3G2379 Manuscript (Kuczynski; C.; McCorkle; S.; Keereetaweep; J.; Shanklin; J.; & Schwender; J. (2022). An expanded role for the transcription factor WRINKLED1 in the biosynthesis of triacylglycerols during seed development. Frontiers in Plant Scienc | Sobic.001G01490 | bidirectiona | 67.02 | 63.47 | cTP | 0.9973 cTP 0.9792 | | | ortholo | no | 2207.9 |
| de novo FA synthesi | FAT-A/B | Acyl-ACP Thioesterase | AT3G2511 Manuscript (Kuczynski; C.; McCorkle; S.; Keereetaweep; J.; Shanklin; J.; & Schwender; J. (2022). An expanded role for the transcription factor WRINKLED1 in the biosynthesis of triacylglycerols during seed development. Frontiers in Plant Scienc | Sobic.002G25680 | bidirectiona | 78.84 | 67.81 | cTP | 0.4914 cTP 0.7381 | | | LDO | no | 5604.9 |
| de novo FA synthesi | FAT-A/B | Acyl-ACP Thioesterase | AT3G2511 Manuscript (Kuczynski; C.; McCorkle; S.; Keereetaweep; J.; Shanklin; J.; & Schwender; J. (2022). An expanded role for the transcription factor WRINKLED1 in the biosynthesis of triacylglycerols during seed development. Frontiers in Plant Scienc | Sobic.002G25690 | yes | 76.19 | 65.62 | cTP | 0.4914 cTP 0.6215 | | | ortholo | no | 2454.2 |
| de novo FA synthesi | KAR | Ketoacyl-ACP Reductase | AT3G4617 Manuscript (Kuczynski; C.; McCorkle; S.; Keereetaweep; J.; Shanklin; J.; & Schwender; J. (2022). An expanded role for the transcription factor WRINKLED1 in the biosynthesis of triacylglycerols during seed development. Frontiers in Plant Scienc | Sobic.007G20980 | yes | 60.89 | 59.42 | OTHER | 0.9997 OTHER 0.9648 | | | ortholo | no | 1109.8 |
| de novo FA synthesi | KAR | Ketoacyl-ACP Reductase | AT3G5529 Manuscript (Kuczynski; C.; McCorkle; S.; Keereetaweep; J.; Shanklin; J.; & Schwender; J. (2022). An expanded role for the transcription factor WRINKLED1 in the biosynthesis of triacylglycerols during seed development. Frontiers in Plant Scienc | Sobic.007G20980 | bidirectiona | 63.7 | 62.32 | OTHER | 0.9997 OTHER 0.9648 | | | LDO | no | 1109.8 |
| de novo FA synthesi | KAR | Ketoacyl-ACP Reductase | AT3G5531 Manuscript (Kuczynski; C.; McCorkle; S.; Keereetaweep; J.; Shanklin; J.; & Schwender; J. (2022). An expanded role for the transcription factor WRINKLED1 in the biosynthesis of triacylglycerols during seed development. Frontiers in Plant Scienc | Sobic.007G20980 | yes | 61.25 | 60 | OTHER | 0.9996 OTHER 0.9648 | | | ortholo | no | 1109.8 |
| de novo FA synthesi | FAT-A/B | Acyl-ACP Thioesterase | AT4G1305 Manuscript (Kuczynski; C.; McCorkle; S.; Keereetaweep; J.; Shanklin; J.; & Schwender; J. (2022). An expanded role for the transcription factor WRINKLED1 in the biosynthesis of triacylglycerols during seed development. Frontiers in Plant Scienc | Sobic.002G25680 | yes | 76.85 | 66.2 | OTHER | 0.6800 cTP 0.7381 | | | ortholo | no | 5604.9 |
| de novo FA synthesi | FAT-A/B | Acyl-ACP Thioesterase | AT4G1305 Manuscript (Kuczynski; C.; McCorkle; S.; Keereetaweep; J.; Shanklin; J.; & Schwender; J. (2022). An expanded role for the transcription factor WRINKLED1 in the biosynthesis of triacylglycerols during seed development. Frontiers in Plant Scienc | Sobic.002G25690 | no | 74.59 | 64.9 | OTHER | 0.6800 cTP 0.6215 | | | ortholo | no | 2454.2 |
| de novo FA synthesi | AAE15/16 | Acyl-ACP Synthetase | AT4G1407 Manuscript (Kuczynski; C.; McCorkle; S.; Keereetaweep; J.; Shanklin; J.; & Schwender; J. (2022). An expanded role for the transcription factor WRINKLED1 in the biosynthesis of triacylglycerols during seed development. Frontiers in Plant Scienc | Sobic.001G01490 | yes | 64.47 | 61.27 | OTHER | 0.5758 cTP 0.9792 | | | LDO | no | 2207.9 |
| de novo FA synthesi | ACP | Acyl carrier protein | AT4G2505 Manuscript (Kuczynski; C.; McCorkle; S.; Keereetaweep; J.; Shanklin; J.; & Schwender; J. (2022). An expanded role for the transcription factor WRINKLED1 in the biosynthesis of triacylglycerols during seed development. Frontiers in Plant Scienc | Sobic.005G12810 | bidirectiona | 54.07 | 61.83 | cTP | 0.9982 cTP 0.9992 | | | LDO | no | 4341.2 |
| de novo FA synthesi | BCA5 | beta carbonic anhydrase 5 | AT4G3358 Manuscript (Kuczynski; C.; McCorkle; S.; Keereetaweep; J.; Shanklin; J.; & Schwender; J. (2022). An expanded role for the transcription factor WRINKLED1 in the biosynthesis of triacylglycerols during seed development. Frontiers in Plant Scienc | Sobic.002G23010 | bidirectiona | 60.94 | 53.69 | cTP | 0.9928 OTHER 0.9841 | | | LDO | yes | 11305.7 |
| de novo FA synthesi | HAD | Hydroxyacyl-ACP Dehydratase | AT5G1016 Manuscript (Kuczynski; C.; McCorkle; S.; Keereetaweep; J.; Shanklin; J.; & Schwender; J. (2022). An expanded role for the transcription factor WRINKLED1 in the biosynthesis of triacylglycerols during seed development. Frontiers in Plant Scienc | Sobic.007G07960 | no | 75 | 69.67 | cTP | 0.9957 | cTP | 0.9826 | ortholo | no | 1021.1 |
| de novo FA synthesi | HAD | Hydroxyacyl-ACP Dehydratase | AT5G1016 Manuscript (Kuczynski; C.; McCorkle; S.; Keereetaweep; J.; Shanklin; J.; & Schwender; J. (2022). An expanded role for the transcription factor WRINKLED1 in the biosynthesis of triacylglycerols during seed development. Frontiers in Plant Scienc | Sobic.009G15090 | bidirectiona | 70.23 | 67.76 | cTP | 0.9957 | cTP | 0.9927 | LDO | no | 804.0 |
| de novo FA synthesi | FAB2 / SAD1-6 | Stearoyl-ACP Desaturase | AT5G1623 Manuscript (Kuczynski; C.; McCorkle; S.; Keereetaweep; J.; Shanklin; J.; & Schwender; J. (2022). An expanded role for the transcription factor WRINKLED1 in the biosynthesis of triacylglycerols during seed development. Frontiers in Plant Scienc | Sobic.003G40400 | bidirectiona | 65.67 | 66.58 | cTP | 0.9983 | cTP | 0.8477 | ortholo | no | 19290.1 |
| de novo FA synthesi | FAB2 / SAD1-6 | Stearoyl-ACP Desaturase | AT5G1623 Manuscript (Kuczynski; C.; McCorkle; S.; Keereetaweep; J.; Shanklin; J.; & Schwender; J. (2022). An expanded role for the transcription factor WRINKLED1 in the biosynthesis of triacylglycerols during seed development. Frontiers in Plant Scienc | Sobic.001G33450 | no | 53.16 | 49.35 | cTP | 0.9983 | cTP | 0.8479 | ortholo | no | nd |
| de novo FA synthesi | FAB2 / SAD1-6 | Stearoyl-ACP Desaturase | AT5G1623 Manuscript (Kuczynski; C.; McCorkle; S.; Keereetaweep; J.; Shanklin; J.; & Schwender; J. (2022). An expanded role for the transcription factor WRINKLED1 in the biosynthesis of triacylglycerols during seed development. Frontiers in Plant Scienc | Sobic.002G25850 | no | 54.27 | 48.66 | cTP | 0.9983 | cTP | 0.7060 | ortholo | no | 22.1 |
| de novo FA synthesi | FAB2 / SAD1-6 | Stearoyl-ACP Desaturase | AT5G1623 Manuscript (Kuczynski; C.; McCorkle; S.; Keereetaweep; J.; Shanklin; J.; & Schwender; J. (2022). An expanded role for the transcription factor WRINKLED1 in the biosynthesis of triacylglycerols during seed development. Frontiers in Plant Scienc | Sobic.010G23530 | yes | 54.23 | 50.13 | cTP | 0.9983 | cTP | 0.8511 | ortholo | no | nd |
| de novo FA synthesi | FAB2 / SAD1-6 | Stearoyl-ACP Desaturase | AT5G1623 Manuscript (Kuczynski; C.; McCorkle; S.; Keereetaweep; J.; Shanklin; J.; & Schwender; J. (2022). An expanded role for the transcription factor WRINKLED1 in the biosynthesis of triacylglycerols during seed development. Frontiers in Plant Scienc | Sobic.007G07160 | no | 57.57 | 49.87 | cTP | 0.9983 | cTP | 0.9867 | ortholo | no | nd |
| de novo FA synthesi | FAB2 / SAD1-6 | Stearoyl-ACP Desaturase | AT5G1623 Manuscript (Kuczynski; C.; McCorkle; S.; Keereetaweep; J.; Shanklin; J.; & Schwender; J. (2022). An expanded role for the transcription factor WRINKLED1 in the biosynthesis of triacylglycerols during seed development. Frontiers in Plant Scienc | Sobic.001G26530 | no | 49.46 | 46.75 | cTP | 0.9983 | cTP | 0.7420 | ortholo | no | nd |
| de novo FA synthesi | FAB2 / SAD1-6 | Stearoyl-ACP Desaturase | AT5G1623 Manuscript (Kuczynski; C.; McCorkle; S.; Keereetaweep; J.; Shanklin; J.; & Schwender; J. (2022). An expanded role for the transcription factor WRINKLED1 in the biosynthesis of triacylglycerols during seed development. Frontiers in Plant Scienc | Sobic.001G28070 | no | 56.25 | 52.89 | cTP | 0.9983 | cTP | 0.9931 | ortholo | no | 1806.0 |
| de novo FA synthesi | FAB2 / SAD1-6 | Stearoyl-ACP Desaturase | AT5G1623 Manuscript (Kuczynski; C.; McCorkle; S.; Keereetaweep; J.; Shanklin; J.; & Schwender; J. (2022). An expanded role for the transcription factor WRINKLED1 in the biosynthesis of triacylglycerols during seed development. Frontiers in Plant Scienc | Sobic.003G37710 | no | 66.37 | 62.36 | cTP | 0.9983 | cTP | 0.9065 | no | no | nd |
| de novo FA synthesi | FAB2 / SAD1-6 | Stearoyl-ACP Desaturase | AT5G1623 Manuscript (Kuczynski; C.; McCorkle; S.; Keereetaweep; J.; Shanklin; J.; & Schwender; J. (2022). An expanded role for the transcription factor WRINKLED1 in the biosynthesis of triacylglycerols during seed development. Frontiers in Plant Scienc | Sobic.004G15330 | no | 61.34 | 60.57 | cTP | 0.9983 | cTP | 0.9992 | ortholo | no | 14.8 |
| de novo FA synthesi | FAB2 / SAD1-6 | Stearoyl-ACP Desaturase | AT5G1623 Manuscript (Kuczynski; C.; McCorkle; S.; Keereetaweep; J.; Shanklin; J.; & Schwender; J. (2022). An expanded role for the transcription factor WRINKLED1 in the biosynthesis of triacylglycerols during seed development. Frontiers in Plant Scienc | Sobic.006G04870 | no | 70.94 | 66.93 | cTP | 0.9983 | cTP | 0.9972 | ortholo | no | 5481.2 |
| de novo FA synthesi FAB2 / SAD1-6 Stearoyl-ACP Desaturase AT5G1623 Manuscript (Kuczynski; C.; McCorkle; S.; Keereetaweep; J.; Shanklin; J.; & Schwender; J. (2022). An expanded role for the transcription factor WRINKLED1 in the biosynthesis of triacylglycerols during seed development. Frontiers in Plant Scienc Sobic.004G15755 no 47.37 34.11 cTP 0.9983 OTHER 0.8453 ortholo no nd | | | | | | | | | | | | | | |
| de novo FA synthesi | FAB2 / SAD1-6 Stearoyl-ACP Desaturase | | AT5G1624 Manuscript (Kuczynski; C.; McCorkle; S.; Keereetaweep; J.; Shanklin; J.; & Schwender; J. (2022). An expanded role for the transcription factor WRINKLED1 in the biosynthesis of triacylglycerols during seed development. Frontiers in Plant Scienc | Sobic.003G40400 | yes | 71.61 | 72.73 | OTHER | 0.7916 | cTP | 0.8477 | ortholo | no | 19290.1 |
| de novo FA synthesi | FAB2 / SAD1-6 Stearoyl-ACP Desaturase | | AT5G1624 Manuscript (Kuczynski; C.; McCorkle; S.; Keereetaweep; J.; Shanklin; J.; & Schwender; J. (2022). An expanded role for the transcription factor WRINKLED1 in the biosynthesis of triacylglycerols during seed development. Frontiers in Plant Scienc | Sobic.001G33450 | no | 51.71 | 50.66 | OTHER | 0.7916 | cTP | 0.8479 | ortholo | no | nd |
| de novo FA synthesi | FAB2 / SAD1-6 Stearoyl-ACP Desaturase | | AT5G1624 Manuscript (Kuczynski; C.; McCorkle; S.; Keereetaweep; J.; Shanklin; J.; & Schwender; J. (2022). An expanded role for the transcription factor WRINKLED1 in the biosynthesis of triacylglycerols during seed development. Frontiers in Plant Scienc | Sobic.002G25850 | no | 53.07 | 47.61 | OTHER | 0.7916 | cTP | 0.7060 | ortholo | no | 22.1 |
| de novo FA synthesi | FAB2 / SAD1-6 Stearoyl-ACP Desaturase | | AT5G1624 Manuscript (Kuczynski; C.; McCorkle; S.; Keereetaweep; J.; Shanklin; J.; & Schwender; J. (2022). An expanded role for the transcription factor WRINKLED1 in the biosynthesis of triacylglycerols during seed development. Frontiers in Plant Scienc | Sobic.010G23530 | no | 54.15 | 47.59 | OTHER | 0.7916 | cTP | 0.8511 | ortholo | no | nd |
| de novo FA synthesi | FAB2 / SAD1-6 Stearoyl-ACP Desaturase | | AT5G1624 Manuscript (Kuczynski; C.; McCorkle; S.; Keereetaweep; J.; Shanklin; J.; & Schwender; J. (2022). An expanded role for the transcription factor WRINKLED1 in the biosynthesis of triacylglycerols during seed development. Frontiers in Plant Scienc | Sobic.007G07160 | no | 53.85 | 48.43 | OTHER | 0.7916 | cTP | 0.9867 | ortholo | no | nd |
| de novo FA synthesi | FAB2 / SAD1-6 Stearoyl-ACP Desaturase | | AT5G1624 Manuscript (Kuczynski; C.; McCorkle; S.; Keereetaweep; J.; Shanklin; J.; & Schwender; J. (2022). An expanded role for the transcription factor WRINKLED1 in the biosynthesis of triacylglycerols during seed development. Frontiers in Plant Scienc | Sobic.001G26530 | no | 50.29 | 45.22 | OTHER | 0.7916 | cTP | 0.7420 | ortholo | no | nd |
| de novo FA synthesi | FAB2 / SAD1-6 Stearoyl-ACP Desaturase | | AT5G1624 Manuscript (Kuczynski; C.; McCorkle; S.; Keereetaweep; J.; Shanklin; J.; & Schwender; J. (2022). An expanded role for the transcription factor WRINKLED1 in the biosynthesis of triacylglycerols during seed development. Frontiers in Plant Scienc | Sobic.001G28070 | no | 55.09 | 49.73 | OTHER | 0.7916 | cTP | 0.9931 | ortholo | no | 1806.0 |
| de novo FA synthesi | FAB2 / SAD1-6 Stearoyl-ACP Desaturase | | AT5G1624 Manuscript (Kuczynski; C.; McCorkle; S.; Keereetaweep; J.; Shanklin; J.; & Schwender; J. (2022). An expanded role for the transcription factor WRINKLED1 in the biosynthesis of triacylglycerols during seed development. Frontiers in Plant Scienc | Sobic.003G37710 | no | 68.96 | 63.51 | OTHER | 0.7916 | cTP | 0.9065 | no | no | nd |
| de novo FA synthesi | FAB2 / SAD1-6 Stearoyl-ACP Desaturase | | AT5G1624 Manuscript (Kuczynski; C.; McCorkle; S.; Keereetaweep; J.; Shanklin; J.; & Schwender; J. (2022). An expanded role for the transcription factor WRINKLED1 in the biosynthesis of triacylglycerols during seed development. Frontiers in Plant Scienc | Sobic.004G15330 | no | 70.06 | 65.13 | OTHER | 0.7916 | cTP | 0.9992 | ortholo | no | 14.8 |
| de novo FA synthesi | FAB2 / SAD1-6 Stearoyl-ACP Desaturase | | AT5G1624 Manuscript (Kuczynski; C.; McCorkle; S.; Keereetaweep; J.; Shanklin; J.; & Schwender; J. (2022). An expanded role for the transcription factor WRINKLED1 in the biosynthesis of triacylglycerols during seed development. Frontiers in Plant Scienc | Sobic.006G04870 | no | 78.03 | 72.15 | OTHER | 0.7916 | cTP | 0.9972 | ortholo | no | 5481.2 |
| de novo FA synthesi | FAB2 / SAD1-6 Stearoyl-ACP Desaturase | | AT5G1624 Manuscript (Kuczynski; C.; McCorkle; S.; Keereetaweep; J.; Shanklin; J.; & Schwender; J. (2022). An expanded role for the transcription factor WRINKLED1 in the biosynthesis of triacylglycerols during seed development. Frontiers in Plant Scienc | Sobic.001G28070 | no | 55.09 | 49.73 | OTHER | 0.7916 | cTP | 0.9931 | ortholo | no | 1806.0 |
| de novo FA synthesi | FAB2 / SAD1-6 Stearoyl-ACP Desaturase | | AT5G1624 Manuscript (Kuczynski; C.; McCorkle; S.; Keereetaweep; J.; Shanklin; J.; & Schwender; J. (2022). An expanded role for the transcription factor WRINKLED1 in the biosynthesis of triacylglycerols during seed development. Frontiers in Plant Scienc | Sobic.010G23530 | no | 54.15 | 47.59 | OTHER | 0.7916 | cTP | 0.8511 | ortholo | no | nd |
| de novo FA synthesi FAB2 / SAD1-6 Stearoyl-ACP Desaturase AT5G1624 Manuscript (Kuczynski; C.; McCorkle; S.; Keereetaweep; J.; Shanklin; J.; & Schwender; J. (2022). An expanded role for the transcription factor WRINKLED1 in the biosynthesis of triacylglycerols during seed development. Frontiers in Plant Scienc | | | | Sobic.004G15755 | no | 54.29 | 33.98 | OTHER | 0.7916 OTHER 0.8453 | | | ortholo | no nd | |
| de novo FA synthesi | ACP | Acyl carrier protein | AT5G2720 Manuscript (Kuczynski; C.; McCorkle; S.; Keereetaweep; J.; Shanklin; J.; & Schwender; J. (2022). An expanded role for the transcription factor WRINKLED1 in the biosynthesis of triacylglycerols during seed development. Frontiers in Plant Scienc | Sobic.001G00570 | yes | 55.45 | 44.12 | cTP | 0.9996 cTP 0.9996 | | | ortholo | no 186.6 | |
| de novo FA synthesi | ACP | Acyl carrier protein | AT5G2720 Manuscript (Kuczynski; C.; McCorkle; S.; Keereetaweep; J.; Shanklin; J.; & Schwender; J. (2022). An expanded role for the transcription factor WRINKLED1 in the biosynthesis of triacylglycerols during seed development. Frontiers in Plant Scienc | Sobic.002G28040 | yes | 47.76 | 48.06 | cTP | 0.9996 cTP 0.9986 | | | ortholo | no 14213.7 | |
| de novo FA synthesi | ACP | Acyl carrier protein | AT5G2720 Manuscript (Kuczynski; C.; McCorkle; S.; Keereetaweep; J.; Shanklin; J.; & Schwender; J. (2022). An expanded role for the transcription factor WRINKLED1 in the biosynthesis of triacylglycerols during seed development. Frontiers in Plant Scienc | Sobic.007G17680 | no | 56.36 | 50 | cTP | 0.9996 cTP 0.9998 | | | ortholo | no 23607.5 | |
| de novo FA synthesi | ACP | Acyl carrier protein | AT5G2720 Manuscript (Kuczynski; C.; McCorkle; S.; Keereetaweep; J.; Shanklin; J.; & Schwender; J. (2022). An expanded role for the transcription factor WRINKLED1 in the biosynthesis of triacylglycerols during seed development. Frontiers in Plant Scienc | Sobic.008G11640 | no | 59.18 | 44.44 | cTP | 0.9996 cTP 0.9995 | | | ortholo | no 635.2 | |
| de novo FA synthesi | KAS I | Ketoacyl-ACP Synthase I | AT5G4629 Manuscript (Kuczynski; C.; McCorkle; S.; Keereetaweep; J.; Shanklin; J.; & Schwender; J. (2022). An expanded role for the transcription factor WRINKLED1 in the biosynthesis of triacylglycerols during seed development. Frontiers in Plant Scienc | Sobic.002G40130 | yes | 68.42 | 74.57 | cTP | 0.6242 OTHER 0.9860 | | | no | yes nd | |
| de novo FA synthesi | KAS I | Ketoacyl-ACP Synthase I | AT5G4629 Manuscript (Kuczynski; C.; McCorkle; S.; Keereetaweep; J.; Shanklin; J.; & Schwender; J. (2022). An expanded role for the transcription factor WRINKLED1 in the biosynthesis of triacylglycerols during seed development. Frontiers in Plant Scienc | Sobic.010G07350 | bidirectiona | 83.14 | 81.92 | cTP | 0.6242 cTP 0.9322 | | | LDO | yes 6391.8 | |
| epidermal lipid synthes | KCS | Ketoacyl-CoA Synthase | AT1G0112 Manuscript (Kuczynski; C.; McCorkle; S.; Keereetaweep; J.; Shanklin; J.; & Schwender; J. (2022). An expanded role for the transcription factor WRINKLED1 in the biosynthesis of triacylglycerols during seed development. Frontiers in Plant Scienc | Sobic.010G18150 | no | 63.51 | 62.33 | OTHER | 1.0000 OTHER 0.9988 | | | no | no 110.4 | |
| epidermal lipid synthes | KCS | Ketoacyl-CoA Synthase | AT1G0112 Manuscript (Kuczynski; C.; McCorkle; S.; Keereetaweep; J.; Shanklin; J.; & Schwender; J. (2022). An expanded role for the transcription factor WRINKLED1 in the biosynthesis of triacylglycerols during seed development. Frontiers in Plant Scienc | Sobic.009G24100 | no | 62.5 | 63.04 | OTHER | 1.0000 OTHER 0.9999 | | | no | no 632.4 | |
| epidermal lipid synthes | KCS | Ketoacyl-CoA Synthase | AT1G0112 Manuscript (Kuczynski; C.; McCorkle; S.; Keereetaweep; J.; Shanklin; J.; & Schwender; J. (2022). An expanded role for the transcription factor WRINKLED1 in the biosynthesis of triacylglycerols during seed development. Frontiers in Plant Scienc | Sobic.004G08680 | no | 62.1 | 60.31 | OTHER | 1.0000 OTHER 1.0000 | | | no | no 1524.2 | |
| epidermal lipid synthes | KCS | Ketoacyl-CoA Synthase | AT1G0112 Manuscript (Kuczynski; C.; McCorkle; S.; Keereetaweep; J.; Shanklin; J.; & Schwender; J. (2022). An expanded role for the transcription factor WRINKLED1 in the biosynthesis of triacylglycerols during seed development. Frontiers in Plant Scienc | Sobic.001G26450 | yes | 61.09 | 62.65 | OTHER | 1.0000 OTHER 0.9999 | | | LDO | no 21.5 | |
| epidermal lipid synthes | KCS | Ketoacyl-CoA Synthase | AT1G0112 Manuscript (Kuczynski; C.; McCorkle; S.; Keereetaweep; J.; Shanklin; J.; & Schwender; J. (2022). An expanded role for the transcription factor WRINKLED1 in the biosynthesis of triacylglycerols during seed development. Frontiers in Plant Scienc | Sobic.001G43810 | bidirectiona | 59.46 | 62.52 | OTHER | 1.0000 OTHER 1.0000 | | | ortholo | no 2121.4 | |
| epidermal lipid synthes | KCS | Ketoacyl-CoA Synthase | AT1G0112 Manuscript (Kuczynski; C.; McCorkle; S.; Keereetaweep; J.; Shanklin; J.; & Schwender; J. (2022). An expanded role for the transcription factor WRINKLED1 in the biosynthesis of triacylglycerols during seed development. Frontiers in Plant Scienc | Sobic.010G18150 | no | 63.51 | 63.51 | OTHER | 1.0000 OTHER 0.9988 | | | no | no 110.4 | |
| epidermal lipid synthes | KCS | Ketoacyl-CoA Synthase | AT1G0112 Manuscript (Kuczynski; C.; McCorkle; S.; Keereetaweep; J.; Shanklin; J.; & Schwender; J. (2022). An expanded role for the transcription factor WRINKLED1 in the biosynthesis of triacylglycerols during seed development. Frontiers in Plant Scienc | Sobic.009G24100 | no | 62.5 | 62.5 | OTHER | 1.0000 OTHER 0.9999 | | | no | no 632.4 | |
| epidermal lipid synthes | KCS | Ketoacyl-CoA Synthase | AT1G0112 Manuscript (Kuczynski; C.; McCorkle; S.; Keereetaweep; J.; Shanklin; J.; & Schwender; J. (2022). An expanded role for the transcription factor WRINKLED1 in the biosynthesis of triacylglycerols during seed development. Frontiers in Plant Scienc | Sobic.004G08680 | no | 62.1 | 62.1 | OTHER | 1.0000 OTHER 1.0000 | | | no | no 1524.2 | |
| epidermal lipid synthes | KCS | Ketoacyl-CoA Synthase | AT1G0112 Manuscript (Kuczynski; C.; McCorkle; S.; Keereetaweep; J.; Shanklin; J.; & Schwender; J. (2022). An expanded role for the transcription factor WRINKLED1 in the biosynthesis of triacylglycerols during seed development. Frontiers in Plant Scienc | Sobic.001G26450 | yes | 61.09 | 61.09 | OTHER | 1.0000 OTHER 0.9999 | | | LDO | no 21.5 | |
| epidermal lipid synthes | KCS | Ketoacyl-CoA Synthase | AT1G0422 Manuscript (Kuczynski; C.; McCorkle; S.; Keereetaweep; J.; Shanklin; J.; & Schwender; J. (2022). An expanded role for the transcription factor WRINKLED1 in the biosynthesis of triacylglycerols during seed development. Frontiers in Plant Scienc | Sobic.010G18150 | yes | 71.94 | 71.15 | OTHER | 1.0000 OTHER 0.9988 | | | no | no 110.4 | |
| epidermal lipid synthes | KCS | Ketoacyl-CoA Synthase | AT1G0422 Manuscript (Kuczynski; C.; McCorkle; S.; Keereetaweep; J.; Shanklin; J.; & Schwender; J. (2022). An expanded role for the transcription factor WRINKLED1 in the biosynthesis of triacylglycerols during seed development. Frontiers in Plant Scienc | Sobic.004G08680 | no | 70.16 | 68.99 | OTHER | 1.0000 OTHER 1.0000 | | | no | no 1524.2 | |
| epidermal lipid synthes | KCS | Ketoacyl-CoA Synthase | AT1G0422 Manuscript (Kuczynski; C.; McCorkle; S.; Keereetaweep; J.; Shanklin; J.; & Schwender; J. (2022). An expanded role for the transcription factor WRINKLED1 in the biosynthesis of triacylglycerols during seed development. Frontiers in Plant Scienc | Sobic.005G16870 | yes | 69.12 | 67.79 | OTHER | 1.0000 OTHER 0.9985 | | | LDO | no 3001.9 | |
| epidermal lipid synthes | KCS | Ketoacyl-CoA Synthase | AT1G0422 Manuscript (Kuczynski; C.; McCorkle; S.; Keereetaweep; J.; Shanklin; J.; & Schwender; J. (2022). An expanded role for the transcription factor WRINKLED1 in the biosynthesis of triacylglycerols during seed development. Frontiers in Plant Scienc | Sobic.009G24100 | no | 67.93 | 66.54 | OTHER | 1.0000 OTHER 0.9999 | | | no | no 632.4 | |
| epidermal lipid synthes | KCS | Ketoacyl-CoA Synthase | AT1G0422 Manuscript (Kuczynski; C.; McCorkle; S.; Keereetaweep; J.; Shanklin; J.; & Schwender; J. (2022). An expanded role for the transcription factor WRINKLED1 in the biosynthesis of triacylglycerols during seed development. Frontiers in Plant Scienc | Sobic.009G23610 | no | 64.86 | 62.4 | OTHER | 1.0000 OTHER 0.9997 | | | no | no 5240.3 | |
| epidermal lipid synthes | KCS | Ketoacyl-CoA Synthase | AT1G0422 Manuscript (Kuczynski; C.; McCorkle; S.; Keereetaweep; J.; Shanklin; J.; & Schwender; J. (2022). An expanded role for the transcription factor WRINKLED1 in the biosynthesis of triacylglycerols during seed development. Frontiers in Plant Scienc | Sobic.010G18150 | yes | 71.94 | 71.94 | OTHER | 1.0000 OTHER 0.9988 | | | no | no 110.4 | |
| epidermal lipid synthes | KCS | Ketoacyl-CoA Synthase | AT1G0422 Manuscript (Kuczynski; C.; McCorkle; S.; Keereetaweep; J.; Shanklin; J.; & Schwender; J. (2022). An expanded role for the transcription factor WRINKLED1 in the biosynthesis of triacylglycerols during seed development. Frontiers in Plant Scienc | Sobic.004G08680 | no | 70.16 | 70.16 | OTHER | 1.0000 OTHER 1.0000 | | | no | no 1524.2 | |
| epidermal lipid synthes | KCS | Ketoacyl-CoA Synthase | AT1G0422 Manuscript (Kuczynski; C.; McCorkle; S.; Keereetaweep; J.; Shanklin; J.; & Schwender; J. (2022). An expanded role for the transcription factor WRINKLED1 in the biosynthesis of triacylglycerols during seed development. Frontiers in Plant Scienc | Sobic.005G16870 | yes | 69.12 | 69.12 | OTHER | 1.0000 OTHER 0.9985 | | | LDO | no 3001.9 | |
| epidermal lipid synthes | KCS | Ketoacyl-CoA Synthase | AT1G0422 Manuscript (Kuczynski; C.; McCorkle; S.; Keereetaweep; J.; Shanklin; J.; & Schwender; J. (2022). An expanded role for the transcription factor WRINKLED1 in the biosynthesis of triacylglycerols during seed development. Frontiers in Plant Scienc | Sobic.009G24100 | no | 67.93 | 67.93 | OTHER | 1.0000 OTHER 0.9999 | | | no | no 632.4 | |
| epidermal lipid synthes | KCS | Ketoacyl-CoA Synthase | AT1G0422 Manuscript (Kuczynski; C.; McCorkle; S.; Keereetaweep; J.; Shanklin; J.; & Schwender; J. (2022). An expanded role for the transcription factor WRINKLED1 in the biosynthesis of triacylglycerols during seed development. Frontiers in Plant Scienc | Sobic.005G16900 | no | 66.07 | 66.07 | OTHER | 1.0000 OTHER 0.9810 | | | no | no nd | |
| epidermal lipid synthes | KCS | Ketoacyl-CoA Synthase | AT1G0422 Manuscript (Kuczynski; C.; McCorkle; S.; Keereetaweep; J.; Shanklin; J.; & Schwender; J. (2022). An expanded role for the transcription factor WRINKLED1 in the biosynthesis of triacylglycerols during seed development. Frontiers in Plant Scienc | Sobic.009G23610 | no | 64.86 | 64.86 | OTHER | 1.0000 OTHER 0.9997 | | | no | no 5240.3 | |
| epidermal lipid synthes | KCS | Ketoacyl-CoA Synthase | AT1G0422 Manuscript (Kuczynski; C.; McCorkle; S.; Keereetaweep; J.; Shanklin; J.; & Schwender; J. (2022). An expanded role for the transcription factor WRINKLED1 in the biosynthesis of triacylglycerols during seed development. Frontiers in Plant Scienc | Sobic.001G45320 | no | 60.89 | 60.89 | OTHER | 1.0000 OTHER 0.9580 | | | no | no 2162.6 | |
| epidermal lipid synthes | KCS | Ketoacyl-CoA Synthase | AT1G0422 Manuscript (Kuczynski; C.; McCorkle; S.; Keereetaweep; J.; Shanklin; J.; & Schwender; J. (2022). An expanded role for the transcription factor WRINKLED1 in the biosynthesis of triacylglycerols during seed development. Frontiers in Plant Scienc | Sobic.001G49550 | no | 60.28 | 60.28 | OTHER | 1.0000 OTHER 0.9290 | | | no | no nd | |
| epidermal lipid synthes | KCS | Ketoacyl-CoA Synthase | AT1G0772 Manuscript (Kuczynski; C.; McCorkle; S.; Keereetaweep; J.; Shanklin; J.; & Schwender; J. (2022). An expanded role for the transcription factor WRINKLED1 in the biosynthesis of triacylglycerols during seed development. Frontiers in Plant Scienc | Sobic.004G34130 | yes | 52.09 | 51.42 | SP | 0.6878 SP 0.8570 | | | ortholo | no 3.7 | |
| epidermal lipid synthes | KCS | Ketoacyl-CoA Synthase | AT1G0772 Manuscript (Kuczynski; C.; McCorkle; S.; Keereetaweep; J.; Shanklin; J.; & Schwender; J. (2022). An expanded role for the transcription factor WRINKLED1 in the biosynthesis of triacylglycerols during seed development. Frontiers in Plant Scienc | Sobic.001G22190 | no | 51.8 | 50.44 | SP | 0.6878 SP 0.8118 | | | ortholo | no nd | |
| epidermal lipid synthes | KCS | Ketoacyl-CoA Synthase | AT1G0772 Manuscript (Kuczynski; C.; McCorkle; S.; Keereetaweep; J.; Shanklin; J.; & Schwender; J. (2022). An expanded role for the transcription factor WRINKLED1 in the biosynthesis of triacylglycerols during seed development. Frontiers in Plant Scienc | Sobic.001G35470 | no | 50.44 | 49.79 | SP | 0.6878 SP 0.8404 | | | ortholo | no 1551.1 | |
| epidermal lipid synthes | KCS | Ketoacyl-CoA Synthase | AT1G0772 Manuscript (Kuczynski; C.; McCorkle; S.; Keereetaweep; J.; Shanklin; J.; & Schwender; J. (2022). An expanded role for the transcription factor WRINKLED1 in the biosynthesis of triacylglycerols during seed development. Frontiers in Plant Scienc | Sobic.004G34130 | yes | 52.09 | 52.09 | SP | 0.6878 SP 0.8570 | | | ortholo | no 3.7 | |
| epidermal lipid synthes | KCS | Ketoacyl-CoA Synthase | AT1G0772 Manuscript (Kuczynski; C.; McCorkle; S.; Keereetaweep; J.; Shanklin; J.; & Schwender; J. (2022). An expanded role for the transcription factor WRINKLED1 in the biosynthesis of triacylglycerols during seed development. Frontiers in Plant Scienc | Sobic.001G22190 | no | 51.8 | 51.8 | SP | 0.6878 SP 0.8118 | | | ortholo | no nd | |
| epidermal lipid synthes | KCS | Ketoacyl-CoA Synthase | AT1G0772 Manuscript (Kuczynski; C.; McCorkle; S.; Keereetaweep; J.; Shanklin; J.; & Schwender; J. (2022). An expanded role for the transcription factor WRINKLED1 in the biosynthesis of triacylglycerols during seed development. Frontiers in Plant Scienc | Sobic.001G35470 | no | 50.44 | 50.44 | SP | 0.6878 SP 0.8404 | | | ortholo | no 1551.1 | |
| epidermal lipid synthes | KCS | Ketoacyl-CoA Synthase | AT1G1944 Manuscript (Kuczynski; C.; McCorkle; S.; Keereetaweep; J.; Shanklin; J.; & Schwender; J. (2022). An expanded role for the transcription factor WRINKLED1 in the biosynthesis of triacylglycerols during seed development. Frontiers in Plant Scienc | Sobic.009G23610 | bidirectiona | 80.94 | 77.5 | OTHER | 1.0000 OTHER 0.9997 | | | ortholo | no 5240.3 | |
| epidermal lipid synthes | KCS | Ketoacyl-CoA Synthase | AT1G1944 Manuscript (Kuczynski; C.; McCorkle; S.; Keereetaweep; J.; Shanklin; J.; & Schwender; J. (2022). An expanded role for the transcription factor WRINKLED1 in the biosynthesis of triacylglycerols during seed development. Frontiers in Plant Scienc | Sobic.010G18150 | no | 68.38 | 65.55 | OTHER | 1.0000 OTHER 0.9988 | | | no | no 110.4 | |
| epidermal lipid synthes | KCS | Ketoacyl-CoA Synthase | AT1G1944 Manuscript (Kuczynski; C.; McCorkle; S.; Keereetaweep; J.; Shanklin; J.; & Schwender; J. (2022). An expanded role for the transcription factor WRINKLED1 in the biosynthesis of triacylglycerols during seed development. Frontiers in Plant Scienc | Sobic.004G08680 | no | 68.3 | 65.05 | OTHER | 1.0000 OTHER 1.0000 | | | no | no 1524.2 | |
| epidermal lipid synthes | KCS | Ketoacyl-CoA Synthase | AT1G1944 Manuscript (Kuczynski; C.; McCorkle; S.; Keereetaweep; J.; Shanklin; J.; & Schwender; J. (2022). An expanded role for the transcription factor WRINKLED1 in the biosynthesis of triacylglycerols during seed development. Frontiers in Plant Scienc | Sobic.001G45320 | no | 67.98 | 66.53 | OTHER | 1.0000 OTHER 0.9580 | | | no | no 2162.6 | |
| epidermal lipid synthes | KCS | Ketoacyl-CoA Synthase | AT1G1944 Manuscript (Kuczynski; C.; McCorkle; S.; Keereetaweep; J.; Shanklin; J.; & Schwender; J. (2022). An expanded role for the transcription factor WRINKLED1 in the biosynthesis of triacylglycerols during seed development. Frontiers in Plant Scienc | Sobic.009G24100 | no | 67.82 | 65.87 | OTHER | 1.0000 OTHER 0.9999 | | | no | no 632.4 | |
| epidermal lipid synthes | KCS | Ketoacyl-CoA Synthase | AT1G1944 Manuscript (Kuczynski; C.; McCorkle; S.; Keereetaweep; J.; Shanklin; J.; & Schwender; J. (2022). An expanded role for the transcription factor WRINKLED1 in the biosynthesis of triacylglycerols during seed development. Frontiers in Plant Scienc | Sobic.009G23610 | bidirectiona | 80.94 | 80.94 | OTHER | 1.0000 OTHER 0.9997 | | | ortholo | no 5240.3 | |
| epidermal lipid synthes | KCS | Ketoacyl-CoA Synthase | AT1G1944 Manuscript (Kuczynski; C.; McCorkle; S.; Keereetaweep; J.; Shanklin; J.; & Schwender; J. (2022). An expanded role for the transcription factor WRINKLED1 in the biosynthesis of triacylglycerols during seed development. Frontiers in Plant Scienc | Sobic.010G18150 | no | 68.38 | 68.38 | OTHER | 1.0000 OTHER 0.9988 | | | no | no 110.4 | |
| epidermal lipid synthes | KCS | Ketoacyl-CoA Synthase | AT1G1944 Manuscript (Kuczynski; C.; McCorkle; S.; Keereetaweep; J.; Shanklin; J.; & Schwender; J. (2022). An expanded role for the transcription factor WRINKLED1 in the biosynthesis of triacylglycerols during seed development. Frontiers in Plant Scienc | Sobic.004G08680 | no | 68.3 | 68.3 | OTHER | 1.0000 OTHER 1.0000 | | | no | no 1524.2 | |
| epidermal lipid synthes | KCS | Ketoacyl-CoA Synthase | AT1G1944 Manuscript (Kuczynski; C.; McCorkle; S.; Keereetaweep; J.; Shanklin; J.; & Schwender; J. (2022). An expanded role for the transcription factor WRINKLED1 in the biosynthesis of triacylglycerols during seed development. Frontiers in Plant Scienc | Sobic.001G45320 | no | 67.98 | 67.98 | OTHER | 1.0000 OTHER 0.9580 | | | no | no 2162.6 | |
| epidermal lipid synthes | KCS | Ketoacyl-CoA Synthase | AT1G1944 Manuscript (Kuczynski; C.; McCorkle; S.; Keereetaweep; J.; Shanklin; J.; & Schwender; J. (2022). An expanded role for the transcription factor WRINKLED1 in the biosynthesis of triacylglycerols during seed development. Frontiers in Plant Scienc | Sobic.009G24100 | no | 67.82 | 67.82 | OTHER | 1.0000 OTHER 0.9999 | | | no | no 632.4 | |
| epidermal lipid synthes | KCR | Ketoacyl-CoA Reductase | AT1G2447 Manuscript (Kuczynski; C.; McCorkle; S.; Keereetaweep; J.; Shanklin; J.; & Schwender; J. (2022). An expanded role for the transcription factor WRINKLED1 in the biosynthesis of triacylglycerols during seed development. Frontiers in Plant Scienc | Sobic.006G12580 | no | 49.24 | 45.7 | OTHER | 0.9616 SP 0.5206 | | | ortholo | no 574.1 | |
| epidermal lipid synthes | KCR | Ketoacyl-CoA Reductase | AT1G2447 Manuscript (Kuczynski; C.; McCorkle; S.; Keereetaweep; J.; Shanklin; J.; & Schwender; J. (2022). An expanded role for the transcription factor WRINKLED1 in the biosynthesis of triacylglycerols during seed development. Frontiers in Plant Scienc | Sobic.004G20390 | yes | 47.99 | 49.18 | OTHER | 0.9616 OTHER 0.8462 | | | ortholo | no 1736.8 | |
| epidermal lipid synthes | KCR | Ketoacyl-CoA Reductase | AT1G2447 Manuscript (Kuczynski; C.; McCorkle; S.; Keereetaweep; J.; Shanklin; J.; & Schwender; J. (2022). An expanded role for the transcription factor WRINKLED1 in the biosynthesis of triacylglycerols during seed development. Frontiers in Plant Scienc | Sobic.001G37000 | no | 46.96 | 47.39 | OTHER | 0.9616 OTHER 0.9996 | | | ortholo | no nd | |
| epidermal lipid synthes | KCR | Ketoacyl-CoA Reductase | AT1G2447 Manuscript (Kuczynski; C.; McCorkle; S.; Keereetaweep; J.; Shanklin; J.; & Schwender; J. (2022). An expanded role for the transcription factor WRINKLED1 in the biosynthesis of triacylglycerols during seed development. Frontiers in Plant Scienc | Sobic.010G12450 | no | 44.77 | 45 | OTHER | 0.9616 SP 0.5028 | | | ortholo | no nd | |
| epidermal lipid synthes | KCS | Ketoacyl-CoA Synthase | AT1G2545 Manuscript (Kuczynski; C.; McCorkle; S.; Keereetaweep; J.; Shanklin; J.; & Schwender; J. (2022). An expanded role for the transcription factor WRINKLED1 in the biosynthesis of triacylglycerols during seed development. Frontiers in Plant Scienc | Sobic.001G45320 | yes | 76.07 | 75.76 | OTHER | 0.9952 OTHER 0.9580 | | | ortholo | no 2162.6 | |
| epidermal lipid synthes | KCS | Ketoacyl-CoA Synthase | AT1G2545 Manuscript (Kuczynski; C.; McCorkle; S.; Keereetaweep; J.; Shanklin; J.; & Schwender; J. (2022). An expanded role for the transcription factor WRINKLED1 in the biosynthesis of triacylglycerols during seed development. Frontiers in Plant Scienc | Sobic.009G23610 | no | 62.19 | 61.3 | OTHER | 0.9952 OTHER 0.9997 | | | no | no 5240.3 | |
| epidermal lipid synthes | KCS | Ketoacyl-CoA Synthase | AT1G2545 Manuscript (Kuczynski; C.; McCorkle; S.; Keereetaweep; J.; Shanklin; J.; & Schwender; J. (2022). An expanded role for the transcription factor WRINKLED1 in the biosynthesis of triacylglycerols during seed development. Frontiers in Plant Scienc | Sobic.004G24940 | yes | 60.37 | 61.09 | OTHER | 0.9952 OTHER 0.9561 | | | ortholo | no 1669.3 | |

epidermal lipid synthes KCS Ketoacyl-CoA Synthase AT1G2545 Manuscript (Kuczynski; C.; McCorkle; S.; Keereetaweep; J.; Shanklin; J.; & Schwender; J. (2022). An expanded role for the transcription factor WRINKLED1 in the biosynthesis of triacylglycerols during seed development. Frontiers in Plant Scienc Sobic.010G18150 no 60.24 60.37 OTHER 0.9952 OTHER 0.9988 no no 110.4

epidermal lipid synthes KCS Ketoacyl-CoA Synthase AT1G2545 Manuscript (Kuczynski; C.; McCorkle; S.; Keereetaweep; J.; Shanklin; J.; & Schwender; J. (2022). An expanded role for the transcription factor WRINKLED1 in the biosynthesis of triacylglycerols during seed development. Frontiers in Plant Scienc Sobic.004G08680 no 59.92 60.37 OTHER 0.9952 OTHER 1.0000 no no 1524.2

epidermal lipid synthes KCR Ketoacyl-CoA Reductase AT1G6773 Manuscript (Kuczynski; C.; McCorkle; S.; Keereetaweep; J.; Shanklin; J.; & Schwender; J. (2022). An expanded role for the transcription factor WRINKLED1 in the biosynthesis of triacylglycerols during seed development. Frontiers in Plant Scienc Sobic.004G20390 bidirectiona 56.33 56.15 OTHER 0.8500 OTHER 0.8462 LDO no 1736.8

epidermal lipid synthes KCR Ketoacyl-CoA Reductase AT1G6773 Manuscript (Kuczynski; C.; McCorkle; S.; Keereetaweep; J.; Shanklin; J.; & Schwender; J. (2022). An expanded role for the transcription factor WRINKLED1 in the biosynthesis of triacylglycerols during seed development. Frontiers in Plant Scienc Sobic.006G12580 yes 55.39 51.91 OTHER 0.8500 SP 0.5206 ortholo no 574.1

epidermal lipid synthes KCR Ketoacyl-CoA Reductase AT1G6773 Manuscript (Kuczynski; C.; McCorkle; S.; Keereetaweep; J.; Shanklin; J.; & Schwender; J. (2022). An expanded role for the transcription factor WRINKLED1 in the biosynthesis of triacylglycerols during seed development. Frontiers in Plant Scienc Sobic.006G12560 yes 52.42 48.74 OTHER 0.8500 OTHER 0.4547 ortholo no nd epidermal lipid synthes KCS Ketoacyl-CoA Synthase AT1G6853 Manuscript (Kuczynski; C.; McCorkle; S.; Keereetaweep; J.; Shanklin; J.; & Schwender; J. (2022). An expanded role for the transcription factor WRINKLED1 in the biosynthesis of triacylglycerols during seed development. Frontiers in Plant Scienc Sobic.001G45320 bidirectiona 77.91 77.96 OTHER 0.9981 OTHER 0.9580 LDO no 2162.6

epidermal lipid synthes KCS Ketoacyl-CoA Synthase AT1G6853 Manuscript (Kuczynski; C.; McCorkle; S.; Keereetaweep; J.; Shanklin; J.; & Schwender; J. (2022). An expanded role for the transcription factor WRINKLED1 in the biosynthesis of triacylglycerols during seed development. Frontiers in Plant Scienc Sobic.009G23610 no 64.34 64.34 OTHER 0.9981 OTHER 0.9997 no no 5240.3

epidermal lipid synthes KCS Ketoacyl-CoA Synthase AT1G6853 Manuscript (Kuczynski; C.; McCorkle; S.; Keereetaweep; J.; Shanklin; J.; & Schwender; J. (2022). An expanded role for the transcription factor WRINKLED1 in the biosynthesis of triacylglycerols during seed development. Frontiers in Plant Scienc Sobic.010G18150 no 61.87 61.87 OTHER 0.9981 OTHER 0.9988 no no 110.4

epidermal lipid synthes KCS Ketoacyl-CoA Synthase AT1G6853 Manuscript (Kuczynski; C.; McCorkle; S.; Keereetaweep; J.; Shanklin; J.; & Schwender; J. (2022). An expanded role for the transcription factor WRINKLED1 in the biosynthesis of triacylglycerols during seed development. Frontiers in Plant Scienc Sobic.004G08680 no 61.45 61.45 OTHER 0.9981 OTHER 1.0000 no no 1524.2

epidermal lipid synthes KCS Ketoacyl-CoA Synthase AT1G7116 Manuscript (Kuczynski; C.; McCorkle; S.; Keereetaweep; J.; Shanklin; J.; & Schwender; J. (2022). An expanded role for the transcription factor WRINKLED1 in the biosynthesis of triacylglycerols during seed development. Frontiers in Plant Scienc Sobic.001G45320 yes 51.95 51.95 OTHER 0.9936 OTHER 0.9580 no no 2162.6

epidermal lipid synthes KCS Ketoacyl-CoA Synthase AT1G7116 Manuscript (Kuczynski; C.; McCorkle; S.; Keereetaweep; J.; Shanklin; J.; & Schwender; J. (2022). An expanded role for the transcription factor WRINKLED1 in the biosynthesis of triacylglycerols during seed development. Frontiers in Plant Scienc Sobic.001G43810 no 48.89 48.89 OTHER 0.9936 OTHER 1.0000 no no 2121.4

epidermal lipid synthes KCS Ketoacyl-CoA Synthase AT1G7116 Manuscript (Kuczynski; C.; McCorkle; S.; Keereetaweep; J.; Shanklin; J.; & Schwender; J. (2022). An expanded role for the transcription factor WRINKLED1 in the biosynthesis of triacylglycerols during seed development. Frontiers in Plant Scienc Sobic.009G23610 no 48.7 48.7 OTHER 0.9936 OTHER 0.9997 no no 5240.3

epidermal lipid synthes KCS Ketoacyl-CoA Synthase AT1G7116 Manuscript (Kuczynski; C.; McCorkle; S.; Keereetaweep; J.; Shanklin; J.; & Schwender; J. (2022). An expanded role for the transcription factor WRINKLED1 in the biosynthesis of triacylglycerols during seed development. Frontiers in Plant Scienc Sobic.009G24100 no 48.46 48.46 OTHER 0.9936 OTHER 0.9999 no no 632.4

epidermal lipid synthes KCS Ketoacyl-CoA Synthase AT1G7116 Manuscript (Kuczynski; C.; McCorkle; S.; Keereetaweep; J.; Shanklin; J.; & Schwender; J. (2022). An expanded role for the transcription factor WRINKLED1 in the biosynthesis of triacylglycerols during seed development. Frontiers in Plant Scienc Sobic.010G10740 no 48.43 48.43 OTHER 0.9936 OTHER 0.9522 no no 38.4

epidermal lipid synthes KCS Ketoacyl-CoA Synthase AT1G7116 Manuscript (Kuczynski; C.; McCorkle; S.; Keereetaweep; J.; Shanklin; J.; & Schwender; J. (2022). An expanded role for the transcription factor WRINKLED1 in the biosynthesis of triacylglycerols during seed development. Frontiers in Plant Scienc Sobic.010G18150 no 48.25 48.25 OTHER 0.9936 OTHER 0.9988 no no 110.4

epidermal lipid synthes KCS Ketoacyl-CoA Synthase AT1G7116 Manuscript (Kuczynski; C.; McCorkle; S.; Keereetaweep; J.; Shanklin; J.; & Schwender; J. (2022). An expanded role for the transcription factor WRINKLED1 in the biosynthesis of triacylglycerols during seed development. Frontiers in Plant Scienc Sobic.005G16900 no 48.15 48.15 OTHER 0.9936 OTHER 0.9810 no no nd epidermal lipid synthes KCS Ketoacyl-CoA Synthase AT1G7116 Manuscript (Kuczynski; C.; McCorkle; S.; Keereetaweep; J.; Shanklin; J.; & Schwender; J. (2022). An expanded role for the transcription factor WRINKLED1 in the biosynthesis of triacylglycerols during seed development. Frontiers in Plant Scienc Sobic.004G08680 no 47.88 47.88 OTHER 0.9936 OTHER 1.0000 no no 1524.2

epidermal lipid synthes KCS Ketoacyl-CoA Synthase AT2G1509 Manuscript (Kuczynski; C.; McCorkle; S.; Keereetaweep; J.; Shanklin; J.; & Schwender; J. (2022). An expanded role for the transcription factor WRINKLED1 in the biosynthesis of triacylglycerols during seed development. Frontiers in Plant Scienc Sobic.009G23610 yes 62.99 62.99 SP 0.7377 OTHER 0.9997 ortholo no 5240.3

epidermal lipid synthes KCS Ketoacyl-CoA Synthase AT2G1509 Manuscript (Kuczynski; C.; McCorkle; S.; Keereetaweep; J.; Shanklin; J.; & Schwender; J. (2022). An expanded role for the transcription factor WRINKLED1 in the biosynthesis of triacylglycerols during seed development. Frontiers in Plant Scienc Sobic.009G24100 no 60.85 60.85 SP 0.7377 OTHER 0.9999 no no 632.4

epidermal lipid synthes KCS Ketoacyl-CoA Synthase AT2G1509 Manuscript (Kuczynski; C.; McCorkle; S.; Keereetaweep; J.; Shanklin; J.; & Schwender; J. (2022). An expanded role for the transcription factor WRINKLED1 in the biosynthesis of triacylglycerols during seed development. Frontiers in Plant Scienc Sobic.005G16900 no 58.93 58.93 SP 0.7377 OTHER 0.9810 no no nd epidermal lipid synthes KCS Ketoacyl-CoA Synthase AT2G1509 Manuscript (Kuczynski; C.; McCorkle; S.; Keereetaweep; J.; Shanklin; J.; & Schwender; J. (2022). An expanded role for the transcription factor WRINKLED1 in the biosynthesis of triacylglycerols during seed development. Frontiers in Plant Scienc Sobic.010G18150 no 57.87 57.87 SP 0.7377 OTHER 0.9988 no no 110.4

epidermal lipid synthes KCS Ketoacyl-CoA Synthase AT2G1509 Manuscript (Kuczynski; C.; McCorkle; S.; Keereetaweep; J.; Shanklin; J.; & Schwender; J. (2022). An expanded role for the transcription factor WRINKLED1 in the biosynthesis of triacylglycerols during seed development. Frontiers in Plant Scienc Sobic.004G08680 no 57.54 57.54 SP 0.7377 OTHER 1.0000 no no 1524.2

epidermal lipid synthes KCS Ketoacyl-CoA Synthase AT2G1509 Manuscript (Kuczynski; C.; McCorkle; S.; Keereetaweep; J.; Shanklin; J.; & Schwender; J. (2022). An expanded role for the transcription factor WRINKLED1 in the biosynthesis of triacylglycerols during seed development. Frontiers in Plant Scienc Sobic.001G24770 no 56.35 56.35 SP 0.7377 OTHER 0.8671 no no nd epidermal lipid synthes KCS Ketoacyl-CoA Synthase AT2G1509 Manuscript (Kuczynski; C.; McCorkle; S.; Keereetaweep; J.; Shanklin; J.; & Schwender; J. (2022). An expanded role for the transcription factor WRINKLED1 in the biosynthesis of triacylglycerols during seed development. Frontiers in Plant Scienc Sobic.001G45320 no 55.81 55.81 SP 0.7377 OTHER 0.9580 no no 2162.6

epidermal lipid synthes KCS Ketoacyl-CoA Synthase AT2G1509 Manuscript (Kuczynski; C.; McCorkle; S.; Keereetaweep; J.; Shanklin; J.; & Schwender; J. (2022). An expanded role for the transcription factor WRINKLED1 in the biosynthesis of triacylglycerols during seed development. Frontiers in Plant Scienc Sobic.001G49550 no 55.58 55.58 SP 0.7377 OTHER 0.9290 no no nd epidermal lipid synthes KCS Ketoacyl-CoA Synthase AT2G1509 Manuscript (Kuczynski; C.; McCorkle; S.; Keereetaweep; J.; Shanklin; J.; & Schwender; J. (2022). An expanded role for the transcription factor WRINKLED1 in the biosynthesis of triacylglycerols during seed development. Frontiers in Plant Scienc Sobic.005G16870 no 55.41 55.41 SP 0.7377 OTHER 0.9985 no no 3001.9

epidermal lipid synthes KCS Ketoacyl-CoA Synthase AT2G1628 Manuscript (Kuczynski; C.; McCorkle; S.; Keereetaweep; J.; Shanklin; J.; & Schwender; J. (2022). An expanded role for the transcription factor WRINKLED1 in the biosynthesis of triacylglycerols during seed development. Frontiers in Plant Scienc Sobic.009G23610 yes 77.87 77.87 OTHER 1.0000 OTHER 0.9997 ortholo no 5240.3

epidermal lipid synthes KCS Ketoacyl-CoA Synthase AT2G1628 Manuscript (Kuczynski; C.; McCorkle; S.; Keereetaweep; J.; Shanklin; J.; & Schwender; J. (2022). An expanded role for the transcription factor WRINKLED1 in the biosynthesis of triacylglycerols during seed development. Frontiers in Plant Scienc Sobic.010G18150 no 65.92 65.92 OTHER 1.0000 OTHER 0.9988 no no 110.4

epidermal lipid synthes KCS Ketoacyl-CoA Synthase AT2G1628 Manuscript (Kuczynski; C.; McCorkle; S.; Keereetaweep; J.; Shanklin; J.; & Schwender; J. (2022). An expanded role for the transcription factor WRINKLED1 in the biosynthesis of triacylglycerols during seed development. Frontiers in Plant Scienc Sobic.001G45320 no 65.22 65.22 OTHER 1.0000 OTHER 0.9580 no no 2162.6

epidermal lipid synthes KCS Ketoacyl-CoA Synthase AT2G1628 Manuscript (Kuczynski; C.; McCorkle; S.; Keereetaweep; J.; Shanklin; J.; & Schwender; J. (2022). An expanded role for the transcription factor WRINKLED1 in the biosynthesis of triacylglycerols during seed development. Frontiers in Plant Scienc Sobic.004G08680 no 63.67 63.67 OTHER 1.0000 OTHER 1.0000 no no 1524.2

epidermal lipid synthes KCS Ketoacyl-CoA Synthase AT2G1628 Manuscript (Kuczynski; C.; McCorkle; S.; Keereetaweep; J.; Shanklin; J.; & Schwender; J. (2022). An expanded role for the transcription factor WRINKLED1 in the biosynthesis of triacylglycerols during seed development. Frontiers in Plant Scienc Sobic.009G24100 no 63.41 63.41 OTHER 1.0000 OTHER 0.9999 no no 632.4

epidermal lipid synthes KCS Ketoacyl-CoA Synthase AT2G1628 Manuscript (Kuczynski; C.; McCorkle; S.; Keereetaweep; J.; Shanklin; J.; & Schwender; J. (2022). An expanded role for the transcription factor WRINKLED1 in the biosynthesis of triacylglycerols during seed development. Frontiers in Plant Scienc Sobic.005G16900 no 60.71 60.71 OTHER 1.0000 OTHER 0.9810 no no nd epidermal lipid synthes KCS Ketoacyl-CoA Synthase AT2G2625 Manuscript (Kuczynski; C.; McCorkle; S.; Keereetaweep; J.; Shanklin; J.; & Schwender; J. (2022). An expanded role for the transcription factor WRINKLED1 in the biosynthesis of triacylglycerols during seed development. Frontiers in Plant Scienc Sobic.001G48230 bidirectiona 69.41 69.41 OTHER 1.0000 OTHER 0.9997 LDO no 2532.9

epidermal lipid synthes KCS Ketoacyl-CoA Synthase AT2G2625 Manuscript (Kuczynski; C.; McCorkle; S.; Keereetaweep; J.; Shanklin; J.; & Schwender; J. (2022). An expanded role for the transcription factor WRINKLED1 in the biosynthesis of triacylglycerols during seed development. Frontiers in Plant Scienc Sobic.009G23610 no 59.19 59.19 OTHER 1.0000 OTHER 0.9997 no no 5240.3

epidermal lipid synthes KCS Ketoacyl-CoA Synthase AT2G2625 Manuscript (Kuczynski; C.; McCorkle; S.; Keereetaweep; J.; Shanklin; J.; & Schwender; J. (2022). An expanded role for the transcription factor WRINKLED1 in the biosynthesis of triacylglycerols during seed development. Frontiers in Plant Scienc Sobic.004G08680 no 55.7 55.7 OTHER 1.0000 OTHER 1.0000 no no 1524.2

epidermal lipid synthes KCS Ketoacyl-CoA Synthase AT2G2625 Manuscript (Kuczynski; C.; McCorkle; S.; Keereetaweep; J.; Shanklin; J.; & Schwender; J. (2022). An expanded role for the transcription factor WRINKLED1 in the biosynthesis of triacylglycerols during seed development. Frontiers in Plant Scienc Sobic.010G18150 no 55.23 55.23 OTHER 1.0000 OTHER 0.9988 no no 110.4

epidermal lipid synthes KCS Ketoacyl-CoA Synthase AT2G2625 Manuscript (Kuczynski; C.; McCorkle; S.; Keereetaweep; J.; Shanklin; J.; & Schwender; J. (2022). An expanded role for the transcription factor WRINKLED1 in the biosynthesis of triacylglycerols during seed development. Frontiers in Plant Scienc Sobic.001G45320 no 55.19 55.19 OTHER 1.0000 OTHER 0.9580 no no 2162.6

epidermal lipid synthes KCS Ketoacyl-CoA Synthase AT2G2664 Manuscript (Kuczynski; C.; McCorkle; S.; Keereetaweep; J.; Shanklin; J.; & Schwender; J. (2022). An expanded role for the transcription factor WRINKLED1 in the biosynthesis of triacylglycerols during seed development. Frontiers in Plant Scienc Sobic.010G18150 bidirectiona 80.2 80.2 OTHER 1.0000 OTHER 0.9988 ortholo no 110.4

epidermal lipid synthes KCS Ketoacyl-CoA Synthase AT2G2664 Manuscript (Kuczynski; C.; McCorkle; S.; Keereetaweep; J.; Shanklin; J.; & Schwender; J. (2022). An expanded role for the transcription factor WRINKLED1 in the biosynthesis of triacylglycerols during seed development. Frontiers in Plant Scienc Sobic.004G08680 yes 75.95 76.55 OTHER 1.0000 OTHER 1.0000 ortholo no 1524.2

epidermal lipid synthes KCS Ketoacyl-CoA Synthase AT2G2664 Manuscript (Kuczynski; C.; McCorkle; S.; Keereetaweep; J.; Shanklin; J.; & Schwender; J. (2022). An expanded role for the transcription factor WRINKLED1 in the biosynthesis of triacylglycerols during seed development. Frontiers in Plant Scienc Sobic.009G24100 yes 73.88 73.93 OTHER 1.0000 OTHER 0.9999 no no 632.4

epidermal lipid synthes KCS Ketoacyl-CoA Synthase AT2G2664 Manuscript (Kuczynski; C.; McCorkle; S.; Keereetaweep; J.; Shanklin; J.; & Schwender; J. (2022). An expanded role for the transcription factor WRINKLED1 in the biosynthesis of triacylglycerols during seed development. Frontiers in Plant Scienc Sobic.009G23610 no 67.28 67.28 OTHER 1.0000 OTHER 0.9997 no no 5240.3

epidermal lipid synthes KCS Ketoacyl-CoA Synthase AT2G2664 Manuscript (Kuczynski; C.; McCorkle; S.; Keereetaweep; J.; Shanklin; J.; & Schwender; J. (2022). An expanded role for the transcription factor WRINKLED1 in the biosynthesis of triacylglycerols during seed development. Frontiers in Plant Scienc Sobic.005G16870 no 64.95 64.95 OTHER 1.0000 OTHER 0.9985 no no 3001.9

epidermal lipid synthes KCS Ketoacyl-CoA Synthase AT2G2664 Manuscript (Kuczynski; C.; McCorkle; S.; Keereetaweep; J.; Shanklin; J.; & Schwender; J. (2022). An expanded role for the transcription factor WRINKLED1 in the biosynthesis of triacylglycerols during seed development. Frontiers in Plant Scienc Sobic.005G16900 no 60.71 60.71 OTHER 1.0000 OTHER 0.9810 no no nd epidermal lipid synthes KCS Ketoacyl-CoA Synthase AT2G2664 Manuscript (Kuczynski; C.; McCorkle; S.; Keereetaweep; J.; Shanklin; J.; & Schwender; J. (2022). An expanded role for the transcription factor WRINKLED1 in the biosynthesis of triacylglycerols during seed development. Frontiers in Plant Scienc Sobic.001G45320 no 60.16 60.16 OTHER 1.0000 OTHER 0.9580 no no 2162.6

epidermal lipid synthes KCS Ketoacyl-CoA Synthase AT2G2664 Manuscript (Kuczynski; C.; McCorkle; S.; Keereetaweep; J.; Shanklin; J.; & Schwender; J. (2022). An expanded role for the transcription factor WRINKLED1 in the biosynthesis of triacylglycerols during seed development. Frontiers in Plant Scienc Sobic.001G24770 yes 59.76 59.57 OTHER 1.0000 OTHER 0.8671 no no nd epidermal lipid synthes KCS Ketoacyl-CoA Synthase AT2G2863 Manuscript (Kuczynski; C.; McCorkle; S.; Keereetaweep; J.; Shanklin; J.; & Schwender; J. (2022). An expanded role for the transcription factor WRINKLED1 in the biosynthesis of triacylglycerols during seed development. Frontiers in Plant Scienc Sobic.001G22190 yes 53.15 52.78 SP 0.9217 SP 0.8118 LDO no nd epidermal lipid synthes KCS Ketoacyl-CoA Synthase AT2G2863 Manuscript (Kuczynski; C.; McCorkle; S.; Keereetaweep; J.; Shanklin; J.; & Schwender; J. (2022). An expanded role for the transcription factor WRINKLED1 in the biosynthesis of triacylglycerols during seed development. Frontiers in Plant Scienc Sobic.001G35470 bidirectiona 52.28 52.28 SP 0.9217 SP 0.8404 ortholo no 1551.1

epidermal lipid synthes KCS Ketoacyl-CoA Synthase AT2G2863 Manuscript (Kuczynski; C.; McCorkle; S.; Keereetaweep; J.; Shanklin; J.; & Schwender; J. (2022). An expanded role for the transcription factor WRINKLED1 in the biosynthesis of triacylglycerols during seed development. Frontiers in Plant Scienc Sobic.004G34130 yes 50.56 50.9 SP 0.9217 SP 0.8570 ortholo no 3.7

epidermal lipid synthes KCS Ketoacyl-CoA Synthase AT2G4672 Manuscript (Kuczynski; C.; McCorkle; S.; Keereetaweep; J.; Shanklin; J.; & Schwender; J. (2022). An expanded role for the transcription factor WRINKLED1 in the biosynthesis of triacylglycerols during seed development. Frontiers in Plant Scienc Sobic.009G24100 yes 61.3 61.3 SP 0.5501 OTHER 0.9999 no no 632.4

epidermal lipid synthes KCS Ketoacyl-CoA Synthase AT2G4672 Manuscript (Kuczynski; C.; McCorkle; S.; Keereetaweep; J.; Shanklin; J.; & Schwender; J. (2022). An expanded role for the transcription factor WRINKLED1 in the biosynthesis of triacylglycerols during seed development. Frontiers in Plant Scienc Sobic.001G26450 no 59.67 59.67 SP 0.5501 OTHER 0.9999 ortholo no 21.5

epidermal lipid synthes KCS Ketoacyl-CoA Synthase AT2G4672 Manuscript (Kuczynski; C.; McCorkle; S.; Keereetaweep; J.; Shanklin; J.; & Schwender; J. (2022). An expanded role for the transcription factor WRINKLED1 in the biosynthesis of triacylglycerols during seed development. Frontiers in Plant Scienc Sobic.004G08680 no 59.38 59.38 SP 0.5501 OTHER 1.0000 no no 1524.2

epidermal lipid synthes KCS Ketoacyl-CoA Synthase AT2G4672 Manuscript (Kuczynski; C.; McCorkle; S.; Keereetaweep; J.; Shanklin; J.; & Schwender; J. (2022). An expanded role for the transcription factor WRINKLED1 in the biosynthesis of triacylglycerols during seed development. Frontiers in Plant Scienc Sobic.010G18150 no 58.55 58.55 SP 0.5501 OTHER 0.9988 no no 110.4

epidermal lipid synthes KCS Ketoacyl-CoA Synthase AT2G4672 Manuscript (Kuczynski; C.; McCorkle; S.; Keereetaweep; J.; Shanklin; J.; & Schwender; J. (2022). An expanded role for the transcription factor WRINKLED1 in the biosynthesis of triacylglycerols during seed development. Frontiers in Plant Scienc Sobic.005G16870 no 57.97 57.97 SP 0.5501 OTHER 0.9985 no no 3001.9

epidermal lipid synthes KCS Ketoacyl-CoA Synthase AT2G4672 Manuscript (Kuczynski; C.; McCorkle; S.; Keereetaweep; J.; Shanklin; J.; & Schwender; J. (2022). An expanded role for the transcription factor WRINKLED1 in the biosynthesis of triacylglycerols during seed development. Frontiers in Plant Scienc Sobic.009G23610 no 57.35 57.35 SP 0.5501 OTHER 0.9997 no no 5240.3

epidermal lipid synthes KCS Ketoacyl-CoA Synthase AT2G4672 Manuscript (Kuczynski; C.; McCorkle; S.; Keereetaweep; J.; Shanklin; J.; & Schwender; J. (2022). An expanded role for the transcription factor WRINKLED1 in the biosynthesis of triacylglycerols during seed development. Frontiers in Plant Scienc Sobic.001G49550 no 56.55 56.55 SP 0.5501 OTHER 0.9290 no no nd epidermal lipid synthes KCS Ketoacyl-CoA Synthase AT2G4672 Manuscript (Kuczynski; C.; McCorkle; S.; Keereetaweep; J.; Shanklin; J.; & Schwender; J. (2022). An expanded role for the transcription factor WRINKLED1 in the biosynthesis of triacylglycerols during seed development. Frontiers in Plant Scienc Sobic.005G19360 no 55.04 55.04 SP 0.5501 OTHER 0.9990 no no 395.6

epidermal lipid synthes KCS Ketoacyl-CoA Synthase AT3G1028 Manuscript (Kuczynski; C.; McCorkle; S.; Keereetaweep; J.; Shanklin; J.; & Schwender; J. (2022). An expanded role for the transcription factor WRINKLED1 in the biosynthesis of triacylglycerols during seed development. Frontiers in Plant Scienc Sobic.009G24100 yes 59.86 59.86 SP 0.3792 OTHER 0.9999 no no 632.4

epidermal lipid synthes KCS Ketoacyl-CoA Synthase AT3G1028 Manuscript (Kuczynski; C.; McCorkle; S.; Keereetaweep; J.; Shanklin; J.; & Schwender; J. (2022). An expanded role for the transcription factor WRINKLED1 in the biosynthesis of triacylglycerols during seed development. Frontiers in Plant Scienc Sobic.001G26450 no 58.23 58.23 SP 0.3792 OTHER 0.9999 ortholo no 21.5

epidermal lipid synthes KCS Ketoacyl-CoA Synthase AT3G1028 Manuscript (Kuczynski; C.; McCorkle; S.; Keereetaweep; J.; Shanklin; J.; & Schwender; J. (2022). An expanded role for the transcription factor WRINKLED1 in the biosynthesis of triacylglycerols during seed development. Frontiers in Plant Scienc Sobic.004G08680 no 57.69 57.69 SP 0.3792 OTHER 1.0000 no no 1524.2

epidermal lipid synthes KCS Ketoacyl-CoA Synthase AT3G1028 Manuscript (Kuczynski; C.; McCorkle; S.; Keereetaweep; J.; Shanklin; J.; & Schwender; J. (2022). An expanded role for the transcription factor WRINKLED1 in the biosynthesis of triacylglycerols during seed development. Frontiers in Plant Scienc Sobic.010G18150 no 56.87 56.87 SP 0.3792 OTHER 0.9988 no no 110.4

epidermal lipid synthes KCS Ketoacyl-CoA Synthase AT3G1028 Manuscript (Kuczynski; C.; McCorkle; S.; Keereetaweep; J.; Shanklin; J.; & Schwender; J. (2022). An expanded role for the transcription factor WRINKLED1 in the biosynthesis of triacylglycerols during seed development. Frontiers in Plant Scienc Sobic.005G16870 no 56.58 56.58 SP 0.3792 OTHER 0.9985 no no 3001.9

epidermal lipid synthes KCS Ketoacyl-CoA Synthase AT3G1028 Manuscript (Kuczynski; C.; McCorkle; S.; Keereetaweep; J.; Shanklin; J.; & Schwender; J. (2022). An expanded role for the transcription factor WRINKLED1 in the biosynthesis of triacylglycerols during seed development. Frontiers in Plant Scienc Sobic.009G23610 no 56.14 56.14 SP 0.3792 OTHER 0.9997 no no 5240.3

epidermal lipid synthes KCS Ketoacyl-CoA Synthase AT3G1028 Manuscript (Kuczynski; C.; McCorkle; S.; Keereetaweep; J.; Shanklin; J.; & Schwender; J. (2022). An expanded role for the transcription factor WRINKLED1 in the biosynthesis of triacylglycerols during seed development. Frontiers in Plant Scienc Sobic.001G49550 no 55.1 55.1 SP 0.3792 OTHER 0.9290 no no nd epidermal lipid synthes KCS Ketoacyl-CoA Synthase AT3G5216 Manuscript (Kuczynski; C.; McCorkle; S.; Keereetaweep; J.; Shanklin; J.; & Schwender; J. (2022). An expanded role for the transcription factor WRINKLED1 in the biosynthesis of triacylglycerols during seed development. Frontiers in Plant Scienc Sobic.005G16900 no 53.12 53.12 OTHER 1.0000 OTHER 0.9810 no no nd epidermal lipid synthes KCS Ketoacyl-CoA Synthase AT3G5216 Manuscript (Kuczynski; C.; McCorkle; S.; Keereetaweep; J.; Shanklin; J.; & Schwender; J. (2022). An expanded role for the transcription factor WRINKLED1 in the biosynthesis of triacylglycerols during seed development. Frontiers in Plant Scienc Sobic.004G08680 yes 45.98 45.98 OTHER 1.0000 OTHER 1.0000 no no 1524.2

epidermal lipid synthes KCS Ketoacyl-CoA Synthase AT3G5216 Manuscript (Kuczynski; C.; McCorkle; S.; Keereetaweep; J.; Shanklin; J.; & Schwender; J. (2022). An expanded role for the transcription factor WRINKLED1 in the biosynthesis of triacylglycerols during seed development. Frontiers in Plant Scienc Sobic.001G26450 no 45.28 45.28 OTHER 1.0000 OTHER 0.9999 no no 21.5

epidermal lipid synthes KCS Ketoacyl-CoA Synthase AT3G5216 Manuscript (Kuczynski; C.; McCorkle; S.; Keereetaweep; J.; Shanklin; J.; & Schwender; J. (2022). An expanded role for the transcription factor WRINKLED1 in the biosynthesis of triacylglycerols during seed development. Frontiers in Plant Scienc Sobic.005G16870 no 45.26 45.26 OTHER 1.0000 OTHER 0.9985 no no 3001.9

epidermal lipid synthes KCS Ketoacyl-CoA Synthase AT3G5216 Manuscript (Kuczynski; C.; McCorkle; S.; Keereetaweep; J.; Shanklin; J.; & Schwender; J. (2022). An expanded role for the transcription factor WRINKLED1 in the biosynthesis of triacylglycerols during seed development. Frontiers in Plant Scienc Sobic.010G18150 no 45.17 45.17 OTHER 1.0000 OTHER 0.9988 no no 110.4

epidermal lipid synthes ECR Enoyl-CoA Reductase AT3G5536 Manuscript (Kuczynski; C.; McCorkle; S.; Keereetaweep; J.; Shanklin; J.; & Schwender; J. (2022). An expanded role for the transcription factor WRINKLED1 in the biosynthesis of triacylglycerols during seed development. Frontiers in Plant Scienc Sobic.003G07160 bidirectiona 81.94 81.94 OTHER 0.9929 OTHER 0.9995 ortholo no 9819.8

epidermal lipid synthes KCS Ketoacyl-CoA Synthase AT4G3425 Manuscript (Kuczynski; C.; McCorkle; S.; Keereetaweep; J.; Shanklin; J.; & Schwender; J. (2022). An expanded role for the transcription factor WRINKLED1 in the biosynthesis of triacylglycerols during seed development. Frontiers in Plant Scienc Sobic.005G16900 no 64.29 64.29 OTHER 0.9225 OTHER 0.9810 no no nd epidermal lipid synthes KCS Ketoacyl-CoA Synthase AT4G3425 Manuscript (Kuczynski; C.; McCorkle; S.; Keereetaweep; J.; Shanklin; J.; & Schwender; J. (2022). An expanded role for the transcription factor WRINKLED1 in the biosynthesis of triacylglycerols during seed development. Frontiers in Plant Scienc Sobic.009G23610 yes 61.49 61.49 OTHER 0.9225 OTHER 0.9997 ortholo no 5240.3

epidermal lipid synthes KCS Ketoacyl-CoA Synthase AT4G3425 Manuscript (Kuczynski; C.; McCorkle; S.; Keereetaweep; J.; Shanklin; J.; & Schwender; J. (2022). An expanded role for the transcription factor WRINKLED1 in the biosynthesis of triacylglycerols during seed development. Frontiers in Plant Scienc Sobic.001G49550 no 57.28 57.28 OTHER 0.9225 OTHER 0.9290 no no nd epidermal lipid synthes KCS Ketoacyl-CoA Synthase AT4G3425 Manuscript (Kuczynski; C.; McCorkle; S.; Keereetaweep; J.; Shanklin; J.; & Schwender; J. (2022). An expanded role for the transcription factor WRINKLED1 in the biosynthesis of triacylglycerols during seed development. Frontiers in Plant Scienc Sobic.010G18150 no 57.23 57.23 OTHER 0.9225 OTHER 0.9988 no no 110.4

epidermal lipid synthes KCS Ketoacyl-CoA Synthase AT4G3425 Manuscript (Kuczynski; C.; McCorkle; S.; Keereetaweep; J.; Shanklin; J.; & Schwender; J. (2022). An expanded role for the transcription factor WRINKLED1 in the biosynthesis of triacylglycerols during seed development. Frontiers in Plant Scienc Sobic.001G24770 no 56.14 56.14 OTHER 0.9225 OTHER 0.8671 no no nd epidermal lipid synthes KCS Ketoacyl-CoA Synthase AT4G3425 Manuscript (Kuczynski; C.; McCorkle; S.; Keereetaweep; J.; Shanklin; J.; & Schwender; J. (2022). An expanded role for the transcription factor WRINKLED1 in the biosynthesis of triacylglycerols during seed development. Frontiers in Plant Scienc Sobic.004G08680 no 56.08 56.08 OTHER 0.9225 OTHER 1.0000 no no 1524.2

epidermal lipid synthes KCS Ketoacyl-CoA Synthase AT4G3425 Manuscript (Kuczynski; C.; McCorkle; S.; Keereetaweep; J.; Shanklin; J.; & Schwender; J. (2022). An expanded role for the transcription factor WRINKLED1 in the biosynthesis of triacylglycerols during seed development. Frontiers in Plant Scienc Sobic.009G24100 no 55.94 55.94 OTHER 0.9225 OTHER 0.9999 no no 632.4

epidermal lipid synthes KCS Ketoacyl-CoA Synthase AT4G3425 Manuscript (Kuczynski; C.; McCorkle; S.; Keereetaweep; J.; Shanklin; J.; & Schwender; J. (2022). An expanded role for the transcription factor WRINKLED1 in the biosynthesis of triacylglycerols during seed development. Frontiers in Plant Scienc Sobic.001G45320 no 55.37 55.37 OTHER 0.9225 OTHER 0.9580 no no 2162.6

epidermal lipid synthes KCS Ketoacyl-CoA Synthase AT4G3451 Manuscript (Kuczynski; C.; McCorkle; S.; Keereetaweep; J.; Shanklin; J.; & Schwender; J. (2022). An expanded role for the transcription factor WRINKLED1 in the biosynthesis of triacylglycerols during seed development. Frontiers in Plant Scienc Sobic.009G23610 yes 71.64 71.64 OTHER 0.9999 OTHER 0.9997 ortholo no 5240.3

epidermal lipid synthes KCS Ketoacyl-CoA Synthase AT4G3451 Manuscript (Kuczynski; C.; McCorkle; S.; Keereetaweep; J.; Shanklin; J.; & Schwender; J. (2022). An expanded role for the transcription factor WRINKLED1 in the biosynthesis of triacylglycerols during seed development. Frontiers in Plant Scienc Sobic.010G18150 no 62.89 62.89 OTHER 0.9999 OTHER 0.9988 no no 110.4

epidermal lipid synthes KCS Ketoacyl-CoA Synthase AT4G3451 Manuscript (Kuczynski; C.; McCorkle; S.; Keereetaweep; J.; Shanklin; J.; & Schwender; J. (2022). An expanded role for the transcription factor WRINKLED1 in the biosynthesis of triacylglycerols during seed development. Frontiers in Plant Scienc Sobic.004G08680 no 61.59 61.59 OTHER 0.9999 OTHER 1.0000 no no 1524.2

epidermal lipid synthes KCS Ketoacyl-CoA Synthase AT4G3451 Manuscript (Kuczynski; C.; McCorkle; S.; Keereetaweep; J.; Shanklin; J.; & Schwender; J. (2022). An expanded role for the transcription factor WRINKLED1 in the biosynthesis of triacylglycerols during seed development. Frontiers in Plant Scienc Sobic.001G45320 no 61.55 61.55 OTHER 0.9999 OTHER 0.9580 no no 2162.6

epidermal lipid synthes KCS Ketoacyl-CoA Synthase AT4G3451 Manuscript (Kuczynski; C.; McCorkle; S.; Keereetaweep; J.; Shanklin; J.; & Schwender; J. (2022). An expanded role for the transcription factor WRINKLED1 in the biosynthesis of triacylglycerols during seed development. Frontiers in Plant Scienc Sobic.009G24100 no 61.54 61.54 OTHER 0.9999 OTHER 0.9999 no no 632.4

epidermal lipid synthes KCS Ketoacyl-CoA Synthase AT4G3452 Manuscript (Kuczynski; C.; McCorkle; S.; Keereetaweep; J.; Shanklin; J.; & Schwender; J. (2022). An expanded role for the transcription factor WRINKLED1 in the biosynthesis of triacylglycerols during seed development. Frontiers in Plant Scienc Sobic.005G16900 no 62.5 62.5 OTHER 0.9691 OTHER 0.9810 no no nd epidermal lipid synthes KCS Ketoacyl-CoA Synthase AT4G3452 Manuscript (Kuczynski; C.; McCorkle; S.; Keereetaweep; J.; Shanklin; J.; & Schwender; J. (2022). An expanded role for the transcription factor WRINKLED1 in the biosynthesis of triacylglycerols during seed development. Frontiers in Plant Scienc Sobic.009G23610 yes 60.85 60.85 OTHER 0.9691 OTHER 0.9997 ortholo no 5240.3

epidermal lipid synthes KCS Ketoacyl-CoA Synthase AT4G3452 Manuscript (Kuczynski; C.; McCorkle; S.; Keereetaweep; J.; Shanklin; J.; & Schwender; J. (2022). An expanded role for the transcription factor WRINKLED1 in the biosynthesis of triacylglycerols during seed development. Frontiers in Plant Scienc Sobic.010G18150 no 58.44 58.44 OTHER 0.9691 OTHER 0.9988 no no 110.4

epidermal lipid synthes KCS Ketoacyl-CoA Synthase AT4G3452 Manuscript (Kuczynski; C.; McCorkle; S.; Keereetaweep; J.; Shanklin; J.; & Schwender; J. (2022). An expanded role for the transcription factor WRINKLED1 in the biosynthesis of triacylglycerols during seed development. Frontiers in Plant Scienc Sobic.009G24100 no 56.53 56.53 OTHER 0.9691 OTHER 0.9999 no no 632.4

epidermal lipid synthes KCS Ketoacyl-CoA Synthase AT4G3452 Manuscript (Kuczynski; C.; McCorkle; S.; Keereetaweep; J.; Shanklin; J.; & Schwender; J. (2022). An expanded role for the transcription factor WRINKLED1 in the biosynthesis of triacylglycerols during seed development. Frontiers in Plant Scienc Sobic.004G08680 no 56.38 56.38 OTHER 0.9691 OTHER 1.0000 no no 1524.2

epidermal lipid synthes KCS Ketoacyl-CoA Synthase AT5G0453 Manuscript (Kuczynski; C.; McCorkle; S.; Keereetaweep; J.; Shanklin; J.; & Schwender; J. (2022). An expanded role for the transcription factor WRINKLED1 in the biosynthesis of triacylglycerols during seed development. Frontiers in Plant Scienc Sobic.001G22190 yes 49.89 49.89 OTHER 0.7783 SP 0.8118 ortholo no nd epidermal lipid synthes KCS Ketoacyl-CoA Synthase AT5G0453 Manuscript (Kuczynski; C.; McCorkle; S.; Keereetaweep; J.; Shanklin; J.; & Schwender; J. (2022). An expanded role for the transcription factor WRINKLED1 in the biosynthesis of triacylglycerols during seed development. Frontiers in Plant Scienc Sobic.001G35470 no 49.78 49.78 OTHER 0.7783 SP 0.8404 ortholo no 1551.1

epidermal lipid synthes KCS Ketoacyl-CoA Synthase AT5G0453 Manuscript (Kuczynski; C.; McCorkle; S.; Keereetaweep; J.; Shanklin; J.; & Schwender; J. (2022). An expanded role for the transcription factor WRINKLED1 in the biosynthesis of triacylglycerols during seed development. Frontiers in Plant Scienc Sobic.004G34130 no 49.43 49.43 OTHER 0.7783 SP 0.8570 ortholo no 3.7

epidermal lipid synthes KCS Ketoacyl-CoA Synthase AT5G0453 Manuscript (Kuczynski; C.; McCorkle; S.; Keereetaweep; J.; Shanklin; J.; & Schwender; J. (2022). An expanded role for the transcription factor WRINKLED1 in the biosynthesis of triacylglycerols during seed development. Frontiers in Plant Scienc Sobic.005G16900 no 43.64 43.64 OTHER 0.7783 OTHER 0.9810 no no nd epidermal lipid synthes HACD Hydroxyacyl-CoA Dehydratas AT5G1048 Manuscript (Kuczynski; C.; McCorkle; S.; Keereetaweep; J.; Shanklin; J.; & Schwender; J. (2022). An expanded role for the transcription factor WRINKLED1 in the biosynthesis of triacylglycerols during seed development. Frontiers in Plant Scienc Sobic.003G07140 bidirectiona 73.18 75.57 OTHER 0.9586 OTHER 0.9708 LDO no 901.9

epidermal lipid synthes HACD Hydroxyacyl-CoA Dehydratas AT5G1048 Manuscript (Kuczynski; C.; McCorkle; S.; Keereetaweep; J.; Shanklin; J.; & Schwender; J. (2022). An expanded role for the transcription factor WRINKLED1 in the biosynthesis of triacylglycerols during seed development. Frontiers in Plant Scienc Sobic.003G07130 yes 52 57.07 OTHER 0.9586 OTHER 0.9997 ortholo no nd epidermal lipid synthes HACD Hydroxyacyl-CoA Dehydratas AT5G1048 Manuscript (Kuczynski; C.; McCorkle; S.; Keereetaweep; J.; Shanklin; J.; & Schwender; J. (2022). An expanded role for the transcription factor WRINKLED1 in the biosynthesis of triacylglycerols during seed development. Frontiers in Plant Scienc Sobic.003G07110 yes 40.76 50 OTHER 0.9586 OTHER 0.9964 ortholo no nd epidermal lipid synthes KCS Ketoacyl-CoA Synthase AT5G4376 Manuscript (Kuczynski; C.; McCorkle; S.; Keereetaweep; J.; Shanklin; J.; & Schwender; J. (2022). An expanded role for the transcription factor WRINKLED1 in the biosynthesis of triacylglycerols during seed development. Frontiers in Plant Scienc Sobic.010G18150 yes 74.75 74.35 OTHER 0.9998 OTHER 0.9988 no no 110.4

epidermal lipid synthes KCS Ketoacyl-CoA Synthase AT5G4376 Manuscript (Kuczynski; C.; McCorkle; S.; Keereetaweep; J.; Shanklin; J.; & Schwender; J. (2022). An expanded role for the transcription factor WRINKLED1 in the biosynthesis of triacylglycerols during seed development. Frontiers in Plant Scienc Sobic.004G08680 yes 69.84 69.77 OTHER 0.9998 OTHER 1.0000 no no 1524.2

epidermal lipid synthes KCS Ketoacyl-CoA Synthase AT5G4376 Manuscript (Kuczynski; C.; McCorkle; S.; Keereetaweep; J.; Shanklin; J.; & Schwender; J. (2022). An expanded role for the transcription factor WRINKLED1 in the biosynthesis of triacylglycerols during seed development. Frontiers in Plant Scienc Sobic.005G16870 yes 69.4 69.46 OTHER 0.9998 OTHER 0.9985 ortholo no 3001.9

epidermal lipid synthes KCS Ketoacyl-CoA Synthase AT5G4376 Manuscript (Kuczynski; C.; McCorkle; S.; Keereetaweep; J.; Shanklin; J.; & Schwender; J. (2022). An expanded role for the transcription factor WRINKLED1 in the biosynthesis of triacylglycerols during seed development. Frontiers in Plant Scienc Sobic.009G24100 yes 68.62 68.62 OTHER 0.9998 OTHER 0.9999 no no 632.4

epidermal lipid synthes KCS Ketoacyl-CoA Synthase AT5G4376 Manuscript (Kuczynski; C.; McCorkle; S.; Keereetaweep; J.; Shanklin; J.; & Schwender; J. (2022). An expanded role for the transcription factor WRINKLED1 in the biosynthesis of triacylglycerols during seed development. Frontiers in Plant Scienc Sobic.009G23610 yes 65.86 65.86 OTHER 0.9998 OTHER 0.9997 no no 5240.3

epidermal lipid synthes KCS Ketoacyl-CoA Synthase AT5G4376 Manuscript (Kuczynski; C.; McCorkle; S.; Keereetaweep; J.; Shanklin; J.; & Schwender; J. (2022). An expanded role for the transcription factor WRINKLED1 in the biosynthesis of triacylglycerols during seed development. Frontiers in Plant Scienc Sobic.005G16900 yes 64.29 64.29 OTHER 0.9998 OTHER 0.9810 no no nd epidermal lipid synthes KCS Ketoacyl-CoA Synthase AT5G4376 Manuscript (Kuczynski; C.; McCorkle; S.; Keereetaweep; J.; Shanklin; J.; & Schwender; J. (2022). An expanded role for the transcription factor WRINKLED1 in the biosynthesis of triacylglycerols during seed development. Frontiers in Plant Scienc Sobic.001G45320 yes 61.21 61.21 OTHER 0.9998 OTHER 0.9580 no no 2162.6

epidermal lipid synthes KCS Ketoacyl-CoA Synthase AT5G4376 Manuscript (Kuczynski; C.; McCorkle; S.; Keereetaweep; J.; Shanklin; J.; & Schwender; J. (2022). An expanded role for the transcription factor WRINKLED1 in the biosynthesis of triacylglycerols during seed development. Frontiers in Plant Scienc Sobic.001G24770 yes 61.05 61.05 OTHER 0.9998 OTHER 0.8671 no no nd epidermal lipid synthes KCS Ketoacyl-CoA Synthase AT5G4907 Manuscript (Kuczynski; C.; McCorkle; S.; Keereetaweep; J.; Shanklin; J.; & Schwender; J. (2022). An expanded role for the transcription factor WRINKLED1 in the biosynthesis of triacylglycerols during seed development. Frontiers in Plant Scienc Sobic.001G45320 yes 52.25 52.25 OTHER 0.9911 OTHER 0.9580 no no 2162.6

epidermal lipid synthes KCS Ketoacyl-CoA Synthase AT5G4907 Manuscript (Kuczynski; C.; McCorkle; S.; Keereetaweep; J.; Shanklin; J.; & Schwender; J. (2022). An expanded role for the transcription factor WRINKLED1 in the biosynthesis of triacylglycerols during seed development. Frontiers in Plant Scienc Sobic.004G08680 no 49.66 49.66 OTHER 0.9911 OTHER 1.0000 no no 1524.2

epidermal lipid synthes KCS Ketoacyl-CoA Synthase AT5G4907 Manuscript (Kuczynski; C.; McCorkle; S.; Keereetaweep; J.; Shanklin; J.; & Schwender; J. (2022). An expanded role for the transcription factor WRINKLED1 in the biosynthesis of triacylglycerols during seed development. Frontiers in Plant Scienc Sobic.009G24100 no 48.95 48.95 OTHER 0.9911 OTHER 0.9999 no no 632.4

epidermal lipid synthes KCS Ketoacyl-CoA Synthase AT5G4907 Manuscript (Kuczynski; C.; McCorkle; S.; Keereetaweep; J.; Shanklin; J.; & Schwender; J. (2022). An expanded role for the transcription factor WRINKLED1 in the biosynthesis of triacylglycerols during seed development. Frontiers in Plant Scienc Sobic.010G18150 no 48.6 48.6 OTHER 0.9911 OTHER 0.9988 no no 110.4

epidermal lipid synthes KCS Ketoacyl-CoA Synthase AT5G4907 Manuscript (Kuczynski; C.; McCorkle; S.; Keereetaweep; J.; Shanklin; J.; & Schwender; J. (2022). An expanded role for the transcription factor WRINKLED1 in the biosynthesis of triacylglycerols during seed development. Frontiers in Plant Scienc Sobic.009G23610 no 48.28 48.28 OTHER 0.9911 OTHER 0.9997 no no 5240.3

epidermal lipid synthes KCS Ketoacyl-CoA Synthase AT5G4907 Manuscript (Kuczynski; C.; McCorkle; S.; Keereetaweep; J.; Shanklin; J.; & Schwender; J. (2022). An expanded role for the transcription factor WRINKLED1 in the biosynthesis of triacylglycerols during seed development. Frontiers in Plant Scienc Sobic.004G24940 no 47.02 47.02 OTHER 0.9911 OTHER 0.9561 no no 1669.3

epidermal lipid synthes KCS Ketoacyl-CoA Synthase AT5G4907 Manuscript (Kuczynski; C.; McCorkle; S.; Keereetaweep; J.; Shanklin; J.; & Schwender; J. (2022). An expanded role for the transcription factor WRINKLED1 in the biosynthesis of triacylglycerols during seed development. Frontiers in Plant Scienc Sobic.010G10720 no 45.9 45.9 OTHER 0.9911 OTHER 0.9513 no no 19.2

epidermal lipid synthes KCS Ketoacyl-CoA Synthase AT5G4907 Manuscript (Kuczynski; C.; McCorkle; S.; Keereetaweep; J.; Shanklin; J.; & Schwender; J. (2022). An expanded role for the transcription factor WRINKLED1 in the biosynthesis of triacylglycerols during seed development. Frontiers in Plant Scienc Sobic.001G26450 no 45.87 45.87 OTHER 0.9911 OTHER 0.9999 no no 21.5

epidermal lipid synthes KCS Ketoacyl-CoA Synthase AT5G4907 Manuscript (Kuczynski; C.; McCorkle; S.; Keereetaweep; J.; Shanklin; J.; & Schwender; J. (2022). An expanded role for the transcription factor WRINKLED1 in the biosynthesis of triacylglycerols during seed development. Frontiers in Plant Scienc Sobic.001G24770 no 45.76 45.76 OTHER 0.9911 OTHER 0.8671 no no nd epidermal lipid synthes HACD Hydroxyacyl-CoA Dehydratas AT5G5977 Manuscript (Kuczynski; C.; McCorkle; S.; Keereetaweep; J.; Shanklin; J.; & Schwender; J. (2022). An expanded role for the transcription factor WRINKLED1 in the biosynthesis of triacylglycerols during seed development. Frontiers in Plant Scienc Sobic.009G16440 bidirectiona 59.56 51.61 OTHER 0.9261 OTHER 0.7282 LDO no 790.3

glycerolipid synthes GPAT glycerol-3-phosphate acyltransferase 1 (GPAT1 AT1G0161 Manuscript (Kuczynski; C.; McCorkle; S.; Keereetaweep; J.; Shanklin; J.; & Schwender; J. (2022). An expanded role for the transcription factor WRINKLED1 in the biosynthesis of triacylglycerols during seed development. Frontiers in Plant Scienc Sobic.003G36070 yes 60.36 60.69 OTHER 1.0000 OTHER 0.9514 no no 166.3

glycerolipid synthes GPAT glycerol-3-phosphate acyltransferase 1 (GPAT1 AT1G0161 Manuscript (Kuczynski; C.; McCorkle; S.; Keereetaweep; J.; Shanklin; J.; & Schwender; J. (2022). An expanded role for the transcription factor WRINKLED1 in the biosynthesis of triacylglycerols during seed development. Frontiers in Plant Scienc Sobic.004G01030 yes 56.31 54.89 OTHER 1.0000 OTHER 0.9146 ortholo no 452.6

glycerolipid synthes GPAT glycerol-3-phosphate acyltransferase 1 (GPAT1 AT1G0239 Manuscript (Kuczynski; C.; McCorkle; S.; Keereetaweep; J.; Shanklin; J.; & Schwender; J. (2022). An expanded role for the transcription factor WRINKLED1 in the biosynthesis of triacylglycerols during seed development. Frontiers in Plant Scienc Sobic.005G21440 no 46.56 45.38 OTHER 0.9138 OTHER 0.5791 ortholo no 487.0

glycerolipid synthes GPAT glycerol-3-phosphate acyltransferase 1 (GPAT1 AT1G0239 Manuscript (Kuczynski; C.; McCorkle; S.; Keereetaweep; J.; Shanklin; J.; & Schwender; J. (2022). An expanded role for the transcription factor WRINKLED1 in the biosynthesis of triacylglycerols during seed development. Frontiers in Plant Scienc Sobic.001G02610 no 47.84 44.38 OTHER 0.9138 cTP 0.0510 ortholo no nd glycerolipid synthes GPAT glycerol-3-phosphate acyltransferase 1 (GPAT1 AT1G0239 Manuscript (Kuczynski; C.; McCorkle; S.; Keereetaweep; J.; Shanklin; J.; & Schwender; J. (2022). An expanded role for the transcription factor WRINKLED1 in the biosynthesis of triacylglycerols during seed development. Frontiers in Plant Scienc Sobic.003G11420 no 42.66 42.2 OTHER 0.9138 cTP 0.3941 ortholo no 191.6

glycerolipid synthes GPAT glycerol-3-phosphate acyltransferase 1 (GPAT1 AT1G0239 Manuscript (Kuczynski; C.; McCorkle; S.; Keereetaweep; J.; Shanklin; J.; & Schwender; J. (2022). An expanded role for the transcription factor WRINKLED1 in the biosynthesis of triacylglycerols during seed development. Frontiers in Plant Scienc Sobic.003G14250 no 43.28 42.94 OTHER 0.9138 OTHER 0.8184 LDO no nd glycerolipid synthes GPAT glycerol-3-phosphate acyltransferase 1 (GPAT1 AT1G0239 Manuscript (Kuczynski; C.; McCorkle; S.; Keereetaweep; J.; Shanklin; J.; & Schwender; J. (2022). An expanded role for the transcription factor WRINKLED1 in the biosynthesis of triacylglycerols during seed development. Frontiers in Plant Scienc Sobic.008G13080 no 42.03 42.48 OTHER 0.9138 cTP 0.6869 ortholo no 83.5

glycerolipid synthes GPAT glycerol-3-phosphate acyltransferase 1 (GPAT1 AT1G0652 Manuscript (Kuczynski; C.; McCorkle; S.; Keereetaweep; J.; Shanklin; J.; & Schwender; J. (2022). An expanded role for the transcription factor WRINKLED1 in the biosynthesis of triacylglycerols during seed development. Frontiers in Plant Scienc Sobic.001G09930 yes 45.07 45 OTHER 0.9403 OTHER 0.9897 ortholo no nd glycerolipid synthes LPCAT Lysophosphatidylcholine acyltransfera AT1G1264 Manuscript (Kuczynski; C.; McCorkle; S.; Keereetaweep; J.; Shanklin; J.; & Schwender; J. (2022). An expanded role for the transcription factor WRINKLED1 in the biosynthesis of triacylglycerols during seed development. Frontiers in Plant Scienc Sobic.004G28360 yes 70.19 70.77 OTHER 0.7577 OTHER 0.9734 LDO no 2174.3

glycerolipid synthes PP phosphate phosphatase AT1G1508 Manuscript (Kuczynski; C.; McCorkle; S.; Keereetaweep; J.; Shanklin; J.; & Schwender; J. (2022). An expanded role for the transcription factor WRINKLED1 in the biosynthesis of triacylglycerols during seed development. Frontiers in Plant Scienc Sobic.002G16514 bidirectiona 68.53 68.07 OTHER 0.9954 OTHER 0.9718 LDO no 2891.9

glycerolipid synthes PP phosphate phosphatase AT1G1508 Manuscript (Kuczynski; C.; McCorkle; S.; Keereetaweep; J.; Shanklin; J.; & Schwender; J. (2022). An expanded role for the transcription factor WRINKLED1 in the biosynthesis of triacylglycerols during seed development. Frontiers in Plant Scienc Sobic.007G10850 yes 67.6 67.72 OTHER 0.9954 OTHER 0.9956 ortholo no 2950.4

glycerolipid synthes PP phosphate phosphatase AT1G1508 Manuscript (Kuczynski; C.; McCorkle; S.; Keereetaweep; J.; Shanklin; J.; & Schwender; J. (2022). An expanded role for the transcription factor WRINKLED1 in the biosynthesis of triacylglycerols during seed development. Frontiers in Plant Scienc Sobic.003G24900 no 63 61.23 OTHER 0.9954 OTHER 0.0274 ortholo no 2.2

glycerolipid synthes PP phosphate phosphatase AT1G1508 Manuscript (Kuczynski; C.; McCorkle; S.; Keereetaweep; J.; Shanklin; J.; & Schwender; J. (2022). An expanded role for the transcription factor WRINKLED1 in the biosynthesis of triacylglycerols during seed development. Frontiers in Plant Scienc Sobic.003G26510 yes 66.91 63.07 OTHER 0.9954 OTHER 0.9933 ortholo no 282.7

glycerolipid synthes PP phosphate phosphatase AT1G1508 Manuscript (Kuczynski; C.; McCorkle; S.; Keereetaweep; J.; Shanklin; J.; & Schwender; J. (2022). An expanded role for the transcription factor WRINKLED1 in the biosynthesis of triacylglycerols during seed development. Frontiers in Plant Scienc Sobic.009G22150 yes 64.31 62.72 OTHER 0.9954 OTHER 0.9999 ortholo no 14.6

| glycerolipid synthes | PP | phosphate phosphatase | AT1G1508 Manuscript (Kuczynski; C.; McCorkle; S.; Keereetaweep; J.; Shanklin; J.; & Schwender; J. (2022). An expanded role for the transcription factor WRINKLED1 in the biosynthesis of triacylglycerols during seed development. Frontiers in Plant Scienc | Sobic.010G19030 | yes | 63.77 | 61.25 | OTHER | 0.9954 OTHER 0.9688 | ortholo | no | nd |
| --- | --- | --- | --- | --- | --- | --- | --- | --- | --- | --- | --- | --- |
| glycerolipid synthes | PP | phosphate phosphatase | AT1G1508 Manuscript (Kuczynski; C.; McCorkle; S.; Keereetaweep; J.; Shanklin; J.; & Schwender; J. (2022). An expanded role for the transcription factor WRINKLED1 in the biosynthesis of triacylglycerols during seed development. Frontiers in Plant Scienc | Sobic.010G19330 | no | 60.97 | 56.94 | OTHER | 0.9954 OTHER 0.9880 | ortholo | no | 503.9 |
| glycerolipid synthes | GPAT | glycerol-3-phosphate acyltransferase 1 (GPAT1 | AT1G3220 Manuscript (Kuczynski; C.; McCorkle; S.; Keereetaweep; J.; Shanklin; J.; & Schwender; J. (2022). An expanded role for the transcription factor WRINKLED1 in the biosynthesis of triacylglycerols during seed development. Frontiers in Plant Scienc | Sobic.001G28370 | bidirectiona | 66.76 | 58.62 | cTP | 0.9980 cTP 0.9918 | LDO | no | 1404.9 |
| glycerolipid synthes | DGAT3 | Diacylglycerol Acyltransferas | AT1G4830 Manuscript (Kuczynski; C.; McCorkle; S.; Keereetaweep; J.; Shanklin; J.; & Schwender; J. (2022). An expanded role for the transcription factor WRINKLED1 in the biosynthesis of triacylglycerols during seed development. Frontiers in Plant Scienc | Sobic.009G03460 | bidirectiona | 39.34 | 36.8 | OTHER | 1.0000 OTHER 0.5823 | no | no | 4782.7 |
| glycerolipid synthes | LPAAT | Lysophosphatidic Acid Acyltransferas | AT1G5126 Manuscript (Kuczynski; C.; McCorkle; S.; Keereetaweep; J.; Shanklin; J.; & Schwender; J. (2022). An expanded role for the transcription factor WRINKLED1 in the biosynthesis of triacylglycerols during seed development. Frontiers in Plant Scienc | Sobic.005G19710 | no | 56.81 | 55.08 | OTHER | 0.9969 OTHER 0.6431 | ortholo | no | 1731.7 |
| glycerolipid synthes | LPAAT | Lysophosphatidic Acid Acyltransferas | AT1G5126 Manuscript (Kuczynski; C.; McCorkle; S.; Keereetaweep; J.; Shanklin; J.; & Schwender; J. (2022). An expanded role for the transcription factor WRINKLED1 in the biosynthesis of triacylglycerols during seed development. Frontiers in Plant Scienc | Sobic.008G11210 | no | 58.14 | 56.68 | OTHER | 0.9969 OTHER 0.8623 | ortholo | no | 4.0 |
| glycerolipid synthes | LPCAT | Lysophosphatidylcholine acyltransfera | AT1G6305 Manuscript (Kuczynski; C.; McCorkle; S.; Keereetaweep; J.; Shanklin; J.; & Schwender; J. (2022). An expanded role for the transcription factor WRINKLED1 in the biosynthesis of triacylglycerols during seed development. Frontiers in Plant Scienc | Sobic.004G28360 | yes | 69.33 | 69.58 | OTHER | 0.9974 OTHER 0.9734 | ortholo | no | 2174.3 |
| glycerolipid synthes | LPAAT | Lysophosphatidic Acid Acyltransferas | AT1G7502 Manuscript (Kuczynski; C.; McCorkle; S.; Keereetaweep; J.; Shanklin; J.; & Schwender; J. (2022). An expanded role for the transcription factor WRINKLED1 in the biosynthesis of triacylglycerols during seed development. Frontiers in Plant Scienc | Sobic.003G31750 | yes | 58.59 | 56.3 | OTHER | 0.9980 OTHER 0.9999 | ortholo | no | 2415.8 |
| glycerolipid synthes | LPAAT | Lysophosphatidic Acid Acyltransferas | AT1G7502 Manuscript (Kuczynski; C.; McCorkle; S.; Keereetaweep; J.; Shanklin; J.; & Schwender; J. (2022). An expanded role for the transcription factor WRINKLED1 in the biosynthesis of triacylglycerols during seed development. Frontiers in Plant Scienc | Sobic.009G18890 | no | 54.29 | 50.53 | OTHER | 0.9980 OTHER 1.0000 | ortholo | no | 136.8 |
| glycerolipid synthes | LPCAT | Lysophosphatidylcholine acyltransfera | AT1G7869 Manuscript (Kuczynski; C.; McCorkle; S.; Keereetaweep; J.; Shanklin; J.; & Schwender; J. (2022). An expanded role for the transcription factor WRINKLED1 in the biosynthesis of triacylglycerols during seed development. Frontiers in Plant Scienc | Sobic.006G25730 | bidirectiona | 60.79 | 60.93 | OTHER | 0.9524 OTHER 0.7726 | LDO | no | 209.9 |
| glycerolipid synthes | LPCAT | Lysophosphatidylcholine acyltransfera | AT1G8095 Manuscript (Kuczynski; C.; McCorkle; S.; Keereetaweep; J.; Shanklin; J.; & Schwender; J. (2022). An expanded role for the transcription factor WRINKLED1 in the biosynthesis of triacylglycerols during seed development. Frontiers in Plant Scienc | Sobic.003G14590 | yes | 58.97 | 61.81 | OTHER | 1.0000 OTHER 1.0000 | ortholo | no | 293.9 |
| glycerolipid synthes | LPCAT | Lysophosphatidylcholine acyltransfera | AT1G8095 Manuscript (Kuczynski; C.; McCorkle; S.; Keereetaweep; J.; Shanklin; J.; & Schwender; J. (2022). An expanded role for the transcription factor WRINKLED1 in the biosynthesis of triacylglycerols during seed development. Frontiers in Plant Scienc | Sobic.009G10850 | yes | 60.76 | 59.04 | OTHER | 1.0000 OTHER 0.9999 | LDO | no | 539.2 |
| glycerolipid synthes | PP | phosphate phosphatase | AT2G0118 Manuscript (Kuczynski; C.; McCorkle; S.; Keereetaweep; J.; Shanklin; J.; & Schwender; J. (2022). An expanded role for the transcription factor WRINKLED1 in the biosynthesis of triacylglycerols during seed development. Frontiers in Plant Scienc | Sobic.003G07870 | yes | 57.84 | 58.64 | OTHER | 0.9950 OTHER 0.9723 | ortholo | no | 31.5 |
| glycerolipid synthes | PP | phosphate phosphatase | AT2G0118 Manuscript (Kuczynski; C.; McCorkle; S.; Keereetaweep; J.; Shanklin; J.; & Schwender; J. (2022). An expanded role for the transcription factor WRINKLED1 in the biosynthesis of triacylglycerols during seed development. Frontiers in Plant Scienc | Sobic.007G10850 | yes | 55.37 | 57.09 | OTHER | 0.9950 OTHER 0.9956 | no | no | 2950.4 |
| glycerolipid synthes | DGAT1 | Diacylglycerol Acyltransferas | AT2G1945 Manuscript (Kuczynski; C.; McCorkle; S.; Keereetaweep; J.; Shanklin; J.; & Schwender; J. (2022). An expanded role for the transcription factor WRINKLED1 in the biosynthesis of triacylglycerols during seed development. Frontiers in Plant Scienc | Sobic.010G17000 | bidirectiona | 64.21 | 66.24 | OTHER | 0.9999 OTHER 1.0000 | LDO | no | 497.3 |
| glycerolipid synthes | DGAT1 | Diacylglycerol Acyltransferas | AT2G1945 Manuscript (Kuczynski; C.; McCorkle; S.; Keereetaweep; J.; Shanklin; J.; & Schwender; J. (2022). An expanded role for the transcription factor WRINKLED1 in the biosynthesis of triacylglycerols during seed development. Frontiers in Plant Scienc | Sobic.009G07270 | yes | 63.16 | 60.73 | OTHER | 0.9999 OTHER 0.9997 | ortholo | no | 433.7 |
| glycerolipid synthes | PLA | Phospholipase A | AT2G1969 Manuscript (Kuczynski; C.; McCorkle; S.; Keereetaweep; J.; Shanklin; J.; & Schwender; J. (2022). An expanded role for the transcription factor WRINKLED1 in the biosynthesis of triacylglycerols during seed development. Frontiers in Plant Scienc | Sobic.004G35780 | bidirectiona | 46.62 | 48.12 | SP | 1.0000 SP 1.0000 | LDO | no | 119.5 |
| glycerolipid synthes | FAD3 | Oleate Desaturase | AT2G2998 Manuscript (Kuczynski; C.; McCorkle; S.; Keereetaweep; J.; Shanklin; J.; & Schwender; J. (2022). An expanded role for the transcription factor WRINKLED1 in the biosynthesis of triacylglycerols during seed development. Frontiers in Plant Scienc | Sobic.001G40760 | yes | 69.44 | 67.62 | OTHER | 0.9999 cTP 0.6195 | ortholo | no | 13263.7 |
| glycerolipid synthes | FAD3 | Oleate Desaturase | AT2G2998 Manuscript (Kuczynski; C.; McCorkle; S.; Keereetaweep; J.; Shanklin; J.; & Schwender; J. (2022). An expanded role for the transcription factor WRINKLED1 in the biosynthesis of triacylglycerols during seed development. Frontiers in Plant Scienc | Sobic.005G00270 | no | 67.58 | 65.08 | OTHER | 0.9999 OTHER 1.0000 | ortholo | no | nd |
| glycerolipid synthes | FAD3 | Oleate Desaturase | AT2G2998 Manuscript (Kuczynski; C.; McCorkle; S.; Keereetaweep; J.; Shanklin; J.; & Schwender; J. (2022). An expanded role for the transcription factor WRINKLED1 in the biosynthesis of triacylglycerols during seed development. Frontiers in Plant Scienc | Sobic.005G00280 | no | 64.51 | 64.75 | OTHER | 0.9999 OTHER 0.9982 | ortholo | no | nd |
| glycerolipid synthes | FAD3 | Oleate Desaturase | AT2G2998 Manuscript (Kuczynski; C.; McCorkle; S.; Keereetaweep; J.; Shanklin; J.; & Schwender; J. (2022). An expanded role for the transcription factor WRINKLED1 in the biosynthesis of triacylglycerols during seed development. Frontiers in Plant Scienc | Sobic.008G00280 | no | 67.33 | 63.66 | OTHER | 0.9999 OTHER 0.9999 | ortholo | no | nd |
| glycerolipid synthes | FAD3 | Oleate Desaturase | AT2G2998 Manuscript (Kuczynski; C.; McCorkle; S.; Keereetaweep; J.; Shanklin; J.; & Schwender; J. (2022). An expanded role for the transcription factor WRINKLED1 in the biosynthesis of triacylglycerols during seed development. Frontiers in Plant Scienc | Sobic.008G00320 | no | 67.12 | 65.01 | OTHER | 0.9999 OTHER 0.9994 | ortholo | no | 32.0 |
| glycerolipid synthes | FAD3 | Oleate Desaturase | AT2G2998 Manuscript (Kuczynski; C.; McCorkle; S.; Keereetaweep; J.; Shanklin; J.; & Schwender; J. (2022). An expanded role for the transcription factor WRINKLED1 in the biosynthesis of triacylglycerols during seed development. Frontiers in Plant Scienc | Sobic.002G43010 | no | 71.85 | 67.47 | OTHER | 0.9999 cTP 0.8832 | ortholo | no | 1095.7 |
| glycerolipid synthes | GPAT | glycerol-3-phosphate acyltransferase 1 (GPAT1 | AT2G3811 Manuscript (Kuczynski; C.; McCorkle; S.; Keereetaweep; J.; Shanklin; J.; & Schwender; J. (2022). An expanded role for the transcription factor WRINKLED1 in the biosynthesis of triacylglycerols during seed development. Frontiers in Plant Scienc | Sobic.003G36070 | bidirectiona | 72.53 | 72.29 | OTHER | 1.0000 OTHER 0.9514 | LDO | no | 166.3 |
| glycerolipid synthes | GPAT | glycerol-3-phosphate acyltransferase 1 (GPAT1 | AT2G3811 Manuscript (Kuczynski; C.; McCorkle; S.; Keereetaweep; J.; Shanklin; J.; & Schwender; J. (2022). An expanded role for the transcription factor WRINKLED1 in the biosynthesis of triacylglycerols during seed development. Frontiers in Plant Scienc | Sobic.009G15700 | yes | 52.71 | 59.76 | OTHER | 1.0000 OTHER 0.9970 | ortholo | no | 9.7 |
| glycerolipid synthes | LPCAT | Lysophosphatidylcholine acyltransfera | AT2G4567 Manuscript (Kuczynski; C.; McCorkle; S.; Keereetaweep; J.; Shanklin; J.; & Schwender; J. (2022). An expanded role for the transcription factor WRINKLED1 in the biosynthesis of triacylglycerols during seed development. Frontiers in Plant Scienc | Sobic.010G26150 | bidirectiona | 56.42 | 53.48 | OTHER | 1.0000 OTHER 0.9802 | LDO | no | 1543.6 |
| glycerolipid synthes | PP | phosphate phosphatase | AT3G0260 Manuscript (Kuczynski; C.; McCorkle; S.; Keereetaweep; J.; Shanklin; J.; & Schwender; J. (2022). An expanded role for the transcription factor WRINKLED1 in the biosynthesis of triacylglycerols during seed development. Frontiers in Plant Scienc | Sobic.003G07870 | yes | 66.19 | 62.25 | OTHER | 0.9699 OTHER 0.9723 | LDO | no | 31.5 |
| glycerolipid synthes | PP | phosphate phosphatase | AT3G0260 Manuscript (Kuczynski; C.; McCorkle; S.; Keereetaweep; J.; Shanklin; J.; & Schwender; J. (2022). An expanded role for the transcription factor WRINKLED1 in the biosynthesis of triacylglycerols during seed development. Frontiers in Plant Scienc | Sobic.007G10850 | yes | 61.46 | 62.42 | OTHER | 0.9699 OTHER 0.9956 | no | no | 2950.4 |
| glycerolipid synthes | PP | phosphate phosphatase | AT3G0956 Manuscript (Kuczynski; C.; McCorkle; S.; Keereetaweep; J.; Shanklin; J.; & Schwender; J. (2022). An expanded role for the transcription factor WRINKLED1 in the biosynthesis of triacylglycerols during seed development. Frontiers in Plant Scienc | Sobic.009G16540 | bidirectiona | 59.22 | 49.13 | OTHER | 0.7949 OTHER 0.9300 | LDO | no | 1190.7 |
| glycerolipid synthes | PP | phosphate phosphatase | AT3G0956 Manuscript (Kuczynski; C.; McCorkle; S.; Keereetaweep; J.; Shanklin; J.; & Schwender; J. (2022). An expanded role for the transcription factor WRINKLED1 in the biosynthesis of triacylglycerols during seed development. Frontiers in Plant Scienc | Sobic.005G18730 | no | 58.08 | 43.35 | OTHER | 0.7949 OTHER 0.6765 | no | no | 2001.4 |
| glycerolipid synthes | GPAT | glycerol-3-phosphate acyltransferase 1 (GPAT1 | AT3G1143 Manuscript (Kuczynski; C.; McCorkle; S.; Keereetaweep; J.; Shanklin; J.; & Schwender; J. (2022). An expanded role for the transcription factor WRINKLED1 in the biosynthesis of triacylglycerols during seed development. Frontiers in Plant Scienc | Sobic.009G16200 | bidirectiona | 61 | 59.27 | OTHER | 1.0000 OTHER 0.9888 | ortholo | no | 1752.0 |
| glycerolipid synthes | FAD2 | Oleate Desaturase | AT3G1212 Manuscript (Kuczynski; C.; McCorkle; S.; Keereetaweep; J.; Shanklin; J.; & Schwender; J. (2022). An expanded role for the transcription factor WRINKLED1 in the biosynthesis of triacylglycerols during seed development. Frontiers in Plant Scienc | Sobic.004G26060 | yes | 65.37 | 66.75 | OTHER | 0.9999 OTHER 1.0000 | ortholo | no | 27.4 |
| glycerolipid synthes | PDCT | Phosphatidylcholine:diacylglycerol cholinephosphotransfer | AT3G1212 Manuscript (Kuczynski; C.; McCorkle; S.; Keereetaweep; J.; Shanklin; J.; & Schwender; J. (2022). An expanded role for the transcription factor WRINKLED1 in the biosynthesis of triacylglycerols during seed development. Frontiers in Plant Scienc | Sobic.004G26080 | bidirectiona | 68.97 | 70.79 | OTHER | 0.9999 OTHER 1.0000 | LDO | no | 19892.9 |
| glycerolipid synthes | FAD2 | Oleate Desaturase | AT3G1212 Manuscript (Kuczynski; C.; McCorkle; S.; Keereetaweep; J.; Shanklin; J.; & Schwender; J. (2022). An expanded role for the transcription factor WRINKLED1 in the biosynthesis of triacylglycerols during seed development. Frontiers in Plant Scienc | Sobic.002G04500 | yes | 53.83 | 53.54 | OTHER | 0.9999 OTHER 0.9999 | ortholo | no | nd |
| glycerolipid synthes | PDCT | Phosphatidylcholine:diacylglycerol cholinephosphotransfer | AT3G1582 Manuscript (Kuczynski; C.; McCorkle; S.; Keereetaweep; J.; Shanklin; J.; & Schwender; J. (2022). An expanded role for the transcription factor WRINKLED1 in the biosynthesis of triacylglycerols during seed development. Frontiers in Plant Scienc | Sobic.010G18710 | bidirectiona | 60.56 | 52.03 | OTHER | 0.9998 OTHER 0.9994 | LDO | yes | 3085.6 |
| glycerolipid synthes | PP | phosphate phosphatase | AT3G1822 Manuscript (Kuczynski; C.; McCorkle; S.; Keereetaweep; J.; Shanklin; J.; & Schwender; J. (2022). An expanded role for the transcription factor WRINKLED1 in the biosynthesis of triacylglycerols during seed development. Frontiers in Plant Scienc | Sobic.003G24900 | no | 60.54 | 54.42 | OTHER | 0.9884 OTHER 0.9645 | no | no | 2.2 |
| glycerolipid synthes | PP | phosphate phosphatase | AT3G1822 Manuscript (Kuczynski; C.; McCorkle; S.; Keereetaweep; J.; Shanklin; J.; & Schwender; J. (2022). An expanded role for the transcription factor WRINKLED1 in the biosynthesis of triacylglycerols during seed development. Frontiers in Plant Scienc | Sobic.007G10850 | yes | 61.96 | 56.86 | OTHER | 0.9884 OTHER 0.9956 | no | no | 2950.4 |
| glycerolipid synthes | PP | phosphate phosphatase | AT3G1822 Manuscript (Kuczynski; C.; McCorkle; S.; Keereetaweep; J.; Shanklin; J.; & Schwender; J. (2022). An expanded role for the transcription factor WRINKLED1 in the biosynthesis of triacylglycerols during seed development. Frontiers in Plant Scienc | Sobic.003G07870 | no | 51.8 | 52.17 | OTHER | 0.9884 OTHER 0.9723 | ortholo | no | 31.5 |
| glycerolipid synthes | LPAAT | Lysophosphatidic Acid Acyltransferas | AT3G1885 Manuscript (Kuczynski; C.; McCorkle; S.; Keereetaweep; J.; Shanklin; J.; & Schwender; J. (2022). An expanded role for the transcription factor WRINKLED1 in the biosynthesis of triacylglycerols during seed development. Frontiers in Plant Scienc | Sobic.003G31750 | bidirectiona | 58.08 | 56.68 | OTHER | 0.9999 OTHER 0.9999 | LDO | no | 2415.8 |
| glycerolipid synthes | LPAAT | Lysophosphatidic Acid Acyltransferas | AT3G1885 Manuscript (Kuczynski; C.; McCorkle; S.; Keereetaweep; J.; Shanklin; J.; & Schwender; J. (2022). An expanded role for the transcription factor WRINKLED1 in the biosynthesis of triacylglycerols during seed development. Frontiers in Plant Scienc | Sobic.009G18890 | yes | 55.8 | 54.01 | OTHER | 0.9999 OTHER 1.0000 | ortholo | no | 136.8 |
| glycerolipid synthes | PLA | Phospholipase A | AT3G1886 Manuscript (Kuczynski; C.; McCorkle; S.; Keereetaweep; J.; Shanklin; J.; & Schwender; J. (2022). An expanded role for the transcription factor WRINKLED1 in the biosynthesis of triacylglycerols during seed development. Frontiers in Plant Scienc | Sobic.002G02070 | bidirectiona | 62.77 | 62.55 | OTHER | 0.9998 OTHER 0.9984 | LDO | no | 972.3 |
| glycerolipid synthes | PDAT | Phospholipid : Diacylglycerol Acyltransfera | AT3G4483 Manuscript (Kuczynski; C.; McCorkle; S.; Keereetaweep; J.; Shanklin; J.; & Schwender; J. (2022). An expanded role for the transcription factor WRINKLED1 in the biosynthesis of triacylglycerols during seed development. Frontiers in Plant Scienc | Sobic.002G21850 | yes | 60.09 | 60.06 | OTHER | 0.8793 OTHER 0.9998 | no | no | 2825.0 |
| glycerolipid synthes | PP | phosphate phosphatase | AT3G5092 Manuscript (Kuczynski; C.; McCorkle; S.; Keereetaweep; J.; Shanklin; J.; & Schwender; J. (2022). An expanded role for the transcription factor WRINKLED1 in the biosynthesis of triacylglycerols during seed development. Frontiers in Plant Scienc | Sobic.004G26830 | yes | 41.41 | 41.6 | lTP | 0.5036 cTP 0.9069 | no | no | 1000.4 |
| glycerolipid synthes | DGAT2 | Diacylglycerol Acyltransferas | AT3G5152 Manuscript (Kuczynski; C.; McCorkle; S.; Keereetaweep; J.; Shanklin; J.; & Schwender; J. (2022). An expanded role for the transcription factor WRINKLED1 in the biosynthesis of triacylglycerols during seed development. Frontiers in Plant Scienc | Sobic.004G26190 | bidirectiona | 57.64 | 56.87 | OTHER | 0.9994 OTHER 1.0000 | LDO | yes | 2681.0 |
| glycerolipid synthes | DGAT2 | Diacylglycerol Acyltransferas | AT3G5152 Manuscript (Kuczynski; C.; McCorkle; S.; Keereetaweep; J.; Shanklin; J.; & Schwender; J. (2022). An expanded role for the transcription factor WRINKLED1 in the biosynthesis of triacylglycerols during seed development. Frontiers in Plant Scienc | Sobic.008G04900 | yes | 50.56 | 46.02 | OTHER | 0.9994 OTHER 0.5059 | ortholo | yes | nd |
| glycerolipid synthes | DGAT2 | Diacylglycerol Acyltransferas | AT3G5152 Manuscript (Kuczynski; C.; McCorkle; S.; Keereetaweep; J.; Shanklin; J.; & Schwender; J. (2022). An expanded role for the transcription factor WRINKLED1 in the biosynthesis of triacylglycerols during seed development. Frontiers in Plant Scienc | Sobic.010G13440 | yes | 58.73 | 49.19 | OTHER | 0.9994 OTHER 1.0000 | ortholo | yes | 202.8 |
| glycerolipid synthes | LPAAT | Lysophosphatidic Acid Acyltransferas | AT3G5765 Manuscript (Kuczynski; C.; McCorkle; S.; Keereetaweep; J.; Shanklin; J.; & Schwender; J. (2022). An expanded role for the transcription factor WRINKLED1 in the biosynthesis of triacylglycerols during seed development. Frontiers in Plant Scienc | Sobic.005G19710 | bidirectiona | 67.14 | 66.49 | OTHER | 0.6728 OTHER 0.6431 | LDO | no | 1731.7 |
| glycerolipid synthes | LPAAT | Lysophosphatidic Acid Acyltransferas | AT3G5765 Manuscript (Kuczynski; C.; McCorkle; S.; Keereetaweep; J.; Shanklin; J.; & Schwender; J. (2022). An expanded role for the transcription factor WRINKLED1 in the biosynthesis of triacylglycerols during seed development. Frontiers in Plant Scienc | Sobic.008G11210 | yes | 68.27 | 67.29 | OTHER | 0.6728 OTHER 0.8623 | ortholo | no | 4.0 |
| glycerolipid synthes | PP | phosphate phosphatase | AT3G5849 Manuscript (Kuczynski; C.; McCorkle; S.; Keereetaweep; J.; Shanklin; J.; & Schwender; J. (2022). An expanded role for the transcription factor WRINKLED1 in the biosynthesis of triacylglycerols during seed development. Frontiers in Plant Scienc | Sobic.001G04930 | bidirectiona | 60.55 | 59.61 | OTHER | 0.9952 OTHER 0.9487 | LDO | no | 1169.1 |
| glycerolipid synthes | GPAT | glycerol-3-phosphate acyltransferase 1 (GPAT1 | AT4G0040 Manuscript (Kuczynski; C.; McCorkle; S.; Keereetaweep; J.; Shanklin; J.; & Schwender; J. (2022). An expanded role for the transcription factor WRINKLED1 in the biosynthesis of triacylglycerols during seed development. Frontiers in Plant Scienc | Sobic.003G36070 | yes | 59.48 | 59.43 | OTHER | 1.0000 OTHER 0.9514 | no | no | 166.3 |
| glycerolipid synthes | GPAT | glycerol-3-phosphate acyltransferase 1 (GPAT1 | AT4G0040 Manuscript (Kuczynski; C.; McCorkle; S.; Keereetaweep; J.; Shanklin; J.; & Schwender; J. (2022). An expanded role for the transcription factor WRINKLED1 in the biosynthesis of triacylglycerols during seed development. Frontiers in Plant Scienc | Sobic.004G01030 | no | 55.95 | 54.82 | OTHER | 1.0000 OTHER 0.9146 | LDO | no | 452.6 |
| glycerolipid synthes | GPAT | glycerol-3-phosphate acyltransferase 1 (GPAT1 | AT4G0195 Manuscript (Kuczynski; C.; McCorkle; S.; Keereetaweep; J.; Shanklin; J.; & Schwender; J. (2022). An expanded role for the transcription factor WRINKLED1 in the biosynthesis of triacylglycerols during seed development. Frontiers in Plant Scienc | Sobic.008G13080 | yes | 43.91 | 44.16 | OTHER | 0.8682 cTP 0.6869 | ortholo | no | 83.5 |
| glycerolipid synthes | GPAT | glycerol-3-phosphate acyltransferase 1 (GPAT1 | AT4G0195 Manuscript (Kuczynski; C.; McCorkle; S.; Keereetaweep; J.; Shanklin; J.; & Schwender; J. (2022). An expanded role for the transcription factor WRINKLED1 in the biosynthesis of triacylglycerols during seed development. Frontiers in Plant Scienc | Sobic.001G02610 | no | 47.63 | 46.91 | OTHER | 0.8682 cTP 0.0510 | ortholo | no | nd |
| glycerolipid synthes | GPAT | glycerol-3-phosphate acyltransferase 1 (GPAT1 | AT4G0195 Manuscript (Kuczynski; C.; McCorkle; S.; Keereetaweep; J.; Shanklin; J.; & Schwender; J. (2022). An expanded role for the transcription factor WRINKLED1 in the biosynthesis of triacylglycerols during seed development. Frontiers in Plant Scienc | Sobic.003G11420 | yes | 43.84 | 42.52 | OTHER | 0.8682 cTP 0.3941 | ortholo | no | 191.6 |
| glycerolipid synthes | GPAT | glycerol-3-phosphate acyltransferase 1 (GPAT1 | AT4G0195 Manuscript (Kuczynski; C.; McCorkle; S.; Keereetaweep; J.; Shanklin; J.; & Schwender; J. (2022). An expanded role for the transcription factor WRINKLED1 in the biosynthesis of triacylglycerols during seed development. Frontiers in Plant Scienc | Sobic.003G14250 | bidirectiona | 42.7 | 42.4 | OTHER | 0.8682 OTHER 0.8184 | ortholo | no | nd |
| glycerolipid synthes | GPAT | glycerol-3-phosphate acyltransferase 1 (GPAT1 | AT4G0195 Manuscript (Kuczynski; C.; McCorkle; S.; Keereetaweep; J.; Shanklin; J.; & Schwender; J. (2022). An expanded role for the transcription factor WRINKLED1 in the biosynthesis of triacylglycerols during seed development. Frontiers in Plant Scienc | Sobic.005G21440 | yes | 47.57 | 47.62 | OTHER | 0.8682 OTHER 0.5791 | ortholo | no | 487.0 |
| glycerolipid synthes | PLA | Phospholipase A | AT4G1986 Manuscript (Kuczynski; C.; McCorkle; S.; Keereetaweep; J.; Shanklin; J.; & Schwender; J. (2022). An expanded role for the transcription factor WRINKLED1 in the biosynthesis of triacylglycerols during seed development. Frontiers in Plant Scienc | Sobic.002G26230 | no | 51.49 | 50.85 | OTHER | 0.9961 OTHER 0.9976 | no | no | 1152.9 |
| glycerolipid synthes | PLA | Phospholipase A | AT4G1986 Manuscript (Kuczynski; C.; McCorkle; S.; Keereetaweep; J.; Shanklin; J.; & Schwender; J. (2022). An expanded role for the transcription factor WRINKLED1 in the biosynthesis of triacylglycerols during seed development. Frontiers in Plant Scienc | Sobic.006G21450 | bidirectiona | 59.29 | 59.55 | OTHER | 0.9961 OTHER 0.9993 | LDO | no | 1586.0 |
| glycerolipid synthes | PP | phosphate phosphatase | AT4G2255 Manuscript (Kuczynski; C.; McCorkle; S.; Keereetaweep; J.; Shanklin; J.; & Schwender; J. (2022). An expanded role for the transcription factor WRINKLED1 in the biosynthesis of triacylglycerols during seed development. Frontiers in Plant Scienc | Sobic.004G29310 | bidirectiona | 41.55 | 42.93 | OTHER | 0.9922 OTHER 0.7027 | LDO | no | 502.3 |
| glycerolipid synthes | PLA | Phospholipase A | AT4G2907 Manuscript (Kuczynski; C.; McCorkle; S.; Keereetaweep; J.; Shanklin; J.; & Schwender; J. (2022). An expanded role for the transcription factor WRINKLED1 in the biosynthesis of triacylglycerols during seed development. Frontiers in Plant Scienc | Sobic.002G40190 | bidirectiona | 65.65 | 44.18 | OTHER | 0.9683 OTHER 0.9730 | LDO | no | 5799.1 |
| glycerolipid synthes | PLA | Phospholipase A | AT4G2946 Manuscript (Kuczynski; C.; McCorkle; S.; Keereetaweep; J.; Shanklin; J.; & Schwender; J. (2022). An expanded role for the transcription factor WRINKLED1 in the biosynthesis of triacylglycerols during seed development. Frontiers in Plant Scienc | Sobic.004G35780 | yes | 45.8 | 46.56 | SP | 1.0000 SP 1.0000 | ortholo | no | 119.5 |
| glycerolipid synthes | LPAAT | Lysophosphatidic Acid Acyltransferas | AT4G3058 Manuscript (Kuczynski; C.; McCorkle; S.; Keereetaweep; J.; Shanklin; J.; & Schwender; J. (2022). An expanded role for the transcription factor WRINKLED1 in the biosynthesis of triacylglycerols during seed development. Frontiers in Plant Scienc | Sobic.001G20600 | bidirectiona | 57.39 | 55.38 | cTP | 0.5503 cTP 0.6995 | LDO | no | 858.1 |
| glycerolipid synthes | PP | phosphate phosphatase | AT5G0308 Manuscript (Kuczynski; C.; McCorkle; S.; Keereetaweep; J.; Shanklin; J.; & Schwender; J. (2022). An expanded role for the transcription factor WRINKLED1 in the biosynthesis of triacylglycerols during seed development. Frontiers in Plant Scienc | Sobic.001G40840 | bidirectiona | 68.49 | 69.12 | OTHER | 0.9996 OTHER 1.0000 | LDO | no | 930.4 |
| glycerolipid synthes | GPAT | glycerol-3-phosphate acyltransferase 1 (GPAT1 | AT5G0609 Manuscript (Kuczynski; C.; McCorkle; S.; Keereetaweep; J.; Shanklin; J.; & Schwender; J. (2022). An expanded role for the transcription factor WRINKLED1 in the biosynthesis of triacylglycerols during seed development. Frontiers in Plant Scienc | Sobic.009G16200 | no | 60.37 | 58.3 | OTHER | 0.9999 OTHER 0.9888 | LDO | no | 1752.0 |
| glycerolipid synthes | DGAT3 | Diacylglycerol Acyltransferas | AT5G1364 Manuscript (Kuczynski; C.; McCorkle; S.; Keereetaweep; J.; Shanklin; J.; & Schwender; J. (2022). An expanded role for the transcription factor WRINKLED1 in the biosynthesis of triacylglycerols during seed development. Frontiers in Plant Scienc | Sobic.002G21850 | bidirectiona | 77.18 | 75.07 | OTHER | 0.9948 OTHER 0.9998 | LDO | no | 2825.0 |
| glycerolipid synthes | PP | phosphate phosphatase | AT5G4287 Manuscript (Kuczynski; C.; McCorkle; S.; Keereetaweep; J.; Shanklin; J.; & Schwender; J. (2022). An expanded role for the transcription factor WRINKLED1 in the biosynthesis of triacylglycerols during seed development. Frontiers in Plant Scienc | Sobic.005G18730 | bidirectiona | 61.07 | 49.45 | OTHER | 0.9374 OTHER 0.6765 | LDO | no | 2001.4 |
| glycerolipid synthes | GPAT | glycerol-3-phosphate acyltransferase 1 (GPAT1 | AT5G6062 Manuscript (Kuczynski; C.; McCorkle; S.; Keereetaweep; J.; Shanklin; J.; & Schwender; J. (2022). An expanded role for the transcription factor WRINKLED1 in the biosynthesis of triacylglycerols during seed development. Frontiers in Plant Scienc | Sobic.002G32530 | bidirectiona | 78.57 | 77.09 | OTHER | 0.9997 OTHER 0.9999 | LDO | no | 2239.4 |
| glycerolipid synthes | PP | phosphate phosphatase | AT5G6645 Manuscript (Kuczynski; C.; McCorkle; S.; Keereetaweep; J.; Shanklin; J.; & Schwender; J. (2022). An expanded role for the transcription factor WRINKLED1 in the biosynthesis of triacylglycerols during seed development. Frontiers in Plant Scienc | Sobic.004G26830 | bidirectiona | 39.58 | 36.43 | cTP | 0.8465 cTP 0.9069 | LDO | no | 1000.4 |
| glycolysi | FRK5 | fructokinase | AT1G0602 Manuscript (Kuczynski; C.; McCorkle; S.; Keereetaweep; J.; Shanklin; J.; & Schwender; J. (2022). An expanded role for the transcription factor WRINKLED1 in the biosynthesis of triacylglycerols during seed development. Frontiers in Plant Scienc | Sobic.003G38600 | yes | 74.92 | 73.07 | OTHER | 0.9985 OTHER 0.9966 | ortholo | no | 5605.6 |
| glycolysi | FRK6 | fructokinase | AT1G0603 Manuscript (Kuczynski; C.; McCorkle; S.; Keereetaweep; J.; Shanklin; J.; & Schwender; J. (2022). An expanded role for the transcription factor WRINKLED1 in the biosynthesis of triacylglycerols during seed development. Frontiers in Plant Scienc | Sobic.003G38600 | no | 74.6 | 72.76 | OTHER | 0.9999 OTHER 0.9966 | ortholo | no | 5605.6 |
| glycolysi | PGLM | phosphoglyceromutas | AT1G0978 Manuscript (Kuczynski; C.; McCorkle; S.; Keereetaweep; J.; Shanklin; J.; & Schwender; J. (2022). An expanded role for the transcription factor WRINKLED1 in the biosynthesis of triacylglycerols during seed development. Frontiers in Plant Scienc | Sobic.001G38410 | yes | 79.17 | 79.17 | OTHER | 0.9991 OTHER 0.9971 | no | no | 6.9 |
| glycolysi | PGLM | phosphoglyceromutas | AT1G0978 Manuscript (Kuczynski; C.; McCorkle; S.; Keereetaweep; J.; Shanklin; J.; & Schwender; J. (2022). An expanded role for the transcription factor WRINKLED1 in the biosynthesis of triacylglycerols during seed development. Frontiers in Plant Scienc | Sobic.003G33600 | yes | 83.87 | 83.48 | OTHER | 0.9991 OTHER 0.9903 | ortholo | no | 16765.6 |
| glycolysi | PFP-ß1 | Pyrophosphate dependent phospho-fructokinas | AT1G1200 Manuscript (Kuczynski; C.; McCorkle; S.; Keereetaweep; J.; Shanklin; J.; & Schwender; J. (2022). An expanded role for the transcription factor WRINKLED1 in the biosynthesis of triacylglycerols during seed development. Frontiers in Plant Scienc | Sobic.010G10160 | bidirectiona | 80.67 | 80.32 | OTHER | 0.8685 cTP 0.8850 | LDO | yes | 1452.6 |
| glycolysi | PFP-ß1 | Pyrophosphate dependent phospho-fructokinas | AT1G1200 Manuscript (Kuczynski; C.; McCorkle; S.; Keereetaweep; J.; Shanklin; J.; & Schwender; J. (2022). An expanded role for the transcription factor WRINKLED1 in the biosynthesis of triacylglycerols during seed development. Frontiers in Plant Scienc | Sobic.004G26180 | no | 40.07 | 39.26 | OTHER | 0.8685 OTHER 0.9995 | no | yes | 5.8 |
| glycolysi | PFP-ß1 | Pyrophosphate dependent phospho-fructokinas | AT1G1200 Manuscript (Kuczynski; C.; McCorkle; S.; Keereetaweep; J.; Shanklin; J.; & Schwender; J. (2022). An expanded role for the transcription factor WRINKLED1 in the biosynthesis of triacylglycerols during seed development. Frontiers in Plant Scienc | Sobic.010G13400 | no | 41.54 | 39.15 | OTHER | 0.8685 OTHER 0.9941 | no | yes | 1220.3 |
| glycolysi | PFP-ß1 | Pyrophosphate dependent phospho-fructokinas | AT1G1200 Manuscript (Kuczynski; C.; McCorkle; S.; Keereetaweep; J.; Shanklin; J.; & Schwender; J. (2022). An expanded role for the transcription factor WRINKLED1 in the biosynthesis of triacylglycerols during seed development. Frontiers in Plant Scienc | Sobic.002G16030 | no | 41.23 | 38.21 | OTHER | 0.8685 OTHER 0.9968 | no | yes | 364.7 |
| glycolysi | PFP-ß1 | Pyrophosphate dependent phospho-fructokinas | AT1G1200 Manuscript (Kuczynski; C.; McCorkle; S.; Keereetaweep; J.; Shanklin; J.; & Schwender; J. (2022). An expanded role for the transcription factor WRINKLED1 in the biosynthesis of triacylglycerols during seed development. Frontiers in Plant Scienc | Sobic.002G02630 | no | 41.03 | 40.85 | OTHER | 0.8685 OTHER 0.9901 | no | yes | 2858.1 |
| glycolysi | PFP-ß1 | Pyrophosphate dependent phospho-fructokinas | AT1G1200 Manuscript (Kuczynski; C.; McCorkle; S.; Keereetaweep; J.; Shanklin; J.; & Schwender; J. (2022). An expanded role for the transcription factor WRINKLED1 in the biosynthesis of triacylglycerols during seed development. Frontiers in Plant Scienc | Sobic.007G10180 | no | 42.56 | 42.26 | OTHER | 0.8685 OTHER 0.9991 | no | yes | 248.3 |
| glycolysi | TA | transaldolase | AT1G1223 Manuscript (Kuczynski; C.; McCorkle; S.; Keereetaweep; J.; Shanklin; J.; & Schwender; J. (2022). An expanded role for the transcription factor WRINKLED1 in the biosynthesis of triacylglycerols during seed development. Frontiers in Plant Scienc | Sobic.007G04340 | bidirectiona | 62.15 | 65.74 | cTP | 0.9444 cTP 0.9982 | LDO | no | 2762.2 |
| glycolysi | GAPDH | Glyceraldehyde 3-phosphate dehydrogenas | AT1G1290 Manuscript (Kuczynski; C.; McCorkle; S.; Keereetaweep; J.; Shanklin; J.; & Schwender; J. (2022). An expanded role for the transcription factor WRINKLED1 in the biosynthesis of triacylglycerols during seed development. Frontiers in Plant Scienc | Sobic.004G20510 | no | 48.64 | 48.65 | cTP | 0.9837 OTHER 0.9225 | no | no | 3691.5 |
| glycolysi | GAPDH | Glyceraldehyde 3-phosphate dehydrogenas | AT1G1290 Manuscript (Kuczynski; C.; McCorkle; S.; Keereetaweep; J.; Shanklin; J.; & Schwender; J. (2022). An expanded role for the transcription factor WRINKLED1 in the biosynthesis of triacylglycerols during seed development. Frontiers in Plant Scienc | Sobic.009G01670 | no | 48.64 | 48.05 | cTP | 0.9837 OTHER 0.6901 | no | no | nd |
| glycolysi | GAPDH | Glyceraldehyde 3-phosphate dehydrogenas | AT1G1290 Manuscript (Kuczynski; C.; McCorkle; S.; Keereetaweep; J.; Shanklin; J.; & Schwender; J. (2022). An expanded role for the transcription factor WRINKLED1 in the biosynthesis of triacylglycerols during seed development. Frontiers in Plant Scienc | Sobic.010G26243 | no | 48.64 | 41.04 | cTP | 0.9837 OTHER 0.9970 | no | no | 1583.8 |
| glycolysi | GAPDH | Glyceraldehyde 3-phosphate dehydrogenas | AT1G1290 Manuscript (Kuczynski; C.; McCorkle; S.; Keereetaweep; J.; Shanklin; J.; & Schwender; J. (2022). An expanded role for the transcription factor WRINKLED1 in the biosynthesis of triacylglycerols during seed development. Frontiers in Plant Scienc | Sobic.010G26246 | no | 46.45 | 44.29 | cTP | 0.9837 OTHER 0.9644 | no | no | 6345.2 |
| glycolysi | GAPDH | Glyceraldehyde 3-phosphate dehydrogenas | AT1G1290 Manuscript (Kuczynski; C.; McCorkle; S.; Keereetaweep; J.; Shanklin; J.; & Schwender; J. (2022). An expanded role for the transcription factor WRINKLED1 in the biosynthesis of triacylglycerols during seed development. Frontiers in Plant Scienc | Sobic.007G02540 | no | 47.58 | 47.15 | cTP | 0.9837 OTHER 0.9663 | no | no | 12281.7 |
| glycolysi | GAPDH | Glyceraldehyde 3-phosphate dehydrogenas | AT1G1290 Manuscript (Kuczynski; C.; McCorkle; S.; Keereetaweep; J.; Shanklin; J.; & Schwender; J. (2022). An expanded role for the transcription factor WRINKLED1 in the biosynthesis of triacylglycerols during seed development. Frontiers in Plant Scienc | Sobic.010G26250 | no | 47.88 | 47.15 | cTP | 0.9837 OTHER 0.9649 | no | no | 4632.5 |
| glycolysi | GAPDH | Glyceraldehyde 3-phosphate dehydrogenas | AT1G1290 Manuscript (Kuczynski; C.; McCorkle; S.; Keereetaweep; J.; Shanklin; J.; & Schwender; J. (2022). An expanded role for the transcription factor WRINKLED1 in the biosynthesis of triacylglycerols during seed development. Frontiers in Plant Scienc | Sobic.004G05640 | no | 48.94 | 43.57 | cTP | 0.9837 cTP 0.9413 | no | no | 819.3 |
| glycolysi | GAPDH | Glyceraldehyde 3-phosphate dehydrogenas | AT1G1290 Manuscript (Kuczynski; C.; McCorkle; S.; Keereetaweep; J.; Shanklin; J.; & Schwender; J. (2022). An expanded role for the transcription factor WRINKLED1 in the biosynthesis of triacylglycerols during seed development. Frontiers in Plant Scienc | Sobic.006G10590 | bidirectiona | 83.92 | 82.91 | cTP | 0.9837 cTP 0.9921 | ortholo | no | 230502. |
| glycolysi | GAPDH | Glyceraldehyde 3-phosphate dehydrogenas | AT1G1290 Manuscript (Kuczynski; C.; McCorkle; S.; Keereetaweep; J.; Shanklin; J.; & Schwender; J. (2022). An expanded role for the transcription factor WRINKLED1 in the biosynthesis of triacylglycerols during seed development. Frontiers in Plant Scienc | Sobic.001G51980 | no | 74.67 | 73.59 | cTP | 0.9837 cTP 0.8801 | no | no | 236570. |
| glycolysi | GAPDH | Glyceraldehyde 3-phosphate dehydrogenas | AT1G1344 Manuscript (Kuczynski; C.; McCorkle; S.; Keereetaweep; J.; Shanklin; J.; & Schwender; J. (2022). An expanded role for the transcription factor WRINKLED1 in the biosynthesis of triacylglycerols during seed development. Frontiers in Plant Scienc | Sobic.010G10020 | no | 85.71 | 44.94 | OTHER | 0.9989 OTHER 0.9336 | no | no | 792.1 |
| glycolysi | GAPDH | Glyceraldehyde 3-phosphate dehydrogenas | AT1G1344 Manuscript (Kuczynski; C.; McCorkle; S.; Keereetaweep; J.; Shanklin; J.; & Schwender; J. (2022). An expanded role for the transcription factor WRINKLED1 in the biosynthesis of triacylglycerols during seed development. Frontiers in Plant Scienc | Sobic.004G20510 | bidirectiona | 87.35 | 86.31 | OTHER | 0.9989 OTHER 0.9225 | LDO | no | 3691.5 |
| glycolysi | GAPDH | Glyceraldehyde 3-phosphate dehydrogenas | AT1G1344 Manuscript (Kuczynski; C.; McCorkle; S.; Keereetaweep; J.; Shanklin; J.; & Schwender; J. (2022). An expanded role for the transcription factor WRINKLED1 in the biosynthesis of triacylglycerols during seed development. Frontiers in Plant Scienc | Sobic.009G01670 | yes | 86.1 | 84.82 | OTHER | 0.9989 OTHER 0.6901 | ortholo | no | nd |
| glycolysi | GAPDH | Glyceraldehyde 3-phosphate dehydrogenas | AT1G1344 Manuscript (Kuczynski; C.; McCorkle; S.; Keereetaweep; J.; Shanklin; J.; & Schwender; J. (2022). An expanded role for the transcription factor WRINKLED1 in the biosynthesis of triacylglycerols during seed development. Frontiers in Plant Scienc | Sobic.010G26243 | no | 81.51 | 73.33 | OTHER | 0.9989 OTHER 0.9970 | no | no | 1583.8 |
| glycolysi | GAPDH | Glyceraldehyde 3-phosphate dehydrogenas | AT1G1344 Manuscript (Kuczynski; C.; McCorkle; S.; Keereetaweep; J.; Shanklin; J.; & Schwender; J. (2022). An expanded role for the transcription factor WRINKLED1 in the biosynthesis of triacylglycerols during seed development. Frontiers in Plant Scienc | Sobic.010G26246 | no | 86.79 | 83.78 | OTHER | 0.9989 OTHER 0.9644 | no | no | 6345.2 |
| glycolysi | GAPDH | Glyceraldehyde 3-phosphate dehydrogenas | AT1G1344 Manuscript (Kuczynski; C.; McCorkle; S.; Keereetaweep; J.; Shanklin; J.; & Schwender; J. (2022). An expanded role for the transcription factor WRINKLED1 in the biosynthesis of triacylglycerols during seed development. Frontiers in Plant Scienc | Sobic.007G02540 | no | 86.49 | 85.71 | OTHER | 0.9989 OTHER 0.9663 | no | no | 12281.7 |
| glycolysi | GAPDH | Glyceraldehyde 3-phosphate dehydrogenas | AT1G1344 Manuscript (Kuczynski; C.; McCorkle; S.; Keereetaweep; J.; Shanklin; J.; & Schwender; J. (2022). An expanded role for the transcription factor WRINKLED1 in the biosynthesis of triacylglycerols during seed development. Frontiers in Plant Scienc | Sobic.010G26250 | no | 85.89 | 85.12 | OTHER | 0.9989 OTHER 0.9649 | no | no | 4632.5 |
| glycolysi | GAPDH | Glyceraldehyde 3-phosphate dehydrogenas | AT1G1344 Manuscript (Kuczynski; C.; McCorkle; S.; Keereetaweep; J.; Shanklin; J.; & Schwender; J. (2022). An expanded role for the transcription factor WRINKLED1 in the biosynthesis of triacylglycerols during seed development. Frontiers in Plant Scienc | Sobic.004G05640 | no | 72.97 | 72.11 | OTHER | 0.9989 cTP 0.9413 | no | no | 819.3 |
| glycolysi | GAPDH | Glyceraldehyde 3-phosphate dehydrogenas | AT1G1344 Manuscript (Kuczynski; C.; McCorkle; S.; Keereetaweep; J.; Shanklin; J.; & Schwender; J. (2022). An expanded role for the transcription factor WRINKLED1 in the biosynthesis of triacylglycerols during seed development. Frontiers in Plant Scienc | Sobic.006G10590 | no | 47.45 | 47.01 | OTHER | 0.9989 cTP 0.9921 | no | no | 230502. |
| glycolysi | GAPDH | Glyceraldehyde 3-phosphate dehydrogenas | AT1G1344 Manuscript (Kuczynski; C.; McCorkle; S.; Keereetaweep; J.; Shanklin; J.; & Schwender; J. (2022). An expanded role for the transcription factor WRINKLED1 in the biosynthesis of triacylglycerols during seed development. Frontiers in Plant Scienc | Sobic.001G51980 | no | 48.21 | 48.36 | OTHER | 0.9989 cTP 0.8801 | no | no | 236570. |
| glycolysi | GAPDH | Glyceraldehyde 3-phosphate dehydrogenas | AT1G1630 Manuscript (Kuczynski; C.; McCorkle; S.; Keereetaweep; J.; Shanklin; J.; & Schwender; J. (2022). An expanded role for the transcription factor WRINKLED1 in the biosynthesis of triacylglycerols during seed development. Frontiers in Plant Scienc | Sobic.010G10020 | no | 80 | 43.82 | cTP | 0.7774 OTHER 0.9336 | no | no | 792.1 |
| glycolysi | GAPDH | Glyceraldehyde 3-phosphate dehydrogenas | AT1G1630 Manuscript (Kuczynski; C.; McCorkle; S.; Keereetaweep; J.; Shanklin; J.; & Schwender; J. (2022). An expanded role for the transcription factor WRINKLED1 in the biosynthesis of triacylglycerols during seed development. Frontiers in Plant Scienc | Sobic.004G20510 | no | 71.69 | 70.83 | cTP | 0.7774 OTHER 0.9225 | no | no | 3691.5 |
| glycolysi | GAPDH | Glyceraldehyde 3-phosphate dehydrogenas | AT1G1630 Manuscript (Kuczynski; C.; McCorkle; S.; Keereetaweep; J.; Shanklin; J.; & Schwender; J. (2022). An expanded role for the transcription factor WRINKLED1 in the biosynthesis of triacylglycerols during seed development. Frontiers in Plant Scienc | Sobic.009G01670 | no | 71.9 | 70.83 | cTP | 0.7774 OTHER 0.6901 | no | no | nd |
| glycolysi | GAPDH | Glyceraldehyde 3-phosphate dehydrogenas | AT1G1630 Manuscript (Kuczynski; C.; McCorkle; S.; Keereetaweep; J.; Shanklin; J.; & Schwender; J. (2022). An expanded role for the transcription factor WRINKLED1 in the biosynthesis of triacylglycerols during seed development. Frontiers in Plant Scienc | Sobic.010G26243 | no | 77.78 | 68.38 | cTP | 0.7774 OTHER 0.9970 | no | no | 1583.8 |
| glycolysi | GAPDH | Glyceraldehyde 3-phosphate dehydrogenas | AT1G1630 Manuscript (Kuczynski; C.; McCorkle; S.; Keereetaweep; J.; Shanklin; J.; & Schwender; J. (2022). An expanded role for the transcription factor WRINKLED1 in the biosynthesis of triacylglycerols during seed development. Frontiers in Plant Scienc | Sobic.010G26246 | no | 68.87 | 66.52 | cTP | 0.7774 OTHER 0.9644 | no | no | 6345.2 |
| glycolysi | GAPDH | Glyceraldehyde 3-phosphate dehydrogenas | AT1G1630 Manuscript (Kuczynski; C.; McCorkle; S.; Keereetaweep; J.; Shanklin; J.; & Schwender; J. (2022). An expanded role for the transcription factor WRINKLED1 in the biosynthesis of triacylglycerols during seed development. Frontiers in Plant Scienc | Sobic.007G02540 | no | 73.19 | 72.32 | cTP | 0.7774 OTHER 0.9663 | no | no | 12281.7 |
| glycolysi | GAPDH | Glyceraldehyde 3-phosphate dehydrogenas | AT1G1630 Manuscript (Kuczynski; C.; McCorkle; S.; Keereetaweep; J.; Shanklin; J.; & Schwender; J. (2022). An expanded role for the transcription factor WRINKLED1 in the biosynthesis of triacylglycerols during seed development. Frontiers in Plant Scienc | Sobic.010G26250 | no | 73.19 | 72.32 | cTP | 0.7774 OTHER 0.9649 | no | no | 4632.5 |
| glycolysi | GAPDH | Glyceraldehyde 3-phosphate dehydrogenas | AT1G1630 Manuscript (Kuczynski; C.; McCorkle; S.; Keereetaweep; J.; Shanklin; J.; & Schwender; J. (2022). An expanded role for the transcription factor WRINKLED1 in the biosynthesis of triacylglycerols during seed development. Frontiers in Plant Scienc | Sobic.004G05640 | yes | 84.57 | 78 | cTP | 0.7774 cTP 0.9413 | ortholo | no | 819.3 |
| glycolysi | GAPDH | Glyceraldehyde 3-phosphate dehydrogenas | AT1G1630 Manuscript (Kuczynski; C.; McCorkle; S.; Keereetaweep; J.; Shanklin; J.; & Schwender; J. (2022). An expanded role for the transcription factor WRINKLED1 in the biosynthesis of triacylglycerols during seed development. Frontiers in Plant Scienc | Sobic.006G10590 | no | 46.94 | 43.39 | cTP | 0.7774 cTP 0.9921 | no | no | 230502. |
| glycolysi | GAPDH | Glyceraldehyde 3-phosphate dehydrogenas | AT1G1630 Manuscript (Kuczynski; C.; McCorkle; S.; Keereetaweep; J.; Shanklin; J.; & Schwender; J. (2022). An expanded role for the transcription factor WRINKLED1 in the biosynthesis of triacylglycerols during seed development. Frontiers in Plant Scienc | Sobic.001G51980 | no | 50 | 44.39 | cTP | 0.7774 cTP 0.8801 | no | no | 236570. |
| glycolysi | PFP-? | Pyrophosphate dependent phospho-fructokinas | AT1G2095 Manuscript (Kuczynski; C.; McCorkle; S.; Keereetaweep; J.; Shanklin; J.; & Schwender; J. (2022). An expanded role for the transcription factor WRINKLED1 in the biosynthesis of triacylglycerols during seed development. Frontiers in Plant Scienc | Sobic.004G26180 | yes | 78.86 | 78.76 | OTHER | 0.9994 OTHER 0.9995 | LDO | no | 5.8 |
| glycolysi | PFP-? | Pyrophosphate dependent phospho-fructokinas | AT1G2095 Manuscript (Kuczynski; C.; McCorkle; S.; Keereetaweep; J.; Shanklin; J.; & Schwender; J. (2022). An expanded role for the transcription factor WRINKLED1 in the biosynthesis of triacylglycerols during seed development. Frontiers in Plant Scienc | Sobic.010G13400 | no | 77.54 | 77.41 | OTHER | 0.9994 OTHER 0.9941 | ortholo | no | 1220.3 |
| glycolysi | PFP-? | Pyrophosphate dependent phospho-fructokinas | AT1G2095 Manuscript (Kuczynski; C.; McCorkle; S.; Keereetaweep; J.; Shanklin; J.; & Schwender; J. (2022). An expanded role for the transcription factor WRINKLED1 in the biosynthesis of triacylglycerols during seed development. Frontiers in Plant Scienc | Sobic.002G16030 | no | 62.24 | 62.75 | OTHER | 0.9994 OTHER 0.9968 | ortholo | no | 364.7 |
| glycolysi | PFP-? | Pyrophosphate dependent phospho-fructokinas | AT1G2095 Manuscript (Kuczynski; C.; McCorkle; S.; Keereetaweep; J.; Shanklin; J.; & Schwender; J. (2022). An expanded role for the transcription factor WRINKLED1 in the biosynthesis of triacylglycerols during seed development. Frontiers in Plant Scienc | Sobic.002G02630 | no | 63.77 | 63.82 | OTHER | 0.9994 OTHER 0.9901 | ortholo | no | 2858.1 |
| glycolysi | PFP-? | Pyrophosphate dependent phospho-fructokinas | AT1G2095 Manuscript (Kuczynski; C.; McCorkle; S.; Keereetaweep; J.; Shanklin; J.; & Schwender; J. (2022). An expanded role for the transcription factor WRINKLED1 in the biosynthesis of triacylglycerols during seed development. Frontiers in Plant Scienc | Sobic.007G10180 | no | 61.3 | 65.31 | OTHER | 0.9994 OTHER 0.9991 | ortholo | no | 248.3 |
| glycolysi | PGLM | phosphoglyceromutas | AT1G2217 Manuscript (Kuczynski; C.; McCorkle; S.; Keereetaweep; J.; Shanklin; J.; & Schwender; J. (2022). An expanded role for the transcription factor WRINKLED1 in the biosynthesis of triacylglycerols during seed development. Frontiers in Plant Scienc | Sobic.004G23410 | bidirectiona | 87.01 | 72.05 | cTP | 0.9946 cTP 0.9970 | LDO | yes | 1564.1 |
| glycolysi | PGLM | phosphoglyceromutas | AT1G2217 Manuscript (Kuczynski; C.; McCorkle; S.; Keereetaweep; J.; Shanklin; J.; & Schwender; J. (2022). An expanded role for the transcription factor WRINKLED1 in the biosynthesis of triacylglycerols during seed development. Frontiers in Plant Scienc | Sobic.010G09010 | yes | 81.44 | 69.02 | cTP | 0.9946 cTP 0.9596 | ortholo | yes | 6.0 |
| glycolysi | PGLM | phosphoglyceromutas | AT1G2319 Manuscript (Kuczynski; C.; McCorkle; S.; Keereetaweep; J.; Shanklin; J.; & Schwender; J. (2022). An expanded role for the transcription factor WRINKLED1 in the biosynthesis of triacylglycerols during seed development. Frontiers in Plant Scienc | Sobic.001G11650 | yes | 83.36 | 83.48 | OTHER | 0.9577 OTHER 0.9577 | LDO | no | 11791.8 |
| glycolysi | PGLM | phosphoglyceromutas | AT1G2319 Manuscript (Kuczynski; C.; McCorkle; S.; Keereetaweep; J.; Shanklin; J.; & Schwender; J. (2022). An expanded role for the transcription factor WRINKLED1 in the biosynthesis of triacylglycerols during seed development. Frontiers in Plant Scienc | Sobic.003G22250 | no | 61.06 | 62.97 | OTHER | 0.9577 OTHER 0.9577 | no | no | 4204.7 |
| glycolysi | PK | pyruvate kinase | AT1G3244 Manuscript (Kuczynski; C.; McCorkle; S.; Keereetaweep; J.; Shanklin; J.; & Schwender; J. (2022). An expanded role for the transcription factor WRINKLED1 in the biosynthesis of triacylglycerols during seed development. Frontiers in Plant Scienc | Sobic.001G29060 | no | 76.29 | 69.89 | cTP | 0.8851 cTP 0.9514 | LDO | no | 12978.9 |
| glycolysi | PK | pyruvate kinase | AT1G3244 Manuscript (Kuczynski; C.; McCorkle; S.; Keereetaweep; J.; Shanklin; J.; & Schwender; J. (2022). An expanded role for the transcription factor WRINKLED1 in the biosynthesis of triacylglycerols during seed development. Frontiers in Plant Scienc | Sobic.003G24470 | yes | 77.75 | 69.96 | cTP | 0.8851 cTP 0.9844 | no | no | 433.7 |
| glycolysi | PK | pyruvate kinase | AT1G3244 Manuscript (Kuczynski; C.; McCorkle; S.; Keereetaweep; J.; Shanklin; J.; & Schwender; J. (2022). An expanded role for the transcription factor WRINKLED1 in the biosynthesis of triacylglycerols during seed development. Frontiers in Plant Scienc | Sobic.006G26720 | no | 36.81 | 41.76 | cTP | 0.8851 OTHER 0.9985 | no | no | 1530.2 |
| glycolysi | PK | pyruvate kinase | AT1G3244 Manuscript (Kuczynski; C.; McCorkle; S.; Keereetaweep; J.; Shanklin; J.; & Schwender; J. (2022). An expanded role for the transcription factor WRINKLED1 in the biosynthesis of triacylglycerols during seed development. Frontiers in Plant Scienc | Sobic.001G38690 | no | 36 | 40.47 | cTP | 0.8851 OTHER 0.9151 | no | no | nd |
| glycolysi | PK | pyruvate kinase | AT1G3244 Manuscript (Kuczynski; C.; McCorkle; S.; Keereetaweep; J.; Shanklin; J.; & Schwender; J. (2022). An expanded role for the transcription factor WRINKLED1 in the biosynthesis of triacylglycerols during seed development. Frontiers in Plant Scienc | Sobic.003G13080 | no | 40.44 | 40.51 | cTP | 0.8851 OTHER 0.9974 | no | no | 5456.7 |
| glycolysi | PK | pyruvate kinase | AT1G3244 Manuscript (Kuczynski; C.; McCorkle; S.; Keereetaweep; J.; Shanklin; J.; & Schwender; J. (2022). An expanded role for the transcription factor WRINKLED1 in the biosynthesis of triacylglycerols during seed development. Frontiers in Plant Scienc | Sobic.005G10140 | no | 31.03 | 62.93 | cTP | 0.8851 OTHER 0.9247 | no | no | 477.0 |
| glycolysi | PK | pyruvate kinase | AT1G3244 Manuscript (Kuczynski; C.; McCorkle; S.; Keereetaweep; J.; Shanklin; J.; & Schwender; J. (2022). An expanded role for the transcription factor WRINKLED1 in the biosynthesis of triacylglycerols during seed development. Frontiers in Plant Scienc | Sobic.005G03440 | no | 29.25 | 86.15 | cTP | 0.8851 OTHER 0.9510 | no | no | 3031.3 |
| glycolysi | PK | pyruvate kinase | AT1G3244 Manuscript (Kuczynski; C.; McCorkle; S.; Keereetaweep; J.; Shanklin; J.; & Schwender; J. (2022). An expanded role for the transcription factor WRINKLED1 in the biosynthesis of triacylglycerols during seed development. Frontiers in Plant Scienc | Sobic.008G03360 | no | 29.53 | 86.34 | cTP | 0.8851 OTHER 0.9373 | no | no | 4890.7 |
| glycolysi | GAPDH | Glyceraldehyde 3-phosphate dehydrogenas | AT1G4297 Manuscript (Kuczynski; C.; McCorkle; S.; Keereetaweep; J.; Shanklin; J.; & Schwender; J. (2022). An expanded role for the transcription factor WRINKLED1 in the biosynthesis of triacylglycerols during seed development. Frontiers in Plant Scienc | Sobic.004G20510 | no | 47.51 | 48.5 | cTP | 0.7302 OTHER 0.9225 | no | no | 3691.5 |

| glycolysi | GAPDH | Glyceraldehyde 3-phosphate dehydrogenas | AT1G4297 Manuscript (Kuczynski; C.; McCorkle; S.; Keereetaweep; J.; Shanklin; J.; & Schwender; J. (2022). An expanded role for the transcription factor WRINKLED1 in the biosynthesis of triacylglycerols during seed development. Frontiers in Plant Scienc | Sobic.009G01670 | no | 47.46 | 47.6 | cTP | 0.7302 OTHER 0.6901 | no | no nd | |
| --- | --- | --- | --- | --- | --- | --- | --- | --- | --- | --- | --- | --- |
| glycolysi | GAPDH | Glyceraldehyde 3-phosphate dehydrogenas | AT1G4297 Manuscript (Kuczynski; C.; McCorkle; S.; Keereetaweep; J.; Shanklin; J.; & Schwender; J. (2022). An expanded role for the transcription factor WRINKLED1 in the biosynthesis of triacylglycerols during seed development. Frontiers in Plant Scienc | Sobic.010G26243 | no | 45.9 | 40.74 | cTP | 0.7302 OTHER 0.9970 | no | no | 1583.8 |
| glycolysi | GAPDH | Glyceraldehyde 3-phosphate dehydrogenas | AT1G4297 Manuscript (Kuczynski; C.; McCorkle; S.; Keereetaweep; J.; Shanklin; J.; & Schwender; J. (2022). An expanded role for the transcription factor WRINKLED1 in the biosynthesis of triacylglycerols during seed development. Frontiers in Plant Scienc | Sobic.010G26246 | no | 46.01 | 44.75 | cTP | 0.7302 OTHER 0.9644 | no | no | 6345.2 |
| glycolysi | GAPDH | Glyceraldehyde 3-phosphate dehydrogenas | AT1G4297 Manuscript (Kuczynski; C.; McCorkle; S.; Keereetaweep; J.; Shanklin; J.; & Schwender; J. (2022). An expanded role for the transcription factor WRINKLED1 in the biosynthesis of triacylglycerols during seed development. Frontiers in Plant Scienc | Sobic.007G02540 | no | 47.46 | 47.01 | cTP | 0.7302 OTHER 0.9663 | no | no | 12281.7 |
| glycolysi | GAPDH | Glyceraldehyde 3-phosphate dehydrogenas | AT1G4297 Manuscript (Kuczynski; C.; McCorkle; S.; Keereetaweep; J.; Shanklin; J.; & Schwender; J. (2022). An expanded role for the transcription factor WRINKLED1 in the biosynthesis of triacylglycerols during seed development. Frontiers in Plant Scienc | Sobic.010G26250 | no | 47.16 | 47.01 | cTP | 0.7302 OTHER 0.9649 | no | no | 4632.5 |
| glycolysi | GAPDH | Glyceraldehyde 3-phosphate dehydrogenas | AT1G4297 Manuscript (Kuczynski; C.; McCorkle; S.; Keereetaweep; J.; Shanklin; J.; & Schwender; J. (2022). An expanded role for the transcription factor WRINKLED1 in the biosynthesis of triacylglycerols during seed development. Frontiers in Plant Scienc | Sobic.004G05640 | no | 47.38 | 42.54 | cTP | 0.7302 cTP 0.9413 | no | no | 819.3 |
| glycolysi | GAPDH | Glyceraldehyde 3-phosphate dehydrogenas | AT1G4297 Manuscript (Kuczynski; C.; McCorkle; S.; Keereetaweep; J.; Shanklin; J.; & Schwender; J. (2022). An expanded role for the transcription factor WRINKLED1 in the biosynthesis of triacylglycerols during seed development. Frontiers in Plant Scienc | Sobic.006G10590 | no | 74.29 | 70.98 | cTP | 0.7302 cTP 0.9921 | no | no | 230502. |
| glycolysi | GAPDH | Glyceraldehyde 3-phosphate dehydrogenas | AT1G4297 Manuscript (Kuczynski; C.; McCorkle; S.; Keereetaweep; J.; Shanklin; J.; & Schwender; J. (2022). An expanded role for the transcription factor WRINKLED1 in the biosynthesis of triacylglycerols during seed development. Frontiers in Plant Scienc | Sobic.001G51980 | bidirectiona | 80.31 | 82.88 | cTP | 0.7302 cTP 0.8801 | LDO | no | 236570. |
| glycolysi | FBA | fructose bisphosphate aldolas | AT1G4367 Manuscript (Kuczynski; C.; McCorkle; S.; Keereetaweep; J.; Shanklin; J.; & Schwender; J. (2022). An expanded role for the transcription factor WRINKLED1 in the biosynthesis of triacylglycerols during seed development. Frontiers in Plant Scienc | Sobic.003G36750 | bidirectiona | 85.34 | 85.34 | OTHER | 0.9998 OTHER 0.9997 | LDO | no | 26722.5 |
| glycolysi | FBA | fructose bisphosphate aldolas | AT1G4367 Manuscript (Kuczynski; C.; McCorkle; S.; Keereetaweep; J.; Shanklin; J.; & Schwender; J. (2022). An expanded role for the transcription factor WRINKLED1 in the biosynthesis of triacylglycerols during seed development. Frontiers in Plant Scienc | Sobic.009G15270 | yes | 84.57 | 83.58 | OTHER | 0.9998 OTHER 0.9996 | LDO | no | 2514.8 |
| glycolysi | PGK | Phosphoglycerokinas | AT1G5619 Manuscript (Kuczynski; C.; McCorkle; S.; Keereetaweep; J.; Shanklin; J.; & Schwender; J. (2022). An expanded role for the transcription factor WRINKLED1 in the biosynthesis of triacylglycerols during seed development. Frontiers in Plant Scienc | Sobic.009G18370 | bidirectiona | 83.15 | 80.25 | cTP | 0.9977 cTP 0.9990 | ortholo | no | 35283.3 |
| glycolysi | PGK | Phosphoglycerokinas | AT1G5619 Manuscript (Kuczynski; C.; McCorkle; S.; Keereetaweep; J.; Shanklin; J.; & Schwender; J. (2022). An expanded role for the transcription factor WRINKLED1 in the biosynthesis of triacylglycerols during seed development. Frontiers in Plant Scienc | Sobic.004G05520 | no | 84.21 | 83.79 | cTP | 0.9977 OTHER 0.9977 | no | no | 22589.2 |
| glycolysi | PGK | Phosphoglycerokinas | AT1G5619 Manuscript (Kuczynski; C.; McCorkle; S.; Keereetaweep; J.; Shanklin; J.; & Schwender; J. (2022). An expanded role for the transcription factor WRINKLED1 in the biosynthesis of triacylglycerols during seed development. Frontiers in Plant Scienc | Sobic.010G22180 | no | 82.66 | 82.04 | cTP | 0.9977 OTHER 0.9992 | no | no | 294.1 |
| glycolysi | FRK3 | fructokinase | AT1G6643 Manuscript (Kuczynski; C.; McCorkle; S.; Keereetaweep; J.; Shanklin; J.; & Schwender; J. (2022). An expanded role for the transcription factor WRINKLED1 in the biosynthesis of triacylglycerols during seed development. Frontiers in Plant Scienc | Sobic.010G09500 | bidirectiona | 78.47 | 72.75 | cTP | 0.9711 cTP 0.9994 | LDO | yes | 820.7 |
| glycolysi | PGLM | phosphoglyceromutas | AT1G7073 Manuscript (Kuczynski; C.; McCorkle; S.; Keereetaweep; J.; Shanklin; J.; & Schwender; J. (2022). An expanded role for the transcription factor WRINKLED1 in the biosynthesis of triacylglycerols during seed development. Frontiers in Plant Scienc | Sobic.001G11650 | bidirectiona | 83.53 | 77.01 | OTHER | 0.5307 OTHER 0.5307 | ortholo | no | 11791.8 |
| glycolysi | PGLM | phosphoglyceromutas | AT1G7073 Manuscript (Kuczynski; C.; McCorkle; S.; Keereetaweep; J.; Shanklin; J.; & Schwender; J. (2022). An expanded role for the transcription factor WRINKLED1 in the biosynthesis of triacylglycerols during seed development. Frontiers in Plant Scienc | Sobic.003G22250 | no | 59.59 | 57.24 | OTHER | 0.5307 OTHER 0.5307 | no | no | 4204.7 |
| glycolysi | Eno | Enolase | AT1G7403 Manuscript (Kuczynski; C.; McCorkle; S.; Keereetaweep; J.; Shanklin; J.; & Schwender; J. (2022). An expanded role for the transcription factor WRINKLED1 in the biosynthesis of triacylglycerols during seed development. Frontiers in Plant Scienc | Sobic.002G18690 | bidirectiona | 88.14 | 81.7 | cTP | 0.8920 cTP 0.9884 | LDO | yes | 510.4 |
| glycolysi | PFP-?2 | Pyrophosphate dependent phospho-fructokinas | AT1G7655 Manuscript (Kuczynski; C.; McCorkle; S.; Keereetaweep; J.; Shanklin; J.; & Schwender; J. (2022). An expanded role for the transcription factor WRINKLED1 in the biosynthesis of triacylglycerols during seed development. Frontiers in Plant Scienc | Sobic.004G26180 | bidirectiona | 80.46 | 79.87 | OTHER | 0.9991 OTHER 0.9995 | ortholo | yes | 5.8 |
| glycolysi | PFP-?2 | Pyrophosphate dependent phospho-fructokinas | AT1G7655 Manuscript (Kuczynski; C.; McCorkle; S.; Keereetaweep; J.; Shanklin; J.; & Schwender; J. (2022). An expanded role for the transcription factor WRINKLED1 in the biosynthesis of triacylglycerols during seed development. Frontiers in Plant Scienc | Sobic.010G13400 | yes | 78.5 | 78.25 | OTHER | 0.9991 OTHER 0.9941 | ortholo | yes | 1220.3 |
| glycolysi | PFP-?2 | Pyrophosphate dependent phospho-fructokinas | AT1G7655 Manuscript (Kuczynski; C.; McCorkle; S.; Keereetaweep; J.; Shanklin; J.; & Schwender; J. (2022). An expanded role for the transcription factor WRINKLED1 in the biosynthesis of triacylglycerols during seed development. Frontiers in Plant Scienc | Sobic.002G16030 | yes | 63.58 | 63.37 | OTHER | 0.9991 OTHER 0.9968 | ortholo | yes | 364.7 |
| glycolysi | PFP-?2 | Pyrophosphate dependent phospho-fructokinas | AT1G7655 Manuscript (Kuczynski; C.; McCorkle; S.; Keereetaweep; J.; Shanklin; J.; & Schwender; J. (2022). An expanded role for the transcription factor WRINKLED1 in the biosynthesis of triacylglycerols during seed development. Frontiers in Plant Scienc | Sobic.002G02630 | yes | 64.97 | 63.95 | OTHER | 0.9991 OTHER 0.9901 | ortholo | yes | 2858.1 |
| glycolysi | PFP-?2 | Pyrophosphate dependent phospho-fructokinas | AT1G7655 Manuscript (Kuczynski; C.; McCorkle; S.; Keereetaweep; J.; Shanklin; J.; & Schwender; J. (2022). An expanded role for the transcription factor WRINKLED1 in the biosynthesis of triacylglycerols during seed development. Frontiers in Plant Scienc | Sobic.007G10180 | yes | 62.12 | 65.25 | OTHER | 0.9991 OTHER 0.9991 | ortholo | yes | 248.3 |
| glycolysi | PGLM | phosphoglyceromutas | AT1G7805 Manuscript (Kuczynski; C.; McCorkle; S.; Keereetaweep; J.; Shanklin; J.; & Schwender; J. (2022). An expanded role for the transcription factor WRINKLED1 in the biosynthesis of triacylglycerols during seed development. Frontiers in Plant Scienc | Sobic.004G23410 | yes | 75.35 | 67.08 | cTP | 0.8832 cTP 0.9970 | ortholo | yes | 1564.1 |
| glycolysi | PGLM | phosphoglyceromutas | AT1G7805 Manuscript (Kuczynski; C.; McCorkle; S.; Keereetaweep; J.; Shanklin; J.; & Schwender; J. (2022). An expanded role for the transcription factor WRINKLED1 in the biosynthesis of triacylglycerols during seed development. Frontiers in Plant Scienc | Sobic.010G09010 | no | 73.05 | 66.26 | cTP | 0.8832 cTP 0.9596 | ortholo | yes | 6.0 |
| glycolysi | GAPDH | Glyceraldehyde 3-phosphate dehydrogenas | AT1G7953 Manuscript (Kuczynski; C.; McCorkle; S.; Keereetaweep; J.; Shanklin; J.; & Schwender; J. (2022). An expanded role for the transcription factor WRINKLED1 in the biosynthesis of triacylglycerols during seed development. Frontiers in Plant Scienc | Sobic.010G10020 | no | 80 | 44.94 | cTP | 0.9186 OTHER 0.9336 | no | no | 792.1 |
| glycolysi | GAPDH | Glyceraldehyde 3-phosphate dehydrogenas | AT1G7953 Manuscript (Kuczynski; C.; McCorkle; S.; Keereetaweep; J.; Shanklin; J.; & Schwender; J. (2022). An expanded role for the transcription factor WRINKLED1 in the biosynthesis of triacylglycerols during seed development. Frontiers in Plant Scienc | Sobic.004G20510 | no | 72.89 | 72.02 | cTP | 0.9186 OTHER 0.9225 | no | no | 3691.5 |
| glycolysi | GAPDH | Glyceraldehyde 3-phosphate dehydrogenas | AT1G7953 Manuscript (Kuczynski; C.; McCorkle; S.; Keereetaweep; J.; Shanklin; J.; & Schwender; J. (2022). An expanded role for the transcription factor WRINKLED1 in the biosynthesis of triacylglycerols during seed development. Frontiers in Plant Scienc | Sobic.009G01670 | no | 72.81 | 71.73 | cTP | 0.9186 OTHER 0.6901 | no | no | nd |
| glycolysi | GAPDH | Glyceraldehyde 3-phosphate dehydrogenas | AT1G7953 Manuscript (Kuczynski; C.; McCorkle; S.; Keereetaweep; J.; Shanklin; J.; & Schwender; J. (2022). An expanded role for the transcription factor WRINKLED1 in the biosynthesis of triacylglycerols during seed development. Frontiers in Plant Scienc | Sobic.010G26243 | no | 79.49 | 69.85 | cTP | 0.9186 OTHER 0.9970 | no | no | 1583.8 |
| glycolysi | GAPDH | Glyceraldehyde 3-phosphate dehydrogenas | AT1G7953 Manuscript (Kuczynski; C.; McCorkle; S.; Keereetaweep; J.; Shanklin; J.; & Schwender; J. (2022). An expanded role for the transcription factor WRINKLED1 in the biosynthesis of triacylglycerols during seed development. Frontiers in Plant Scienc | Sobic.010G26246 | no | 70.28 | 67.87 | cTP | 0.9186 OTHER 0.9644 | no | no | 6345.2 |
| glycolysi | GAPDH | Glyceraldehyde 3-phosphate dehydrogenas | AT1G7953 Manuscript (Kuczynski; C.; McCorkle; S.; Keereetaweep; J.; Shanklin; J.; & Schwender; J. (2022). An expanded role for the transcription factor WRINKLED1 in the biosynthesis of triacylglycerols during seed development. Frontiers in Plant Scienc | Sobic.007G02540 | no | 79.49 | 73.81 | cTP | 0.9186 OTHER 0.9663 | no | no | 12281.7 |
| glycolysi | GAPDH | Glyceraldehyde 3-phosphate dehydrogenas | AT1G7953 Manuscript (Kuczynski; C.; McCorkle; S.; Keereetaweep; J.; Shanklin; J.; & Schwender; J. (2022). An expanded role for the transcription factor WRINKLED1 in the biosynthesis of triacylglycerols during seed development. Frontiers in Plant Scienc | Sobic.010G26250 | no | 74.7 | 73.81 | cTP | 0.9186 OTHER 0.9649 | no | no | 4632.5 |
| glycolysi | GAPDH | Glyceraldehyde 3-phosphate dehydrogenas | AT1G7953 Manuscript (Kuczynski; C.; McCorkle; S.; Keereetaweep; J.; Shanklin; J.; & Schwender; J. (2022). An expanded role for the transcription factor WRINKLED1 in the biosynthesis of triacylglycerols during seed development. Frontiers in Plant Scienc | Sobic.004G05640 | bidirectiona | 85.4 | 78.35 | cTP | 0.9186 cTP 0.9413 | ortholo | no | 819.3 |
| glycolysi | GAPDH | Glyceraldehyde 3-phosphate dehydrogenas | AT1G7953 Manuscript (Kuczynski; C.; McCorkle; S.; Keereetaweep; J.; Shanklin; J.; & Schwender; J. (2022). An expanded role for the transcription factor WRINKLED1 in the biosynthesis of triacylglycerols during seed development. Frontiers in Plant Scienc | Sobic.006G10590 | no | 48.4 | 44.89 | cTP | 0.9186 cTP 0.9921 | no | no | 230502. |
| glycolysi | GAPDH | Glyceraldehyde 3-phosphate dehydrogenas | AT1G7953 Manuscript (Kuczynski; C.; McCorkle; S.; Keereetaweep; J.; Shanklin; J.; & Schwender; J. (2022). An expanded role for the transcription factor WRINKLED1 in the biosynthesis of triacylglycerols during seed development. Frontiers in Plant Scienc | Sobic.001G51980 | no | 51.18 | 45.52 | cTP | 0.9186 cTP 0.8801 | no | no | 236570. |
| glycolysi | PGK | Phosphoglycerokinas | AT1G7955 Manuscript (Kuczynski; C.; McCorkle; S.; Keereetaweep; J.; Shanklin; J.; & Schwender; J. (2022). An expanded role for the transcription factor WRINKLED1 in the biosynthesis of triacylglycerols during seed development. Frontiers in Plant Scienc | Sobic.009G18370 | no | 82.63 | 82.29 | OTHER | 0.9973 cTP 0.9990 | no | no | 35283.3 |
| glycolysi | PGK | Phosphoglycerokinas | AT1G7955 Manuscript (Kuczynski; C.; McCorkle; S.; Keereetaweep; J.; Shanklin; J.; & Schwender; J. (2022). An expanded role for the transcription factor WRINKLED1 in the biosynthesis of triacylglycerols during seed development. Frontiers in Plant Scienc | Sobic.004G05520 | bidirectiona | 86.6 | 86.78 | OTHER | 0.9973 OTHER 0.9977 | LDO | no | 22589.2 |
| glycolysi | PGK | Phosphoglycerokinas | AT1G7955 Manuscript (Kuczynski; C.; McCorkle; S.; Keereetaweep; J.; Shanklin; J.; & Schwender; J. (2022). An expanded role for the transcription factor WRINKLED1 in the biosynthesis of triacylglycerols during seed development. Frontiers in Plant Scienc | Sobic.010G22180 | yes | 85.57 | 85.54 | OTHER | 0.9973 OTHER 0.9992 | LDO | no | 294.1 |
| glycolysi | FBA | fructose bisphosphate aldolas | AT2G0114 Manuscript (Kuczynski; C.; McCorkle; S.; Keereetaweep; J.; Shanklin; J.; & Schwender; J. (2022). An expanded role for the transcription factor WRINKLED1 in the biosynthesis of triacylglycerols during seed development. Frontiers in Plant Scienc | Sobic.003G09600 | bidirectiona | 85.75 | 83.03 | cTP | 0.9933 cTP 0.9998 | LDO | no | 2060.9 |
| glycolysi | TPI | Triose phosphate isomeras | AT2G2117 Manuscript (Kuczynski; C.; McCorkle; S.; Keereetaweep; J.; Shanklin; J.; & Schwender; J. (2022). An expanded role for the transcription factor WRINKLED1 in the biosynthesis of triacylglycerols during seed development. Frontiers in Plant Scienc | Sobic.002G27710 | bidirectiona | 78.28 | 72.09 | cTP | 0.9942 cTP 0.9972 | ortholo | no | 12177.6 |
| glycolysi | FBA | fructose bisphosphate aldolas | AT2G2133 Manuscript (Kuczynski; C.; McCorkle; S.; Keereetaweep; J.; Shanklin; J.; & Schwender; J. (2022). An expanded role for the transcription factor WRINKLED1 in the biosynthesis of triacylglycerols during seed development. Frontiers in Plant Scienc | Sobic.005G05640 | no | 79.6 | 81.35 | cTP | 0.9996 cTP 0.9663 | ortholo | no | 40819.7 |
| glycolysi | FBA | fructose bisphosphate aldolas | AT2G2133 Manuscript (Kuczynski; C.; McCorkle; S.; Keereetaweep; J.; Shanklin; J.; & Schwender; J. (2022). An expanded role for the transcription factor WRINKLED1 in the biosynthesis of triacylglycerols during seed development. Frontiers in Plant Scienc | Sobic.008G05320 | no | 80.4 | 81.14 | cTP | 0.9996 cTP 0.9911 | LDO | no | 110939. |
| glycolysi | PFK | phosphofructokinas | AT2G2248 Manuscript (Kuczynski; C.; McCorkle; S.; Keereetaweep; J.; Shanklin; J.; & Schwender; J. (2022). An expanded role for the transcription factor WRINKLED1 in the biosynthesis of triacylglycerols during seed development. Frontiers in Plant Scienc | Sobic.001G25330 | yes | 71.34 | 66.4 | cTP | 0.9066 OTHER 0.5076 | no | no | 696.7 |
| glycolysi | PFK | phosphofructokinas | AT2G2248 Manuscript (Kuczynski; C.; McCorkle; S.; Keereetaweep; J.; Shanklin; J.; & Schwender; J. (2022). An expanded role for the transcription factor WRINKLED1 in the biosynthesis of triacylglycerols during seed development. Frontiers in Plant Scienc | Sobic.002G20170 | bidirectiona | 67.67 | 69.13 | cTP | 0.9066 cTP 0.8516 | no | no | 70.1 |
| glycolysi | PFK | phosphofructokinas | AT2G2248 Manuscript (Kuczynski; C.; McCorkle; S.; Keereetaweep; J.; Shanklin; J.; & Schwender; J. (2022). An expanded role for the transcription factor WRINKLED1 in the biosynthesis of triacylglycerols during seed development. Frontiers in Plant Scienc | Sobic.007G13950 | yes | 67.98 | 67.06 | cTP | 0.9066 cTP 0.9939 | no | no | 16.3 |
| glycolysi | PFK | phosphofructokinas | AT2G2248 Manuscript (Kuczynski; C.; McCorkle; S.; Keereetaweep; J.; Shanklin; J.; & Schwender; J. (2022). An expanded role for the transcription factor WRINKLED1 in the biosynthesis of triacylglycerols during seed development. Frontiers in Plant Scienc | Sobic.009G20460 | no | 46.49 | 44.82 | cTP | 0.9066 mTP 0.9787 | no | no | 3719.0 |
| glycolysi | PFK | phosphofructokinas | AT2G2248 Manuscript (Kuczynski; C.; McCorkle; S.; Keereetaweep; J.; Shanklin; J.; & Schwender; J. (2022). An expanded role for the transcription factor WRINKLED1 in the biosynthesis of triacylglycerols during seed development. Frontiers in Plant Scienc | Sobic.003G29000 | no | 46.19 | 45.59 | cTP | 0.9066 OTHER 0.9998 | no | no | 811.3 |
| glycolysi | PFK | phosphofructokinas | AT2G2248 Manuscript (Kuczynski; C.; McCorkle; S.; Keereetaweep; J.; Shanklin; J.; & Schwender; J. (2022). An expanded role for the transcription factor WRINKLED1 in the biosynthesis of triacylglycerols during seed development. Frontiers in Plant Scienc | Sobic.003G03470 | no | 48.58 | 49.12 | cTP | 0.9066 OTHER 0.9978 | no | no | 6163.5 |
| glycolysi | PFK | phosphofructokinas | AT2G2248 Manuscript (Kuczynski; C.; McCorkle; S.; Keereetaweep; J.; Shanklin; J.; & Schwender; J. (2022). An expanded role for the transcription factor WRINKLED1 in the biosynthesis of triacylglycerols during seed development. Frontiers in Plant Scienc | Sobic.001G05250 | no | 47.26 | 47.81 | cTP | 0.9066 OTHER 0.8309 | no | no | 7.7 |
| glycolysi | PFK | phosphofructokinas | AT2G2248 Manuscript (Kuczynski; C.; McCorkle; S.; Keereetaweep; J.; Shanklin; J.; & Schwender; J. (2022). An expanded role for the transcription factor WRINKLED1 in the biosynthesis of triacylglycerols during seed development. Frontiers in Plant Scienc | Sobic.009G07180 | no | 48.8 | 49.12 | cTP | 0.9066 OTHER 0.6405 | no | no | 1481.7 |
| glycolysi | PFK | phosphofructokinas | AT2G2248 Manuscript (Kuczynski; C.; McCorkle; S.; Keereetaweep; J.; Shanklin; J.; & Schwender; J. (2022). An expanded role for the transcription factor WRINKLED1 in the biosynthesis of triacylglycerols during seed development. Frontiers in Plant Scienc | Sobic.010G04140 | no | 47.25 | 46.6 | cTP | 0.9066 OTHER 0.9984 | no | no | 1127.6 |
| glycolysi | PFK | phosphofructokinas | AT2G2248 Manuscript (Kuczynski; C.; McCorkle; S.; Keereetaweep; J.; Shanklin; J.; & Schwender; J. (2022). An expanded role for the transcription factor WRINKLED1 in the biosynthesis of triacylglycerols during seed development. Frontiers in Plant Scienc | Sobic.006G07480 | no | 34.5 | 37.75 | cTP | 0.9066 OTHER 0.9991 | no | no | nd |
| glycolysi | PFK | phosphofructokinas | AT2G2248 Manuscript (Kuczynski; C.; McCorkle; S.; Keereetaweep; J.; Shanklin; J.; & Schwender; J. (2022). An expanded role for the transcription factor WRINKLED1 in the biosynthesis of triacylglycerols during seed development. Frontiers in Plant Scienc | Sobic.006G11470 | no | 46.07 | 40.26 | cTP | 0.9066 OTHER 0.9997 | no | no | 43.1 |
| glycolysi | ALDH | non-phosphorylating Glyceraldehyde 3-phosphate dehydrogena | AT2G2427 Manuscript (Kuczynski; C.; McCorkle; S.; Keereetaweep; J.; Shanklin; J.; & Schwender; J. (2022). An expanded role for the transcription factor WRINKLED1 in the biosynthesis of triacylglycerols during seed development. Frontiers in Plant Scienc | Sobic.007G14070 | bidirectiona | 87.7 | 87.7 | OTHER | 0.9995 OTHER 0.9674 | ortholo | no | 17099.6 |
| glycolysi | Eno | Enolase | AT2G2956 Manuscript (Kuczynski; C.; McCorkle; S.; Keereetaweep; J.; Shanklin; J.; & Schwender; J. (2022). An expanded role for the transcription factor WRINKLED1 in the biosynthesis of triacylglycerols during seed development. Frontiers in Plant Scienc | Sobic.001G42610 | bidirectiona | 64.83 | 64.42 | OTHER | 0.9993 OTHER 0.9995 | LDO | no | 491.7 |
| glycolysi | FRK2 | fructokinase | AT2G3139 Manuscript (Kuczynski; C.; McCorkle; S.; Keereetaweep; J.; Shanklin; J.; & Schwender; J. (2022). An expanded role for the transcription factor WRINKLED1 in the biosynthesis of triacylglycerols during seed development. Frontiers in Plant Scienc | Sobic.003G38600 | bidirectiona | 74.84 | 73.37 | OTHER | 0.9997 OTHER 0.9966 | ortholo | no | 5605.6 |
| glycolysi | FBA | fructose bisphosphate aldolas | AT2G3646 Manuscript (Kuczynski; C.; McCorkle; S.; Keereetaweep; J.; Shanklin; J.; & Schwender; J. (2022). An expanded role for the transcription factor WRINKLED1 in the biosynthesis of triacylglycerols during seed development. Frontiers in Plant Scienc | Sobic.003G39390 | yes | 81.34 | 81.97 | OTHER | 0.9980 OTHER 0.9998 | LDO | no | 9802.6 |
| glycolysi | FBA | fructose bisphosphate aldolas | AT2G3646 Manuscript (Kuczynski; C.; McCorkle; S.; Keereetaweep; J.; Shanklin; J.; & Schwender; J. (2022). An expanded role for the transcription factor WRINKLED1 in the biosynthesis of triacylglycerols during seed development. Frontiers in Plant Scienc | Sobic.004G14600 | bidirectiona | 83.57 | 83.52 | OTHER | 0.9980 OTHER 0.9998 | ortholo | no | 1756.7 |
| glycolysi | Eno | Enolase | AT2G3653 Manuscript (Kuczynski; C.; McCorkle; S.; Keereetaweep; J.; Shanklin; J.; & Schwender; J. (2022). An expanded role for the transcription factor WRINKLED1 in the biosynthesis of triacylglycerols during seed development. Frontiers in Plant Scienc | Sobic.010G02692 | bidirectiona | 86.71 | 86.49 | OTHER | 0.9248 OTHER 0.9613 | ortholo | yes | 8594.2 |
| glycolysi | Eno | Enolase | AT2G3653 Manuscript (Kuczynski; C.; McCorkle; S.; Keereetaweep; J.; Shanklin; J.; & Schwender; J. (2022). An expanded role for the transcription factor WRINKLED1 in the biosynthesis of triacylglycerols during seed development. Frontiers in Plant Scienc | Sobic.010G02700 | yes | 86.71 | 86.49 | OTHER | 0.9248 OTHER 0.9606 | ortholo | yes | 13170.6 |
| glycolysi | PK | pyruvate kinase | AT2G3658 Manuscript (Kuczynski; C.; McCorkle; S.; Keereetaweep; J.; Shanklin; J.; & Schwender; J. (2022). An expanded role for the transcription factor WRINKLED1 in the biosynthesis of triacylglycerols during seed development. Frontiers in Plant Scienc | Sobic.005G10140 | yes | 63.62 | 62.93 | OTHER | 0.9300 OTHER 0.9247 | no | no | 477.0 |
| glycolysi | PK | pyruvate kinase | AT2G3658 Manuscript (Kuczynski; C.; McCorkle; S.; Keereetaweep; J.; Shanklin; J.; & Schwender; J. (2022). An expanded role for the transcription factor WRINKLED1 in the biosynthesis of triacylglycerols during seed development. Frontiers in Plant Scienc | Sobic.005G03440 | no | 86.17 | 86.15 | OTHER | 0.9300 OTHER 0.9510 | LDO | no | 3031.3 |
| glycolysi | PK | pyruvate kinase | AT2G3658 Manuscript (Kuczynski; C.; McCorkle; S.; Keereetaweep; J.; Shanklin; J.; & Schwender; J. (2022). An expanded role for the transcription factor WRINKLED1 in the biosynthesis of triacylglycerols during seed development. Frontiers in Plant Scienc | Sobic.008G03360 | yes | 86.36 | 86.34 | OTHER | 0.9300 OTHER 0.9373 | ortholo | no | 4890.7 |
| glycolysi | GPDH | NAD- glycerol-3-phosphate dehydrogenas | AT2G4154 Manuscript (Kuczynski; C.; McCorkle; S.; Keereetaweep; J.; Shanklin; J.; & Schwender; J. (2022). An expanded role for the transcription factor WRINKLED1 in the biosynthesis of triacylglycerols during seed development. Frontiers in Plant Scienc | Sobic.003G32680 | yes | 80.68 | 78.17 | OTHER | 0.9973 OTHER 0.9999 | ortholo | yes | 23.2 |
| glycolysi | GPDH | NAD- glycerol-3-phosphate dehydrogenas | AT2G4154 Manuscript (Kuczynski; C.; McCorkle; S.; Keereetaweep; J.; Shanklin; J.; & Schwender; J. (2022). An expanded role for the transcription factor WRINKLED1 in the biosynthesis of triacylglycerols during seed development. Frontiers in Plant Scienc | Sobic.009G18330 | yes | 81.92 | 79.96 | OTHER | 0.9973 OTHER 0.9952 | LDO | yes | 104.2 |
| glycolysi | GPDH | NAD- glycerol-3-phosphate dehydrogenas | AT2G4154 Manuscript (Kuczynski; C.; McCorkle; S.; Keereetaweep; J.; Shanklin; J.; & Schwender; J. (2022). An expanded role for the transcription factor WRINKLED1 in the biosynthesis of triacylglycerols during seed development. Frontiers in Plant Scienc | Sobic.003G42080 | bidirectiona | 83.19 | 84.25 | OTHER | 0.9973 OTHER 0.9918 | ortholo | yes | 36.9 |
| glycolysi | PK | pyruvate kinase | AT3G0405 Manuscript (Kuczynski; C.; McCorkle; S.; Keereetaweep; J.; Shanklin; J.; & Schwender; J. (2022). An expanded role for the transcription factor WRINKLED1 in the biosynthesis of triacylglycerols during seed development. Frontiers in Plant Scienc | Sobic.006G26720 | no | 70.1 | 67.98 | OTHER | 0.9882 OTHER 0.9985 | no | no | 1530.2 |
| glycolysi | PK | pyruvate kinase | AT3G0405 Manuscript (Kuczynski; C.; McCorkle; S.; Keereetaweep; J.; Shanklin; J.; & Schwender; J. (2022). An expanded role for the transcription factor WRINKLED1 in the biosynthesis of triacylglycerols during seed development. Frontiers in Plant Scienc | Sobic.001G38690 | no | 68.69 | 68.64 | OTHER | 0.9882 OTHER 0.9151 | ortholo | no | nd |
| glycolysi | PK | pyruvate kinase | AT3G0405 Manuscript (Kuczynski; C.; McCorkle; S.; Keereetaweep; J.; Shanklin; J.; & Schwender; J. (2022). An expanded role for the transcription factor WRINKLED1 in the biosynthesis of triacylglycerols during seed development. Frontiers in Plant Scienc | Sobic.003G13080 | yes | 71.23 | 69.9 | OTHER | 0.9882 OTHER 0.9974 | ortholo | no | 5456.7 |
| glycolysi | PK | pyruvate kinase | AT3G0405 Manuscript (Kuczynski; C.; McCorkle; S.; Keereetaweep; J.; Shanklin; J.; & Schwender; J. (2022). An expanded role for the transcription factor WRINKLED1 in the biosynthesis of triacylglycerols during seed development. Frontiers in Plant Scienc | Sobic.006G26720 | no | 70.1 | 68.28 | OTHER | 0.9882 OTHER 0.9985 | no | no | 1530.2 |
| glycolysi | PK | pyruvate kinase | AT3G0405 Manuscript (Kuczynski; C.; McCorkle; S.; Keereetaweep; J.; Shanklin; J.; & Schwender; J. (2022). An expanded role for the transcription factor WRINKLED1 in the biosynthesis of triacylglycerols during seed development. Frontiers in Plant Scienc | Sobic.001G38690 | no | 68.69 | 69.49 | OTHER | 0.9882 OTHER 0.9151 | ortholo | no | nd |
| glycolysi | PK | pyruvate kinase | AT3G0405 Manuscript (Kuczynski; C.; McCorkle; S.; Keereetaweep; J.; Shanklin; J.; & Schwender; J. (2022). An expanded role for the transcription factor WRINKLED1 in the biosynthesis of triacylglycerols during seed development. Frontiers in Plant Scienc | Sobic.003G13080 | no | 81.78 | 70.45 | OTHER | 0.9882 OTHER 0.9974 | ortholo | no | 5456.7 |
| glycolysi | GAPDH | Glyceraldehyde 3-phosphate dehydrogenas | AT3G0412 Manuscript (Kuczynski; C.; McCorkle; S.; Keereetaweep; J.; Shanklin; J.; & Schwender; J. (2022). An expanded role for the transcription factor WRINKLED1 in the biosynthesis of triacylglycerols during seed development. Frontiers in Plant Scienc | Sobic.010G10020 | yes | 85.71 | 44.94 | OTHER | 0.9990 OTHER 0.9336 | no | no | 792.1 |
| glycolysi | GAPDH | Glyceraldehyde 3-phosphate dehydrogenas | AT3G0412 Manuscript (Kuczynski; C.; McCorkle; S.; Keereetaweep; J.; Shanklin; J.; & Schwender; J. (2022). An expanded role for the transcription factor WRINKLED1 in the biosynthesis of triacylglycerols during seed development. Frontiers in Plant Scienc | Sobic.004G20510 | yes | 87.05 | 86.01 | OTHER | 0.9990 OTHER 0.9225 | ortholo | no | 3691.5 |
| glycolysi | GAPDH | Glyceraldehyde 3-phosphate dehydrogenas | AT3G0412 Manuscript (Kuczynski; C.; McCorkle; S.; Keereetaweep; J.; Shanklin; J.; & Schwender; J. (2022). An expanded role for the transcription factor WRINKLED1 in the biosynthesis of triacylglycerols during seed development. Frontiers in Plant Scienc | Sobic.009G01670 | no | 86.1 | 84.82 | OTHER | 0.9990 OTHER 0.6901 | ortholo | no | nd |
| glycolysi | GAPDH | Glyceraldehyde 3-phosphate dehydrogenas | AT3G0412 Manuscript (Kuczynski; C.; McCorkle; S.; Keereetaweep; J.; Shanklin; J.; & Schwender; J. (2022). An expanded role for the transcription factor WRINKLED1 in the biosynthesis of triacylglycerols during seed development. Frontiers in Plant Scienc | Sobic.010G26243 | yes | 82.35 | 74.07 | OTHER | 0.9990 OTHER 0.9970 | no | no | 1583.8 |
| glycolysi | GAPDH | Glyceraldehyde 3-phosphate dehydrogenas | AT3G0412 Manuscript (Kuczynski; C.; McCorkle; S.; Keereetaweep; J.; Shanklin; J.; & Schwender; J. (2022). An expanded role for the transcription factor WRINKLED1 in the biosynthesis of triacylglycerols during seed development. Frontiers in Plant Scienc | Sobic.010G26246 | yes | 86.79 | 83.33 | OTHER | 0.9990 OTHER 0.9644 | no | no | 6345.2 |
| glycolysi | GAPDH | Glyceraldehyde 3-phosphate dehydrogenas | AT3G0412 Manuscript (Kuczynski; C.; McCorkle; S.; Keereetaweep; J.; Shanklin; J.; & Schwender; J. (2022). An expanded role for the transcription factor WRINKLED1 in the biosynthesis of triacylglycerols during seed development. Frontiers in Plant Scienc | Sobic.007G02540 | yes | 86.79 | 86.01 | OTHER | 0.9990 OTHER 0.9663 | no | no | 12281.7 |
| glycolysi | GAPDH | Glyceraldehyde 3-phosphate dehydrogenas | AT3G0412 Manuscript (Kuczynski; C.; McCorkle; S.; Keereetaweep; J.; Shanklin; J.; & Schwender; J. (2022). An expanded role for the transcription factor WRINKLED1 in the biosynthesis of triacylglycerols during seed development. Frontiers in Plant Scienc | Sobic.010G26250 | yes | 86.19 | 85.42 | OTHER | 0.9990 OTHER 0.9649 | no | no | 4632.5 |
| glycolysi | GAPDH | Glyceraldehyde 3-phosphate dehydrogenas | AT3G0412 Manuscript (Kuczynski; C.; McCorkle; S.; Keereetaweep; J.; Shanklin; J.; & Schwender; J. (2022). An expanded role for the transcription factor WRINKLED1 in the biosynthesis of triacylglycerols during seed development. Frontiers in Plant Scienc | Sobic.004G05640 | no | 86.79 | 71.81 | OTHER | 0.9990 cTP 0.9413 | no | no | 819.3 |
| glycolysi | GAPDH | Glyceraldehyde 3-phosphate dehydrogenas | AT3G0412 Manuscript (Kuczynski; C.; McCorkle; S.; Keereetaweep; J.; Shanklin; J.; & Schwender; J. (2022). An expanded role for the transcription factor WRINKLED1 in the biosynthesis of triacylglycerols during seed development. Frontiers in Plant Scienc | Sobic.006G10590 | no | 47.15 | 46.71 | OTHER | 0.9990 cTP 0.9921 | no | no | 230502. |
| glycolysi | GAPDH | Glyceraldehyde 3-phosphate dehydrogenas | AT3G0412 Manuscript (Kuczynski; C.; McCorkle; S.; Keereetaweep; J.; Shanklin; J.; & Schwender; J. (2022). An expanded role for the transcription factor WRINKLED1 in the biosynthesis of triacylglycerols during seed development. Frontiers in Plant Scienc | Sobic.001G51980 | no | 48.81 | 48.96 | OTHER | 0.9990 cTP 0.8801 | no | no | 236570. |
| glycolysi | GPDH | NAD- glycerol-3-phosphate dehydrogenas | AT3G0769 Manuscript (Kuczynski; C.; McCorkle; S.; Keereetaweep; J.; Shanklin; J.; & Schwender; J. (2022). An expanded role for the transcription factor WRINKLED1 in the biosynthesis of triacylglycerols during seed development. Frontiers in Plant Scienc | Sobic.003G32680 | no | 76.44 | 75.94 | OTHER | 0.9999 OTHER 0.9999 | ortholo | yes | 23.2 |
| glycolysi | GPDH | NAD- glycerol-3-phosphate dehydrogenas | AT3G0769 Manuscript (Kuczynski; C.; McCorkle; S.; Keereetaweep; J.; Shanklin; J.; & Schwender; J. (2022). An expanded role for the transcription factor WRINKLED1 in the biosynthesis of triacylglycerols during seed development. Frontiers in Plant Scienc | Sobic.009G18330 | no | 76.17 | 75.5 | OTHER | 0.9999 OTHER 0.9952 | ortholo | yes | 104.2 |
| glycolysi | GPDH | NAD- glycerol-3-phosphate dehydrogenas | AT3G0769 Manuscript (Kuczynski; C.; McCorkle; S.; Keereetaweep; J.; Shanklin; J.; & Schwender; J. (2022). An expanded role for the transcription factor WRINKLED1 in the biosynthesis of triacylglycerols during seed development. Frontiers in Plant Scienc | Sobic.003G42080 | no | 79.73 | 79.2 | OTHER | 0.9999 OTHER 0.9918 | ortholo | yes | 36.9 |
| glycolysi | GPDH | NAD- glycerol-3-phosphate dehydrogenas | AT3G0859 Manuscript (Kuczynski; C.; McCorkle; S.; Keereetaweep; J.; Shanklin; J.; & Schwender; J. (2022). An expanded role for the transcription factor WRINKLED1 in the biosynthesis of triacylglycerols during seed development. Frontiers in Plant Scienc | Sobic.001G38410 | no | 80 | 79.11 | OTHER | 0.9998 OTHER 0.9971 | no | no | 6.9 |
| glycolysi | GPDH | NAD- glycerol-3-phosphate dehydrogenas | AT3G0859 Manuscript (Kuczynski; C.; McCorkle; S.; Keereetaweep; J.; Shanklin; J.; & Schwender; J. (2022). An expanded role for the transcription factor WRINKLED1 in the biosynthesis of triacylglycerols during seed development. Frontiers in Plant Scienc | Sobic.003G33600 | bidirectiona | 84.31 | 83.9 | OTHER | 0.9998 OTHER 0.9903 | LDO | no | 16765.6 |
| glycolysi | PGLM | phosphoglyceromutas | AT3G0859 Manuscript (Kuczynski; C.; McCorkle; S.; Keereetaweep; J.; Shanklin; J.; & Schwender; J. (2022). An expanded role for the transcription factor WRINKLED1 in the biosynthesis of triacylglycerols during seed development. Frontiers in Plant Scienc | Sobic.003G33600 | bidirectiona | 84.31 | 84.31 | OTHER | 0.9998 OTHER 0.9903 | LDO | no | 16765.6 |
| glycolysi | PGK | Phosphoglycerokinas | AT3G1278 Manuscript (Kuczynski; C.; McCorkle; S.; Keereetaweep; J.; Shanklin; J.; & Schwender; J. (2022). An expanded role for the transcription factor WRINKLED1 in the biosynthesis of triacylglycerols during seed development. Frontiers in Plant Scienc | Sobic.009G18370 | yes | 83.52 | 79.33 | cTP | 0.9980 cTP 0.9990 | LDO | no | 35283.3 |
| glycolysi | PGK | Phosphoglycerokinas | AT3G1278 Manuscript (Kuczynski; C.; McCorkle; S.; Keereetaweep; J.; Shanklin; J.; & Schwender; J. (2022). An expanded role for the transcription factor WRINKLED1 in the biosynthesis of triacylglycerols during seed development. Frontiers in Plant Scienc | Sobic.004G05520 | no | 84.21 | 83.79 | cTP | 0.9980 OTHER 0.9977 | no | no | 22589.2 |
| glycolysi | PGK | Phosphoglycerokinas | AT3G1278 Manuscript (Kuczynski; C.; McCorkle; S.; Keereetaweep; J.; Shanklin; J.; & Schwender; J. (2022). An expanded role for the transcription factor WRINKLED1 in the biosynthesis of triacylglycerols during seed development. Frontiers in Plant Scienc | Sobic.010G22180 | no | 83.92 | 83.29 | cTP | 0.9980 OTHER 0.9992 | no | no | 294.1 |
| glycolysi | PK | pyruvate kinase | AT3G2296 Manuscript (Kuczynski; C.; McCorkle; S.; Keereetaweep; J.; Shanklin; J.; & Schwender; J. (2022). An expanded role for the transcription factor WRINKLED1 in the biosynthesis of triacylglycerols during seed development. Frontiers in Plant Scienc | Sobic.001G29060 | no | 44.42 | 42.1 | cTP | 0.9816 cTP 0.9844 | no | yes | 12978.9 |
| glycolysi | PK | pyruvate kinase | AT3G2296 Manuscript (Kuczynski; C.; McCorkle; S.; Keereetaweep; J.; Shanklin; J.; & Schwender; J. (2022). An expanded role for the transcription factor WRINKLED1 in the biosynthesis of triacylglycerols during seed development. Frontiers in Plant Scienc | Sobic.003G24470 | no | 46.48 | 41.08 | cTP | 0.9816 cTP 0.9844 | no | yes | 433.7 |
| glycolysi | PK | pyruvate kinase | AT3G2296 Manuscript (Kuczynski; C.; McCorkle; S.; Keereetaweep; J.; Shanklin; J.; & Schwender; J. (2022). An expanded role for the transcription factor WRINKLED1 in the biosynthesis of triacylglycerols during seed development. Frontiers in Plant Scienc | Sobic.001G14070 | yes | 60.71 | 58.86 | cTP | 0.9816 cTP 0.8821 | no | yes | 121.2 |
| glycolysi | PK | pyruvate kinase | AT3G2296 Manuscript (Kuczynski; C.; McCorkle; S.; Keereetaweep; J.; Shanklin; J.; & Schwender; J. (2022). An expanded role for the transcription factor WRINKLED1 in the biosynthesis of triacylglycerols during seed development. Frontiers in Plant Scienc | Sobic.001G05720 | yes | 84.58 | 76.27 | cTP | 0.9816 cTP 0.7216 | ortholo | yes | 691.8 |
| glycolysi | PK | pyruvate kinase | AT3G2296 Manuscript (Kuczynski; C.; McCorkle; S.; Keereetaweep; J.; Shanklin; J.; & Schwender; J. (2022). An expanded role for the transcription factor WRINKLED1 in the biosynthesis of triacylglycerols during seed development. Frontiers in Plant Scienc | Sobic.002G05440 | bidirectiona | 78.69 | 78.07 | cTP | 0.9816 cTP 0.9818 | LDO | yes | 6679.5 |
| glycolysi | PK | pyruvate kinase | AT3G2596 Manuscript (Kuczynski; C.; McCorkle; S.; Keereetaweep; J.; Shanklin; J.; & Schwender; J. (2022). An expanded role for the transcription factor WRINKLED1 in the biosynthesis of triacylglycerols during seed development. Frontiers in Plant Scienc | Sobic.006G26720 | no | 68.84 | 68.28 | OTHER | 0.9172 OTHER 0.9985 | no | no | 1530.2 |
| glycolysi | PK | pyruvate kinase | AT3G2596 Manuscript (Kuczynski; C.; McCorkle; S.; Keereetaweep; J.; Shanklin; J.; & Schwender; J. (2022). An expanded role for the transcription factor WRINKLED1 in the biosynthesis of triacylglycerols during seed development. Frontiers in Plant Scienc | Sobic.001G38690 | no | 67.84 | 69.49 | OTHER | 0.9172 OTHER 0.9151 | ortholo | no | nd |
| glycolysi | PK | pyruvate kinase | AT3G2596 Manuscript (Kuczynski; C.; McCorkle; S.; Keereetaweep; J.; Shanklin; J.; & Schwender; J. (2022). An expanded role for the transcription factor WRINKLED1 in the biosynthesis of triacylglycerols during seed development. Frontiers in Plant Scienc | Sobic.003G13080 | yes | 70.3 | 70.45 | OTHER | 0.9172 OTHER 0.9974 | ortholo | no | 5456.7 |
| glycolysi | GAPDH | Glyceraldehyde 3-phosphate dehydrogenas | AT3G2665 Manuscript (Kuczynski; C.; McCorkle; S.; Keereetaweep; J.; Shanklin; J.; & Schwender; J. (2022). An expanded role for the transcription factor WRINKLED1 in the biosynthesis of triacylglycerols during seed development. Frontiers in Plant Scienc | Sobic.004G20510 | no | 48.04 | 48.05 | cTP | 0.9230 OTHER 0.9225 | no | no | 3691.5 |
| glycolysi | GAPDH | Glyceraldehyde 3-phosphate dehydrogenas | AT3G2665 Manuscript (Kuczynski; C.; McCorkle; S.; Keereetaweep; J.; Shanklin; J.; & Schwender; J. (2022). An expanded role for the transcription factor WRINKLED1 in the biosynthesis of triacylglycerols during seed development. Frontiers in Plant Scienc | Sobic.009G01670 | no | 47.73 | 47.15 | cTP | 0.9230 OTHER 0.6901 | no | no | nd |
| glycolysi | GAPDH | Glyceraldehyde 3-phosphate dehydrogenas | AT3G2665 Manuscript (Kuczynski; C.; McCorkle; S.; Keereetaweep; J.; Shanklin; J.; & Schwender; J. (2022). An expanded role for the transcription factor WRINKLED1 in the biosynthesis of triacylglycerols during seed development. Frontiers in Plant Scienc | Sobic.010G26243 | no | 46.02 | 39.55 | cTP | 0.9230 OTHER 0.9970 | no | no | 1583.8 |
| glycolysi | GAPDH | Glyceraldehyde 3-phosphate dehydrogenas | AT3G2665 Manuscript (Kuczynski; C.; McCorkle; S.; Keereetaweep; J.; Shanklin; J.; & Schwender; J. (2022). An expanded role for the transcription factor WRINKLED1 in the biosynthesis of triacylglycerols during seed development. Frontiers in Plant Scienc | Sobic.010G26246 | no | 45.97 | 43.84 | cTP | 0.9230 OTHER 0.9644 | no | no | 6345.2 |
| glycolysi | GAPDH | Glyceraldehyde 3-phosphate dehydrogenas | AT3G2665 Manuscript (Kuczynski; C.; McCorkle; S.; Keereetaweep; J.; Shanklin; J.; & Schwender; J. (2022). An expanded role for the transcription factor WRINKLED1 in the biosynthesis of triacylglycerols during seed development. Frontiers in Plant Scienc | Sobic.007G02540 | no | 46.36 | 45.95 | cTP | 0.9230 OTHER 0.9663 | no | no | 12281.7 |
| glycolysi | GAPDH | Glyceraldehyde 3-phosphate dehydrogenas | AT3G2665 Manuscript (Kuczynski; C.; McCorkle; S.; Keereetaweep; J.; Shanklin; J.; & Schwender; J. (2022). An expanded role for the transcription factor WRINKLED1 in the biosynthesis of triacylglycerols during seed development. Frontiers in Plant Scienc | Sobic.010G26250 | no | 46.67 | 45.95 | cTP | 0.9230 OTHER 0.9649 | no | no | 4632.5 |
| glycolysi | GAPDH | Glyceraldehyde 3-phosphate dehydrogenas | AT3G2665 Manuscript (Kuczynski; C.; McCorkle; S.; Keereetaweep; J.; Shanklin; J.; & Schwender; J. (2022). An expanded role for the transcription factor WRINKLED1 in the biosynthesis of triacylglycerols during seed development. Frontiers in Plant Scienc | Sobic.004G05640 | no | 45.9 | 41.16 | cTP | 0.9230 cTP 0.9413 | no | no | 819.3 |
| glycolysi | GAPDH | Glyceraldehyde 3-phosphate dehydrogenas | AT3G2665 Manuscript (Kuczynski; C.; McCorkle; S.; Keereetaweep; J.; Shanklin; J.; & Schwender; J. (2022). An expanded role for the transcription factor WRINKLED1 in the biosynthesis of triacylglycerols during seed development. Frontiers in Plant Scienc | Sobic.006G10590 | yes | 85.82 | 84.3 | cTP | 0.9230 cTP 0.9921 | ortholo | no | 230502. |
| glycolysi | GAPDH | Glyceraldehyde 3-phosphate dehydrogenas | AT3G2665 Manuscript (Kuczynski; C.; McCorkle; S.; Keereetaweep; J.; Shanklin; J.; & Schwender; J. (2022). An expanded role for the transcription factor WRINKLED1 in the biosynthesis of triacylglycerols during seed development. Frontiers in Plant Scienc | Sobic.001G51980 | no | 73.63 | 72.94 | cTP | 0.9230 cTP 0.8801 | no | no | 236570. |
| glycolysi | PGLM | phosphoglyceromutas | AT3G5052 Manuscript (Kuczynski; C.; McCorkle; S.; Keereetaweep; J.; Shanklin; J.; & Schwender; J. (2022). An expanded role for the transcription factor WRINKLED1 in the biosynthesis of triacylglycerols during seed development. Frontiers in Plant Scienc | Sobic.007G15830 | bidirectiona | 56.56 | 56.56 | OTHER | 1.0000 cTP 0.5202 | LDO | no | 1168.3 |
| glycolysi | FBA | fructose bisphosphate aldolas | AT3G5293 Manuscript (Kuczynski; C.; McCorkle; S.; Keereetaweep; J.; Shanklin; J.; & Schwender; J. (2022). An expanded role for the transcription factor WRINKLED1 in the biosynthesis of triacylglycerols during seed development. Frontiers in Plant Scienc | Sobic.003G39390 | no | 81.34 | 81.97 | OTHER | 0.9974 OTHER 0.9998 | ortholo | no | 9802.6 |
| glycolysi | FBA | fructose bisphosphate aldolas | AT3G5293 Manuscript (Kuczynski; C.; McCorkle; S.; Keereetaweep; J.; Shanklin; J.; & Schwender; J. (2022). An expanded role for the transcription factor WRINKLED1 in the biosynthesis of triacylglycerols during seed development. Frontiers in Plant Scienc | Sobic.004G14600 | no | 82.73 | 82.68 | OTHER | 0.9974 OTHER 0.9998 | ortholo | no | 1756.7 |
| glycolysi | PK | pyruvate kinase | AT3G5299 Manuscript (Kuczynski; C.; McCorkle; S.; Keereetaweep; J.; Shanklin; J.; & Schwender; J. (2022). An expanded role for the transcription factor WRINKLED1 in the biosynthesis of triacylglycerols during seed development. Frontiers in Plant Scienc | Sobic.005G10140 | no | 63.43 | 62.74 | OTHER | 0.9048 OTHER 0.9247 | no | no | 477.0 |
| glycolysi | PK | pyruvate kinase | AT3G5299 Manuscript (Kuczynski; C.; McCorkle; S.; Keereetaweep; J.; Shanklin; J.; & Schwender; J. (2022). An expanded role for the transcription factor WRINKLED1 in the biosynthesis of triacylglycerols during seed development. Frontiers in Plant Scienc | Sobic.005G03440 | yes | 86.93 | 86.91 | OTHER | 0.9048 OTHER 0.9510 | LDO | no | 3031.3 |
| glycolysi | PK | pyruvate kinase | AT3G5299 Manuscript (Kuczynski; C.; McCorkle; S.; Keereetaweep; J.; Shanklin; J.; & Schwender; J. (2022). An expanded role for the transcription factor WRINKLED1 in the biosynthesis of triacylglycerols during seed development. Frontiers in Plant Scienc | Sobic.008G03360 | bidirectiona | 87.31 | 87.29 | OTHER | 0.9048 OTHER 0.9373 | ortholo | no | 4890.7 |
| glycolysi | FBP | fructose-1;6-bisphosphatas | AT3G5405 Manuscript (Kuczynski; C.; McCorkle; S.; Keereetaweep; J.; Shanklin; J.; & Schwender; J. (2022). An expanded role for the transcription factor WRINKLED1 in the biosynthesis of triacylglycerols during seed development. Frontiers in Plant Scienc | Sobic.001G42540 | bidirectiona | 80.71 | 77.97 | cTP | 0.9989 cTP 0.8521 | LDO | no | 10647.5 |
| glycolysi | TPI | Triose phosphate isomeras | AT3G5544 Manuscript (Kuczynski; C.; McCorkle; S.; Keereetaweep; J.; Shanklin; J.; & Schwender; J. (2022). An expanded role for the transcription factor WRINKLED1 in the biosynthesis of triacylglycerols during seed development. Frontiers in Plant Scienc | Sobic.003G07230 | yes | 81.03 | 81.03 | OTHER | 0.9871 OTHER 0.9996 | LDO | yes | 4170.7 |
| glycolysi | TPI | Triose phosphate isomeras | AT3G5544 Manuscript (Kuczynski; C.; McCorkle; S.; Keereetaweep; J.; Shanklin; J.; & Schwender; J. (2022). An expanded role for the transcription factor WRINKLED1 in the biosynthesis of triacylglycerols during seed development. Frontiers in Plant Scienc | Sobic.003G35230 | bidirectiona | 80.16 | 79.53 | OTHER | 0.9871 OTHER 0.9957 | no | yes | 97.7 |
| glycolysi | PK | pyruvate kinase | AT3G5565 Manuscript (Kuczynski; C.; McCorkle; S.; Keereetaweep; J.; Shanklin; J.; & Schwender; J. (2022). An expanded role for the transcription factor WRINKLED1 in the biosynthesis of triacylglycerols during seed development. Frontiers in Plant Scienc | Sobic.006G26720 | no | 68.28 | 66.8 | OTHER | 0.8656 OTHER 0.9985 | no | no | 1530.2 |
| glycolysi | PK | pyruvate kinase | AT3G5565 Manuscript (Kuczynski; C.; McCorkle; S.; Keereetaweep; J.; Shanklin; J.; & Schwender; J. (2022). An expanded role for the transcription factor WRINKLED1 in the biosynthesis of triacylglycerols during seed development. Frontiers in Plant Scienc | Sobic.001G38690 | no | 66.73 | 67.06 | OTHER | 0.8656 OTHER 0.9151 | ortholo | no | nd |
| glycolysi | PK | pyruvate kinase | AT3G5565 Manuscript (Kuczynski; C.; McCorkle; S.; Keereetaweep; J.; Shanklin; J.; & Schwender; J. (2022). An expanded role for the transcription factor WRINKLED1 in the biosynthesis of triacylglycerols during seed development. Frontiers in Plant Scienc | Sobic.003G13080 | yes | 69.42 | 68.32 | OTHER | 0.8656 OTHER 0.9974 | ortholo | no | 5456.7 |
| glycolysi | PK | pyruvate kinase | AT3G5581 Manuscript (Kuczynski; C.; McCorkle; S.; Keereetaweep; J.; Shanklin; J.; & Schwender; J. (2022). An expanded role for the transcription factor WRINKLED1 in the biosynthesis of triacylglycerols during seed development. Frontiers in Plant Scienc | Sobic.006G26720 | no | 64.48 | 66.39 | OTHER | 0.8938 OTHER 0.9985 | no | no | 1530.2 |
| glycolysi | PK | pyruvate kinase | AT3G5581 Manuscript (Kuczynski; C.; McCorkle; S.; Keereetaweep; J.; Shanklin; J.; & Schwender; J. (2022). An expanded role for the transcription factor WRINKLED1 in the biosynthesis of triacylglycerols during seed development. Frontiers in Plant Scienc | Sobic.001G38690 | no | 64.59 | 66.05 | OTHER | 0.8938 OTHER 0.9151 | ortholo | no | nd |
| glycolysi | PK | pyruvate kinase | AT3G5581 Manuscript (Kuczynski; C.; McCorkle; S.; Keereetaweep; J.; Shanklin; J.; & Schwender; J. (2022). An expanded role for the transcription factor WRINKLED1 in the biosynthesis of triacylglycerols during seed development. Frontiers in Plant Scienc | Sobic.003G13080 | yes | 66.6 | 67.97 | OTHER | 0.8938 OTHER 0.9974 | ortholo | no | 5456.7 |
| glycolysi | FRK7 | fructokinase | AT3G5948 Manuscript (Kuczynski; C.; McCorkle; S.; Keereetaweep; J.; Shanklin; J.; & Schwender; J. (2022). An expanded role for the transcription factor WRINKLED1 in the biosynthesis of triacylglycerols during seed development. Frontiers in Plant Scienc | Sobic.003G38600 | no | 71.61 | 70.28 | OTHER | 0.9987 OTHER 0.9966 | LDO | no | 5605.6 |
| glycolysi | PFP-ß2 | Pyrophosphate dependent phospho-fructokinas | AT4G0404 Manuscript (Kuczynski; C.; McCorkle; S.; Keereetaweep; J.; Shanklin; J.; & Schwender; J. (2022). An expanded role for the transcription factor WRINKLED1 in the biosynthesis of triacylglycerols during seed development. Frontiers in Plant Scienc | Sobic.010G10160 | yes | 79.93 | 77.88 | OTHER | 0.9987 cTP 0.8850 | ortholo | no | 1452.6 |
| glycolysi | PFP-ß2 | Pyrophosphate dependent phospho-fructokinas | AT4G0404 Manuscript (Kuczynski; C.; McCorkle; S.; Keereetaweep; J.; Shanklin; J.; & Schwender; J. (2022). An expanded role for the transcription factor WRINKLED1 in the biosynthesis of triacylglycerols during seed development. Frontiers in Plant Scienc | Sobic.004G26180 | no | 40.47 | 38.93 | OTHER | 0.9987 OTHER 0.9995 | no | no | 5.8 |
| glycolysi | PFP-ß2 | Pyrophosphate dependent phospho-fructokinas | AT4G0404 Manuscript (Kuczynski; C.; McCorkle; S.; Keereetaweep; J.; Shanklin; J.; & Schwender; J. (2022). An expanded role for the transcription factor WRINKLED1 in the biosynthesis of triacylglycerols during seed development. Frontiers in Plant Scienc | Sobic.010G13400 | no | 40.75 | 38.83 | OTHER | 0.9987 OTHER 0.9941 | no | no | 1220.3 |

| glycolysi | PFP-ß2 | Pyrophosphate dependent phospho-fructokinas | AT4G0404 Manuscript (Kuczynski; C.; McCorkle; S.; Keereetaweep; J.; Shanklin; J.; & Schwender; J. (2022). An expanded role for the transcription factor WRINKLED1 in the biosynthesis of triacylglycerols during seed development. Frontiers in Plant Scienc | Sobic.002G16030 | no | 41.13 | 37.52 | OTHER | 0.9987 OTHER 0.9968 | no | no | 364.7 |
| --- | --- | --- | --- | --- | --- | --- | --- | --- | --- | --- | --- | --- |
| glycolysi | PFP-ß2 | Pyrophosphate dependent phospho-fructokinas | AT4G0404 Manuscript (Kuczynski; C.; McCorkle; S.; Keereetaweep; J.; Shanklin; J.; & Schwender; J. (2022). An expanded role for the transcription factor WRINKLED1 in the biosynthesis of triacylglycerols during seed development. Frontiers in Plant Scienc | Sobic.002G02630 | no | 40 | 39.04 | OTHER | 0.9987 OTHER 0.9901 | no | no | 2858.1 |
| glycolysi | PFP-ß2 | Pyrophosphate dependent phospho-fructokinas | AT4G0404 Manuscript (Kuczynski; C.; McCorkle; S.; Keereetaweep; J.; Shanklin; J.; & Schwender; J. (2022). An expanded role for the transcription factor WRINKLED1 in the biosynthesis of triacylglycerols during seed development. Frontiers in Plant Scienc | Sobic.007G10180 | no | 42.2 | 41.24 | OTHER | 0.9987 OTHER 0.9991 | no | no | 248.3 |
| glycolysi | FRK4 | fructokinase | AT4G1026 Manuscript (Kuczynski; C.; McCorkle; S.; Keereetaweep; J.; Shanklin; J.; & Schwender; J. (2022). An expanded role for the transcription factor WRINKLED1 in the biosynthesis of triacylglycerols during seed development. Frontiers in Plant Scienc | Sobic.007G01470 | bidirectiona | 75.16 | 73.37 | OTHER | 0.9980 OTHER 0.9997 | LDO | no | 4567.9 |
| glycolysi | PPDK | pyruvate orthophosphate dikinas | AT4G1553 Manuscript (Kuczynski; C.; McCorkle; S.; Keereetaweep; J.; Shanklin; J.; & Schwender; J. (2022). An expanded role for the transcription factor WRINKLED1 in the biosynthesis of triacylglycerols during seed development. Frontiers in Plant Scienc | Sobic.001G32690 | yes | 74.46 | 74.72 | OTHER | 0.8906 OTHER 0.9999 | ortholo | no | 238.4 |
| glycolysi | PPDK | pyruvate orthophosphate dikinas | AT4G1553 Manuscript (Kuczynski; C.; McCorkle; S.; Keereetaweep; J.; Shanklin; J.; & Schwender; J. (2022). An expanded role for the transcription factor WRINKLED1 in the biosynthesis of triacylglycerols during seed development. Frontiers in Plant Scienc | Sobic.009G13290 | bidirectiona | 76.5 | 75.96 | OTHER | 0.8906 cTP 0.9963 | LDO | no | 494085. |
| glycolysi | PGI | phosphoglucose isomeras | AT4G2462 Manuscript (Kuczynski; C.; McCorkle; S.; Keereetaweep; J.; Shanklin; J.; & Schwender; J. (2022). An expanded role for the transcription factor WRINKLED1 in the biosynthesis of triacylglycerols during seed development. Frontiers in Plant Scienc | Sobic.002G23060 | bidirectiona | 83.21 | 76.4 | cTP | 0.9496 cTP 0.6765 | LDO | yes | 4144.0 |
| glycolysi | PFK | phosphofructokinas | AT4G2627 Manuscript (Kuczynski; C.; McCorkle; S.; Keereetaweep; J.; Shanklin; J.; & Schwender; J. (2022). An expanded role for the transcription factor WRINKLED1 in the biosynthesis of triacylglycerols during seed development. Frontiers in Plant Scienc | Sobic.001G25330 | no | 51.33 | 47.78 | OTHER | 0.9947 OTHER 0.5076 | no | no | 696.7 |
| glycolysi | PFK | phosphofructokinas | AT4G2627 Manuscript (Kuczynski; C.; McCorkle; S.; Keereetaweep; J.; Shanklin; J.; & Schwender; J. (2022). An expanded role for the transcription factor WRINKLED1 in the biosynthesis of triacylglycerols during seed development. Frontiers in Plant Scienc | Sobic.002G20170 | no | 55.05 | 50.93 | OTHER | 0.9947 cTP 0.8516 | no | no | 70.1 |
| glycolysi | PFK | phosphofructokinas | AT4G2627 Manuscript (Kuczynski; C.; McCorkle; S.; Keereetaweep; J.; Shanklin; J.; & Schwender; J. (2022). An expanded role for the transcription factor WRINKLED1 in the biosynthesis of triacylglycerols during seed development. Frontiers in Plant Scienc | Sobic.007G13950 | no | 53.91 | 46.86 | OTHER | 0.9947 cTP 0.9939 | no | no | 16.3 |
| glycolysi | PFK | phosphofructokinas | AT4G2627 Manuscript (Kuczynski; C.; McCorkle; S.; Keereetaweep; J.; Shanklin; J.; & Schwender; J. (2022). An expanded role for the transcription factor WRINKLED1 in the biosynthesis of triacylglycerols during seed development. Frontiers in Plant Scienc | Sobic.009G20460 | yes | 73.59 | 70.87 | OTHER | 0.9947 mTP 0.9787 | no | no | 3719.0 |
| glycolysi | PFK | phosphofructokinas | AT4G2627 Manuscript (Kuczynski; C.; McCorkle; S.; Keereetaweep; J.; Shanklin; J.; & Schwender; J. (2022). An expanded role for the transcription factor WRINKLED1 in the biosynthesis of triacylglycerols during seed development. Frontiers in Plant Scienc | Sobic.003G29000 | yes | 75.55 | 71.46 | OTHER | 0.9947 OTHER 0.9998 | no | no | 811.3 |
| glycolysi | PFK | phosphofructokinas | AT4G2627 Manuscript (Kuczynski; C.; McCorkle; S.; Keereetaweep; J.; Shanklin; J.; & Schwender; J. (2022). An expanded role for the transcription factor WRINKLED1 in the biosynthesis of triacylglycerols during seed development. Frontiers in Plant Scienc | Sobic.003G03470 | bidirectiona | 75.51 | 75.26 | OTHER | 0.9947 OTHER 0.9978 | no | no | 6163.5 |
| glycolysi | PFK | phosphofructokinas | AT4G2627 Manuscript (Kuczynski; C.; McCorkle; S.; Keereetaweep; J.; Shanklin; J.; & Schwender; J. (2022). An expanded role for the transcription factor WRINKLED1 in the biosynthesis of triacylglycerols during seed development. Frontiers in Plant Scienc | Sobic.001G05250 | yes | 73.96 | 70.37 | OTHER | 0.9947 OTHER 0.8309 | no | no | 7.7 |
| glycolysi | PFK | phosphofructokinas | AT4G2627 Manuscript (Kuczynski; C.; McCorkle; S.; Keereetaweep; J.; Shanklin; J.; & Schwender; J. (2022). An expanded role for the transcription factor WRINKLED1 in the biosynthesis of triacylglycerols during seed development. Frontiers in Plant Scienc | Sobic.009G07180 | yes | 76.55 | 72.35 | OTHER | 0.9947 OTHER 0.6405 | no | no | 1481.7 |
| glycolysi | PFK | phosphofructokinas | AT4G2627 Manuscript (Kuczynski; C.; McCorkle; S.; Keereetaweep; J.; Shanklin; J.; & Schwender; J. (2022). An expanded role for the transcription factor WRINKLED1 in the biosynthesis of triacylglycerols during seed development. Frontiers in Plant Scienc | Sobic.010G04140 | yes | 80.38 | 78.83 | OTHER | 0.9947 OTHER 0.9984 | no | no | 1127.6 |
| glycolysi | PFK | phosphofructokinas | AT4G2627 Manuscript (Kuczynski; C.; McCorkle; S.; Keereetaweep; J.; Shanklin; J.; & Schwender; J. (2022). An expanded role for the transcription factor WRINKLED1 in the biosynthesis of triacylglycerols during seed development. Frontiers in Plant Scienc | Sobic.006G07480 | no | 40 | 42.21 | OTHER | 0.9947 OTHER 0.9991 | no | no | nd |
| glycolysi | PFK | phosphofructokinas | AT4G2627 Manuscript (Kuczynski; C.; McCorkle; S.; Keereetaweep; J.; Shanklin; J.; & Schwender; J. (2022). An expanded role for the transcription factor WRINKLED1 in the biosynthesis of triacylglycerols during seed development. Frontiers in Plant Scienc | Sobic.006G11470 | no | 52.14 | 49.45 | OTHER | 0.9947 OTHER 0.9997 | no | no | 43.1 |
| glycolysi | PK | pyruvate kinase | AT4G2639 Manuscript (Kuczynski; C.; McCorkle; S.; Keereetaweep; J.; Shanklin; J.; & Schwender; J. (2022). An expanded role for the transcription factor WRINKLED1 in the biosynthesis of triacylglycerols during seed development. Frontiers in Plant Scienc | Sobic.006G26720 | no | 77.64 | 76.66 | OTHER | 0.9661 OTHER 0.9985 | no | no | 1530.2 |
| glycolysi | PK | pyruvate kinase | AT4G2639 Manuscript (Kuczynski; C.; McCorkle; S.; Keereetaweep; J.; Shanklin; J.; & Schwender; J. (2022). An expanded role for the transcription factor WRINKLED1 in the biosynthesis of triacylglycerols during seed development. Frontiers in Plant Scienc | Sobic.001G38690 | no | 75.56 | 74.85 | OTHER | 0.9661 OTHER 0.9151 | ortholo | no | nd |
| glycolysi | PK | pyruvate kinase | AT4G2639 Manuscript (Kuczynski; C.; McCorkle; S.; Keereetaweep; J.; Shanklin; J.; & Schwender; J. (2022). An expanded role for the transcription factor WRINKLED1 in the biosynthesis of triacylglycerols during seed development. Frontiers in Plant Scienc | Sobic.003G13080 | yes | 79.35 | 78.83 | OTHER | 0.9661 OTHER 0.9974 | ortholo | no | 5456.7 |
| glycolysi | FBA | fructose bisphosphate aldolas | AT4G2652 Manuscript (Kuczynski; C.; McCorkle; S.; Keereetaweep; J.; Shanklin; J.; & Schwender; J. (2022). An expanded role for the transcription factor WRINKLED1 in the biosynthesis of triacylglycerols during seed development. Frontiers in Plant Scienc | Sobic.003G39390 | no | 75.49 | 71.55 | OTHER | 0.9992 OTHER 0.9998 | no | no | 9802.6 |
| glycolysi | FBA | fructose bisphosphate aldolas | AT4G2652 Manuscript (Kuczynski; C.; McCorkle; S.; Keereetaweep; J.; Shanklin; J.; & Schwender; J. (2022). An expanded role for the transcription factor WRINKLED1 in the biosynthesis of triacylglycerols during seed development. Frontiers in Plant Scienc | Sobic.004G14600 | no | 72.14 | 72.07 | OTHER | 0.9992 OTHER 0.9998 | no | no | 1756.7 |
| glycolysi | FBA | fructose bisphosphate aldolas | AT4G2652 Manuscript (Kuczynski; C.; McCorkle; S.; Keereetaweep; J.; Shanklin; J.; & Schwender; J. (2022). An expanded role for the transcription factor WRINKLED1 in the biosynthesis of triacylglycerols during seed development. Frontiers in Plant Scienc | Sobic.010G18830 | no | 74.09 | 74.02 | OTHER | 0.9992 OTHER 0.9958 | ortholo | no | 9113.8 |
| glycolysi | FBA | fructose bisphosphate aldolas | AT4G2653 Manuscript (Kuczynski; C.; McCorkle; S.; Keereetaweep; J.; Shanklin; J.; & Schwender; J. (2022). An expanded role for the transcription factor WRINKLED1 in the biosynthesis of triacylglycerols during seed development. Frontiers in Plant Scienc | Sobic.003G39390 | no | 75.49 | 76.06 | OTHER | 0.9978 OTHER 0.9998 | no | no | 9802.6 |
| glycolysi | FBA | fructose bisphosphate aldolas | AT4G2653 Manuscript (Kuczynski; C.; McCorkle; S.; Keereetaweep; J.; Shanklin; J.; & Schwender; J. (2022). An expanded role for the transcription factor WRINKLED1 in the biosynthesis of triacylglycerols during seed development. Frontiers in Plant Scienc | Sobic.004G14600 | no | 77.72 | 77.65 | OTHER | 0.9978 OTHER 0.9998 | no | no | 1756.7 |
| glycolysi | FBA | fructose bisphosphate aldolas | AT4G2653 Manuscript (Kuczynski; C.; McCorkle; S.; Keereetaweep; J.; Shanklin; J.; & Schwender; J. (2022). An expanded role for the transcription factor WRINKLED1 in the biosynthesis of triacylglycerols during seed development. Frontiers in Plant Scienc | Sobic.010G18830 | bidirectiona | 80.78 | 80.73 | OTHER | 0.9978 OTHER 0.9958 | LDO | no | 9113.8 |
| glycolysi | PFK | phosphofructokinas | AT4G2922 Manuscript (Kuczynski; C.; McCorkle; S.; Keereetaweep; J.; Shanklin; J.; & Schwender; J. (2022). An expanded role for the transcription factor WRINKLED1 in the biosynthesis of triacylglycerols during seed development. Frontiers in Plant Scienc | Sobic.001G25330 | no | 45.7 | 47.42 | OTHER | 0.9976 OTHER 0.5076 | no | no | 696.7 |
| glycolysi | PFK | phosphofructokinas | AT4G2922 Manuscript (Kuczynski; C.; McCorkle; S.; Keereetaweep; J.; Shanklin; J.; & Schwender; J. (2022). An expanded role for the transcription factor WRINKLED1 in the biosynthesis of triacylglycerols during seed development. Frontiers in Plant Scienc | Sobic.002G20170 | no | 75 | 50.59 | OTHER | 0.9976 cTP 0.8516 | no | no | 70.1 |
| glycolysi | PFK | phosphofructokinas | AT4G2922 Manuscript (Kuczynski; C.; McCorkle; S.; Keereetaweep; J.; Shanklin; J.; & Schwender; J. (2022). An expanded role for the transcription factor WRINKLED1 in the biosynthesis of triacylglycerols during seed development. Frontiers in Plant Scienc | Sobic.007G13950 | no | 53.49 | 46.62 | OTHER | 0.9976 cTP 0.9939 | no | no | 16.3 |
| glycolysi | PFK | phosphofructokinas | AT4G2922 Manuscript (Kuczynski; C.; McCorkle; S.; Keereetaweep; J.; Shanklin; J.; & Schwender; J. (2022). An expanded role for the transcription factor WRINKLED1 in the biosynthesis of triacylglycerols during seed development. Frontiers in Plant Scienc | Sobic.009G20460 | no | 74.55 | 71.34 | OTHER | 0.9976 mTP 0.9787 | no | no | 3719.0 |
| glycolysi | PFK | phosphofructokinas | AT4G2922 Manuscript (Kuczynski; C.; McCorkle; S.; Keereetaweep; J.; Shanklin; J.; & Schwender; J. (2022). An expanded role for the transcription factor WRINKLED1 in the biosynthesis of triacylglycerols during seed development. Frontiers in Plant Scienc | Sobic.003G29000 | no | 74.55 | 70.7 | OTHER | 0.9976 OTHER 0.9998 | no | no | 811.3 |
| glycolysi | PFK | phosphofructokinas | AT4G2922 Manuscript (Kuczynski; C.; McCorkle; S.; Keereetaweep; J.; Shanklin; J.; & Schwender; J. (2022). An expanded role for the transcription factor WRINKLED1 in the biosynthesis of triacylglycerols during seed development. Frontiers in Plant Scienc | Sobic.003G03470 | no | 73.38 | 72.77 | OTHER | 0.9976 OTHER 0.9978 | no | no | 6163.5 |
| glycolysi | PFK | phosphofructokinas | AT4G2922 Manuscript (Kuczynski; C.; McCorkle; S.; Keereetaweep; J.; Shanklin; J.; & Schwender; J. (2022). An expanded role for the transcription factor WRINKLED1 in the biosynthesis of triacylglycerols during seed development. Frontiers in Plant Scienc | Sobic.001G05250 | no | 72.67 | 69.51 | OTHER | 0.9976 OTHER 0.8309 | no | no | 7.7 |
| glycolysi | PFK | phosphofructokinas | AT4G2922 Manuscript (Kuczynski; C.; McCorkle; S.; Keereetaweep; J.; Shanklin; J.; & Schwender; J. (2022). An expanded role for the transcription factor WRINKLED1 in the biosynthesis of triacylglycerols during seed development. Frontiers in Plant Scienc | Sobic.009G07180 | no | 75.06 | 71.37 | OTHER | 0.9976 OTHER 0.6405 | no | no | 1481.7 |
| glycolysi | PFK | phosphofructokinas | AT4G2922 Manuscript (Kuczynski; C.; McCorkle; S.; Keereetaweep; J.; Shanklin; J.; & Schwender; J. (2022). An expanded role for the transcription factor WRINKLED1 in the biosynthesis of triacylglycerols during seed development. Frontiers in Plant Scienc | Sobic.010G04140 | yes | 79.73 | 75.16 | OTHER | 0.9976 OTHER 0.9984 | no | no | 1127.6 |
| glycolysi | PFK | phosphofructokinas | AT4G2922 Manuscript (Kuczynski; C.; McCorkle; S.; Keereetaweep; J.; Shanklin; J.; & Schwender; J. (2022). An expanded role for the transcription factor WRINKLED1 in the biosynthesis of triacylglycerols during seed development. Frontiers in Plant Scienc | Sobic.006G07480 | no | 42.86 | 45.45 | OTHER | 0.9976 OTHER 0.9991 | no | no | nd |
| glycolysi | PFK | phosphofructokinas | AT4G2922 Manuscript (Kuczynski; C.; McCorkle; S.; Keereetaweep; J.; Shanklin; J.; & Schwender; J. (2022). An expanded role for the transcription factor WRINKLED1 in the biosynthesis of triacylglycerols during seed development. Frontiers in Plant Scienc | Sobic.006G11470 | no | 53.04 | 49 | OTHER | 0.9976 OTHER 0.9997 | no | no | 43.1 |
| glycolysi | PFK | phosphofructokinas | AT4G3284 Manuscript (Kuczynski; C.; McCorkle; S.; Keereetaweep; J.; Shanklin; J.; & Schwender; J. (2022). An expanded role for the transcription factor WRINKLED1 in the biosynthesis of triacylglycerols during seed development. Frontiers in Plant Scienc | Sobic.001G25330 | no | 50.27 | 46.82 | OTHER | 0.9996 OTHER 0.5076 | no | no | 696.7 |
| glycolysi | PFK | phosphofructokinas | AT4G3284 Manuscript (Kuczynski; C.; McCorkle; S.; Keereetaweep; J.; Shanklin; J.; & Schwender; J. (2022). An expanded role for the transcription factor WRINKLED1 in the biosynthesis of triacylglycerols during seed development. Frontiers in Plant Scienc | Sobic.002G20170 | no | 52.94 | 48.83 | OTHER | 0.9996 cTP 0.8516 | no | no | 70.1 |
| glycolysi | PFK | phosphofructokinas | AT4G3284 Manuscript (Kuczynski; C.; McCorkle; S.; Keereetaweep; J.; Shanklin; J.; & Schwender; J. (2022). An expanded role for the transcription factor WRINKLED1 in the biosynthesis of triacylglycerols during seed development. Frontiers in Plant Scienc | Sobic.007G13950 | no | 52.3 | 46.08 | OTHER | 0.9996 cTP 0.9939 | no | no | 16.3 |
| glycolysi | PFK | phosphofructokinas | AT4G3284 Manuscript (Kuczynski; C.; McCorkle; S.; Keereetaweep; J.; Shanklin; J.; & Schwender; J. (2022). An expanded role for the transcription factor WRINKLED1 in the biosynthesis of triacylglycerols during seed development. Frontiers in Plant Scienc | Sobic.009G20460 | no | 74.44 | 71.96 | OTHER | 0.9996 mTP 0.9787 | no | no | 3719.0 |
| glycolysi | PFK | phosphofructokinas | AT4G3284 Manuscript (Kuczynski; C.; McCorkle; S.; Keereetaweep; J.; Shanklin; J.; & Schwender; J. (2022). An expanded role for the transcription factor WRINKLED1 in the biosynthesis of triacylglycerols during seed development. Frontiers in Plant Scienc | Sobic.003G29000 | no | 76.91 | 74.13 | OTHER | 0.9996 OTHER 0.9998 | no | no | 811.3 |
| glycolysi | PFK | phosphofructokinas | AT4G3284 Manuscript (Kuczynski; C.; McCorkle; S.; Keereetaweep; J.; Shanklin; J.; & Schwender; J. (2022). An expanded role for the transcription factor WRINKLED1 in the biosynthesis of triacylglycerols during seed development. Frontiers in Plant Scienc | Sobic.003G03470 | no | 78.88 | 76.19 | OTHER | 0.9996 OTHER 0.9978 | no | no | 6163.5 |
| glycolysi | PFK | phosphofructokinas | AT4G3284 Manuscript (Kuczynski; C.; McCorkle; S.; Keereetaweep; J.; Shanklin; J.; & Schwender; J. (2022). An expanded role for the transcription factor WRINKLED1 in the biosynthesis of triacylglycerols during seed development. Frontiers in Plant Scienc | Sobic.001G05250 | no | 72.47 | 71.46 | OTHER | 0.9996 OTHER 0.8309 | no | no | 7.7 |
| glycolysi | PFK | phosphofructokinas | AT4G3284 Manuscript (Kuczynski; C.; McCorkle; S.; Keereetaweep; J.; Shanklin; J.; & Schwender; J. (2022). An expanded role for the transcription factor WRINKLED1 in the biosynthesis of triacylglycerols during seed development. Frontiers in Plant Scienc | Sobic.009G07180 | no | 76.36 | 73.8 | OTHER | 0.9996 OTHER 0.6405 | no | no | 1481.7 |
| glycolysi | PFK | phosphofructokinas | AT4G3284 Manuscript (Kuczynski; C.; McCorkle; S.; Keereetaweep; J.; Shanklin; J.; & Schwender; J. (2022). An expanded role for the transcription factor WRINKLED1 in the biosynthesis of triacylglycerols during seed development. Frontiers in Plant Scienc | Sobic.010G04140 | yes | 79.23 | 76.14 | OTHER | 0.9996 OTHER 0.9984 | no | no | 1127.6 |
| glycolysi | PFK | phosphofructokinas | AT4G3284 Manuscript (Kuczynski; C.; McCorkle; S.; Keereetaweep; J.; Shanklin; J.; & Schwender; J. (2022). An expanded role for the transcription factor WRINKLED1 in the biosynthesis of triacylglycerols during seed development. Frontiers in Plant Scienc | Sobic.006G07480 | no | 41.76 | 44.44 | OTHER | 0.9996 OTHER 0.9991 | no | no | nd |
| glycolysi | PFK | phosphofructokinas | AT4G3284 Manuscript (Kuczynski; C.; McCorkle; S.; Keereetaweep; J.; Shanklin; J.; & Schwender; J. (2022). An expanded role for the transcription factor WRINKLED1 in the biosynthesis of triacylglycerols during seed development. Frontiers in Plant Scienc | Sobic.006G11470 | no | 57.33 | 50.89 | OTHER | 0.9996 OTHER 0.9997 | no | no | 43.1 |
| glycolysi | FBA | fructose bisphosphate aldolas | AT4G3897 Manuscript (Kuczynski; C.; McCorkle; S.; Keereetaweep; J.; Shanklin; J.; & Schwender; J. (2022). An expanded role for the transcription factor WRINKLED1 in the biosynthesis of triacylglycerols during seed development. Frontiers in Plant Scienc | Sobic.005G05640 | bidirectiona | 80.45 | 82.08 | cTP | 0.9989 cTP 0.9663 | ortholo | no | 40819.7 |
| glycolysi | FBA | fructose bisphosphate aldolas | AT4G3897 Manuscript (Kuczynski; C.; McCorkle; S.; Keereetaweep; J.; Shanklin; J.; & Schwender; J. (2022). An expanded role for the transcription factor WRINKLED1 in the biosynthesis of triacylglycerols during seed development. Frontiers in Plant Scienc | Sobic.008G05320 | yes | 80 | 81.61 | cTP | 0.9989 cTP 0.9911 | ortholo | no | 110939. |
| glycolysi | FBA | fructose bisphosphate aldolas | AT5G0369 Manuscript (Kuczynski; C.; McCorkle; S.; Keereetaweep; J.; Shanklin; J.; & Schwender; J. (2022). An expanded role for the transcription factor WRINKLED1 in the biosynthesis of triacylglycerols during seed development. Frontiers in Plant Scienc | Sobic.003G39390 | no | 81.71 | 80.56 | cTP | 0.0158 OTHER 0.9998 | ortholo | no | 9802.6 |
| glycolysi | FBA | fructose bisphosphate aldolas | AT5G0369 Manuscript (Kuczynski; C.; McCorkle; S.; Keereetaweep; J.; Shanklin; J.; & Schwender; J. (2022). An expanded role for the transcription factor WRINKLED1 in the biosynthesis of triacylglycerols during seed development. Frontiers in Plant Scienc | Sobic.004G14600 | no | 82.86 | 81.01 | cTP | 0.0158 OTHER 0.9998 | ortholo | no | 1756.7 |
| glycolysi | PGLM | phosphoglyceromutas | AT5G0412 Manuscript (Kuczynski; C.; McCorkle; S.; Keereetaweep; J.; Shanklin; J.; & Schwender; J. (2022). An expanded role for the transcription factor WRINKLED1 in the biosynthesis of triacylglycerols during seed development. Frontiers in Plant Scienc | Sobic.007G15830 | yes | 49.07 | 49.07 | OTHER | 1.0000 cTP 0.5202 | ortholo | no | 1168.3 |
| glycolysi | PK | pyruvate kinase | AT5G0857 Manuscript (Kuczynski; C.; McCorkle; S.; Keereetaweep; J.; Shanklin; J.; & Schwender; J. (2022). An expanded role for the transcription factor WRINKLED1 in the biosynthesis of triacylglycerols during seed development. Frontiers in Plant Scienc | Sobic.006G26720 | bidirectiona | 84.24 | 84.71 | OTHER | 0.9995 OTHER 0.9985 | ortholo | no | 1530.2 |
| glycolysi | PK | pyruvate kinase | AT5G0857 Manuscript (Kuczynski; C.; McCorkle; S.; Keereetaweep; J.; Shanklin; J.; & Schwender; J. (2022). An expanded role for the transcription factor WRINKLED1 in the biosynthesis of triacylglycerols during seed development. Frontiers in Plant Scienc | Sobic.001G38690 | no | 74.55 | 72.35 | OTHER | 0.9995 OTHER 0.9151 | no | no | nd |
| glycolysi | PK | pyruvate kinase | AT5G0857 Manuscript (Kuczynski; C.; McCorkle; S.; Keereetaweep; J.; Shanklin; J.; & Schwender; J. (2022). An expanded role for the transcription factor WRINKLED1 in the biosynthesis of triacylglycerols during seed development. Frontiers in Plant Scienc | Sobic.003G13080 | no | 77.6 | 76.48 | OTHER | 0.9995 OTHER 0.9974 | no | no | 5456.7 |
| glycolysi | TA | transaldolase | AT5G1342 Manuscript (Kuczynski; C.; McCorkle; S.; Keereetaweep; J.; Shanklin; J.; & Schwender; J. (2022). An expanded role for the transcription factor WRINKLED1 in the biosynthesis of triacylglycerols during seed development. Frontiers in Plant Scienc | Sobic.003G41050 | bidirectiona | 81.91 | 74.77 | cTP | 0.9969 cTP 0.9988 | LDO | yes | 3472.4 |
| glycolysi | GPDH | NAD- glycerol-3-phosphate dehydrogenas | AT5G4061 Manuscript (Kuczynski; C.; McCorkle; S.; Keereetaweep; J.; Shanklin; J.; & Schwender; J. (2022). An expanded role for the transcription factor WRINKLED1 in the biosynthesis of triacylglycerols during seed development. Frontiers in Plant Scienc | Sobic.003G44340 | bidirectiona | 76.22 | 75.35 | mTP | 0.9944 OTHER 0.9842 | LDO | no | 166.5 |
| glycolysi | PGI | phosphoglucose isomeras | AT5G4274 Manuscript (Kuczynski; C.; McCorkle; S.; Keereetaweep; J.; Shanklin; J.; & Schwender; J. (2022). An expanded role for the transcription factor WRINKLED1 in the biosynthesis of triacylglycerols during seed development. Frontiers in Plant Scienc | Sobic.001G07180 | bidirectiona | 81.82 | 80.5 | OTHER | 0.9999 OTHER 0.9993 | LDO | no | 6385.8 |
| glycolysi | PFK | phosphofructokinas | AT5G4781 Manuscript (Kuczynski; C.; McCorkle; S.; Keereetaweep; J.; Shanklin; J.; & Schwender; J. (2022). An expanded role for the transcription factor WRINKLED1 in the biosynthesis of triacylglycerols during seed development. Frontiers in Plant Scienc | Sobic.001G25330 | no | 46.19 | 44.13 | OTHER | 0.9930 OTHER 0.5076 | no | no | 696.7 |
| glycolysi | PFK | phosphofructokinas | AT5G4781 Manuscript (Kuczynski; C.; McCorkle; S.; Keereetaweep; J.; Shanklin; J.; & Schwender; J. (2022). An expanded role for the transcription factor WRINKLED1 in the biosynthesis of triacylglycerols during seed development. Frontiers in Plant Scienc | Sobic.002G20170 | no | 48.16 | 45.2 | OTHER | 0.9930 cTP 0.8516 | no | no | 70.1 |
| glycolysi | PFK | phosphofructokinas | AT5G4781 Manuscript (Kuczynski; C.; McCorkle; S.; Keereetaweep; J.; Shanklin; J.; & Schwender; J. (2022). An expanded role for the transcription factor WRINKLED1 in the biosynthesis of triacylglycerols during seed development. Frontiers in Plant Scienc | Sobic.007G13950 | no | 48.37 | 43.82 | OTHER | 0.9930 cTP 0.9939 | no | no | 16.3 |
| glycolysi | PFK | phosphofructokinas | AT5G4781 Manuscript (Kuczynski; C.; McCorkle; S.; Keereetaweep; J.; Shanklin; J.; & Schwender; J. (2022). An expanded role for the transcription factor WRINKLED1 in the biosynthesis of triacylglycerols during seed development. Frontiers in Plant Scienc | Sobic.009G20460 | no | 56.46 | 54.11 | OTHER | 0.9930 mTP 0.9787 | no | no | 3719.0 |
| glycolysi | PFK | phosphofructokinas | AT5G4781 Manuscript (Kuczynski; C.; McCorkle; S.; Keereetaweep; J.; Shanklin; J.; & Schwender; J. (2022). An expanded role for the transcription factor WRINKLED1 in the biosynthesis of triacylglycerols during seed development. Frontiers in Plant Scienc | Sobic.003G29000 | no | 56.7 | 54.57 | OTHER | 0.9930 OTHER 0.9998 | no | no | 811.3 |
| glycolysi | PFK | phosphofructokinas | AT5G4781 Manuscript (Kuczynski; C.; McCorkle; S.; Keereetaweep; J.; Shanklin; J.; & Schwender; J. (2022). An expanded role for the transcription factor WRINKLED1 in the biosynthesis of triacylglycerols during seed development. Frontiers in Plant Scienc | Sobic.003G03470 | no | 56.7 | 54.32 | OTHER | 0.9930 OTHER 0.9978 | no | no | 6163.5 |
| glycolysi | PFK | phosphofructokinas | AT5G4781 Manuscript (Kuczynski; C.; McCorkle; S.; Keereetaweep; J.; Shanklin; J.; & Schwender; J. (2022). An expanded role for the transcription factor WRINKLED1 in the biosynthesis of triacylglycerols during seed development. Frontiers in Plant Scienc | Sobic.001G05250 | no | 54.67 | 53.18 | OTHER | 0.9930 OTHER 0.8309 | no | no | 7.7 |
| glycolysi | PFK | phosphofructokinas | AT5G4781 Manuscript (Kuczynski; C.; McCorkle; S.; Keereetaweep; J.; Shanklin; J.; & Schwender; J. (2022). An expanded role for the transcription factor WRINKLED1 in the biosynthesis of triacylglycerols during seed development. Frontiers in Plant Scienc | Sobic.009G07180 | no | 55.94 | 54.55 | OTHER | 0.9930 OTHER 0.6405 | no | no | 1481.7 |
| glycolysi | PFK | phosphofructokinas | AT5G4781 Manuscript (Kuczynski; C.; McCorkle; S.; Keereetaweep; J.; Shanklin; J.; & Schwender; J. (2022). An expanded role for the transcription factor WRINKLED1 in the biosynthesis of triacylglycerols during seed development. Frontiers in Plant Scienc | Sobic.010G04140 | no | 55.11 | 53.42 | OTHER | 0.9930 OTHER 0.9984 | no | no | 1127.6 |
| glycolysi | PFK | phosphofructokinas | AT5G4781 Manuscript (Kuczynski; C.; McCorkle; S.; Keereetaweep; J.; Shanklin; J.; & Schwender; J. (2022). An expanded role for the transcription factor WRINKLED1 in the biosynthesis of triacylglycerols during seed development. Frontiers in Plant Scienc | Sobic.006G07480 | yes | 53.8 | 57.32 | OTHER | 0.9930 OTHER 0.9991 | no | no | nd |
| glycolysi | PFK | phosphofructokinas | AT5G4781 Manuscript (Kuczynski; C.; McCorkle; S.; Keereetaweep; J.; Shanklin; J.; & Schwender; J. (2022). An expanded role for the transcription factor WRINKLED1 in the biosynthesis of triacylglycerols during seed development. Frontiers in Plant Scienc | Sobic.006G11470 | bidirectiona | 74.41 | 71.11 | OTHER | 0.9930 OTHER 0.9997 | LDO | no | 43.1 |
| glycolysi | PGLM | phosphoglyceromutas | AT5G5182 Manuscript (Kuczynski; C.; McCorkle; S.; Keereetaweep; J.; Shanklin; J.; & Schwender; J. (2022). An expanded role for the transcription factor WRINKLED1 in the biosynthesis of triacylglycerols during seed development. Frontiers in Plant Scienc | Sobic.001G11650 | no | 59 | 56.81 | cTP | 0.9286 cTP 0.9286 | ortholo | no | 11791.8 |
| glycolysi | PGLM | phosphoglyceromutas | AT5G5182 Manuscript (Kuczynski; C.; McCorkle; S.; Keereetaweep; J.; Shanklin; J.; & Schwender; J. (2022). An expanded role for the transcription factor WRINKLED1 in the biosynthesis of triacylglycerols during seed development. Frontiers in Plant Scienc | Sobic.003G22250 | bidirectiona | 80.14 | 76.15 | cTP | 0.9286 cTP 0.9286 | LDO | no | 4204.7 |
| glycolysi | FRK1 | fructokinase | AT5G5183 Manuscript (Kuczynski; C.; McCorkle; S.; Keereetaweep; J.; Shanklin; J.; & Schwender; J. (2022). An expanded role for the transcription factor WRINKLED1 in the biosynthesis of triacylglycerols during seed development. Frontiers in Plant Scienc | Sobic.010G09500 | no | 72.78 | 67.85 | OTHER | 1.0000 cTP 0.9994 | no | no | 820.7 |
| glycolysi | FRK1 | fructokinase | AT5G5183 Manuscript (Kuczynski; C.; McCorkle; S.; Keereetaweep; J.; Shanklin; J.; & Schwender; J. (2022). An expanded role for the transcription factor WRINKLED1 in the biosynthesis of triacylglycerols during seed development. Frontiers in Plant Scienc | Sobic.003G38600 | no | 65.19 | 63.47 | OTHER | 1.0000 OTHER 0.9966 | no | no | 5605.6 |
| glycolysi | PK | pyruvate kinase | AT5G5292 Manuscript (Kuczynski; C.; McCorkle; S.; Keereetaweep; J.; Shanklin; J.; & Schwender; J. (2022). An expanded role for the transcription factor WRINKLED1 in the biosynthesis of triacylglycerols during seed development. Frontiers in Plant Scienc | Sobic.001G29060 | yes | 76.09 | 68.86 | cTP | 0.6677 cTP 0.9514 | no | yes | 12978.9 |
| glycolysi | PK | pyruvate kinase | AT5G5292 Manuscript (Kuczynski; C.; McCorkle; S.; Keereetaweep; J.; Shanklin; J.; & Schwender; J. (2022). An expanded role for the transcription factor WRINKLED1 in the biosynthesis of triacylglycerols during seed development. Frontiers in Plant Scienc | Sobic.003G24470 | bidirectiona | 81.11 | 78.7 | cTP | 0.6677 cTP 0.9844 | LDO | yes | 433.7 |
| glycolysi | PK | pyruvate kinase | AT5G5292 Manuscript (Kuczynski; C.; McCorkle; S.; Keereetaweep; J.; Shanklin; J.; & Schwender; J. (2022). An expanded role for the transcription factor WRINKLED1 in the biosynthesis of triacylglycerols during seed development. Frontiers in Plant Scienc | Sobic.001G05720 | no | 44.21 | 40.82 | cTP | 0.6677 cTP 0.7216 | no | yes | 691.8 |
| glycolysi | PK | pyruvate kinase | AT5G5292 Manuscript (Kuczynski; C.; McCorkle; S.; Keereetaweep; J.; Shanklin; J.; & Schwender; J. (2022). An expanded role for the transcription factor WRINKLED1 in the biosynthesis of triacylglycerols during seed development. Frontiers in Plant Scienc | Sobic.002G05440 | no | 44.54 | 40.04 | cTP | 0.6677 cTP 0.9818 | no | yes | 6679.5 |
| glycolysi | PK | pyruvate kinase | AT5G5635 Manuscript (Kuczynski; C.; McCorkle; S.; Keereetaweep; J.; Shanklin; J.; & Schwender; J. (2022). An expanded role for the transcription factor WRINKLED1 in the biosynthesis of triacylglycerols during seed development. Frontiers in Plant Scienc | Sobic.006G26720 | no | 79.47 | 78.51 | OTHER | 0.8995 OTHER 0.9985 | no | no | 1530.2 |
| glycolysi | PK | pyruvate kinase | AT5G5635 Manuscript (Kuczynski; C.; McCorkle; S.; Keereetaweep; J.; Shanklin; J.; & Schwender; J. (2022). An expanded role for the transcription factor WRINKLED1 in the biosynthesis of triacylglycerols during seed development. Frontiers in Plant Scienc | Sobic.001G38690 | yes | 78.18 | 77.51 | OTHER | 0.8995 OTHER 0.9151 | ortholo | no | nd |
| glycolysi | PK | pyruvate kinase | AT5G5635 Manuscript (Kuczynski; C.; McCorkle; S.; Keereetaweep; J.; Shanklin; J.; & Schwender; J. (2022). An expanded role for the transcription factor WRINKLED1 in the biosynthesis of triacylglycerols during seed development. Frontiers in Plant Scienc | Sobic.003G13080 | bidirectiona | 81.78 | 81.09 | OTHER | 0.8995 OTHER 0.9974 | LDO | no | 5456.7 |
| glycolysi | PK | pyruvate kinase | AT5G5635 Manuscript (Kuczynski; C.; McCorkle; S.; Keereetaweep; J.; Shanklin; J.; & Schwender; J. (2022). An expanded role for the transcription factor WRINKLED1 in the biosynthesis of triacylglycerols during seed development. Frontiers in Plant Scienc | Sobic.005G10140 | no | 40.61 | 40.08 | OTHER | 0.8995 OTHER 0.9247 | no | no | 477.0 |
| glycolysi | PK | pyruvate kinase | AT5G5635 Manuscript (Kuczynski; C.; McCorkle; S.; Keereetaweep; J.; Shanklin; J.; & Schwender; J. (2022). An expanded role for the transcription factor WRINKLED1 in the biosynthesis of triacylglycerols during seed development. Frontiers in Plant Scienc | Sobic.005G03440 | no | 42.54 | 42.02 | OTHER | 0.8995 OTHER 0.9510 | no | no | 3031.3 |
| glycolysi | PK | pyruvate kinase | AT5G5635 Manuscript (Kuczynski; C.; McCorkle; S.; Keereetaweep; J.; Shanklin; J.; & Schwender; J. (2022). An expanded role for the transcription factor WRINKLED1 in the biosynthesis of triacylglycerols during seed development. Frontiers in Plant Scienc | Sobic.008G03360 | no | 42.14 | 41.82 | OTHER | 0.8995 OTHER 0.9373 | no | no | 4890.7 |
| glycolysi | PFK | phosphofructokinas | AT5G5663 Manuscript (Kuczynski; C.; McCorkle; S.; Keereetaweep; J.; Shanklin; J.; & Schwender; J. (2022). An expanded role for the transcription factor WRINKLED1 in the biosynthesis of triacylglycerols during seed development. Frontiers in Plant Scienc | Sobic.001G25330 | no | 51.47 | 48.12 | OTHER | 0.9975 OTHER 0.5076 | no | no | 696.7 |
| glycolysi | PFK | phosphofructokinas | AT5G5663 Manuscript (Kuczynski; C.; McCorkle; S.; Keereetaweep; J.; Shanklin; J.; & Schwender; J. (2022). An expanded role for the transcription factor WRINKLED1 in the biosynthesis of triacylglycerols during seed development. Frontiers in Plant Scienc | Sobic.002G20170 | no | 54.93 | 51.05 | OTHER | 0.9975 cTP 0.8516 | no | no | 70.1 |
| glycolysi | PFK | phosphofructokinas | AT5G5663 Manuscript (Kuczynski; C.; McCorkle; S.; Keereetaweep; J.; Shanklin; J.; & Schwender; J. (2022). An expanded role for the transcription factor WRINKLED1 in the biosynthesis of triacylglycerols during seed development. Frontiers in Plant Scienc | Sobic.007G13950 | no | 54.32 | 47.3 | OTHER | 0.9975 cTP 0.9939 | no | no | 16.3 |
| glycolysi | PFK | phosphofructokinas | AT5G5663 Manuscript (Kuczynski; C.; McCorkle; S.; Keereetaweep; J.; Shanklin; J.; & Schwender; J. (2022). An expanded role for the transcription factor WRINKLED1 in the biosynthesis of triacylglycerols during seed development. Frontiers in Plant Scienc | Sobic.009G20460 | no | 73.66 | 71.04 | OTHER | 0.9975 mTP 0.9787 | no | no | 3719.0 |
| glycolysi | PFK | phosphofructokinas | AT5G5663 Manuscript (Kuczynski; C.; McCorkle; S.; Keereetaweep; J.; Shanklin; J.; & Schwender; J. (2022). An expanded role for the transcription factor WRINKLED1 in the biosynthesis of triacylglycerols during seed development. Frontiers in Plant Scienc | Sobic.003G29000 | no | 77.63 | 72.67 | OTHER | 0.9975 OTHER 0.9998 | no | no | 811.3 |
| glycolysi | PFK | phosphofructokinas | AT5G5663 Manuscript (Kuczynski; C.; McCorkle; S.; Keereetaweep; J.; Shanklin; J.; & Schwender; J. (2022). An expanded role for the transcription factor WRINKLED1 in the biosynthesis of triacylglycerols during seed development. Frontiers in Plant Scienc | Sobic.003G03470 | no | 77.32 | 74.84 | OTHER | 0.9975 OTHER 0.9978 | no | no | 6163.5 |
| glycolysi | PFK | phosphofructokinas | AT5G5663 Manuscript (Kuczynski; C.; McCorkle; S.; Keereetaweep; J.; Shanklin; J.; & Schwender; J. (2022). An expanded role for the transcription factor WRINKLED1 in the biosynthesis of triacylglycerols during seed development. Frontiers in Plant Scienc | Sobic.001G05250 | no | 73.26 | 70.54 | OTHER | 0.9975 OTHER 0.8309 | no | no | 7.7 |
| glycolysi | PFK | phosphofructokinas | AT5G5663 Manuscript (Kuczynski; C.; McCorkle; S.; Keereetaweep; J.; Shanklin; J.; & Schwender; J. (2022). An expanded role for the transcription factor WRINKLED1 in the biosynthesis of triacylglycerols during seed development. Frontiers in Plant Scienc | Sobic.009G07180 | no | 74.46 | 72.48 | OTHER | 0.9975 OTHER 0.6405 | no | no | 1481.7 |
| glycolysi | PFK | phosphofructokinas | AT5G5663 Manuscript (Kuczynski; C.; McCorkle; S.; Keereetaweep; J.; Shanklin; J.; & Schwender; J. (2022). An expanded role for the transcription factor WRINKLED1 in the biosynthesis of triacylglycerols during seed development. Frontiers in Plant Scienc | Sobic.010G04140 | arab | 80.39 | 78.44 | OTHER | 0.9975 OTHER 0.9984 | no | no | 1127.6 |
| glycolysi | PFK | phosphofructokinas | AT5G5663 Manuscript (Kuczynski; C.; McCorkle; S.; Keereetaweep; J.; Shanklin; J.; & Schwender; J. (2022). An expanded role for the transcription factor WRINKLED1 in the biosynthesis of triacylglycerols during seed development. Frontiers in Plant Scienc | Sobic.006G07480 | no | 41.14 | 43.79 | OTHER | 0.9975 OTHER 0.9991 | no | no | nd |
| glycolysi | PFK | phosphofructokinas | AT5G5663 Manuscript (Kuczynski; C.; McCorkle; S.; Keereetaweep; J.; Shanklin; J.; & Schwender; J. (2022). An expanded role for the transcription factor WRINKLED1 in the biosynthesis of triacylglycerols during seed development. Frontiers in Plant Scienc | Sobic.006G11470 | no | 51.41 | 49.56 | OTHER | 0.9975 OTHER 0.9997 | no | no | 43.1 |
| glycolysi | PFK | phosphofructokinas | AT5G6158 Manuscript (Kuczynski; C.; McCorkle; S.; Keereetaweep; J.; Shanklin; J.; & Schwender; J. (2022). An expanded role for the transcription factor WRINKLED1 in the biosynthesis of triacylglycerols during seed development. Frontiers in Plant Scienc | Sobic.001G25330 | no | 50.53 | 45.3 | cTP | 0.9021 OTHER 0.5076 | no | no | 696.7 |
| glycolysi | PFK | phosphofructokinas | AT5G6158 Manuscript (Kuczynski; C.; McCorkle; S.; Keereetaweep; J.; Shanklin; J.; & Schwender; J. (2022). An expanded role for the transcription factor WRINKLED1 in the biosynthesis of triacylglycerols during seed development. Frontiers in Plant Scienc | Sobic.002G20170 | no | 52.12 | 46.07 | cTP | 0.9021 cTP 0.8516 | no | no | 70.1 |
| glycolysi | PFK | phosphofructokinas | AT5G6158 Manuscript (Kuczynski; C.; McCorkle; S.; Keereetaweep; J.; Shanklin; J.; & Schwender; J. (2022). An expanded role for the transcription factor WRINKLED1 in the biosynthesis of triacylglycerols during seed development. Frontiers in Plant Scienc | Sobic.007G13950 | no | 51.74 | 42.83 | cTP | 0.9021 cTP 0.9939 | no | no | 16.3 |
| glycolysi | PFK | phosphofructokinas | AT5G6158 Manuscript (Kuczynski; C.; McCorkle; S.; Keereetaweep; J.; Shanklin; J.; & Schwender; J. (2022). An expanded role for the transcription factor WRINKLED1 in the biosynthesis of triacylglycerols during seed development. Frontiers in Plant Scienc | Sobic.009G20460 | no | 73.3 | 64.38 | cTP | 0.9021 mTP 0.9787 | ortholo | no | 3719.0 |
| glycolysi | PFK | phosphofructokinas | AT5G6158 Manuscript (Kuczynski; C.; McCorkle; S.; Keereetaweep; J.; Shanklin; J.; & Schwender; J. (2022). An expanded role for the transcription factor WRINKLED1 in the biosynthesis of triacylglycerols during seed development. Frontiers in Plant Scienc | Sobic.003G29000 | arab | 75.79 | 66.02 | cTP | 0.9021 OTHER 0.9998 | ortholo | no | 811.3 |
| glycolysi | PFK | phosphofructokinas | AT5G6158 Manuscript (Kuczynski; C.; McCorkle; S.; Keereetaweep; J.; Shanklin; J.; & Schwender; J. (2022). An expanded role for the transcription factor WRINKLED1 in the biosynthesis of triacylglycerols during seed development. Frontiers in Plant Scienc | Sobic.003G03470 | no | 68.88 | 67.59 | cTP | 0.9021 OTHER 0.9978 | ortholo | no | 6163.5 |
| glycolysi | PFK | phosphofructokinas | AT5G6158 Manuscript (Kuczynski; C.; McCorkle; S.; Keereetaweep; J.; Shanklin; J.; & Schwender; J. (2022). An expanded role for the transcription factor WRINKLED1 in the biosynthesis of triacylglycerols during seed development. Frontiers in Plant Scienc | Sobic.001G05250 | no | 72.5 | 65.16 | cTP | 0.9021 OTHER 0.8309 | ortholo | no | 7.7 |
| glycolysi | PFK | phosphofructokinas | AT5G6158 Manuscript (Kuczynski; C.; McCorkle; S.; Keereetaweep; J.; Shanklin; J.; & Schwender; J. (2022). An expanded role for the transcription factor WRINKLED1 in the biosynthesis of triacylglycerols during seed development. Frontiers in Plant Scienc | Sobic.009G07180 | no | 74.09 | 66.73 | cTP | 0.9021 OTHER 0.6405 | ortholo | no | 1481.7 |
| glycolysi | PFK | phosphofructokinas | AT5G6158 Manuscript (Kuczynski; C.; McCorkle; S.; Keereetaweep; J.; Shanklin; J.; & Schwender; J. (2022). An expanded role for the transcription factor WRINKLED1 in the biosynthesis of triacylglycerols during seed development. Frontiers in Plant Scienc | Sobic.010G04140 | no | 74.83 | 65.35 | cTP | 0.9021 OTHER 0.9984 | ortholo | no | 1127.6 |
| glycolysi | PFK | phosphofructokinas | AT5G6158 Manuscript (Kuczynski; C.; McCorkle; S.; Keereetaweep; J.; Shanklin; J.; & Schwender; J. (2022). An expanded role for the transcription factor WRINKLED1 in the biosynthesis of triacylglycerols during seed development. Frontiers in Plant Scienc | Sobic.006G07480 | no | 40.12 | 41.03 | cTP | 0.9021 OTHER 0.9991 | no | no | nd |
| glycolysi | PFK | phosphofructokinas | AT5G6158 Manuscript (Kuczynski; C.; McCorkle; S.; Keereetaweep; J.; Shanklin; J.; & Schwender; J. (2022). An expanded role for the transcription factor WRINKLED1 in the biosynthesis of triacylglycerols during seed development. Frontiers in Plant Scienc | Sobic.006G11470 | no | 50.82 | 47.35 | cTP | 0.9021 OTHER 0.9997 | no | no | 43.1 |
| glycolysi | PK | pyruvate kinase | AT5G6368 Manuscript (Kuczynski; C.; McCorkle; S.; Keereetaweep; J.; Shanklin; J.; & Schwender; J. (2022). An expanded role for the transcription factor WRINKLED1 in the biosynthesis of triacylglycerols during seed development. Frontiers in Plant Scienc | Sobic.006G26720 | yes | 83.27 | 83.73 | OTHER | 0.9995 OTHER 0.9985 | LDO | no | 1530.2 |
| glycolysi | PK | pyruvate kinase | AT5G6368 Manuscript (Kuczynski; C.; McCorkle; S.; Keereetaweep; J.; Shanklin; J.; & Schwender; J. (2022). An expanded role for the transcription factor WRINKLED1 in the biosynthesis of triacylglycerols during seed development. Frontiers in Plant Scienc | Sobic.001G38690 | no | 75.15 | 72.75 | OTHER | 0.9995 OTHER 0.9151 | no | no | nd |
| glycolysi | PK | pyruvate kinase | AT5G6368 Manuscript (Kuczynski; C.; McCorkle; S.; Keereetaweep; J.; Shanklin; J.; & Schwender; J. (2022). An expanded role for the transcription factor WRINKLED1 in the biosynthesis of triacylglycerols during seed development. Frontiers in Plant Scienc | Sobic.003G13080 | no | 77.8 | 76.68 | OTHER | 0.9995 OTHER 0.9974 | no | no | 5456.7 |
| glycolysi | FBP | fructose-1;6-bisphosphatas | AT5G6438 Manuscript (Kuczynski; C.; McCorkle; S.; Keereetaweep; J.; Shanklin; J.; & Schwender; J. (2022). An expanded role for the transcription factor WRINKLED1 in the biosynthesis of triacylglycerols during seed development. Frontiers in Plant Scienc | Sobic.010G21970 | bidirectiona | 74.78 | 70.11 | cTP | 0.5389 cTP 0.8923 | LDO | no | 588.1 |
| glyoxylate cycl | ICL | Isocitrate lyase | AT3G2172 http[s://www.uniprot.org/uniprotkb/P28297/ent](http://www.uniprot.org/uniprotkb/P28297/ent) | Sobic.002G32400 | bidirectiona | 77.07 | 78.11 | OTHER | 0.9995 OTHER 0.9997 | LDO | no | 26.4 |
| glyoxylate cycl | MS | Malate synthase | AT5G0386 http[s://www.uniprot.org/uniprotkb/Q56Z42/ent](http://www.uniprot.org/uniprotkb/Q56Z42/ent) | Sobic.006G12710 | bidirectiona | 74.59 | 74.11 | OTHER | 1.0000 OTHER 0.9992 | LDO | no | 82.5 |
| Intracellular transpor | NTT | plastidic ATP/ADP transporter | AT1G1550 Manuscript (Kuczynski; C.; McCorkle; S.; Keereetaweep; J.; Shanklin; J.; & Schwender; J. (2022). An expanded role for the transcription factor WRINKLED1 in the biosynthesis of triacylglycerols during seed development. Frontiers in Plant Scienc | Sobic.003G23740 | no | 73.89 | 70.43 | cTP | 0.9075 cTP 0.9677 | ortholo | no | 2539.9 |
| Intracellular transpor | NTT | plastidic ATP/ADP transporter | AT1G1550 Manuscript (Kuczynski; C.; McCorkle; S.; Keereetaweep; J.; Shanklin; J.; & Schwender; J. (2022). An expanded role for the transcription factor WRINKLED1 in the biosynthesis of triacylglycerols during seed development. Frontiers in Plant Scienc | Sobic.004G08750 | yes | 78.37 | 69.06 | cTP | 0.9075 cTP 0.8584 | ortholo | no | 3998.2 |
| Intracellular transpor | ABCAT | ABC Acyl Transporter | AT1G5435 Manuscript (Kuczynski; C.; McCorkle; S.; Keereetaweep; J.; Shanklin; J.; & Schwender; J. (2022). An expanded role for the transcription factor WRINKLED1 in the biosynthesis of triacylglycerols during seed development. Frontiers in Plant Scienc | Sobic.003G01710 | bidirectiona | 66.92 | 64.09 | cTP | 0.8277 cTP 0.7103 | LDO | no | 3916.3 |
| Intracellular transpor | GPT2 | chloroplast envelope phosphate antiporter family (TPT; PPT; GPT; XP | AT1G6180 Manuscript (Kuczynski; C.; McCorkle; S.; Keereetaweep; J.; Shanklin; J.; & Schwender; J. (2022). An expanded role for the transcription factor WRINKLED1 in the biosynthesis of triacylglycerols during seed development. Frontiers in Plant Scienc | Sobic.002G32200 | yes | 86.8 | 86.75 | cTP | 0.9324 OTHER 0.9254 | no | no | 34072.1 |
| Intracellular transpor | GPT2 | chloroplast envelope phosphate antiporter family (TPT; PPT; GPT; XP | AT1G6180 Manuscript (Kuczynski; C.; McCorkle; S.; Keereetaweep; J.; Shanklin; J.; & Schwender; J. (2022). An expanded role for the transcription factor WRINKLED1 in the biosynthesis of triacylglycerols during seed development. Frontiers in Plant Scienc | Sobic.007G06550 | bidirectiona | 78.12 | 74.27 | cTP | 0.9324 cTP 0.8866 | LDO | no | 1325.1 |
| Intracellular transpor | NTT | plastidic ATP/ADP transporter | AT1G8030 Manuscript (Kuczynski; C.; McCorkle; S.; Keereetaweep; J.; Shanklin; J.; & Schwender; J. (2022). An expanded role for the transcription factor WRINKLED1 in the biosynthesis of triacylglycerols during seed development. Frontiers in Plant Scienc | Sobic.003G23740 | yes | 81.95 | 69.88 | cTP | 0.8624 cTP 0.9677 | ortholo | no | 2539.9 |
| Intracellular transpor | NTT | plastidic ATP/ADP transporter | AT1G8030 Manuscript (Kuczynski; C.; McCorkle; S.; Keereetaweep; J.; Shanklin; J.; & Schwender; J. (2022). An expanded role for the transcription factor WRINKLED1 in the biosynthesis of triacylglycerols during seed development. Frontiers in Plant Scienc | Sobic.004G08750 | bidirectiona | 77.16 | 69.71 | cTP | 0.8624 cTP 0.8584 | ortholo | no | 3998.2 |
| Intracellular transpor | PPT | chloroplast envelope phosphate antiporter family (TPT; PPT; GPT; XP | AT3G0155 Manuscript (Kuczynski; C.; McCorkle; S.; Keereetaweep; J.; Shanklin; J.; & Schwender; J. (2022). An expanded role for the transcription factor WRINKLED1 in the biosynthesis of triacylglycerols during seed development. Frontiers in Plant Scienc | Sobic.002G15990 | no | 62.62 | 56.91 | cTP | 0.9776 cTP 0.7533 | ortholo | no | 3650.4 |
| Intracellular transpor | PPT | chloroplast envelope phosphate antiporter family (TPT; PPT; GPT; XP | AT3G0155 Manuscript (Kuczynski; C.; McCorkle; S.; Keereetaweep; J.; Shanklin; J.; & Schwender; J. (2022). An expanded role for the transcription factor WRINKLED1 in the biosynthesis of triacylglycerols during seed development. Frontiers in Plant Scienc | Sobic.004G35310 | yes | 63.69 | 57.5 | cTP | 0.9776 cTP 0.9904 | ortholo | no | 27067.6 |
| Intracellular transpor | PPT | chloroplast envelope phosphate antiporter family (TPT; PPT; GPT; XP | AT3G0155 Manuscript (Kuczynski; C.; McCorkle; S.; Keereetaweep; J.; Shanklin; J.; & Schwender; J. (2022). An expanded role for the transcription factor WRINKLED1 in the biosynthesis of triacylglycerols during seed development. Frontiers in Plant Scienc | Sobic.003G05080 | no | 60.19 | 54.42 | cTP | 0.9776 cTP 0.7297 | ortholo | no | 9568.9 |
| Intracellular transpor | PPT | chloroplast envelope phosphate antiporter family (TPT; PPT; GPT; XP | AT3G0155 Manuscript (Kuczynski; C.; McCorkle; S.; Keereetaweep; J.; Shanklin; J.; & Schwender; J. (2022). An expanded role for the transcription factor WRINKLED1 in the biosynthesis of triacylglycerols during seed development. Frontiers in Plant Scienc | Sobic.009G06250 | no | 58.31 | 53.93 | cTP | 0.9776 cTP 0.8904 | ortholo | no | 3833.1 |
| Intracellular transpor | FAX1 | Fatty Acid Export | AT3G5728 Manuscript (Kuczynski; C.; McCorkle; S.; Keereetaweep; J.; Shanklin; J.; & Schwender; J. (2022). An expanded role for the transcription factor WRINKLED1 in the biosynthesis of triacylglycerols during seed development. Frontiers in Plant Scienc | Sobic.002G05020 | yes | 46.06 | 42.06 | cTP | 0.9920 OTHER 0.7186 | LDO | no | 1175.3 |
| Intracellular transpor | FAX1 | Fatty Acid Export | AT3G5728 Manuscript (Kuczynski; C.; McCorkle; S.; Keereetaweep; J.; Shanklin; J.; & Schwender; J. (2022). An expanded role for the transcription factor WRINKLED1 in the biosynthesis of triacylglycerols during seed development. Frontiers in Plant Scienc | Sobic.006G11510 | bidirectiona | 50.97 | 41.29 | cTP | 0.9920 cTP 0.9847 | O | no | 791.8 |

| Intracellular transpor | ABCG4 | ABC Transporter | AT4G2575 Manuscript (Kuczynski; C.; McCorkle; S.; Keereetaweep; J.; Shanklin; J.; & Schwender; J. (2022). An expanded role for the transcription factor WRINKLED1 in the biosynthesis of triacylglycerols during seed development. Frontiers in Plant Scienc | Sobic.007G16240 | bidirectiona | 54.19 | 55.9 | cTP | 0.5267 OTHER 0.9997 | O | no | 39.9 |
| --- | --- | --- | --- | --- | --- | --- | --- | --- | --- | --- | --- | --- |
| Intracellular transpor | ABCG4 | ABC Transporter | AT4G2575 Manuscript (Kuczynski; C.; McCorkle; S.; Keereetaweep; J.; Shanklin; J.; & Schwender; J. (2022). An expanded role for the transcription factor WRINKLED1 in the biosynthesis of triacylglycerols during seed development. Frontiers in Plant Scienc | Sobic.009G12230 | no | 40.26 | 44.54 | cTP | 0.5267 OTHER 0.9999 | no | no | 22.1 |
| Intracellular transpor | ABCG4 | ABC Transporter | AT4G2575 Manuscript (Kuczynski; C.; McCorkle; S.; Keereetaweep; J.; Shanklin; J.; & Schwender; J. (2022). An expanded role for the transcription factor WRINKLED1 in the biosynthesis of triacylglycerols during seed development. Frontiers in Plant Scienc | Sobic.003G22000 | no | 43.11 | 44.42 | cTP | 0.5267 OTHER 0.9492 | no | no | 11.0 |
| Intracellular transpor | ABCG4 | ABC Transporter | AT4G2575 Manuscript (Kuczynski; C.; McCorkle; S.; Keereetaweep; J.; Shanklin; J.; & Schwender; J. (2022). An expanded role for the transcription factor WRINKLED1 in the biosynthesis of triacylglycerols during seed development. Frontiers in Plant Scienc | Sobic.003G38730 | no | 41.28 | 42.35 | cTP | 0.5267 OTHER 0.9996 | no | no | 19.2 |
| Intracellular transpor | PGLCT | plastidic glucose translocato | AT5G1615 Manuscript (Kuczynski; C.; McCorkle; S.; Keereetaweep; J.; Shanklin; J.; & Schwender; J. (2022). An expanded role for the transcription factor WRINKLED1 in the biosynthesis of triacylglycerols during seed development. Frontiers in Plant Scienc | Sobic.004G12450 | no | 46.68 | 44.35 | cTP | 0.7303 OTHER 0.9952 | no | yes | 2174.3 |
| Intracellular transpor | PGLCT | plastidic glucose translocato | AT5G1615 Manuscript (Kuczynski; C.; McCorkle; S.; Keereetaweep; J.; Shanklin; J.; & Schwender; J. (2022). An expanded role for the transcription factor WRINKLED1 in the biosynthesis of triacylglycerols during seed development. Frontiers in Plant Scienc | Sobic.003G08400 | bidirectiona | 83.01 | 74.4 | cTP | 0.7303 cTP 0.7447 | LDO | yes | 2549.3 |
| Intracellular transpor | PPT | chloroplast envelope phosphate antiporter family (TPT; PPT; GPT; XP | AT5G3332 Manuscript (Kuczynski; C.; McCorkle; S.; Keereetaweep; J.; Shanklin; J.; & Schwender; J. (2022). An expanded role for the transcription factor WRINKLED1 in the biosynthesis of triacylglycerols during seed development. Frontiers in Plant Scienc | Sobic.002G15990 | bidirectiona | 74.92 | 64.3 | cTP | 0.9908 cTP 0.7533 | LDO | yes | 3650.4 |
| Intracellular transpor | PPT | chloroplast envelope phosphate antiporter family (TPT; PPT; GPT; XP | AT5G3332 Manuscript (Kuczynski; C.; McCorkle; S.; Keereetaweep; J.; Shanklin; J.; & Schwender; J. (2022). An expanded role for the transcription factor WRINKLED1 in the biosynthesis of triacylglycerols during seed development. Frontiers in Plant Scienc | Sobic.004G35310 | yes | 73.6 | 64.91 | cTP | 0.9908 cTP 0.9904 | ortholo | yes | 27067.6 |
| Intracellular transpor | PPT | chloroplast envelope phosphate antiporter family (TPT; PPT; GPT; XP | AT5G3332 Manuscript (Kuczynski; C.; McCorkle; S.; Keereetaweep; J.; Shanklin; J.; & Schwender; J. (2022). An expanded role for the transcription factor WRINKLED1 in the biosynthesis of triacylglycerols during seed development. Frontiers in Plant Scienc | Sobic.003G05080 | yes | 64.06 | 54.5 | cTP | 0.9908 cTP 0.7297 | ortholo | yes | 9568.9 |
| Intracellular transpor | PPT | chloroplast envelope phosphate antiporter family (TPT; PPT; GPT; XP | AT5G3332 Manuscript (Kuczynski; C.; McCorkle; S.; Keereetaweep; J.; Shanklin; J.; & Schwender; J. (2022). An expanded role for the transcription factor WRINKLED1 in the biosynthesis of triacylglycerols during seed development. Frontiers in Plant Scienc | Sobic.009G06250 | yes | 64.54 | 56.43 | cTP | 0.9908 cTP 0.8904 | ortholo | yes | 3833.1 |
| Intracellular transpor | TPT | chloroplast envelope phosphate antiporter family (TPT; PPT; GPT; XP | AT5G4611 Manuscript (Kuczynski; C.; McCorkle; S.; Keereetaweep; J.; Shanklin; J.; & Schwender; J. (2022). An expanded role for the transcription factor WRINKLED1 in the biosynthesis of triacylglycerols during seed development. Frontiers in Plant Scienc | Sobic.009G08820 | yes | 75.62 | 71.17 | mTP | 0.8599 cTP 0.0031 | ortholo | no | 26.4 |
| Intracellular transpor | TPT | chloroplast envelope phosphate antiporter family (TPT; PPT; GPT; XP | AT5G4611 Manuscript (Kuczynski; C.; McCorkle; S.; Keereetaweep; J.; Shanklin; J.; & Schwender; J. (2022). An expanded role for the transcription factor WRINKLED1 in the biosynthesis of triacylglycerols during seed development. Frontiers in Plant Scienc | Sobic.003G00230 | bidirectiona | 76.2 | 72.36 | mTP | 0.8599 cTP 0.0042 | LDO | no | 111887. |
| Intracellular transpor | GPT1 | chloroplast envelope phosphate antiporter family (TPT; PPT; GPT; XP | AT5G5480 Manuscript (Kuczynski; C.; McCorkle; S.; Keereetaweep; J.; Shanklin; J.; & Schwender; J. (2022). An expanded role for the transcription factor WRINKLED1 in the biosynthesis of triacylglycerols during seed development. Frontiers in Plant Scienc | Sobic.002G32200 | no | 83.47 | 83.13 | cTP | 0.9639 OTHER 0.9254 | LDO | no | 34072.1 |
| Intracellular transpor | GPT1 | chloroplast envelope phosphate antiporter family (TPT; PPT; GPT; XP | AT5G5480 Manuscript (Kuczynski; C.; McCorkle; S.; Keereetaweep; J.; Shanklin; J.; & Schwender; J. (2022). An expanded role for the transcription factor WRINKLED1 in the biosynthesis of triacylglycerols during seed development. Frontiers in Plant Scienc | Sobic.007G06550 | yes | 76.82 | 74.32 | cTP | 0.9639 cTP 0.8866 | no | no | 1325.1 |
| lipoate synthesi | LT | Lipoyltransferase | AT1G0464 Manuscript (Kuczynski; C.; McCorkle; S.; Keereetaweep; J.; Shanklin; J.; & Schwender; J. (2022). An expanded role for the transcription factor WRINKLED1 in the biosynthesis of triacylglycerols during seed development. Frontiers in Plant Scienc | Sobic.001G38890 | bidirectiona | 64.29 | 61.28 | OTHER | 0.9345 OTHER 0.9847 | no | no | 43.5 |
| lipoate synthesi | LT | Lipoyltransferase | AT1G4757 Manuscript (Kuczynski; C.; McCorkle; S.; Keereetaweep; J.; Shanklin; J.; & Schwender; J. (2022). An expanded role for the transcription factor WRINKLED1 in the biosynthesis of triacylglycerols during seed development. Frontiers in Plant Scienc | Sobic.008G15200 | bidirectiona | 57.63 | 49.46 | OTHER | 0.6304 cTP 0.9987 | ortholo | no | 821.6 |
| lipoate synthesi | LS | Lipoate Synthase | AT2G2086 Manuscript (Kuczynski; C.; McCorkle; S.; Keereetaweep; J.; Shanklin; J.; & Schwender; J. (2022). An expanded role for the transcription factor WRINKLED1 in the biosynthesis of triacylglycerols during seed development. Frontiers in Plant Scienc | Sobic.006G03890 | no | 57.58 | 40.54 | mTP | 0.9909 OTHER 0.9536 | no | no | 1.9 |
| lipoate synthesi | LS | Lipoate Synthase | AT2G2086 Manuscript (Kuczynski; C.; McCorkle; S.; Keereetaweep; J.; Shanklin; J.; & Schwender; J. (2022). An expanded role for the transcription factor WRINKLED1 in the biosynthesis of triacylglycerols during seed development. Frontiers in Plant Scienc | Sobic.006G10310 | bidirectiona | 74.74 | 76.76 | mTP | 0.9909 mTP 0.6886 | LDO | no | 3607.3 |
| lipoate synthesi | LS | Lipoate Synthase | AT2G2086 Manuscript (Kuczynski; C.; McCorkle; S.; Keereetaweep; J.; Shanklin; J.; & Schwender; J. (2022). An expanded role for the transcription factor WRINKLED1 in the biosynthesis of triacylglycerols during seed development. Frontiers in Plant Scienc | Sobic.003G30930 | no | 55.84 | 51.29 | mTP | 0.9909 cTP 0.9670 | no | no | 3798.8 |
| lipoate synthesi | LT | Lipoyltransferase | AT4G3105 Manuscript (Kuczynski; C.; McCorkle; S.; Keereetaweep; J.; Shanklin; J.; & Schwender; J. (2022). An expanded role for the transcription factor WRINKLED1 in the biosynthesis of triacylglycerols during seed development. Frontiers in Plant Scienc | Sobic.008G15200 | no | 60.79 | 52.17 | OTHER | 0.9955 cTP 0.9987 | LDO | no | 821.6 |
| lipoate synthesi | LS | Lipoate Synthase | AT5G0841 Manuscript (Kuczynski; C.; McCorkle; S.; Keereetaweep; J.; Shanklin; J.; & Schwender; J. (2022). An expanded role for the transcription factor WRINKLED1 in the biosynthesis of triacylglycerols during seed development. Frontiers in Plant Scienc | Sobic.006G03890 | yes | 49.12 | 45.21 | cTP | 0.6061 OTHER 0.9536 | no | no | 1.9 |
| lipoate synthesi | LS | Lipoate Synthase | AT5G0841 Manuscript (Kuczynski; C.; McCorkle; S.; Keereetaweep; J.; Shanklin; J.; & Schwender; J. (2022). An expanded role for the transcription factor WRINKLED1 in the biosynthesis of triacylglycerols during seed development. Frontiers in Plant Scienc | Sobic.006G10310 | no | 57.1 | 50.95 | cTP | 0.6061 mTP 0.6886 | no | no | 3607.3 |
| lipoate synthesi | LS | Lipoate Synthase | AT5G0841 Manuscript (Kuczynski; C.; McCorkle; S.; Keereetaweep; J.; Shanklin; J.; & Schwender; J. (2022). An expanded role for the transcription factor WRINKLED1 in the biosynthesis of triacylglycerols during seed development. Frontiers in Plant Scienc | Sobic.003G30930 | bidirectiona | 74.92 | 68.07 | cTP | 0.6061 cTP 0.9670 | ortholo | no | 3798.8 |
| long chain FA synthesi | LACS2 | Long-Chain Acyl-CoA Synthetas | AT1G4943 Manuscript (Kuczynski; C.; McCorkle; S.; Keereetaweep; J.; Shanklin; J.; & Schwender; J. (2022). An expanded role for the transcription factor WRINKLED1 in the biosynthesis of triacylglycerols during seed development. Frontiers in Plant Scienc | Sobic.003G24200 | no | 54.81 | 48.24 | OTHER | 0.9975 OTHER 0.9898 | no | no | 6624.4 |
| long chain FA synthesi | LACS2 | Long-Chain Acyl-CoA Synthetas | AT1G4943 Manuscript (Kuczynski; C.; McCorkle; S.; Keereetaweep; J.; Shanklin; J.; & Schwender; J. (2022). An expanded role for the transcription factor WRINKLED1 in the biosynthesis of triacylglycerols during seed development. Frontiers in Plant Scienc | Sobic.003G25820 | no | 54.79 | 49.24 | OTHER | 0.9975 OTHER 0.9936 | no | no | 416.1 |
| long chain FA synthesi LACS2 Long-Chain Acyl-CoA Synthetas AT1G4943 Manuscript (Kuczynski; C.; McCorkle; S.; Keereetaweep; J.; Shanklin; J.; & Schwender; J. (2022). An expanded role for the transcription factor WRINKLED1 in the biosynthesis of triacylglycerols during seed development. Frontiers in Plant Scienc Sobic.005G15610 bidirectiona 62.08 48.18 OTHER 0.9975 OTHER 0.9989 LDO no nd long chain FA synthesi LACS2 Long-Chain Acyl-CoA Synthetas AT1G4943 Manuscript (Kuczynski; C.; McCorkle; S.; Keereetaweep; J.; Shanklin; J.; & Schwender; J. (2022). An expanded role for the transcription factor WRINKLED1 in the biosynthesis of triacylglycerols during seed development. Frontiers in Plant Scienc Sobic.006G24720 no 63.46 44.44 OTHER 0.9975 OTHER 0.8174 no no nd  long chain FA synthesi LACS2 Long-Chain Acyl-CoA Synthetas AT1G4943 Manuscript (Kuczynski; C.; McCorkle; S.; Keereetaweep; J.; Shanklin; J.; & Schwender; J. (2022). An expanded role for the transcription factor WRINKLED1 in the biosynthesis of triacylglycerols during seed development. Frontiers in Plant Scienc Sobic.006G24730 no 52.72 45.14 OTHER 0.9975 OTHER 0.9965 no no nd | | | | | | | | | | | | |

|  |  |  |  |  |  |  |  |  |  |  |  |  |
| --- | --- | --- | --- | --- | --- | --- | --- | --- | --- | --- | --- | --- |
|  |  |  |  |  |  |  |  |  |  |  |
|  |  |  |  |  |  |  |  |  |  |  |
| long chain FA synthesi | LACS2 | Long-Chain Acyl-CoA Synthetas | AT1G4943 Manuscript (Kuczynski; C.; McCorkle; S.; Keereetaweep; J.; Shanklin; J.; & Schwender; J. (2022). An expanded role for the transcription factor WRINKLED1 in the biosynthesis of triacylglycerols during seed development. Frontiers in Plant Scienc | Sobic.009G03140 | no | 46.66 | 62.42 | OTHER | 0.9975 OTHER 0.9999 | no | no | 459.6 |
| long chain FA synthesi long chain FA synthesi | LACS2 LACS2 | Long-Chain Acyl-CoA Synthetas Long-Chain Acyl-CoA Synthetas | AT1G4943 Manuscript (Kuczynski; C.; McCorkle; S.; Keereetaweep; J.; Shanklin; J.; & Schwender; J. (2022). An expanded role for the transcription factor WRINKLED1 in the biosynthesis of triacylglycerols during seed development. Frontiers in Plant Scienc AT1G4943 Manuscript (Kuczynski; C.; McCorkle; S.; Keereetaweep; J.; Shanklin; J.; & Schwender; J. (2022). An expanded role for the transcription factor WRINKLED1 in the biosynthesis of triacylglycerols during seed development. Frontiers in Plant Scienc | Sobic.010G04540 Sobic.010G04550 | no no | 54.7  53.62 | 48.46  48.3 | OTHER OTHER | 0.9975 OTHER 0.9953  0.9975 OTHER 0.9953 | no no | no no | 158.4  nd |
| long chain FA synthesi | LACS3 | Long-Chain Acyl-CoA Synthetas | AT1G6440 Manuscript (Kuczynski; C.; McCorkle; S.; Keereetaweep; J.; Shanklin; J.; & Schwender; J. (2022). An expanded role for the transcription factor WRINKLED1 in the biosynthesis of triacylglycerols during seed development. Frontiers in Plant Scienc | Sobic.003G24200 | yes | 69.68 | 69.47 | OTHER | 0.9995 OTHER 0.9898 | ortholo | no | 6624.4 |
| long chain FA synthesi | LACS3 | Long-Chain Acyl-CoA Synthetas | AT1G6440 Manuscript (Kuczynski; C.; McCorkle; S.; Keereetaweep; J.; Shanklin; J.; & Schwender; J. (2022). An expanded role for the transcription factor WRINKLED1 in the biosynthesis of triacylglycerols during seed development. Frontiers in Plant Scienc | Sobic.003G25820 | no | 67.17 | 66.57 | OTHER | 0.9995 OTHER 0.9936 | ortholo | no | 416.1 |
| long chain FA synthesi LACS3 Long-Chain Acyl-CoA Synthetas AT1G6440 Manuscript (Kuczynski; C.; McCorkle; S.; Keereetaweep; J.; Shanklin; J.; & Schwender; J. (2022). An expanded role for the transcription factor WRINKLED1 in the biosynthesis of triacylglycerols during seed development. Frontiers in Plant Scienc Sobic.005G15610 no 57.7 56.34 OTHER 0.9995 OTHER 0.9989 no no nd long chain FA synthesi LACS3 Long-Chain Acyl-CoA Synthetas AT1G6440 Manuscript (Kuczynski; C.; McCorkle; S.; Keereetaweep; J.; Shanklin; J.; & Schwender; J. (2022). An expanded role for the transcription factor WRINKLED1 in the biosynthesis of triacylglycerols during seed development. Frontiers in Plant Scienc Sobic.006G24720 no 70 64.81 OTHER 0.9995 OTHER 0.8174 no no nd  long chain FA synthesi LACS3 Long-Chain Acyl-CoA Synthetas AT1G6440 Manuscript (Kuczynski; C.; McCorkle; S.; Keereetaweep; J.; Shanklin; J.; & Schwender; J. (2022). An expanded role for the transcription factor WRINKLED1 in the biosynthesis of triacylglycerols during seed development. Frontiers in Plant Scienc Sobic.006G24730 no 63.92 59.52 OTHER 0.9995 OTHER 0.9965 ortholo no nd | | | | | | | | | | | | |

|  | | | | | | | | | | | | |
| --- | --- | --- | --- | --- | --- | --- | --- | --- | --- | --- | --- | --- |
|  |  |  |  |  |  |  |  |  |  |  |  |  |
|  |  |  |  |  |  |  |  |  |  |  |  |  |
|  |  |  |  |  |  |  |  |  |  |  |  |  |
| long chain FA synthesi LACS3 Long-Chain Acyl-CoA Synthetas AT1G6440 Manuscript (Kuczynski; C.; McCorkle; S.; Keereetaweep; J.; Shanklin; J.; & Schwender; J. (2022). An expanded role for the transcription factor WRINKLED1 in the biosynthesis of triacylglycerols during seed development. Frontiers in Plant Scienc Sobic.009G03140 no 50.15 49.25 OTHER 0.9995 OTHER 0.9999 no no long chain FA synthesi LACS3 Long-Chain Acyl-CoA Synthetas AT1G6440 Manuscript (Kuczynski; C.; McCorkle; S.; Keereetaweep; J.; Shanklin; J.; & Schwender; J. (2022). An expanded role for the transcription factor WRINKLED1 in the biosynthesis of triacylglycerols during seed development. Frontiers in Plant Scienc Sobic.010G04540 no 63.36 64.04 OTHER 0.9995 OTHER 0.9953 ortholo no  long chain FA synthesi LACS3 Long-Chain Acyl-CoA Synthetas AT1G6440 Manuscript (Kuczynski; C.; McCorkle; S.; Keereetaweep; J.; Shanklin; J.; & Schwender; J. (2022). An expanded role for the transcription factor WRINKLED1 in the biosynthesis of triacylglycerols during seed development. Frontiers in Plant Scienc Sobic.010G04550 no 62.75 63.43 OTHER 0.9995 OTHER 0.9953 ortholo no nd | | | | | | | | | | | | |

|  | | | | | | | | | | | | |
| --- | --- | --- | --- | --- | --- | --- | --- | --- | --- | --- | --- | --- |
|  |  |  |  |  |  |  |  |  |  |  |  |  |
|  |  |  |  |  |  |  |  |  |  |  |  |  |
|  |  |  |  |  |  |  |  |  |  |  |  |  |
| long chain FA synthesi LACS5 Long-Chain Acyl-CoA Synthetas AT4G1103 Manuscript (Kuczynski; C.; McCorkle; S.; Keereetaweep; J.; Shanklin; J.; & Schwender; J. (2022). An expanded role for the transcription factor WRINKLED1 in the biosynthesis of triacylglycerols during seed development. Frontiers in Plant Scienc Sobic.008G01080 no 40.62 40.22 OTHER 0.9987 OTHER 0.9940 no no long chain FA synthesi LACS5 Long-Chain Acyl-CoA Synthetas AT4G1103 Manuscript (Kuczynski; C.; McCorkle; S.; Keereetaweep; J.; Shanklin; J.; & Schwender; J. (2022). An expanded role for the transcription factor WRINKLED1 in the biosynthesis of triacylglycerols during seed development. Frontiers in Plant Scienc Sobic.009G03140 no 49.08 47.9 OTHER 0.9987 OTHER 0.9999 no no long chain FA synthesi LACS5 Long-Chain Acyl-CoA Synthetas AT4G1103 Manuscript (Kuczynski; C.; McCorkle; S.; Keereetaweep; J.; Shanklin; J.; & Schwender; J. (2022). An expanded role for the transcription factor WRINKLED1 in the biosynthesis of triacylglycerols during seed development. Frontiers in Plant Scienc Sobic.010G04540 no 64.23 64.66 OTHER 0.9987 OTHER 0.9953 ortholo no  long chain FA synthesi LACS5 Long-Chain Acyl-CoA Synthetas AT4G1103 Manuscript (Kuczynski; C.; McCorkle; S.; Keereetaweep; J.; Shanklin; J.; & Schwender; J. (2022). An expanded role for the transcription factor WRINKLED1 in the biosynthesis of triacylglycerols during seed development. Frontiers in Plant Scienc Sobic.010G04550 no 63 63.43 OTHER 0.9987 OTHER 0.9953 ortholo no nd | | | | | | | | | | | | |

long chain FA synthesi LACS4 Long-Chain Acyl-CoA Synthetas AT4G2385 Manuscript (Kuczynski; C.; McCorkle; S.; Keereetaweep; J.; Shanklin; J.; & Schwender; J. (2022). An expanded role for the transcription factor WRINKLED1 in the biosynthesis of triacylglycerols during seed development. Frontiers in Plant Scienc Sobic.010G04550 yes 65.41 64.66 OTHER 0.9996 OTHER 0.9953 ortholo no nd

|  | | | | | | | | | | | | 459.6 |
| --- | --- | --- | --- | --- | --- | --- | --- | --- | --- | --- | --- | --- |
|  |  |  |  |  |  |  |  |  |  |  |  | 158.4 |
| long chain FA synthesi | LACS9 | Long-Chain Acyl-CoA Synthetas | AT1G7759 Manuscript (Kuczynski; C.; McCorkle; S.; Keereetaweep; J.; Shanklin; J.; & Schwender; J. (2022). An expanded role for the transcription factor WRINKLED1 in the biosynthesis of triacylglycerols during seed development. Frontiers in Plant Scienc | Sobic.005G02030 | no | 64.23 | 63.86 | OTHER | 0.8444 OTHER 0.9998 | no | no | 5010.7 |
| long chain FA synthesi | LACS9 | Long-Chain Acyl-CoA Synthetas | AT1G7759 Manuscript (Kuczynski; C.; McCorkle; S.; Keereetaweep; J.; Shanklin; J.; & Schwender; J. (2022). An expanded role for the transcription factor WRINKLED1 in the biosynthesis of triacylglycerols during seed development. Frontiers in Plant Scienc | Sobic.005G05490 | bidirectiona | 70.49 | 71.06 | OTHER | 0.8444 SP 0.6476 | ortholo | no | 1849.4 |
| long chain FA synthesi | LACS9 | Long-Chain Acyl-CoA Synthetas | AT1G7759 Manuscript (Kuczynski; C.; McCorkle; S.; Keereetaweep; J.; Shanklin; J.; & Schwender; J. (2022). An expanded role for the transcription factor WRINKLED1 in the biosynthesis of triacylglycerols during seed development. Frontiers in Plant Scienc | Sobic.008G05130 | yes | 72.53 | 73.66 | OTHER | 0.8444 OTHER 0.8508 | LDO | no | 1586.4 |
| long chain FA synthesi | LACS8 | Long-Chain Acyl-CoA Synthetas | AT2G0435 Manuscript (Kuczynski; C.; McCorkle; S.; Keereetaweep; J.; Shanklin; J.; & Schwender; J. (2022). An expanded role for the transcription factor WRINKLED1 in the biosynthesis of triacylglycerols during seed development. Frontiers in Plant Scienc | Sobic.005G02030 | bidirectiona | 69.27 | 68.3 | OTHER | 0.9993 OTHER 0.9998 | LDO | no | 5010.7 |
| long chain FA synthesi | LACS8 | Long-Chain Acyl-CoA Synthetas | AT2G0435 Manuscript (Kuczynski; C.; McCorkle; S.; Keereetaweep; J.; Shanklin; J.; & Schwender; J. (2022). An expanded role for the transcription factor WRINKLED1 in the biosynthesis of triacylglycerols during seed development. Frontiers in Plant Scienc | Sobic.005G05490 | bidirectiona | 63.26 | 62.84 | OTHER | 0.9993 SP 0.6476 | no | no | 1849.4 |
| long chain FA synthesi | LACS8 | Long-Chain Acyl-CoA Synthetas | AT2G0435 Manuscript (Kuczynski; C.; McCorkle; S.; Keereetaweep; J.; Shanklin; J.; & Schwender; J. (2022). An expanded role for the transcription factor WRINKLED1 in the biosynthesis of triacylglycerols during seed development. Frontiers in Plant Scienc | Sobic.008G05130 | no | 64.17 | 64.13 | OTHER | 0.9993 OTHER 0.8508 | no | no | 1586.4 |
| long chain FA synthesi | LACS1 | Long-Chain Acyl-CoA Synthetas | AT2G4724 Manuscript (Kuczynski; C.; McCorkle; S.; Keereetaweep; J.; Shanklin; J.; & Schwender; J. (2022). An expanded role for the transcription factor WRINKLED1 in the biosynthesis of triacylglycerols during seed development. Frontiers in Plant Scienc | Sobic.003G24200 | no | 48.84 | 48.24 | OTHER | 0.9966 OTHER 0.9898 | no | no | 6624.4 |
| long chain FA synthesi | LACS1 | Long-Chain Acyl-CoA Synthetas | AT2G4724 Manuscript (Kuczynski; C.; McCorkle; S.; Keereetaweep; J.; Shanklin; J.; & Schwender; J. (2022). An expanded role for the transcription factor WRINKLED1 in the biosynthesis of triacylglycerols during seed development. Frontiers in Plant Scienc | Sobic.003G25820 | no | 49.77 | 49.24 | OTHER | 0.9966 OTHER 0.9936 | no | no | 416.1 |
| long chain FA synthesi LACS1 Long-Chain Acyl-CoA Synthetas AT2G4724 Manuscript (Kuczynski; C.; McCorkle; S.; Keereetaweep; J.; Shanklin; J.; & Schwender; J. (2022). An expanded role for the transcription factor WRINKLED1 in the biosynthesis of triacylglycerols during seed development. Frontiers in Plant Scienc Sobic.005G15610 no 48.7 48.18 OTHER 0.9966 OTHER 0.9989 no no nd long chain FA synthesi LACS1 Long-Chain Acyl-CoA Synthetas AT2G4724 Manuscript (Kuczynski; C.; McCorkle; S.; Keereetaweep; J.; Shanklin; J.; & Schwender; J. (2022). An expanded role for the transcription factor WRINKLED1 in the biosynthesis of triacylglycerols during seed development. Frontiers in Plant Scienc Sobic.006G24720 no 47.06 44.44 OTHER 0.9966 OTHER 0.8174 no no nd long chain FA synthesi LACS1 Long-Chain Acyl-CoA Synthetas AT2G4724 Manuscript (Kuczynski; C.; McCorkle; S.; Keereetaweep; J.; Shanklin; J.; & Schwender; J. (2022). An expanded role for the transcription factor WRINKLED1 in the biosynthesis of triacylglycerols during seed development. Frontiers in Plant Scienc Sobic.006G24730 no 49 45.14 OTHER 0.9966 OTHER 0.9965 no no nd  long chain FA synthesi LACS1 Long-Chain Acyl-CoA Synthetas AT2G4724 Manuscript (Kuczynski; C.; McCorkle; S.; Keereetaweep; J.; Shanklin; J.; & Schwender; J. (2022). An expanded role for the transcription factor WRINKLED1 in the biosynthesis of triacylglycerols during seed development. Frontiers in Plant Scienc Sobic.009G03140 bidirectiona 62.8 62.42 OTHER 0.9966 OTHER 0.9999 LDO no 459.6  long chain FA synthesi LACS1 Long-Chain Acyl-CoA Synthetas AT2G4724 Manuscript (Kuczynski; C.; McCorkle; S.; Keereetaweep; J.; Shanklin; J.; & Schwender; J. (2022). An expanded role for the transcription factor WRINKLED1 in the biosynthesis of triacylglycerols during seed development. Frontiers in Plant Scienc Sobic.010G04540 no 49.06 48.46 OTHER 0.9966 OTHER 0.9953 no no 158.4  long chain FA synthesi LACS1 Long-Chain Acyl-CoA Synthetas AT2G4724 Manuscript (Kuczynski; C.; McCorkle; S.; Keereetaweep; J.; Shanklin; J.; & Schwender; J. (2022). An expanded role for the transcription factor WRINKLED1 in the biosynthesis of triacylglycerols during seed development. Frontiers in Plant Scienc Sobic.010G04550 no 48.9 48.3 OTHER 0.9966 OTHER 0.9953 no no nd | | | | | | | | | | | | |

|  | | | | | | | | | | | | | |
| --- | --- | --- | --- | --- | --- | --- | --- | --- | --- | --- | --- | --- | --- |
|
|
|
|
|  |  |  |  |  |  |  |  |  |  |  |  |  |  |
|  |  |  |  |  |  |  |  |  |  |  |  |  |  |
|  |  |  |  |  |  |  |  |  |  |  |  |  |  |
|  |  |  |  |  |  |  |  |  |  |  |  |  |  |
|  |  |  |  |  |  |  |  |  |  |  |  |  |  |
|  |  |  |  |  |  |  |  |  |  |  |  |  |  |
|  |  |  |  |  |  |  |  |  |  |  |  |  |  |

|  | | | | | | | | | | | | 1066.1 |
| --- | --- | --- | --- | --- | --- | --- | --- | --- | --- | --- | --- | --- |
|  |  |  |  |  |  |  |  |  |  |  |  | 459.6 |
|  |  |  |  |  |  |  |  |  |  |  |  | 158.4 |
| long chain FA synthesi | LACS4 | Long-Chain Acyl-CoA Synthetas | AT4G2385 Manuscript (Kuczynski; C.; McCorkle; S.; Keereetaweep; J.; Shanklin; J.; & Schwender; J. (2022). An expanded role for the transcription factor WRINKLED1 in the biosynthesis of triacylglycerols during seed development. Frontiers in Plant Scienc | Sobic.003G24200 | yes | 72.15 | 71.6 | OTHER | 0.9996 OTHER 0.9898 | LDO | no | 6624.4 |
| long chain FA synthesi | LACS4 | Long-Chain Acyl-CoA Synthetas | AT4G2385 Manuscript (Kuczynski; C.; McCorkle; S.; Keereetaweep; J.; Shanklin; J.; & Schwender; J. (2022). An expanded role for the transcription factor WRINKLED1 in the biosynthesis of triacylglycerols during seed development. Frontiers in Plant Scienc | Sobic.003G25820 | no | 68.85 | 68.44 | OTHER | 0.9996 OTHER 0.9936 | ortholo | no | 416.1 |
[truncated: 51,333 more chars]
