# Supplementary material for: Development of vegetative oil sorghum: From lab‐to‐field
Source: Plant Biotechnol J. 2024 Nov 30;23(2):660–73. doi: 10.1111/pbi.14527 (PMC11772366; doi:10.1111/pbi.14527)
Supplement: Supplementary file 6 — Table S5 Primers used in this study. [file PBI-23-660-s005.docx]

Table S5. Primers used in this study. Primers for genotyping PCR and droplet digital PCR are listed.

| Primer name | Sequence |
| --- | --- |
| EIF4α ddPCR F | 5'-caactttgtcacccgcgatga-3' |
| EIF4α ddPCR R | 5'-tccagaaaccttagcagccca-3' |
| SbWri1 genotyping F | 5'-aagccgctgctagggcttac-3' |
| SbWri1 genotyping R | 5'-gattgatgctggagccgaag-3' |
| SbWri1 ddPCR F | 5'-gcgaggctaagacaccagac-3' |
| SbWri1 ddPCR R | 5'-gccgatattgctgtcgaaat-3' |
| AtDGAT1 genotyping ddPCR F | 5'-ggcagacaaggcaaatccag-3' |
| AtDGAT1 genotyping R | 5'-cctacaagggactgcgatgc-3' |
| AtDGAT1 ddPCR R | 5'-tgggttgatgtactgctcgat-3' |
| SiOle genotyping ddPCR F | 5'-cccacctccaactccaacct-3' |
| SiOle genotyping R | 5'-ggtctggctacctgcgactg-3' |
| SiOle ddPCR R | 5'-ccaggaggaaaatcgtgatg-3' |
| CpuDGAT1 genotyping F | 5'-atcctggtgaacgtggtcat-3' |
| CpuDGAT1 genotyping R | 5'-gagctcagccacaatgttca-3' |
| CvFatB1 genotyping ddPCR F | 5'- aacacctggttcagccagtc-3' |
| CvFatB1 genotyping ddPCR R | 5'- gtgtgggctatccacgaagt-3' |
| CvLPAT2 genotyping ddPCR F | 5'-ctgggcaatcctgtgaagat-3' |
| CvLPAT2 genotyping ddPCR R | 5'-agccagagcgtagagctgtc-3' |
| Thio14 genotyping ddPCR F | 5'- tcggaaggactcctgaaatg-3' |
| Thio14 genotyping ddPCR R | 5'-caaacagacgtggccctaat-3' |
| CpuLPATB genotyping ddPCR F | 5'-ctgggcaatcctgtgaagat-3' |
| CpuLPATB genotyping ddPCR R | 5'-agccagagcgtagagctgtc-3' |
