# Supplementary material for: Development of vegetative oil sorghum: From lab‐to‐field
Source: Plant Biotechnol J. 2024 Nov 30;23(2):660–73. doi: 10.1111/pbi.14527 (PMC11772366; doi:10.1111/pbi.14527)
Supplement: Supplementary file 7 — Data S1 Supporting experimental procedures. [file PBI-23-660-s002.docx]

**Supporting Experimental Procedures**

***Galactolipids and neutral lipids analysis***

The content of MGDG and DGDG in sorghum leaves was measured following the method of Cahoon and Ohlrogge (1994), with modifications to separate neutral lipids from MGDG. Fully expanded leaf tissues of field-grown sorghum plants were collected at soft-dough stage. Total lipid extracts from 20 mg of freeze-dried sorghum leaves (100 μg of C17:0 TAG was added at the beginning of extraction) were initially developed on TLC plates using a mobile phase of chloroform:methanol:acetic acid (75:25:8, v/v/v) until the solvent reached 3/4 of the plate. After drying, the TLC plate was re-developed to the top with a mobile phase of heptane:ethyl ether:acetic acid (70:30:1, v/v/v). MGDG, DGDG, and neutral lipids were visualized by primuline staining under UV light. The marked lipid fractions were scraped separately and 50 μg of C17:0 fatty acid (Nu-Check Prep) was added to MGDG and DGDG fractions as an internal standard. Lipid fractions were transesterified and then analyzed via GC-FID.

***Nonstructural carbohydrate measurement***

The content of sucrose, D-glucose, and starch in sorghum stems was measured using Sucrose/D-Glucose Assay Kit and Total Starch Assay Kit according to manufacturer’s protocol (Megazyme). Stem tissues of field-grown sorghum plants were collected at soft-dough stage. Lyophilized stem tissues were homogenized via TissueLyser II (Qiagen) with steel beads and 50 mg of tissue powder was used for each analysis.

***Gas exchange measurement***

Leaf-level gas exchange measurements were conducted on the third leaf below the flag leaf of oil sorghum events at the boot stage, grown in the field during the 2022 season. A LI-6400/XT system (LI-Cor Biosciences) was used for the analysis. The system was set to a photosynthetically active radiation (PAR) of 1500 µmol m⁻² s⁻¹, with reference CO₂ concentration at 400 ppm, and a flow rate of 500 µmol s⁻¹. Relative humidity was maintained below 70%. Measurements were taken on a sunny day between 10:00 AM and 12:00 PM.

***Acyl-ACP thioesterase activity assay***

Acyl-ACP thioesterase activity assay was examined from the leaves of GH-grown sorghum plants at the vegetative stage. Total crude protein extracts were prepared by grinding sorghum leaves in 100 mM Tris (pH 8.5) buffer with 1 mM EDTA. After centrifugation to remove cell debris, the enzyme activity assay was carried out using soluble protein extracts. Reactions (50 µl total volume) consisted of 50 mg of soluble protein extract with 1000 DPM [1-^14^C] acyl-ACP (myristoyl-ACP, 14:0 or oleoyl-ACP, 18:1Δ9; 55 mCi/mmol) in 100 mM Tris (pH 8.5) buffer at room temperature for 5 min. Both acyl-ACPs were enzymatically synthesized using recombinant spinach ACP (Rock and Garwin, 1979). After 5 min, the reaction was terminated by adding 50 µl of 1 M acetic acid in isopropanol. Free FAs produced by thioesterase were extracted with heptane saturated with 50% (v/v) isopropanol three times. Residual acyl-ACPs were collected from a lower phase of the reactant separately. Free FAs and unreacted acyl-ACPs were dried under nitrogen gas and re-dissolved in 15 ml of scintillation mixture (Bio-Safe II, Research Products International, Mt Prospect, IL). Radioactivity was measured using a liquid scintillation counter (Beckman Coulter LS6500; Beckman Coulter, Brea, CA).

***^13^C labeling***

Seeds for WT and transgenic *S. bicolor* to be used for ^13^C labeling were planted in 12-inch pots containing PRO-MIX BX soil and 30 g Osmocote Plus slow-release fertilizer pellets. They were grown in a greenhouse at 15-22°C with a 16/8 h photoperiod. [^13^C_2_]acetate labeling was performed on leaf discs (1.0 cm diameter, ~3.7 mg DW) excised from 9-week-old plants that each had about 10 attached leaves on the main stalk. The leaves used for labeling ranged from still expanding (~1 week old; denoted as Leaf 1) to starting to senesce (~6 weeks old; denoted as Leaf 5). Discs were incubated in 20 mM MES (pH 5.5), 2 mM CaCl_2_ under continuous 500 µmol/m^2^/s light (Mars Hydro) for 2 days. Labeling was terminated by freezing the discs in liquid nitrogen. To minimize endogenous lipase activity, frozen leaf discs were placed in hot isopropanol and incubated at 75°C for 15 min. After cooling to room temperature, 0.001% (w/v) butylated hydroxytoluene and an internal standard (methyl heptadecanoate) were added. Total lipids were then extracted with chloroform:methanol:water (30:41.5:3.5 v/v/v) according to the method (Shiva et al., 2018). Fatty acids were removed from the glyceryl backbone by transmethylation in 1 M HCl in methanol at 90°C for 1 hr, followed by phase-separation with hexane and water. The resulting FAMEs were quantified for total content and label enrichment by running on a 7890a GC/7000E Triple Quadrupole GC-MS (Agilent) with an Agilent DB-23 column (30 m x 0.25 mm internal diameter x 0.25 um film thickness) and an oven temperature ramp from 90°C to 245°C in 30.8 min. To quantify the concentration of fatty acids, methyl heptadecanoate was added prior to transmethylation to each sample as an internal standard. Quantification was done by peak area comparison with the internal standard using MassHunter Workstation Software (version B.03.01) to integrate GC–MS peak areas (total ion current). We quantified methyl esters of lauric (C12:0), myristic (C14:0), palmitic (C16:0), palmitoleic (C16:1), stearic (C18:0), oleic (C18:1), linoleic (C18:2) and linolenic acid (C18:3), being mostly abundant throughout the samples. In addition to quantification, mass isotopomer measurements for the fatty acid molecular ion of these fatty acids were made in single ion monitoring mode. After baseline subtraction, isotopomer abundances (MassHunter Workstation Software version B.03.01) were expressed as fractional abundances. The contribution of naturally occurring heavy isotopes of hydrogen and oxygen and of ^13^C in the methyl group was corrected by isotope distribution matrices that were computed and applied according to Allen and Ratcliffe (Allen and Ratcliffe, 2009). When applying the same corrections to fatty acid mass isotopomers in unlabeled control samples we noticed that the corrected M+1 peaks of the molecular ion (mass M) were slightly higher in abundance than expected for natural isotope abundance. This effect is small but consistent and results in the detection of ^13^C enrichment in unlabeled samples of less than 1% above the natural ^13^C. This type of measurement distortion in the mass isotopomer analysis of fatty acid methyl esters by GC/MS was previously described and analyzed (Fagerquist et al., 1999) . They concluded that, in the electron impact ionization process, a concentration dependent gas-phase reaction mechanism involving a proton transfer to the molecular ion is responsible for the bias in the observed isotopomer measurements. To correct for this effect, we applied an additional matrix correction to the labeled samples. For each fatty acid species, a correction matrix was obtained from the mass isotopomer distributions of unlabeled control samples, after they were corrected for contributions of natural heavy carbon, hydrogen and oxygen isotopes (natural abundance). Finally, after the combined correction steps the relative mass isotopomer abundances M_0_, M_1_, M_2_ … M_n_ were obtained (*n*, number of carbon atoms; M_i_, fractional molar abundance of the mass isotopomer containing *i* ^13^C-atoms). From this, the average ^13^C enrichment in fatty acid chains was derived as the weighted sum: ∑M*_i_ i* /*n*. To obtain the molar amount of ^13^C incorporated into a fatty acid, the natural abundance of ^13^C (1.07%) is subtracted from average ^13^C enrichment and the result is multiplied by the molar amount of the fatty acid and by the number of carbon atoms per fatty acid.

**References**

**Allen, D., and Ratcliffe, R.** (2009). Quantification of isotope label. Plant metabolic networks**,** 105-149.

**Cahoon, E.B., and Ohlrogge, J.B.** (1994). Apparent role of phosphatidylcholine in the metabolism of petroselinic acid in developing Umbelliferae endosperm. Plant physiology, **104,** 845-855.

**Fagerquist, C.K., Neese, R.A., and Hellerstein, M.K.** (1999). Molecular ion fragmentation and its effects on mass isotopomer abundances of fatty acid methyl esters ionized by electron impact. Journal of the American Society for Mass Spectrometry **10,** 430-439.

**Rock, C., and Garwin, J.** (1979). Preparative enzymatic synthesis and hydrophobic chromatography of acyl-acyl carrier protein. Journal of Biological Chemistry **254,** 7123-7128.

**Shiva, S., Enninful, R., Roth, M.R., Tamura, P., Jagadish, K., and Welti, R.** (2018). An efficient modified method for plant leaf lipid extraction results in improved recovery of phosphatidic acid. Plant Methods **14,** 1-8.
